# Systematic Dual Targeting of Dendritic Cell C-Type Lectin Receptor DC-SIGN and TLR7 Using a Trifunctional Mannosylated Antigen

**DOI:** 10.3389/fchem.2019.00650

**Published:** 2019-10-04

**Authors:** Rui-Jun Eveline Li, Tim P. Hogervorst, Silvia Achilli, Sven C. Bruijns, Tim Arnoldus, Corinne Vivès, Chung C. Wong, Michel Thépaut, Nico J. Meeuwenoord, Hans van den Elst, Herman S. Overkleeft, Gijs A. van der Marel, Dmitri V. Filippov, Sandra J. van Vliet, Franck Fieschi, Jeroen D. C. Codée, Yvette van Kooyk

**Affiliations:** ^1^Department of Molecular Cell Biology and Immunology, Cancer Center Amsterdam, Amsterdam Infection and Immunity Institute, Amsterdam Universitair Medische Centra, Vrije Universiteit Amsterdam, Amsterdam, Netherlands; ^2^Department of Bio-organic Synthesis, Faculty of Science, Leiden Institute of Chemistry, Leiden University, Leiden, Netherlands; ^3^Univ. Grenoble Alpes, CNRS, CEA, Institut de Biologie Structurale, Grenoble, France

**Keywords:** DC-SIGN, TLR7, glyco-antigen, vaccine model, peptide conjugate, tumor-associated antigens, mannoside

## Abstract

Dendritic cells (DCs) are important initiators of adaptive immunity, and they possess a multitude of Pattern Recognition Receptors (PRR) to generate an adequate T cell mediated immunity against invading pathogens. PRR ligands are frequently conjugated to tumor-associated antigens in a vaccination strategy to enhance the immune response toward such antigens. One of these PPRs, DC-SIGN, a member of the C-type lectin receptor (CLR) family, has been extensively targeted with Lewis structures and mannose glycans, often presented in multivalent fashion. We synthesized a library of well-defined mannosides (mono-, di-, and tri-mannosides), based on known “high mannose” structures, that we presented in a systematically increasing number of copies (*n* = 1, 2, 3, or 6), allowing us to simultaneously study the effect of mannoside configuration and multivalency on DC-SIGN binding via Surface Plasmon Resonance (SPR) and flow cytometry. Hexavalent presentation of the clusters showed the highest binding affinity, with the hexa-α1,2-di-mannoside being the most potent ligand. The four highest binding hexavalent mannoside structures were conjugated to a model melanoma gp100-peptide antigen and further equipped with a Toll-like receptor 7 (TLR7)-agonist as adjuvant for DC maturation, creating a trifunctional vaccine conjugate. Interestingly, DC-SIGN affinity of the mannoside clusters did not directly correlate with antigen presentation enhancing properties and the α1,2-di-mannoside cluster with the highest binding affinity in our library even hampered T cell activation. Overall, this systematic study has demonstrated that multivalent glycan presentation can improve DC-SIGN binding but enhanced binding cannot be directly translated into enhanced antigen presentation and the sole assessment of binding affinity is thus insufficient to determine further functional biological activity. Furthermore, we show that well-defined antigen conjugates combining two different PRR ligands can be generated in a modular fashion to increase the effectiveness of vaccine constructs.

**Graphical Abstract:**
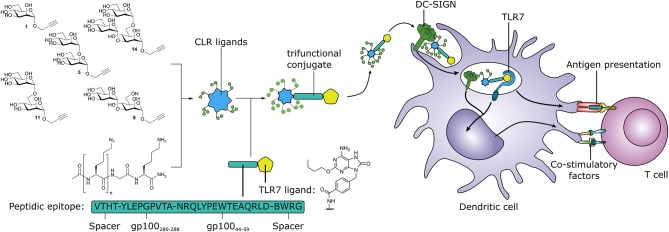
Synthetic multivalent presentation of CLR ligands on tumor antigens together with TLR7 agonist, provides DC targeting and induction of tumor specific T cells.

## Introduction

DC-SIGN (CD209) is an extensively studied receptor, due to its expression on various dendritic cell (DC) populations and its role in infection of certain viruses, like HIV (Bernardi et al., 2013; van Kooyk et al., 2013). This C-type Lectin Receptor (CLR) recognizes carbohydrate-based Pathogen-Associated Molecular Patterns (PAMPs) containing Lewis structures and high mannose glycans commonly found on bacteria, fungi and viruses (Geijtenbeek and Van Kooyk, 2003). DC-SIGN occurs on the cell surface as a tetramer, and therefore multivalent presentation of its carbohydrate ligand is favored for high affinity binding (van Kooyk et al., 2013).

**Figure 1:**
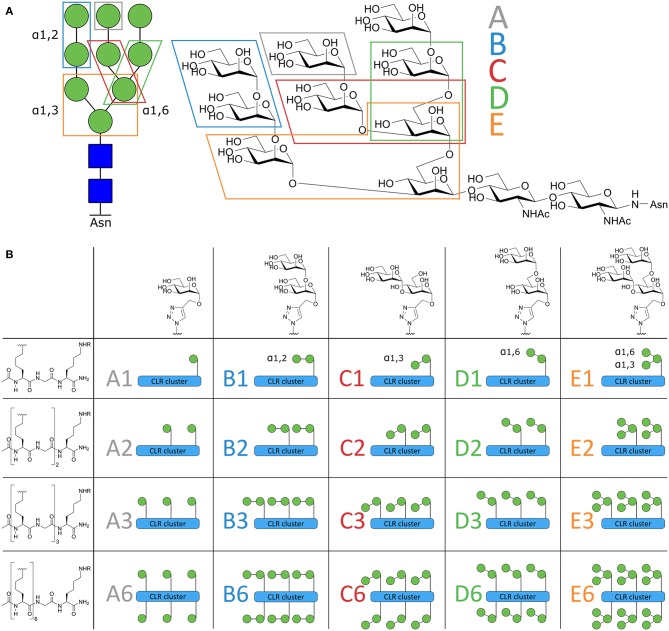
Schematic overview of the Mannose-cluster library. **(A)** Symbolic and chemical representation of the high affinity DC-SIGN ligand high mannose N-glycan Man_9_. The 5 mannoside configurations within Man9 are indicated in the figure. **(B)** From left to right, the 5 different oligomannosides. Top-down, the peptide backbone with increased valency. The total library of 20 mannoside clusters are illustrated as symbols and coded **A1–E6**.

Two strategies have been developed to target DC-SIGN using mannose-based ligands to deliver cargo to DCs. The first strategy uses mannosyl monosaccharides or analogs thereof, as these are readily available. Because the binding affinity of the mono-mannosides for DC-SIGN is relatively low with respect to larger and more complex oligo-mannosides (van Liempt et al., 2006), they are generally incorporated into dendrimers, liposomes or nanoparticles to achieve a multivalent presentation, enhancing the binding to DC-SIGN (Fehres et al., 2015; Ordanini et al., 2015; Silva et al., 2015; Berzi et al., 2016; Le Moignic et al., 2018). The other strategy uses larger and more complex oligomannosides with intrinsic multivalence. Various oligosaccharides have been explored for DC-SIGN binding (Ni et al., 2006; McIntosh et al., 2015), a prime example being the “high mannose” structure Man_9_ (Figure 1A). Both strategies have previously been used to deliver cancer antigens to DCs to enhance uptake and antigen presentation for the development of more effective cancer immunotherapies (Buskas et al., 2005; Moyle et al., 2007; Srinivas et al., 2007; McIntosh et al., 2015; Glaffig et al., 2018).

Another approach for the development of well-defined anti-cancer vaccines entails the covalent attachment of other adjuvants to the antigens of choice, targeting other Pattern Recognition Receptors (PRR), such as members of the Toll-Like Receptor (TLR) family (Deres et al., 1989; Cho et al., 2000; Blander and Medzhitov, 2006; Fujita and Taguchi, 2012; Willems et al., 2014), the NOD-like receptor (NLR) family (Willems et al., 2016), or combinations thereof (Buskas et al., 2005; Moyle et al., 2007; Sedaghat et al., 2016; Zom et al., 2019). PAMP recognition by TLRs induces DC maturation, stimulating antigen processing, and presentation for the induction of pathogen-specific T cells (Ackerman and Cresswell, 2003). The covalent attachment of a TLR agonist to an antigen can accelerate uptake and enhances antigen presentation while DC maturation *via* the TLR-ligands is maintained (Khan et al., 2007; Ignacio et al., 2018). Furthermore, it has been shown by simultaneous targeting of CLRs and TLRs, that CLR stimulation influences the TLR signaling cascades. For example, simultaneous triggering of DC-SIGN with TLR4 strengthens and prolongs TLR- signaling to enhance pro-inflammatory cytokine production in DCs (Fritz et al., 2005; Gringhuis et al., 2007). Since DC maturation is a necessity for upregulation of antigen processing and presentation, we hypothesized that a peptide-antigen conjugate, equipped with both a mannose-based DC-SIGN targeting glycan and a TLR-ligand, could lead to synergy in antigen presentation and improve specific T cell activation. We here describe the generation of such conjugate vaccine modalities, composed of a well-defined DC-SIGN targeting oligomannose cluster, a synthetic long peptide gp100 antigen, and a TLR7-agonist. TLR7 was selected as candidate due to its residency within the endosomes. We hypothesized that upon binding and internalization via DC-SIGN, the vaccine conjugate would be processed in endosomes where it can encounter TLR7. Using this strategy, we additionally avoid competition between binding of DC-SIGN and other cell surface TLRs.

Synthesizing high mannose structures is time and labor intensive and obtaining these structures in large quantities is challenging (Evers et al., 1998; Umekawa et al., 2008; Amin et al., 2011; Temme et al., 2013). We have therefore dissected the “high mannose” Man_9_-structure in smaller oligomannosides to explore which oligomannoside configurations could be used as a tool to effectively target the DC-SIGN receptor. To this end, we synthesized an array of oligomannose containing clusters (Figure 1B) that varied in number (ranging from 1 to 6 copies) and type of mannoside, each representing a part of the Man_9_ oligosaccharide (mono-; α1,2-di-; α1,3-di-; α1,6-di-; and an α1,3-α1,6-tri-mannoside, coded **A–E**, Figure 1A). This library has allowed us to compare side-by-side, the different mannoside configurations in different, well-defined clustered representations. The high affinity binders were used for conjugation to a model peptide antigen, containing the helper T cell epitope gp100_280−288_ and effector T cell epitope gp100_44−59_, to generate conjugates that could be targeted to DCs. To enhance the presentation of the antigens embedded in the conjugates by the DCs, we equipped the conjugates with a previously reported 8-oxo-adenosine analog (Jin et al., 2006; Gential et al., 2019), a ligand for the endosomal TLR7 which resulted in trifunctional conjugates (CLR-antigen-TLR). Using monocyte-derived dendritic cells (moDCs) we showed that the generated trifunctional conjugates represent attractive vaccine modalities that effectively targeted and activated DCs allowing effective antigen presentation.

## Results and Discussion

### Synthesis of the Oligomannoside Clusters

**Figure 2:**
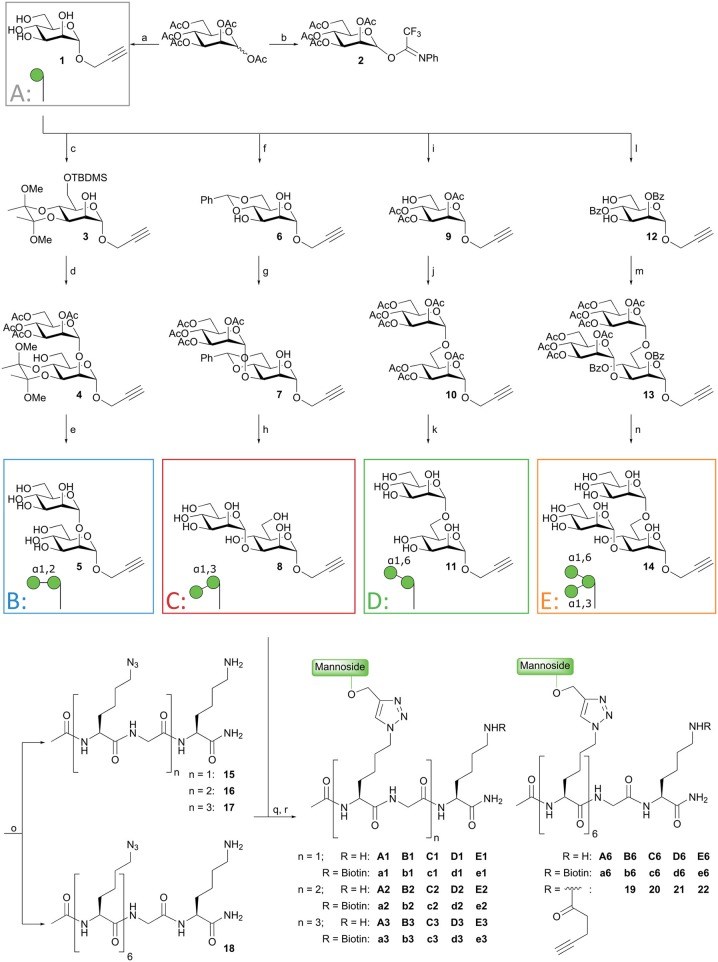
Synthesis of mannoside clusters. *Reagents and conditions:* (a) Daly et al. (2012); (b) Thomas et al. (2007); (c) *i*. 2,3-butanedione, HC(OMe)_3_, MeOH, CSA, reflux, 55%; *ii*. TBDMSCl, imidazole, DMF, 99%; (d) Donor **2**, TMSOTf, DCM, −20°C, 82%; (e) *i*. TFA, H_2_O, 71%; *ii*. NaOMe, MeOH, 82%; (f) PhCH(OMe)_2_, CSA, ACN, 50°C, 300 mbar, 51%; (g) Donor **2**, TMSOTf, DCM, −20°C, 80%; (h) *i*. AcOH, H_2_O, 95%; *ii*. NaOMe, MeOH, 95%; (i) *i*. Ph_3_CCl, imidazole, DCM, followed by Ac_2_O, pyridine, 90%; *ii*. BF_3_·Et_2_O, MeOH, toluene, 70%; (j) *i*. Donor **2**, TMSOTf, DCM, −20°C, 79%; (h) NaOMe, MeOH, 66%; (l) *i*. PhC(OMe)_3_, CSA, ACN; *ii*. H_2_O, 52%; (m) Donor **2**, TMSOTf, DCM, −25°C, 82%; (n) NaOMe, MeOH, 75% (see reference Wong et al., 2015); (o) Fmoc-SPPS; (q) CuI, THPTA, DIPEA, DMSO, H_2_O; (r) BiotinOSu or Pent-4-ynoic acid succinimidyl ester, DIPEA, DMSO.

The design of the oligomannoside cluster array is based on the Man_9_–N-glycan structure as depicted in Figure 1A and encompasses structures displaying 1, 2, 3, or 6 copies of mono-, di-, or trimannosides. The assembly of the array is shown in Figure 2 and it employs an oligo-azidolysine (6-azidonorleucine) backbone to which propargyl mannosides can be coupled. The required oligomannosides were all generated using propargyl α-D-mannopyranoside **1** as a starting compound (Daly et al., 2012). In order to keep the anomeric alkyne moiety intact, reductive transformations were avoided and acid/base labile protective groups were applied throughout the syntheses. Selective protection of the equatorial hydroxyls in **1** with a 1,2-butane diacetal, was followed by silylation of the primary hydroxyl to yield acceptor **3** in 54% yield over two steps. Glycosylation of **3** with imidate donor **2** (Thomas et al., 2007) provided the protected 1,2-linked disaccharide **4** in 82% yield. Acidic removal of the ketal and silyl ethers was followed by a basic deacetylation leading to α1,2-di-mannoside **6** in 58% yield over two steps. For the assembly of the 1,3-linked dimannoside, the higher nucleophilicity of the C-3-OH in **6** with respect to the neighboring, axial C-2-OH (van der Vorm et al., 2018) was exploited in a regio- and stereoselective glycosylation reaction. The condensation of acceptor **6** and donor **2** (Carpenter and Nepogodiev, 2005; Sauer et al., 2019) provided disaccharide **7** in 80% yield. Subsequent removal of the protective groups by sequential acid and base treatment resulted in α1,3-di-mannoside **8** in 90% yield over two steps. The 1,6-linked disaccharide was obtained by tritylation of the primary hydroxyl in propargyl mannopyranoside **1**, acetylation of secondary hydroxyls and trityl removal to give acceptor **9** in 63% yield over three steps. Glycosylation of **9** with donor **2** yielded disaccharide **10** in 79% yield, and after deacetylation α1,6-di-mannoside **11** was obtained in 66% yield. The set of propargyl mannnosides was completed with the previously described synthesis of tri-mannoside **14** from 2,4-di-*O*-benzoyl mannose acceptor **12**. This diol was mannosylated with two copies of donor **2** to yield the fully protected trisaccharide **13** in 82% yield, which was deacylated to effectively generate trimer **14** (Wong et al., 2015).

With the required propargyl mannosides in hand, the assembly of the array was undertaken. Using solid phase peptide synthesis (SPPS), four different backbones with 1, 2, 3, or 6 azides were synthesized for the attachment of the mannose clusters (**15–18**). To match the length of the hexavalent scaffold **18** with the trivalent backbone, glycine residues were incorporated in the latter scaffold to separate the azidolysines in **17**. Similarly, the azidolysines in the divalent scaffold **16** were also separated by a glycine residue. All backbones contained a lysine at the C-terminus for further functionalization. Via Cu(I) catalyzed Azide-Alkyne Cycloaddition (CuAAC) reactions the propargyl mannosides (**5**, **8**, **11, 14**) and peptides backbones (**15–18**) were clicked together. This resulted in a library of 20 well-defined structures (**A1–A6**; **B1–B6**; **C1–C6**; **D1–D6**; **E1–E6**, Figures 1B, 2), subdivided in five series in which the **A** series bears α-mannose **1**, the **B** series carries α1,2-di-mannoside **5**, the **C** series presents α1,3-di-mannoside **8**, the **D** series displays α1,6-di-mannoside **11**, and the **E** series is equipped with α1,3-α1,6-tri-mannoside **14**. All mannoside-clusters were equipped with a biotin handle for cellular assays by reacting the primary amine of the C-terminal lysine with Biotin-OSu to provide compounds **a1–e6**. For conjugation with the model epitope clusters the clusters **B6, C6, D6**, and **E6** were functionalized with an alkyne handle resulting in conjugation-ready compounds **19–22**.

### Binding Profile of the Mannoside Library

**Figure 3:**
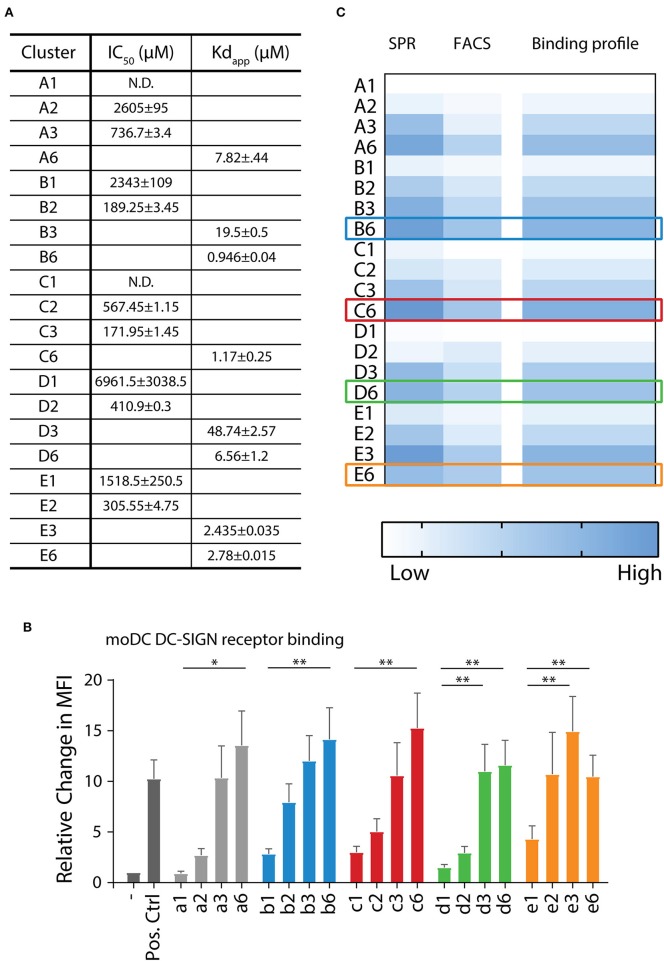
DC-SIGN has the highest affinity for α1,2-di-mannoside in a hexavalent configuration on multiple interaction levels. **(A)** Surface plasmon resonance (SPR) analysis demonstrates increase of affinity for DC-SIGN with increasing multivalence (*n* = 1 > 2 > 3 > 6). Clusters presenting the α1,2-di-mannoside **5** (**B** series) have the highest affinity in comparison to the other mannosides in this array. **(B)** Binding of the biotinylated mannoside library to DC-SIGN on moDC was measured by flow cytometry. Normalized to the unbound control, the clusters displayed increased binding with increasing multivalence. **(C)** Overall binding profile of the library, normalized to **A1**, indicating that the highest affinity binders are the **B6**, **C6**, **D6**, **E3**, and **E6** clusters. *p < 0.05 and **p < 0.01.

The affinity of the clusters for the extracellular domain (ECD) of the DC-SIGN receptor (DC-SIGN ECD) was estimated *via* surface plasmon resonance (SPR) assays (Tabarani et al., 2009). The apparent *K*_*d*_ was calculated in direct interaction mode using a surface functionalized in an oriented manner with DC-SIGN ECD. In this assay, tetrameric DC-SIGN ECD is attached to the surface of the sensor chip via the N-terminus of its neck oligomerization domain, thus presenting its four carbohydrate recognition domains toward the solvent, realistically mimicking the presentation of the receptor on cell surface (Porkolab et al., 2019). For some of the low affinity ligands, in the mM range, it was not possible to determine their affinity with this assay, and therefore a competition experiment was performed providing IC_50_ values (Timpano et al., 2008) (Figure 3A). A-specific interactions with the peptide backbone were excluded, since control clusters **G1** and **G2** (propargyl β-d-galactose clicked to backbones **15** and **16**) showed no interaction (Supplementary Figures 1A, 5). When comparing equivalent clusters, the α1,2-di-mannoside (**B** series) bound with the highest affinity (Figure 3A). Hexavalent presentation (*n* = 6) of the oligomannosides showed micromolar affinity toward DC-SIGN. **B6** had the highest affinity in the library with an apparent *K*_*d*_ of 0.95 μM, followed by the α1,3-dimannoside cluster **C6** (1.17 μM), and the trimannoside clusters **E3** (2.44 μM) and **E6** (2.78 μM). Interestingly, the affinity of the trisaccharide series (**E** series) did not improve going form the tri- to the hexavalent representation (**E3** vs. **E6**, Figure 3A). A potential explanation for this effect could be that the spacing of clusters is more important for the larger tri-mannosides. For the monovalent mannosides **A1** and **C1** we could not determine a reliable IC_50_ in this setup, indicating that their binding affinity for DC-SIGN is too weak (see Supplementary Figures 2, 3 for all SPR sensorgrams).

Next, we assessed the binding of our clusters to cellular DC-SIGN using moDCs (Figure 3B, Supplementary Figure 1B). To this end, binding of the clusters **a1**–**a6**, **b1**–**b6**, **c1**–**c6**, **d1**–**d6**, and **e1–e6**, decorated with a biotin handle, was determined by flow cytometry. Clusters were bound to moDCs for 30 min at 4°C. By staining using fluorophore-conjugated streptavidin and washing at 4°C, the bound clusters could be quantified by flow cytometry. Complementary to the SPR assays, the flow cytometric experiments revealed an enhancement in binding with increasing amount of mannosides for the **a**, **b**, and **c** series (Figure 3B). We observed significantly higher binding for the hexavalent mono-mannoside compared to the monovalent mannoside (**a1** vs. **a6**). These results are in agreement with earlier work suggesting DC-SIGN has a preference for high mannose like mannosides (Feinberg et al., 2007). The α1,2-mannosides (**b** series) showed enhanced binding in comparison to the mono-mannoside and the α1,6- or α1,3-dimannosides, in line with the SPR results and earlier results (Feinberg et al., 2007). In the cellular assay we also did not observe an increase in binding with an increasing number of tri-mannosides from the trivalent to the hexavalent cluster (**e3** vs. **e6**), in line with the SPR results. This result again illustrates the need to carefully consider the spacing between oligomannoses in multivalent mannoside clusters. The cellular assay also showed no increase in binding of the α1,6-dimannoside clusters when increasing the valency from **d3** to **d6**, again highlighting the potential influence of the scaffold design. In control experiments ligand binding to DC-SIGN was inhibited using a blocking anti-DC-SIGN antibody (Supplementary Figure 1C). Small residual binding remained, revealing a cluster-dependent increase in binding for the **a–c** series and a similar trend in binding of the **d**- and **e**-series. This suggests that other carbohydrate binding receptors, such as the mannose receptor, may play a role in binding the mannoside clusters (Raiber et al., 2010; He et al., 2015).

The binding profile of all mannose clusters is graphically summarized in Figure 3C. The strongest binding was observed for the hexavalent scaffolds, engaging DC-SIGN with μM affinity. The α1,2-di saccharide (**B** series) bound strongest and the monosaccharide (**A** series) bound with the lowest affinity. Therefore, we selected the **B6, C6, D6**, and **E6** clusters for conjugation to the melanoma gp100 antigen-TLR7 construct. Although cluster **E6** bound without affinity improvement for DC-SIGN comparing to **E3**, the former cluster was selected to allow for a direct comparison between the different clusters at the glycoconjugate level.

### Antigen and Adjuvant Conjugation

**Figure 4:**
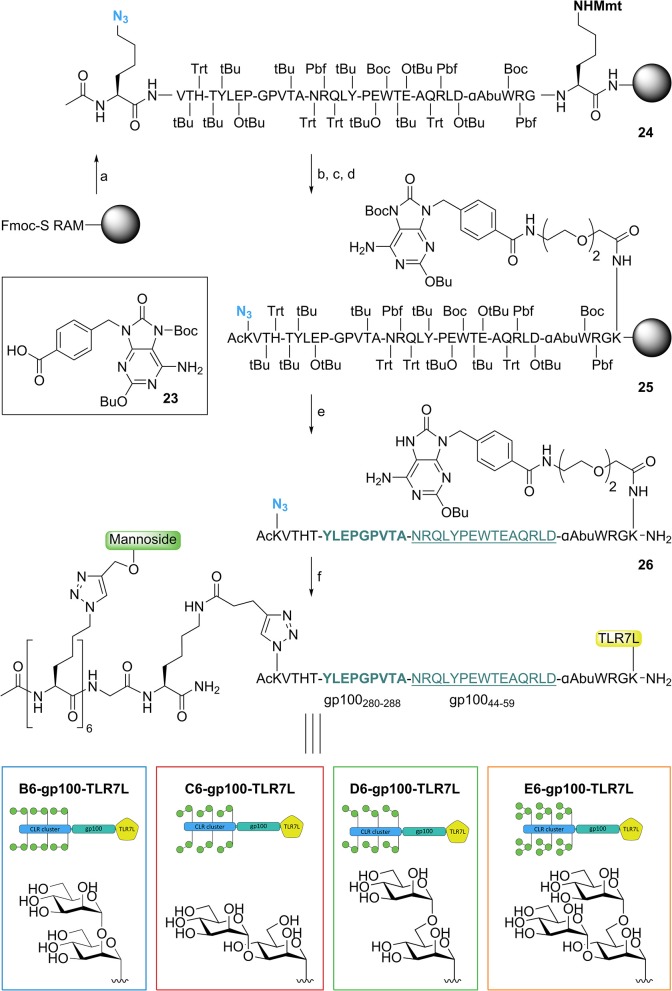
Synthesis of trifunctional CLR-epitope-TLR conjugates. *Reagents and conditions*: (a) Fmoc-SPPS; (b) TFA, DCM; (c) Fmoc-SPPS; (d) **23** (Gential et al., 2019), HCTU, DIPEA, DMF; (e) TFA, TIS, H_2_O, octanethiol, phenol; (f) CuI, THPTA, DIPEA, DMSO, H_2_O, (+ NaAsc, Arg, H_2_O), CLR clusters (**19–22**).

We next proceeded by synthesizing the mannose cluster-peptide-TLR7-agonist-conjugates via Fmoc-SPPS chemistry. Starting from Tentagel^®^ S RAM amide resin we coupled Fmoc-Lys(Mmt)-OH as the first amino acid to allow the conjugation of the TLR7 ligand after assembly of the peptide (see Figure 4). The gp100 peptide contains the gp100_280−288_ sequence for antigen presentation to CD4^+^ T cells connected to the N-terminus of the gp100_44−59_ sequence for CD8^+^ T cells. The epitope was elongated with four extra amino acids on each side to act as spacers. To prevent potential oxidation Cys_60_ was replaced by its isosteric analog α-amino-butyric acid (Wlodawer et al., 1989), which did not influence the antigen presentation of the peptide (Supplementary Figures 1G, 6). The peptide was elongated with Fmoc-Lys(N_3_)-OH followed by acetylation of the N-terminus resulting in **24**. C-terminal functionalization was achieved by selective removal of the Lys(Mmt) group, and subsequent coupling of an ethylene glycol spacer followed by introduction of the TLR7 agonist (Chan et al., 2009). Using our previously described protocol (Gential et al., 2019), we could introduce Boc protected TLR7-ligand **23** on-resin, resulting in functionalized solid support **25**. Release of the peptide-TLR7 ligand conjugate from the resin and concomitant global deprotection of the side chains under acidic conditions resulted in azido-peptide **26**, which was purified by HPLC. Control peptides lacking the N-terminal azide and/or the TLR7 ligand were synthesized to investigate the effect of the CLR clusters (**gp100** and **gp100-TLR7L**, see Supplementary Figure 6).

Attempts to synthesize gp100 peptides elongated with six azidolysines through SPPS proved to be troublesome and therefore we used a modular approach in which the pre-assembled CLR clusters (**19**–**22**) (Figure 2) were ligated to TLR7-peptide conjugate **26** via a CuAAC click reaction (Figure 4) (Conibear et al., 2016). This resulted in four conjugates containing a TLR7 agonist and a hexavalent α1,2-dimannnoside cluster (**B6-gp100-TLR7L**); an α1,3-dimannoside cluster (**C6-gp100-TLR7L**); an α1,6-dimannoside cluster (**D6-gp100-TLR7L**); or an α1,3-α1,6-trimannoside cluster (**E6-gp100-TLR7L**) that could be tested for their antigen presenting capacities.

### Targeting Efficacy of the Mannoside-Peptide Conjugates

**Figure 5:**
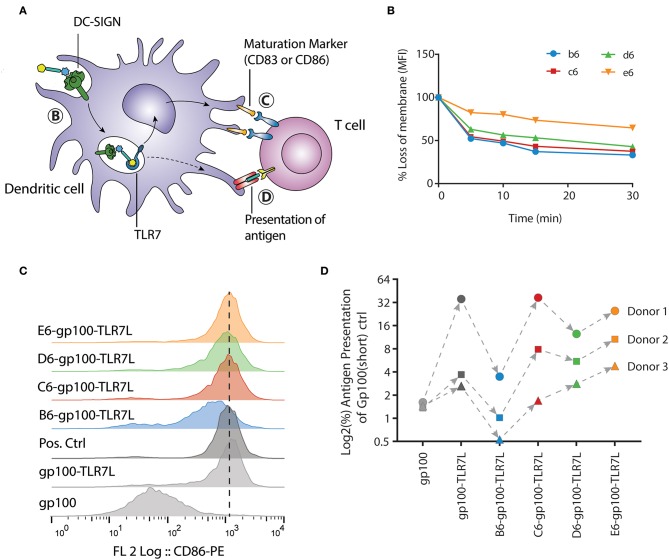
E-gp100-TLR7L increases antigen presentation of monocyte-derived dendritic cells despite slower internalization. **(A)** Schematic representation of the moDC readouts used. **(B)** The internalization of the hexavalent mannoside clusters by moDCs was measured by flow cytometry. One donor is depicted here as representation of four individual clusters **b6**, **c6**, **d6** are rapidly internalized, while **e6** remains longer at the surface. **(C)** Expression of the DC maturation marker CD86 upon overnight stimulation with the trifunctional conjugates is measured by flow cytometry. All compounds harboring the mannoside cluster and a TLR7 ligand induce expression of CD86. LPS stimulation (10 ng/mL) is used as positive control. **(D)** Antigen presentation by the moDCs was determined by IFNγ release of the activated T cells. The **B6-gp100-TLR7L** conjugate shows minimal T cell activation while the **C6**-, **D6**-, and **E6**-**gp100-TLR7L** conjugates increased T cell activation compared to **gp100-TLR7L**. Overall, conjugate **E6-gp100-TLR7L** showed the strongest antigen presentation enhancement by moDCs.

Immature dendritic cells are present in the peripheral tissue, acting as the first-line of defense against pathogens. In immature state, dendritic cells are optimized for phagocytosis of extracellular material and antigens. Upon maturation, triggered by e.g., pathogenic stimuli that activates TLRs, phagocytic processes are downregulated, while co-stimulatory molecules for T cell activation are upregulated and antigen presentation is enhanced (Ackerman and Cresswell, 2003). After the trifunctional peptides are internalized, the maturation process prepares the dendritic cell for optimal antigen presentation to T cells. To assess the efficacy of the selected compounds, we analyzed different biological processes that the trifunctional peptides are routed through. First, the uptake of biotinylated clusters by immature DCs was measured, followed by the ability of the trifunctional conjugates to induce DC maturation, and lastly the capability of the gp100 epitopes to be presented (Figure 5A).

Among the various conjugates produced, two could be evaluated by SPR for their binding properties to ensure that conjugating the mannose-cluster to gp100 alone or gp100-TLR7L modules do not mask their accessibility for DC-SIGN recognition. **B6-gp100** and **E6-gp100-TLR7L** are still binders with μM affinities of DC-SIGN surfaces, however the conjugation to either gp100 or gp100-TLR7L module decreased this affinity by a factor of about 10, suggesting that gp100 conjugation reduced binding somewhat (Comparing Figure 3A and Supplementary Figure 1A, the *K*_*d*__−app_ goes from 0.9 to 10.6 μM for **B6** to **B6-gp100**, and for **E6** to **E6-gp100-TLR7L** the *K*_*d*__−app_ goes from 2.7 to 31 μM, each time increasing by about a factor 10, Supplementary Figure 3).

For the internalization, the moDCs were incubated with clusters **b6, c6, d6**, and **e6** for 1 h at 4°C, where after unbound ligands were washed away with ice-cold medium. Warm medium was added to the moDCs, and samples were taken at the indicated time points and put on ice. Upon staining the moDCs with fluorophore-conjugated streptavidin, we could measure the signal loss of the membrane *via* flow cytometry. To exclude ligand-receptor dissociation before internalization, the moDCs were fixed under gentle conditions, hereby inhibiting receptor-mediated endocytosis. On fixed moDCs the clusters remained at the surface, as no signal loss could be detected (Supplementary Figure 1D). Notably, the uptake of clusters **b6, c6, d6**, and **e6** by immature moDCs did not correlate with their affinity for DC-SIGN (Figures 3B, 5B). The di-mannoside clusters (**b6**, **c6**, and **d6**) were internalized relatively fast, with a 50% uptake within 5 min (Figure 5B). The tri-mannoside cluster **e6** however remained longer at the membrane surface and only a 25% uptake was seen after 30 min. Similar results were seen using a pH-sensitive fluorophore. In acidic environments, such as the endosomes and lysosomes, the fluorescence of this dye increases. Pre-complexed clusters mirrored the accelerated uptake of the di-mannoside clusters over the **e6** cluster (Supplementary Figure 1E). Although the DC-SIGN mediated uptake mechanism is known (Cambi et al., 2009), the initiation trigger for endocytosis upon DC-SIGN-ligand binding remains unclear. Recognition of the di-mannoside clusters could induce signaling leading to accelerated uptake, whereas the tri-mannoside cluster could trigger a different signaling pathway. Nonetheless, the clusters harboring the smaller mannosides are preferred. Although the binding affinity of the clusters ranged from 0.9 to 6.6 μM, the di-mannosides induce more rapid DC-SIGN internalization over the tri-mannosides, and can thus increase intracellular peptide concentrations more efficiently for further antigen processing and presentation.

Next, the four trifunctional-conjugates were evaluated for their ability to mature moDCs and compared to non-mannosylated conjugates **gp100** and **gp100-TLR7L**. Maturation of the moDCs was measured by the expression of CD86 and CD83, two costimulatory molecules necessary for T cell activation. As expected, **gp100**, lacking the TLR7 agonist, did not induce maturation of the moDCs. All the conjugates with the mannoside clusters and the TLR7 ligand induced the expression of the moDCs maturation marker CD86 (Figure 5C), as well as CD83 (Supplementary Figure 1F) compared to the TLR7 agonist lacking gp100 control, indicating the DCs potential to activate T cells. These results show that the conjugation of TLR7 ligand to the peptide antigen or the peptide-mannose cluster conjugates does not hamper the TLR activating ability of the ligand.

Lastly, antigen presentation was determined in a human T cell antigen presentation model (Figure 5D). In this assay, the potency of DCs to present the internalized gp100 antigen on the cell surface to gp100-specific T cells is analyzed. The IFNγ secretion of activated T cells upon recognition of the cell surface presented gp100 is measured. Day 5 moDCs were stimulated for 30 min, as the internalization was relatively fast, with the glycopeptides at the assay-optimized concentration of 20 μM (Supplementary Figure 1H). The constructs were washed away before overnight co-culture with gp100-specific T cells. Although we observed significant natural donor variability, all conjugates showed similar mannoside-dependent trends in response. Surprisingly, although the α1,2-di-mannoside cluster **B6** showed the strongest binding affinity and **b6** was internalized rapidly, **B6-gp100-TLR7L** did not enhance antigen presentation. The obstruction of antigen presentation was primarily seen with the **B6-gp100-TLR7L** contradicting the assumption that the best binding constructs will simultaneously maximize T cell activation. The other di-mannoside conjugates (**C6-gp100-TLR7L** & **D6-gp100-TLR7L**) performed better in the antigen presentation assay than **B6-gp100-TLR7L**. **E6-gp100-TLR7L** demonstrated the strongest antigen presentation in the three donors tested. With some exceptions in donor 1, the antigen presentation was readily enhanced compared to **gp100-TLR7L** and all other constructs within each donor, even though the binding affinity of **E6** was a 3-fold lower than **B6**. The enhancement in antigen presentation of tri-mannoside conjugate **E6-gp100-TLR7L** may be a result of the stagnant uptake of tri-mannoside clusters (Figure 5B), altered DC-SIGN signaling and/or different intracellular routing or processing of the conjugates, compared to the di-mannosides. This indicates that oligosaccharides with high binding affinity for DC-SIGN are not *per se* the most suitable for use in covalent saccharide-antigen conjugates, designed for optimal antigen presentation and that the size of the clusters may affect the rate by which peptidases trim the conjugates enabling loading on MHC molecules for presentation.

It has previously been reported that a negative correlation between high internalization efficiency and antigen presentation may be due to altered intracellular processing (Chatterjee et al., 2012) and that DC-SIGN endocytosed ligands can traffic to differential endosomal compartments upon internalization. The trifunctional conjugates here are assumed to dissociate from the DC-SIGN receptor in the early endosomes, to allow triggering of TLR7 and enable their processing (Engering et al., 2002; Wilson et al., 2019). Our data may be explained by differential routing of the conjugates or differences in processing efficiency (Chatterjee et al., 2012). Alternatively, binding of the clusters to different mannose binding lectins, may also impact uptake and routing. In this regard, the mannose receptor could be a contributing inhibitory factor as high affinity binding of the smaller mannoside clusters to this receptor is known to prohibit ligand-receptor dissociation, halting further antigen processing in the early endosomes (Hiltbold et al., 2000). Future experiments will have to shed light on how and where conjugates of this type are processed, to enable the further optimization of rationally designed self-adjuvating peptide vaccines.

## Conclusion

With the field of dendritic cell-based immunotherapy in acceleration, the range of glycoconjugates aimed at modulating the dendritic cell phenotype has rapidly expanded (Hotaling et al., 2014). Our paper documents a systematic array of DC-SIGN-targeting clusters, with well-defined mannoside structures (mono-, di-, and tri-mannosides) and controlled (mono-, di-, tri-, hexavalent) presentation. From this array, we have identified multiple hexavalent ligands that bind DC-SIGN with micromolar affinity, with the α1,2-dimannoside cluster **B6** being the best binder. The hexavalent clusters were conjugated to a model antigen and a TLR7 agonist, and tested for their ability to mature DCs and to enhance antigen presentation. Conjugation of the peptide to the sugar clusters does not hamper their binding to DC-SIGN nor does the conjugation impede TLR7 activation. Improved antigen presentation was observed for three of the four conjugates that were equipped with a TLR7 ligand and a mannoside cluster. Surprisingly, the conjugate harboring the highest affinity DC-SIGN binder, **B6**, showed lower antigen presentation that its **C6**-, **D6**, and **E6**-counterparts. This indicates that the affinity for DC-SIGN of particular mannoside clusters does not directly translate into enhanced antigen presentation of conjugates equipped with the clusters. Differences in processing pathways and speed of the multifunctional conjugates have to be taken into account and future research will be directed at mapping the events between uptake and presentation to enable the design of the next generation vaccine conjugates with tailor made activity. Taking natural variations between donors into account, the **E6-gp100-TLR7L** conjugate showed the best T cell activating properties and will serve as a lead for further conjugate development. The modular chemistry developed here, allows the future design of conjugates bearing multiple PRR ligands in a single molecule. The multivalent presentation of a TLR ligand may enhance the activation of its cognate TLR, and different PRR-ligands may be combined to achieve synergistic activation of DCs, for example by exploiting simultaneous TLR and NLR activation (Ignacio et al., 2018).

## Materials and Methods

### General Synthesis

The brief general synthetic procedures are described below, comprehensive experimental descriptions and analytical spectra for each construct can be found in the Supplementary Materials section.

The solid-phase peptide synthesis of the azidolysine backbones was performed on a TRIBUTE® Peptide Synthesizer (Gyros Protein Technologies AB, Arizona, USA) applying Fmoc based protocol starting with Tentagel S-RAM resin (~0.22 mmol/g) on a 100 μmol scale using established synthetic protocols (Chan and White, 2000).

For the conjugation of propargyl glycosides and azidopeptides, all solvents were degassed by sonicating while bubbling argon through the solutions. A solution of azidopeptides in DMSO (0.5 M, 1 eq) was mixed with a solution of propargyl glycoside in water (0.5 M, 1.2 eq per azide) followed by addition of an aliquot of a stock solution of CuI (0.1 eq), THPTA (0.3 eq), and DIPEA (0.2 eq) in water ([Cu^+^] = 0.5 M). The reaction was stirred at 40°C and the process was followed via LC-MS. When reactions did not progress and turned blue, a sodium ascorbate solution (0.2–1 eq, 1 M, aq) was added. Generally, reactions were stirred overnight at 40°C. When not complete after 16 h an extra aliquot of the copper stock was added. After completion a small amount of Quadrasil® AP (washed with water) was added, stirred for 1 h, filtered and applied on gel filtration (Toyopearl HW40S, 150 mM NH_4_HCO_3_, 1.6 × 60 cm, 1 mL/min) followed by lyophilization.

To introduce the biotin handle, glycoclusters (**A1–E6**) were dissolved in DMSO (0.02 M). To this, a stock solution of Biotin-OSu (0.15 M, 3–4 eq) and DIPEA (0.015 M, 0.3–0.4 eq) in DMSO were added and shaken overnight after which compounds were purified via RP-HPLC (linear gradient 10–16 % B in A, 12 min, 5 mL/min, Develosil RPAQUEOUS 10.0 × 250 mm) followed by lyophilization.

For the synthesis of alkyne labeled clusters **19–22**, a solution of glycoclusters (**B6, C6, D6**, or **E6**) in water (0.2 M, 1 eq) was mixed with a stock solution of **S9** (0.15 M, 3 eq) and DIPEA (0.05 M, 1 eq) in DMSO and shaken 1 h. Reaction progress was followed via LC-MS and when completed, the 4-pentynoic amides were purified via gel filtration (Toyopearl HW-40S, 1.6 × 60 cm, 150 mM NH_4_HCO_3_, 1 mL/min) or RP-HPLC followed by lyophilization.

The solid-phase peptide synthesis of the gp100 peptides was performed on a TRIBUTE® Peptide Synthesizer (Gyros Protein Technologies AB, Arizona, USA) applying Fmoc based protocol starting with Tentagel S-RAM resin (~0.22 mmol/g) on a 100–250 μmol scale using established synthetic protocols (Chan and White, 2000). The consecutive steps for synthesis on 250 μmol scale^*^ performed in each cycle were:

*(1)* DMF wash (1x) followed by nitrogen purge; *(2)* Deprotection of the Fmoc-group with 20% piperidine in DMF (8 mL) (3 × 3 min at 50°C); *(3)* DMF wash (3x) followed by nitrogen purge; *(4.1)* Coupling of the appropriate amino acid^**^ in 4-fold excess (unless stated otherwise)^***^; *(4.2)* Step 4.1 was repeated; *(5)* DMF wash (3x) followed by nitrogen purge; *(6)* capping with a solution of Ac_2_O/DMF/DIPEA (8 mL, 10/88/2, v/v/v) for 2 min; *(7)* DMF wash (2x).

After the complete sequence the resin was washed with DMF (3x), DCM (3x), Et_2_O (3x), followed by nitrogen purge before treatment with the cleavage cocktail.

^*^All amounts are scaled-down in equimolar proportions for smaller scale.

^**^The amino acids applied in this synthesis were: Fmoc-Lys(Mmt)-OH, Fmoc-Gly-OH, Fmoc-Arg(Pbf)-OH, Fmoc-Trp(Boc)-OH, Fmoc-L-α-aminobutyric acid, Fmoc-Asp(O*t*Bu)-OH^****^, Fmoc-Leu-OH^****^, Fmoc-Gln(Trt)-OH, Fmoc-Ala-OH, Fmoc-Glu(O*t*Bu)-OH, Fmoc-Thr(*t*Bu)-OH, Fmoc-Pro-OH, Fmoc-Tyr(*t*Bu)-OH, Fmoc-Asn(Trt)-OH, Fmoc-Val-OH, Fmoc-His(Trt)-OH, Fmoc-AEEA-OH *(Fmoc-8-amino-3,6-dioxaoctanoic acid)* (Carbosynth), Fmoc-Cys(Trt)-OH, Fmoc-Lys(N_3_)-OH (IRIS biotech), and **23**.

^***^Generally, the Fmoc amino acid is dissolved in a HCTU solution in DMF (5.00 mL, 0.20 M, 1.0 mmol, 4 eq) The resulting solution was transferred to the reaction vessel followed by a DIPEA solution in DMF (4.00 mL, 0.50 M, 2.0 mmol, 8 eq) to initiate the coupling. The reaction vessel was shaken for 30 min at 50°C (unless stated otherwise).

^****^Aspartic acid and the adjacent Leucine and Arginine were introduced at with 1 h reaction time at room temperature. Fmoc removal was achieved with piperide/DMF in 3 × 5 min at room temperature (Behrendt and Offer, 2016).

For the final conjugation of the gp100 peptide **26** with glycoclusters **19–22**, all solvents were degassed by sonicating while bubbling argon through the solutions. A solution of azidopeptide **26** in DMSO was mixed with a solution of alkyne functionalized glycoclusters in water (**19, 20, 21**, or **22**) followed by addition of an aliquot of a stock solution of CuI (0.1 eq), THPTA (0.3 eq), and DIPEA (0.2 eq) in water ([Cu^+^] = 0.5 M). The reaction was stirred at 45°C and the process was followed via LC-MS. When reactions did not progress and turned blue, a stock solution of sodium ascorbate (0.25 M) and arginine (Conibear et al., 2016) (0.5 M) (0.2–1 eq ascorbate) in water was added. After completion a small amount of Quadrasil® AP (washed with water) was added, stirred for 1 h, filtered and applied on gel filtration (Toyopearl HW40S, 150 mM NH_4_HCO_3_, 1.6 × 60 cm, 1 mL/min) or purified via RP-HPLC followed by lyophilization.

(All compound characterization can be found in the Supplementary Materials section page 30 and further).

### Cell Isolation and Culture

Monocytes were isolated from buffy coats of healthy donors (Sanquin Amsterdam, reference: S03.0023-XT) using sequential Ficoll (STEMCELL Technologies) and Percoll (Sigma) gradient centrifugation, and cultured for 5 days in RPMI 1640 (Invitrogen) with 10% FCS (Biowittaker), 1.000 U/mL penicillin (Lonza), 1 U/mL streptomycin (Lonza), 262.5 U/mL IL-4 (Biosource), and 112.5 U/mL GM-CSF (Biosource). The differentiation of the moDCs was monitored via flow cytometric analysis of DC-SIGN AZN-D1-Alexa488, in house (Geijtenbeek et al., 2002), CD83 and CD86 (both PE-conjugated, Becton Dickinson) expression.

### Surface Plasmon Resonance Analysis

The ECD of DC-SIGN (residues 66–404) was overexpressed and purified as previously described (Tabarani et al., 2009). The DC-SIGN S-ECD construct used for direct interaction experiment (see below) has been overexpressed and purified as described elsewhere (Porkolab et al., 2019). The SPR competition experiments were performed on a BIAcore T200 using a CM3 series S sensor chip. Flow cells were activated as previously described (Halary et al., 2002). Flow cell 1 was functionalized with BSA, blocked with ethanolamine and subsequently used as a control surface. Flow cells 2 and 3 were treated with BSA-Man α1-3[Manα1-6]Man (Dextra) (60 μg/mL) in 10 mM NaOAc pH 4 and blocked with ethanolamine. The final densities on flow cells 2 and 3 were about 2,100 RU. The affinities of the various compounds for DC-SIGN ECD were evaluated via an established inhibition assay (Andreini et al., 2011) in which DCSIGN ECD was injected at 20 μM alone or in the presence of increasing concentration of inhibitors. Injections were performed at 5 μL/min using 25 mM Tris-HCl pH 8, 150 mM NaCl, 4 mM CaCl_2_, 0.05% P20 surfactant as running buffer. The surface was regenerated by the injection of 50 mM EDTA. The data was analyzed in BIAcore BIAevaluation software using four parameter equation.

The direct interaction experiments were executed on a T200 Biacore with a CM3 series S sensor chip. Contrary to the competition assay described above, in this test, DC-SIGN ECD used harbors a StreptagII in its N-terminus (DC-SIGN S-ECD) to allow its capture and functionalization onto the surface in an oriented manner. Flow cells were functionalized as previously described (Porkolab et al., 2019). Briefly, after EDC/NHS activation, flow cells were functionalized with streptactin protein in a first step. Flow cell 1 was used as control, while other flow cells were, in a second round of activation, functionalized with 100 μg/mL of a DC-SIGN S-ECD up to a final density ranging between 2,500 and 3,000 RU, via tag specific capture and linkage by amine coupling chemistry simultaneously. The compounds were injected in running buffer of 25 mM Tris pH 8, 150 mM NaCl, 4 mM CaCl_2_, 0.05% Tween 20 onto the surface at increasing concentrations with a flow rate of 30 μL/min. The ligand titration led to the determination of an apparent *K*_*d*_ value. The data was analyzed in BIAcore BIAevaluation software for direct interaction 1:1 calculation assuming that the *K*_*d*_ will reflect the affinity of the ligands (glycoclusters) for the DC-SIGN oriented surface used as a whole.

### Binding of the Mannose Library to moDCs

Approximately 10^5^ day 5 moDCs were washed and resuspended in 100 μL culture medium (pre-cooled to 4°C). 20 μg/mL AZN-D1 (anti-DC-SIGN, in house Geijtenbeek et al., 2002) or purified mouse anti-human CD206 antibody (Clone 19.2, BD Bioscience) was added to the moDCs and pre-incubated for 45 min on ice. Subsequently, 10 μM of the biotinylated mannoside clusters or 1 μg/mL of Lewis^Y^-conjugated polyacrylamide (positive control) was added, and incubated for 30 min at 4°C. Cells were then washed with pre-cooled PBS (4°C), and stained with Alexa647-labeled streptavidin (Invitrogen^TM^) in PBS supplemented with 0.5% BSA and 0.02% NaN_3_ (PBA) for 30 min at 4°C. Upon washing in ice-cold PBA and fixation in PBS with 0.5% PFA, the fluorescence was measured by flow cytometry (CyAn™ ADP with Summit™ Software), and further analyzed using FlowJo v10.

### Antigen Presentation

Immature day 5 moDCs were seeded in 96-well plates (Greiner) at 50·10^3^ cells/well and incubated with 20 μM of the different gp100-conjugates in the presence or absence of the TLR4 ligand LPS (10 ng/mL) or the TLR7 ligand Imiquimod (2.5 μg/mL). The gp100 short peptide, containing the gp100_280−288_ sequence, was taken along as control, as well as the gp100 long peptide without the four C-terminal linker amino acids (gp100(ctrl)). After 30 min, moDCs were washed, and co-cultured overnight with CD8^+^ HLA-A2.1 restricted T cell clone transduced with the TCR specific for the gp100_280−288_ peptide (10^5^ cells per well, E:T ratio 1:2) (Schaft et al., 2003). IFNγ in the supernatant was measured by sandwich ELISA according to the manufacturer's protocol (Biosource), and measured by spectrophotometric analysis on the iMarkTM Microplate Absorbance Reader (Bio-RAD) at 450 nm.

### Internalization Assay

Immature day 5 moDCs were harvested and washed with cold HBSS (Thermo Fischer), after which half of the moDCs were gently fixed for 20 min at RT with 1% PFA in PBS. Afterwards, 20 μM of the different biotinylated mannose-clusters in cold HBSS were added. The moDCs were incubated for 1 h on ice, and washed in cold HBSS. Subsequently, warm HBSS was added to the cells, and cells were incubated at 37°C in a shaking heating block. At the indicated time points, a sample of the cells was taken and put on ice. After the last time point, the cells were stained with Streptavidin-Alexa647 (Thermo Fisher), measured using flow cytometry (CyAn™ ADP with Summit™ Software), and further analyzed using FlowJo v10. The same procedure was used for internalization with pHrodo^TM^ Red Avidin (Thermo Fischer). The different biotinylated mannose-clusters were however incubated with pHrodo-Avidin (ratio 2:1) for 15 min at 37°C, prior to moDC exposure. The fluorescence upon internalization was measured using flow cytometry (BD LSRFortessa™ X-20 with FACSDiva Software), and further analyzed using FlowJo v10.

### Statistics

Unless otherwise stated, data are presented as the mean ± SD of at least three independent experiments or healthy donors. Statistical analyses were performed in GraphPad Prism v7.04. Statistical significance was set at *P* < 0.05 and was evaluated by the Mann–Whitney *U*-test.

## Data Availability Statement

All datasets generated for this study are included in the manuscript/Supplementary Files.

## Author Contributions

R-JL and TH wrote the first drafts of this manuscript. TH synthesized the described constructs under supervision of HO, DF, GM, and JC. CW synthesized some of the propargyl mannosides. NM and HE assisted in purification and high resolution mass measurements. R-JL determined the cellular affinity, uptake, maturation, and antigen presentation aided by SB and TA under supervision of SV and YK. SPR experiments were performed by SA and CV under supervision of FF. MT was involved in the preparation of DC-SIGN samples.

### Conflict of Interest

The authors declare that the research was conducted in the absence of any commercial or financial relationships that could be construed as a potential conflict of interest.
