# Supplementary material for: Systematic Dual Targeting of Dendritic Cell C-Type Lectin Receptor DC-SIGN and TLR7 Using a Trifunctional Mannosylated Antigen
Source: Front Chem. 2019 Oct 4;7:650. doi: 10.3389/fchem.2019.00650 (PMC6787163; doi:10.3389/fchem.2019.00650)
Supplement: Supplementary file 1 [file Data_Sheet_1.PDF]

Systematic dual targeting of dendritic cell C-type lectin receptor DC-SIGN and TLR7 using a trifunctional mannosylated antigen

Supplementary Material

Supplementary figures and schemes **1**

Experimental - Synthesis **11**

General procedure for automated solid phase synthesis *14*

General copper catalysed click procedure *16*

General biotin introduction procedure *21*

Generated building blocks *23*

General alkyne introduction procedure *24*

General procedure for automated solid phase synthesis *25*

General copper catalysed click procedure *28*

References **29**

Spectral data of Alkynes: *30*

Spectra of azido-backbones: *92*

Spectra of CLR ligands, free amine: *118*

Spectra of CLR Ligands, biotin functionalised *173*

Spectra of generated building blocks *193*

Spectra of CLR Ligands with Alkyne *198*

Spectra of gp100 peptides: *213*

Spectra of glycopeptides *235*

Supplementary figures and schemes

SM Figure 1 | Monocyte-derived dendritic cell DC-SIGN was targeted by the tri-functional conjugates and did not compromise antigen presentation to gp100(Cys)-specific T cells.

(A) Analysis of the SPR assays shows an undetermined (ND; not determined) IC_{50} for β-galactose backbone control **G1**, and low affinity binding of the **G2** cluster, indication minimal interaction with the peptide backbone. Both **gp100** and **gp100-TLR7L** did not show any interaction with DC-SIGN (N.I; no interaction). (B) Flow cytometric analysis of DC-SIGN expression on moDCs. One representative donor out of six is shown. (C) The inhibition in binding of the biotinylated mannoside library by blocking DC-SIGN on moDC was measured with flow cytometry. The binding was reduced, however the cluster-dependent residual binding profiles remain. (D) The internalization of the hexavalent mannoside clusters by fixed moDCs was measured by flow cytometry. One representative donor out of four is depicted. No decay of signal is seen upon binding of all the clusters over time. (E) The biotinylated mannoside clusters were pre-incubated with a pH sensitive avidin-labeled dye (pHrodo). Internalization of the hexavalent clusters was measured by flow cytometry. Depited is the increase in fluorescence upon endosomal routing of the clusters. Clusters **b6**, **c6**, **d6** are internalized rapidly, while **e6** is endocytosed at lower rate. One representative donor out of three is shown. (F) MoDC maturation was assessed by analyzing the CD83 expression, indicating moDC maturation of all compounds harboring a TLR7 ligand. LPS stimulation (10 ng/mL) was used as a positive control. (G) The gp100(Cys) was compared to a gp100 peptide with a methyl analogue α-amino-butyric acid instead of a Cys_{60}. No significant difference was seen between the two peptides in presence of TLR7 ligand. One representative donor out of six is shown. (H) The antigenic peptide used in this paper (gp100) was compared to a long gp100 peptide control without the four C-terminal linker amino acids. An optimum of 20 µM was chosen, as at this concentrations it is possible to measure an increase or decrease in antigen presentation.

A

Conjugate	IC_{50} (μM)	Kd_{app} (μM)
G1	N.D.	
G2	3401	
gp100		N.I.
gp100-TLR7L		N.I.
B6-gp100		10.66
E6-gp100-TLR7L		31.66

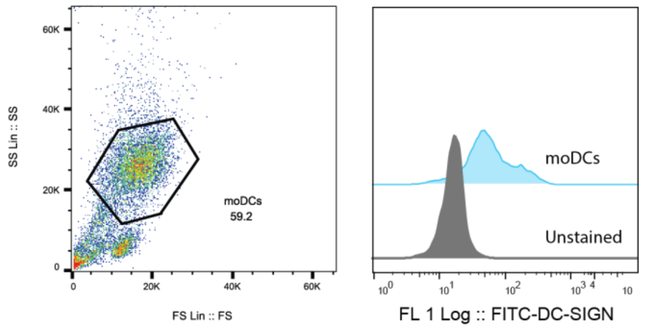

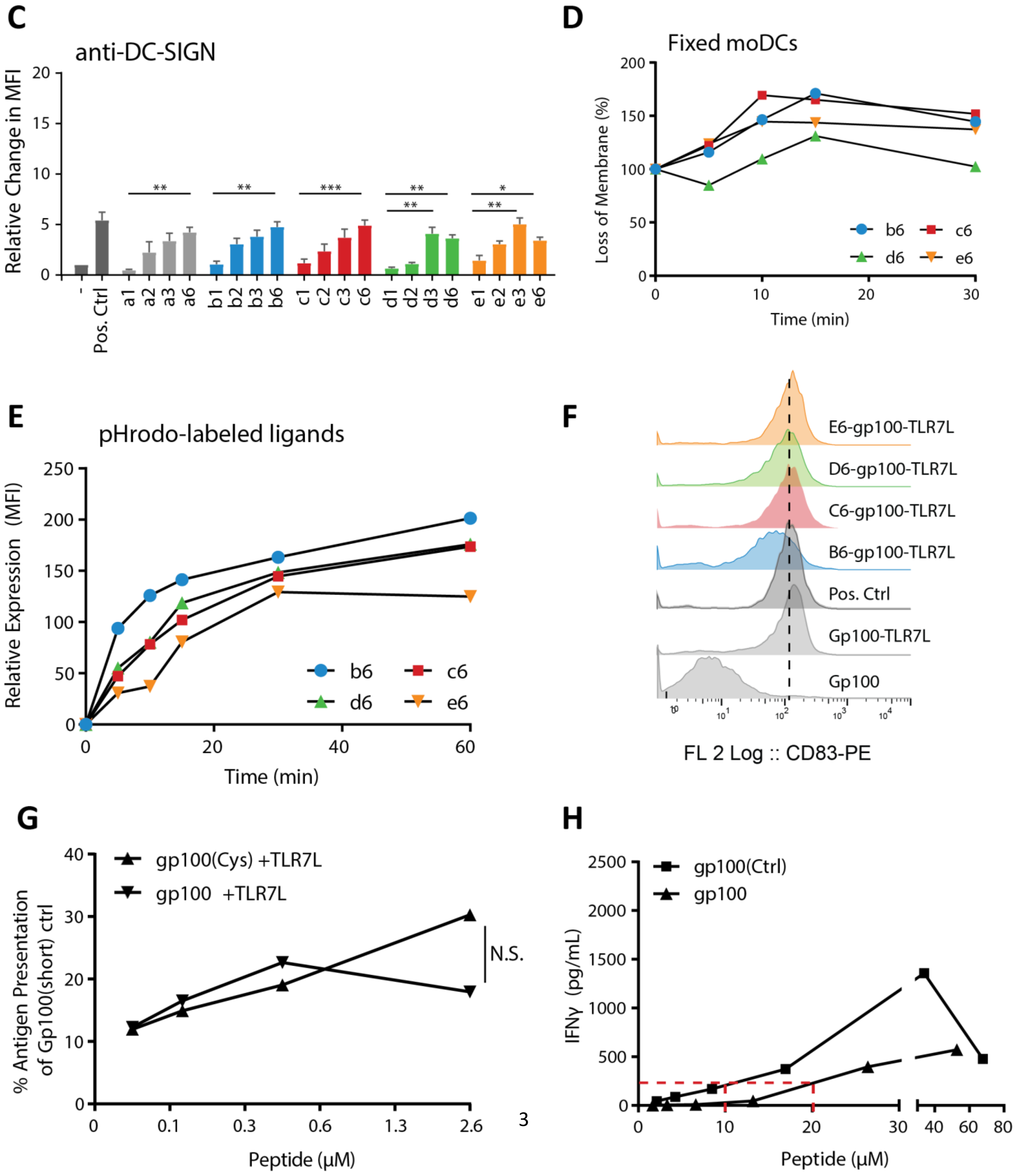

SM Figure 2 | Competition assays (SPR).

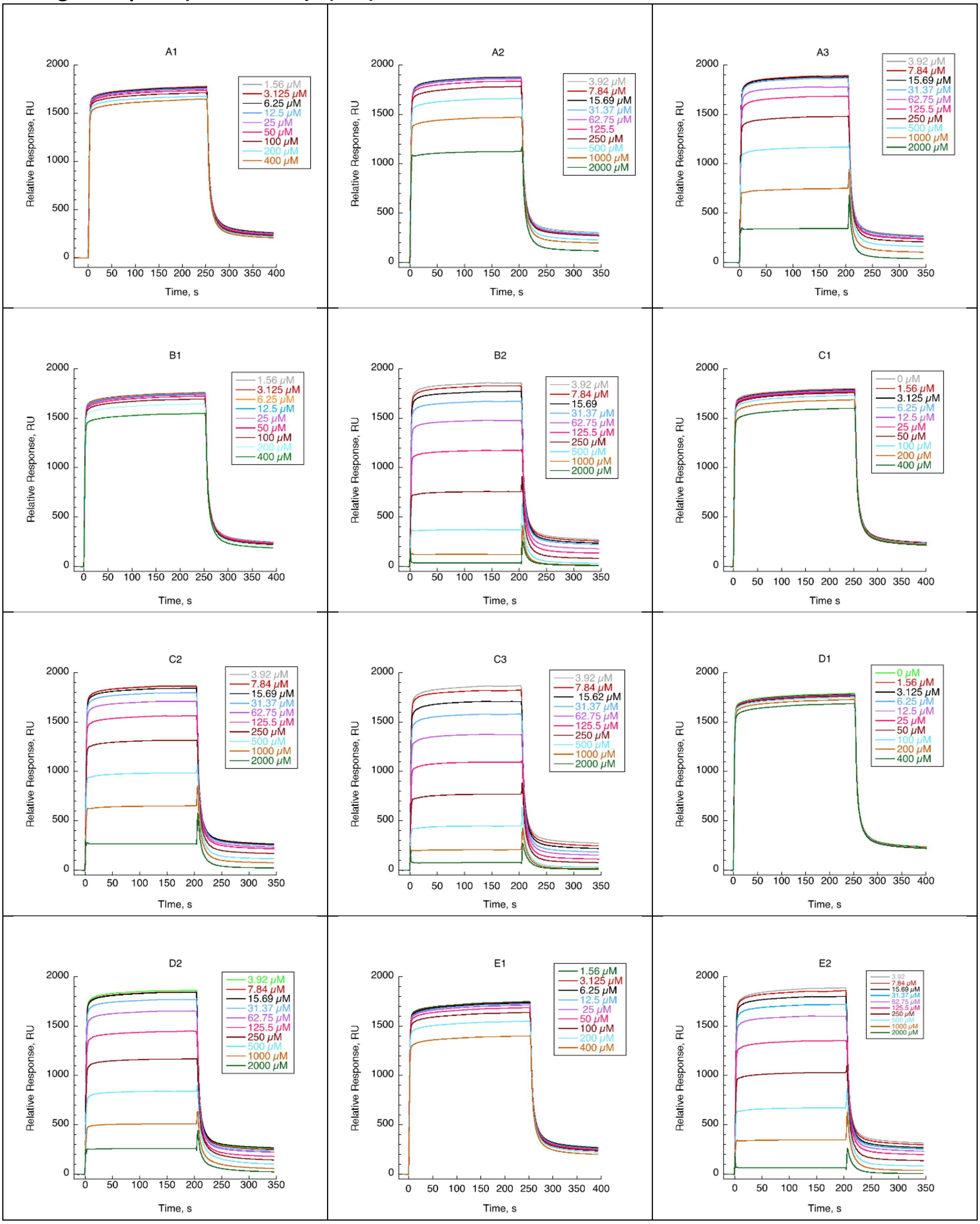

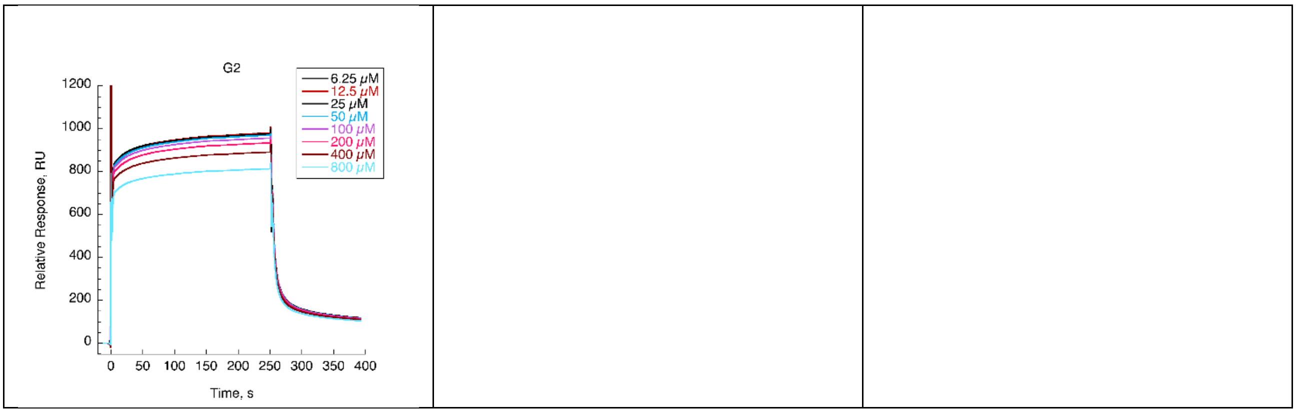

Reference surface corrected sensorgrams showing the inhibition of DC-SIGN interaction with a mannosylated surface

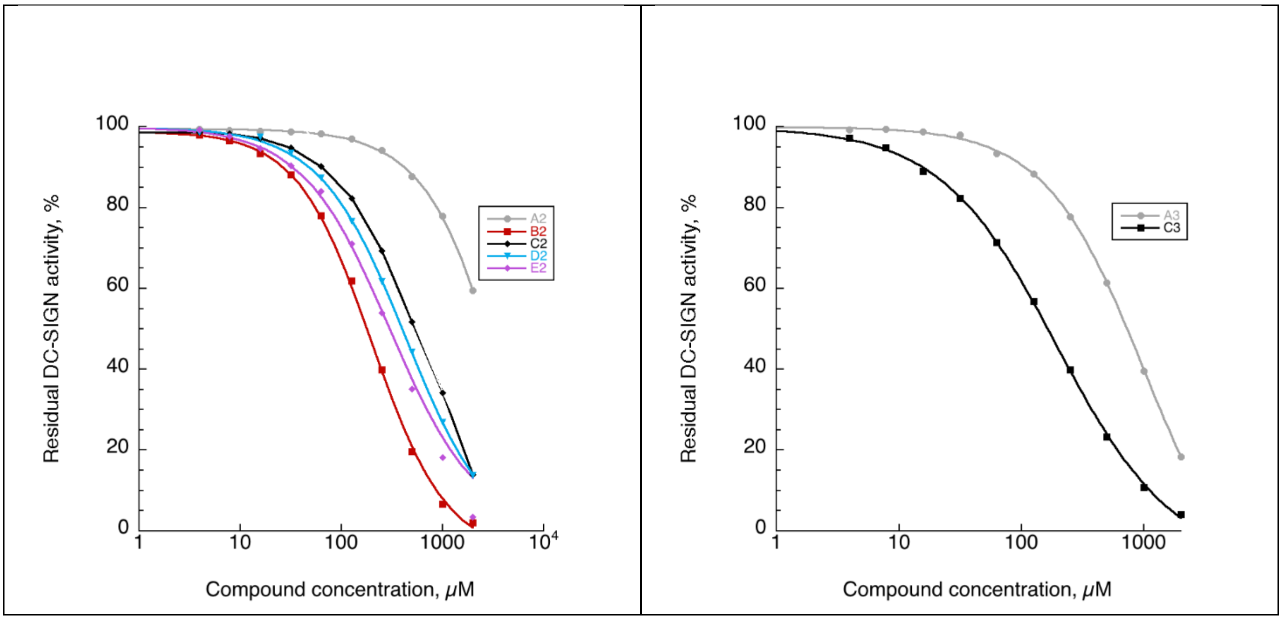

Inhibition curves against DC-SIGN interaction with a mannosylated surface

SM Figure 3 | Direct interaction assays (SPR).

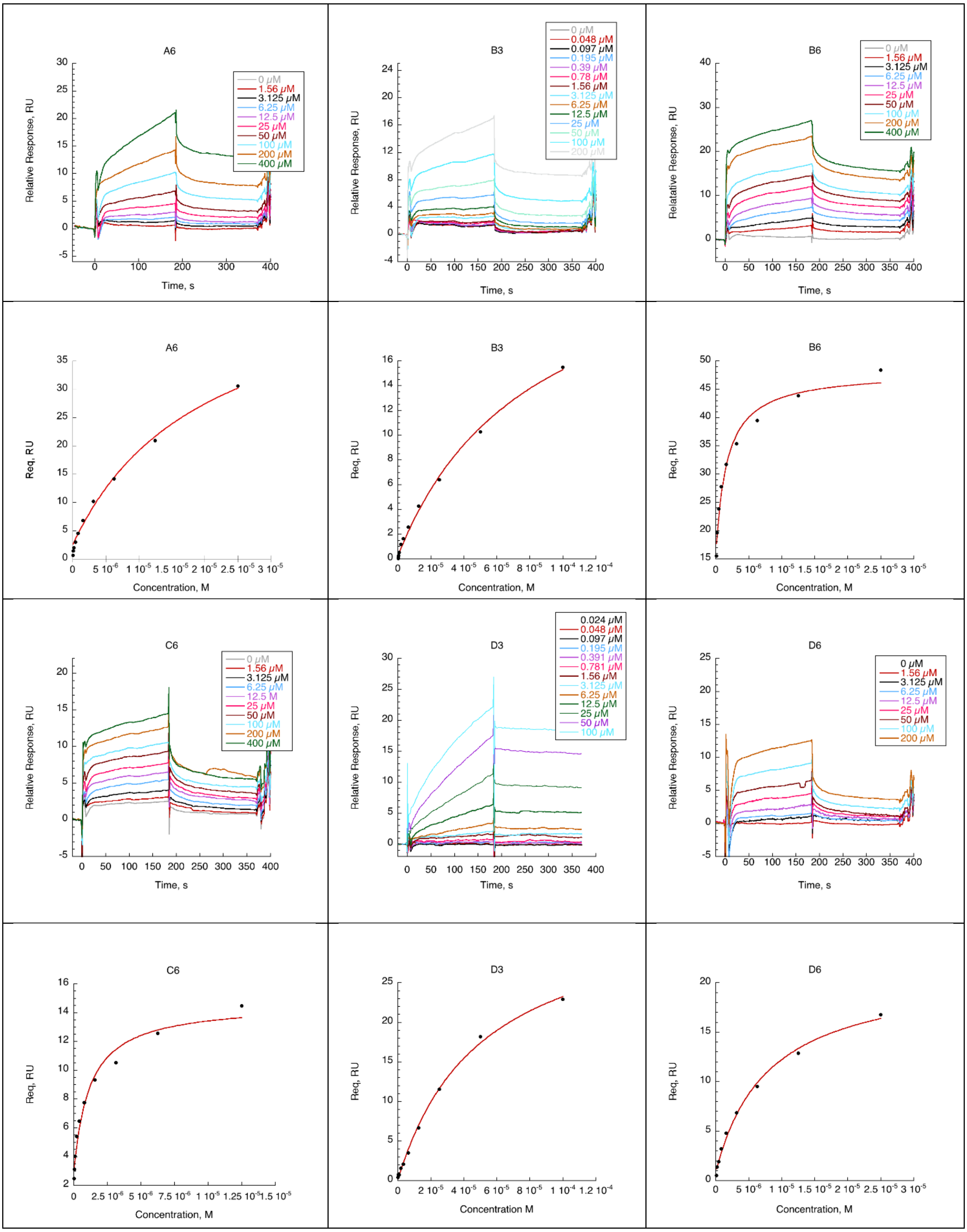

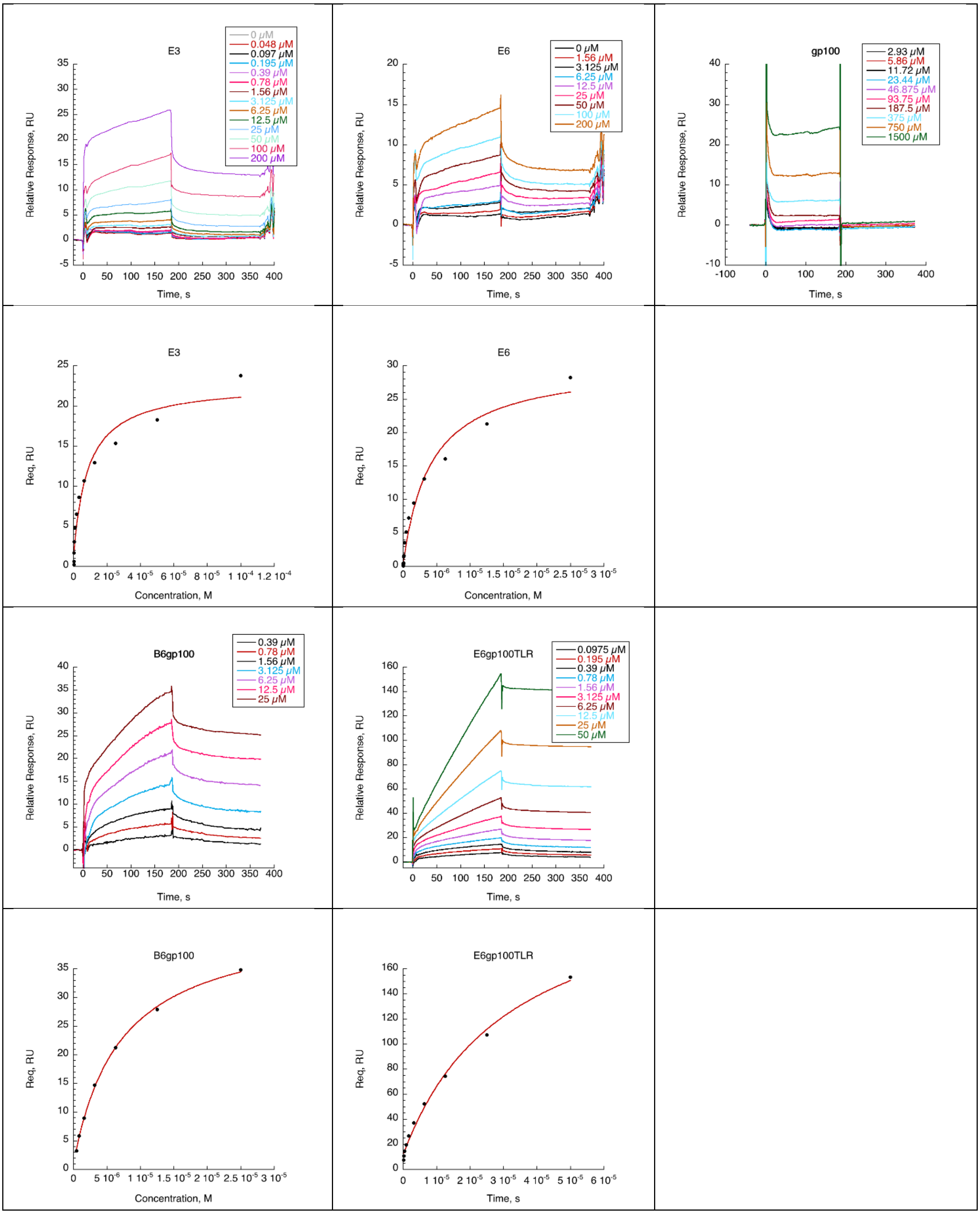

The interaction of mannoside-clusters with oriented DC-SIGN surfaces.

Upper lines: Reference surface corrected sensorgrams of the indicated compound binding to DC-SIGN surfaces.

Bottom lines: Plots of compound binding responses as a function of their concentration by steady state affinity model.

SM Figure 4 | Synthesis of propargyl mannosides.

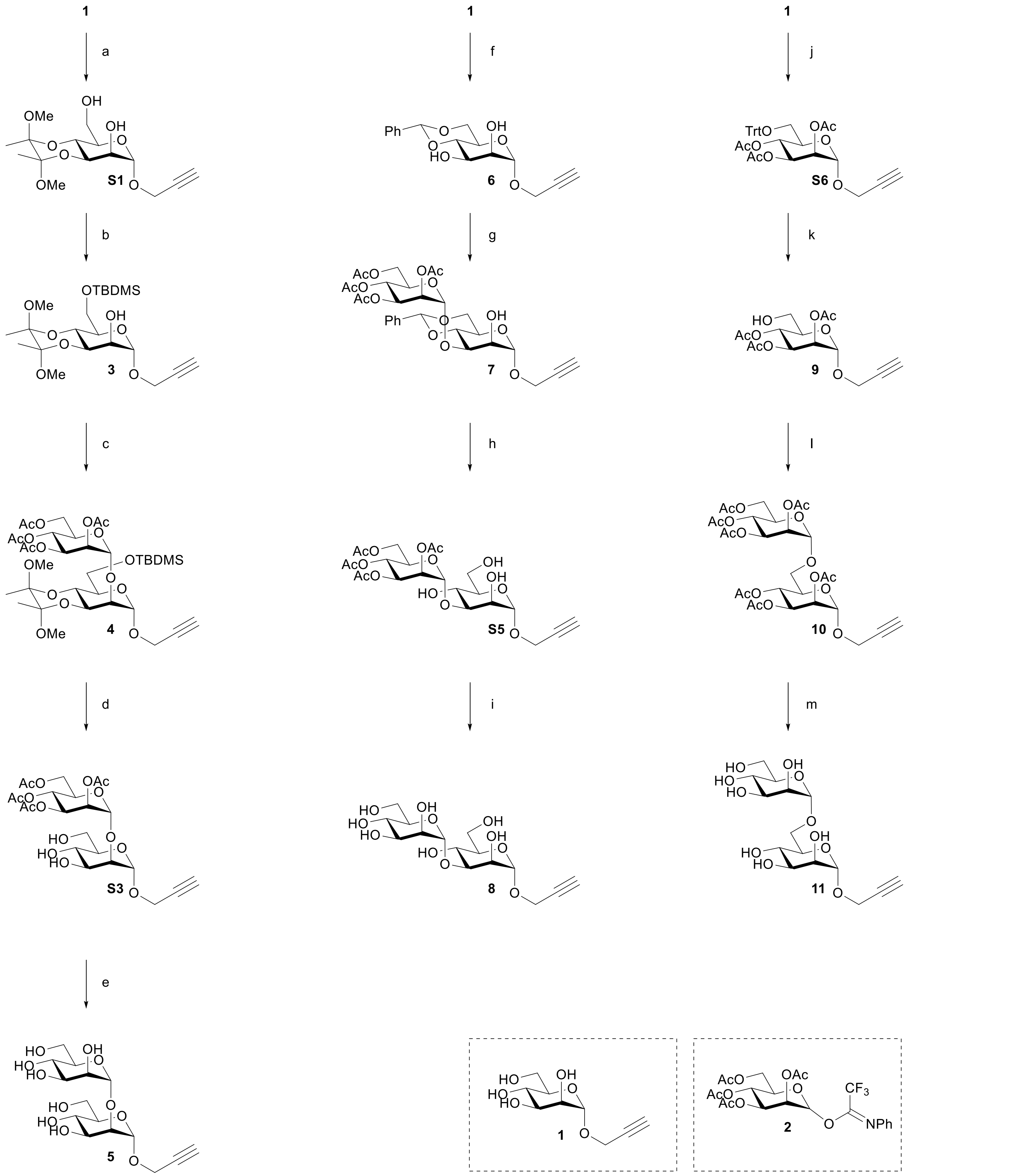

Reagents and conditions: a) 2,3-butanedione, $HC(OMe)_3$, MeOH, CSA, reflux, 55%; b) TBDMSCl, imidazole, DMF, 99%; c) Donor **2**, TMSOTf, DCM,-20°C, 82%; d) TFA, H_2O, 71%; e) NaOMe, MeOH, 82%; f) $PhCH(OMe)_2$, CSA, ACN, 50°C, 300 mbar, 51%; g) Donor **2**, TMSOTf, DCM,-20°C, 80%; h) AcOH, H2O, 95%; i) NaOMe, MeOH, 95%; j) Ph3CCl, imidazole, DCM, followed by Ac_2O, pyridine, 90%; k) $BF_3{\cdot}Et_2O$, MeOH, toluene, 70%; l) Donor **2**, TMSOTf, DCM,-20°C, 79%; m) NaOMe, MeOH, 66%.

SM Figure 5 | Synthesis of β-galactose control clusters.

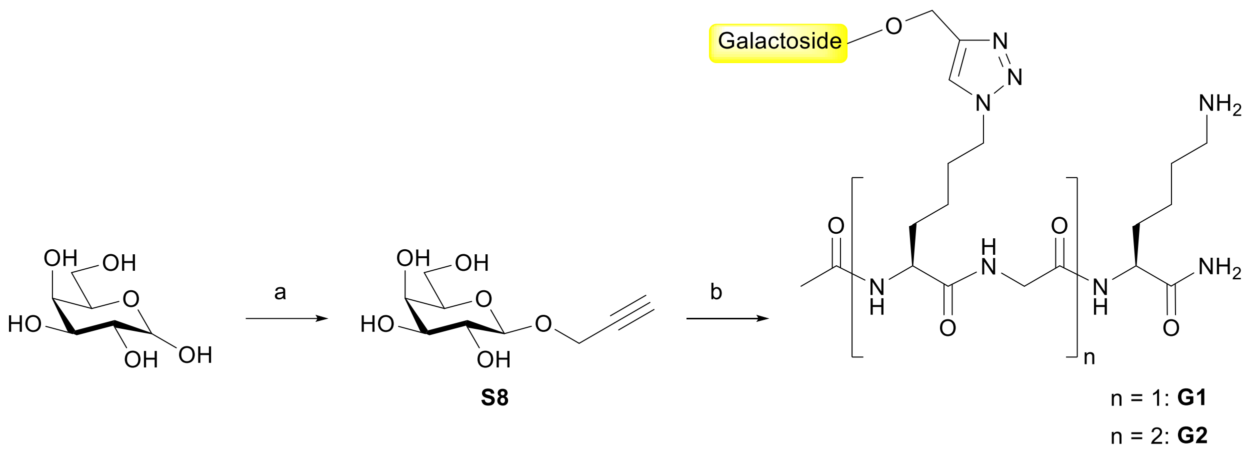

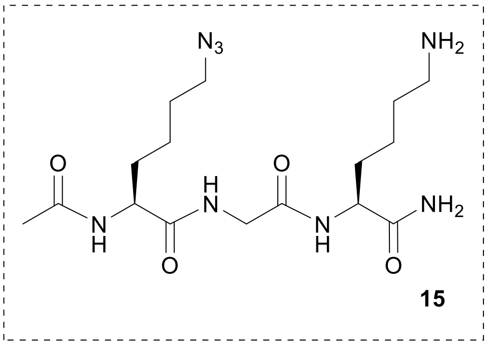

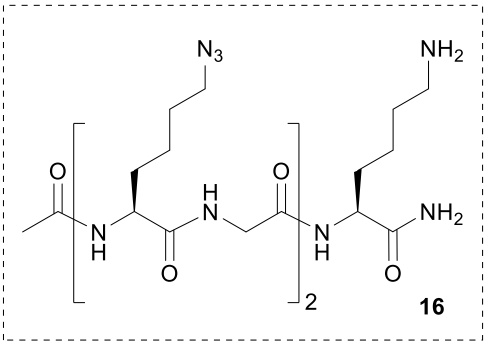

Reagents and conditions: a) see reference(Mereyala and Gurrala, 1998); b) **15** or **16**, CuI, THPTA, DIPEA, DMSO, H_2O.

SM Figure 6 | Synthesis of control gp100 peptides and attempts toward [K(N3)]6-gp100.

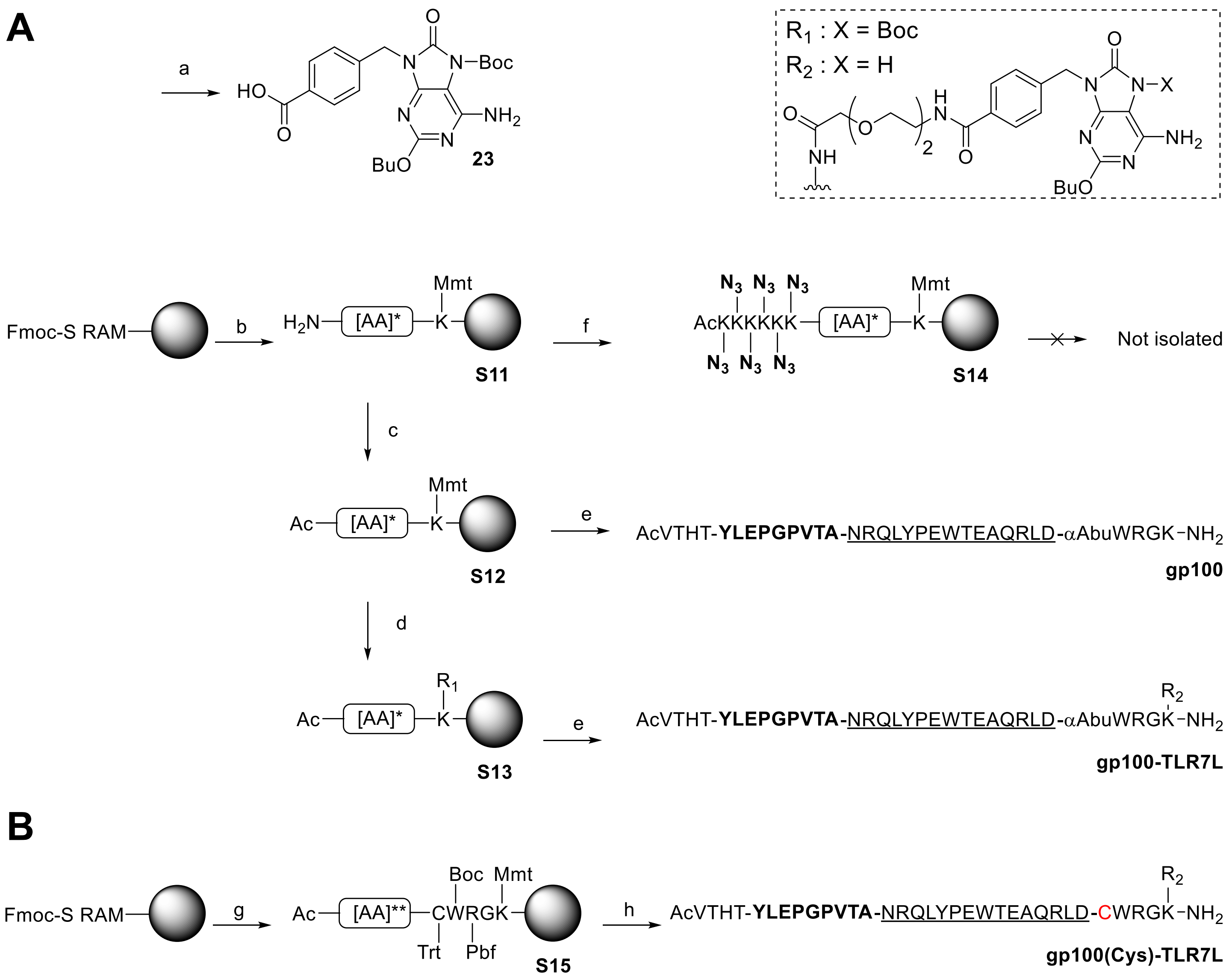

[AA]* = -Val-Thr(tBu)-His(Trt)-Thr(tBu)-Tyr(tBu)-Leu-Glu(OtBu)-Pro-Gly-Pro-Val-Thr(tBu)-Ala-Asn(Trt)-Arg(Pbf)-Gln(Trt)-Leu-Tyr(tBu)-Pro-Glu(OtBu)-Trp(Boc)-Thr(tBu)-Glu(OtBu)-Ala-Gln(Trt)-Arg(Pbf)-Leu-Asp(OtBu)-aAbu-Trp(Boc)-Arg(Pbf)-Gly-

[AA]** = -Val-Thr(tBu)-His(Trt)-Thr(tBu)-Tyr(tBu)-Leu-Glu(OtBu)-Pro-Gly-Pro-Val-Thr(tBu)-Ala-Asn(Trt)-Arg(Pbf)-Gln(Trt)-Leu-Tyr(tBu)-Pro-Glu(OtBu)-Trp(Boc)-Thr(tBu)-Glu(OtBu)-Ala-Gln(Trt)-Arg(Pbf)-Leu-Asp(OtBu)-

Reagents and conditions: a) see reference(Gential et al., 2019); b) standard SPPS; c) Ac_2O, DSIPEA, DMF; d) *i.* TFA, DCM; *ii.* Fmoc-SPPS; *iii.* **23**, HCTU, DIPEA, DMF; e) TFA, TIS, H_2O, octanethiol, phenol; f) Fmoc-SPPS using Fmoc-Lys(N_3)-OH; g) Fmoc-SPPS; h) TFA, TIS, H_2O.

Experimental - Synthesis

Propargyl-α-D-mannopyranoside (1).

Synthesis and spectral data were as described in previous literature(Daly et al., 2012). ^{1}H NMR (400 MHz, MeOD) δ 4.96 (d, *J* = 1.3 Hz, 1H), 4.27 (d, *J* = 2.4 Hz, 2H), 3.88 - 3.76 (m, 2H), 3.75 - 3.58 (m, 3H), 3.51 (ddd, *J* = 8.8, 5.9, 2.2 Hz, 1H), 2.86 (t, *J* = 2.4 Hz, 1H); ;^{13}C NMR (101 MHz, $CDCl_3$) δ 99.8, 80.0, 76.0, 75.1, 72.5, 72.0, 68.4, 62.8, 54.8; HRMS $[C_9H_{14}O_6 + Na]^+$: 241.06827 found, 241.06826 calculated.

2,3,4,6-Tetra-O-acetyl-α/β-D-mannopyranosyl 1-(*N*-phenyl)-2,2,2-trifluoroacetimidate (2).

Synthesis and spectral data were as described in previous literature(Thomas et al., 2007).

Propargyl 3,4-*O*-(2',3'-dimethoxybutan-2',3'-diyl)-α-D-manno-pyranoside (S1).

Propargyl mannose **1** (2.27 g, 10.4 mmol) was dissolved in MeOH (100 mL, 0.1 M) and butan-2,3-dione (1.05 mL, 12.5 mmol, 1.2 eq), trimethyl orthoformate (6.02 mL, 57.1 mmol, 5.5 eq) and 10-Camphorsulfonic acid (232 mg, 1.04 mmol. 0.1 eq) were added successively and the mixture was refluxed overnight. After neutralizing the mixture with Et_3N, the mixture was concentrated *in vacuo* and purified by silica gel column chromatography to yield **S1**. (1/1 → 8/2, Et_2O/PE, v/v).(1.83 g, 5.50 mmol, 55%) ^{1}H NMR (400 MHz, $CDCl_3$) δ 5.06 (s, 1H), 4.24 (d, *J* = 2.4 Hz, 2H), 4.13 (t, *J* = 9.8 Hz, 1H), 4.03 (dd, *J* = 10.3, 3.1 Hz, 1H), 3.96 (s, 1H), 3.87 - 3.74 (m, 3H), 3.28 (s, 3H), 3.27 (s, 3H), 2.46 (t, *J* = 2.4 Hz, 1H), 1.33 (s, 3H), 1.29 (s, 3H);^{13}C NMR (101 MHz, $CDCl_3$) δ 100.5, 100.0, 98.7, 78.8, 75.1, 71.3, 69.6, 68.1, 66.0, 62.9, 61.2, 54.5, 48.3, 48.0, 17.9, 17.8; HRMS $[C_{15}H_{25}O_8 + H]^+$: 333.15444 found, 333.15439 calculated.

Propargyl 3,4-*O*-(2',3'-dimethoxybutan-2',3'-diyl)-6-*O*-tert-butyldimethylsilyl-α-D-manno-pyranoside (3).

Compound **S1** (1.83g, 5.50 mmol) was dissolved in DMF (27.5 mL, 0.2 M) and cooled to 0°C. TBDMSCl (1.04 g, 6.9 mmol, 1.25 eq) and imidazole (1.12 g, 16.5 mmol, 3 eq) were added successively. After stirring overnight at RT, the reaction mixture was quenched with MeOH, diluted in Et_2O and washed with brine (3x). The organic layer was collected, dried over $MgSO_4$ (s), filtered and concentrated *in vacuo*. Purification by silica gel column chromatography (2/8 → 3/6, Et_2O/PE, v/v) yielded acceptor **3** as a colorless oil. (2.42 g, 5.42 mmol, 99%) ^{1}H NMR (400 MHz, $CDCl_3$) δ 4.98 (s, 1H), 4.20 (d, *J* = 2.4 Hz, 2H), 4.03 - 3.94 (m, 2H), 3.91 (s, 1H), 3.82 (dd, *J* = 11.3, 2.0 Hz, 1H), 3.77 (dd, *J* = 11.3, 5.0 Hz, 1H), 3.68 (ddt, *J* = 7.3, 4.8, 2.2 Hz, 1H), 3.25 (s, 3H), 3.22 (s, 3H), 2.48 (s, 1H), 2.40 (t, *J* = 2.4 Hz, 1H), 1.30 (s, 3H), 1.26 (s, 3H), 0.86 (s, 9H), 0.05 (s, 3H), 0.04 (s, 3H); ^{13}C NMR (101 MHz, $CDCl_3$) δ 100.4, 99.9, 98.3, 79.0, 74.7, 72.1, 69.6, 68.4, 62.8, 61.6, 53.8, 48.2, 48.0, 25.9, 18.4, 17.9, 17.8, -5.0, -5.3; HRMS $[C_{21}H_{39}O_8Si + H]^+$: 447.24090 found, 447.24087 calculated.

Propargyl 2-*O*-(2,3,4,6-tetra-*O*-acetyl-α-D-mannopyranosyl)-3,4-*O*-(2',3'-dimethoxybutan-2',3'-diyl)-6-*O*-tert-butyldimethylsilyl-α-D-manno-pyranoside (4).

Donor **2** (390 mg, 0.75 mmol, 1.5 eq) and acceptor **3** (223 mg, 0.5 mmol) were combined and co-evaporated with toluene under N_2 (2x), dissolved in DCM (5 mL, 0.1 M) and stirred with molecular sieves (3Å) for 30 minutes at RT. The mixture was cooled to -20°C and TMSOTf (9 µL, 0.05 mmol, 0.1 eq) was added with stirring at -20°C. The mixture was stirred for three hours, after it was quenched with Et_3N (0.1 mL), allowed to warm up, diluted with DCM and washed with $NaHCO_3$ (sat aq. 1x). The organic layer was dried over MgSO4 (s), filtered, concentrated *in vacuo* and purified via size exclusion (Sephadex LH20, 1/1 MeOH/DCM, v/v) to yield dimer **4** as a clear oil. (320 mg, 0.41 mmol, 82%) ^{1}H NMR (400 MHz, $CDCl_3$) δ .43 (dd, *J* = 3.5, 1.9 Hz, 1H), 5.35 (dd, *J* = 10.0, 3.4 Hz, 1H), 5.24 (t, *J* = 9.9 Hz, 1H), 5.20 (d, *J* = 1.9 Hz, 1H), 4.97 (s, 1H), 4.23 (dd, *J* = 12.1, 5.0 Hz, 1H), 4.20 (d, *J* = 1.5 Hz, 2H), 4.13 - 3.97 (m, 4H), 3.91 (s, 1H), 3.84 (dd, *J* = 11.3, 2.0 Hz, 1H), 3.77 (dd, *J* = 11.3, 5.8 Hz, 1H), 3.71 - 3.61 (m, 1H), 3.22 (s, 3H), 3.20 (s, 3H), 2.41 (t, *J* = 2.4 Hz, 1H), 2.10 (s, 3H), 2.08 (s, 3H), 2.02 (s, 3H), 1.95 (s, 3H), 1.22 (s, 3H), 1.18 (s, 3H), 0.86 (s, 9H), 0.06 (s, 3H), 0.05 (s, 3H); ^{13}C NMR (100 MHz, $CDCl_3$) δ170.7, 169.9, 169.6, 169.6, 100.1, 100.1, 99.6, 99.0, 97.3, 78.7, 75.8, 74.9, 72.8, 69.5, 69.1, 68.9, 68.5, 66.4, 63.1, 62.5, 61.8, 53.7, 48.2, 47.9, 25.9, 20.9, 20.9, 20.8, 20.8, 18.3, 17.7, 17.5, -5.1, -5.3; ^{13}C-GATED (100 MHz, $CDCl_3$) δ 99.6 (d, *J* = 176 Hz, C-1'), 97.4 (d, *J* = 171 Hz, C-1); HRMS $[C_{35}H_{57}O_{17}Si + H]^+$: 777.33598 found, 777.33595 calculated.

Propargyl 2-*O*-(2,3,4,6-tetra-*O*-acetyl-α-D-mannopyranosyl)-α-D-manno-pyranoside (S3).
Dimer **4** was dissolved in TFA/H_2O (12.3 mL, 9/1, v/v) stirred at RT for 30 minutes followed by co-evaporation with toluene (3x). Purification by column chromatography yielded **S3** as a colorless amorphous solid (159 mg, 0.29 mmol, 71%). ^{1}H NMR (400 MHz, $CDCl_3$) δ 5.41 (dd, *J* = 3.3, 1.8 Hz, 1H), 5.31 (dd, *J* = 10.0, 3.3 Hz, 1H), 5.24 (d, *J* = 1.6 Hz, 1H), 5.20 (t, *J* = 10.0 Hz, 1H), 5.05 (d, *J* = 1.8 Hz, 1H), 4.29 (dd, *J* = 6.7, 1.6 Hz, 1H), 4.25 (s, 1H), 4.24 - 4.21 (m, 2H), 4.16 - 4.12 (m, 1H), 4.10 (d, *J* = 6.9 Hz, 1H), 3.99 - 3.87 (m, 3H), 3.83 (dd, *J* = 12.3, 3.0 Hz, 1H), 3.57 (dt, *J* = 9.7, 3.3 Hz, 1H), 3.37 (s, 3H), 2.49 (t, *J* = 2.4 Hz, 1H), 2.14 (s, 6H), 2.08 (s, 3H), 2.00 (s, 3H); ^{13}C NMR (101 MHz, $CDCl_3$) δ 171.6, 170.7, 170.1, 170.0, 99.6, 96.9, 80.3, 78.6, 75.3, 73.1, 70.7, 69.4, 69.2, 68.9, 67.4, 66.2, 63.1, 61.7, 54.5, 21.0, 20.9, 20.8, 20.8; HRMS $[C_{23}H_{33}O_{15} + H]^+$: 549.18138 found, 549.18140 calculated.

Propargyl 2-*O*-(α-D-mannopyranosyl)-α-D-mannopyranoside (5).
To a solution of **S3** (137 mg, 0.25 mmol) in MeOH (2.5 mL, 0.1 M) NaOMe (1.3 mg, 0.025 mmol, 0.1 eq) was added and the mixture was stirred at RT for 4h. The reaction mixture was neutralized with Amberlite H^+ resin, filtered and concentrated. 1,2-Man_2 dimer **5** was precipitated from MeOH in cold vigorously stirred Et_2O. The solids were washed with Et_2O and dried under N_2 flow to yield a white powder (78 mg, 0.21 mmol, 82%). ^{1}H NMR (400 MHz, D_2O) δ 5.14 (d, *J* = 1.6 Hz, 1H), 4.89 (d, *J* = 1.7 Hz, 1H), 4.26 - 4.12 (m, 2H), 3.95 (dd, *J* = 3.3, 1.8 Hz, 1H), 3.84 (dd, *J* = 3.4, 1.8 Hz, 1H), 3.80 - 3.74 (m, 3H), 3.72 (dd, *J* = 9.6, 3.4 Hz, 1H), 3.68 - 3.55 (m, 4H), 3.54 - 3.47 (m, 2H), 2.77 (t, *J* = 2.4 Hz, 1H); ^{13}C NMR (101 MHz, D_2O) δ 102.4, 97.0, 78.7, 76.1, 73.2, 73.0, 70.2, 69.9, 69.9, 69.7, 66.8, 66.7, 61.0, 60.7, 54.4; HRMS $[C_{15}H_{25}O_{11} + H]^+$: 381.13911 found, 381.13914 calculated.

Propargyl 4,6-*O*-benzylidene-α-D-mannopyranoside (6).
Propargyl mannose **1** (2.27 g, 10.4 mmol) was dissolved in ACN (100 mL, 0.1 M) and 10-camphorsulfonic acid (462 mg, 2.08 mmol. 0.2 eq, pH = 1) and benzaldehyde dimethyl acetal (1.86 mL, 12.5 mmol, 1.2 eq) were added successively and the mixture was swirled at 50°C at 300 mbar for three hours after which it was neutralized with Et_3N (0.35 mL), concentrated and the crude product was recrystallized from hot EtOH (± 50 mL) resulting in pure **6** as a white solid. (1.57 g, 5.12 mmol, 51%). ^{1}H NMR (400 MHz, $CDCl_3$) δ 7.54 - 7.45 (m, 2H), 7.43 - 7.33 (m, 3H), 5.55 (s, 1H), 5.04 (d, *J* = 1.3 Hz, 1H), 4.31 - 4.22 (m, 3H), 4.09 - 4.00 (m, 2H), 3.96 - 3.90 (m, 1H), 3.88 - 3.78 (m, 2H), 2.48 (t, *J* = 2.4 Hz, 1H); ^{13}C NMR (101 MHz, $CDCl_3$) δ 137.2, 129.5, 128.5, 126.4, 102.4, 99.0, 78.8, 75.2, 70.9, 68.8, 68.6, 63.6, 54.8; HRMS $[C_{16}H_{19}O_6 + H]^+$: 307.11763 found, 307.11761 calculated.

Propargyl 3-*O*-(2,3,4,6-tetra-*O*-acetyl-α-D-mannopyranosyl)-4,6-*O*-benzylidene-α-D-mannopyranoside (7).
Donor **2** (286 mg, 0.55 mmol, 1.1 eq) and acceptor **6** (153 mg, 0.5 mmol) were combined and co-evaporated with toluene under N_2 (2x), dissolved in DCM (5 mL, 0.1 M) and stirred with molecular sieves (3Å) for 30 minutes at RT. The mixture was cooled to -20°C, TMSOTf (9 µL, 0.05 mmol, 0.1 eq) was added and the stirring was continued at -20°C for two hours. The reaction mixture was quenched with Et_3N (0.1 mL) diluted with DCM and washed with $NaHCO_3$ (sat aq. 1x). The organic layer was dried over $MgSO_4$ (s), filtered, concentrated *in vacuo* and purified via size exclusion (Sephadex LH20, 1/1 MeOH/DCM, v/v) to yield dimer **7** as a foam. (253 mg, 0.40 mmol, 80%). ^{1}H NMR (400 MHz, $CDCl_3$) δ 7.44 (dd, *J* = 6.5, 2.8 Hz, 2H), 7.40 - 7.32 (m, 3H), 5.61 (s, 1H), 5.43 - 5.39 (m, 1H), 5.37 (dd, *J* = 9.8, 3.4 Hz, 1H), 5.29 (s, 1H), 5.24 (t, *J* = 9.9 Hz, 1H), 5.09 (s, 1H), 4.31 - 4.08 (m, 9H), 3.90 - 3.84 (m, 2H), 3.05 (s, 1H), 2.48 (t, *J* = 2.2 Hz, 1H), 2.13 (s, 3H), 2.10 (s, 3H), 2.06 (s, 3H), 2.01 (s, 3H); ^{13}C NMR (101 MHz, $CDCl_3$) δ 170.8, 170.1, 169.7, 169.7, 137.2, 128.9, 128.1, 126.0, 101.4, 98.9, 98.4, 78.4, 78.2, 75.2, 73.6, 70.8, 69.1, 69.1, 69.0, 68.5, 66.4, 64.0, 62.7, 54.5, 20.7, 20.7; ; ^{13}C-GATED (100 MHz, $CDCl_3$) δ 98.9 (d, *J* = 172 Hz, C-1), 98.4 (d, *J* = 176 Hz, C-1'); HRMS $[C_{30}H_{37}O_{15} + H]^+$: 637.21264 found, 637.21270 calculated.

Propargyl 3-*O*-(2,3,4,6-tetra-*O*-acetyl-α-D-mannopyranosyl)-α-D-mannopyranoside (S5).
Dimer **7** (0.113.8 g, 0.179 mmol) was dissolved in an aqueous solution of AcOH (2.7 mL, 70%) and stirred at 55°C for four hours. The solution was diluted with H_2O, neutralized with $NaHCO_3$ (s) and extracted with DCM (5x). The combined organic layers were dried over $MgSO_4$ (s), filtered and concentrated *in vacuo*. Purification by column chromatography (1/1 → 2/8, EtOAc/PE, v/v) yielded compound **S5** as an oil (91.1 mg, 0.170 mmol, 95%).

^{1}H NMR (400 MHz, $CDCl_3$) δ 5.47 - 5.38 (m, 2H), 5.32 - 5.20 (m, 2H), 5.04 (s, 1H), 4.32 - 4.09 (m, 7H), 4.06 (s, 1H), 4.03 - 3.92 (m, 3H), 3.83 (d, *J* = 11.1 Hz, 1H), 3.61 (d, *J* = 9.8 Hz, 1H), 2.44 (t, *J* = 2.8 Hz, 1H), 2.15 (s, 3H), 2.12 (s, 3H), 2.07 (s, 3H), 2.01 (s, 3H); '); ^{13}C NMR (101 MHz, $CDCl_3$) δ 170.9, 170.8, 170.6, 169.7, 99.5, 98.5, 79.3, 78.8, 74.8, 73.1, 70.7, 69.6, 69.4, 68.9, 66.2, 65.1, 62.7, 61.0, 54.4, 20.9, 20.8, 20.8, 20.7; HRMS $[C_{23}H_{33}O_{15} + H]^+$: 549.18142 found, 549.18140 calculated.

Propargyl 3-*O*-(α-D-mannopyranosyl)-α-D-mannopyranoside (8).
To a solution of triol **S5** (91.1 mg, 0.17 mmol) in MeOH (1.7 mL, 0.1 M) NaOMe (1 mg, 17 µmol, 0.1 eq) was added and the mixture was stirred at RT for six hours, then neutralized with Amberlite H^+ resin, filtered and concentrated. The 1,3-Man_2 dimer **8** was precipitated from MeOH in vigorously stirred cold Et_2O. The solids were washed with Et_2O and dried under N_2 flow to yield a white powder (61 mg, 0.16 mmol, 95%). ^{1}H NMR (400 MHz, D_2O) δ 5.00 (d, *J* = 1.7 Hz, 1H), 4.89 (d, *J* = 1.7 Hz, 1H), 4.29 - 4.15 (m, 2H), 3.98 (dd, *J* = 3.3, 1.9 Hz, 1H), 3.95 (dd, *J* = 3.4, 1.7 Hz, 1H), 3.83 - 3.73 (m, 4H), 3.73 - 3.50 (m, 6H), 2.79 (t, *J* = 2.4 Hz, 1H); ^{13}C NMR (101 MHz, D_2O) δ 102.3, 98.6, 78.0, 76.1, 73.3, 73.2, 70.3, 70.0, 69.5, 66.7, 65.9, 60.9, 60.6, 54.5; HRMS $[C_{15}H_{24}O_{11} + Na]^+$: 403.12094 found, 403.12108 calculated.

Propargyl 2,3,4-tri-*O*-acetyl-6-*O*-trityl-α-D-mannopyranoside (S6).
Propargyl mannose **1** (2.27 g, 10.4 mmol) was dissolved in DCM (50 mL, 0.2 M), imidazole (2.12 g, 31.2 mmol. 3 eq) and trityl chloride (3.62 g, 13,0 mmol, 1.25 eq) were added successively and the mixture was stirred overnight. Next, the mixture was cooled to 0°C and Ac_2O (4,4 mL, 46.8 mmol, 4.5 eq) and pyridine (4 mL) were added. The mixture was stirred at RT for four hours, then quenched at 0°C with MeOH, concentrated *in vacuo*, diluted with EtOAc and successively washed with HCl (1x, 1 M, aq.), $NaHCO_3$ (1x, sat. aq.) and brine (2x). The organic layer was dried over $MgSO_4$ (s) filtered and concentrated *in vacuo* to yield crude trityl mannoside **S6** that was used without further purification (5.51 g, 9.40 mmol, 90%). ^{1}H NMR (399 MHz, $CDCl_3$) δ 7.49 - 7.43 (m, 6H), 7.33 - 7.19 (m, 9H), 5.34 - 5.26 (m, 3H), 5.08 (s, 1H), 4.36 - 4.31 (m, 2H), 3.90 (t, *J* = 3.6 Hz, 1H), 3.27 - 3.15 (m, 2H), 2.47 (t, *J* = 2.3 Hz, 1H), 2.17 (s, 3H), 1.96 (s, 3H), 1.73 (s, 3H); ^{13}C NMR (100 MHz, $CDCl_3$) δ 170.1, 170.0, 169.4, 143.8, 128.8, 127.9, 127.1, 95.8, 86.7, 78.3, 77.5, 77.2, 76.8, 75.5, 70.7, 69.7, 69.3, 66.6, 62.4, 54.5, 21.0, 20.8, 20.6; HRMS $[C_{34}H_{35}O_9 + H]^+$: 587.22762 found, 587.22756 calculated.

Propargyl 2,3,4-tri-*O*-acetyl-α-D-mannopyranoside (9).
Mannoside **S6** (4.4 g, 7.5 mmol) was dissolved in a mixture of MeOH/toluene (75 mL, 0.1 M, 1/1, v/v), $BF_3{\cdot}Et_2O$ (1.0 mL, 8.25 mmol, 1.1 eq) was added dropwise and the mixture was stirred at RT for three hours, diluted with EtOAc, washed with H_2O (2x), brine (2x), dried over $MgSO_4$ (s), filtered and concentrated *in vacuo*. Purification by column chromatography (1/1 → 6/4, Et_2O/PE, v/v) yielded acceptor **9** as a white solid (1.81 g, 5.25 mmol, 70%). ^{1}H NMR (399 MHz, $CDCl_3$) δ 5.40 (dd, *J* = 10.2, 3.4 Hz, 1H), 5.33 - 5.22 (m, 2H), 5.05 (d, *J* = 1.4 Hz, 1H), 4.28 (d, *J* = 2.4 Hz, 2H), 3.82 (ddd, *J* = 10.0, 3.9, 2.3 Hz, 1H), 3.77 - 3.68 (m, 1H), 3.68 - 3.58 (m, 1H), 2.47 (t, *J* = 2.4 Hz, 1H), 2.40 (dd, *J* = 8.4, 5.7 Hz, 1H), 2.16 (s, 3H), 2.08 (s, 3H), 2.01 (s, 3H); ^{13}C NMR (100 MHz, $CDCl_3$) δ 171.0, 170.2, 170.0, 96.5, 75.7, 71.3, 69.5, 68.8, 66.5, 61.3, 55.1, 21.0, 20.9, 20.8; HRMS $[C_{15}H_{21}O_9 + H]^+$: 345.11804 found, 345.11801 calculated.

Propargyl 6-*O*-(2,3,4,6-tetra-*O*-acetyl-α-D-mannopyranosyl)-α-D-mannopyranoside (10).
Donor **2** (390 mg, 0.75 mmol, 1.5 eq) and acceptor **9** (172 mg, 0.5 mmol) were combined and co-evaporated with toluene under N_2 (2x), dissolved in DCM (5 mL, 0.1 M) and stirred with molecular sieves (3Å) for 30 minutes at RT. The mixture was cooled to -25°C, TMSOTf (9 µL, 0.05 mmol, 0.1 eq) was added and the mixture was allowed to warm up to 0°C. After two hours the mixture was quenched with Et_3N (0.1 mL) diluted with DCM and washed with $NaHCO_3$ (sat aq. 1x). The aqueous layer was back extracted with DCM (2x), the organic layers were combined, dried over $MgSO_4$ (s), filtered, concentrated *in vacuo* and purified via size exclusion (Sephadex LH20, 1/1 MeOH/DCM, v/v) to yield dimer **10** as a colorless oil. (257 mg, 0.38 mmol, 79%). ^{1}H NMR (400 MHz, $CDCl_3$) δ 5.38 - 5.23 (m, 6H), 5.02 (d, *J* = 1.4 Hz, 1H), 4.86 (d, *J* = 1.4 Hz, 1H), 4.30 (d, *J* = 2.4 Hz, 2H), 4.27 (dd, *J* =

12.2, 5.2 Hz, 1H), 4.17 - 4.05 (m, 2H), 3.99 (ddd, *J* = 9.3, 5.6, 2.3 Hz, 1H), 3.79 (dd, *J* = 11.0, 5.7 Hz, 1H), 3.59 (dd, *J* = 11.0, 2.4 Hz, 1H), 2.52 (t, *J* = 2.4 Hz, 1H), 2.17 (s, 3H), 2.16 (s, 3H), 2.12 (s, 3H), 2.06 (s, 3H), 2.05 (s, 3H), 2.00 (s, 3H), 1.99 (s, 3H); ^{13}C NMR (101 MHz, $CDCl_3$) δ 170.8, 170.2, 170.1, 170.0, 169.9, 169.9, 169.9, 97.6, 96.1, 78.1, 75.7, 69.9, 69.4, 69.4, 69.1, 69.1, 68.7, 66.7, 66.5, 66.0, 62.5, 55.0, 21.0, 20.9, 20.9, 20.8, 20.8, 20.8; ^{13}C-GATED (100 MHz, $CDCl_3$) δ 97.6 (d, *J* = 173 Hz, C-1′), 96.1 (d, *J* = 174 Hz, C-1); HRMS [$C_{29}H_{40}O_{18}$ + H]$^+$: 675.21300 found, 675.21309 calculated.

Propargyl 6-*O*-(α-D-mannopyranosyl)-α-D-mannopyranoside (11).
To a solution of protected dimer **10** (257 mg, 0.38 mmol) in MeOH (3.8 mL, 0.1 M) NaOMe (5 mg, 0.095 mmol, 0.25 eq) was added and the stirring was continued for one hour after which the solution was neutralized with Amberlite H$^+$ resin, filtered and concentrated. The 1,6-Man_2 dimer **11** was precipitated from MeOH in vigorously stirred cold Et_2O. The solids were washed with Et_2O and dried under N_2 flow to yield a white powder (94 mg, 0.25 mmol, 66%). ^{1}H NMR (400 MHz, D_2O) δ 4.90 (d, *J* = 1.6 Hz, 1H), 4.79 (d, *J* = 1.7 Hz, 1H), 4.21 (dd, *J* = 3.4, 2.4 Hz, 2H), 3.89 - 3.51 (m, 12H), 2.80 (t, *J* = 2.4 Hz, 1H); ^{13}C NMR (101 MHz, D_2O) δ 99.4, 99.0, 76.1, 72.7, 71.2, 70.6, 70.5, 69.9, 69.8, 66.6, 66.3, 65.4, 60.8, 54.7; HRMS [$C_{15}H_{24}O_{11}$ + NH_4]$^+$: 398.16561 found, 398.16569 calculated.

Propargyl 3,6-di-*O*-(α-D-mannopyranosyl)-α-D-mannopyranoside (14).
Synthesis and spectral data were as described in previous literature (Wong et al., 2015). ^{1}H NMR (400 MHz, D_2O) δ 4.99 (d, *J* = 1.7 Hz, 1H), 4.87 (d, *J* = 1.7 Hz, 1H), 4.79 (d, *J* = 1.7 Hz, 1H), 4.27 - 4.16 (m, 2H), 3.99 (t, *J* = 2.0 Hz, 1H), 3.95 (dd, *J* = 3.4, 1.7 Hz, 1H), 3.93 - 3.86 (m, 2H), 3.85 - 3.70 (m, 7H), 3.69 - 3.50 (m, 7H), 2.80 (t, *J* = 2.4 Hz, 1H); ^{13}C NMR (101 MHz, D_2O) δ 102.4, 99.3, 99.0, 78.4, 76.1, 73.3, 72.6, 71.4, 70.5, 70.3, 70.0, 69.9, 69.5, 66.6, 65.4, 65.0, 60.9, 54.8, 29.5; HRMS [$C_{21}H_{34}O_{16}$ + Na]$^+$: 565.17399 found, 565.17391 calculated.

Propargyl-β-D-gallactopyranoside (S8).
Synthesis and spectral data were as described in previous literature (Mereyala and Gurrala, 1998). ^{1}H NMR (400 MHz, D_2O) δ 5.14 (s, 1H), 4.35 - 4.23 (m, 2H), 4.09 - 4.03 (m, 2H), 3.96 (td, *J* = 4.2, 2.6 Hz, 2H), 3.80 (dt, *J* = 7.4, 4.4 Hz, 1H), 3.72 - 3.57 (m, 2H), 2.85 (t, *J* = 2.4 Hz, 1H); ^{13}C NMR (101 MHz, D_2O) δ 105.9, 83.4, 81.0, 76.9, 76.9, 70.8, 62.7, 54.5; HRMS [$C_9H_{14}O_6$ + Na]$^+$: 241.06823 found, 241.06826 calculated

General procedure for automated solid phase synthesis of azido-backbones

The solid-phase peptide synthesis was performed on a TRIBUTE® Peptide Synthesizer (Gyros Protein Technologies AB, Arizona, USA) applying Fmoc based protocol starting with Tentagel S-RAM resin (~0.22 mmol/g) on a 100 µmol scale using established synthetic protocols.(Chan and White, 2000) The consecutive steps performed in each cycle were:

1) DMF wash (1x) followed by nitrogen purge; *2)* Deprotection of the Fmoc-group with 20% piperidine in DMF (4 mL)(3 x 5 min); *3)* DMF wash (3x) followed by nitrogen purge; *4)* Coupling of the appropriate amino acid* in five-fold excess (unless stated otherwise)**; *5)* DMF wash (3x) followed by nitrogen purge; *6)* capping with a Ac_2O/DMF/DIPEA solution (4mL, 20/88/2, v/v/v) for 2 min; *7)* DMF wash (2x).

After the complete sequence capping was achieved by utilization of steps 1,2 & 3 followed by 6 and washing with DMF (3x), DCM (3x) and Et_2O (2x) followed by nitrogen purge.

*The Fmoc amino acids applied in this synthesis were: Fmoc-Lys(Boc)-OH, Fmoc-Gly-OH and Fmoc-Lys(N_3)-OH (4eq per coupling, IRIS biotech).

**Generally, the Fmoc amino acid was dissolved in a HCTU solution in DMF (2.50 mL ,0.20 M, 0.5 mmol, 5 eq) This solution was transferred to the reaction vessel followed by a DIPEA solution in DMF (2.00 mL, 0.50 M, 1.0 mmol, 10 eq) to initiate the coupling. Next, the reaction vessel was shaken for 60 min at room temperature

Ac-Lys(N_3)-Gly-Lys-NH_2 (15).

Ac-Lys(N_3)-Gly-Lys(Boc)-Tentagel-S-RAM was transferred to a flask and treated for 90 minutes with a cleavage cocktail (10 mL, TFA/TIS/H_2O, 190/5/5, v/v/v). The resin was filtered off and washed with neat TFA (3 x 1 mL). The filtrate was concentrated, co-evaporated with toluene (2x). Purification via RP-HPLC yielded title compound **15** (5-30% B, 10 min) as an clear oil after lyophilization. (32.2 mg, 80.9 µmol, 81%). LC-MS: R_t = 2.79 min (0 - 90% ACN; 13 min); ^{1}H NMR (500 MHz, DMSO) δ 8.27 (t, *J* = 5.8 Hz, 1H, NH (G)), 8.14 (d, *J* = 7.2 Hz, 1H, NH (K)), 7.77 (d, *J* = 8.2 Hz, 1H, NH (K)), 7.68 (s, 2H, CH_2N*H_2*), 7.29 (s, 1H, (CO)N*H*H), 7.08 (s, 1H, (CO)NH*H*), 4.18 - 4.10 (m, 2H, CH), 3.75 (dd, *J* = 16.6, 6.0 Hz, 1H, C*H*H (G)), 3.66 (dd, *J* = 16.6, 5.6 Hz, 1H, CH*H* (G)), 3.31 (td, *J* = 6.8, 1.2 Hz, 2H, CH_2-N_3), 2.74 (p, *J* = 6.5 Hz, 2H, C*H_2*-NH_2), 1.85 (s, 3H, Ac), 1.75 - 1.61 (m, 2H, CH_2), 1.51 (td, *J* = 12.1, 10.9, 4.7 Hz, 6H, CH_2), 1.42 - 1.21 (m, 4H, CH_2); ^{13}C NMR (126 MHz, DMSO) δ 173.5, 172.4, 169.8, 168.7 (C=O), 52.9, 52.1 (CH), 50.5 (CH_2-N_3), 42.2 (CH_2 (G)), 38.7 (CH_2-NH_2), 31.3, 31.0, 27.9, 26.6, 22.6 (CH_2), 22.5 (Ac), 22.2 (CH_2); HRMS [$C_{16}H_{30}N_8O_4$ + H]$^+$: 399.2475 found , 399.2463 calculated.

Ac-Lys(N_3)-Gly-Lys(N_3)-Gly-Lys-NH_2 (16).

Ac-Lys(N_3)-Gly-Lys(N_3)-Gly-Lys(Boc)-Tentagel-S-RAM was transferred to a flask and treated for 90 minutes with a cleavage cocktail (10 mL, TFA/TIS/H_2O, 190/5/5, v/v/v). The mixture was concentrated to approximately one mL after which the resin was filtered off into a cold mixture of diethylether/pentane (45 mL, 5/4, v/v) and the resin was washed off with neat TFA (3 x 1 mL) into the ether solution. This solution was centrifuged (10 minutes, 5000 rpm) after which the supernatant was removed and the precipitate was dried under nitrogen flow. Purification via RP-HPLC yielded title compound **16** (10-40% B, 10 min) as a white powder after lyophilization. (30.47 mg, 49.9 µmol, 49.9%). LC-MS: R_t = 4.21 min (0 - 90% ACN; 13 min); ^{1}H NMR (500 MHz, DMSO) δ 8.25 (dt, *J* = 17.2, 5.5 Hz, 2H, NH (G)), 8.13 (d, *J* = 7.1 Hz, 1H, NH (K)), 7.90 (d, *J* = 7.6 Hz, 1H, NH (K)), 7.82 - 7.69 (m, 3H, NH (K), CH_2N*H_2*), 7.32 (s, 1H, (CO)N*H*H), 7.05 (s, 1H, (CO)NH*H*), 4.28 - 4.06 (m, 3H, CH), 3.79 - 3.64 (m, 4H, CH_2 (G)), 3.29 (q, *J* = 6.8 Hz, 4H, CH_2-N_3), 2.75 (q, *J* = 6.3 Hz, 2H, C*H_2*NH_2), 1.85 (s, 3H, Ac), 1.75 - 1.44 (m, 12H, CH_2), 1.42 - 1.20 (m, 6H, CH_2); ^{13}C NMR (126 MHz, DMSO) δ 173.5, 172.3, 172.0, 169.8, 169.1, 168.6 (C=O), 52.9, 52.6, 52.1 (CH), 50.5 (CH_2-N_3), 42.2, 42.1 (CH_2 (G)), 38.7 (CH_2-NH_2), 31.3, 31.2, 31.2, 31.1, 27.9, 27.9, 26.6, 22.6, 22.5 (CH_2), 22.5 (Ac), 22.3 (CH_2); HRMS [$C_{24}H_{43}N_{13}O_6$ + H]$^+$: 610.3539 found , 610.3532 calculated.

Ac-Lys(N_3)-Gly-Lys(N_3)-Gly-Lys(N_3)-Gly-Lys-NH_2 (17).

Ac-Lys(N_3)-Gly-Lys(N_3)-Gly-Lys(N_3)-Gly-Lys(Boc)-Tentagel-S-RAM was transferred to a flask and treated for 90 minutes with a cleavage cocktail (10 mL, TFA/TIS/H_2O, 190/5/5, v/v/v). The resin was filtered off into a cold mixture of diethylether/pentane (45 mL, 5/4, v/v) and the resin was washed off with neat TFA (3 x 1 mL) into the ether solution. This solution was centrifuged (10 minutes, 5000 rpm) after which the supernatant was removed and the precipitate was dried under nitrogen flow. Purification via RP-HPLC yielded title compound **17** (16-38% B, 10 min) as a white powder after lyophilization (36.01 mg, 43.9 µmol, 43.9% (89% per step)). LC-MS: R_t = 4.80 min (0 - 90% ACN; 13 min); ^{1}H NMR (400 MHz, DMSO) δ 8.33 - 8.20 (m, 3H, NH (G)), 8.14 (d, *J* = 7.3 Hz, 1H, NH (K)), 7.97 (d, *J* = 7.7 Hz, 1H, NH (K)), 7.90 (d, *J* = 7.8 Hz, 1H, NH (K)), 7.85 (d, *J* = 8.2 Hz, 1H, NH (K)), 7.70 (s, 2H, CH_2N*H_2*), 7.36 (s, 1H, (CO)N*H*H), 7.09 (s, 1H, (CO)NH*H*), 4.33 - 4.09 (m, 4H, CH), 3.87 - 3.63 (m, 6H, CH_2 (G)), 3.31 (tt, *J* = 6.9, 2.8 Hz, 6H, CH_2N_3), 2.76 (t, *J* = 7.2 Hz, 2H, C*H_2*NH_2), 1.86 (s, 3H, Ac), 1.76 - 1.20 (m, 24H, CH_2); ^{13}C NMR (101 MHz, DMSO) δ 173.9, 172.7, 172.4, 172.3, 170.2, 169.4, 169.3, 169.0 (C=O), 53.2, 52.9, 52.5 (CH), 51.0 (CH_2N_3), 42.5 (CH_2 (G)), 39.2 (CH_2NH_2), 31.7, 28.4, 28.3, 27.1, 23.1 (CH_2), 22.9 (Ac), 22.9, 22.7 (CH_2); HRMS [$C_{32}H_{56}N_{18}O_8$ + H]$^+$: 821.4608 found, 821.4601 calculated.

Ac-Lys(N_3)-Lys(N_3)-Lys(N_3)-Lys(N_3)-Lys(N_3)-Lys(N_3)-Gly-Lys-NH_2 (18).

Ac-Lys(N_3)-Lys(N_3)-Lys(N_3)-Lys(N_3)-Lys(N_3)-Lys(N_3)-Gly-Lys(Boc)-Tentagel-S-RAM was transferred to a flask and treated for 90 minutes with a cleavage cocktail (10 mL, TFA/TIS/H_2O, 190/5/5, v/v/v). The mixture was concentrated to approximately one mL after which the resin was filtered off into a cold mixture of diethylether/pentane (45 mL, 5/4, v/v) and the resin was washed off with neat TFA (3 x 1 mL) into the ether solution. This solution was centrifuged (10 minutes, 5000 rpm) after which the supernatant was removed and the precipitate was dried under nitrogen flow and dissolved (1mL, *t*BuOH/ACN/H_2O, 1/1/1, v/v/v). Purification via RP-HPLC yielded title compound **18** (30-60% B, 10 min) as a white powder after lyophilization (51.57 mg, 44.1 µmol, 44.1% (90% per step)). LC-MS: R_t = 6.70 min (0 - 90% ACN; 13 min); ^{1}H NMR (400 MHz, DMSO) δ 8.14 (t, *J* = 5.6 Hz, 1H, NH (G)), 8.09 - 8.01 (m, 2H, NH (K)), 7.92 (dt, *J* = 18.6, 6.4 Hz, 5H, NH (K)), 7.70 (s, 2H, $CH_2N\mathit{H_2}$), 7.36 (s, 1H, (CO)N*H*H), 7.09 (s, 1H, (CO)NH*H*), 4.32 - 4.10 (m, 7H, CH), 3.78 - 3.72 (m, 2H, CH_2 (G)), 3.34 - 3.26 (m, 12H, CH_2N_3), 2.76 (s, 2H, $C\mathit{H_2}NH_2$), 1.86 (s, 3H, Ac), 1.77 - 1.18 (m, 42H, CH_2); ^{13}C NMR (101 MHz, DMSO) δ 173.4, 171.9, 171.8, 171.5, 171.4, 169.5, 168.5 (C=O), 52.3, 52.0 (CH), 50.5 (CH_2N_3), 42.5 (CH_2 (G)), 38.7 (CH_2NH_2), 31.4, 27.9, 26.7, 22.6 (CH_2), 22.5 (Ac), 22.4 (CH_2); HRMS [$C_{46}H_{80}N_{28}O_9$ + H]$^+$: 1169.6738 found, 1169.6736 calculated.

General procedure for propargyl azide conjugation.

All solvents used in these reactions were degassed by sonicating while bubbling argon through the solutions. The "general click protocol" used was the following procedure: A solution of azidopeptides in DMSO (0.5 M, 1eq) was mixed with a solution of propargyl glycoside in water (0.5 M, 1.2 eq per azide) followed by addition of an aliquot of a stock solution of CuI (0.1 eq), THPTA (0.3 eq) and DIPEA (0.2 eq) in water ([Cu^+] = 0.5 M). The reaction was stirred at 40°C and the process was followed via LC-MS. When reactions do not progress and turn blue, a sodium ascorbate solution (0.2 - 1 eq, 1 M, aq) was added. Generally reactions were stirred overnight at 40°C. When not complete after 16 h an extra aliquot of the copper stock was added. After completion a small amount of Quadrasil® AP (washed with water) was added, stirred for 1 h, filtered and applied on gel filtration (Toyopearl HW40S, 150 mM NH_4HCO_3 aq. (sometimes a percentage of ACN was added to the elution buffer), 1.6x60 cm, 1 mL/min) followed by lyophilization.

Ac-Lys(Man_1)-Gly-Lys-NH_2 (A1).

Azide **15** (5.37 mg, 13.48 µmol) was conjugated to propargyl mannoside **1** using the general click protocol. Compound **A1** was obtained after purification by gel filtration (eluting buffer contains 10% ACN, elutes at 49.5 - 57 mL) followed by RP-HPLC (8 - 16% B, 12 min) as a white powder (3.30 mg, 5.34 µmol, 39%). LC-MS: R_t = 4.01 min (0 - 50% ACN; 13 min); ^{1}H NMR (400 MHz, D_2O) δ 8.03 (s, 1H, trzl), 4.94 (d, *J*=1.6, 1H, H-1), 4.68 (d, *J*=12.5, 1H Hz, CH*H*), 4.42 (t, *J*=6.6 Hz, 2H), 4.27 (dd, *J*=9.4, 4.9 Hz, 1H), 4.18 (dd, *J*=8.7 Hz, 5.9, 1H), 3.97 - 3.52 (m, 8H), 2.94 (t, *J*=7.6 Hz, 2H), 1.98 (s, 3H, Ac), 1.96 - 1.56 (m, 8H), 1.51 - 1.18 (m, 4H); HRMS [$C_{25}H_{44}N_8O_{10}$ + H]$^+$: 617.3254 found, 617.3253 calculated.

Ac-Lys(Man_1)-Gly-Lys(Man_1)-Gly-Lys-NH_2 (A2).

Azide **16** (17.00 mg, 27.89 µmol) was conjugated to propargyl mannoside **1** using the general click protocol. Compound **A2** was obtained after purification by gel filtration (elutes at 42-52 mL) as a white powder (17.66 mg, 16.88 µmol, 61%). LC-MS: R_t = 4.37 min (0 - 50% ACN; 13 min); ^{1}H NMR (500 MHz, D_2O) δ 8.07 (s, 2H, trzl), 4.98 (s, 2H, H-1), 4.84 (dd, *J* = 12.5, 2.1 Hz, 2H, O-C*H*H-trzl), 4.73 (d, *J* = 12.4 Hz, 2H, O-CH*H*-trzl), 4.51 - 4.42 (m, 4H, CH_2-trzl), 4.36 (d, *J* = 2.6 Hz, 1H), 4.34 - 4.19 (m, 3H, CH), 4.14 - 4.10 (m, 1H), 4.05 (d, *J* = 2.6 Hz, 2H), 4.03 -

3.57 (m, 16H, H-2, H-3, H-4, H-5, H-6, CH_2 (G)), 3.01 (t, *J* = 6.6 Hz, 2H, C*H₂*-NH_2), 2.01 (s, 3H, Ac), 1.98 - 1.23 (m, 18H, CH_2); <u>HRMS</u> [$C_{42}H_{71}N_{13}O_{18}$ + H]$^+$: 1046.5118 found, 1046.5113 calculated.

Ac-Lys(Man_1)-Gly-Lys(Man_1)-Gly-Lys(Man_1)-Gly-Lys-NH_2 (A3).

Azide **17** (2.92 mg, 3.56 µmol) was conjugated to propargyl mannosides **1** using the general click protocol. Compound **A3** was obtained after purification by gel filtration (elutes at 38-46 mL) followed by RP-HPLC as a white powder (3.61 mg, 2.44 µmol, 69%). <u>LC-MS</u>: R_t = 4.60 min (0 - 50% ACN; 13 min); <u>^{1}H NMR</u> (400 MHz, D_2O) δ 7.96 - 7.89 (m, 3H, trzl), 4.84 (s, 3H, H-1), 4.71 - 4.67 (m, 3H, O-C*H*H-trzl), 4.58 (d, *J* = 12.5 Hz, 3H, O-CH*H*-trzl), 4.32 (t, *J* = 6.8 Hz, 6H, CH_2-trzl), 4.22 - 4.04 (m, 4H, CH), 3.90 - 3.43 (m, 24H, CH_2 (G), H-2, H-3, H-4, H-5, H-6), 2.87 (t, *J* = 7.5 Hz, 2H, CH_2NH_2), 1.87 (s, 3H, Ac), 1.84 - 1.07 (m, 24H, CH_2); <u>^{13}C NMR</u> (101 MHz, D_2O) δ 143.4, 99.4 (C-1), 72.9, 70.4, 69.9, 66.6 (C-2, C-3, C-4, C-5), 60.8 (C-6), 28.7; <u>HRMS</u> [$C_{59}H_{98}N_{18}O_{26}$ + 2H]$^{2+}$: 738.3528 found, 738.3523 calculated.

Ac-Lys(Man_1)-Lys(Man_1)-Lys(Man_1)-Lys(Man_1)-Lys(Man_1)-Lys(Man_1)-Gly-Lys-NH_2 (A6).

Azide **18** (2.51 mg, 2.15 µmol) was conjugated to propargyl mannosides **1** using the general click protocol. Compound **A6** was obtained after purification by gel filtration (eluting buffer contains 20% ACN, elutes at 39-47 mL) as a white powder (4.31 mg, 1.76 µmol, 82%). <u>LC-MS</u>: R_t = 4.73 min (0 - 50% ACN; 13 min); <u>^{1}H NMR</u> (500 MHz, D_2O) δ 8.03 (d, *J* = 5.1 Hz, 6H, trzl), 4.96 (d, *J* = 1.8 Hz, 6H, H-1), 4.89 - 4.71 (m, 146H, HDO, O-C*H*H-trzl), 4.67 (dd, *J* = 12.5, 2.9 Hz, 6H, O-CH*H*-trzl), 4.40 (t, *J* = 6.4 Hz, 12H, CH_2-trzl), 4.34 - 4.09 (m, 7H, CH (K)), 3.99 - 3.57 (m, 38H, H-2, H-3, H-4, H-5, H-6, CH_2 (G)), 3.05 - 2.98 (m, 2H, C*H₂*-NH_2), 2.01 (s, 3H, Ac), 1.97 - 1.20 (m, 42H, CH_2); <u>^{13}C NMR</u> (126 MHz, D_2O) δ 160.3, 143.5, 125.0 (CH-trzl), 99.5 (C-1), 73.0, 70.6, 70.0, 66.7 (C-2, C-3, C-4, C-5), 60.9 (C-6), 59.8 (O-*C*H_2-trzl), 50.1 (*C*H_2-trzl), 39.3 (CH_2-NH_2), 28.9, 22.1 (CH_2); <u>HRMS</u> [$C_{100}H_{164}N_{28}O_{45}$ + 2H]$^{2+}$: 1240.0857 found, 1240.0790 calculated.

Ac-Lys(1,2-Man_2)-Gly-Lys-NH_2 (B1).

Azide **15** (11.03 mg, 27.67 µmol) was conjugated to propargyl mannosides **5** using the general click protocol. Compound **B1** was obtained after purification by gel filtration (elutes at 50-62 mL) followed by RP-HPLC (8 - 16% B, 12 min) as a white powder (14.71 mg, 18.88 µmol, 68%). <u>LC-MS</u>: R_t = 3.87 min (0 - 50% ACN; 13 min); <u>^{1}H NMR</u> (500 MHz, D_2O) δ 8.07 (s, 1H, trzl), 5.19 (d, *J* = 1.6 Hz, 1H, H-1), 5.01 (d, *J* = 1.8 Hz, 1H, H-1′), 4.84 (d, *J* = 12.6 Hz, 1H, O-C*H*H-trzl), 4.73 (d, *J* = 12.6 Hz, 1H, O-CH*H*-trzl), 4.47 (t, *J* = 6.9 Hz, 2H, CH_2-trzl), 4.32 (dd, *J* = 9.5, 5.0 Hz, 1H, CH), 4.23 (dd, *J* = 8.8, 5.7 Hz, 1H, CH), 4.08 (dd, *J* = 3.3, 1.8 Hz, 1H, H-2), 3.99 - 3.58 (m, 14H, CH, H-2′, H-3, H-3′, H-4, H-4′, H-5, H-5′, H-6, H-6′, CH_2 (G)), 3.01 (td, *J* = 8.0, 2.1 Hz, 2H, C*H₂*-NH_2), 2.03 (s, 3H, Ac), 2.00 - 1.64 (m, 8H, CH_2), 1.54 - 1.24 (m, 4H, CH_2); <u>HRMS</u> [$C_{31}H_{54}N_8O_{15}$ + H]$^+$: 779.3784 found,779.3781 calculated.

Ac-Lys(1,2-Man_2)-Gly-Lys(1,2-Man_2)-Gly-Lys-NH_2 (B2).

Azide **16** (4.58 mg, 7.51 µmol) was conjugated to propargyl mannosides **5** using the general click protocol. Compound **B2** was obtained after purification by gel filtration (instead of HW40S resin a superdex30, 1.6x60 cm, column was used with 150 mM NH_4HCO_3 aq. 1 mL/min as elution buffer; elutes at 65 - 76 mL)as a white powder (4.27 mg, 3.12 µmol, 41%). <u>LC-MS</u>: R_t = 4.31 min (0 - 50% ACN; 13 min); <u>HRMS</u> [$C_{54}H_{91}N_{13}O_{28}$ + H]$^+$: 1370.6175 found 1370.6169 calculated.

Ac-Lys(1,2-Man_2)-Gly-Lys(1,2-Man_2)-Gly-Lys(1,2-Man_2)-Gly-Lys-NH_2 (B3).

Azide **17** (4.20 mg, 5.12 µmol) was conjugated to propargyl mannosides **5** using the general click protocol. Compound **B3** was obtained after purification by gel filtration (34 - 44.5 mL) as a white powder (yield: 8.01 mg, 4.08 µmol, 80%). <u>LC-MS</u>: R_t = 4.45 min (0 - 50% ACN; 13 min); <u>^{1}H NMR</u> (500 MHz, D_2O) δ 8.07 (d, *J* = 2.3 Hz, 3H, trzl), 5.18 (s, 3H, H-1), 5.01 (s, 3H, H-1′), 4.83 (d, *J* = 12.4 Hz, 3H, O-C*H*H-trzl), 4.72 (d, *J* = 12.7 Hz, 3H,

O-CH*H*-trzl), 4.45 (t, *J* = 6.9 Hz, 6H, CH_2-trzl), 4.30 (tt, *J* = 9.4, 5.1 Hz, 3H, CH), 4.23 (dd, *J* = 8.5, 6.0 Hz, 1H, CH), 4.08 (dd, *J* = 3.3, 1.8 Hz, 3H, H-2), 4.00 - 3.59 (m, 42H, CH_2 (G), H-2′, H-3, H-3′, H-4, H-4′, H-5, H-5′, H-6, H-6′), 3.01 (t, *J* = 7.0 Hz, 2H, CH_2NH_2), 2.01 (s, 3H, Ac), 1.98 - 1.60 (m, 16H, CH_2), 1.55 - 1.28 (m, 8H, CH_2); HRMS $[C_{77}H_{128}N_{18}O_{41} + H]^+$: 1961.8643 found, 1961.8557 calculated.

Ac-Lys(1,2-Man₂)-Lys(1,2-Man₂)-Lys(1,2-Man₂)-Lys(1,2-Man₂)-Lys(1,2-Man₂)-Lys(1,2-Man₂)-Gly-Lys-NH₂ (B6).
Azide **18** (3.84 mg, 3.28 µmol) was conjugated to propargyl mannosides **5** using the general click protocol. Compound **B6** was obtained after purification by gel filtration (elution buffer contains 20% ACN, elutes at 28 - 38 mL) as a white powder (11.10 mg, 3.21 µmol, 98%). LC-MS: R_t = 4.51 min (0 - 50% ACN; 13 min); ^{1}H NMR (500 MHz, D_2O) δ 8.09 - 7.91 (m, 6H, trzl), 5.13 (s, 6H, H-1), 4.95 (s, 6H, H-1′), 4.74 (d, *J*=12.6, 6H, O-C*H*H-trzl), 4.63 (d, *J*=12.5, 6H, O-CH*H*-trzl), 4.36 (s, 12H, CH_2-trzl), 4.30 - 4.05 (m, 7H, CH), 4.02 (dd, *J* = 3.2, 1.7 Hz, 6H, H-2), 4.01 - 3.50 (m, 68H, CH_2 (G), H-2′, H-3, H-3′, H-4, H-4′, H-5, H-5′, H-6, H-6′), 2.98 - 2.92 (m, 2H, CH_2NH_2), 1.95 (s, 3H, Ac), 1.91 - 1.12 (m, 42H, CH_2); ^{13}C NMR (126 MHz, D_2O) δ 143.4, 125.0 (*C*H-trzl), 105.0 (CH_2), 102.3, 97.7 (C-1, C-1′), 78.6, 73.2, 72.9, 70.3, 70.1, 69.9, 69.5, 66.8, 66.7, 60.9, 60.8, 59.8, 50.0, 22.0; HRMS $[C_{136}H_{224}N_{28}O_{75} + 2H]^{2+}$: 1726.2408 found, 1726.2375 calculated.

Ac-Lys(1,3-Man₂)-Gly-Lys-NH₂ (C1).
Azide **15** (5.28 mg, 13.24 µmol)) was conjugated to propargyl mannosides **8** using the general click protocol. Compound **C1** was obtained after purification by gel filtration (elutes at 42-55 mL) followed by RP-HPLC (8 - 16% B, 12 min) as a white powder (10.16 mg, 13.05 µmol, 99%). LC-MS: Rt = 3.89 min (0 - 50% ACN; 13 min); ^{1}H NMR (500 MHz, D_2O) δ 8.07 (s, 1H, trzl), 5.12 (d, *J* = 1.6 Hz, 1H, H-1), 4.97 (d, *J* = 1.7 Hz, 1H, H-1′), 4.85 (d, *J* = 12.4 Hz, 1H, O-C*H*H-trzl), 4.76 - 4.72 (m, 1H, O-CH*H*-trzl), 4.47 (t, *J* = 7.1 Hz, 2H, CH_2-trzl), 4.32 (dd, *J* = 9.4, 5.0 Hz, 1H, CH), 4.23 (dd, *J* = 8.8, 5.8 Hz, 1H, CH), 4.09 (ddd, *J* = 12.9, 3.3, 1.8 Hz, 2H), 4.00 - 3.62 (m, 12H, CH_2 (G), H-2, H-2′, H-3, H-3′, H-4, H-4′, H-5, H-5′, H-6, H-6′), 3.06 - 2.95 (m, 2H, CH_2NH_2), 2.03 (s, 3H, Ac), 2.00 - 1.63 (m, 8H, CH_2), 1.54 - 1.27 (m, 4H, CH_2); HRMS $[C_{31}H_{54}N_8O_{15} + H]^+$: 779.3785 found, 779.3781 calculated.

Ac-Lys(1,3-Man₂)-Gly-Lys(1,3-Man₂)-Gly-Lys-NH₂ (C2).
Azide **16** (8.14 mg, 13.36 µmol) was conjugated to propargyl mannosides **8** using the general click protocol. Compound **C2** was obtained after purification by gel filtration (elutes at 38.5 - 46 mL) as a white powder (2.48 mg, 1.81 mol, 14%). LC-MS: R_t = 4.46 min (0 - 50% ACN; 13 min); HRMS $[C_{54}H_{91}N_{13}O_{28} + H]^+$: 1370.6176 found, 1370.6169 calculated.

Ac-Lys(1,3-Man₂)-Gly-Lys(1,3-Man₂)-Gly-Lys(1,3-Man₂)-Gly-Lys-NH₂ (C3).
Azide **17** (2.92 mg, 3.56 µmol) was conjugated to propargyl mannosides **8** using the general click protocol. Compound **3C** was obtained after purification by gel filtration (buffer contains 10% ACN, elutes at 33.5 - 42.5 mL) as a white powder (5.71 mg, 2.91 µmol, 82%). LC-MS: R_t = 4.37 min (0 - 50% ACN; 13 min); ^{1}H NMR (400 MHz, D_2O) δ 7.99 - 7.84 (m, 3H, trzl), 4.98 (s, 3H, H-1), 4.83 (d, *J* = 1.5 Hz, 3H, H-1′), 4.76 - 4.66 (m, 3H, O-C*H*H-trzl), 4.60 (d, *J* = 12.4 Hz, 3H, O-CH*H*-trzl), 4.32 (t, *J* = 6.8 Hz, 6H, CH_2-trzl), 4.22 - 4.12 (m, 3H, CH), 4.09 (dd, *J* = 8.7, 5.6 Hz, 1H, CH), 4.00 - 3.42 (m, 42H, CH_2 (G), H-2, H-2′, H-3, H-3′, H-4, H-4′, H-5, H-5′, H-6, H-6′), 2.85 (t, *J* = 7.5 Hz, 2H, CH_2NH_2), 1.87 (s, 3H, Ac), 1.85 - 1.09 (m, 24H, CH_2); HRMS $[C_{77}H_{128}N_{18}O_{41} + H]^+$: 1961.8589 found 1961.8557 calculated.

Ac-Lys(1,3-Man₂)-Lys(1,3-Man₂)-Lys(1,3-Man₂)-Lys(1,3-Man₂)-Lys(1,3-Man₂)-Lys(1,3-Man₂)-Gly-Lys-NH₂ (C6).
Azide **18** (3.78 mg, 3.23 µmol) was conjugated to propargyl mannosides **8** using the general click protocol. Compound **C6** was obtained after purification by gel filtration (elutes at 30.5 - 39 mL) as a white powder (11.04 mg, 3.19 µmol, 99%). LC-MS: R_t = 4.63 min (0 - 50% ACN; 13 min); ^{1}H NMR (500 MHz, D_2O) δ 7.92 (d, *J*

= 4.1 Hz, 6H, trzl), 4.97 (s, 6H, H-1), 4.81 (s, 6H, H-1′), 4.69 - 4.66 (m, 6H, O-C*H*H-trzl), 4.56 (d, *J* = 12.6 Hz, 6H, O-CH*H*-trzl), 4.28 (s, 12H, CH_2-trzl), 4.19 - 3.97 (m, 7H, CH), 3.97 - 3.48 (m, 74H, CH_2 (G), H-2, H-2′, H-3, H-3′, H-4, H-4′, H-5, H-5′, H-6, H-6′), 2.79 - 2.72 (m, 2H, CH_2NH_2), 1.87 (s, 3H, Ac), 1.84 - 1.44 (m, 28H, CH_2), 1.40 - 1.05 (m, 14H, CH_2); ^{13}C NMR (126 MHz, D_2O) δ 143.5, 125.0, 102.4, 99.5 (C-1, C-1′), 78.3, 73.4, 73.1, 70.4, 70.1, 69.6, 66.8, 66.0, 61.0, 60.8, 59.9, 50.1, 28.9; HRMS $[C_{136}H_{224}N_{28}O_{75} + 2H]^{2+}$: 1726.2405 found, 1726.2375 calculated.

Ac-Lys(1,6-Man$_2$)-Gly-Lys-NH$_2$ (D1).

Azide **15** (4.81 mg, 12.06 µmol) was conjugated to propargyl mannosides **11** using the general click protocol. Compound **D1** was obtained after purification by gel filtration (elutes at 51 - 60 mL) followed by RP-HPLC (8 - 16% B, 12 min) as a white powder (7.99 mg, 10.26 µmol, 85%). LC-MS: R_t = 3.95 min (0 - 50% ACN; 13 min); HRMS $[C_{31}H_{54}N_8O_{15} + H]^+$: 779.3785 found, 779.3781 calculated.

Ac-Lys(1,6-Man$_2$)-Gly-Lys(1,6-Man$_2$)-Gly-Lys-NH$_2$ (D2).

Azide **16** (4.22 mg, 6.92 µmol) was conjugated to propargyl mannosides **11** using the general click protocol. Compound **D2** was obtained after purification by gel filtration (elutes at 39.5 - 47 mL) as a white powder (2.69 mg, 1.96 µmol, 28%). LC-MS: R_t = 4.31 min (0 - 50% ACN; 13 min); HRMS $[C_{54}H_{91}N_{13}O_{28} + H]^+$: 1370.6177 found, 1370.6169 calculated.

Ac-Lys(1,6-Man$_2$)-Gly-Lys(1,6-Man$_2$)-Gly-Lys(1,6-Man$_2$)-Gly-Lys-NH$_2$ (D3).

Azide **17** (3.92 mg, 3.56 µmol) was conjugated to propargyl mannosides **11** using the general click protocol. Compound **D3** was obtained after purification by gel filtration (elutes at 34 - 41.5 mL) as a white powder (5.96 mg, 3.04 µmol, 85%). LC-MS: R_t = 4.37 min (0 - 50% ACN; 13 min); ^{1}H NMR (500 MHz, D_2O) δ 8.03 - 7.91 (m, 3H, trzl), 4.88 (s, 3H, H-1), 4.83 (s, 3H, H-1′), 4.74 (d, *J* = 12.6 Hz, 3H, O-C*H*H-trzl), 4.64 (d, *J* = 12.5 Hz, 3H, O-CH*H*-trzl), 4.37 (t, *J* = 6.9 Hz, 6H, CH_2-trzl), 4.21 (tt, *J* = 9.2, 5.3 Hz, 3H, CH), 4.14 (dd, *J* = 8.8, 5.7 Hz, 1H, CH), 3.95 - 3.56 (m, 42H, CH_2 (G), H-2, H-2′, H-3, H-3′, H-4, H-4′, H-5, H-5′, H-6, H-6′), 2.88 (t, *J* = 7.1 Hz, 2H, CH_2NH_2), 1.92 (s, 3H, Ac), 1.90 - 1.51 (m, 16H, CH_2), 1.44 - 1.14 (m, 8H, CH_2); HRMS $[C_{77}H_{128}N_{18}O_{41} + H]^+$: 1961.8616 found, 1961.8557 calculated.

Ac-Lys(1,6-Man$_2$)-Lys(1,6-Man$_2$)-Lys(1,6-Man$_2$)-Lys(1,6-Man$_2$)-Lys(1,6-Man$_2$)-Lys(1,6-Man$_2$)-Gly-Lys-NH$_2$ (D6).

Azide **18** (2.63 mg, 2.25 µmol) was conjugated to propargyl mannosides **11** using the general click protocol. Compound **D6** was obtained after purification by gel filtration (elutes at 36 - 50 mL) as a white powder (4.45 mg, 1.29 µmol, 57%). LC-MS: R_t = 4.63 min (0 - 50% ACN; 13 min); HRMS $[C_{136}H_{224}N_{28}O_{75} + 2H]^{2+}$: 1726.2408 found, 1726.2375 calculated.

Ac-Lys(Man$_3$)-Gly-Lys-NH$_2$ (E1).

Azide **15** (5.52 mg, 13.84 µmol) was conjugated to propargyl mannosides **14** using the general click protocol. Compound **E1** was obtained after purification by gel filtration (elutes at 46 - 55 mL) followed by RP-HPLC (8 - 16% B, 12 min) as a white powder (12.99 mg, 13.80 µmol, 99%). LC-MS: R_t = 4.15 min (0 - 50% ACN; 13 min); ^{1}H NMR (500 MHz, D_2O) δ 8.06 (s, 1H, trzl), 5.09 (s, 1H), 4.94 (s, 1H), 4.90 (s, 1H, H-1, H-1′, H-1′′), 4.82 (d, *J* = 12.4 Hz, 1H, O-C*H*H-trzl), 4.74 (d, *J* = 12.6 Hz, 1H, O-CH*H*-trzl), 4.46 (t, *J* = 6.8 Hz, 2H, CH*2*-trzl), 4.30 (dd, *J* = 9.4, 4.9 Hz, 1H, CH), 4.22 (dd, *J* = 8.6, 5.8 Hz, 1H, CH), 4.14 - 3.58 (m, 20H, CH_2(G), H-2, H-2′, H-2′′, H-3, H-3′, H-3′′, H-4, H-4′, H-4′′, H-5, H-5′, H-5′′, H-6, H-6′, H-6′′), 3.00 (t, *J* = 7.4 Hz, 2H, CH_2NH_2), 2.02 (s, 3H, Ac), 1.92 - 1.23 (m, 18H, CH_2); HRMS $[C_{37}H_{64}N_8O_{20} + H]^+$: 941.4316 found, 941.4310 calculated.

Ac-Lys(Man₃)-Gly-Lys(Man₃)-Gly-Lys-NH₂ (E2).

Azide **16** (7.91 mg, 12.98 µmol) was conjugated to propargyl mannosides **14** using the general click protocol. Compound **E2** was obtained after purification by gel filtration (elutes at 34 - 44.5 mL) as a white powder (9.44 mg, 5.57 µmol, 43%). LC-MS: R_t = 4.18 min (0 - 50% ACN; 13 min); HRMS [$C_{66}H_{111}N_{13}O_{38}$ + H]$^+$: 1694.7229 found, 1694.7226 calculated.

Ac-Lys(Man₃)-Gly-Lys(Man₃)-Gly-Lys(Man₃)-Gly-Lys-NH₂ (E3).

Azide **17** (2.92 mg, 3.56 µmol) was conjugated to propargyl mannosides **14** using the general click protocol. Compound **E3** was obtained after purification by gel filtration (elutes at 31- 39.5 mL) as a white powder (6.97 mg, 2.85 µmol, 80%). LC-MS: R_t = 4.54 min (0 - 50% ACN; 13 min); ^{1}H NMR (500 MHz, D_2O) δ 8.06 (d, *J* = 1.8 Hz, 3H, trzl), 5.10 (s, 3H), 4.95 (d, *J* = 1.5 Hz, 3H), 4.91 (d, *J* = 1.2 Hz, 3H, H-1, H-1′ & H-1′′), 4.83 (d, *J* = 12.7 Hz, 3H, O-C*H*H-trzl), 4.74 (d, *J* = 12.6 Hz, 3H, O-CH*H*-trzl), 4.46 (t, *J* = 6.9 Hz, 6H, CH_2-trzl), 4.35 - 4.27 (m, 3H, CH), 4.23 (dd, *J* = 8.8, 5.7 Hz, 1H, CH), 4.13 - 3.60 (m, 60H, CH_2(G), H-2, H-2′, H-2′′, H-3, H-3′, H-3′′, H-4, H-4′, H-4′′, H-5, H-5′, H-5′′, H-6, H-6′, H-6′′), 2.95 (t, *J* = 7.3 Hz, 2H, C*H₂*NH₂), 2.01 (s, 3H, Ac), 1.99 - 1.23 (m, 24H, CH_2); HRMS [$C_{95}H_{158}N_{18}O_{56}$ + 2H]$^{2+}$: 1225.0138 found, 1225.01225 calculated.

Ac-Lys(Man₃)-Lys(Man₃)-Lys(Man₃)-Lys(Man₃)-Lys(Man₃)-Lys(Man₃)-Gly-Lys-NH₂ (E6).

Azide **18** (6.90 mg, 5.90 µmol) was conjugated to propargyl mannosides **14** using the general click protocol. Compound **E6** was obtained after purification by gel filtration (elutes at 28 - 39 mL) as a white powder (22.22 mg, 5.02 µmol, 85%). LC-MS: R_t = 4.34 min (0 - 50% ACN; 13 min); ^{1}H NMR (500 MHz, D_2O) δ 8.01 (s, 6H, trzl), 5.04 (s, 6H), 4.89 (s, 6H), 4.86 (s, 6H, H-1, H-1′, H-1′′), 4.77 - 4.73 (m, 6H, O-C*H*H-trzl), 4.69 - 4.62 (m, 6H, O-CH*H*-trzl), 4.37 (s, 12H, CH_2-trzl), 4.28 - 4.14 (m, 7H, CH), 4.13 - 3.50 (m, 110H, CH_2(G), H-2, H-2′, H-2′′, H-3, H-3′, H-3′′, H-4, H-4′, H-4′′, H-5, H-5′, H-5′′, H-6, H-6′, H-6′′), 2.93 (t, *J* = 6.7 Hz, 2H, CH_2NH_2), 1.96 (s, 3H, Ac), 1.91 - 1.14 (m, 42H, CH_2); ^{13}C NMR (126 MHz, D_2O) δ 143.4, 124.9, 102.4, 99.6, 99.4 (C-1,C-1′& C-1′′), 78.6, 73.3, 72.6, 71.2, 70.6, 70.3, 70.0, 69.9, 69.5, 69.5, 66.7, 65.4, 64.9, 60.9, 60.0, 50.0, 39.2, 28.9, 22.0; HRMS [$C_{172}H_{284}N_{28}O_{105}$ + 2H]$^{2+}$: 2212.9033 found, 2212.8975 calculated.

Ac-Lys(Gal)-Gly-Lys-NH₂ (G1).

Azide **15** (5.54 mg, 13.90 µmol) was conjugated to propargyl galactoside **S8** using the general click protocol. Compound **G1** was obtained after purification by gel filtration (elutes at 46.5 - 51 mL) as a white powder (4.93 mg, 7.99 µmol, 57%). LC-MS: R_t = 4.02 min (0 - 50% ACN; 13 min); ^{1}H NMR (500 MHz, D_2O) δ 7.97 (s, 1H, trzl), 5.03 (s, 1H, H-1), 4.75 (d, *J* = 13.4 Hz, 1H, O-C*H*H-trzl), 4.73 - 4.67 (m, 1H, O-CH*H*-trzl), 4.39 (t, *J* = 6.8 Hz, 2H, CH_2-trzl), 4.23 (dd, *J* = 9.5, 5.0 Hz, 1H, CH), 4.14 (dd, *J* = 8.7, 5.7 Hz, 1H, CH), 4.00 (dd, *J* = 3.6, 1.7 Hz, 2H, CH_2), 3.92 - 3.71 (m, 4H, H-2, H-3, H-4, H-5), 3.64 - 3.50 (m, 2H, H-6), 2.93 (td, *J* = 8.0, 2.1 Hz, 2H, C*H₂*NH₂), 1.94 (s, 3H, Ac), 1.92 - 1.14 (m, 12H, CH_2); HRMS [$C_{25}H_{44}N_8O_{10}$ + H]$^+$: 617.32531 found, 617.3253 calculated.

Ac-Lys(Gal)-Gly-Lys(Gal)-Gly-Lys-NH₂ (G2).

Azide **16** (6.07 mg, 9.95 µmol) was conjugated to propargyl galactoside **S8** using the general click protocol. Compound **G2** was obtained after purification by gel filtration (elutes at 44 - 52 mL) as a white powder (5.54 mg, 5.29 µmol, 53%). LC-MS: R_t = 4.38 min (0 - 50% ACN; 13 min); ^{1}H NMR (500 MHz, D_2O) δ 7.97 (s, 2H, trzl), 5.06 - 4.91 (m, 2H, H-1), 4.72 - 4.68 (m, 4H, O-CH_2-trzl), 4.38 (t, *J* = 6.7 Hz, 4H, CH_2-trzl), 4.27 - 4.17 (m, 2H, CH), 4.14 (dd, *J* = 8.8, 5.7 Hz, 1H, CH), 4.04 - 3.98 (m, 4H, CH_2 (G)), 3.92 - 3.71 (m, 8H, H-2, H-3, H-4, H-5), 3.64 - 3.50 (m, 4H, H-6), 2.92 (td, *J* = 8.0, 2.2 Hz, 2H, C*H₂*NH₂), 1.93 (s, 3H, Ac), 1.91 - 1.07 (m, 18H, CH_2); HRMS [$C_{42}H_{71}N_{13}O_{18}$ + H]$^+$: 1046.51147 found, 1046.5113 calculated.

General procedure for biotinylation

The "general procedure" to introduce the biotin handle: Glycoclusters described above with a free amine (**A1 - E6**) were dissolved in DMSO (0.02 M). To this, a stock solution of Biotin-OSu (0.15 M, 3-4 eq) and DIPEA (0.015M, 0.3-0.4 eq) in DMSO was added and shaken overnight after which compounds were purified via RP-HPLC (linear gradient 10 - 16 % B in A, 12 min, 5 mL/min, Develosil RPAQUEOUS 10.0 x 250 mm) followed by lyophilization.

Ac-Lys(Man$_1$)-Gly-Lys(biotin)-NH$_2$ (a1).
Compound **A1** (1.01 mg, 1.64 µmol) was coupled with biotin-OSu using the general procedure. Compound **a1** was obtained after purification by RP-HPLC as a white powder (1.37 mg, 1.62 µmol, 99%). LC-MS: R_t = 5.25 min (0 - 50% ACN; 13 min); HRMS [$C_{35}H_{58}N_{10}O_{12}S$ + H]$^+$: 843.4031 found, 843.4029 calculated.

Ac-Lys(Man$_1$)-Gly-Lys(Man$_1$)-Gly-Lys(biotin)-NH$_2$ (a2).
Compound **A2** (11.06 mg, 10.57 µmol) was coupled with biotin-OSu using the general procedure. Compound **a2** was obtained after purification by RP-HPLC as a white powder (2.62 mg, 2.06 µmol, 19%). LC-MS: R_t = 5.24 min (0 - 50% ACN; 13 min); HRMS [$C_{52}H_{85}N_{15}O_{20}S$ + 2H]$^{2+}$: 636.7984 found, 636.7981 calculated.

Ac-Lys(Man$_1$)-Gly-Lys(Man$_1$)-Gly-Lys(Man$_1$)-Gly-Lys(biotin)-NH$_2$ (a3).
Compound **A3** (2.63 mg, 1.78 µmol) was coupled with biotin-OSu using the general procedure. Compound **a3** was obtained after purification by RP-HPLC as a white powder (1.92 mg, 1.13 µmol, 63%). LC-MS: R_t = 5.22 min (0 - 50% ACN; 13 min); HRMS [$C_{69}H_{112}N_{20}O_{28}S$ +2H]$^{2+}$: 851.3912 found, 851.3911 calculated.

Ac-Lys(Man$_1$)-Lys(Man$_1$)-Lys(Man$_1$)-Lys(Man$_1$)-Lys(Man$_1$)-Lys(Man$_1$)-Gly-Lys(biotin)-NH$_2$ (a6).
Compound **A6** (1.75 mg, 0.72 µmol) was coupled with biotin-OSu using the general procedure. Compound **a6** was obtained after purification by RP-HPLC as a white powder (1.14 mg, 0.42 µmol, 59%). LC-MS: R_t = 5.22 min (0 - 50% ACN; 13 min); HRMS [$C_{110}H_{178}N_{30}O_{47}S$ + 2H]$^{2+}$: 1353.1169 found, 1353.1178 calculated.

Ac-Lys(1,2-Man$_2$)-Gly-Lys(biotin)-NH$_2$ (b1).
Compound **B1** (8.97 mg, 11.51 µmol) was coupled with biotin-OSu using the general procedure. Compound **b1** was obtained after purification by RP-HPLC as a white powder (1.90 mg, 1.89 µmol, 16%). LC-MS: R_t = 5.18 min (0 - 50% ACN; 13 min); HRMS [$C_{41}H_{68}N_{10}O_{17}S$ + 2H]$^{2+}$: 503.2315 found, 503.2315 calculated.

Ac-Lys(1,2-Man$_2$)-Gly-Lys(1,2-Man$_2$)-Gly-Lys(biotin)-NH$_2$ (b2).
Compound **B2** (2.67 mg, 1.95 µmol) was coupled with biotin-OSu using the general procedure. Compound **b2** was obtained after purification by RP-HPLC as a white powder (1.52 mg, 0.95 µmol, 49%). LC-MS: R_t = 5.14 min (0 - 50% ACN; 13 min); HRMS [$C_{64}H_{105}N_{15}O_{30}S$+2H]$^{2+}$: 798.8514 found, 798.8509 calculated.

Ac-Lys(1,2-Man$_2$)-Gly-Lys(1,2-Man$_2$)-Gly-Lys(1,2-Man$_2$)-Gly-Lys(biotin)-NH$_2$ (b3).
Compound **B3** (4.91 mg, 2.50 µmol) was coupled with biotin-OSu using the general procedure. Compound **b3** was obtained after purification by RP-HPLC as a white powder (1.82 mg, 0.83 µmol, 33%). LC-MS: R_t = 5.07 min (0 - 50% ACN; 13 min); HRMS [$C_{87}H_{142}N_{20}O_{43}S$ + 2H]$^{2+}$: 1094.9727 found, 1094.9718 calculated.

Ac-Lys(1,2-Man$_2$)-Lys(1,2-Man$_2$)-Lys(1,2-Man$_2$)-Lys(1,2-Man$_2$)-Lys(1,2-Man$_2$)-Lys(1,2-Man$_2$)-Gly-Lys(biotin)-NH$_2$ (b6).
Compound **B6** (5.24 mg, 1.60 µmol) was coupled with biotin-OSu using the general procedure. Compound **b6** was obtained after purification by RP-HPLC as a white powder (3.65 mg, 0.99 µmol, 62%). LC-MS: R_t = 4.94 min (0 - 50% ACN; 13 min); HRMS [$C_{146}H_{238}N_{30}O_{77}S$ + 3H]$^{3+}$: 1226.5201 found, 1226.5200 calculated.

Ac-Lys(1,3-Man$_2$)-Gly-Lys(biotin)-NH$_2$ (c1).
Compound **C1** (6.46 mg, 8.30 µmol) was coupled with biotin-OSu using the general procedure. Compound **c1** was obtained after purification by RP-HPLC as a white powder (1.44 mg, 1.44 µmol, 17%). LC-MS: R_t = 5.23 min (0 - 50% ACN; 13 min); HRMS [$C_{41}H_{68}N_{10}O_{17}S$ +2H]$^{2+}$: 503.2316 found, 503.2315 calculated.

Ac-Lys(1,3-Man$_2$)-Gly-Lys(1,3-Man$_2$)-Gly-Lys(biotin)-NH$_2$ (c2).
Compound **C2** (1.50 mg, 1.10 µmol) was coupled with biotin-OSu using the general procedure. Compound **c2** was obtained after purification by RP-HPLC as a white powder (1.74 mg, 1.09 µmol, 99%). LC-MS: R_t = 5.18 min (0 - 50% ACN; 13 min); HRMS [$C_{64}H_{105}N_{15}O_{30}S$+2H]$^{2+}$: 798.8516 found, 798.8509 calculated.

Ac-Lys(1,3-Man$_2$)-Gly-Lys(1,3-Man$_2$)-Gly-Lys(1,3-Man$_2$)-Gly-Lys(biotin)-NH$_2$ (c3).
Compound **C3** (3.53 mg, 1.80 µmol) was coupled with biotin-OSu using the general procedure. Compound **c3** was obtained after purification by RP-HPLC as a white powder (3.78 mg, 1.72 µmol, 96%). LC-MS: R_t = 5.13 min (0 - 50% ACN; 13 min); HRMS [$C_{87}H_{142}N_{20}O_{43}S$ + 2H]$^{2+}$: 1094.9719 found, 1094.9718 calculated.

Ac-Lys(1,3-Man$_2$)-Lys(1,3-Man$_2$)-Lys(1,3-Man$_2$)-Lys(1,3-Man$_2$)-Lys(1,3-Man$_2$)-Lys(1,3-Man$_2$)-Gly-Lys(biotin)-NH$_2$ (c6).
Compound **C6** (5.24 mg, 1.60 µmol) was coupled with biotin-OSu using the general procedure. Compound **c6** was obtained after purification by RP-HPLC as a white powder (3.65 mg, 0.99 µmol, 62%). LC-MS: R_t = 5.00 min (0 - 50% ACN; 13 min); HRMS [$C_{146}H_{238}N_{30}O_{77}S$ + 3H]$^{3+}$: 1226.5195 found, 1226.5200 calculated.

Ac-Lys(1,6-Man$_2$)-Gly-Lys(biotin)-NH$_2$ (d1).
Compound **D1** (1.50 mg, 1.93 µmol) was coupled with biotin-OSu using the general procedure. Compound **d1** was obtained after purification by RP-HPLC as a white powder (0.97 mg, 0.97 µmol, 50%). LC-MS: R_t = 5.23 min (0 - 50% ACN; 13 min); HRMS [$C_{41}H_{68}N_{10}O_{17}S$ + 2H]$^{2+}$: 503.2314 found, 503.2315 calculated.

Ac-Lys(1,6-Man$_2$)-Gly-Lys(1,6-Man$_2$)-Gly-Lys(biotin)-NH$_2$ (d2).
Compound **D2** (1.68 mg, 1.22 µmol) was coupled with biotin-OSu using the general procedure. Compound **d2** was obtained after purification by RP-HPLC as a white powder (1.48 mg, 0.93 µmol, 76%). LC-MS: R_t = 5.11 min (0 - 50% ACN; 13 min); HRMS [$C_{64}H_{105}N_{15}O_{30}S$ + 2H]$^{2+}$: 798.8512 found, 798.8509 calculated.

Ac-Lys(1,6-Man$_2$)-Gly-Lys(1,6-Man$_2$)-Gly-Lys(1,6-Man$_2$)-Gly-Lys(biotin)-NH$_2$ (d3).
Compound **D3** (3.53 mg, 1.80 µmol) was coupled with biotin-OSu using the general procedure. Compound **d3** was obtained after purification by RP-HPLC as a white powder (3.55 mg, 1.62 µmol, 90%). LC-MS: R_t = 5.09 min (0 - 50% ACN; 13 min); HRMS [$C_{87}H_{142}N_{20}O_{43}S$ + 2H]$^{2+}$: 1094.9721 found, 1094.9718 calculated.

Ac-Lys(1,6-Man$_2$)-Lys(1,6-Man$_2$)-Lys(1,6-Man$_2$)-Lys(1,6-Man$_2$)-Lys(1,6-Man$_2$)-Lys(1,6-Man$_2$)-Gly-Lys(biotin)-NH$_2$ (d6).
Compound **D6** (1.82 mg, 0.53 µmol) was coupled with biotin-OSu using the general procedure. Compound **d6** was obtained after purification by RP-HPLC as a white powder (1.08 mg, 0.29 µmol, 56%). LC-MS: R_t = 4.94 min (0 - 50% ACN; 13 min); HRMS [$C_{146}H_{238}N_{30}O_{77}S$ + 3H]$^{3+}$: 1226.5203 found, 1226.5200 calculated.

Ac-Lys(Man$_3$)-Gly-Lys(biotin)-NH$_2$ (e1).

Compound **E1** (8.25 mg, 8.77 µmol) was coupled with biotin-OSu using the general procedure. Compound **e1** was obtained after purification by RP-HPLC as a white powder (1.48 mg, 1.26 µmol, 14%). LC-MS: R_t = 5.15 min (0 - 50% ACN; 13 min); HRMS [$C_{47}H_{78}N_{10}O_{22}S$ + 2H]$^{2+}$: 584.2582 found, 584.2579 calculated.

Ac-Lys(Man$_3$)-Gly-Lys(Man$_3$)-Gly-Lys(biotin)-NH$_2$ (e2).

Compound **E2** (6.02 mg, 3.55 µmol) was coupled with biotin-OSu using the general procedure. Compound **e2** was obtained after purification by RP-HPLC as a white powder (3.34 mg, 1.74 µmol, 49%). LC-MS: R_t = 5.02 min (0 - 50% ACN; 13 min); HRMS [$C_{76}H_{125}N_{15}O_{40}S$+ 2H]$^{2+}$: 960.9051 found, 960.9037 calculated.

Ac-Lys(Man$_3$)-Gly-Lys(Man$_3$)-Gly-Lys(Man$_3$)-Gly-Lys(biotin)-NH$_2$ (e3).

Compound **E3** (4.41 mg, 1.80 µmol) was coupled with biotin-OSu using the general procedure. Com-pound **e3** was obtained after purification by RP-HPLC as a white powder (2.44 mg, 0.91 µmol, 51%). LC-MS: R_t = 4.94 min (0 - 50% ACN; 13 min); HRMS [$C_{105}H_{172}N_{20}O_{58}S$ + 2H]$^{2+}$: 1338.0503 found, 1338.0510 calculated.

Ac-Lys(Man$_3$)-Lys(Man$_3$)-Lys(Man$_3$)-Lys(Man$_3$)-Lys(Man$_3$)-Lys(Man$_3$)-Gly-Lys(biotin)-NH$_2$ (e6).

Compound **E6** (8.40 mg, 1.90 µmol) was coupled with biotin-OSu using the general procedure. Compound **e6** was obtained after purification by RP-HPLC as a white powder (7.50 mg, 1.61 µmol, 85%). LC-MS: R_t = 4.68 min (0 - 50% ACN; 13 min); HRMS [$C_{182}H_{298}N_{30}O_{107}S$ + 3H]$^{3+}$: 1550.9603 found, 1550.9599 calculated.

Other generated building blocks

Pent-4-ynoic acid succinimidyl ester (S9).

Synthesis and spectral data were as described in previous literature(Horatscheck et al., 2012).

4-((6-amino-2-butoxy-7-(tert-butoxycarbonyl)-8-oxo-7,8-dihydro-9H-purin-9-yl) methyl) benzoic acid (23).

Synthesis and spectral data were as described in previous literature(Gential et al., 2019).

General procedure for alkyne introduction

The "general procedure" for introduction of the alkyne: A solution of Glycoclusters with a free amine (**B6, C6, D6** or **E6**) (0.2 M, aq., 1 eq) was mixed with a stock solution of **S9** (0.15 M, 3 eq) and DIPEA (0.05 M, 1 eq) in DMSO and shaken for one hour. Reaction progress was followed via LC-MS and when completed, the 4-pentynoic amides were purified via gel filtration (Toyopearl HW-40S, 1.6x60 cm, 150 mM NH_4HCO_3 aq.; sometimes the elution buffer contained a percentage of ACN, 1 ml/min) followed by lyophilization.

Ac-Lys(1,2-Man$_2$)-Lys(1,2-Man$_2$)-Lys(1,2-Man$_2$)-Lys(1,2-Man$_2$)-Lys(1,2-Man$_2$)-Lys(1,2-Man$_2$)-Gly-Lys(pent-4-ynoic amide)-NH$_2$ (19).

Compound **B6** (7.06 mg, 2.04 µmol) was coupled with **S9** using the general procedure. Compound **19** was obtained after purification by gel filtration (buffer with 20% ACN, eluted at 25 - 40 mL) as a white powder (6.95 mg, 1.96 µmol, 96%). LC-MS: R_t = 4.79 min (0 - 50% ACN; 13 min); ^{1}H NMR (500 MHz, D_2O) δ 7.93 - 7.88 (m, 6H, trzl), 5.04 (s, 6H, H-1), 4.86 (s, 6H, H-1′), 4.66 (d, *J* = 12.4 Hz, 6H, O-C*H*H-trzl), 4.54 (d, *J* = 12.7 Hz, 6H, O-CH*H*-trzl), 4.27 (t, *J* = 6.4 Hz, 12H, CH_2-trzl), 4.18 - 3.97 (m, 7H, CH), 3.96 - 3.43 (m, 74H, CH_2 (G), H-2, H-2′, H-3, H-3′, H-4, H-4′, H-5, H-5′, H-6, H-6′), 3.03 (t, *J* = 6.4 Hz, 2H, C*H$_2$*NH$_2$), 2.35 - 2.17 (m, 5H, CH_2, CH_2, C≡CH), 1.87 (s, 3H, Ac), 1.82 - 1.03 (m, 42H, CH_2); HRMS [$C_{141}H_{228}N_{28}O_{76}$ + 3H]$^{3+}$: 1177.83838 found, 1177.83618 calculated.

Ac-Lys(1,3-Man$_2$)-Lys(1,3-Man$_2$)-Lys(1,3-Man$_2$)-Lys(1,3-Man$_2$)-Lys(1,3-Man$_2$)-Lys(1,3-Man$_2$)-Gly-Lys(pent-4-ynoic amide)-NH$_2$ (20).

A solution of compound **C6** (150 µL, 5 mM, 750 nmol) was coupled with **S9** using the general procedure. Compound **20** was obtained after purification by gel filtration (buffer contained 20% ACN, eluted at 23.5 - 40 mL) as a white powder (2.23 mg, 645 nmol, 95%). LC-MS: R_t = 4.60 min (0 - 50% ACN; 13 min); ^{1}H NMR (500 MHz, D_2O) δ 7.93 - 7.88 (m, 6H, trzl), 4.96 (s, 6H, H-1), 4.80 (s, 6H, H-1′), 4.66 (d, *J* = 5.8 Hz, 6H, O-C*H*H-trzl), 4.55 (d, *J* = 12.6 Hz, 6H, O-CH*H*-trzl), 4.26 (d, *J* = 6.3 Hz, 12H, CH_2-trzl), 4.14 - 3.96 (m, 7H, CH), 3.96 - 3.43 (m, 74H, CH_2 (G), H-2, H-2′, H-3, H-3′, H-4, H-4′, H-5, H-5′, H-6, H-6′), 3.03 (t, *J* = 6.7 Hz, 2H, C*H$_2$*NH$_2$), 2.39 - 2.16 (m, 5H, CH_2, CH_2, C≡CH), 1.86 (s, 3H, Ac), 1.82 - 1.04 (m, 42H, CH_2); HRMS [$C_{141}H_{228}N_{28}O_{76}$ + 3H]$^{3+}$: 1177.83747 found, 1177.83618 calculated.

Ac-Lys(1,6-Man$_2$)-Lys(1,6-Man$_2$)-Lys(1,6-Man$_2$)-Lys(1,6-Man$_2$)-Lys(1,6-Man$_2$)-Lys(1,6-Man$_2$)-Gly-Lys(pent-4-ynoic amide)-NH$_2$ (21).

Compound **D6** (2.35 mg, 679 nmol) was coupled with **S9** using the general procedure. Compound **21** was obtained after purification by gel filtration (eluted at 31.5 - 42 mL) as a white powder (2.23 mg, 645 nmol, 95%). LC-MS: R_t = 4.76 min (0 - 50% ACN; 13 min); HRMS [$C_{141}H_{228}N_{28}O_{76}$ + 3H]$^{3+}$: 1177.83621 found, 1177.83618 calculated.

Ac-Lys(Man$_3$)-Lys(Man$_3$)-Lys(Man$_3$)-Lys(Man$_3$)-Lys(Man$_3$)-Lys(Man$_3$)-Gly-Lys(pent-4-ynoic amide)-NH$_2$ (22).

Compound **E6** (18.77 mg, 4.24 µmol) was coupled with **S9** using the general procedure. Compound **22** was obtained after purification by gel filtration (buffer contained 20% ACN, eluted at 26 - 38.5 mL) as a white powder (17.96 mg, 3.98 µmol, 94%). LC-MS: R_t = 4.64 min (0 - 50% ACN; 13 min); ^{1}H NMR (500 MHz, D_2O) δ 7.92 (s, 6H, trzl), 4.95 (s, 6H, H-1), 4.80 (s, 6H, H-1′), 4.76 (s, 6H , H-1″), 4.68 - 4.63 (m, 6H, O-C*H*H-trzl), 4.57 (d, *J* = 12.7 Hz, 6H, O-CH*H*-trzl), 4.28 (t, *J* = 6.3 Hz, 12H, CH_2-trzl), 4.18 - 4.05 (m, 6H, CH), 4.05 - 3.39 (m, 111H, CH, CH_2 (G), H-2, H-2′, H-2″, H-3, H-3′, H-3″, H-4, H-4′, H-4″, H-5, H-5′, H-5″, H-6, H-6′, H-6″), 3.04 (t, *J* = 6.7 Hz, 2H, C*H$_2$*NH$_2$), 2.41 - 2.18 (m, 5H, CH_2, CH_2, C≡CH), 1.87 (s, 3H, Ac), 1.84 - 1.01 (m, 42H, CH_2); ^{13}C NMR (126 MHz, D_2O) δ 143.5, 124.2 (CH trzl), 102.4, 99.7, 99.4 (C-1, C-1′, C-1″), 78.6, 73.3, 72.7, 71.2, 70.6, 70.3, 70.0, 69.9, 69.5, 69.5, 66.7, 65.4, 64.9, 60.9 (C-2, C-2′, C-2″, C-3, C-3′, C-3″, C-4, C-4′, C-4″, C-5, C-5′, C-5″, C-6, C-6′, C-6″), 59.8 (O-*C*H$_2$trzl), 53.9, 53.5 (CH), 50.0 (*C*H$_2$-trzl), 42.4 (CH_2 (G)), 39.1 (CH_2NH_2), 34.5 (CH_2), 30.0, 28.9, 22.0 (CH_2), 21.7 (Ac), 14.6 (CH_2); HRMS [$C_{177}H_{288}N_{28}O_{106}$ + 3H]$^{3+}$: 1502.27589 found, 1502.27616 calculated.

General procedure for automated solid phase synthesis of gp100 peptides

The solid-phase peptide synthesis was performed on a TRIBUTE® Peptide Synthesizer (Gyros Protein Technologies AB, Arizona, USA) applying Fmoc based protocol starting with Tentagel S-RAM resin (~0.22 mmol/g) on a 100-250 µmol scale using established synthetic protocols.(Chan and White, 2000) The consecutive steps for synthesis on 250 µmol scale* performed in each cycle were:

1) DMF wash (1x) followed by nitrogen purge; *2)* Deprotection of the Fmoc-group with 20% piperidine in DMF (8 mL)(3 x 3 min at 50 °C); *3)* DMF wash (3x) followed by nitrogen purge; *4.1)* Coupling of the appropriate amino acid** in four-fold excess (unless stated otherwise)***; *4.2)* Step 4.1 was repeated *5)* DMF wash (3x) followed by nitrogen purge; *6)* capping with a solution of Ac_2O/DMF/DIPEA (8mL, 10/88/2, v/v/v) for 2 min; *7)* DMF wash (2x).

After the complete sequence the resin was washed with DMF (3x), DCM (3x), Et_2O (3x), followed by nitrogen purge before treatment with the cleavage cocktail.

*All amounts are scaled-down in equimolar proportions for smaller scale.

The amino acids applied in this synthesis were: Fmoc-Lys(Mmt)-OH, Fmoc-Gly-OH, Fmoc-Arg(Pbf)-OH, Fmoc-Trp(Boc)-OH, Fmoc-L-α-aminobutyric acid, Fmoc-Asp(O*t*Bu)-OH**, Fmoc-Leu-OH****, Fmoc-Gln(Trt)-OH, Fmoc-Ala-OH, Fmoc-Glu(O*t*Bu)-OH, Fmoc-Thr(*t*Bu)-OH, Fmoc-Pro-OH, Fmoc-Tyr(*t*Bu)-OH, Fmoc-Asn(Trt)-OH, Fmoc-Val-OH, Fmoc-His(Trt)-OH, Fmoc-AEEA-OH *(Fmoc-8-amino-3,6-dioxaoctanoic acid)* (Carbosynth), Fmoc-Cys(Trt)-OH, Fmoc-Lys(N_3)-OH (IRIS biotech) and **23**.

***Generally, the Fmoc amino acid is dissolved in a HCTU solution in DMF (5.00 mL ,0.20 M, 1.0 mmol, 4 eq) The resulting solution was transferred to the reaction vessel followed by a DIPEA solution in DMF (4.00 mL, 0.50 M, 2.0 mmol, 8 eq) to initiate the coupling. The reaction vessel was shaken for 30 min at 50°C (unless stated otherwise)

****Aspartic acid and the adjacent Leucine and Arginine were introduced at with one hour reaction time at room temperature. Fmoc removal was achieved with piperide/DMF in 3 x 5 min at room temperature.(Behrendt and Offer, 2016)

Ac-Lys(N_3)-Val-Thr(*t*Bu)-His(Trt)-Thr(*t*Bu)-Tyr(*t*Bu)-Leu-Glu(O*t*Bu)-Pro-Gly-Pro-Val-Thr(*t*Bu)-Ala-Asn(Trt)-Arg(Pbf)-Gln(Trt)-Leu-Tyr(*t*Bu)-Pro-Glu(O*t*Bu)-Trp(Boc)-Thr(*t*Bu)-Glu(O*t*Bu)-Ala-Gln(Trt)-Arg(Pbf)-Leu-Asp(O*t*Bu)-$_{\alpha}$Abu-Trp(Boc)-Arg(Pbf)-Gly-Lys(Mmt)-Tentagel-S-Rink amide (24).
Peptide synthesis was performed on a 250 µmol scale using the general procedure. Resulting in functionalized **24** used in further reactions.

Ac-Lys(N_3)-Val-Thr-His-Thr-Tyr-Leu-Glu-Pro-Gly-Pro-Val-Thr-Ala-Asn-Arg-Gln-Leu-Tyr-Pro-Glu-Trp-Thr-Glu-Ala-Gln-Arg-Leu-Asp-$_{\alpha}$Abu-Trp-Arg-Gly-Lys(Peg-TLR7L)-NH_2 (26).
Resin **24** (theoretical loading of 100 µmol) was loaded in a syringe with frit and treated with a TFA (1% in DCM) shaken for five minutes followed by filtration. This was repeated until the filtrate lost the orange/yellow color (~ 12x). After which the resin was washed with DCM (5x), DMF (5x) Piperidine (20% in DMF, 1x) and DMF (5x). The lysine was elongated with Fmoc-AEEA-OH using the general protocol followed by introduction of **23** (183 mg, 400 µmol, 4eq) by shaking for one hour with HCTU (165.4 mg, 400 µmol, 4 eq) and DIPEA (140 µL, 800 µmol, 8eq) in DMF (3.6 mL, 0.11 M of **23**). Resulting in resin **25** Ac-Lys(N_3)-Val-Thr(*t*Bu)-His(Trt)-Thr(*t*Bu)-Tyr(*t*Bu)-Leu-Glu(O*t*Bu)-Pro-Gly-Pro-Val-Thr(*t*Bu)-Ala-Asn(Trt)-Arg(Pbf)-Gln(Trt)-Leu-Tyr(*t*Bu)-Pro-Glu(O*t*Bu)-Trp(Boc)-Thr(*t*Bu)-Glu(O*t*Bu)-Ala-Gln(Trt)-Arg(Pbf)-Leu-Asp(O*t*Bu)-$_{\alpha}$Abu-Trp(Boc)-Arg(Pbf)-Gly-Lys(Peg-TLR7L)-Tentagel-S-Rink amide. The resin was washed with DMF (3x), DCM (3x) and Et_2O (2x) followed by nitrogen purge, transferred to a flask and treated for 120 minutes with a cleavage cocktail (25 mL, TFA/TIS/H_2O/phenol, 188/5/5/2, v/v/v/w). The mixture was concentrated to approximately one mL, and the resin was filtered off into

a cold mixture of diethylether/pentane (45 mL, 5/4, v/v). The resin was washed off extra with neat TFA (3x 1 mL) into the ether solution. This solution was centrifuged (10 minutes, 5000 rpm) after which the supernatant was removed and the precipitate was dried under nitrogen. Purification via RP-HPLC (linear gradient 23 - 36 % B in A, 12 min, Gemini-NX 5µm C18, 110 Å, 250 x 10.0 mm, 5 mL/min) yielded azido-peptide **26** as a white powder after lyophilisation. (23.30 mg, 5.09 µmol, 5.09% over 36 couplings, 92% per step). LC-MS: R_t = 7.05 min (10 - 50% ACN; 13 min); R_t = 4.71 min (10 - 90% ACN; 13 min); HRMS $[C_{207}H_{307}N_{61}O_{58} + 5H]^{5+}$: 916.4700 found, 916.46737 calculated.

Val-Thr(*t*Bu)-His(Trt)-Thr(*t*Bu)-Tyr(*t*Bu)-Leu-Glu(O*t*Bu)-Pro-Gly-Pro-Val-Thr(*t*Bu)-Ala-Asn(Trt)-Arg(Pbf)-Gln(Trt)-Leu-Tyr(*t*Bu)-Pro-Glu(O*t*Bu)-Trp(Boc)-Thr(*t*Bu)-Glu(O*t*Bu)-Ala-Gln(Trt)-Arg(Pbf)-Leu-Asp(O*t*Bu)-$_{\alpha}$Abu-Trp(Boc)-Arg(Pbf)-Gly-Lys(Mmt)-Tentagel-S-Rink amide (S11).

Peptide synthesis was performed on a 250 µmol scale using the general procedure. Resulting in functionalized **S11** used in further reactions.

Ac-Val-Thr-His-Thr-Tyr-Leu-Glu-Pro-Gly-Pro-Val-Thr-Ala-Asn-Arg-Gln-Leu-Tyr-Pro-Glu-Trp-Thr-Glu-Ala-Gln-Arg-Leu-Asp-$_{\alpha}$Abu-Trp-Arg-Gly-Lys-NH_2 (gp100).

Resin **S11** (theoretical loading of 25 µmol), was capped using the general protocol resulting in **S12** Ac-Val-Thr(*t*Bu)-His(Trt)-Thr(*t*Bu)-Tyr(*t*Bu)-Leu-Glu(O*t*Bu)-Pro-Gly-Pro-Val-Thr(*t*Bu)-Ala-Asn(Trt)-Arg(Pbf)-Gln(Trt)-Leu-Tyr(*t*Bu)-Pro-Glu(O*t*Bu)-Trp(Boc)-Thr(*t*Bu)-Glu(O*t*Bu)-Ala-Gln(Trt)-Arg(Pbf)-Leu-Asp(O*t*Bu)-$_{\alpha}$Abu-Trp(Boc)-Arg(Pbf)-Gly-Lys(Mmt)-Tentagel-S-Rink amide. The resin was transferred to a flask and treated for 120 minutes with a cleavage cocktail (10 mL, TFA/TIS/H_2O, 190/5/5, v/v/v). The mixture was concentrated to approximately one mL, and the resin was filtered off into a cold mixture of diethylether/pentane (45 mL, 5/4, v/v). The resin was washed off extra with neat TFA (3x 1 mL) into the ether solution. This solution was centrifuged (10 minutes, 5000 rpm) after which the supernatant was removed and the precipitate was dried under nitrogen flow and dissolved (4 mL, DMF/H_2O, 1/3, v/v). Purification via RP-HPLC (linear gradient 19.5 - 29.5 % B in A, 10 min, Gemini-NX 5µm C18, 110 Å, 250 x 10.0 mm, 5 mL/min) yielded title compound **gp100** as a white powder after lyophilization. (8.97 mg, 2.28 µmol, 9.1% over 33 couplings, 93% per step). LC-MS: R_t = 6.01 min (10 - 50% ACN; 13 min); R_t = 4.25 min (10 - 90% ACN; 13 min); HRMS $[C_{178}H_{269}N_{51}O_{51} + 5H]^{5+}$: 788.8100 found, 788.80888 calculated.

Ac-Val-Thr-His-Thr-Tyr-Leu-Glu-Pro-Gly-Pro-Val-Thr-Ala-Asn-Arg-Gln-Leu-Tyr-Pro-Glu-Trp-Thr-Glu-Ala-Gln-Arg-Leu-Asp-$_{\alpha}$Abu-Trp-Arg-Gly-Lys(Peg-TLR7L)-NH_2 (gp100-TLR7L).

Resin **S12** was synthesized on 100 µmol scale using single couplings at room temperature only. The resin was transferred to a syringe with frit and treated with a TFA (1% in DCM) shaken for five minutes followed by filtration. This was repeated until the filtrate lost the orange/yellow color (~ 12x). After which the resin was washed with DCM (5x), DMF (5x) Piperidine (20% in DMF, 1x) and DMF (5x). The lysine was elongated with Fmoc-AEEA-OH using the general protocol followed by introduction of **23** (91.5 mg, 200 µmol, 2eq) by shaking for two hour with HCTU (82.7 mg, 200 µmol, 2eq) and DIPEA (69.7 µL, 400 µmol, 4eq) in DMF (1.8 mL, 0.11 M of **23**). The resin was washed with DMF (3x), DCM (3x) and Et_2O (2x) followed by nitrogen purge, transferred to a flask and treated for 120 minutes with a cleavage cocktail (10 mL, TFA/TIS/H_2O, 190/5/5, v/v/v). The mixture was concentrated to approximately one mL, and the resin was filtered off into a cold mixture of diethylether/pentane (45 mL, 5/4, v/v). The resin was washed off extra with neat TFA (3x 1 mL) into the ether solution. This solution was centrifuged (10 minutes, 5000 rpm) after which the supernatant was removed and the precipitate was dried under nitrogen flow. Purification via RP-HPLC (linear gradient 24 - 36 % B in A, 12 min, Gemini-NX 5µm C18, 110 Å, 250 x 10.0 mm, 5 mL/min) yielded title compound **gp100-TLR7L** as a white powder after lyophilization. (7.85 mg, 1.78 µmol, 1.78% over 35 couplings, 89% per step). LC-MS: R_t = 4.94 min (10 - 90% ACN; 13 min); HRMS $[C_{201}H_{297}N_{57}O_{57} + 5H]^{5+}$: 885.6479 found, 885.65029 calculated.

Ac-Val-Thr-His-Thr-Tyr-Leu-Glu-Pro-Gly-Pro-Val-Thr-Ala-Asn-Arg-Gln-Leu-Tyr-Pro-Glu-Trp-Thr-Glu-Ala-Gln-Arg-Leu-Asp-Cys-Trp--Gly-Lys(Peg-TLR7L)-NH_2 (gp100(Cys)-TLR7L).

Ac-Val-Thr(*t*Bu)-His(Trt)-Thr(*t*Bu)-Tyr(*t*Bu)-Leu-Glu(O*t*Bu)-Pro-Gly-Pro-Val-Thr(*t*Bu)-Ala-Asn(Trt)-Arg(Pbf)-Gln(Trt)-Leu-Tyr(*t*Bu)-Pro-Glu(O*t*Bu)-Trp(Boc)-Thr(*t*Bu)-Glu(O*t*Bu)-Ala-Gln(Trt)-Arg(Pbf)-Leu-Asp(O*t*Bu)-Cys(Trt)-Trp(Boc)-Arg(Pbf)-Gly-Lys(Mmt)-Tentagel-S-Rink amide **S15** was synthesized on a 100 µmol scale using single couplings at room temperature only. The resin was loaded in a syringe with frit and treated with a TFA (1% in DCM) shaken for five minutes followed by filtration. This was repeated until the filtrate lost the orange/yellow color (12x). After which the resin was washed with DCM (5x), DMF (5x) Piperidine (20% in DMF, 1x) and DMF (5x). The lysine was elongated with Fmoc-AEEA-OH using the general protocol followed by introduction of **23** (91.5 mg, 200 µmol, 2 eq) by shaking for two hour with HCTU (82.7 mg, 200 µmol, 2 eq) and DIPEA (69.7 µL, 400 µmol, 4eq) in DMF (1.8 mL, 0.11 M of **23**). The resin was washed with DMF (3x), DCM (3x) and Et_2O (2x) followed by nitrogen purge, transferred to a flask and treated for 120 minutes with a cleavage cocktail (10 mL, TFA/TIS/H_2O, 190/5/5, v/v/v). The mixture was concentrated to approximately one mL, and the resin was filtered off into a cold mixture of diethylether/pentane (45 mL, 5/4, v/v). The resin was washed off extra with neat TFA (3x 1 mL) into the ether solution. This solution was centrifuged (10 minutes, 5000 rpm) after which the supernatant was removed and the precipitate was dried under nitrogen flow. Purification via RP-HPLC (linear gradient 24 - 36 % B in A, 12 min, Gemini-NX 5µm C18, 110 Å, 250 x 10.0 mm, 5 mL/min) yielded title compound **gp100(Cys)-TLR7L** as a white powder after lyophilization. (1.03 mg, 0.232 µmol, 0.23% over 35 couplings, 84% per step). LC-MS: R_t = 5.01 min (10 - 90% ACN; 13 min); HRMS $[C_{201}H_{296}N_{56}O_{57}S + 5H]^{5+}$: 889.0417 found, 889.04249 calculated.

General procedure towards final trifunctional conjugates

The “general click protocol” used for the final conjugation: All solvents used in these reactions were degassed by sonicating while bubbling argon through the solutions. A solution of azidopeptide **26** in DMSO was mixed with a solution of alkyne functionalized glycoclusters in water **(19,20,21** or **22)** followed by addition of an aliquot of a stock solution of CuI (0.1 eq), THPTA (0.3 eq) and DIPEA (0.2 eq) in water ([Cu^+] = 0.5 M). The reaction was stirred at 45°C and the process was followed via LC-MS. When reactions do not progress and turn blue, a stock solution of sodium ascorbate (0.25 M) and arginine(Conibear et al., 2016) (0.5 M) (0.2 - 1 eq ascorbate) in water was added. After completion a small amount of Quadrasil® AP (washed with water) was added, stirred for 1 h, filtered and applied on gel filtration (Toyopearl HW40S, 150 mM NH_4HCO_3 aq., buffer contained 20% ACN, 1.6x60 cm, 1 mL/min) and/or purified via RP-HPLC (linear gradient, 5 mL/min, Gemini-NX 5μm C18, 110 Å, 250 x 10.0 mm) followed by lyophilization.

Ac-Lys(*[Ac-Lys(1,2-Man$_2$)-Lys(1,2-Man$_2$)-Lys(1,2-Man$_2$)-Lys(1,2-Man$_2$)-Lys(1,2-Man$_2$)-Lys(1,2-Man$_2$)-Gly-Lys(*triazolylpentyl-5-amide)-NH$_2$])-Val-Thr-His-Thr-Tyr-Leu-Glu-Pro-Gly-Pro-Val-Thr-Ala-Asn-Arg-Gln-Leu-Tyr-Pro-Glu-Trp-Thr-Glu-Ala-Gln-Arg-Leu-Asp-$_\alpha$Abu-Trp-Arg-Gly-Lys(Peg-TLR7L)-NH$_2$ (B6-gp100-TLR7L).
A solution of mannosides cluster **19** (71.5 μL, 0.01 M, 715 nmol, 1 eq, aq.) and azido-peptide **26** in DMSO (75 μL, 0.01 M, 750 μmol, 1.05 eq) were conjugated using the general click protocol. After purification by RP-HPLC (8 - 42 % B, 10 min) compound **B6-gp100-TLR7L** was obtained as a white powder (1.395 mg, 172 nmol, 24%). LC-MS: R_t = 7.10 min (0 - 50% ACN; 13 min); HRMS [$C_{348}H_{535}N_{89}O_{134}$ + 6H]$^{6+}$: 1352.47638 found, 1352.47226 calculated.

Ac-Lys(*[Ac-Lys(1,3-Man$_2$)-Lys(1,3-Man$_2$)-Lys(1,3-Man$_2$)-Lys(1,3-Man$_2$)-Lys(1,3-Man$_2$)-Lys(1,3-Man$_2$)-Gly-Lys(*triazolylpentyl-5-amide)-NH$_2$])-Val-Thr-His-Thr-Tyr-Leu-Glu-Pro-Gly-Pro-Val-Thr-Ala-Asn-Arg-Gln-Leu-Tyr-Pro-Glu-Trp-Thr-Glu-Ala-Gln-Arg-Leu-Asp-$_\alpha$Abu-Trp-Arg-Gly-Lys(PEG-TLR7L)-NH$_2$ (C6-gp100-TLR7L).
A solution of mannosides cluster **20** (38 μL, 0.005 M, 190 nmol, 1 eq, aq.) and azido-peptide **26** in DMSO (28.5 μL, 0.01 M, 285 μmol, 1.5 eq) were conjugated using the general click protocol. After purification by RP-HPLC (8 - 42% B, 10 min) compound **C6-gp100-TLR7L** was obtained as a white powder (0.630 mg, 78 nmol, 41%). LC-MS: R_t = 7.12 min (10 - 50% ACN; 13 min); HRMS [$C_{348}H_{535}N_{89}O_{134}$ + 6H]$^{6+}$: 1352.47991 found, 1352.47226 calculated.

Ac-Lys(*[Ac-Lys(1,6-Man$_2$)-Lys(1,6-Man$_2$)-Lys(1,6-Man$_2$)-Lys(1,6-Man$_2$)-Lys(1,6-Man$_2$)-Lys(1,6-Man$_2$)-Gly-Lys(*triazolylpentyl-5-amide)-NH$_2$])-Val-Thr-His-Thr-Tyr-Leu-Glu-Pro-Gly-Pro-Val-Thr-Ala-Asn-Arg-Gln-Leu-Tyr-Pro-Glu-Trp-Thr-Glu-Ala-Gln-Arg-Leu-Asp-$_\alpha$Abu-Trp-Arg-Gly-Lys(Peg-TLR7L)-NH$_2$ (D6-GP100-TLR).
A solution of mannosides cluster **21** (35 μL, 0.01 M, 350 nmol, 1 eq, aq.) and azido-peptide **26** in DMSO (40 μL, 0.01 M, 400 nmol, 1.15 eq) were conjugated using the general click protocol. After purification by gel-filtration (eluted at 37.5 - 49.5 mL) compound **D6-gp100-TLR7L** was obtained as a white powder (0.520 mg, 64 nmol, 18 %). LC-MS: R_t = 4.68 min (10 - 90% ACN; 13 min); HRMS [$C_{348}H_{535}N_{89}O_{134}$ + 5H]$^{5+}$: 1622.56900 found, 1622.56470 calculated.

Ac-Lys(*[Ac-Lys(Man$_3$)-Lys(Man$_3$)-Lys(Man$_3$)-Lys(Man$_3$)-Lys(Man$_3$)-Lys(Man$_3$)-Gly-Lys(*triazolylpentyl-5-amide)-NH$_2$])-Val-Thr-His-Thr-Tyr-Leu-Glu-Pro-Gly-Pro-Val-Thr-Ala-Asn-Arg-Gln-Leu-Tyr-Pro-Glu-Trp-Thr-Glu-Ala-Gln-Arg-Leu-Asp-$_\alpha$Abu-Trp-Arg-Gly-Lys(Peg-TLR7L)--NH$_2$ (E6-GP$_{100}$-TLR).
A solution of mannosides cluster **22** (200 μL, 0.01 M, 2.0 μmol, 2 eq, aq.) and azido-peptide **26** in DMSO (100 μL, 0.01 M, 1.0 μmol, 1 eq) were conjugated using the general click protocol. After purification by RP-HPLC (8 - 42% B, 10 min) compound **E6-gp100-TLR7L** was obtained as a white powder (2.483 mg, 273 nmol, 27%). LC-MS: R_t = 7.07 min (10 - 50% ACN; 13 min); ^1H NMR (500 MHz, D_2O) δ 8.55 (s, 1H, H_{arom} (H)), 7.98 (s, 7H, trzl), 7.74 - 6.60 (m, 23H, H_{arom}), 5.03 (s, 6H, H-1), 4.88 (s, 6H, H-1′), 4.85 (s, 6H, H-1′′), 4.77 - 1.40 (m, nd), 1.38 (s, 3H, Ac), 1.36 (s, 3H, Ac), 1.35 - 0.66 (m, nd); HRMS [$C_{384}H_{595}N_{89}O_{164}$ + 6H]$^{6+}$: 1514.69741 found, 1514.69225 calculated.

References

Behrendt, R., and Offer, J. (2016). Advances in Fmoc solid-phase peptide synthesis. *J. Pept.*, 4–27. doi:10.1002/psc.2836.

Chan, W. C., and White, P. D. (2000). *Fmoc solid phase peptide synthesis : a practical approach*. Oxford University Press.

Conibear, A. C., Farbiarz, K., Mayer, R. L., Matveenko, M., Kählig, H., and Becker, C. F. W. (2016). Arginine side-chain modification that occurs during copper-catalysed azide–alkyne click reactions resembles an advanced glycation end product. *Org. Biomol. Chem.* 14, 6205–6211. doi:10.1039/C6OB00932H.

Daly, R., Vaz, G., Davies, A. M., Senge, M. O., and Scanlan, E. M. (2012). Synthesis and Biological Evaluation of a Library of Glycoporphyrin Compounds. *Chem. - A Eur. J.* 18, 14671–14679. doi:10.1002/chem.201202064.

Gential, G. P. P., Hogervorst, T. P., Tondini, E., van de Graaff, M. J., Overkleeft, H. S., Codée, J. D. C., et al. (2019). Peptides conjugated to 2-alkoxy-8-oxo-adenine as potential synthetic vaccines triggering TLR7. *Bioorg. Med. Chem. Lett.*, tbd. doi:10.1016/j.bmcl.2019.03.048.

Horatscheck, A., Wagner, S., Ortwein, J., Kim, B. G., Lisurek, M., Beligny, S., et al. (2012). Benzoylphosphonate-Based Photoactive Phosphopeptide Mimetics for Modulation of Protein Tyrosine Phosphatases and Highly Specific Labeling of SH2 Domains. *Angew. Chemie Int. Ed.* 51, 9441–9447. doi:10.1002/anie.201201475.

Mereyala, H. B., and Gurrala, S. R. (1998). Design, Development and Utility of Glycosyl Donors Bearing an Acetoxymethoxy Leaving Group. *Chem. Lett.* 27, 863–864. doi:10.1246/cl.1998.863.

Thomas, M., Gesson, J.-P., and Papot, S. (2007). First O -Glycosylation of Hydroxamic Acids. *J. Org. Chem.* 72, 4262–4264. doi:10.1021/jo0701839.

Wong, C. S., Hoogendoorn, S., van der Marel, G. A., Overkleeft, H. S., and Codée, J. D. C. (2015). Targeted Delivery of Fluorescent High-Mannose-Type Oligosaccharide Cathepsin Inhibitor Conjugates. *Chempluschem* 80, 928–937. doi:10.1002/cplu.201500004.

Spectral data of Alkynes:

1

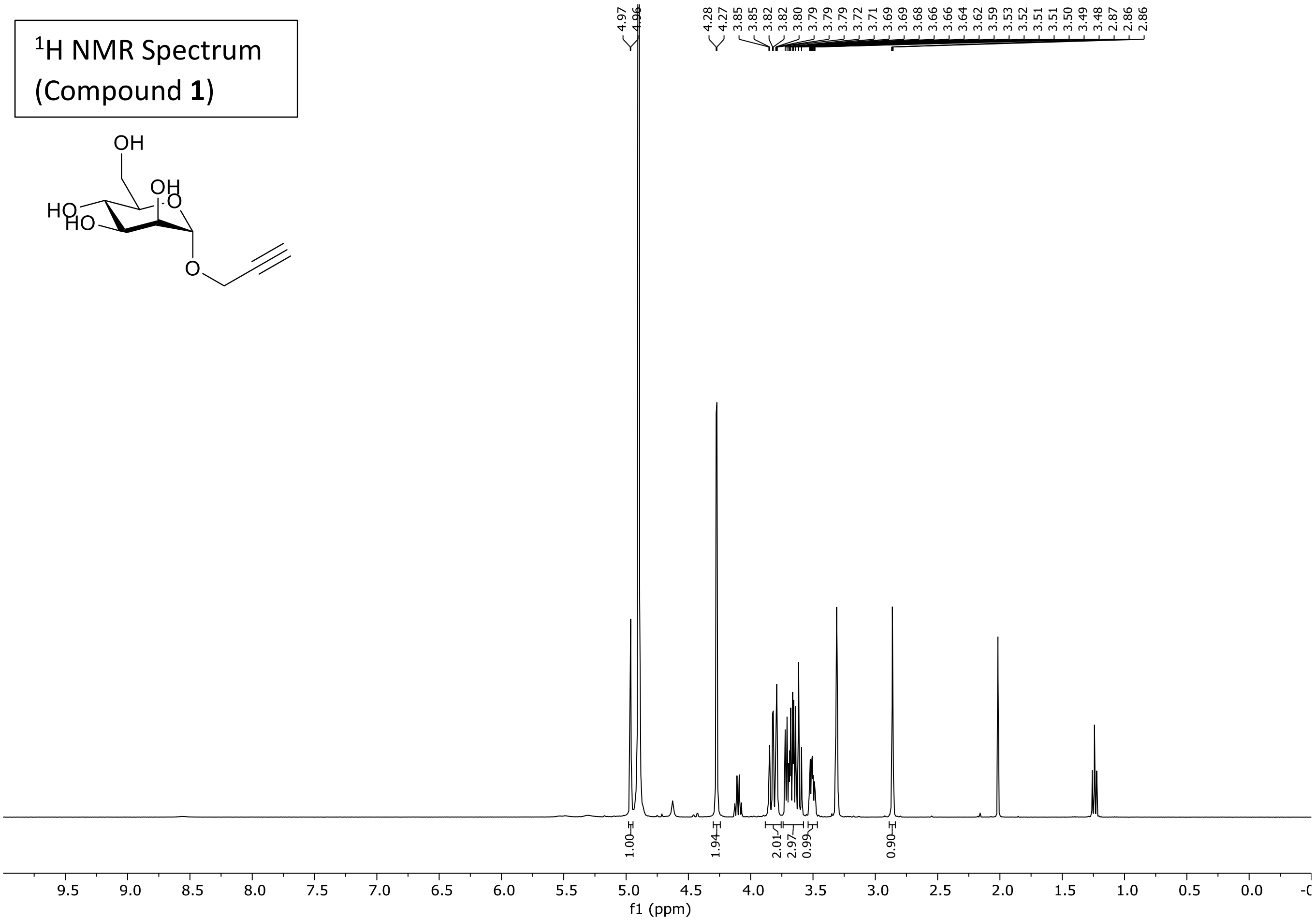

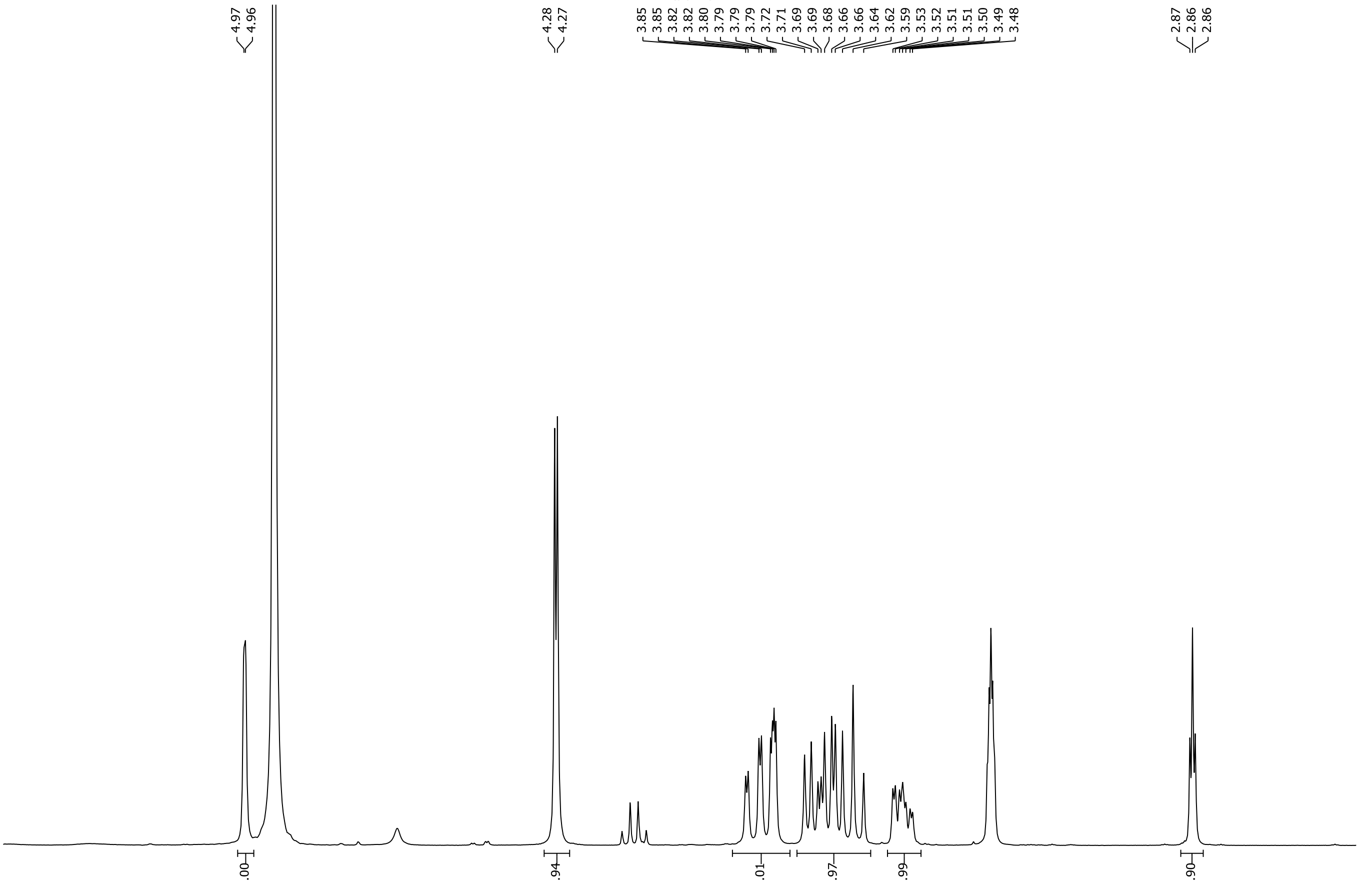

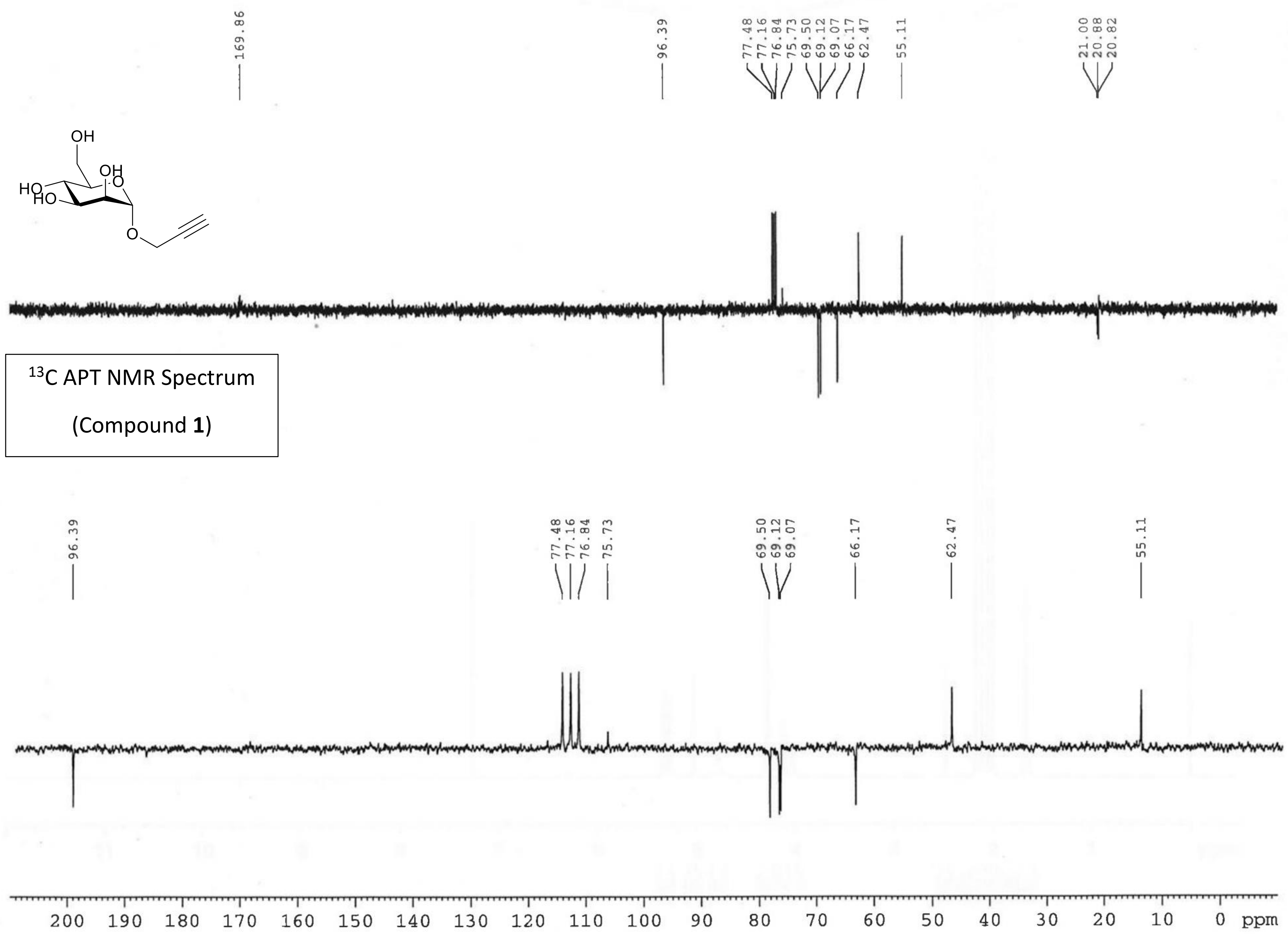

^{13}C APT NMR Spectrum

(Compound **1**)

2

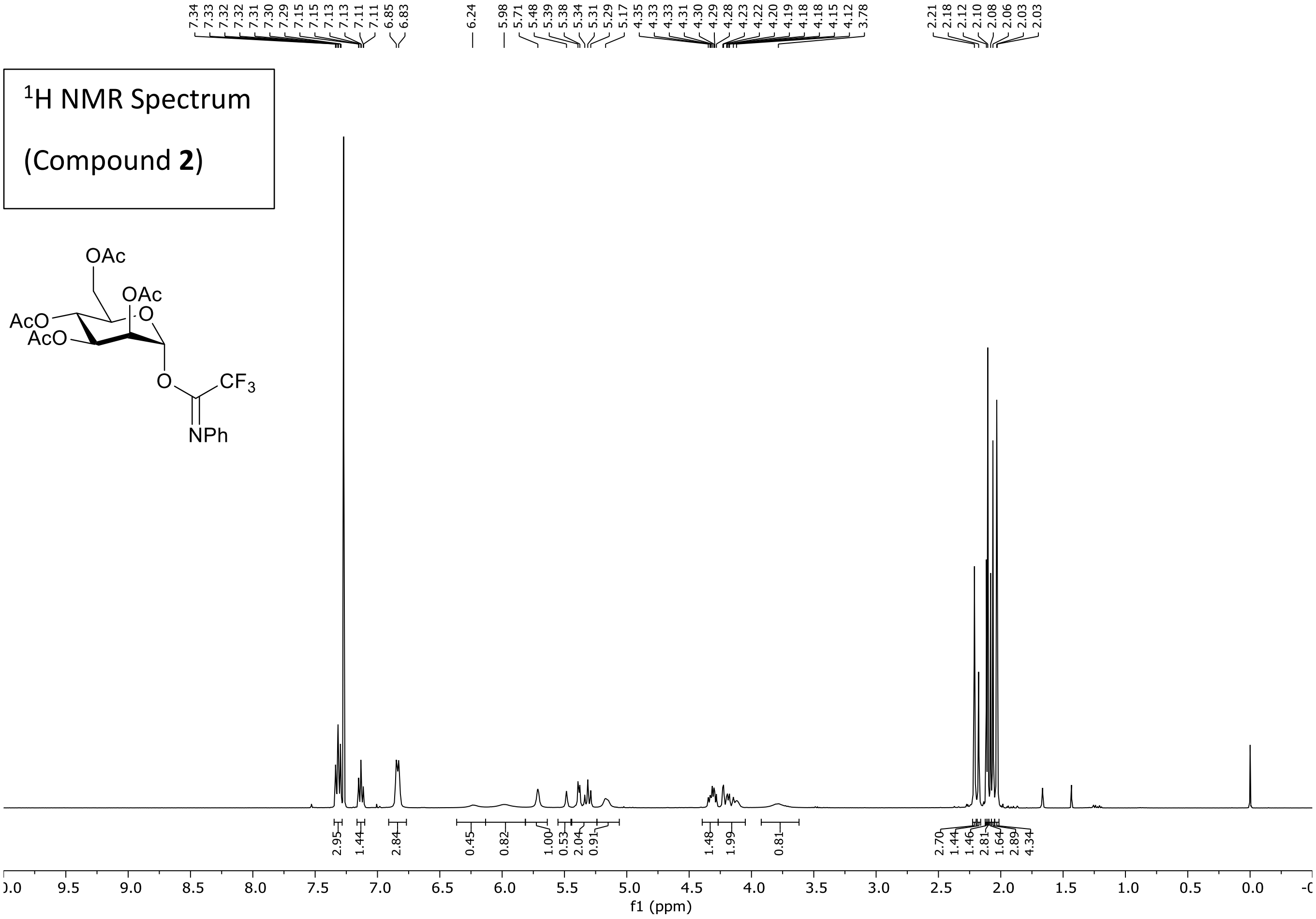

^{1}H NMR Spectrum

(Compound **2**)

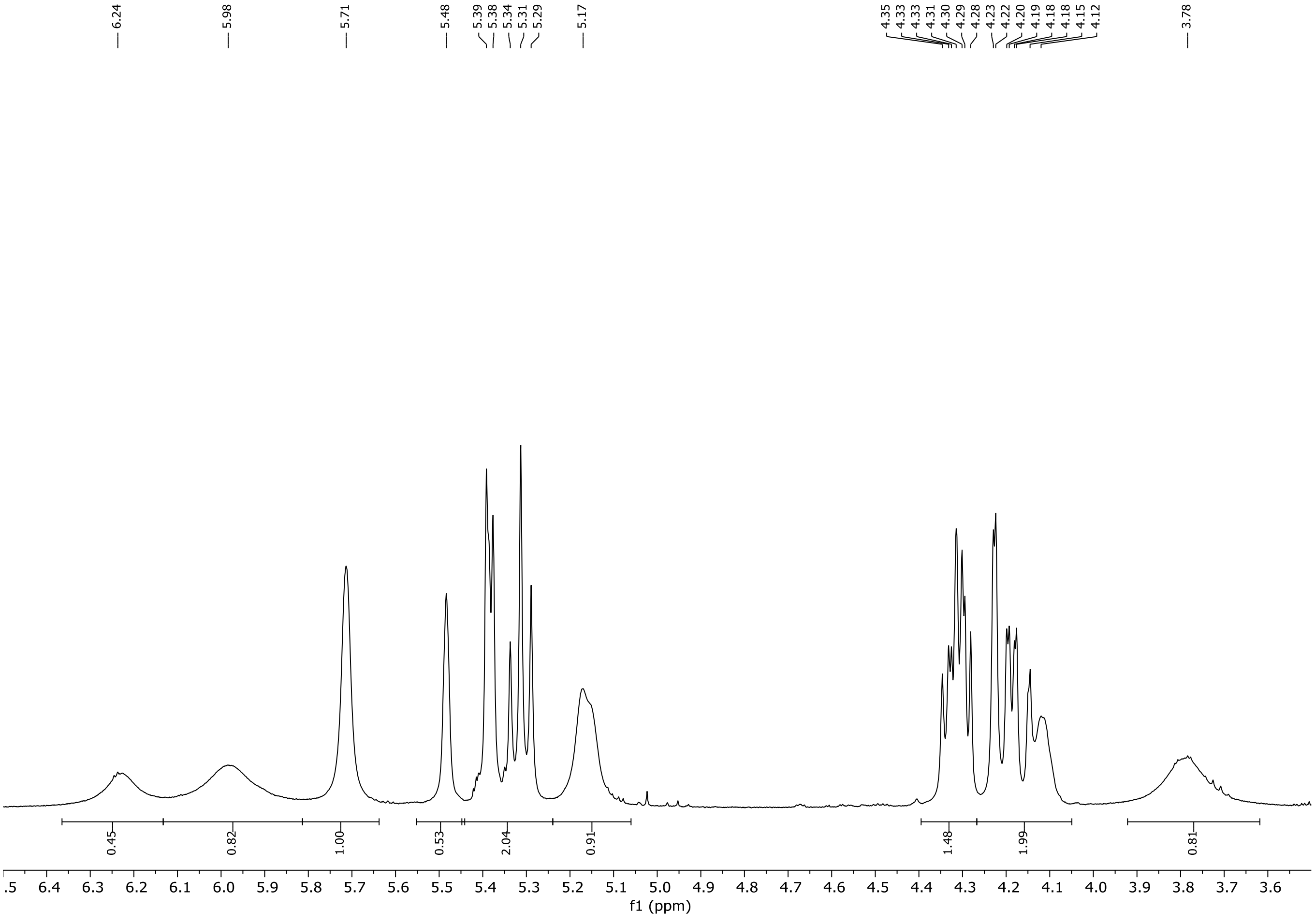

^{13}C APT NMR Spectrum

(Compound **2**)

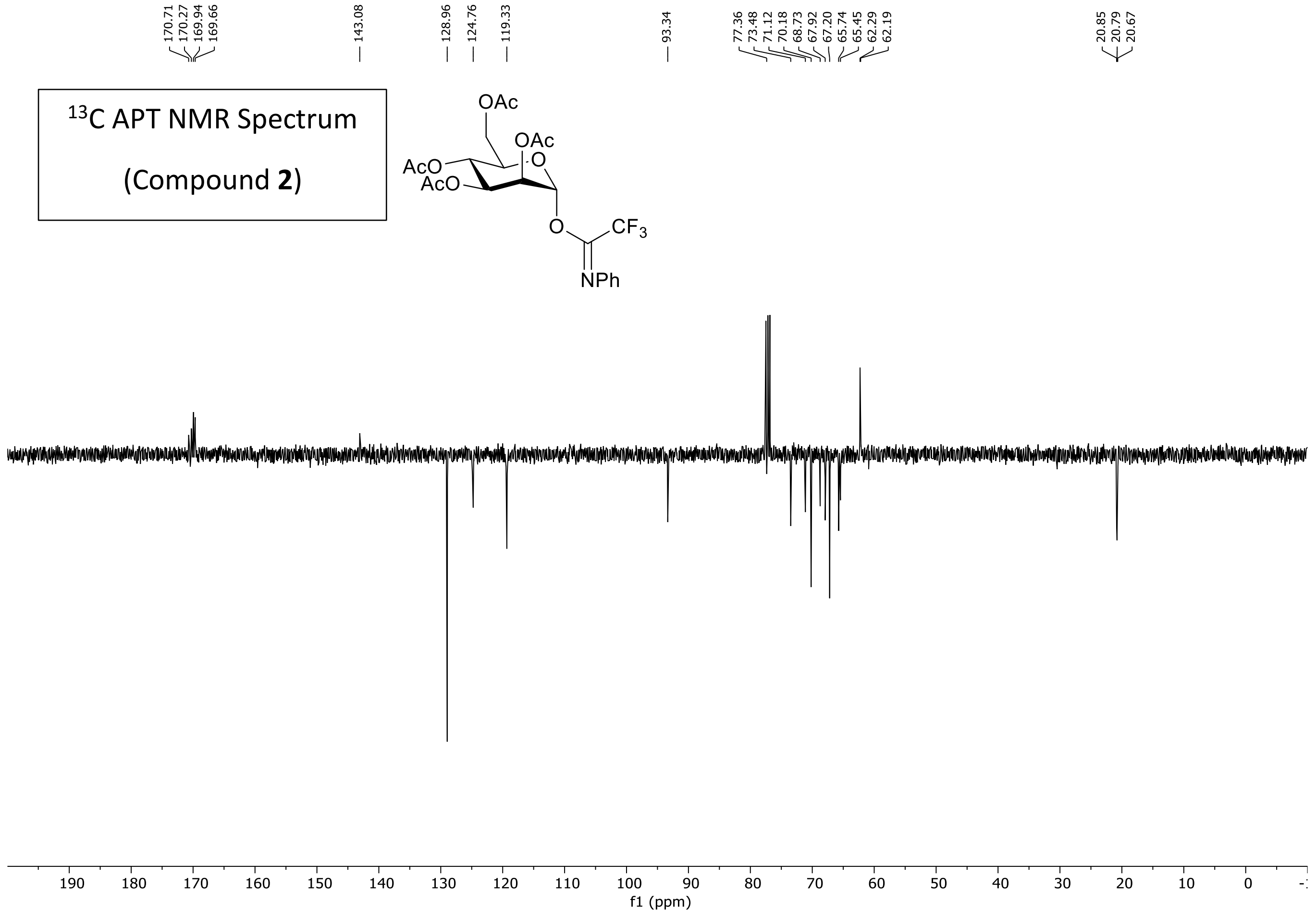

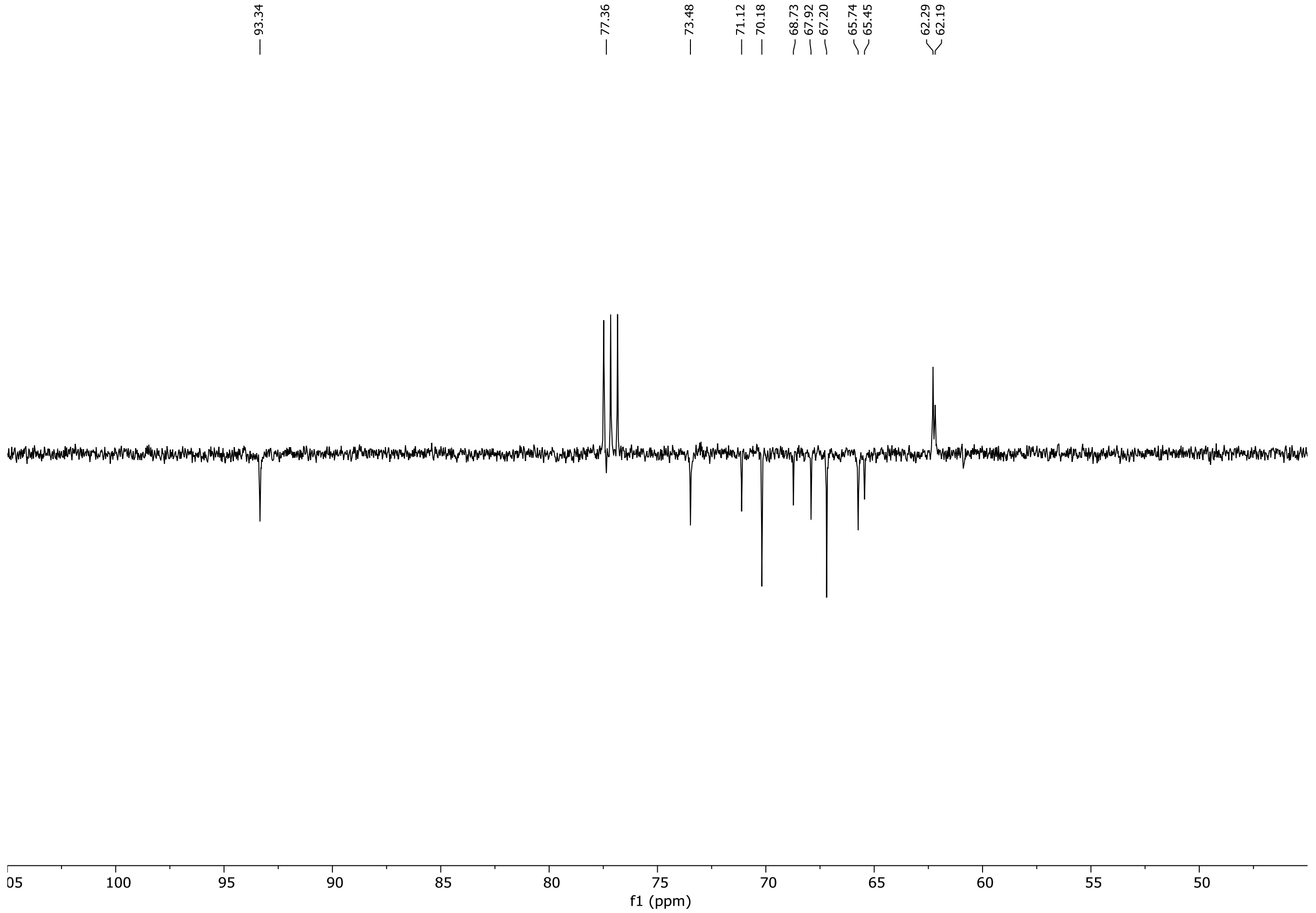

S1

^{1}H NMR Spectrum (Compound **S1**)

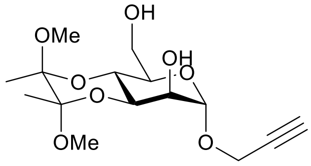

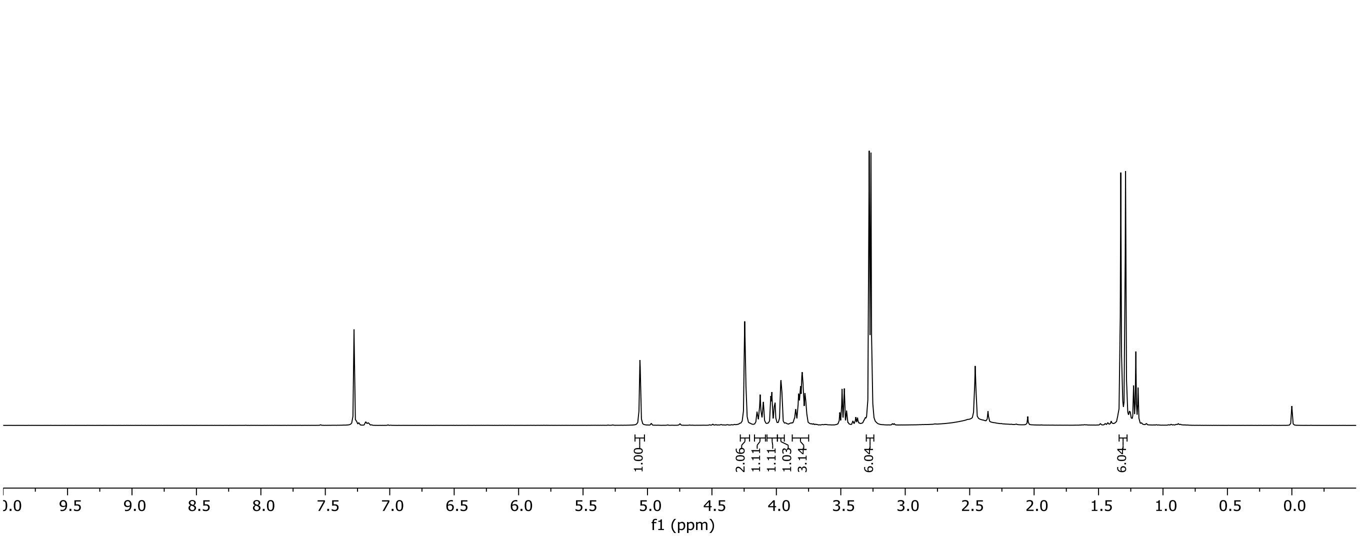

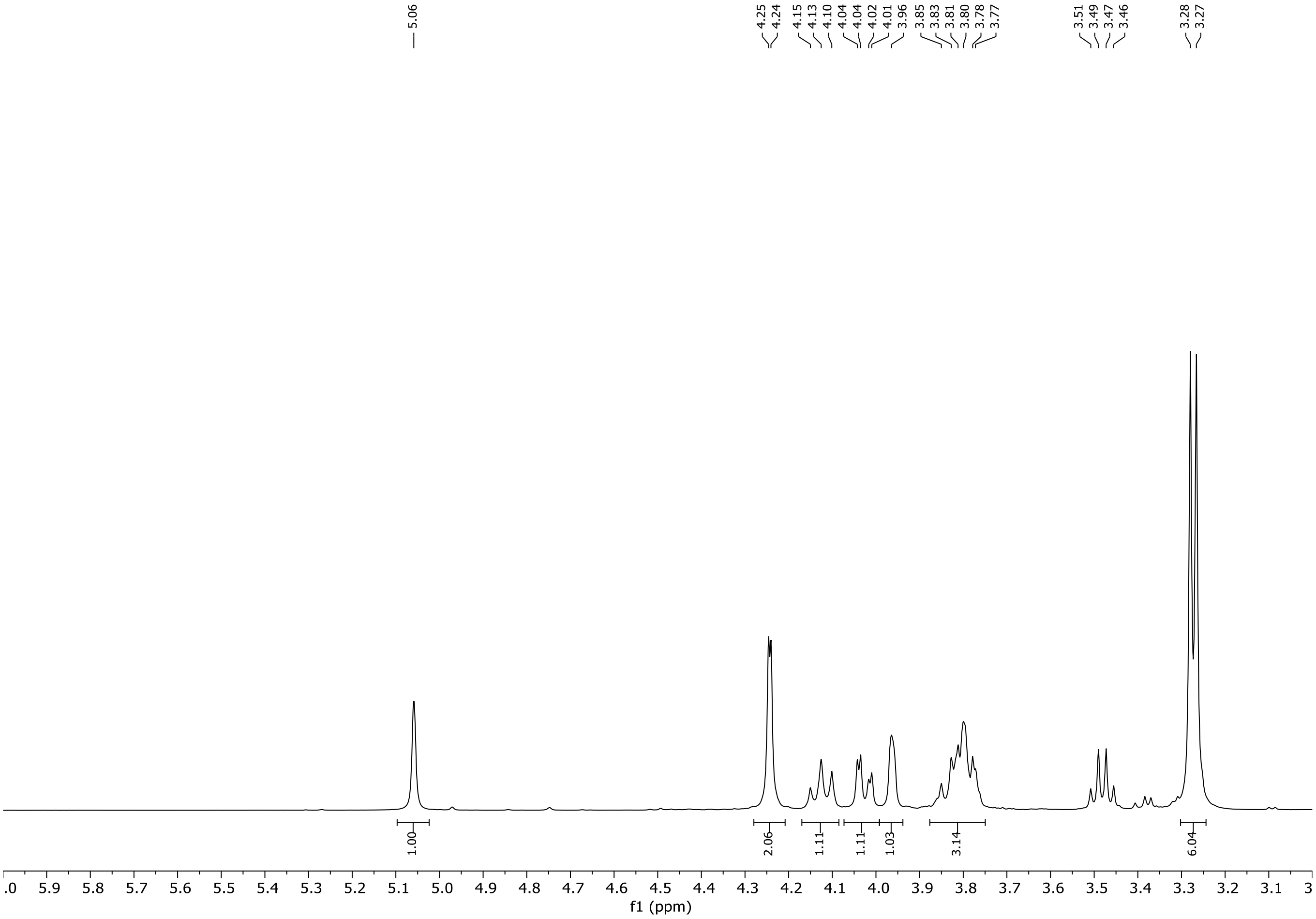

^{13}C APT NMR Spectrum

(Compound **S1**)

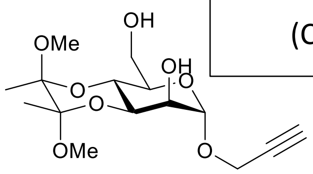

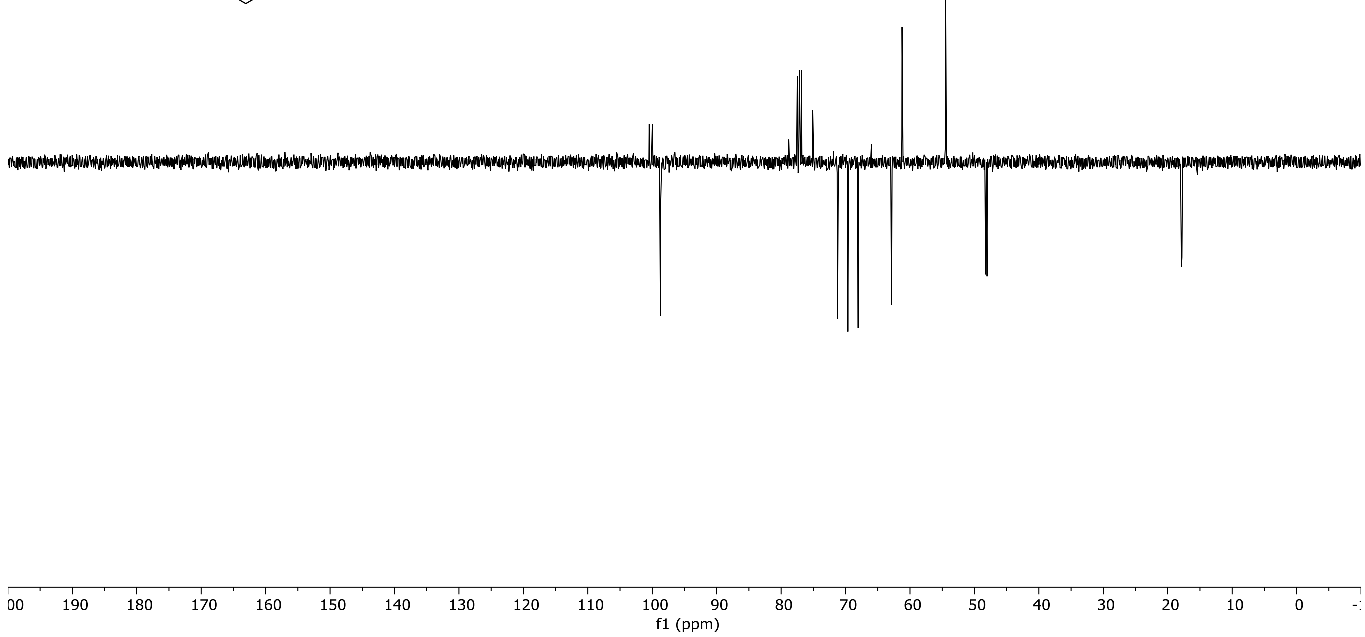

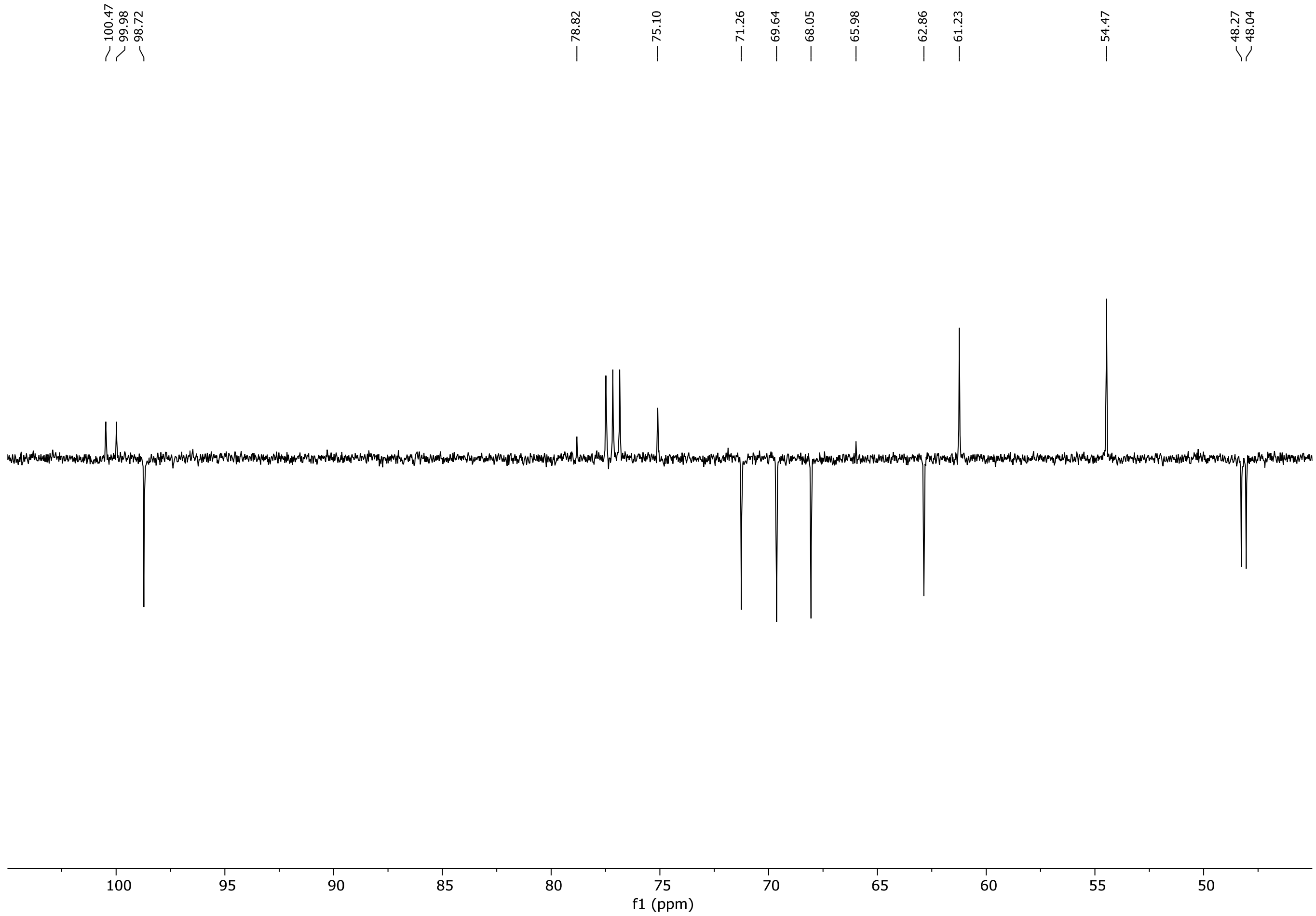

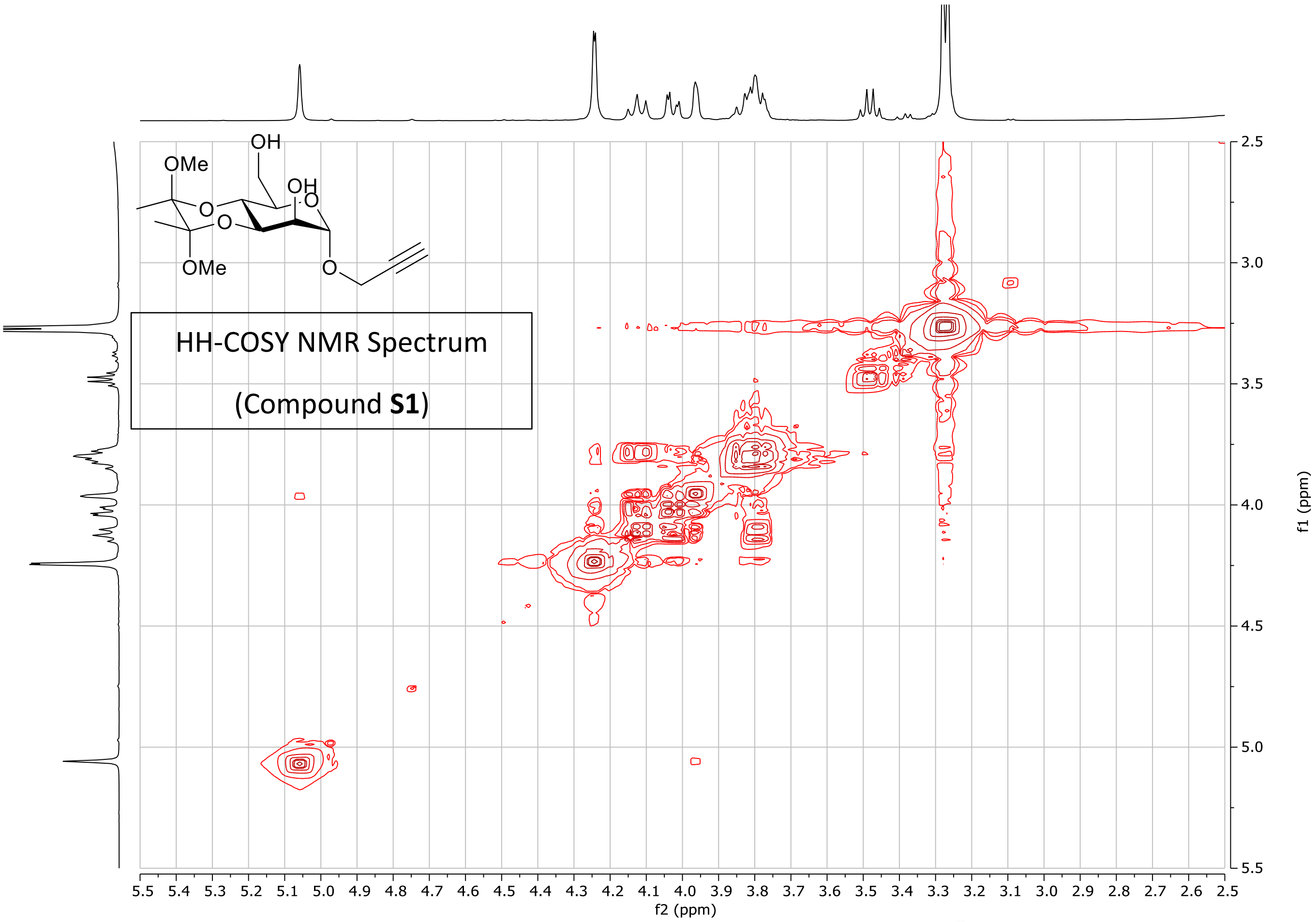
HH-COSY NMR Spectrum
(Compound **S1**)
OH
OMe
OH
O
O
O
OMe
O
f2 (ppm)
f1 (ppm)

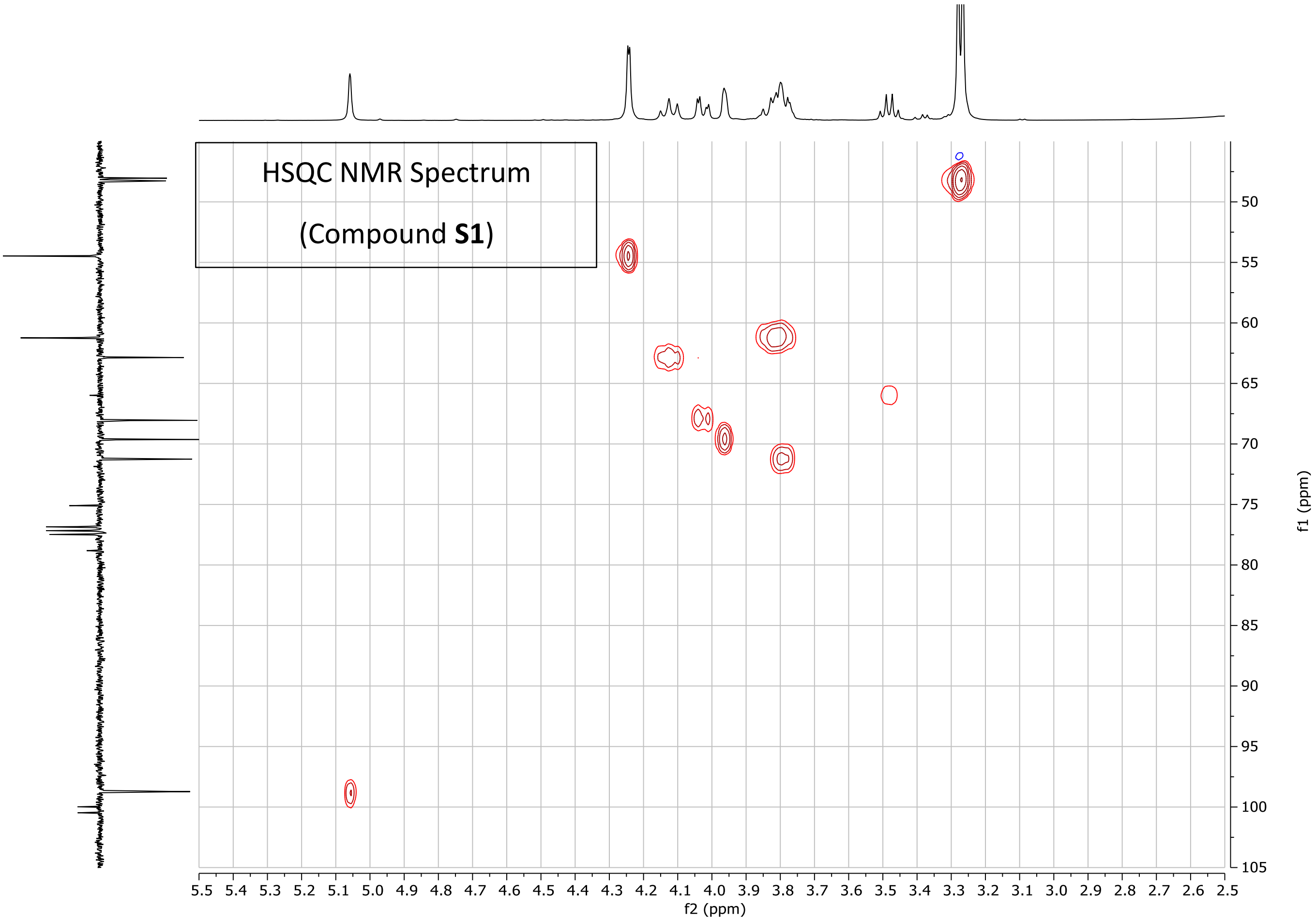
HSQC NMR Spectrum
(Compound **S1**)
f2 (ppm)
f1 (ppm)

3

^{1}H NMR Spectrum

(Compound **3**)

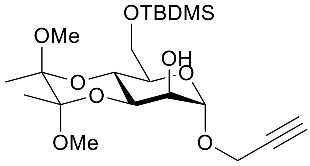

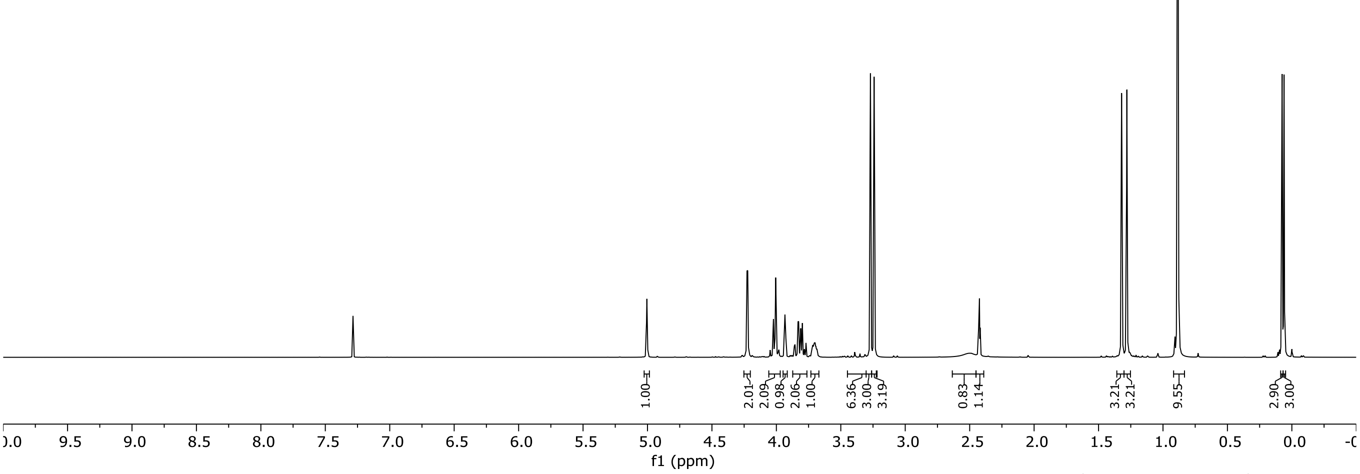

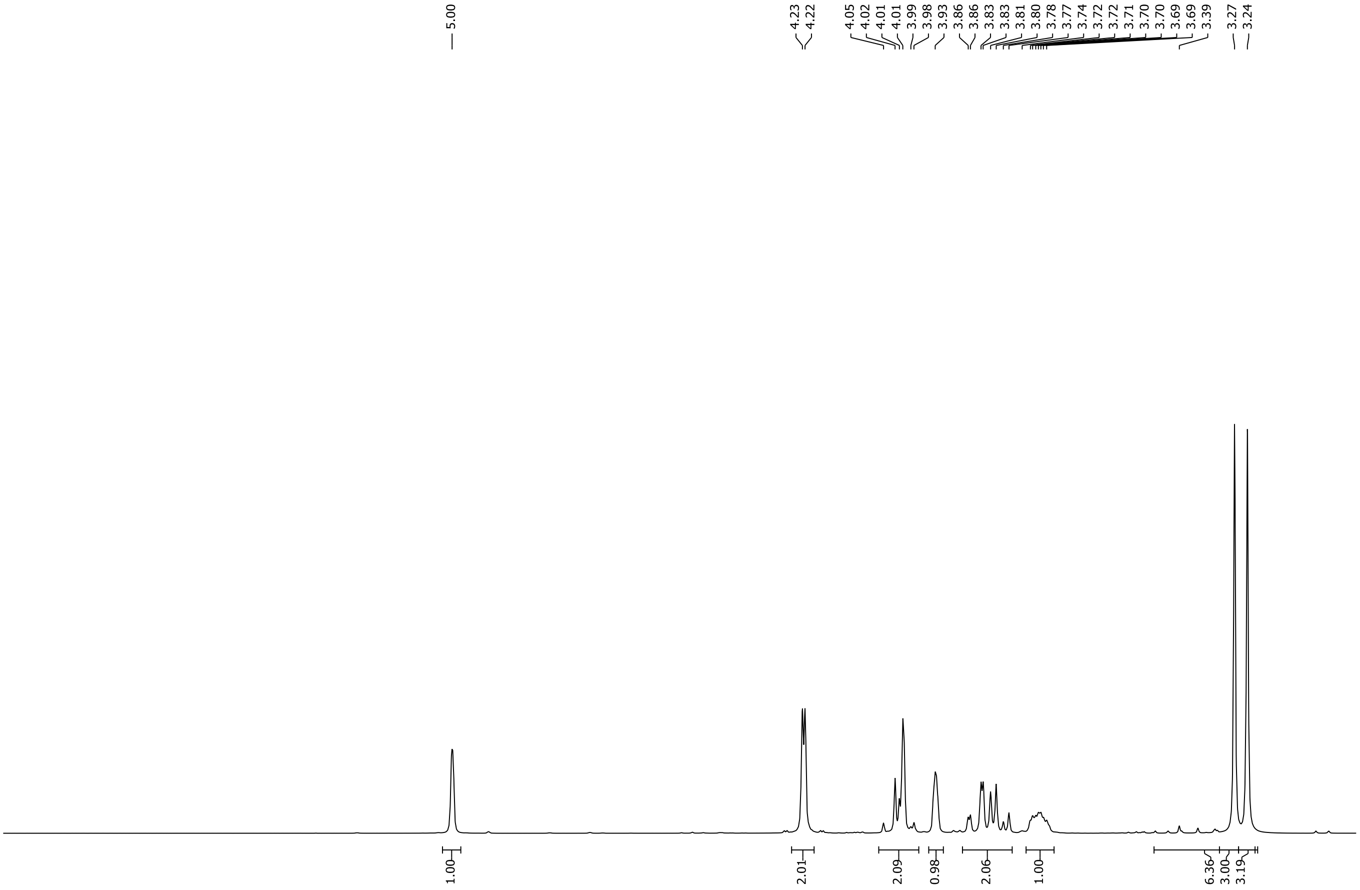

^{13}C APT NMR Spectrum

(Compound **3**)

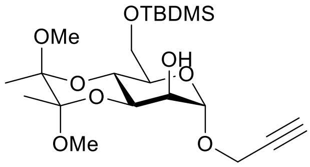

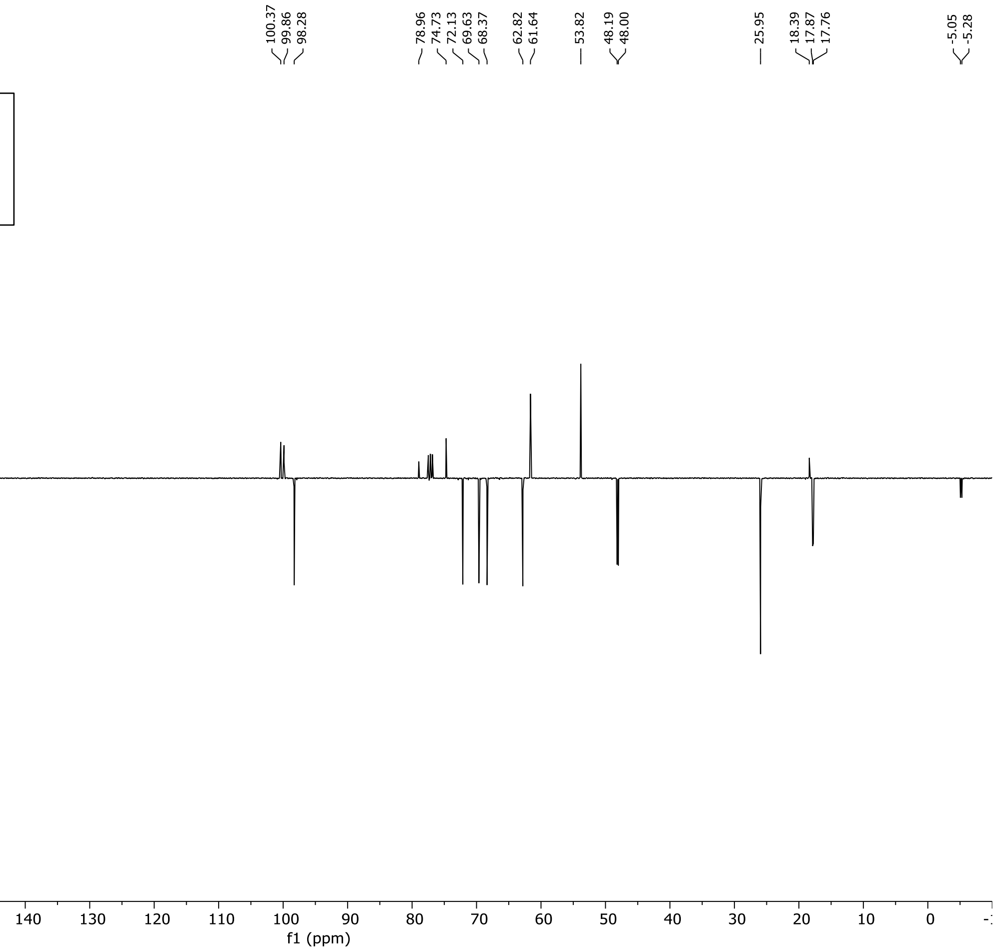

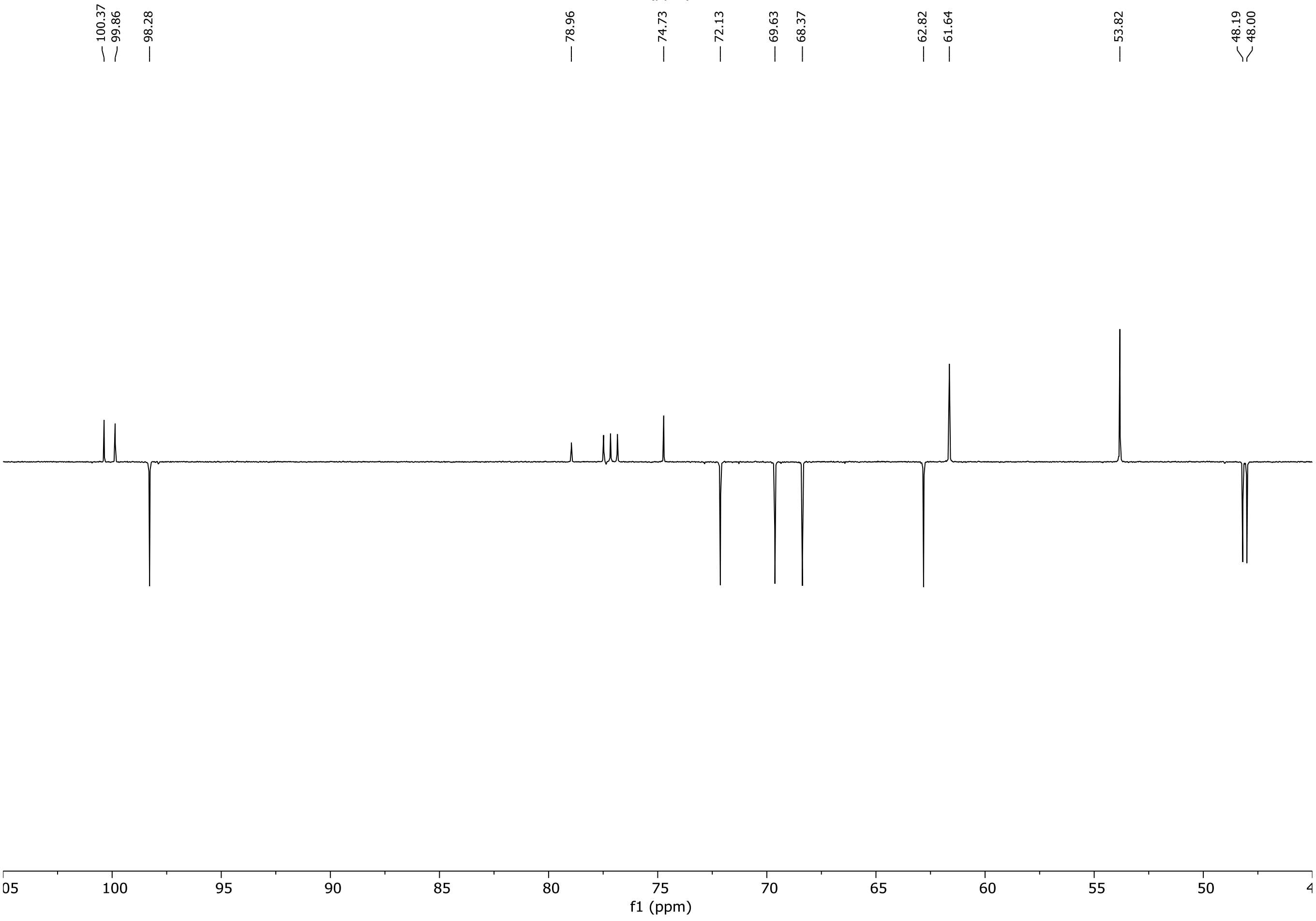

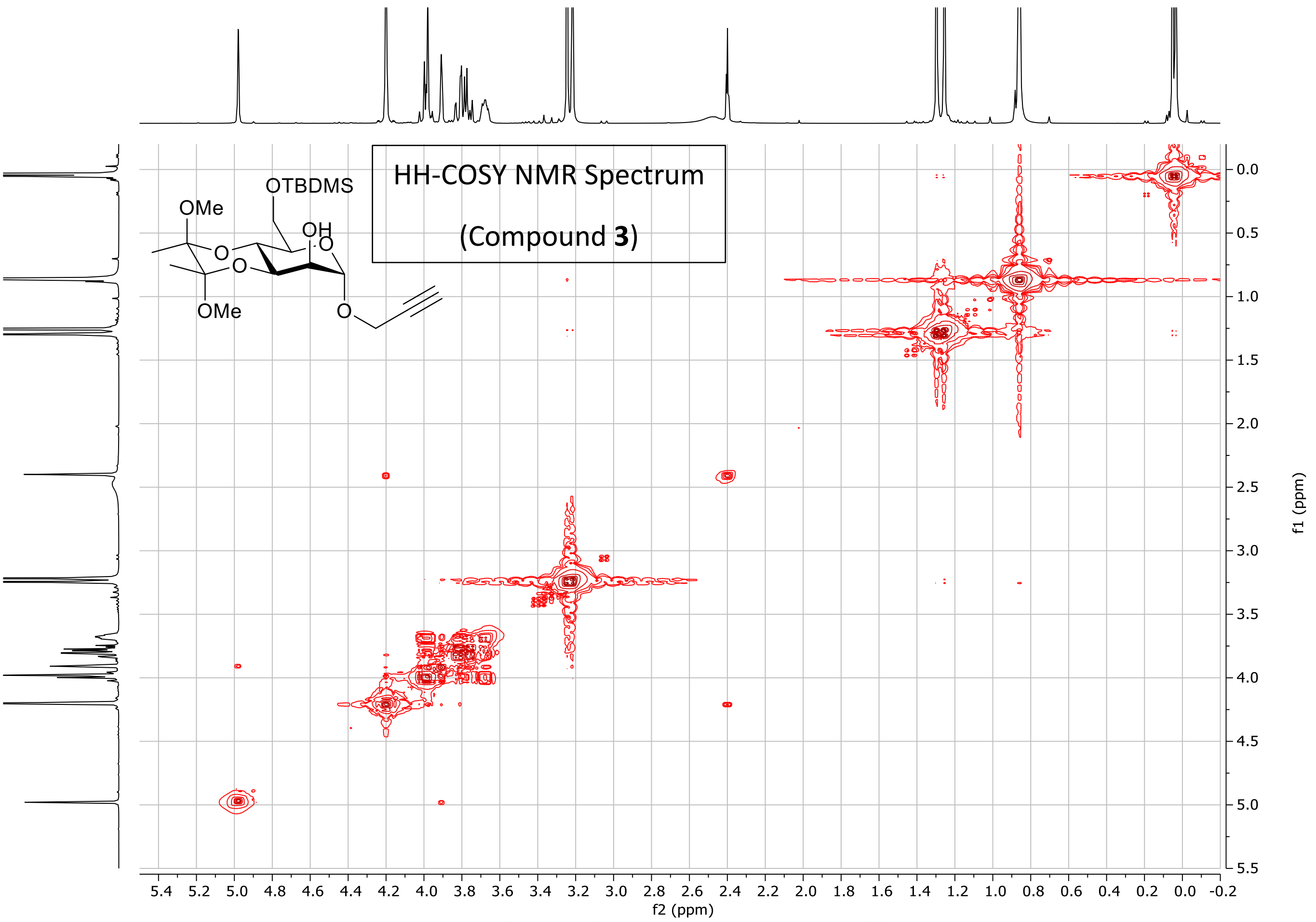

HH-COSY NMR Spectrum
(Compound **3**)
OTBDMS
OMe
OH
O
O
O
OMe
O
f2 (ppm)
f1 (ppm)

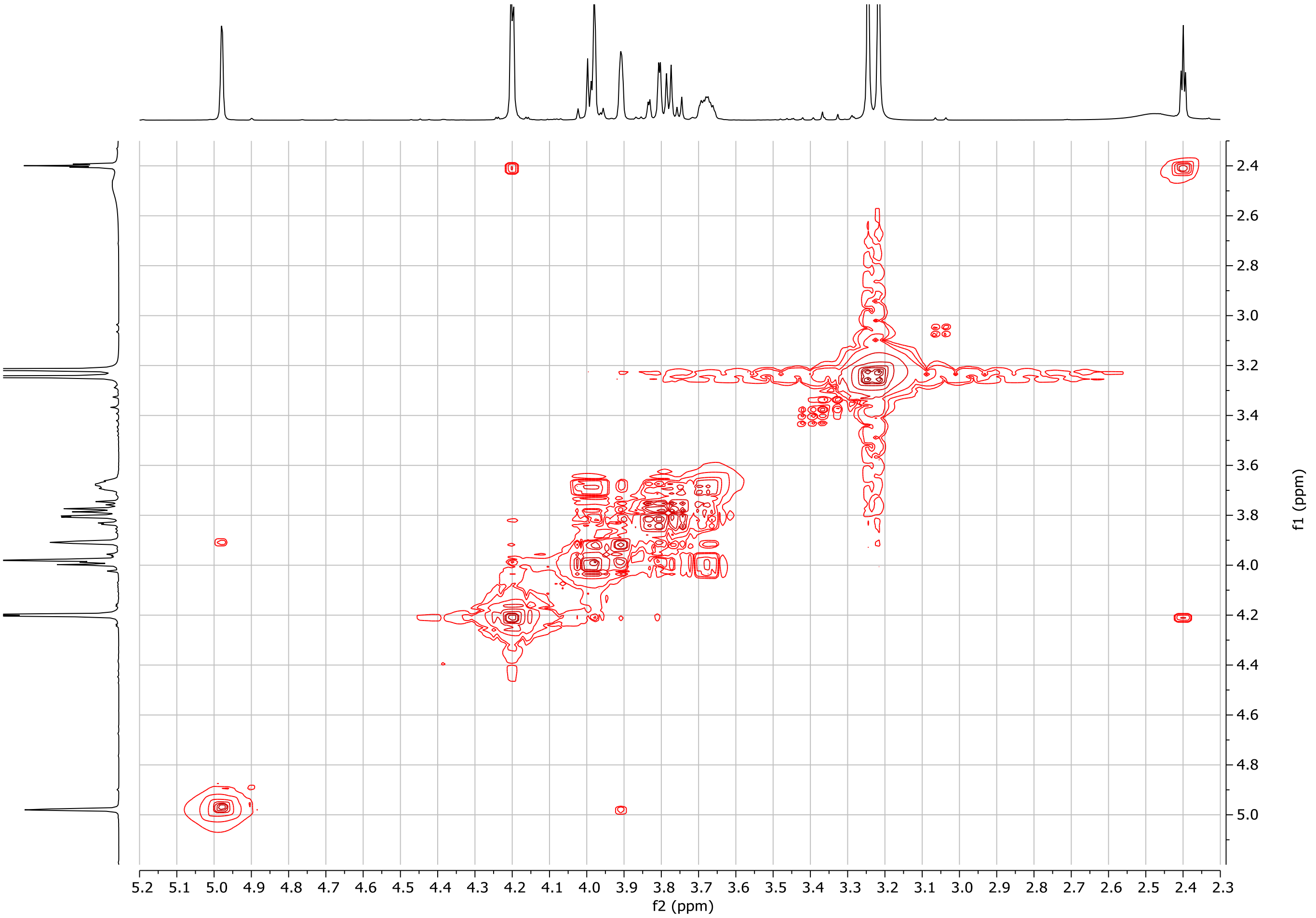

f2 (ppm)
f1 (ppm)

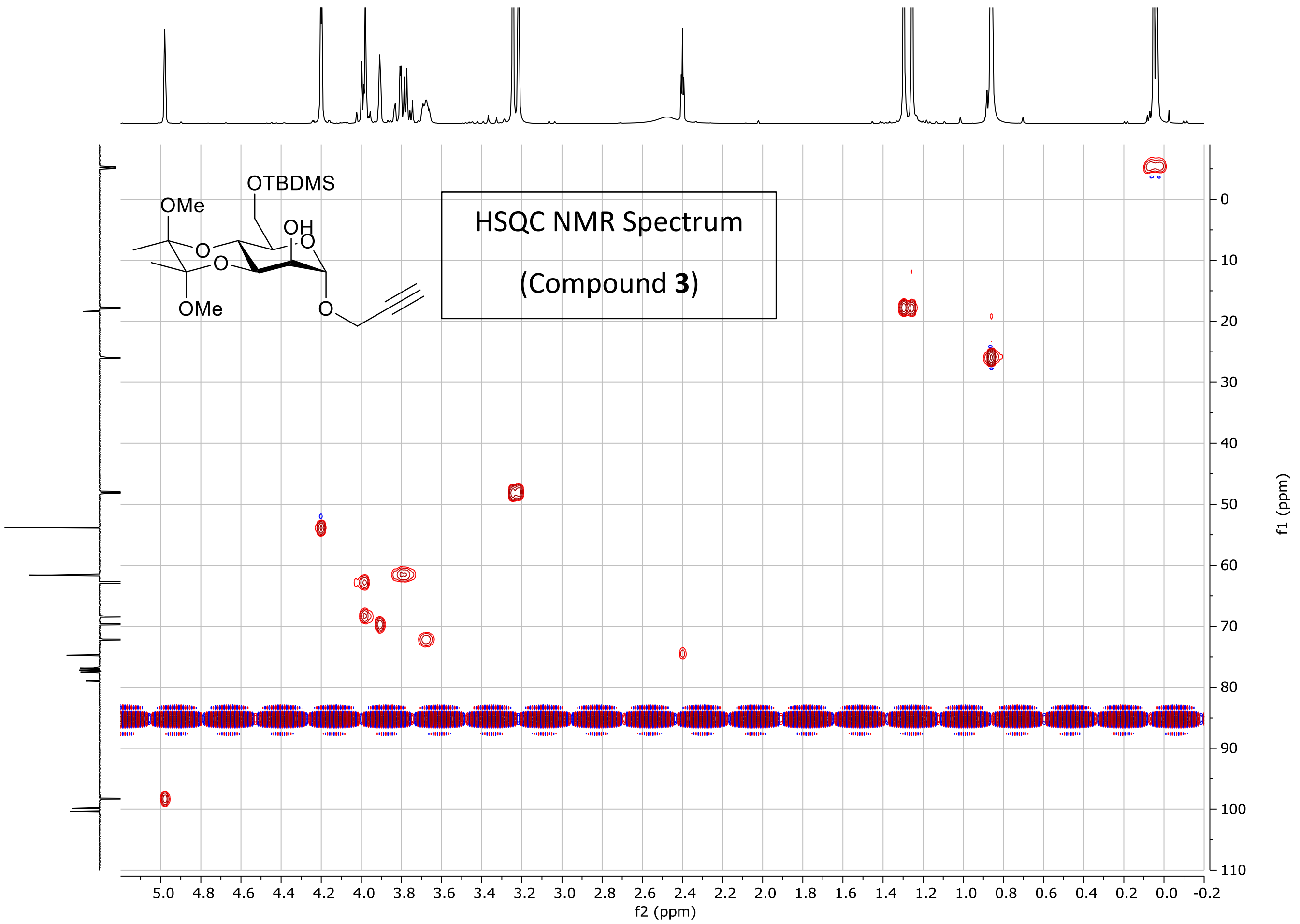
OTBDMS
OMe
OH
O
O
O
OMe
O
HSQC NMR Spectrum
(Compound 3)
f2 (ppm)
f1 (ppm)

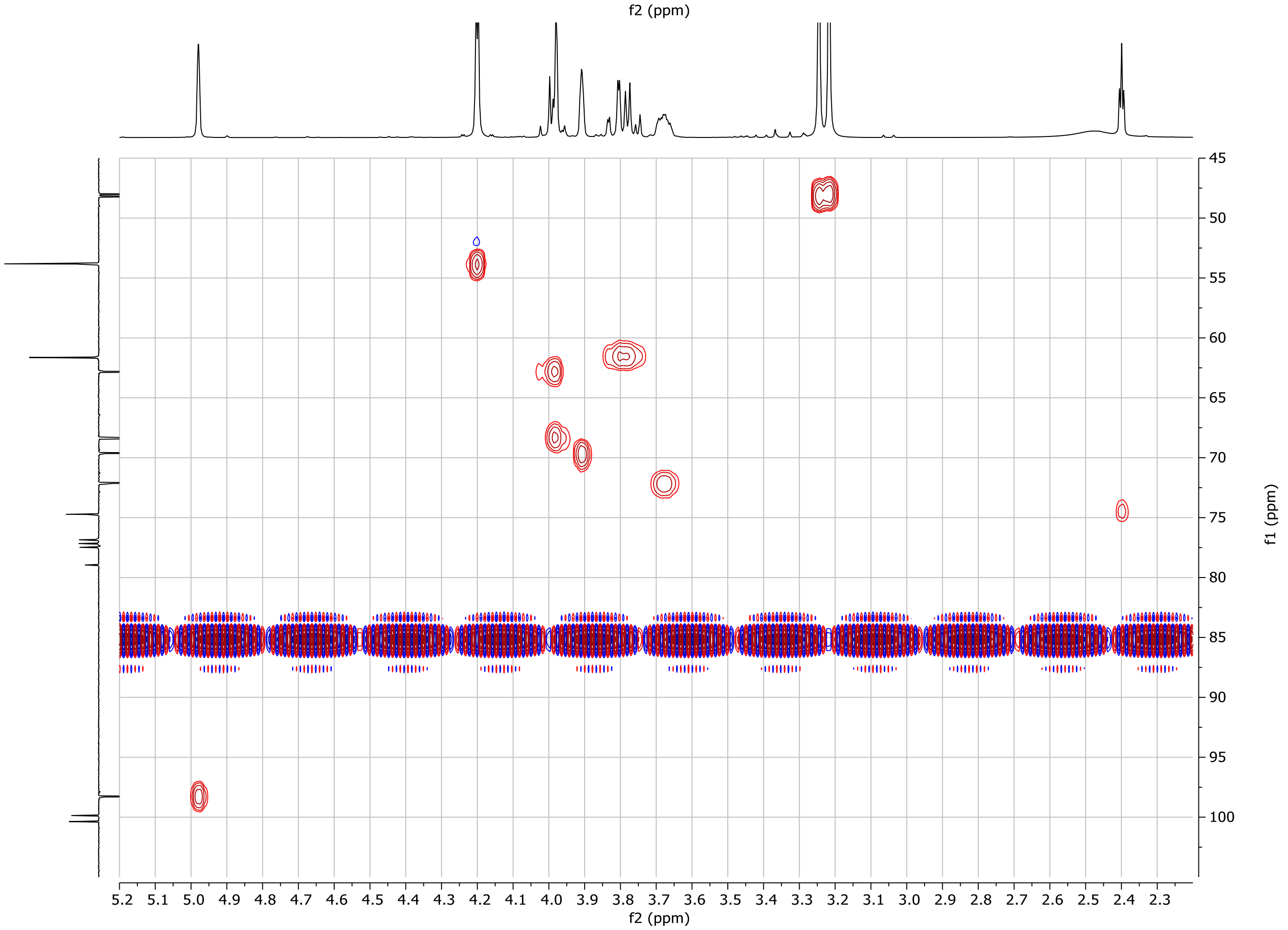
f2 (ppm)
f1 (ppm)

4

^{1}H NMR Spectrum

(Compound **4**)

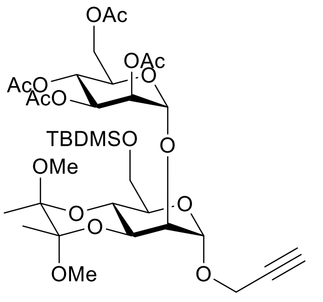

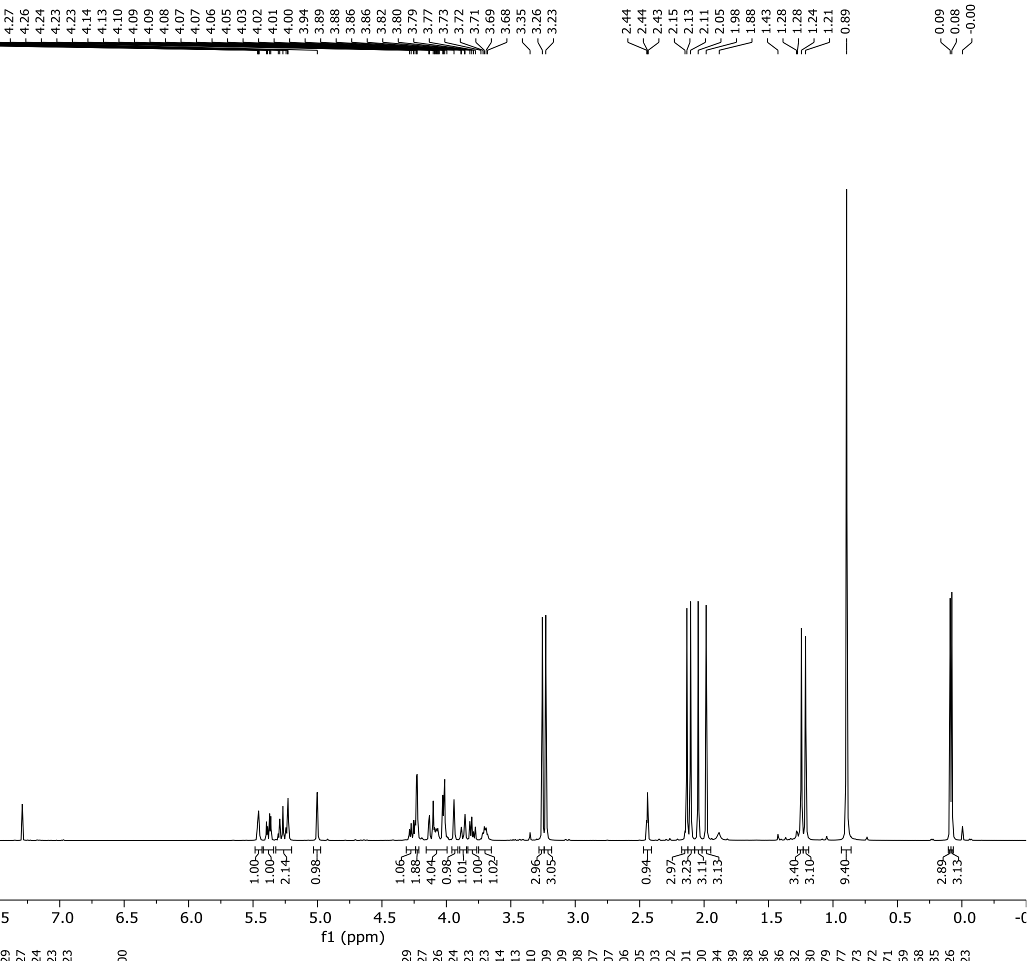

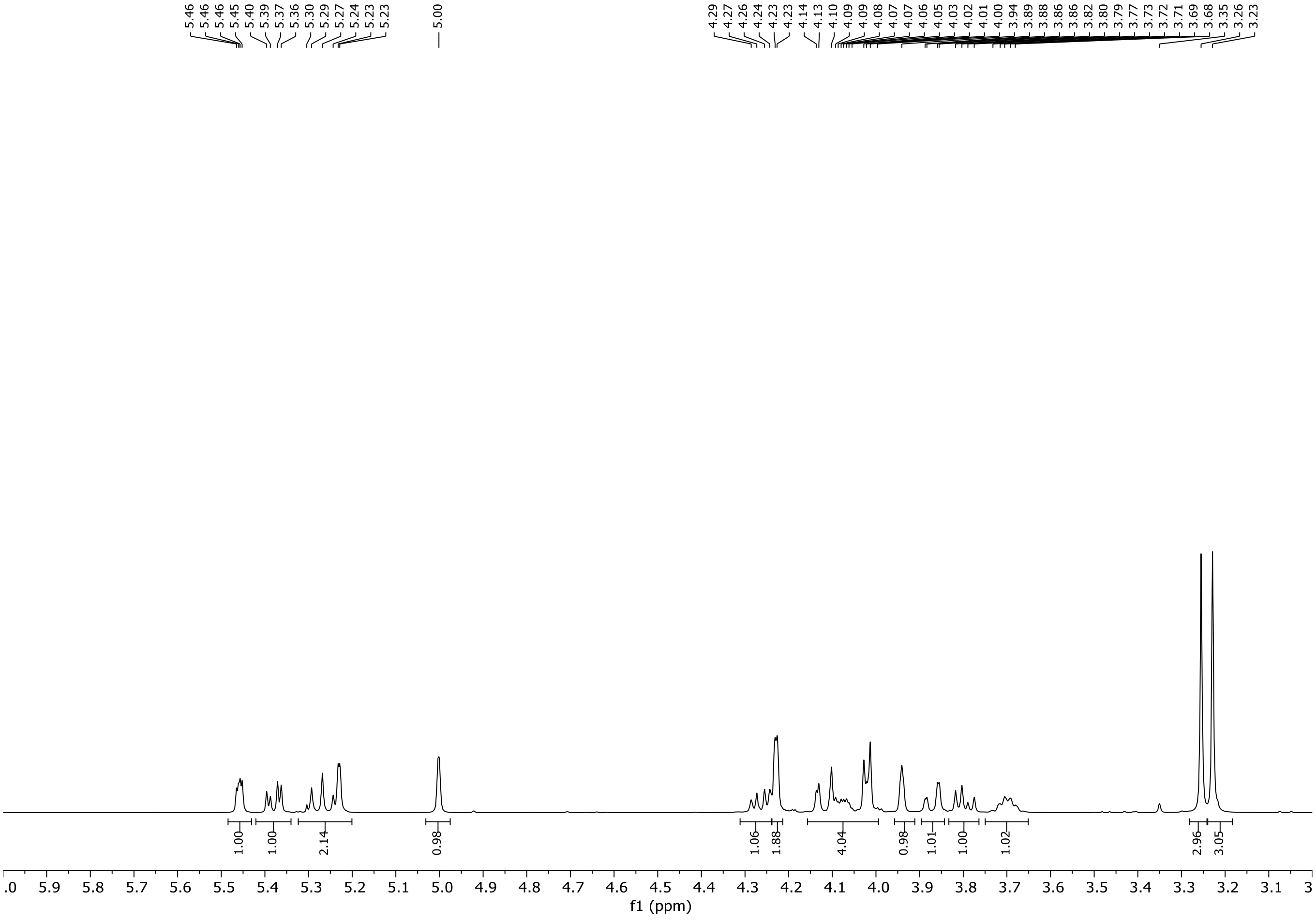

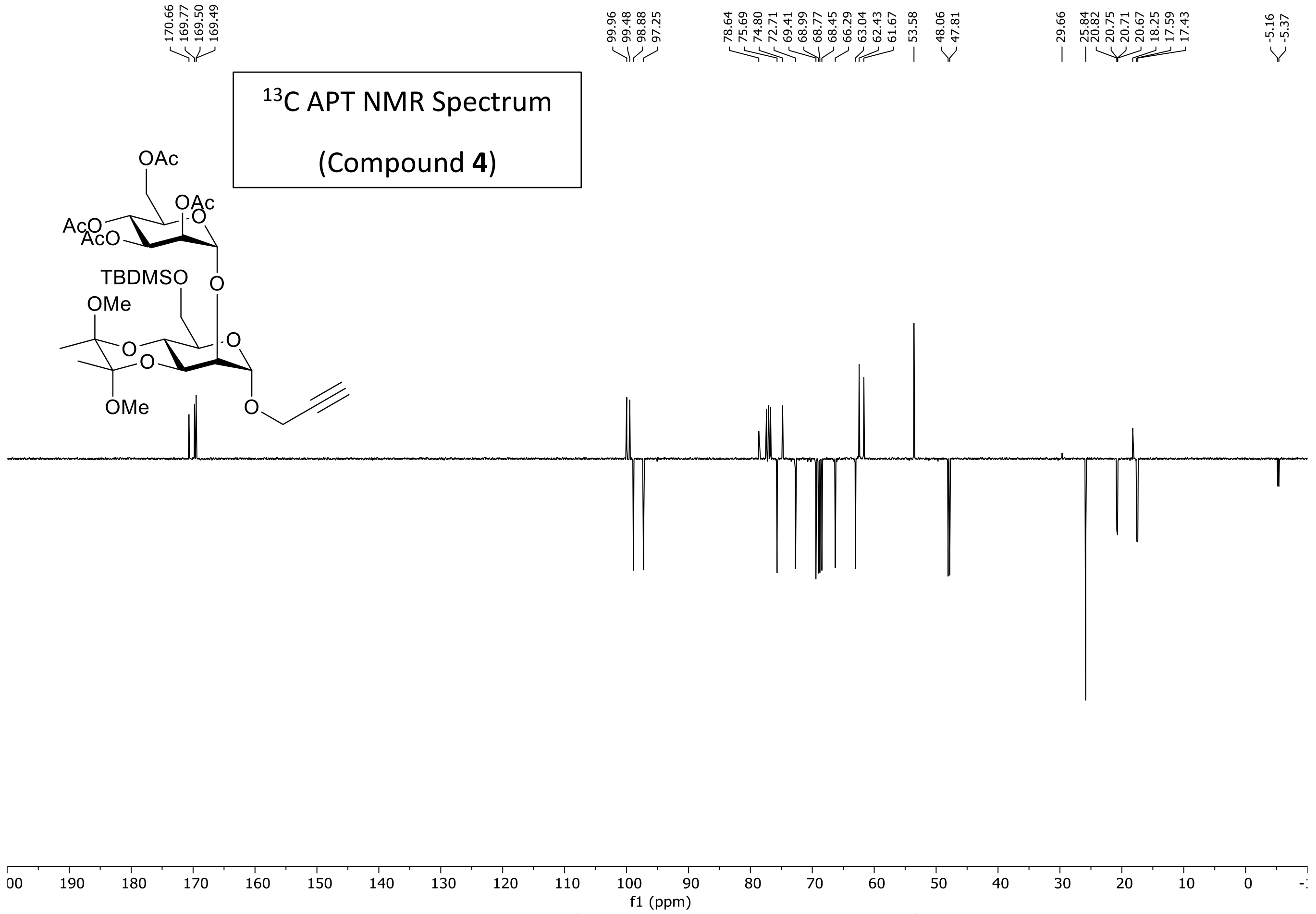
13C APT NMR Spectrum
(Compound 4)
170.66
169.77
169.50
169.49
99.96
99.48
98.88
97.25
78.64
75.69
74.80
72.71
69.41
68.99
68.77
68.45
66.29
63.04
62.43
61.67
53.58
48.06
47.81
29.66
25.84
20.82
20.75
20.71
20.67
18.25
17.59
17.43
-5.16
-5.37
OAc
OAc
AcO
AcO
O
TBDMSO
O
OMe
O
O
O
OMe
O
f1 (ppm)

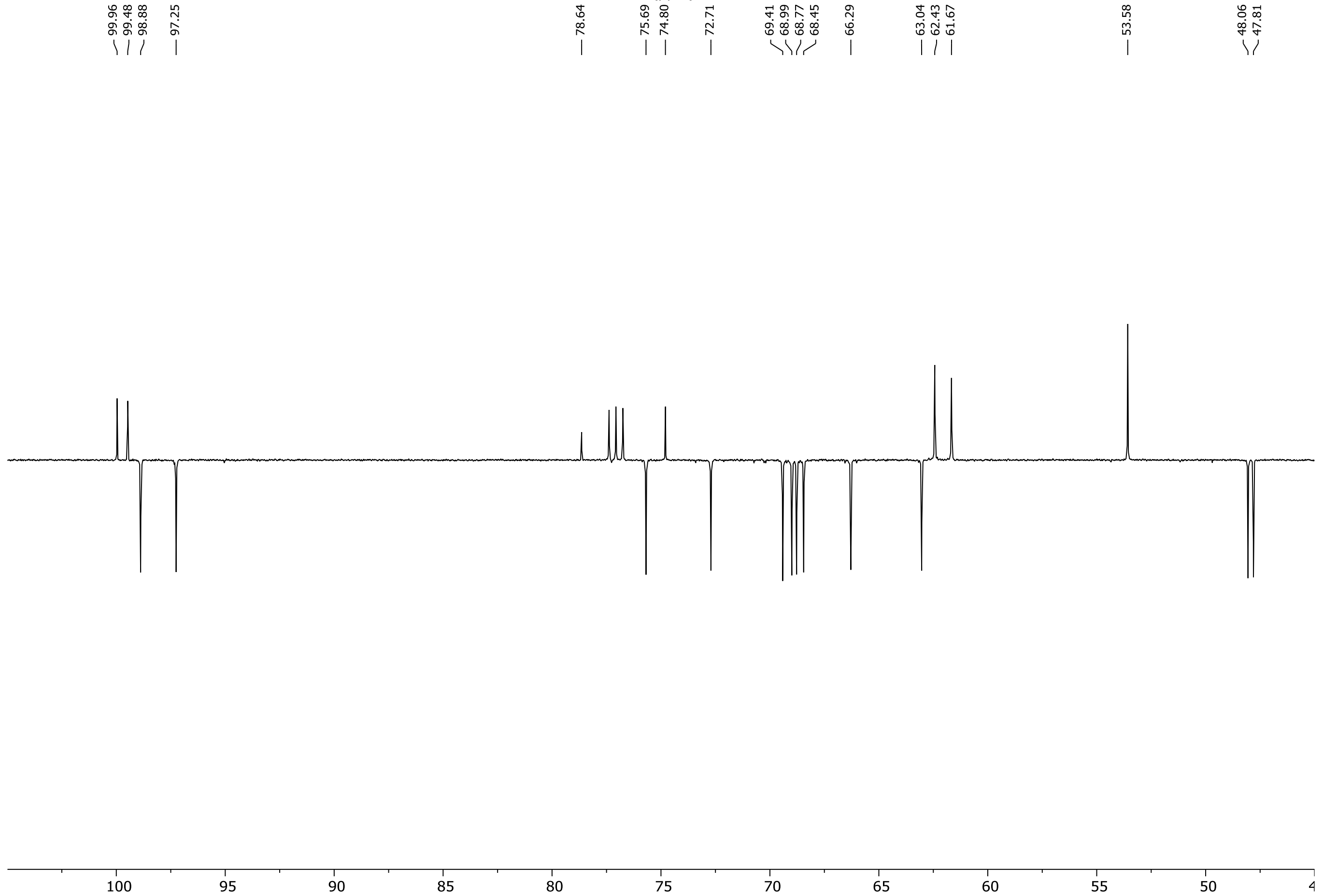
99.96
99.48
98.88
97.25
78.64
75.69
74.80
72.71
69.41
68.99
68.77
68.45
66.29
63.04
62.43
61.67
53.58
48.06
47.81
f1 (ppm)

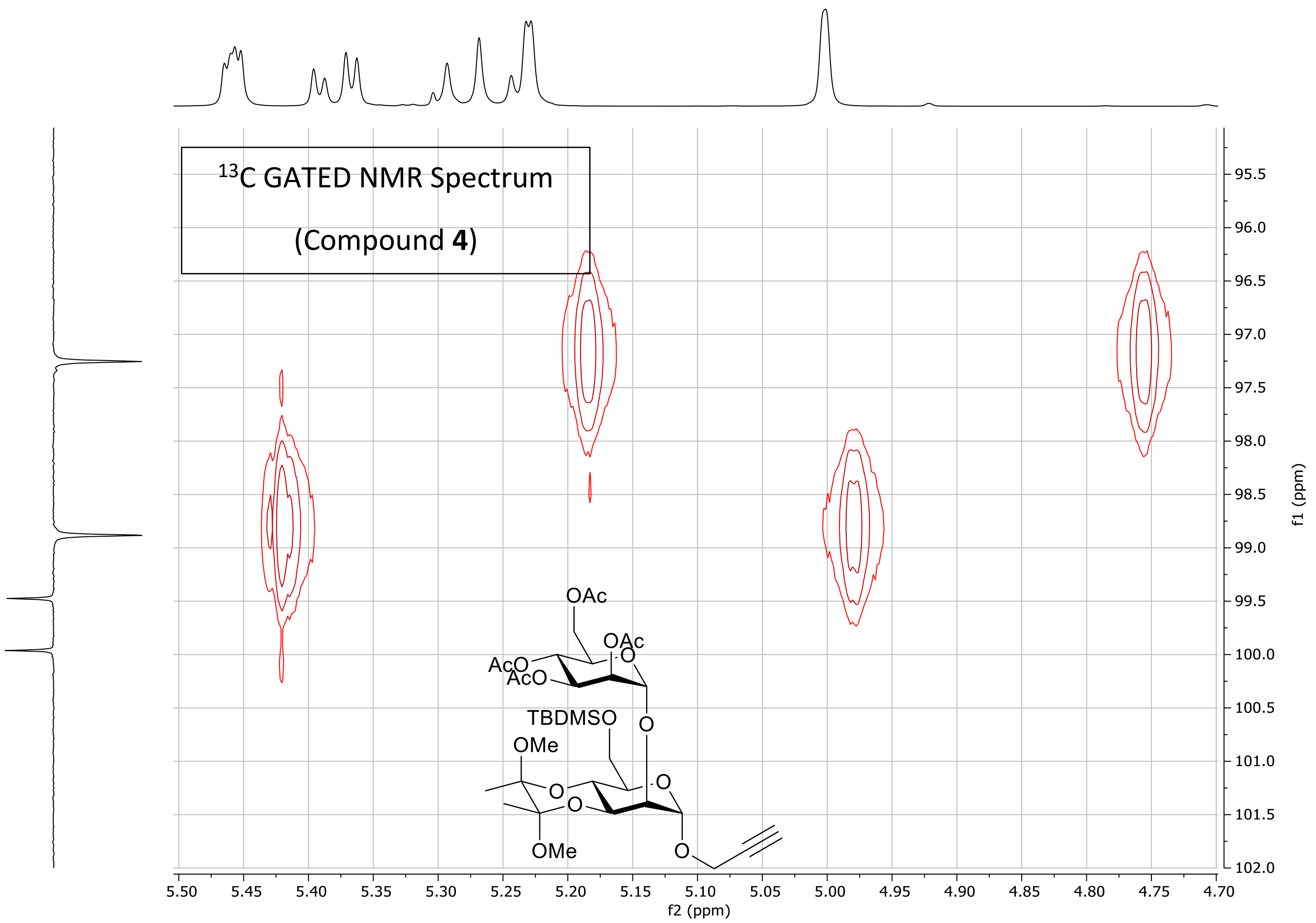

13C GATED NMR Spectrum
(Compound 4)
OAc
OAc
AcO
AcO
TBDMSO
OMe
OMe
f2 (ppm)
f1 (ppm)

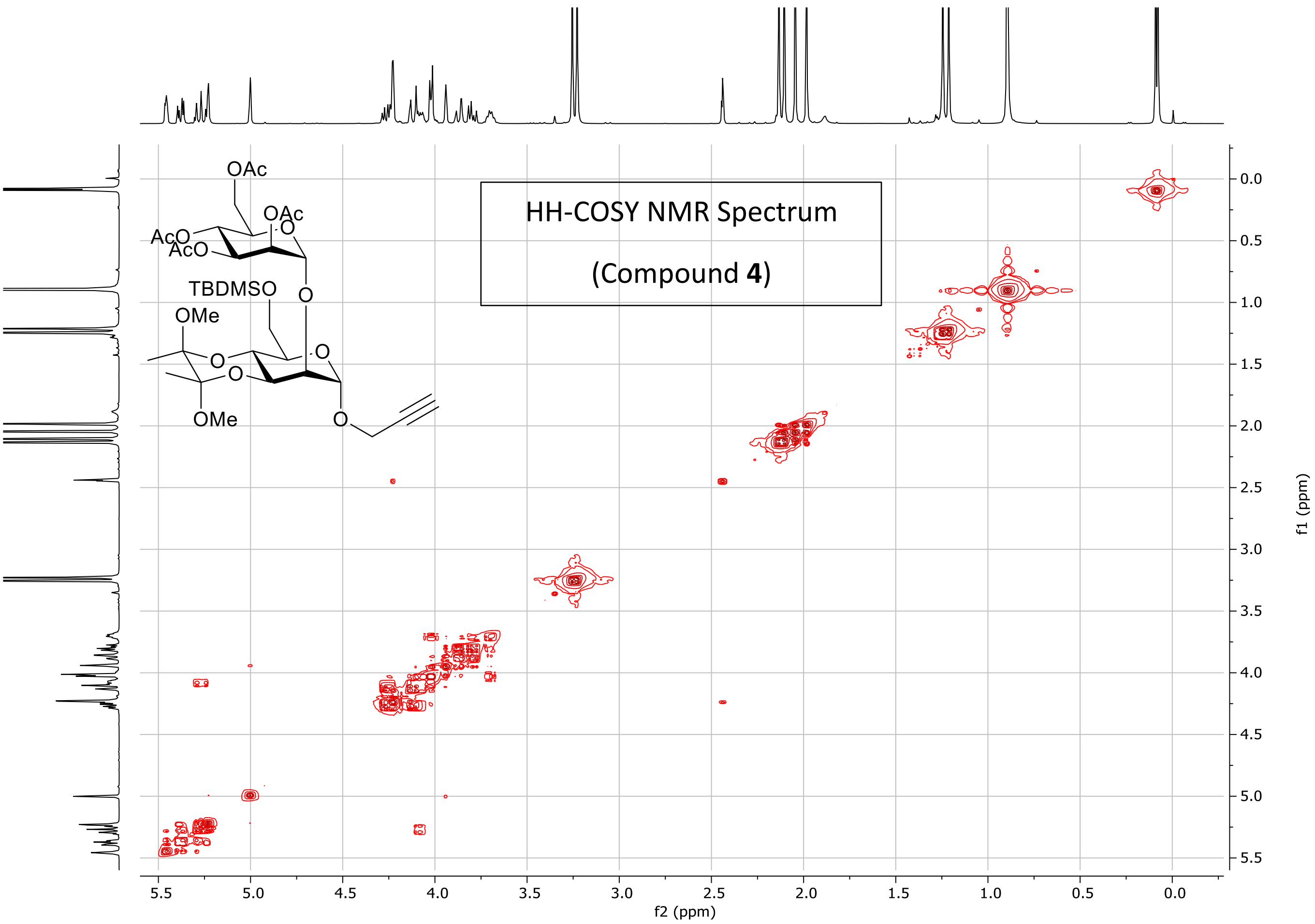
HH-COSY NMR Spectrum
(Compound **4**)
OAc
OAc
AcO
AcO
O
TBDMSO
O
OMe
O
O
O
OMe
O
f2 (ppm)
f1 (ppm)

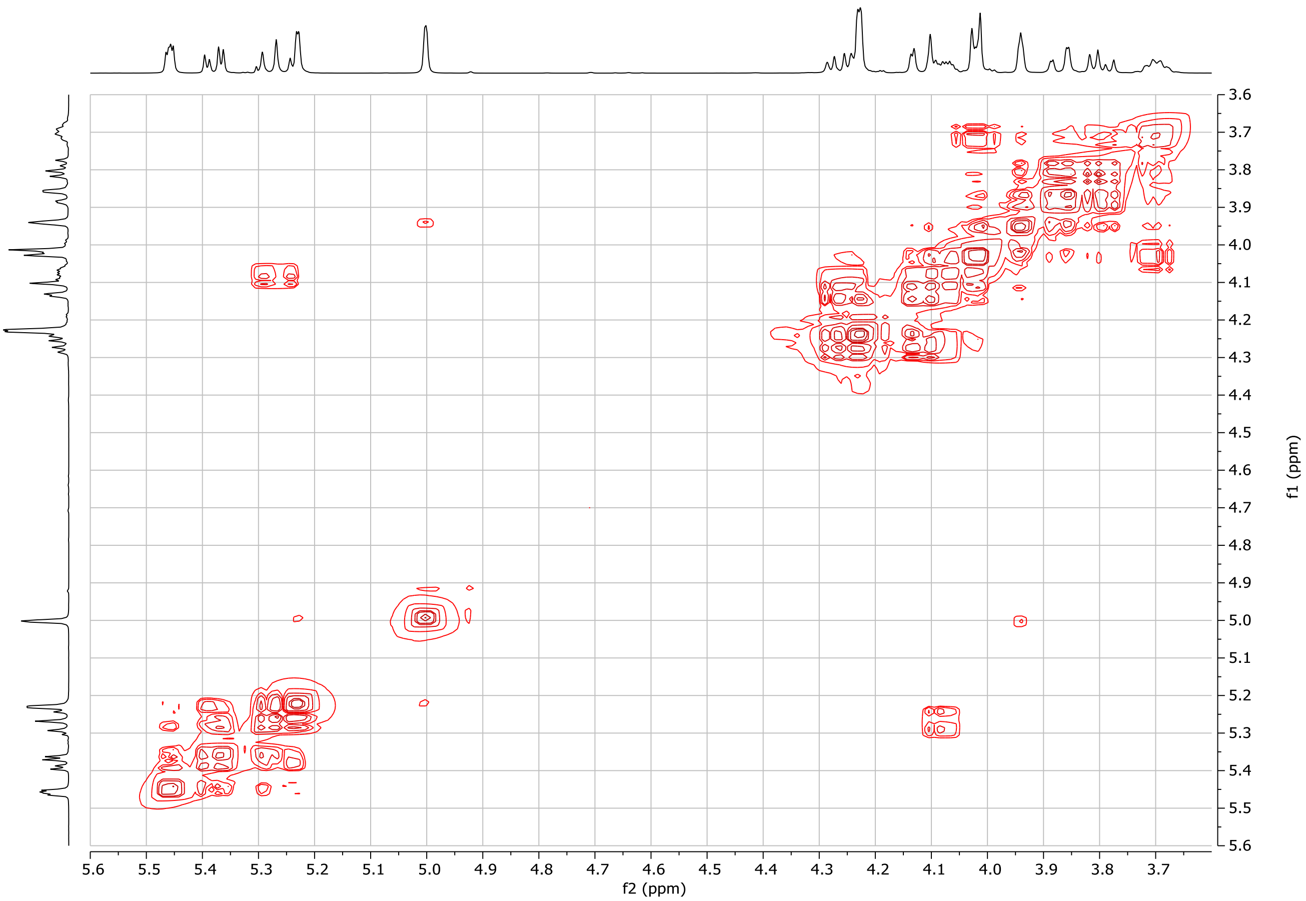
f2 (ppm)
f1 (ppm)

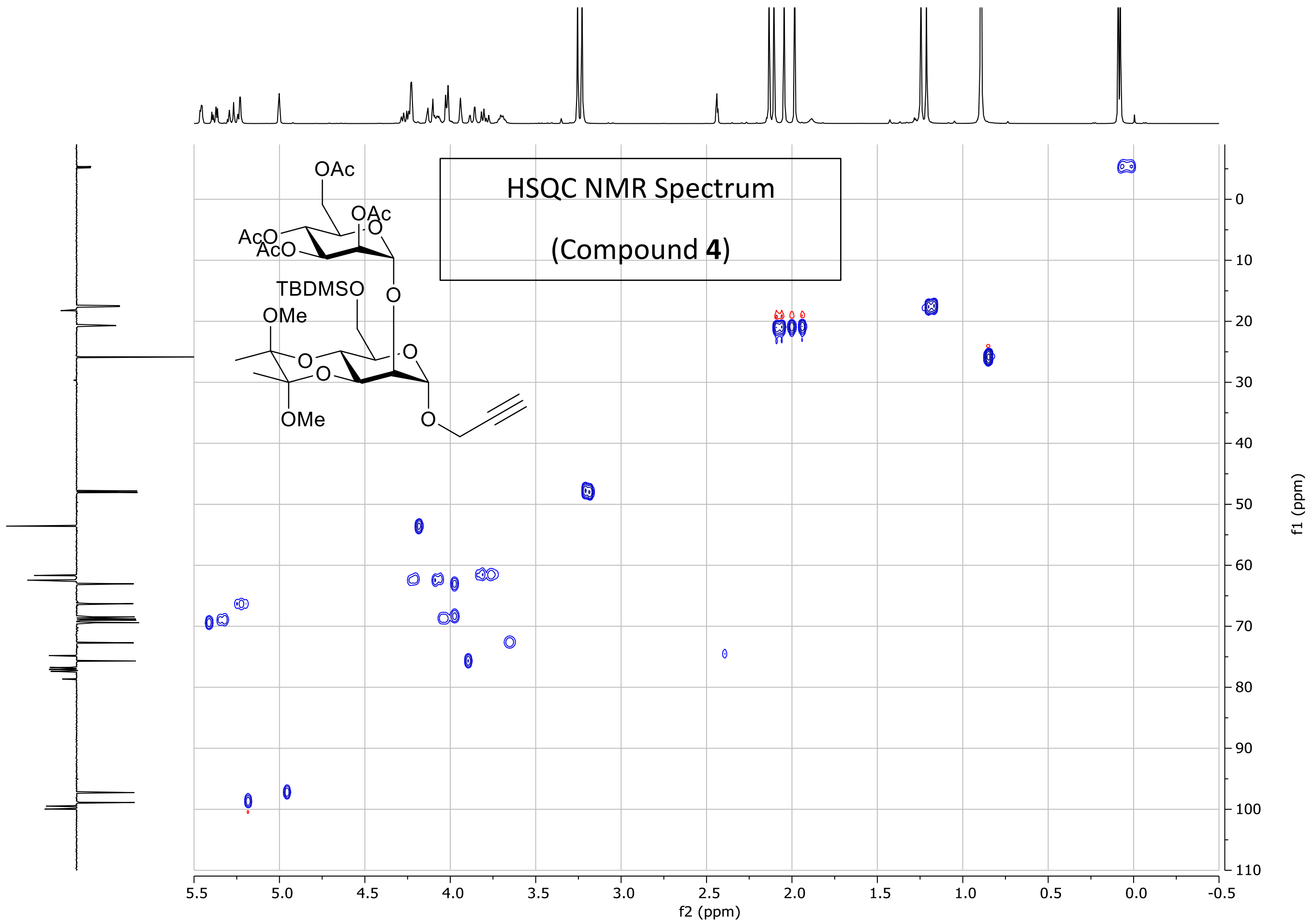
HSQC NMR Spectrum
(Compound 4)
OAc
OAc
AcO
AcO
O
TBDMSO
O
OMe
O
O
O
OMe
O
f2 (ppm)
f1 (ppm)

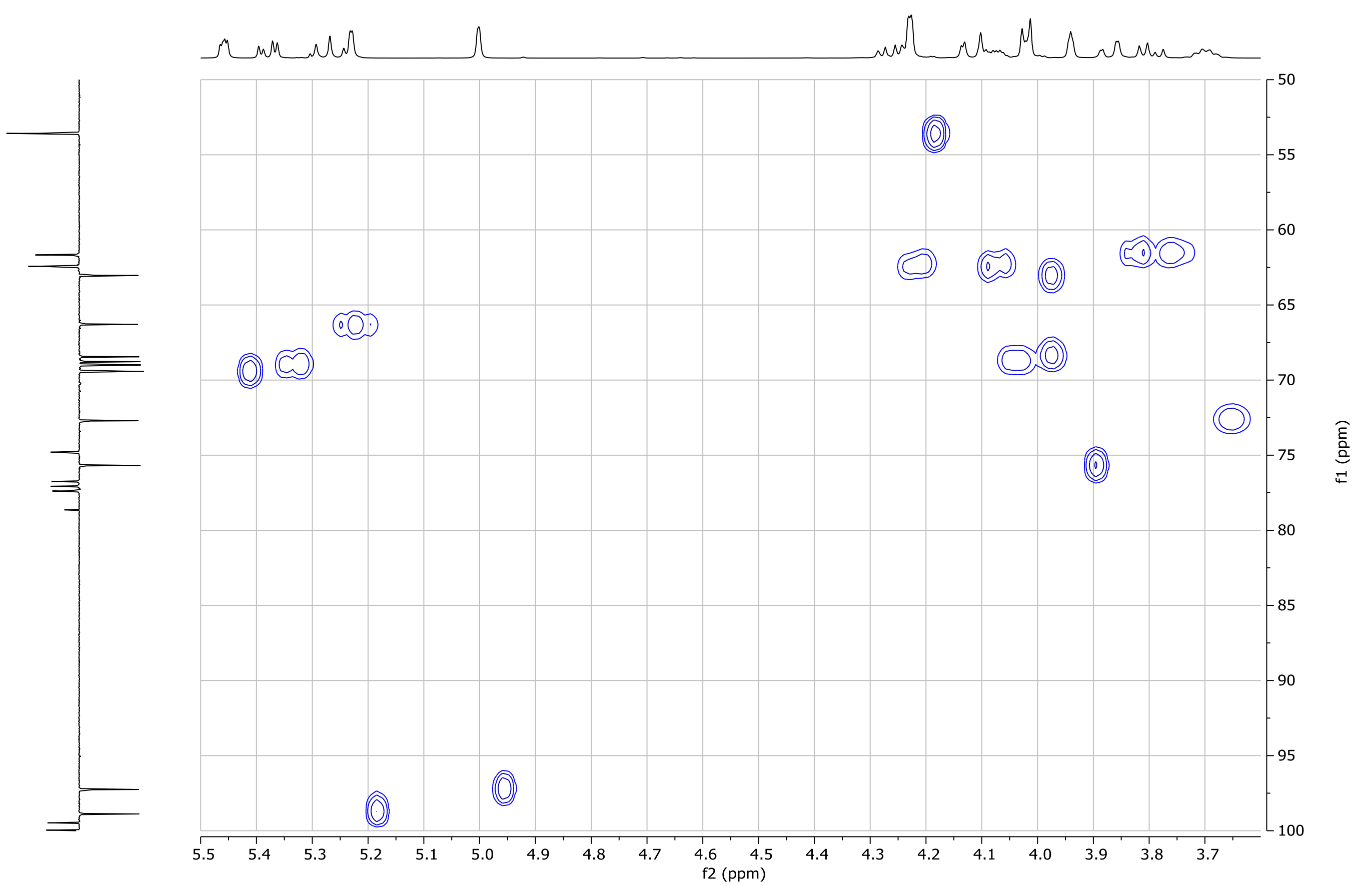
f2 (ppm)
f1 (ppm)

S3

^{1}H NMR Spectrum

(Compound **S3**)

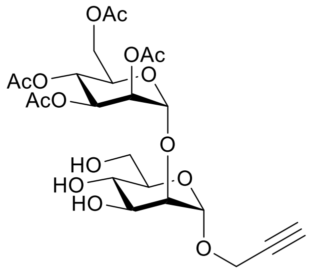

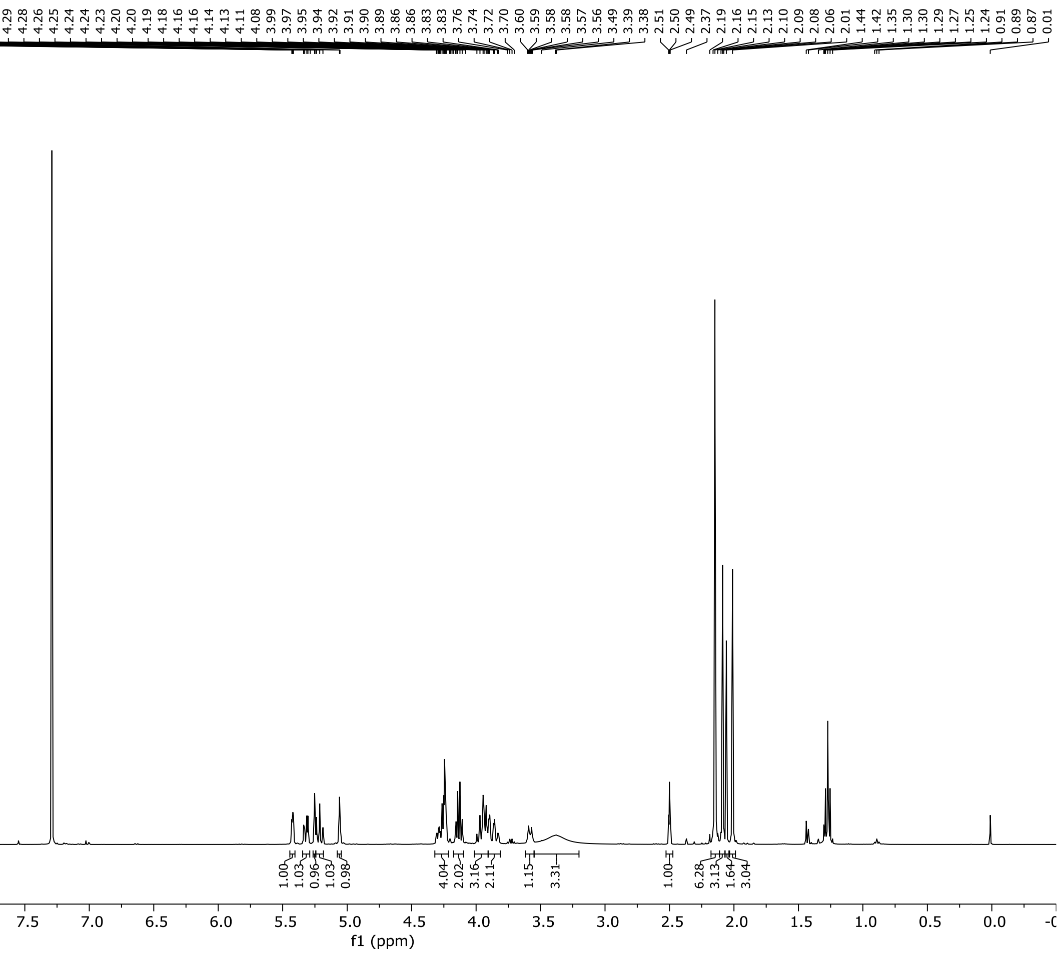

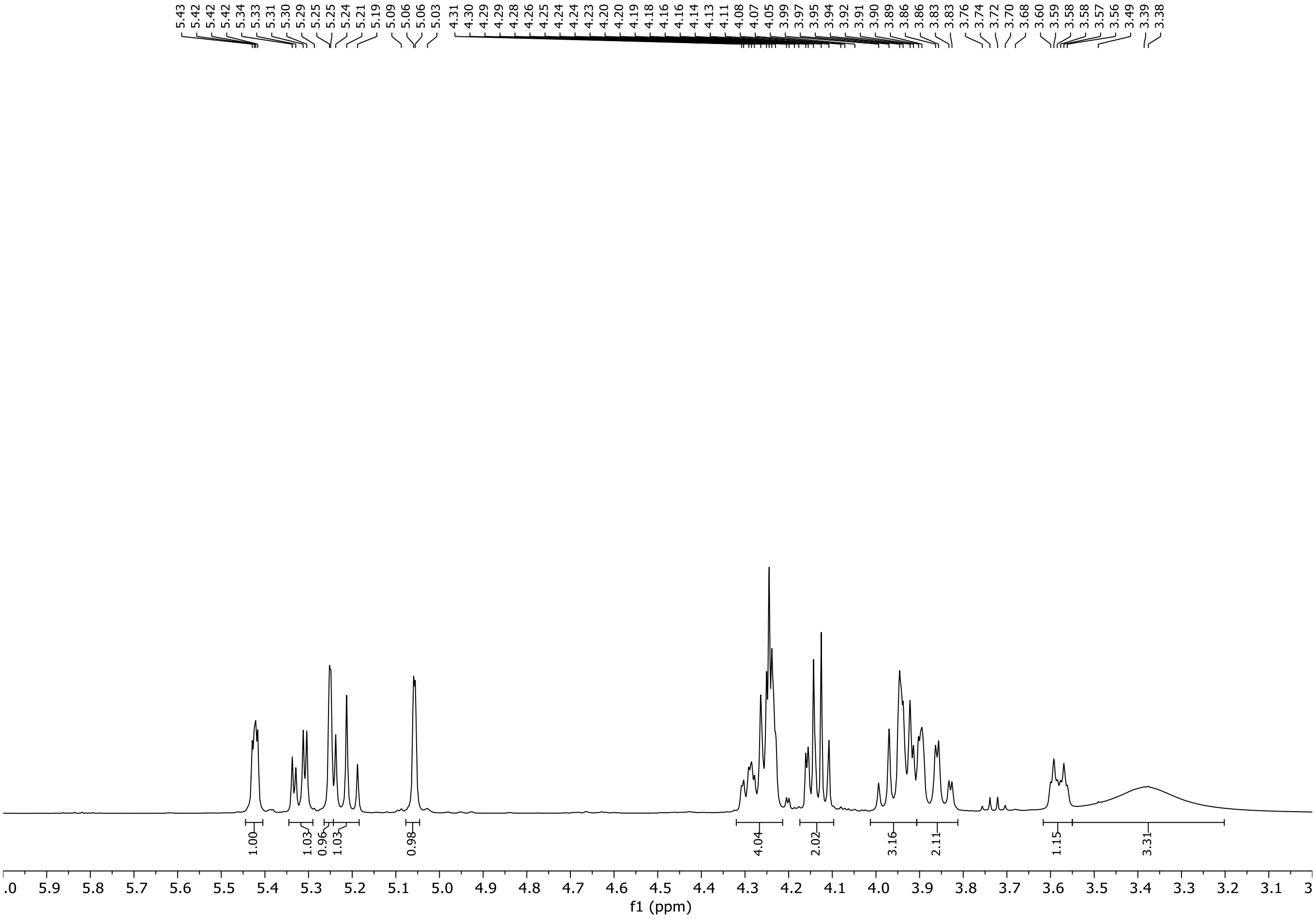

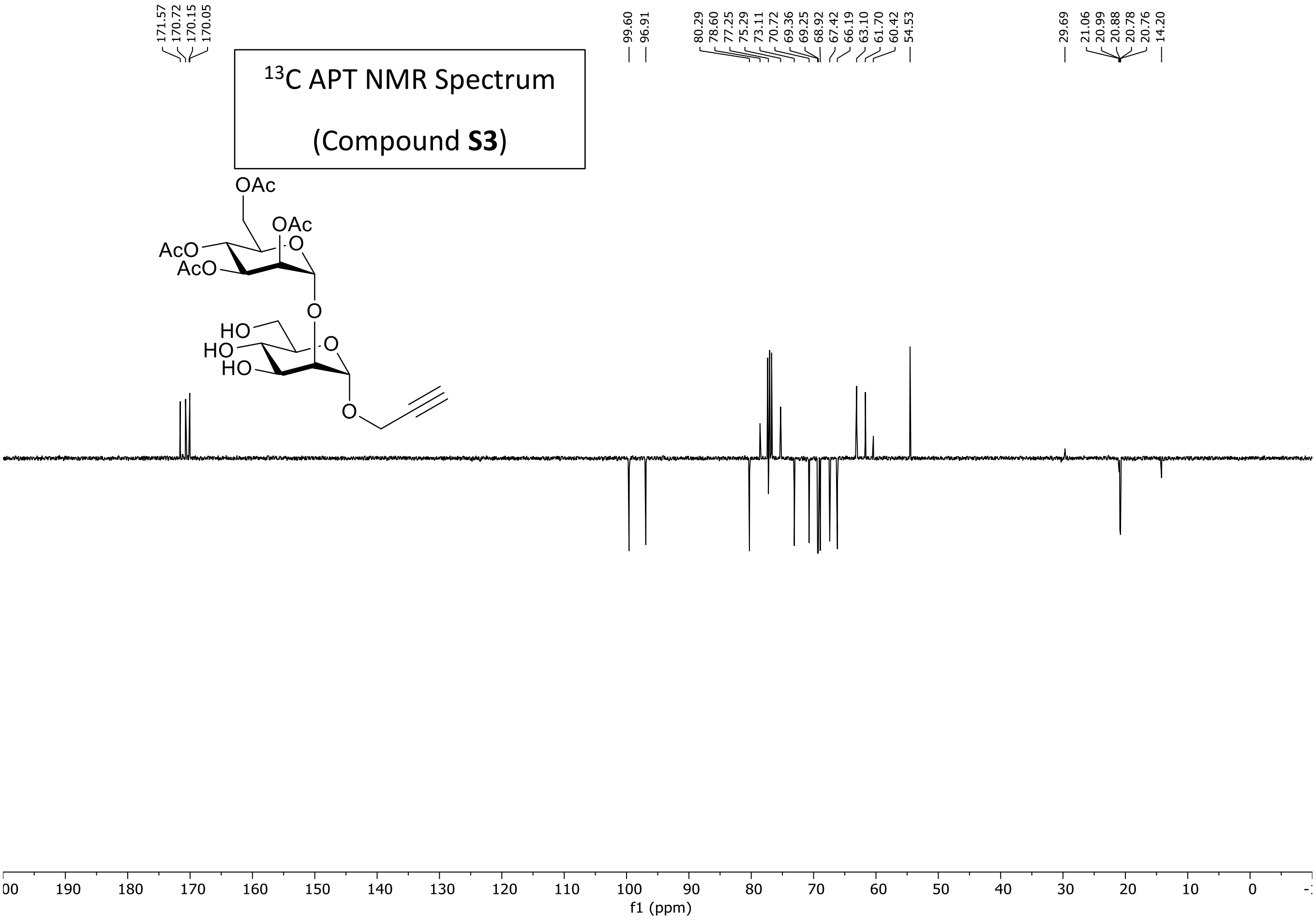
13C APT NMR Spectrum
(Compound S3)
171.57
170.72
170.15
170.05
99.60
96.91
80.29
78.60
77.25
75.29
73.11
70.72
69.36
69.25
68.92
67.42
66.19
63.10
61.70
60.42
54.53
29.69
21.06
20.99
20.88
20.78
20.76
14.20
OAc
OAc
AcO
AcO
O
O
HO
HO
HO
O
O
f1 (ppm)

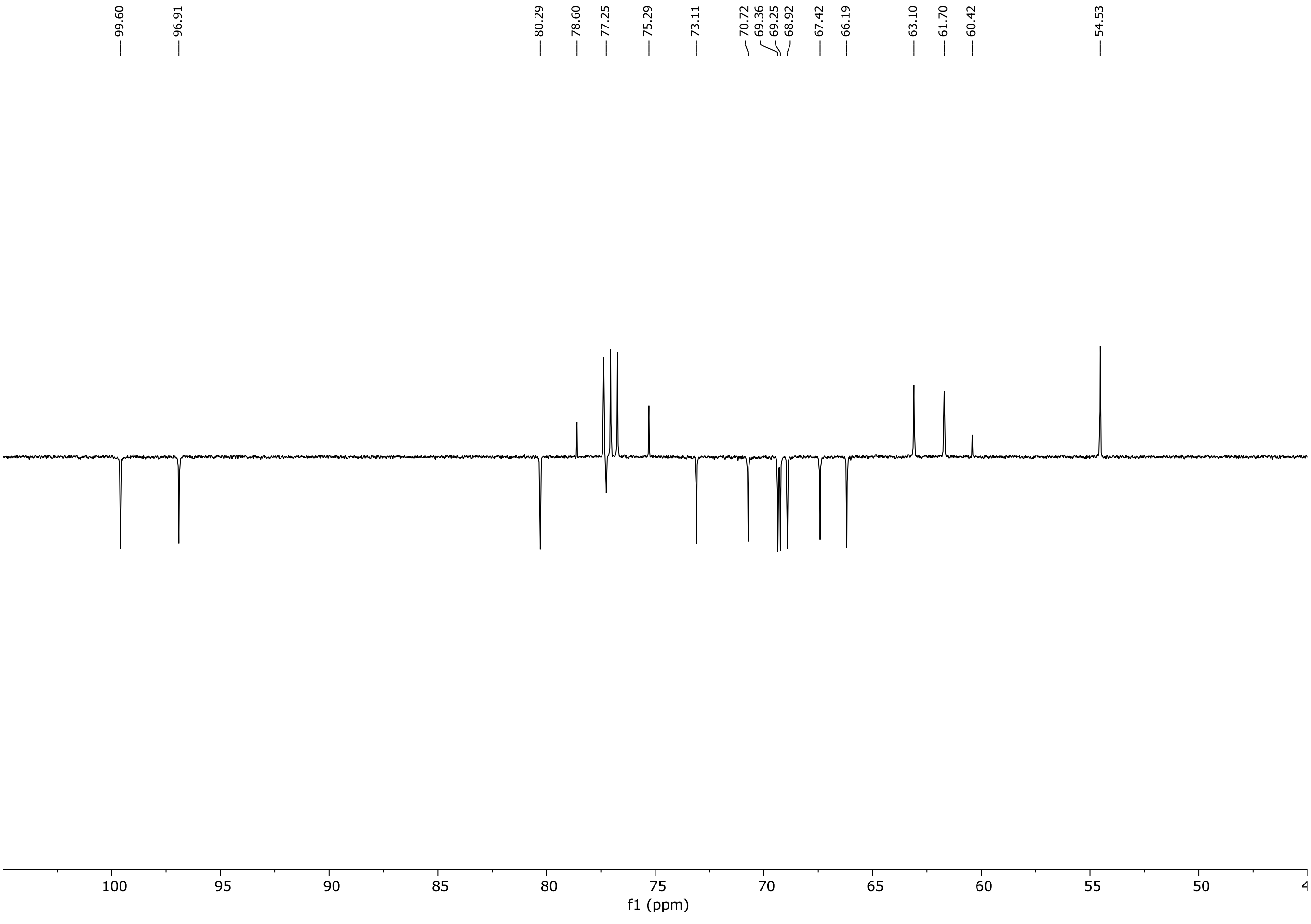
99.60
96.91
80.29
78.60
77.25
75.29
73.11
70.72
69.36
69.25
68.92
67.42
66.19
63.10
61.70
60.42
54.53
f1 (ppm)

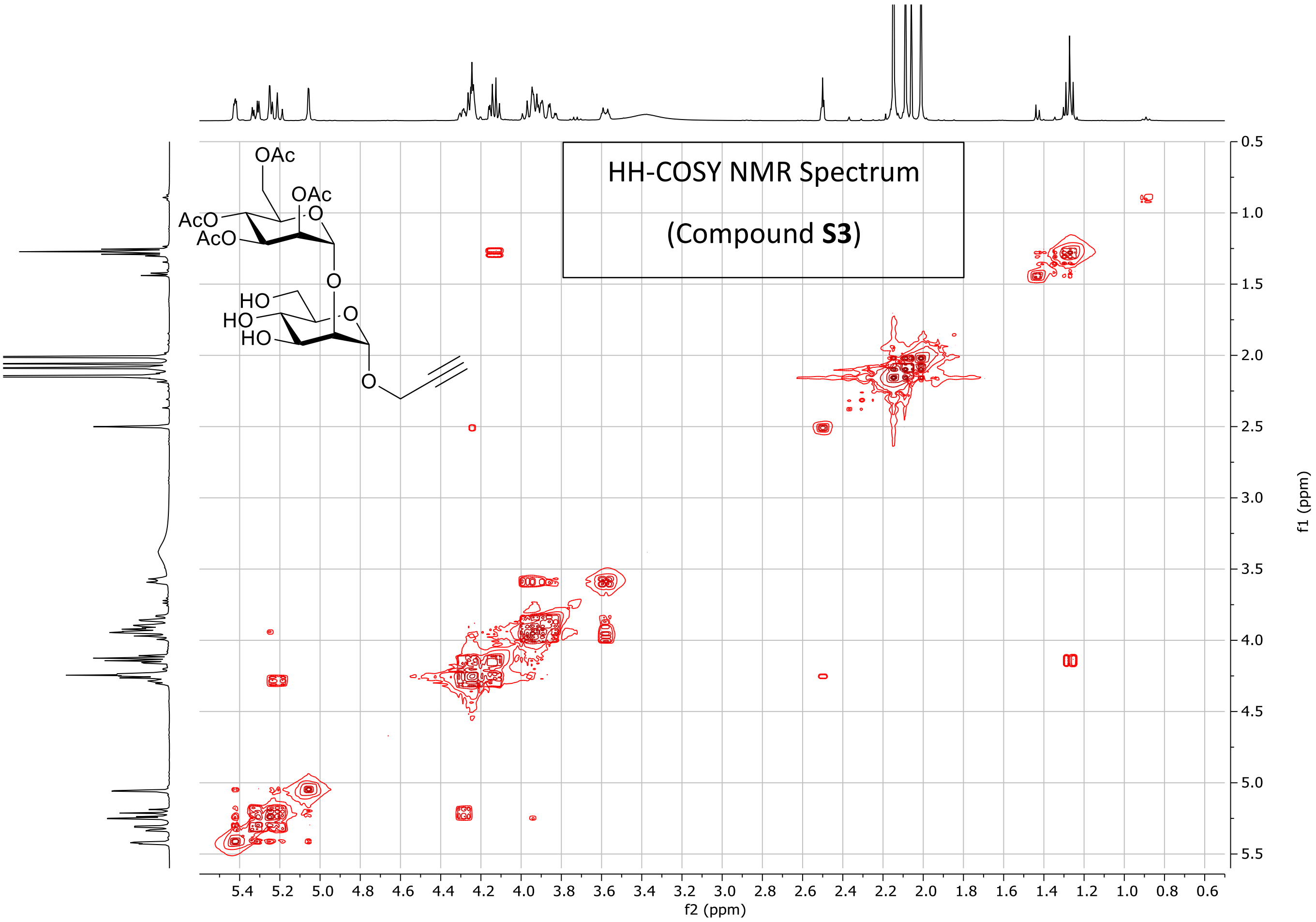
HH-COSY NMR Spectrum
(Compound **S3**)
OAc
OAc
AcO
AcO
O
O
HO
HO
HO
O
O
f1 (ppm)
f2 (ppm)

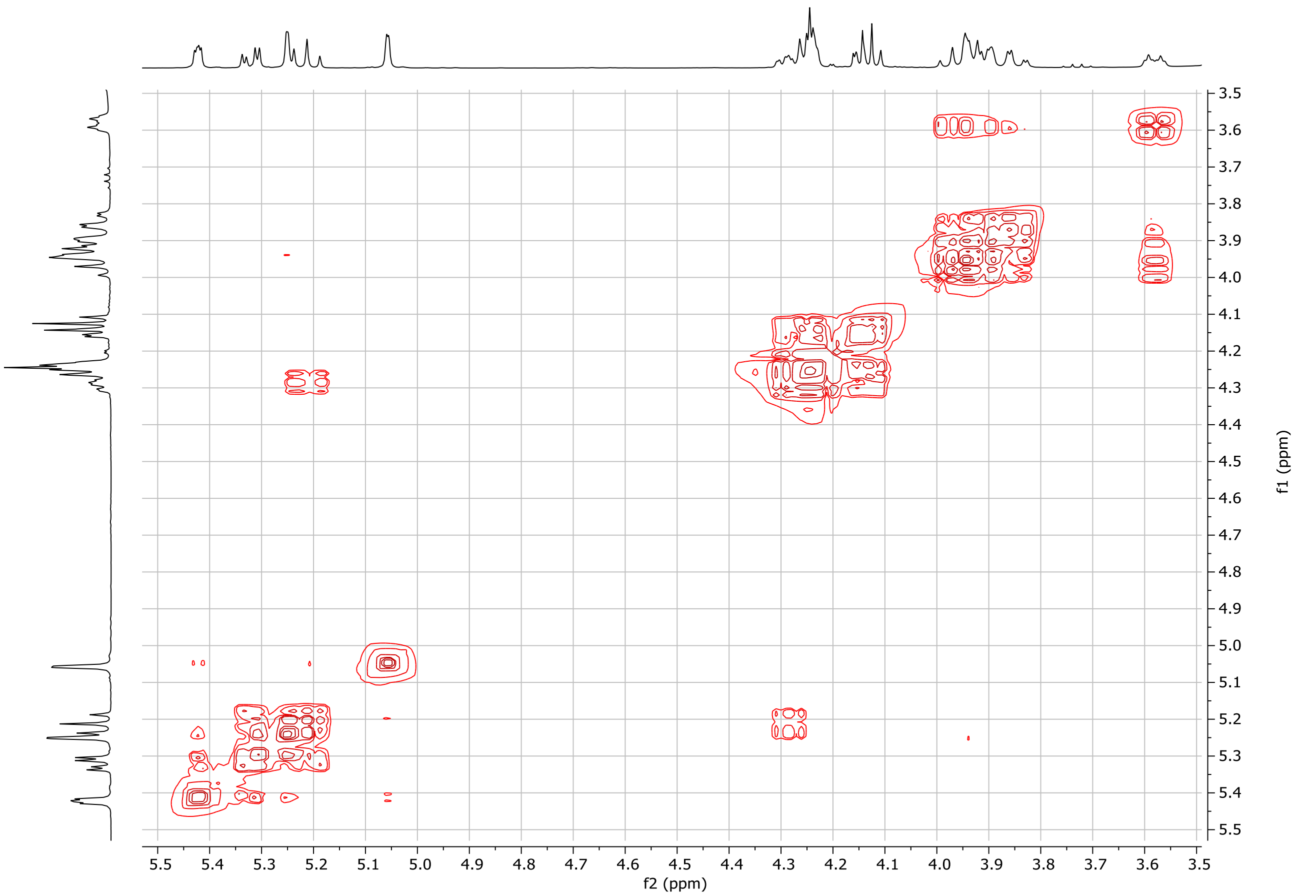
f1 (ppm)
f2 (ppm)

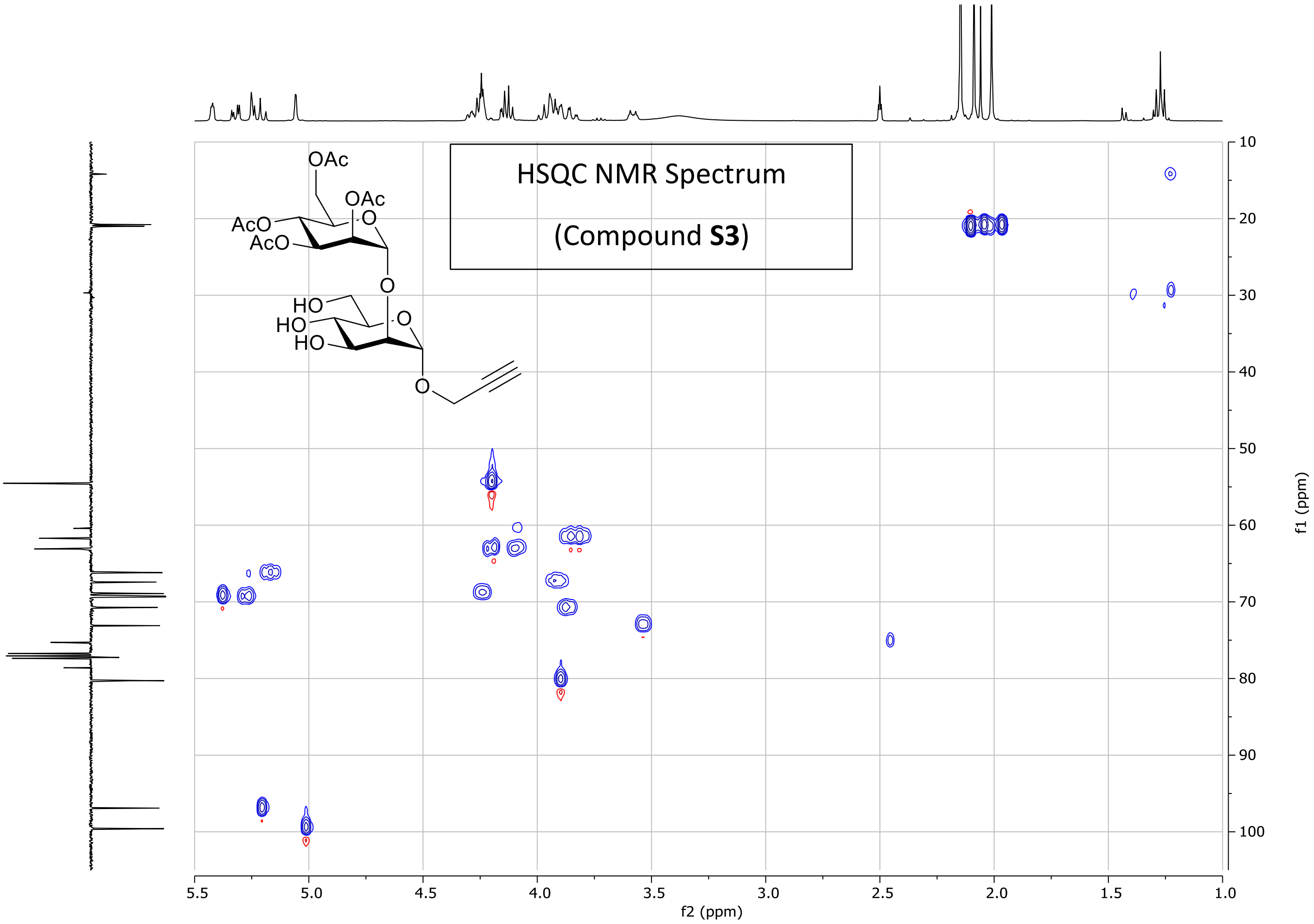
HSQC NMR Spectrum
(Compound **S3**)
OAc
OAc
AcO
AcO
O
O
HO
HO
HO
O
O
f2 (ppm)
f1 (ppm)

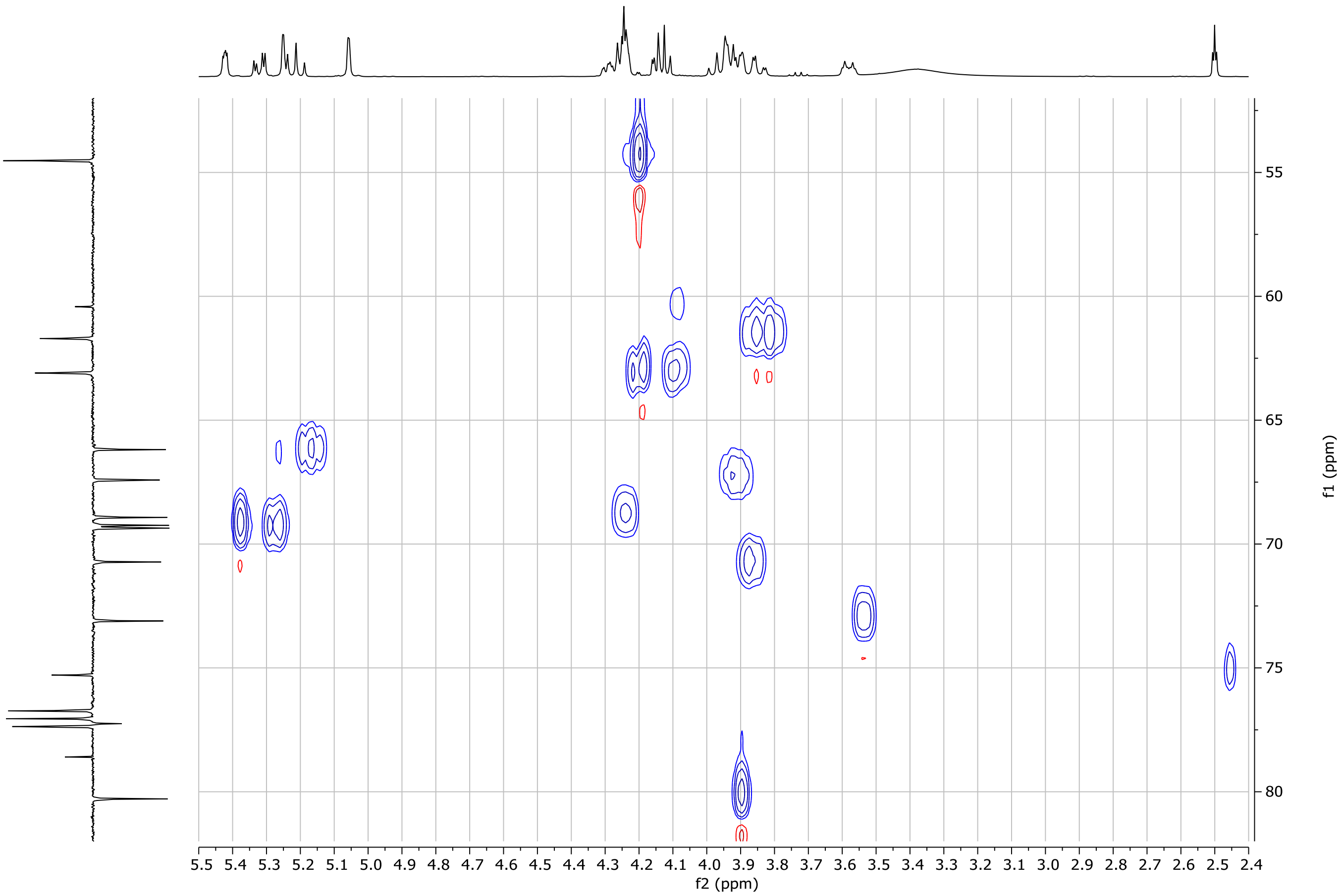
f2 (ppm)
f1 (ppm)

5

^1H NMR Spectrum

(Compound **5**)

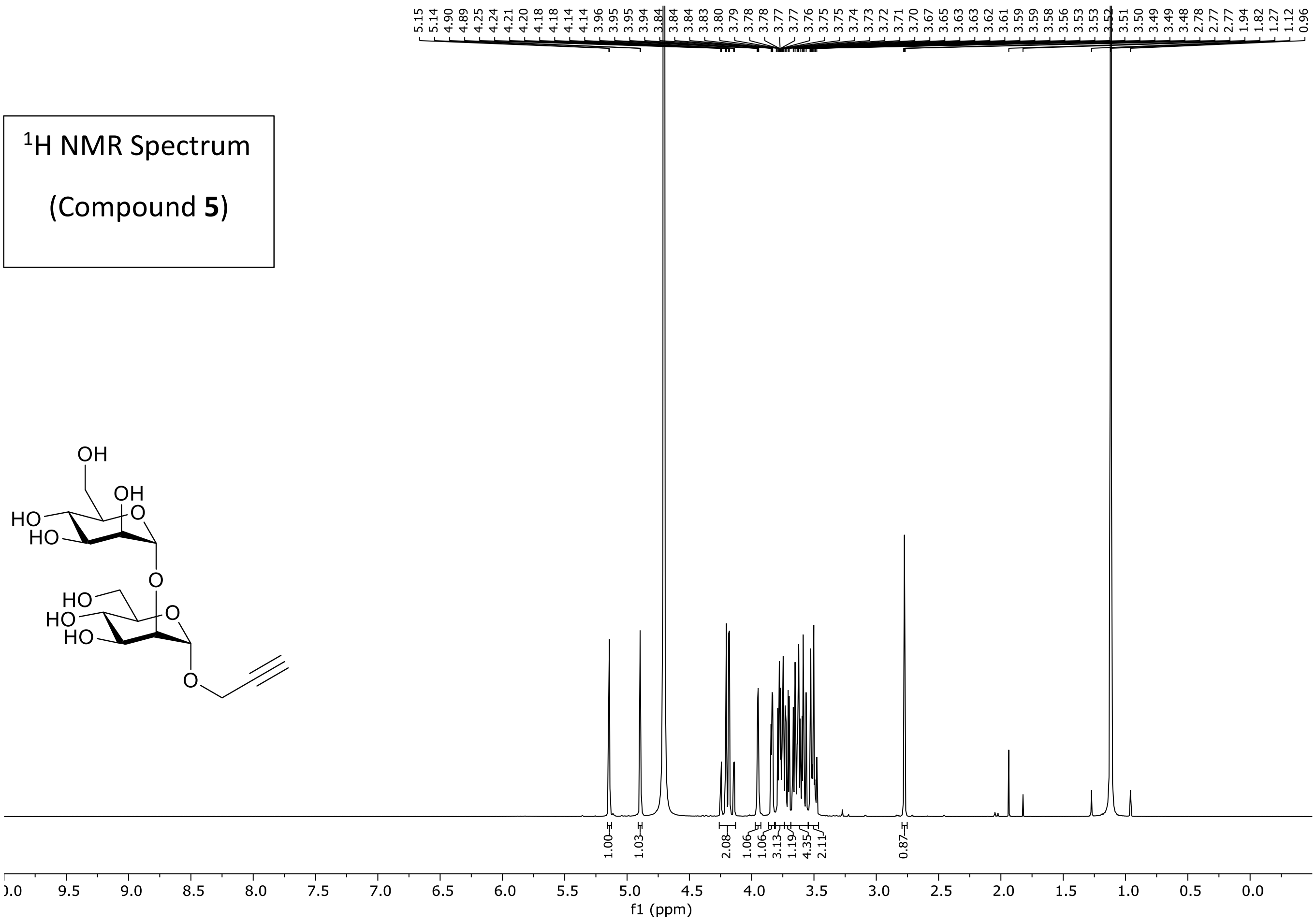

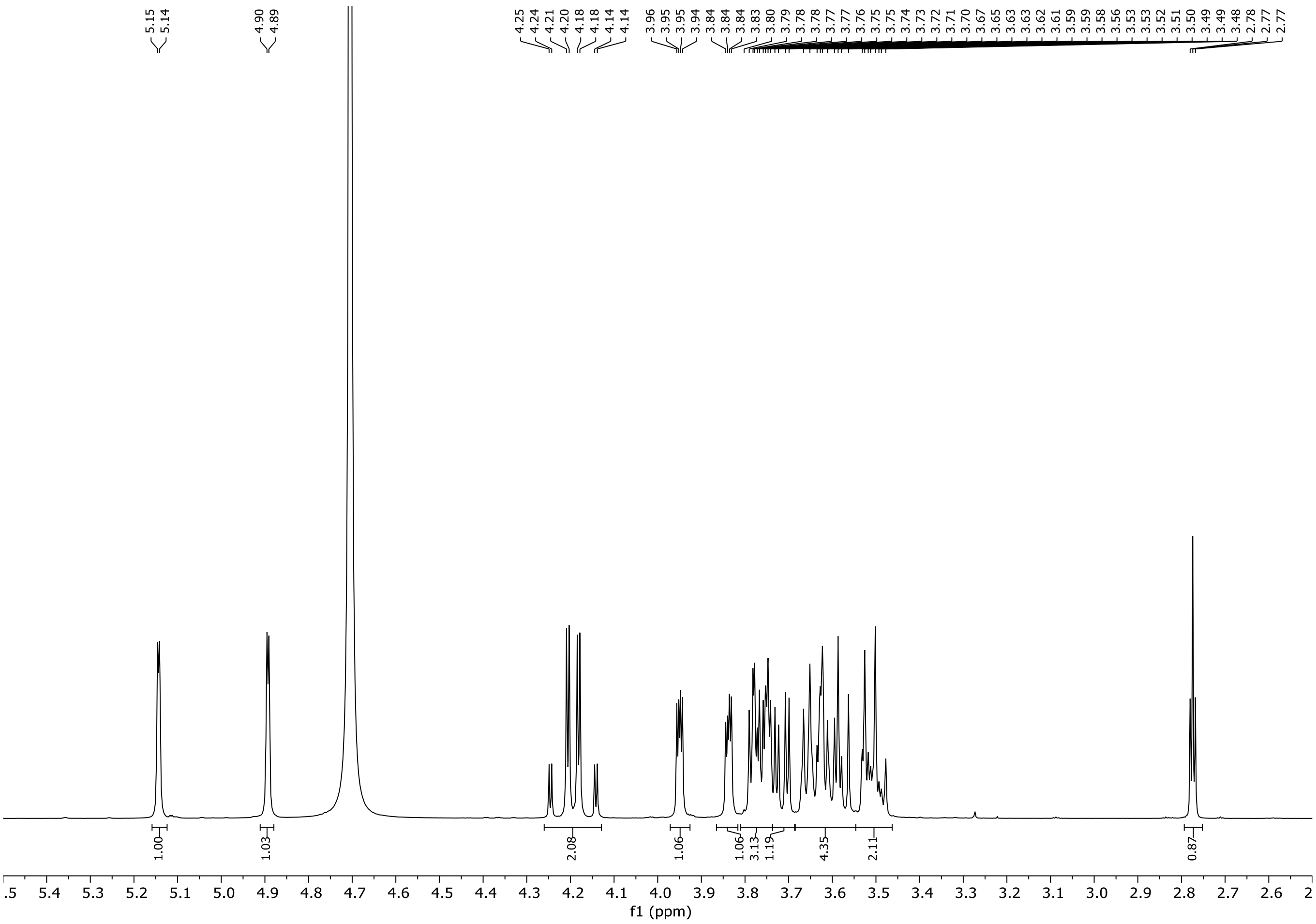

^{13}C APT NMR Spectrum

(Compound **5**)

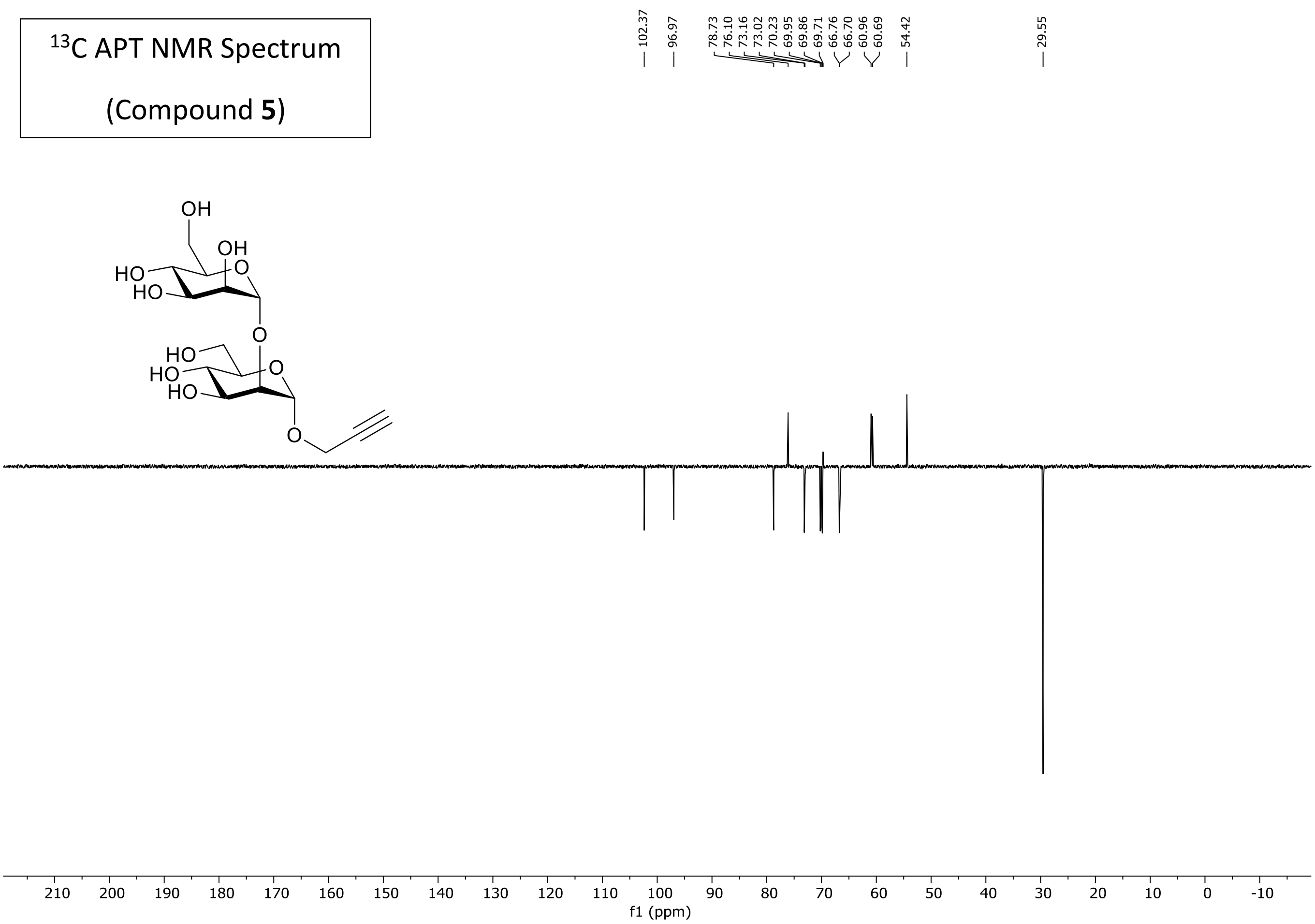

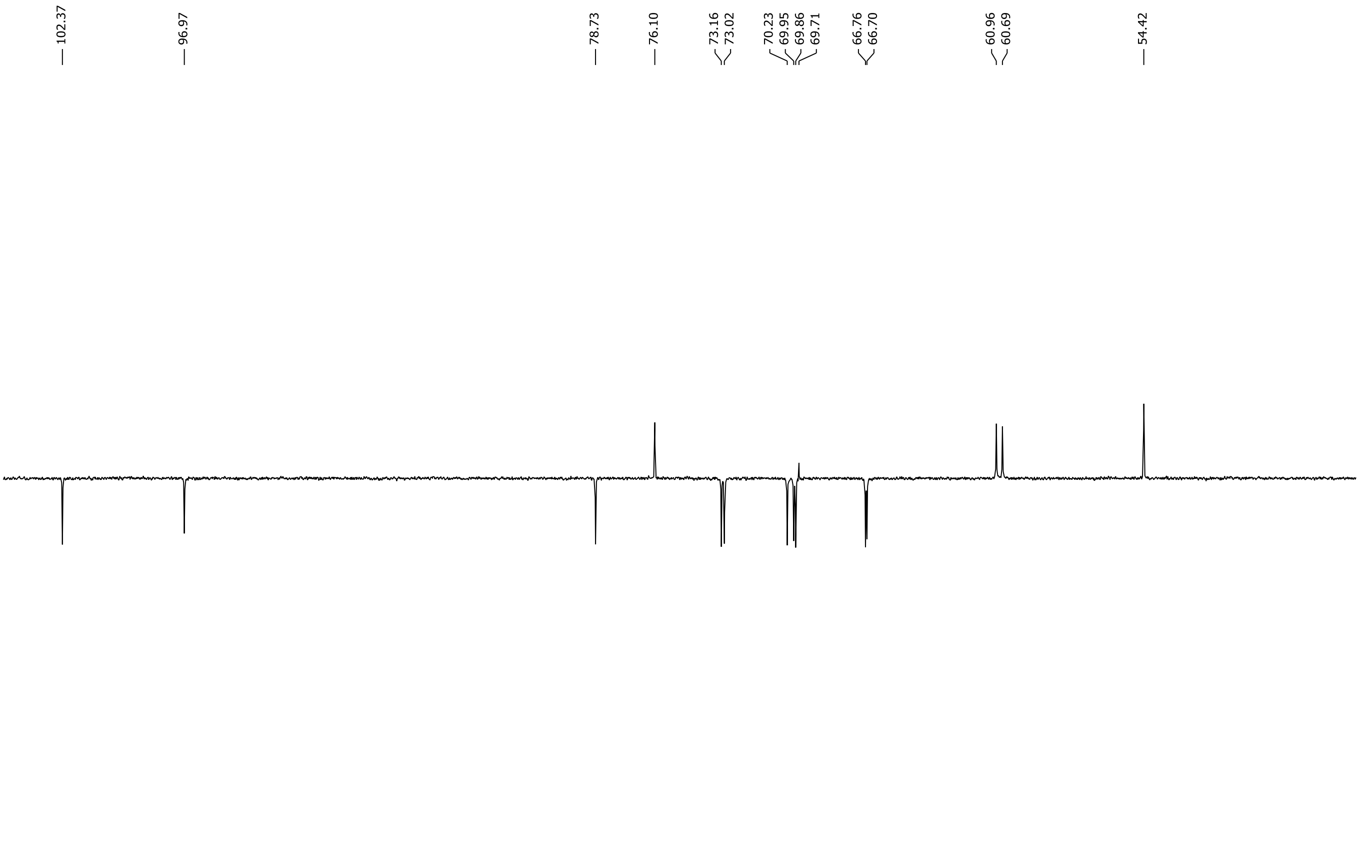

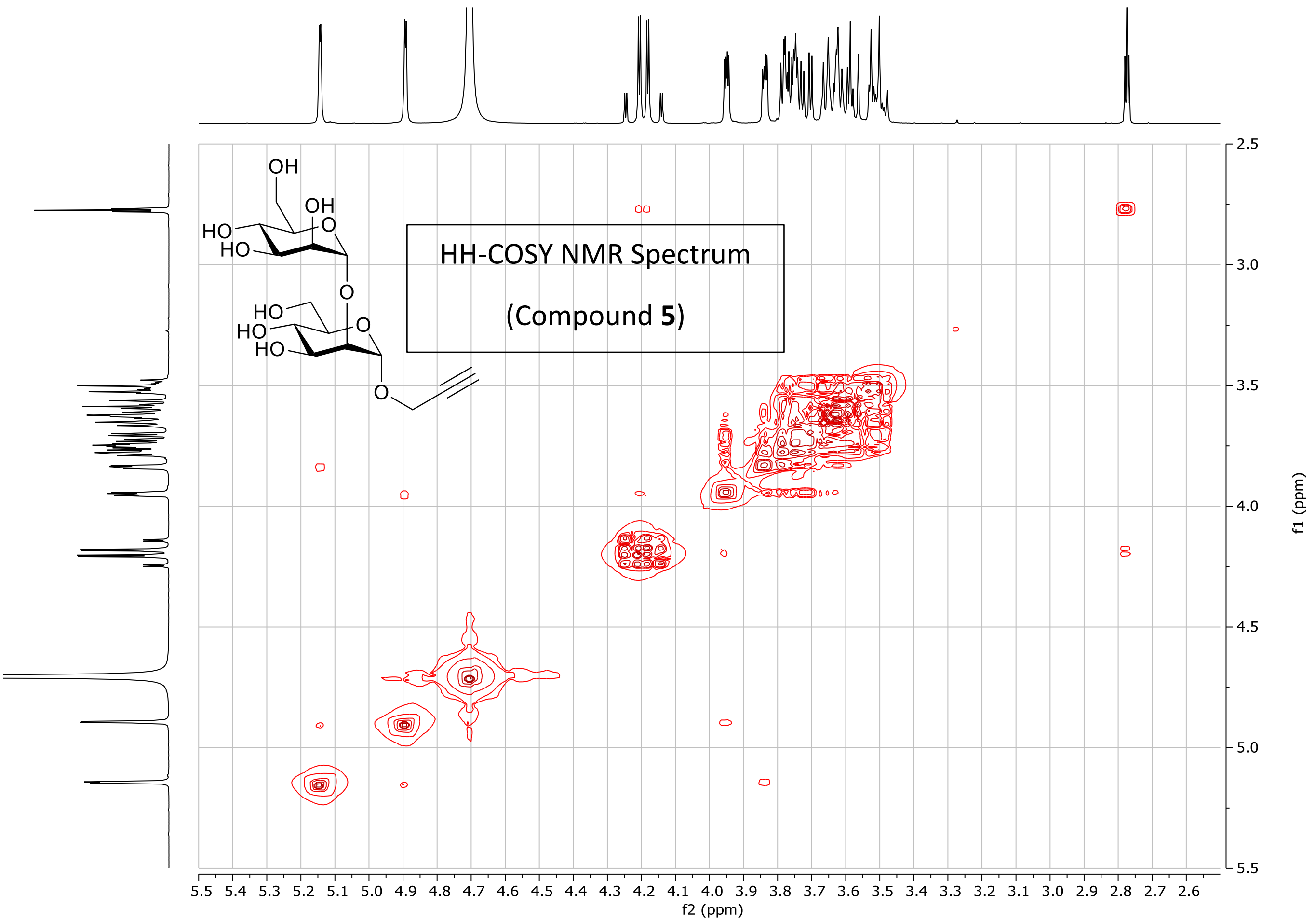
HH-COSY NMR Spectrum
(Compound **5**)
OH
OH
HO
HO
O
O
HO
HO
O
HO
O
f2 (ppm)
f1 (ppm)

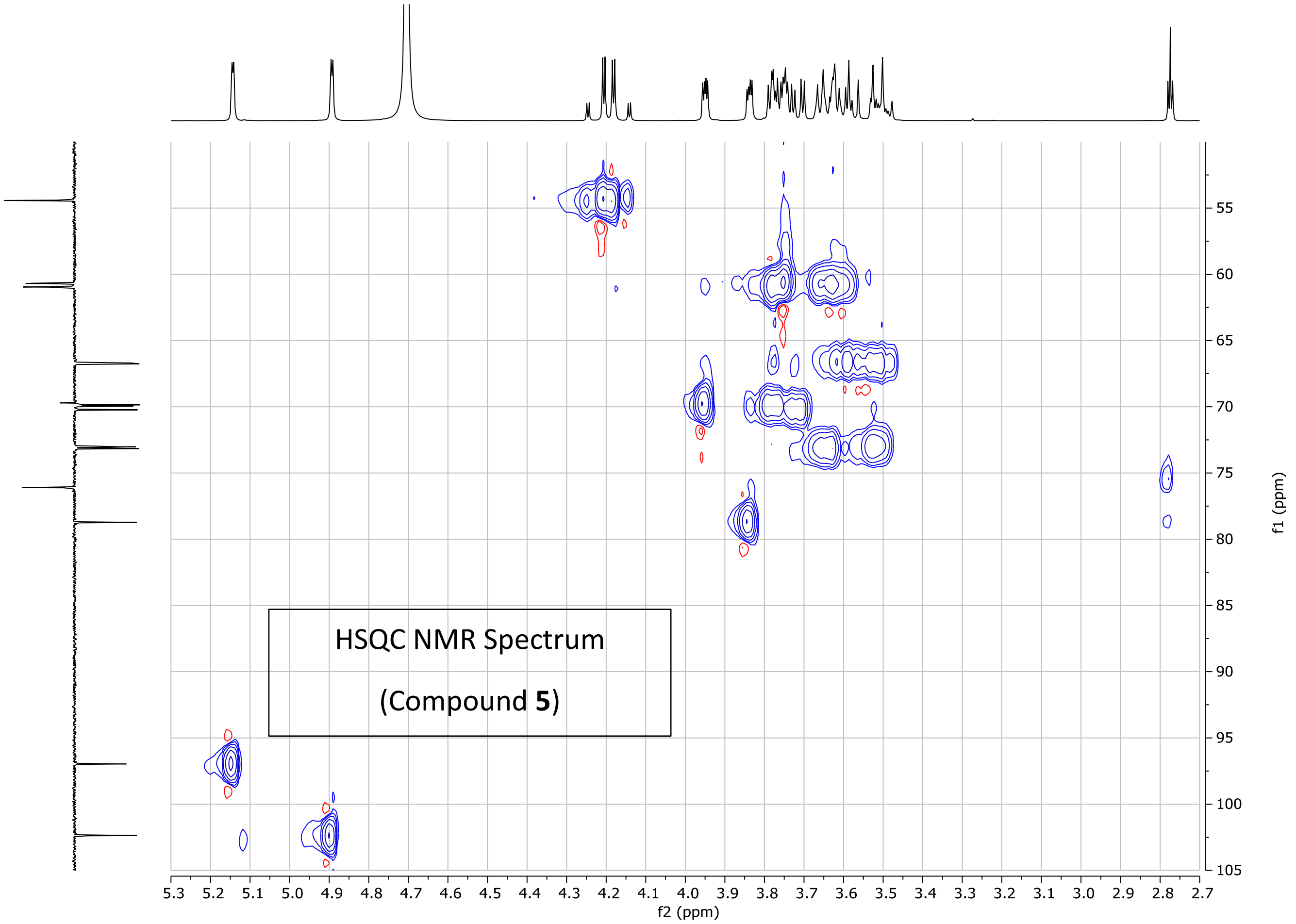
HSQC NMR Spectrum
(Compound **5**)
f2 (ppm)
f1 (ppm)

6

^{1}H NMR Spectrum

(Compound **6**)

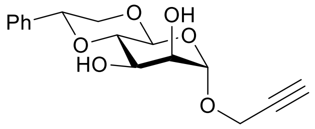

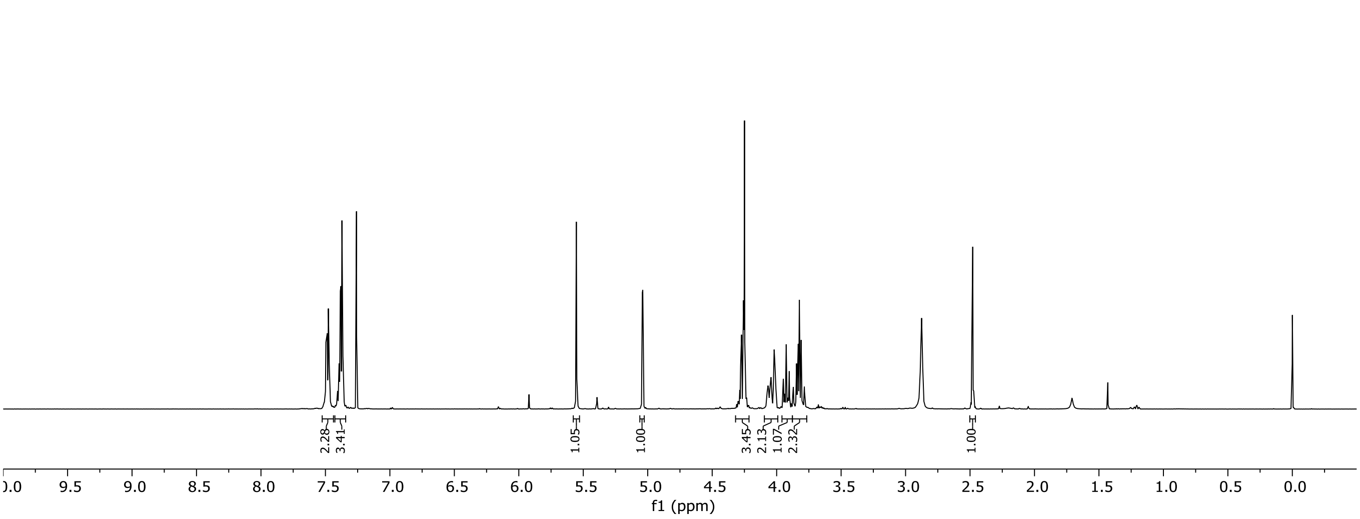

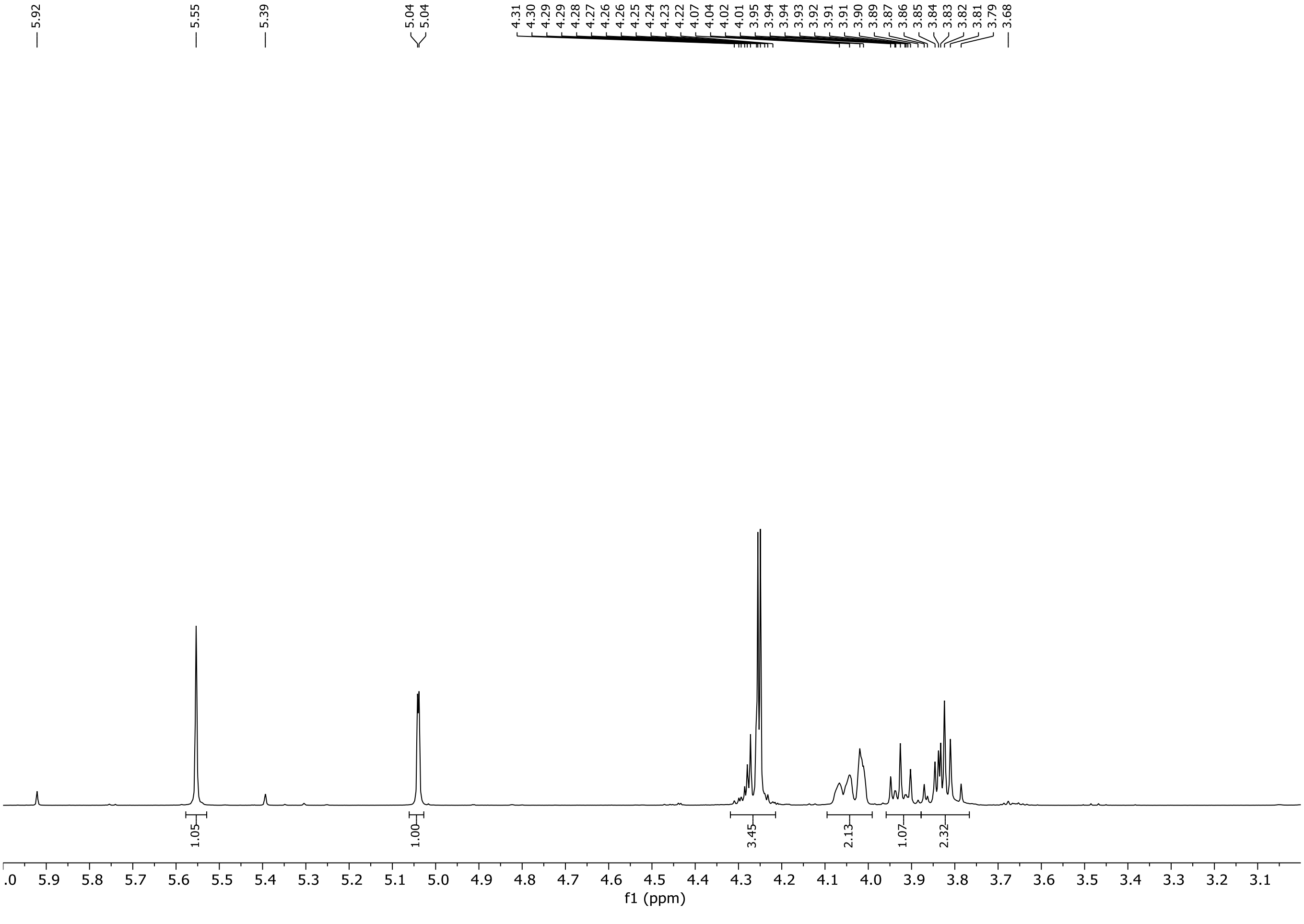

^{13}C APT NMR Spectrum

(Compound **6**)

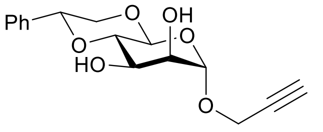

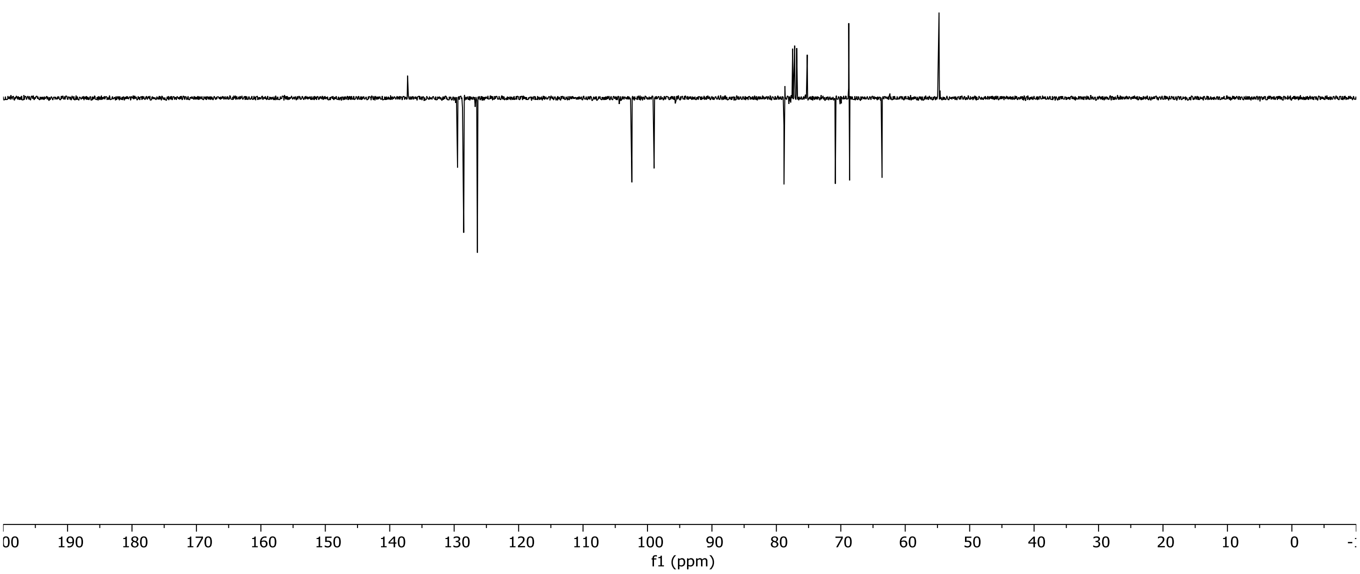

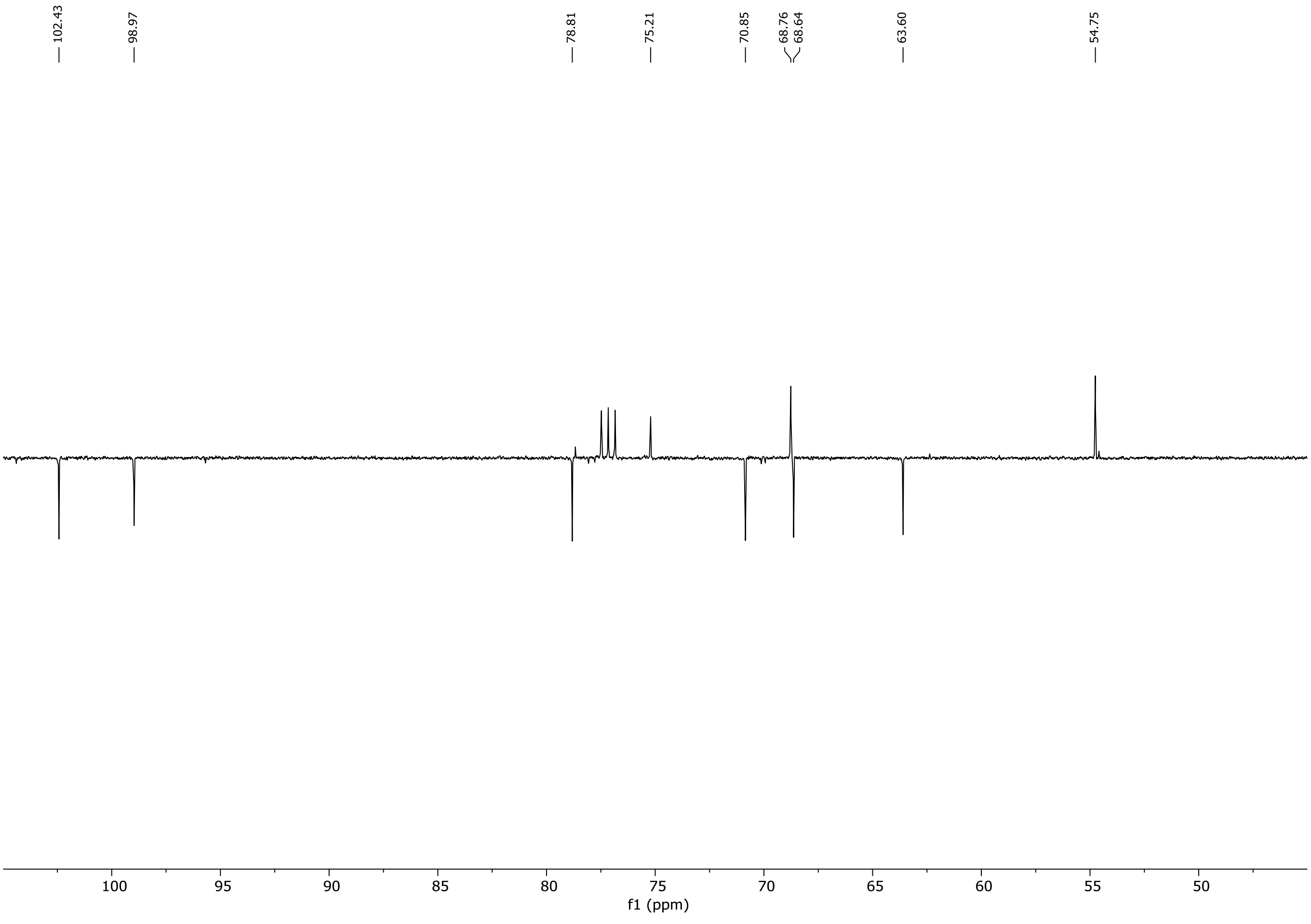

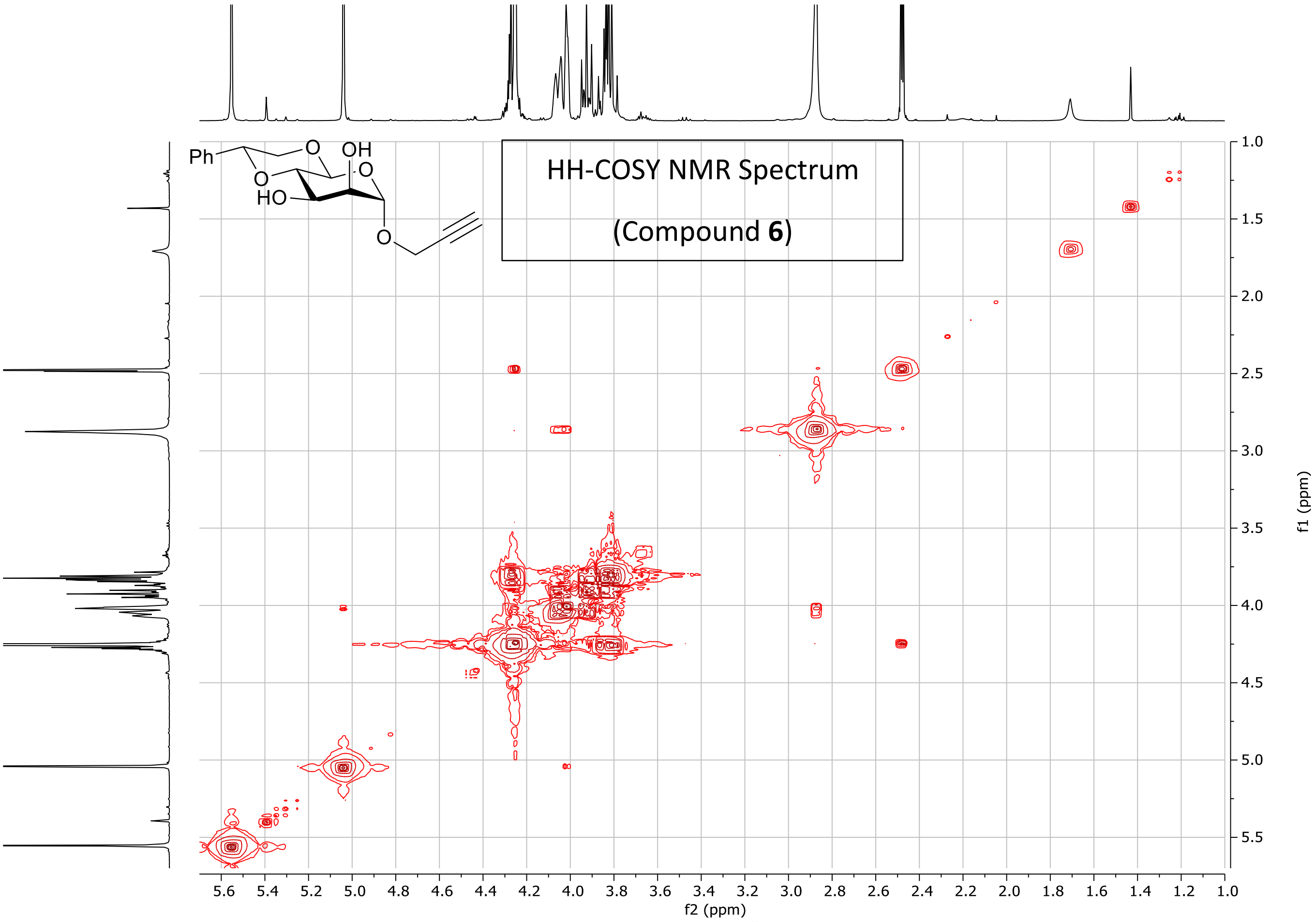
HH-COSY NMR Spectrum
(Compound **6**)
Ph
O
OH
HO
f2 (ppm)
f1 (ppm)

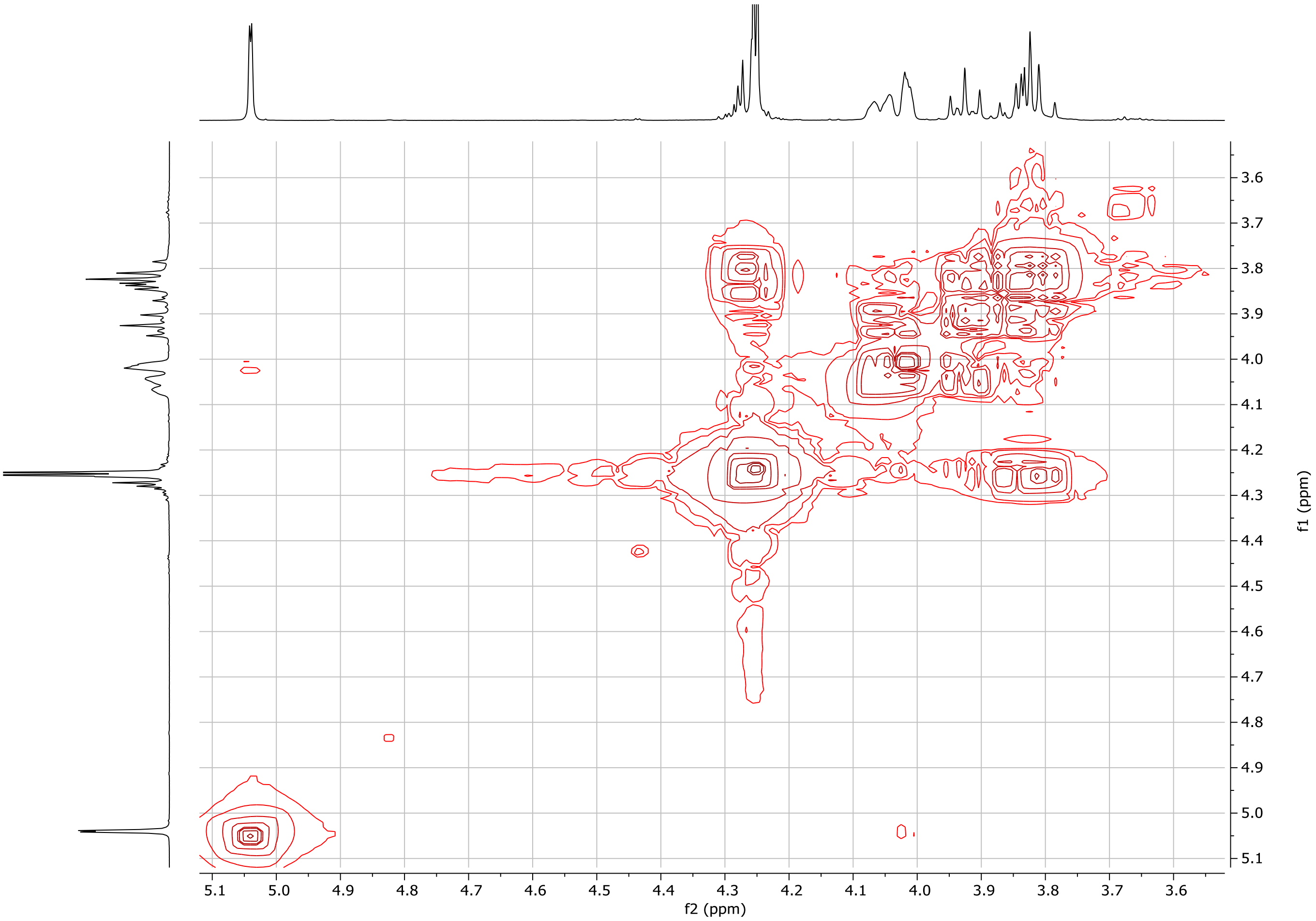
f2 (ppm)
f1 (ppm)

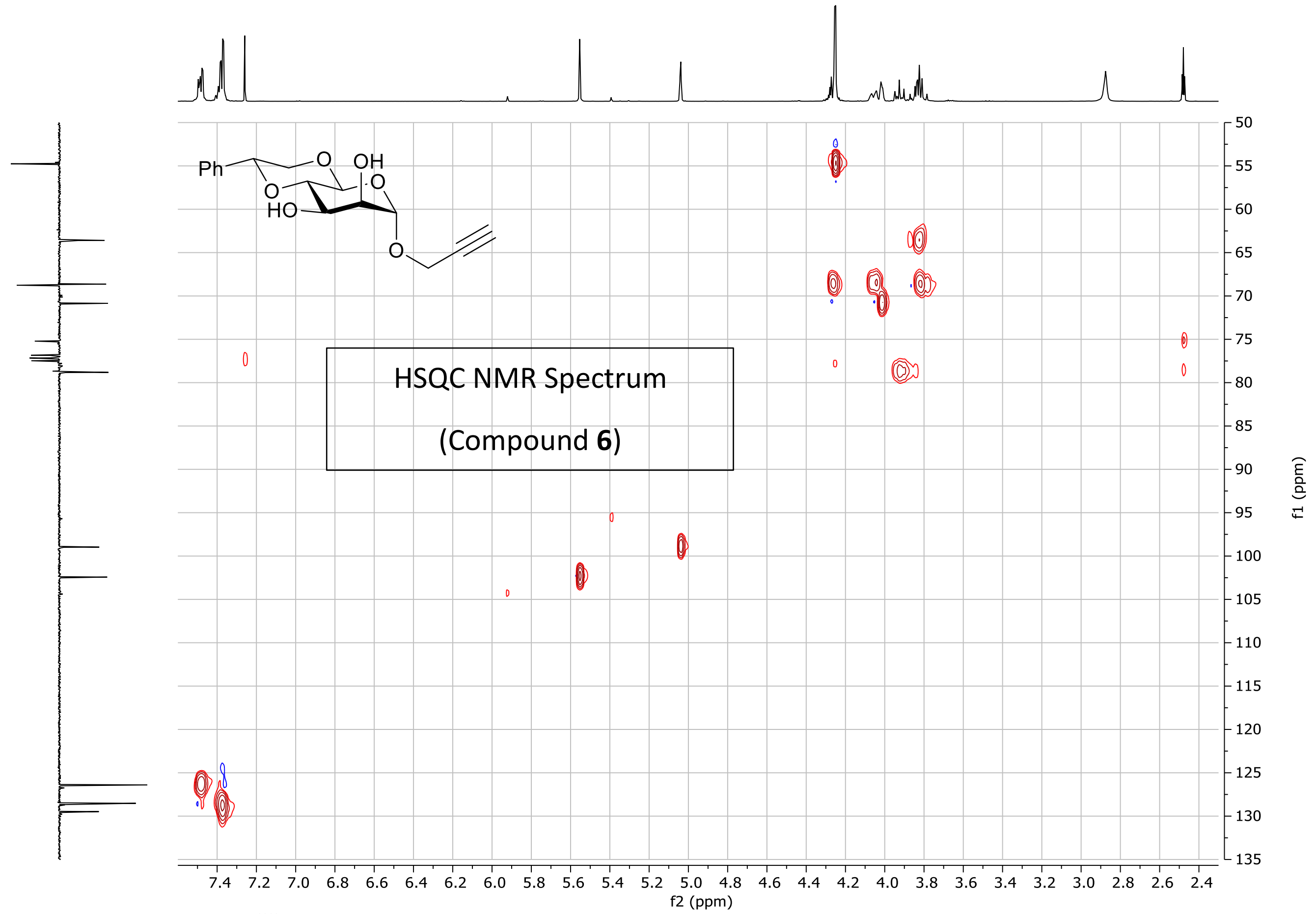
Ph
O
OH
O
O
HO
O
HSQC NMR Spectrum
(Compound **6**)
f2 (ppm)
f1 (ppm)

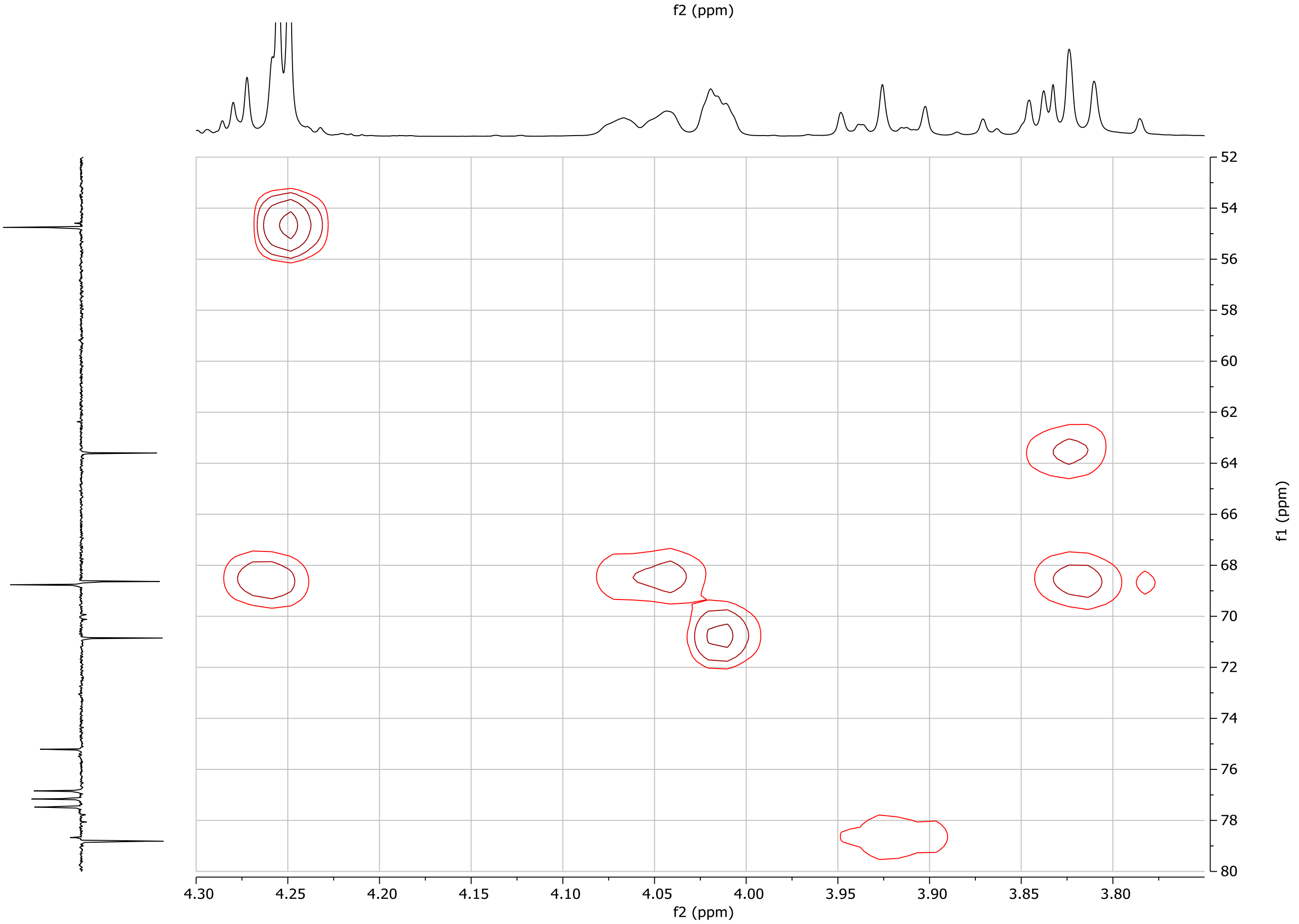
f2 (ppm)
f1 (ppm)

7

^{1}H NMR Spectrum

(Compound **7**)

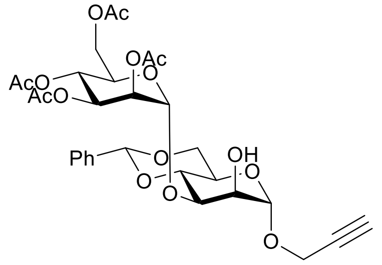

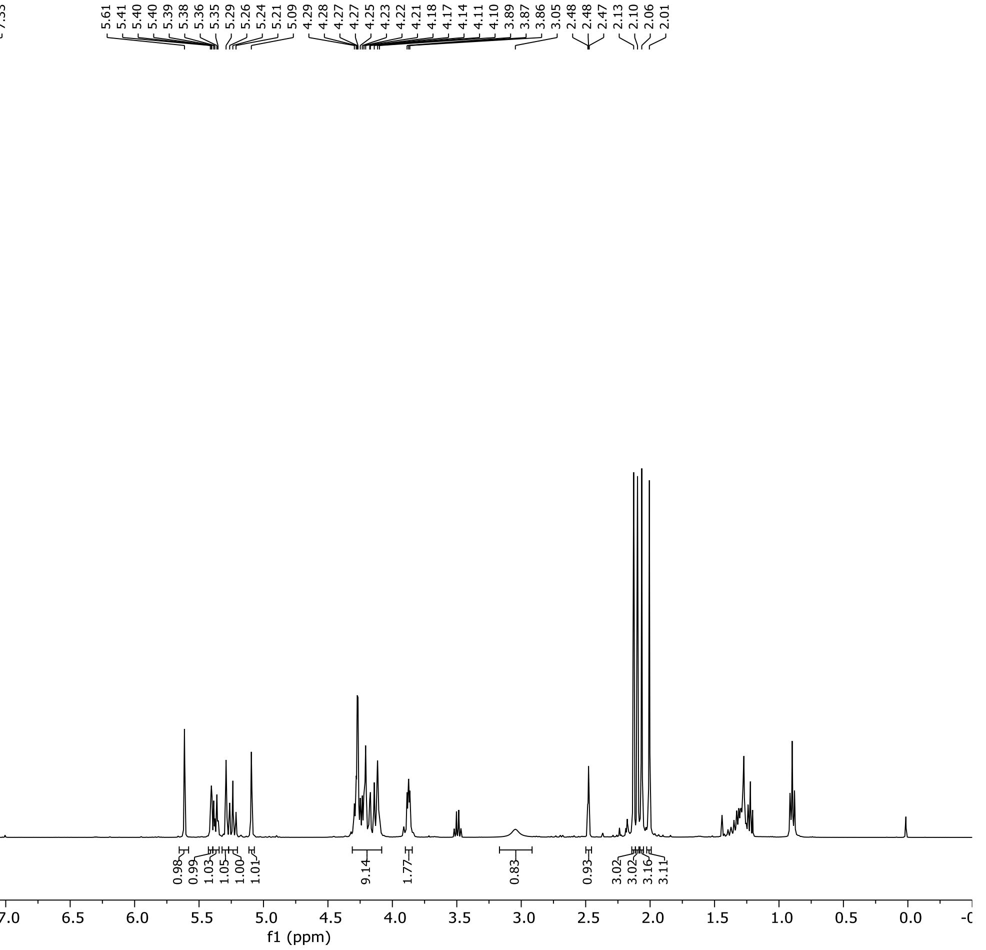

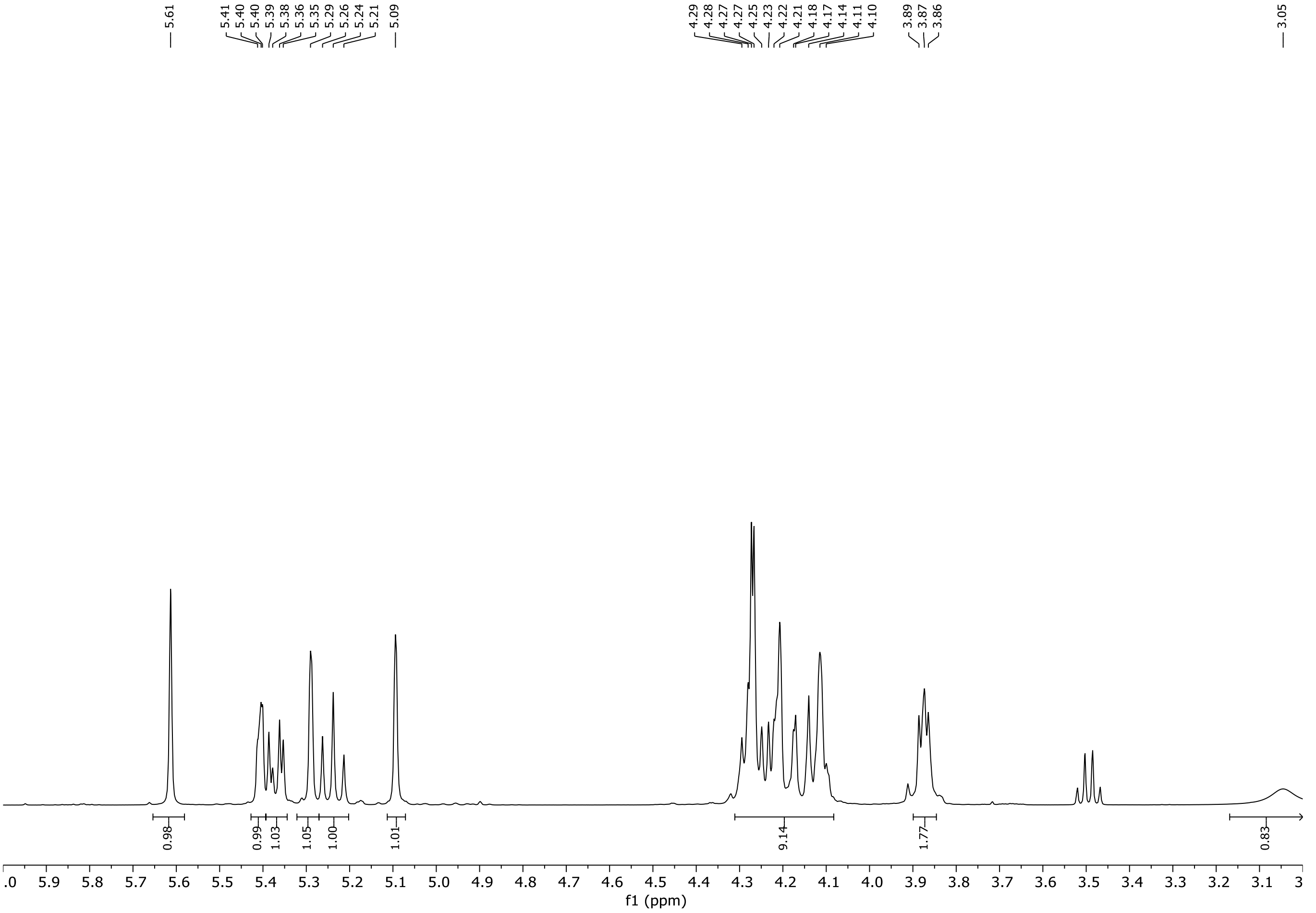

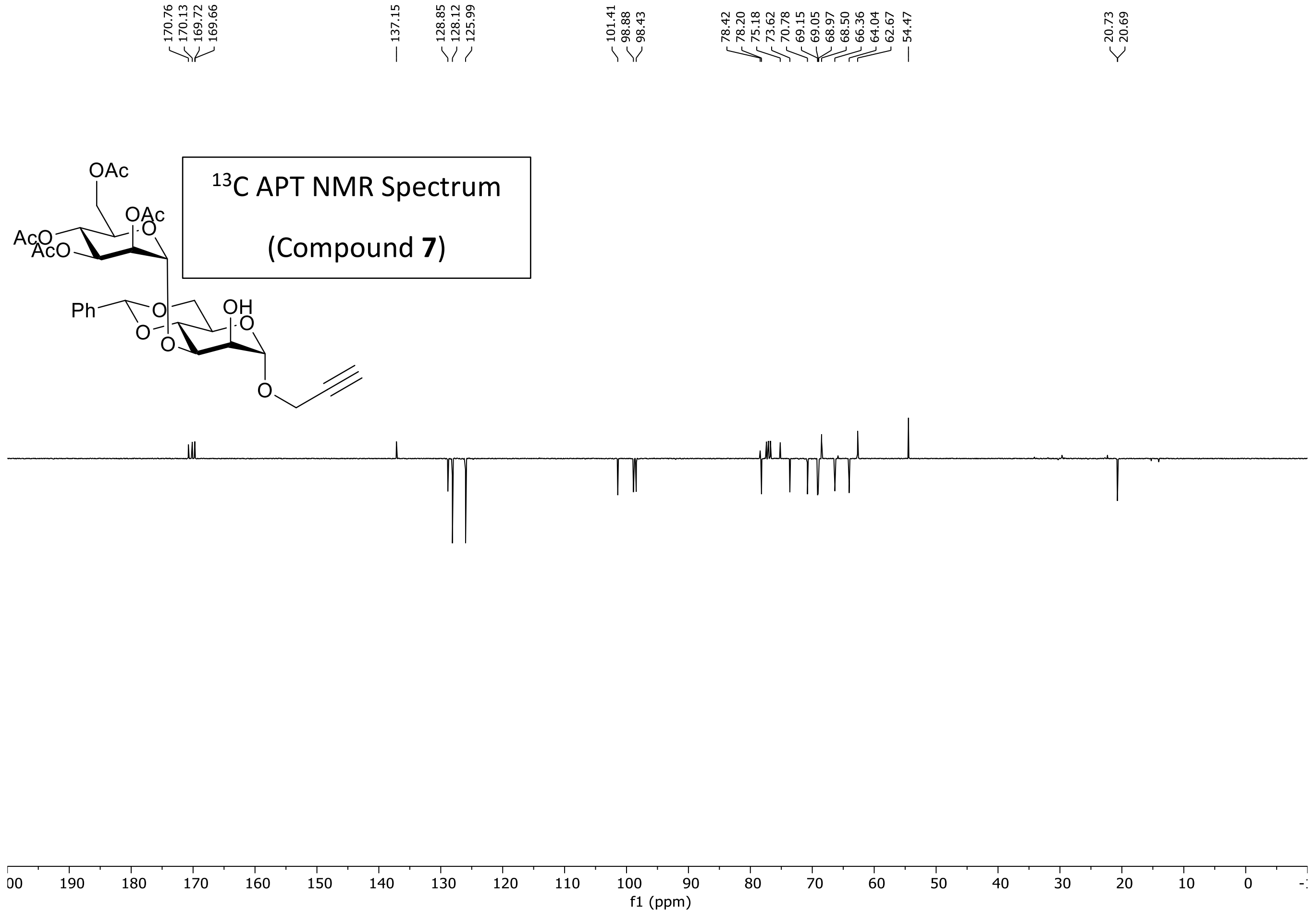
^{13}C APT NMR Spectrum
(Compound **7**)
OAc
OAc
AcO
AcO
Ph
O
OH
O
170.76
170.13
169.72
169.66
137.15
128.85
128.12
125.99
101.41
98.88
98.43
78.42
78.20
75.18
73.62
70.78
69.15
69.05
68.97
68.50
66.36
64.04
62.67
54.47
20.73
20.69
f1 (ppm)

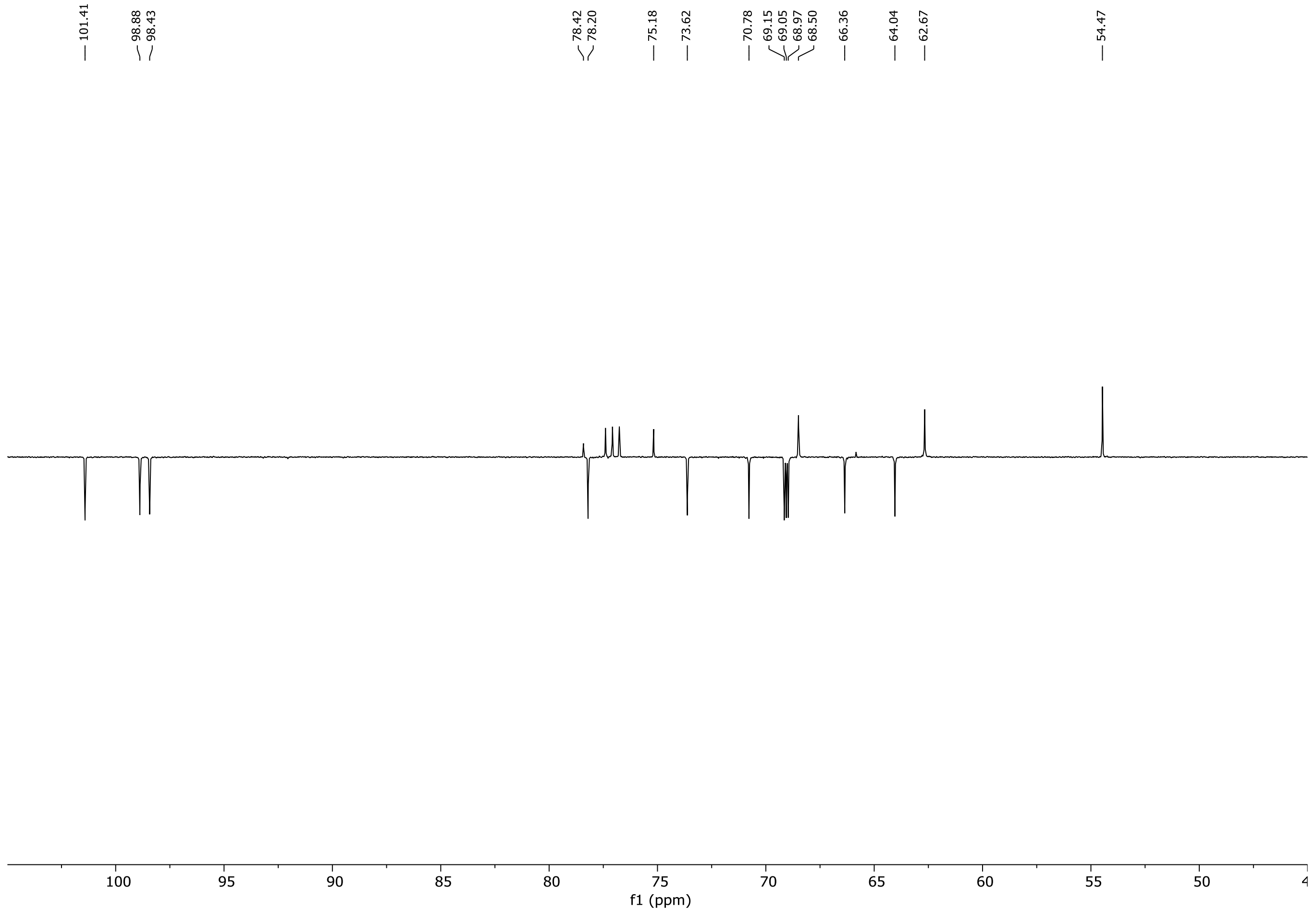
101.41
98.88
98.43
78.42
78.20
75.18
73.62
70.78
69.15
69.05
68.97
68.50
66.36
64.04
62.67
54.47
f1 (ppm)

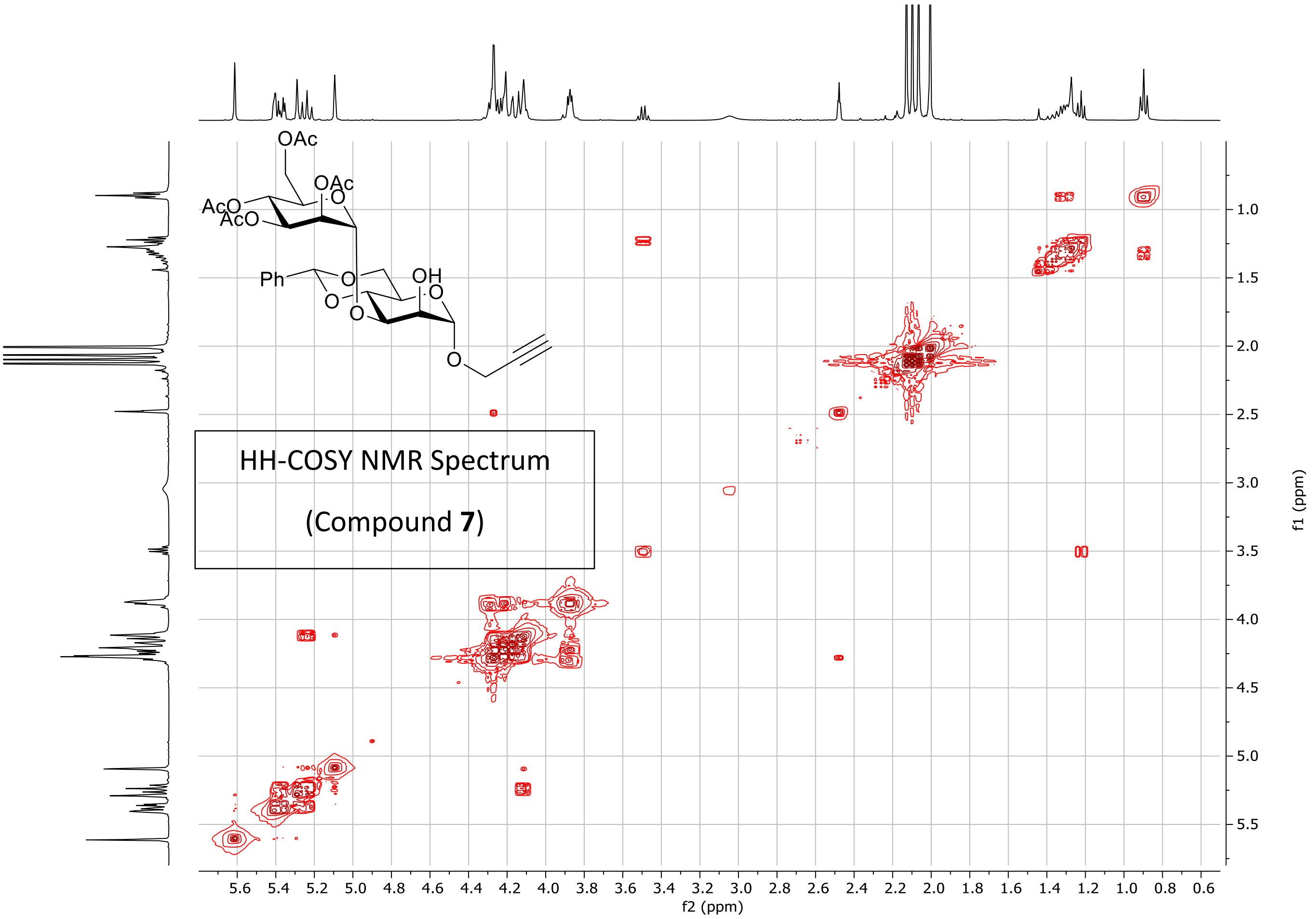
OAc
OAc
AcO
AcO
O
Ph
O
O
O
OH
O
O
HH-COSY NMR Spectrum
(Compound 7)
f2 (ppm)
f1 (ppm)

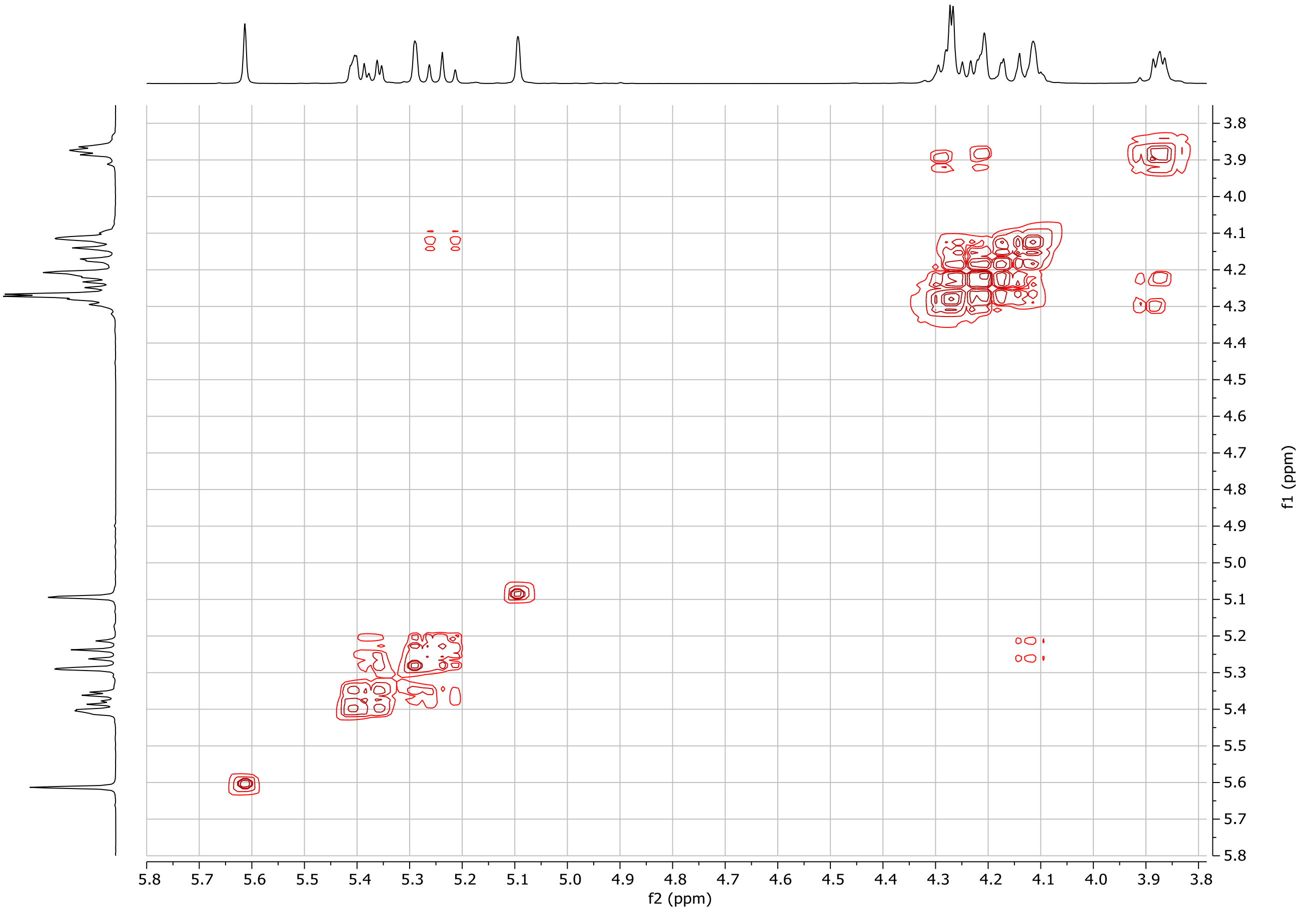
f2 (ppm)
f1 (ppm)

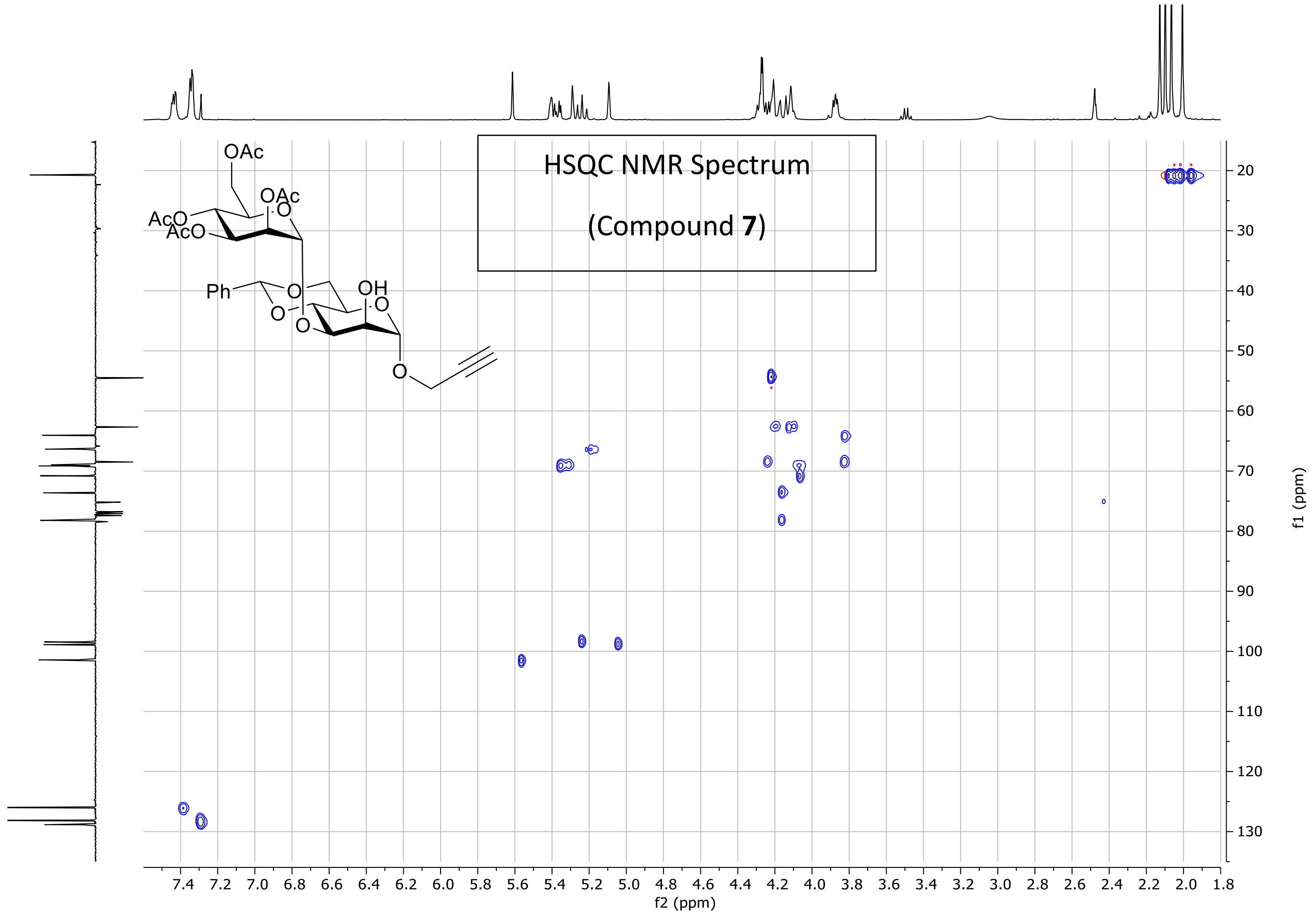
HSQC NMR Spectrum
(Compound 7)
OAc
OAc
AcO
AcO
O
Ph
O
O
O
OH
O
O
f2 (ppm)
f1 (ppm)

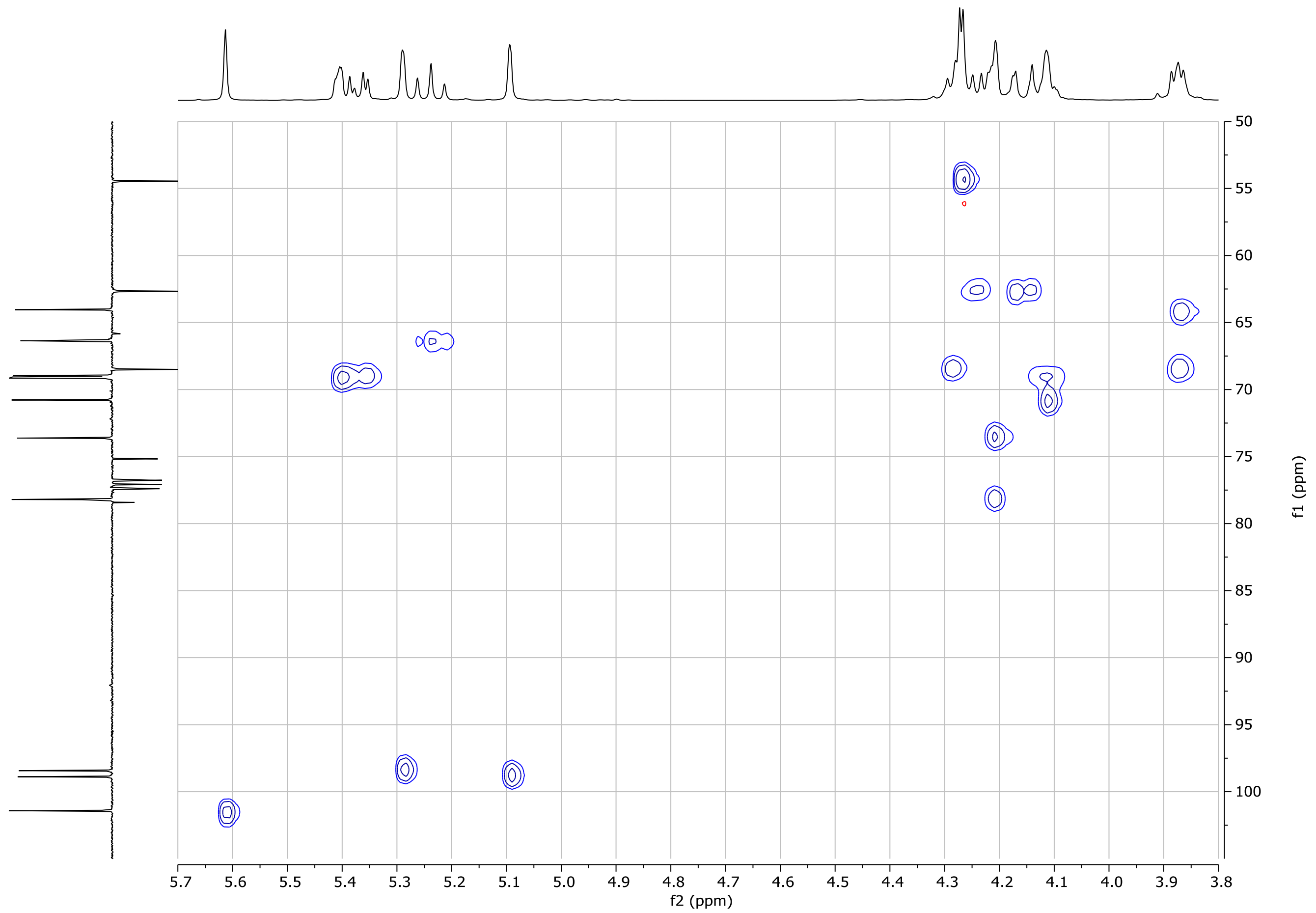
f2 (ppm)
f1 (ppm)

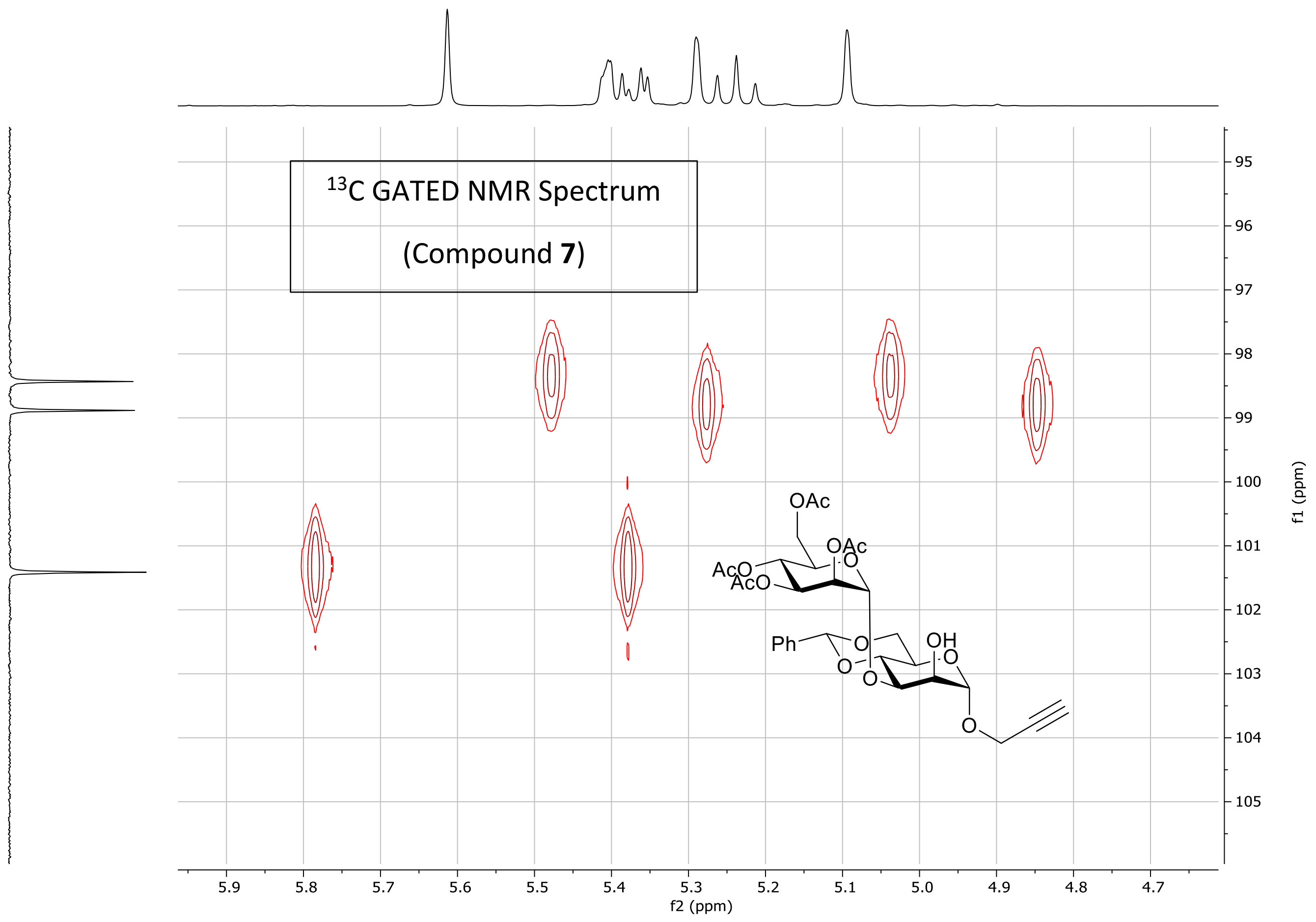

13C GATED NMR Spectrum
(Compound 7)
OAc
OAc
AcO
AcO
O
Ph
O
O
O
OH
O
O
f1 (ppm)
f2 (ppm)

S5

^{1}H NMR Spectrum

(Compound **S5**)

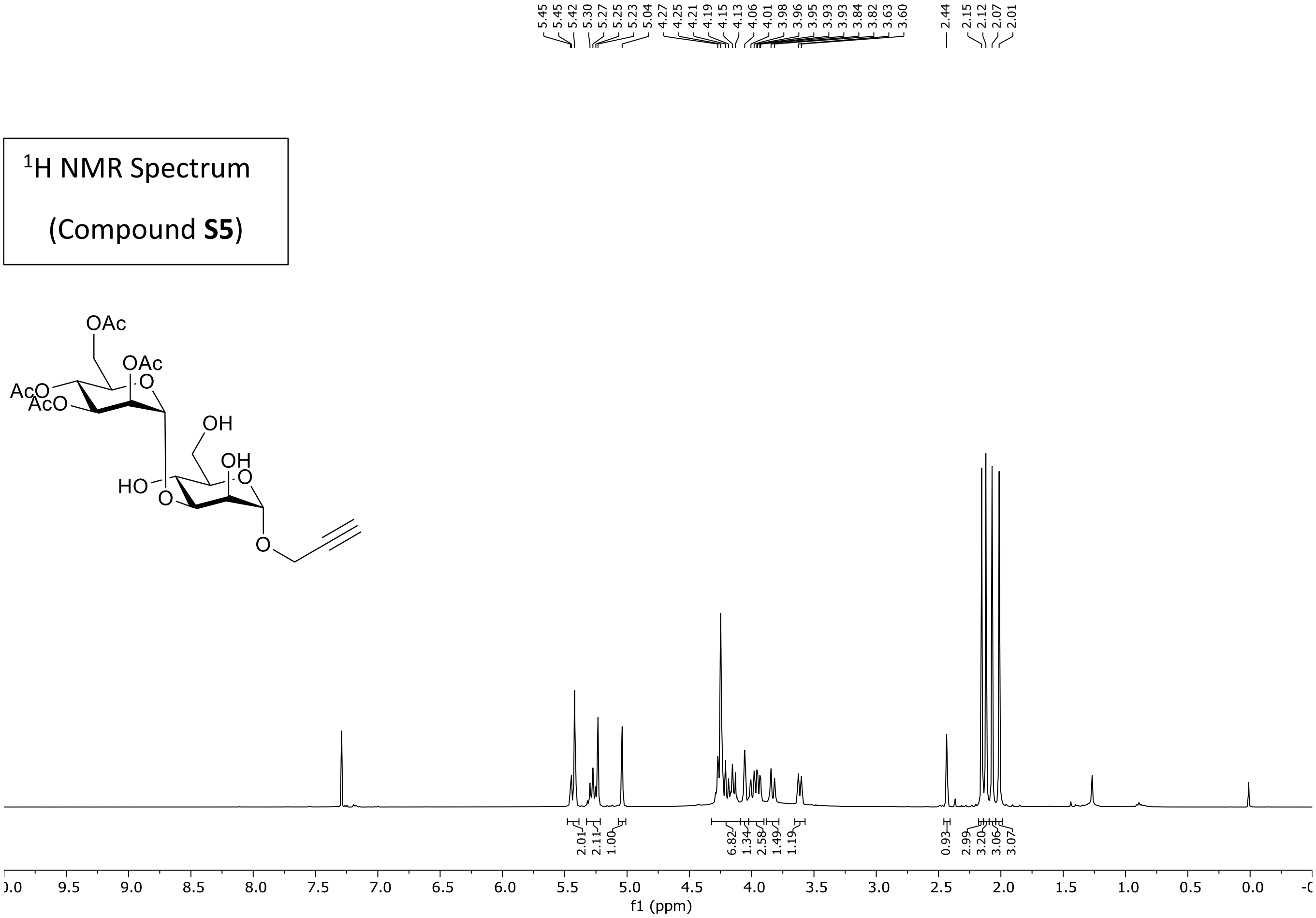

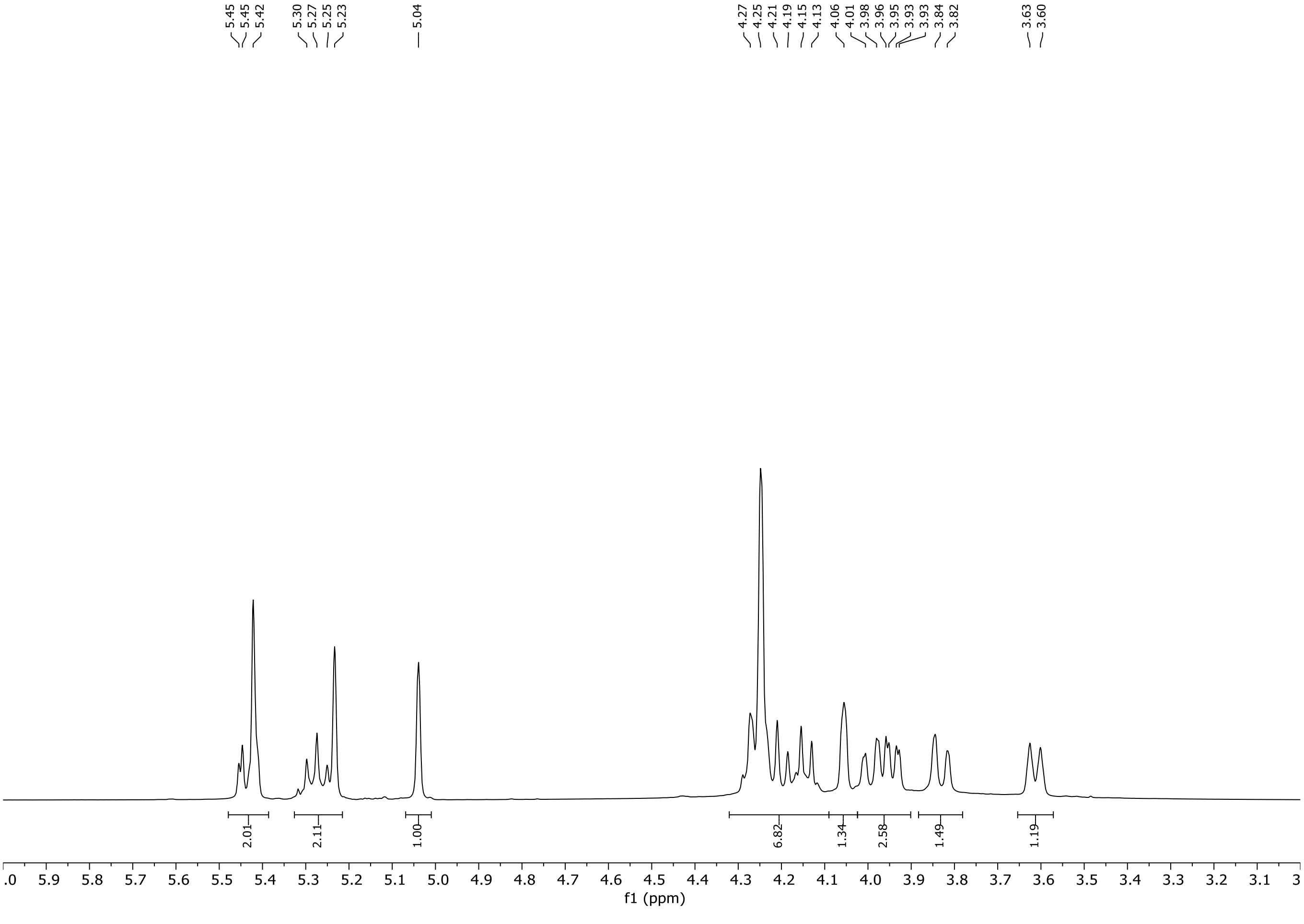

^{13}C APT NMR Spectrum

(Compound **S5**)

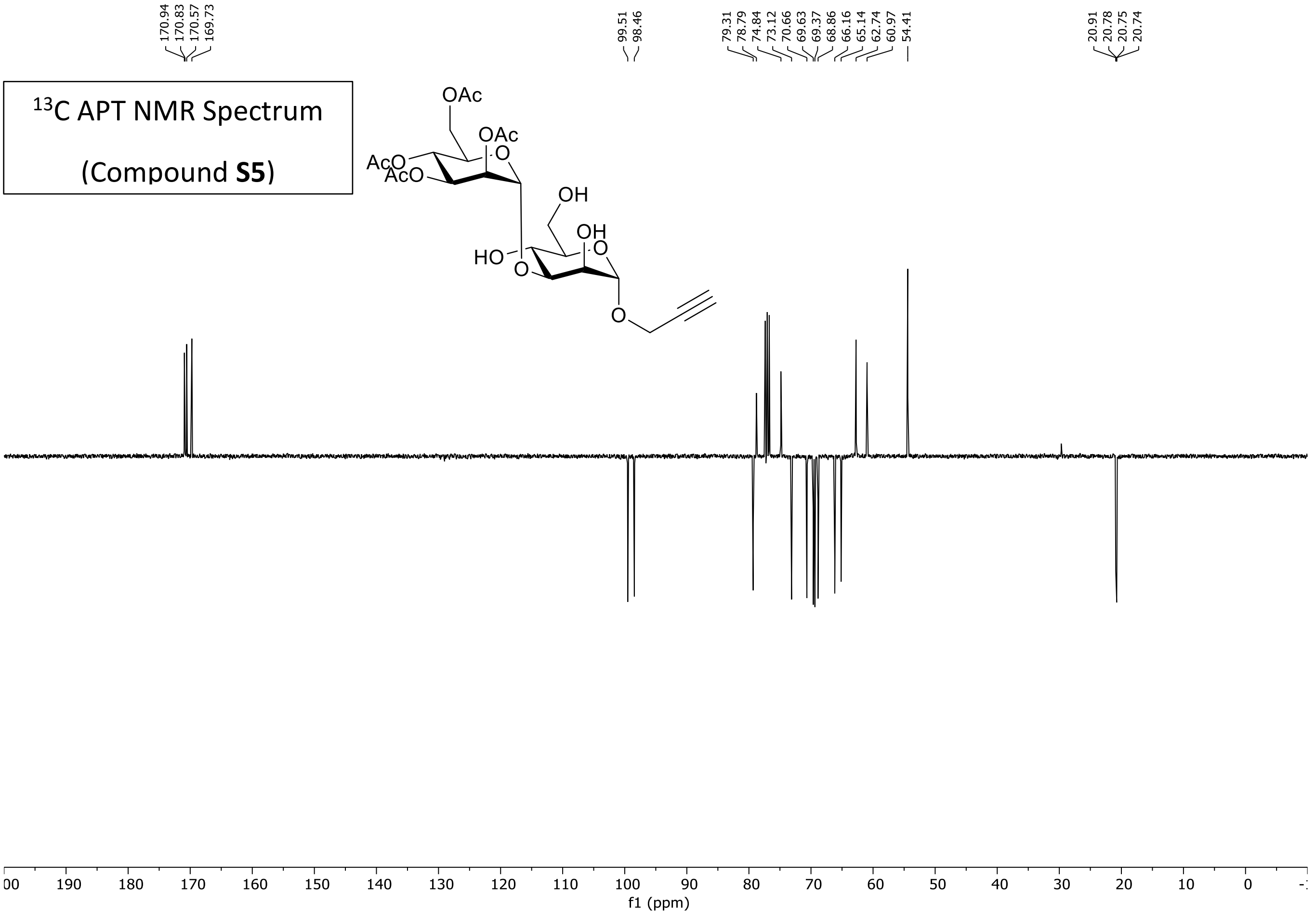

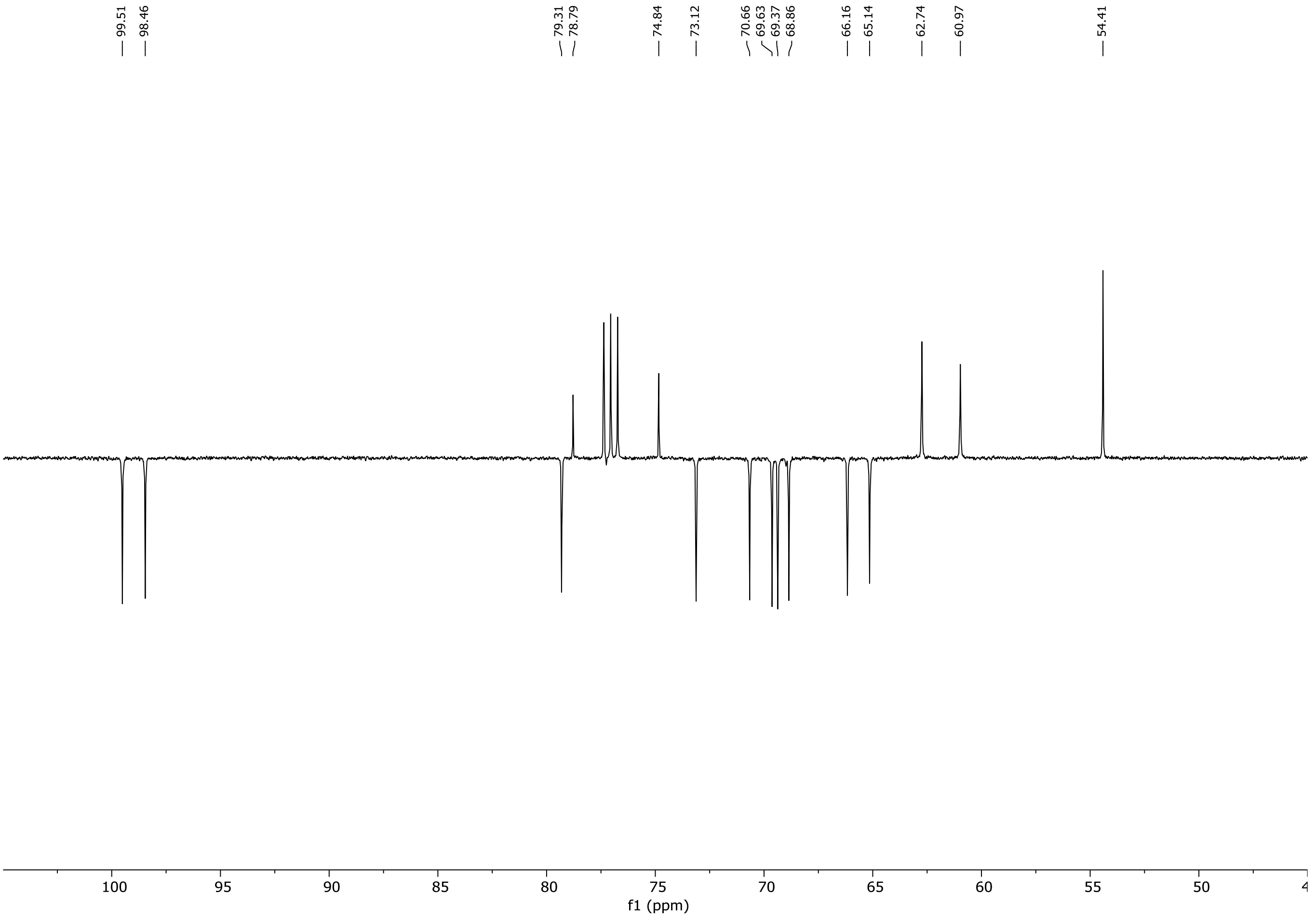

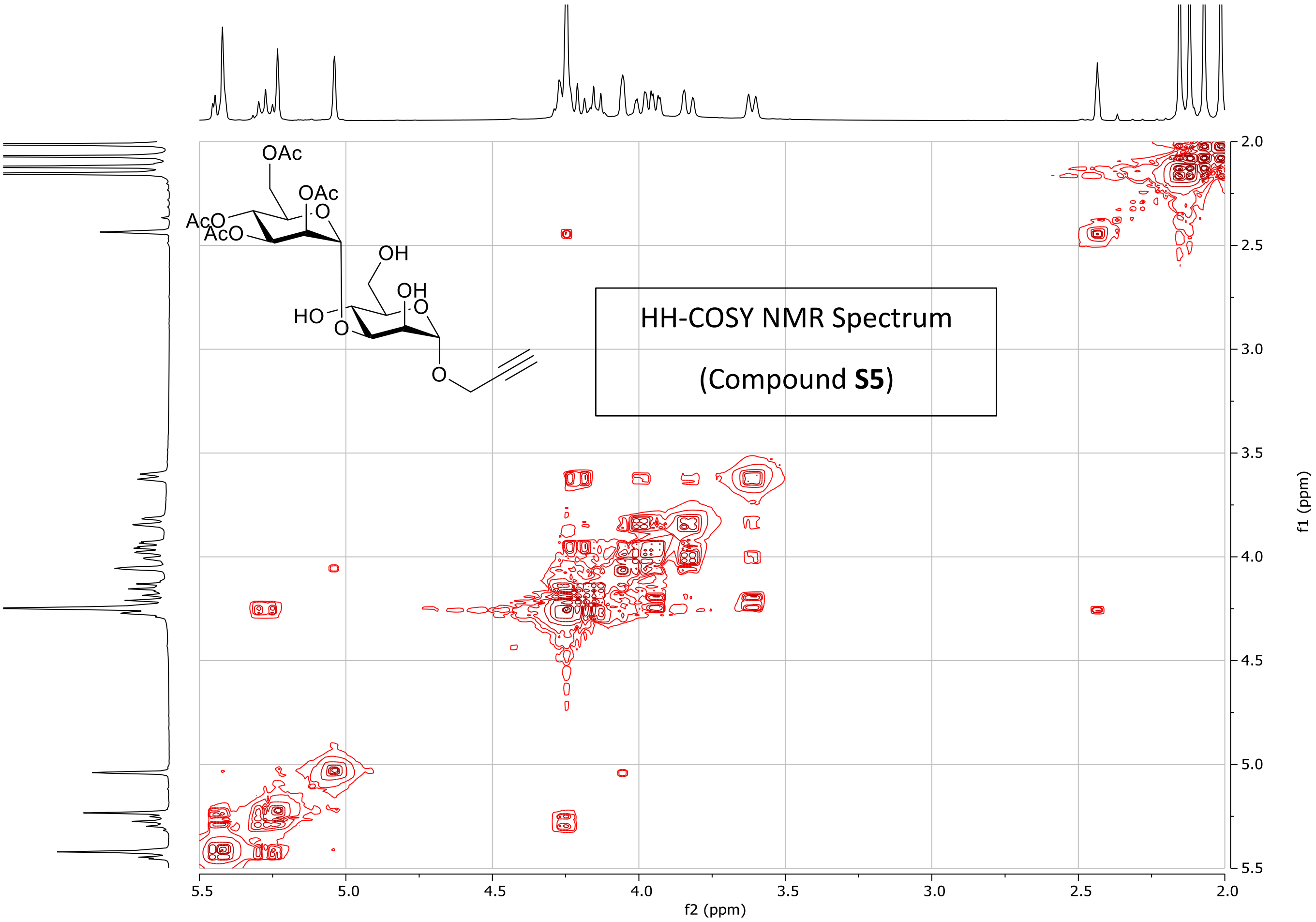
HH-COSY NMR Spectrum
(Compound **S5**)
OAc
OAc
AcO
AcO
O
OH
OH
HO
O
O
O
f2 (ppm)
f1 (ppm)

8

^{1}H NMR Spectrum

(Compound **8**)

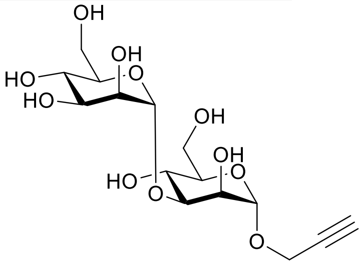

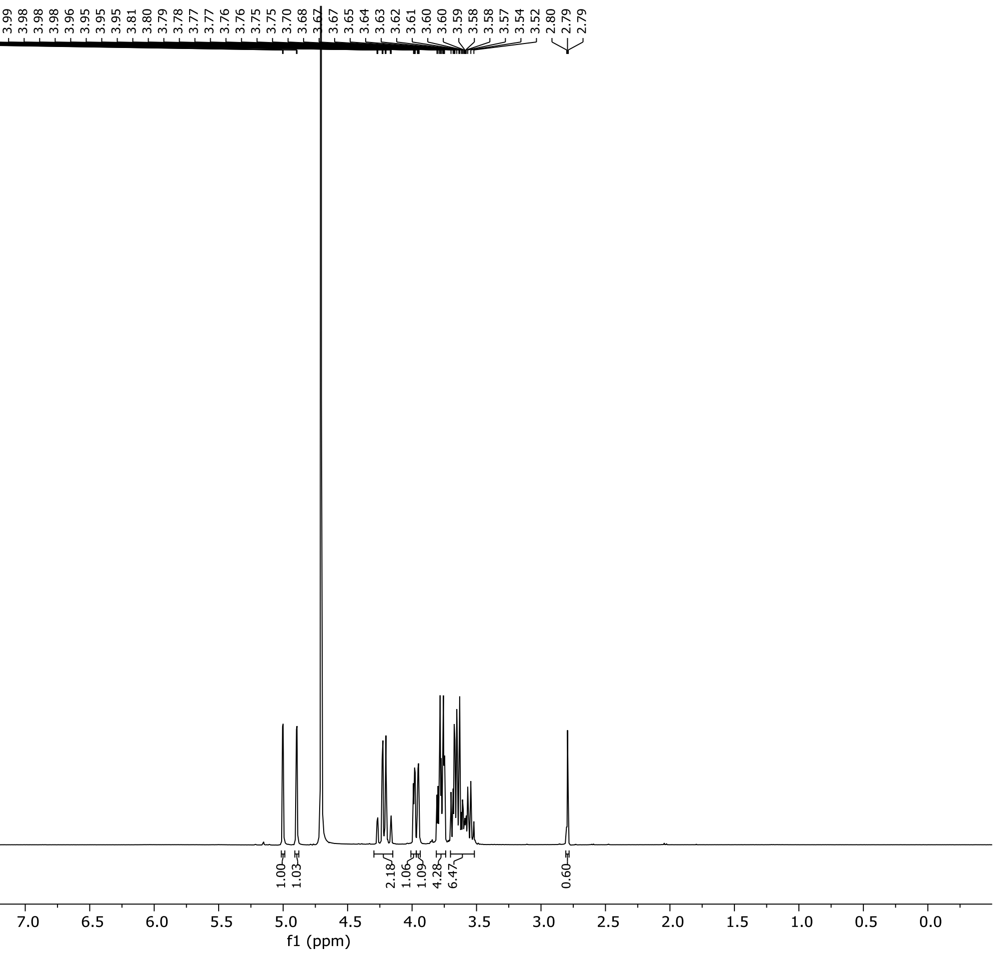

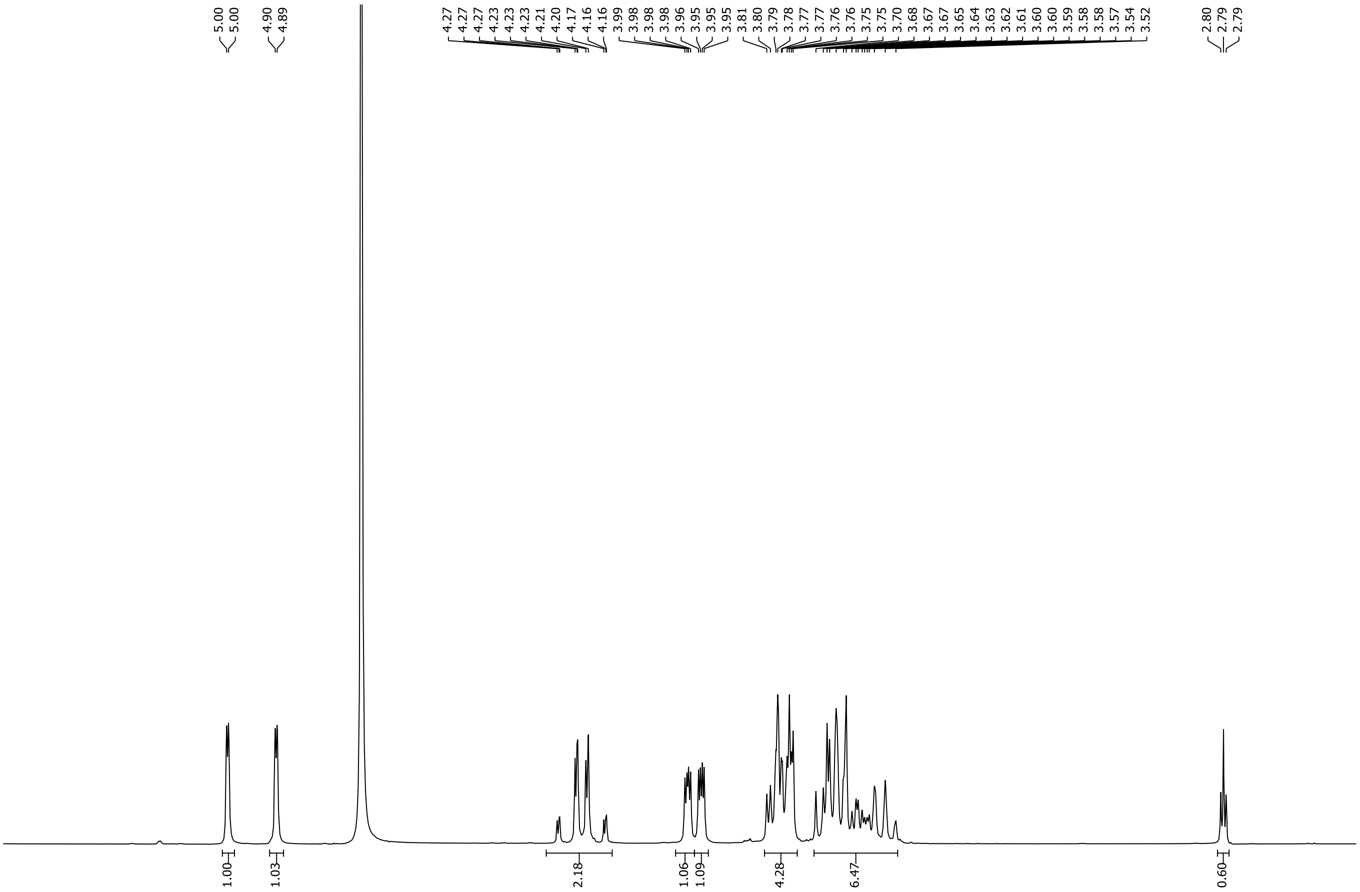

^{13}C APT NMR Spectrum

(Compound **8**)

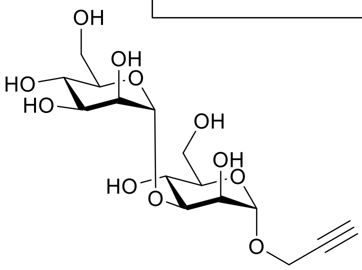

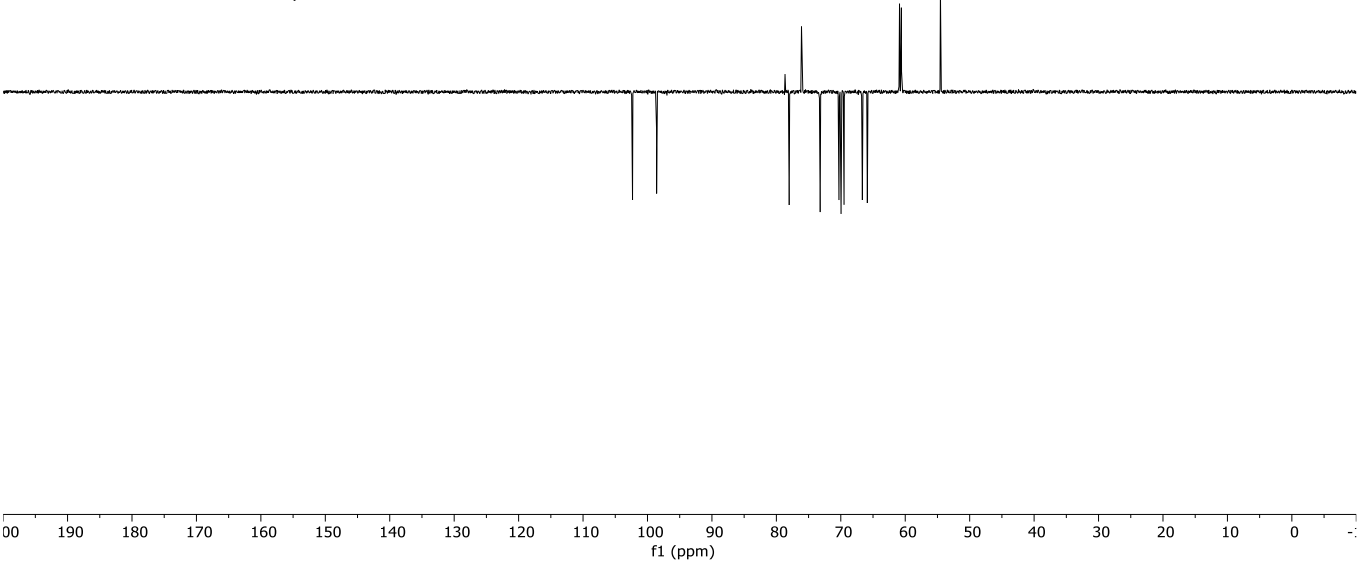

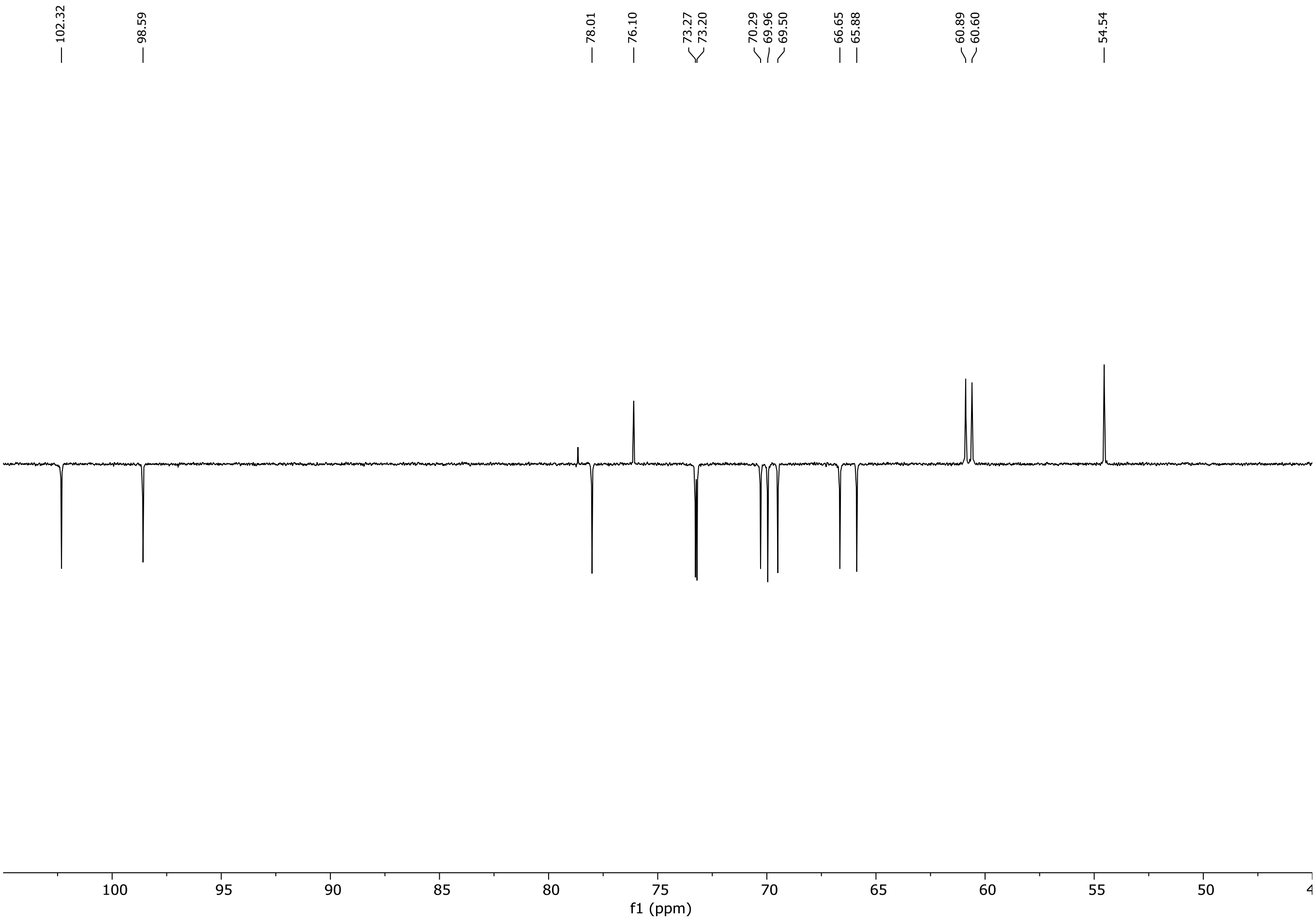

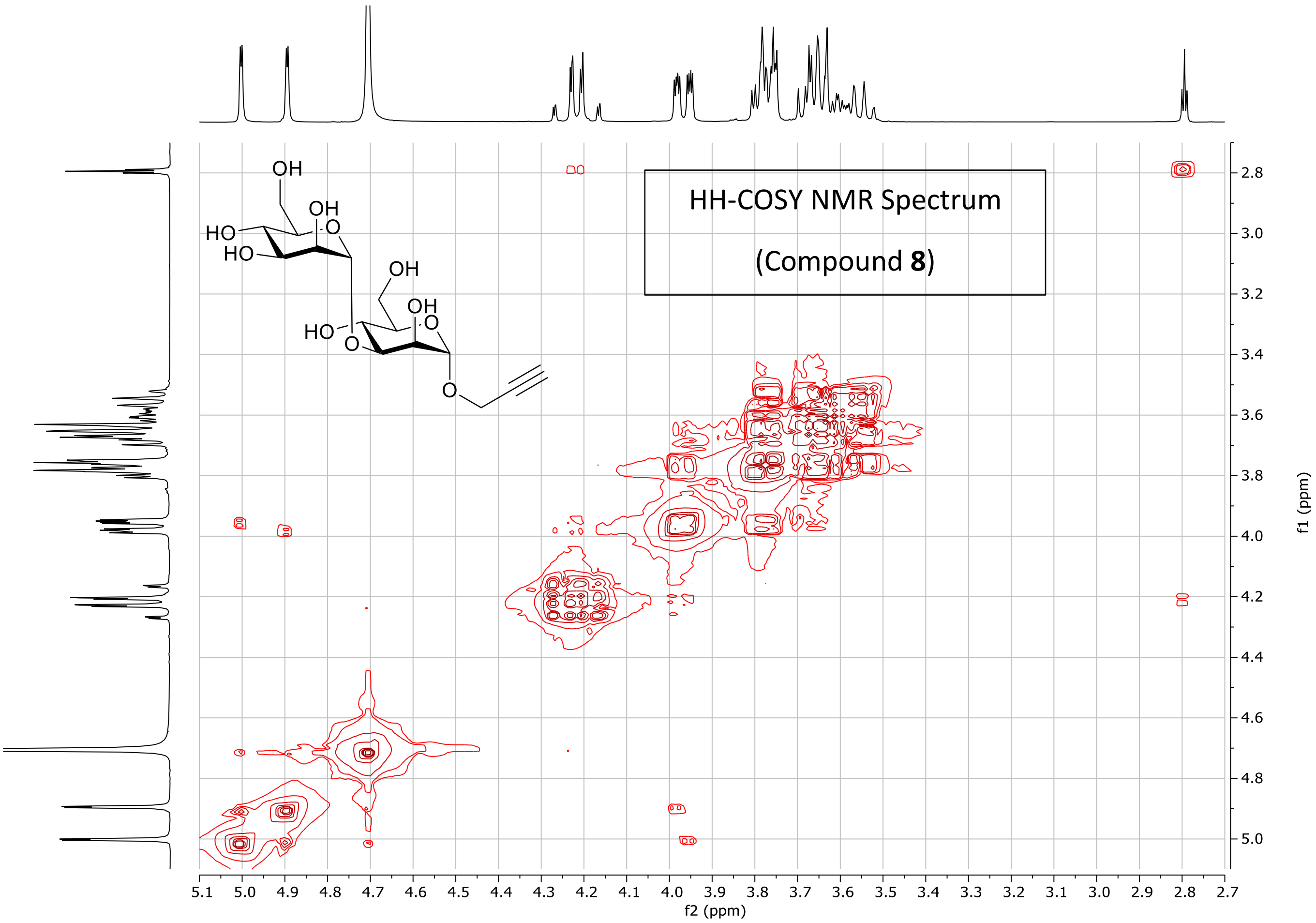
HH-COSY NMR Spectrum
(Compound **8**)
OH
OH
HO
HO
O
OH
OH
HO
O
O
O
5.1 5.0 4.9 4.8 4.7 4.6 4.5 4.4 4.3 4.2 4.1 4.0 3.9 3.8 3.7 3.6 3.5 3.4 3.3 3.2 3.1 3.0 2.9 2.8 2.7
f2 (ppm)
2.8 3.0 3.2 3.4 3.6 3.8 4.0 4.2 4.4 4.6 4.8 5.0
f1 (ppm)

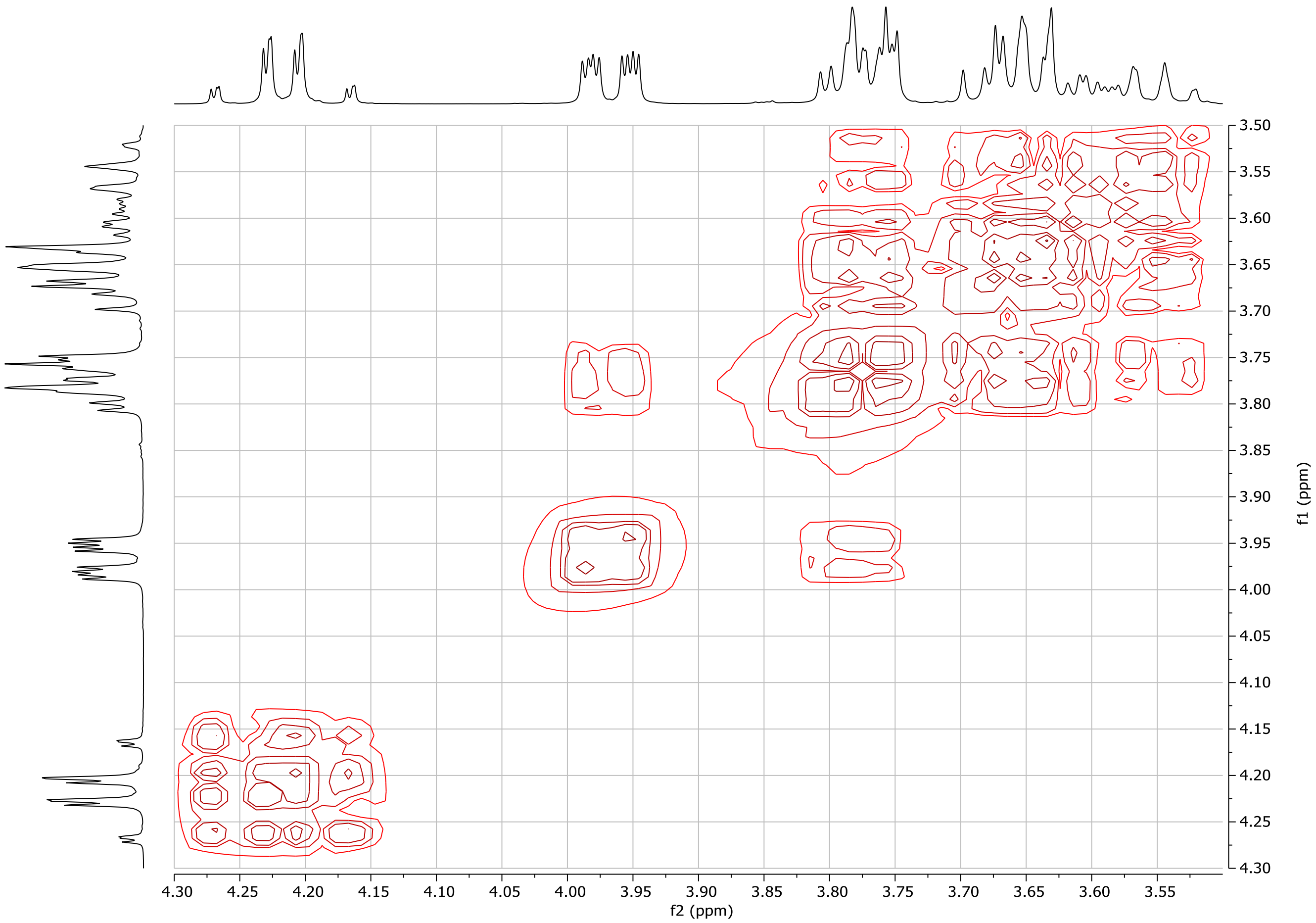
4.30 4.25 4.20 4.15 4.10 4.05 4.00 3.95 3.90 3.85 3.80 3.75 3.70 3.65 3.60 3.55
f2 (ppm)
3.50 3.55 3.60 3.65 3.70 3.75 3.80 3.85 3.90 3.95 4.00 4.05 4.10 4.15 4.20 4.25 4.30
f1 (ppm)

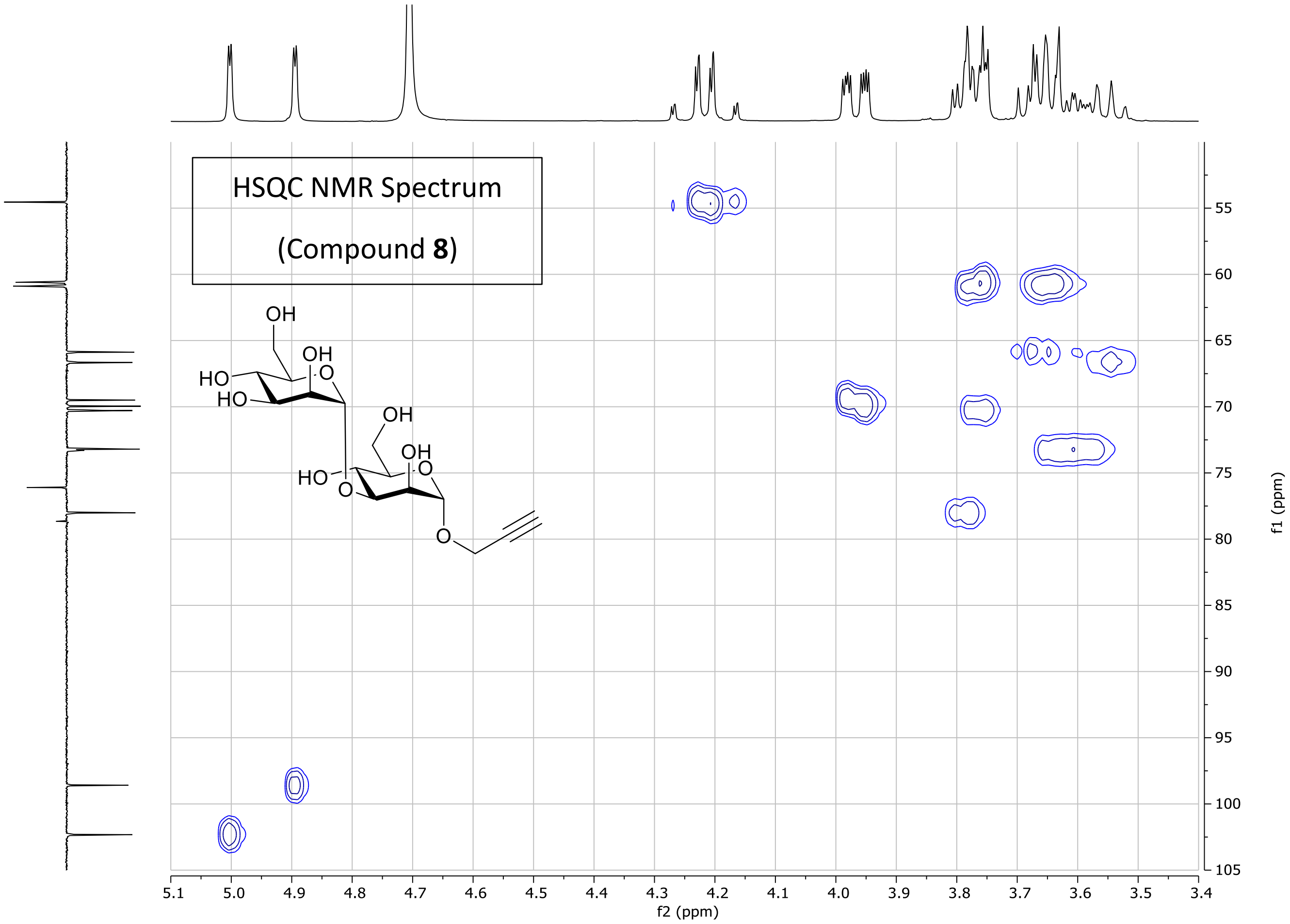
HSQC NMR Spectrum
(Compound **8**)
OH
OH
HO
HO
O
OH
OH
HO
O
O
O
f2 (ppm)
f1 (ppm)
5.1
5.0
4.9
4.8
4.7
4.6
4.5
4.4
4.3
4.2
4.1
4.0
3.9
3.8
3.7
3.6
3.5
3.4
55
60
65
70
75
80
85
90
95
100
105

S6

^{1}H NMR Spectrum

(Compound **S6**)

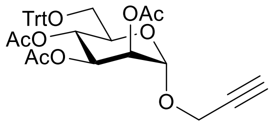

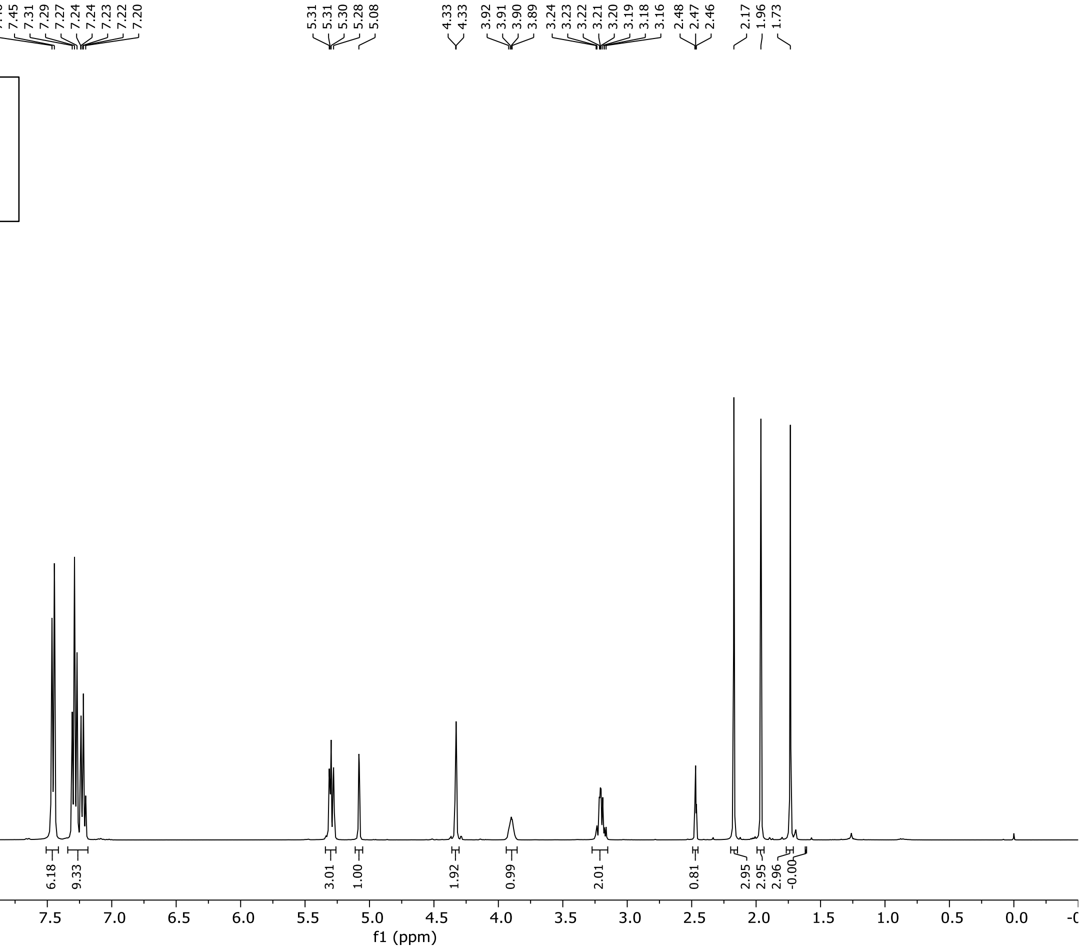

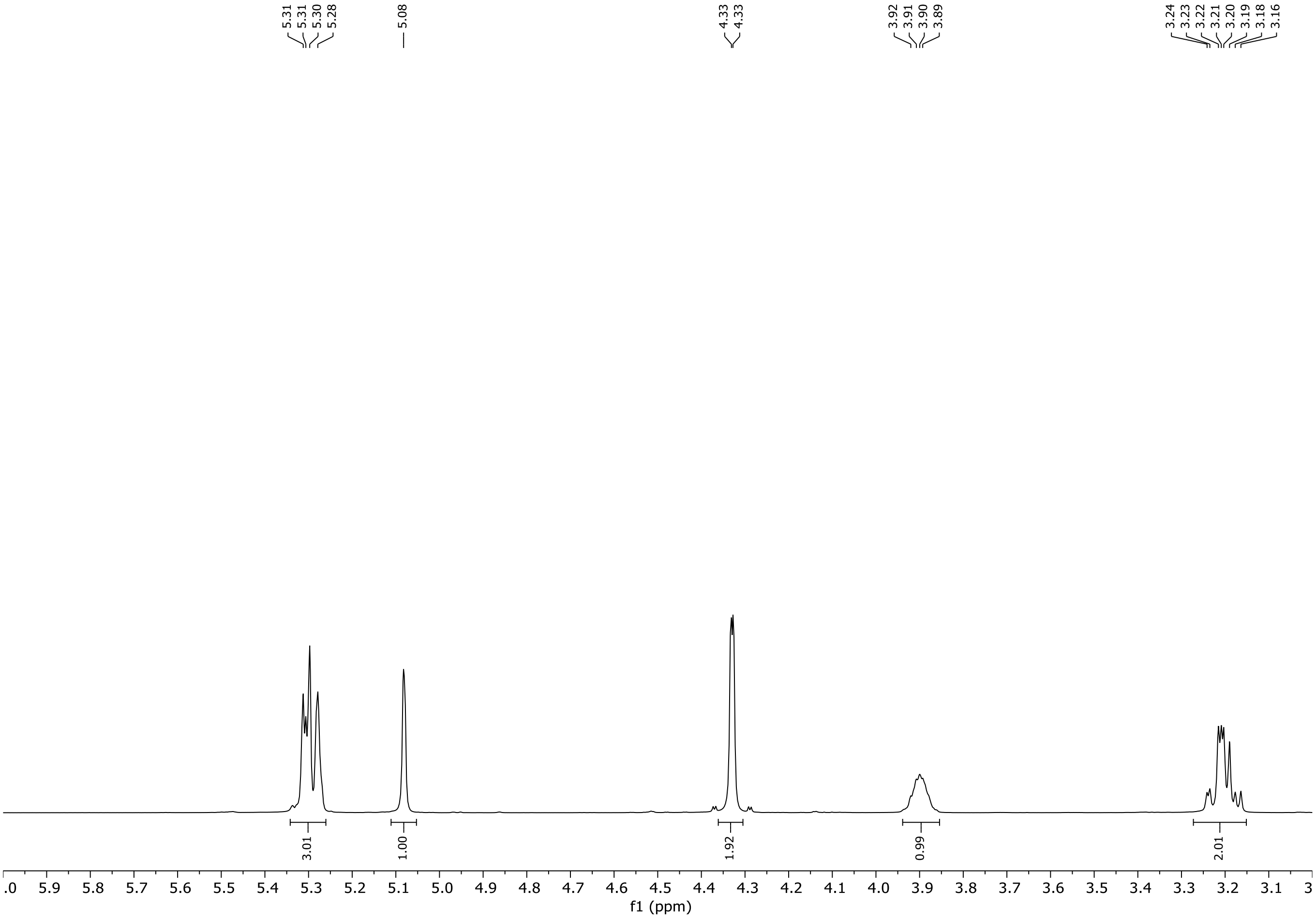

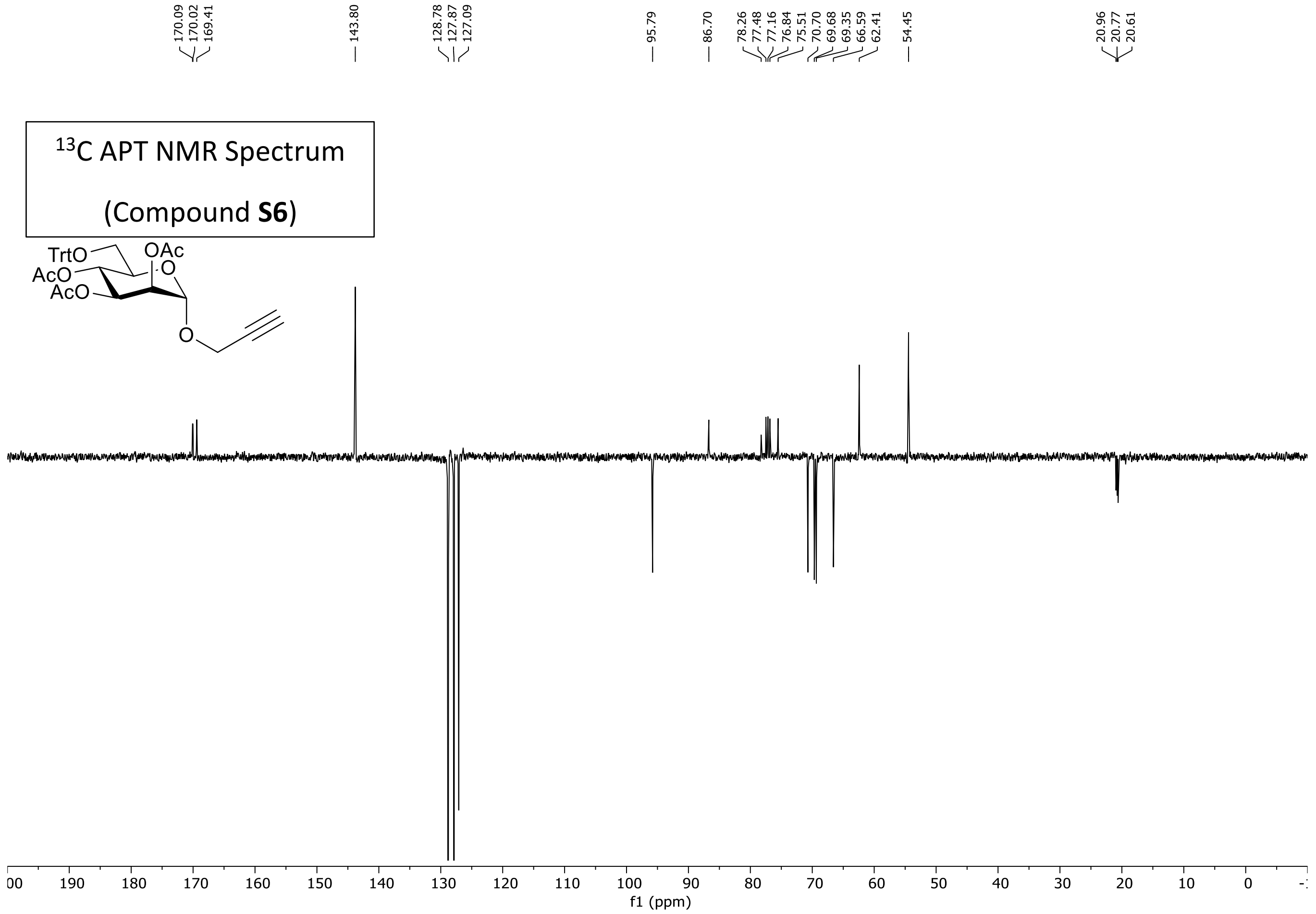
170.09
170.02
169.41
143.80
128.78
127.87
127.09
95.79
86.70
78.26
77.48
77.16
76.84
75.51
70.70
69.68
69.35
66.59
62.41
54.45
20.96
20.77
20.61
^{13}C APT NMR Spectrum
(Compound **S6**)
TrtO
AcO
AcO
OAc
O
O
f1 (ppm)

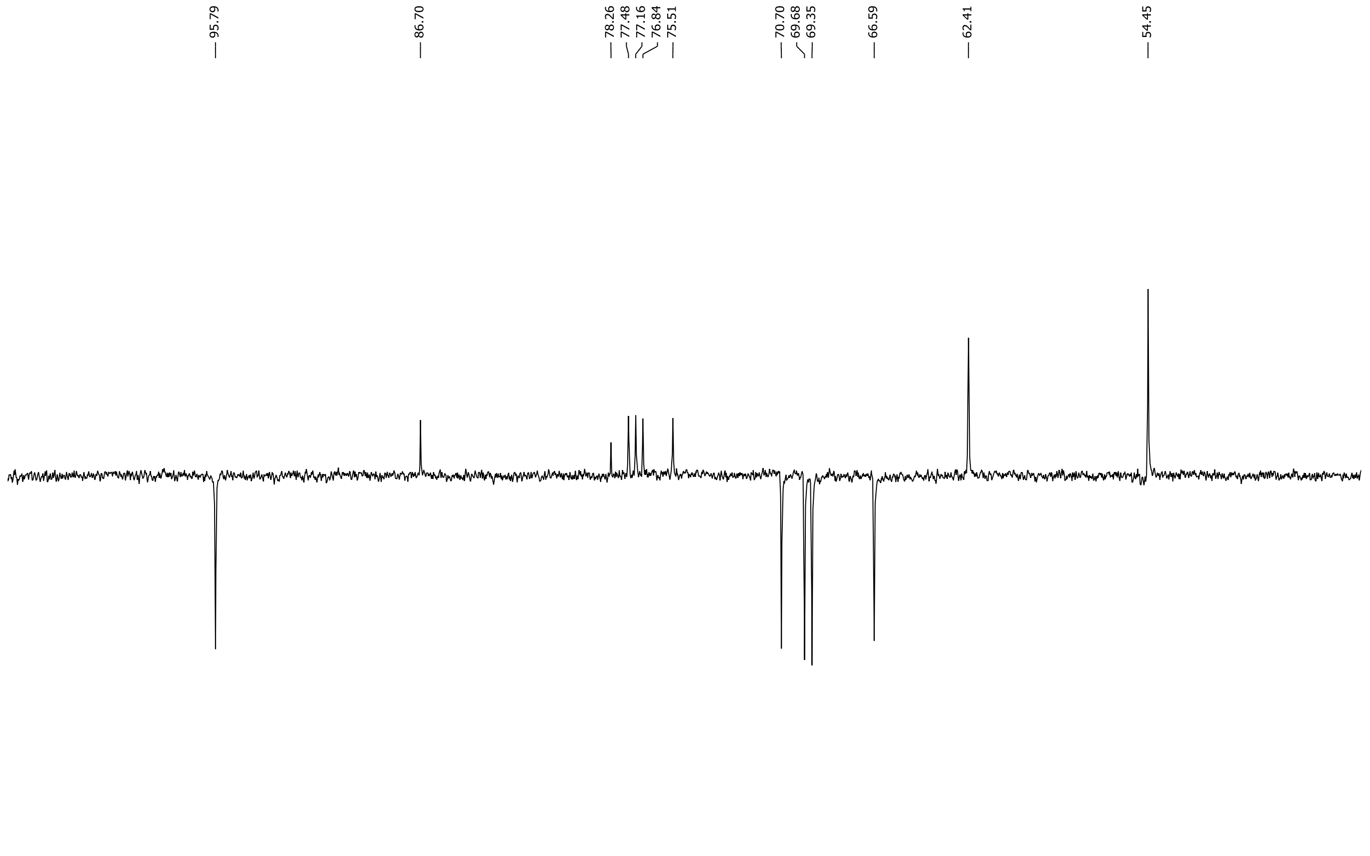
95.79
86.70
78.26
77.48
77.16
76.84
75.51
70.70
69.68
69.35
66.59
62.41
54.45
f1 (ppm)

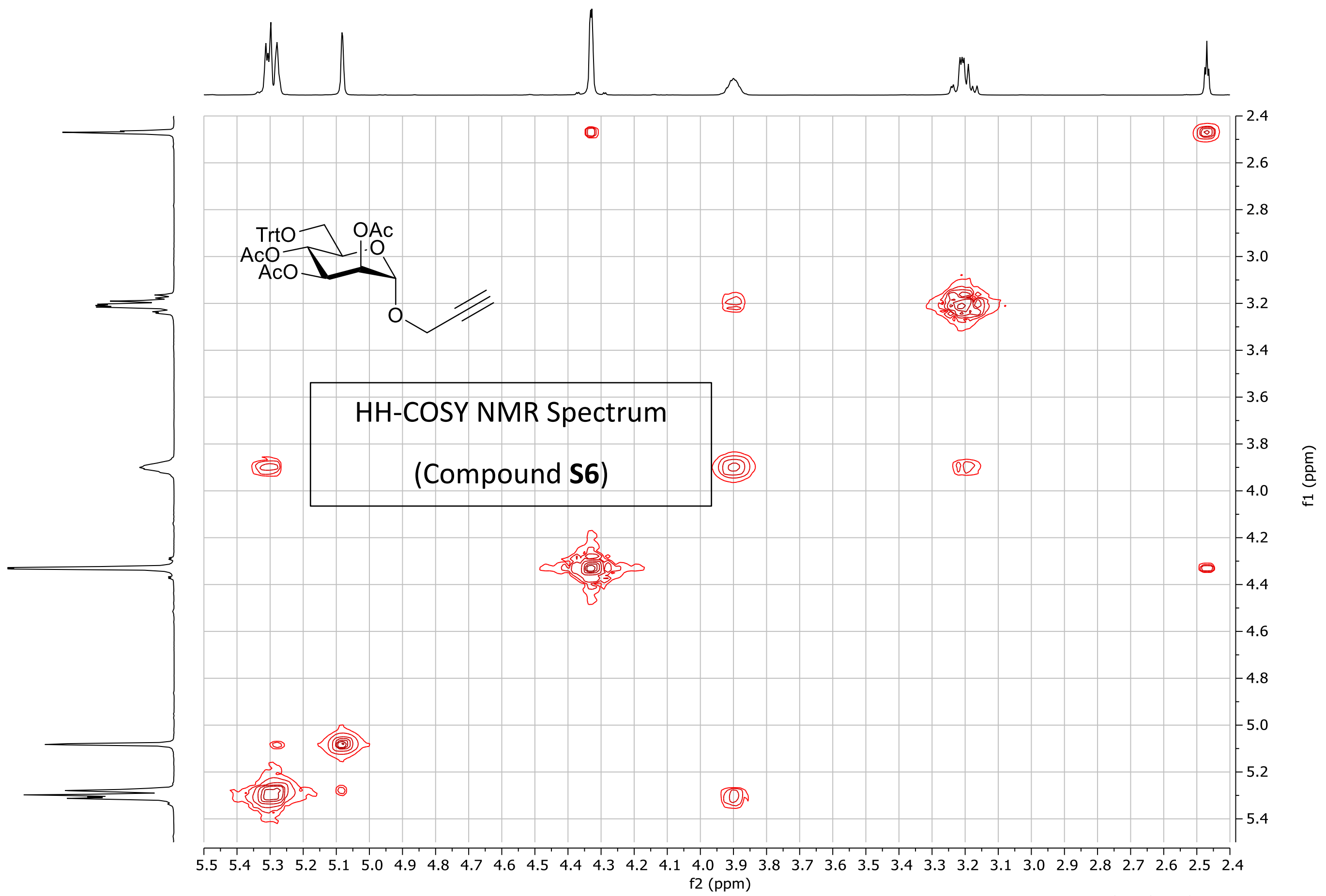
TrtO
AcO
AcO
OAc
O
O
HH-COSY NMR Spectrum
(Compound **S6**)
f2 (ppm)
f1 (ppm)

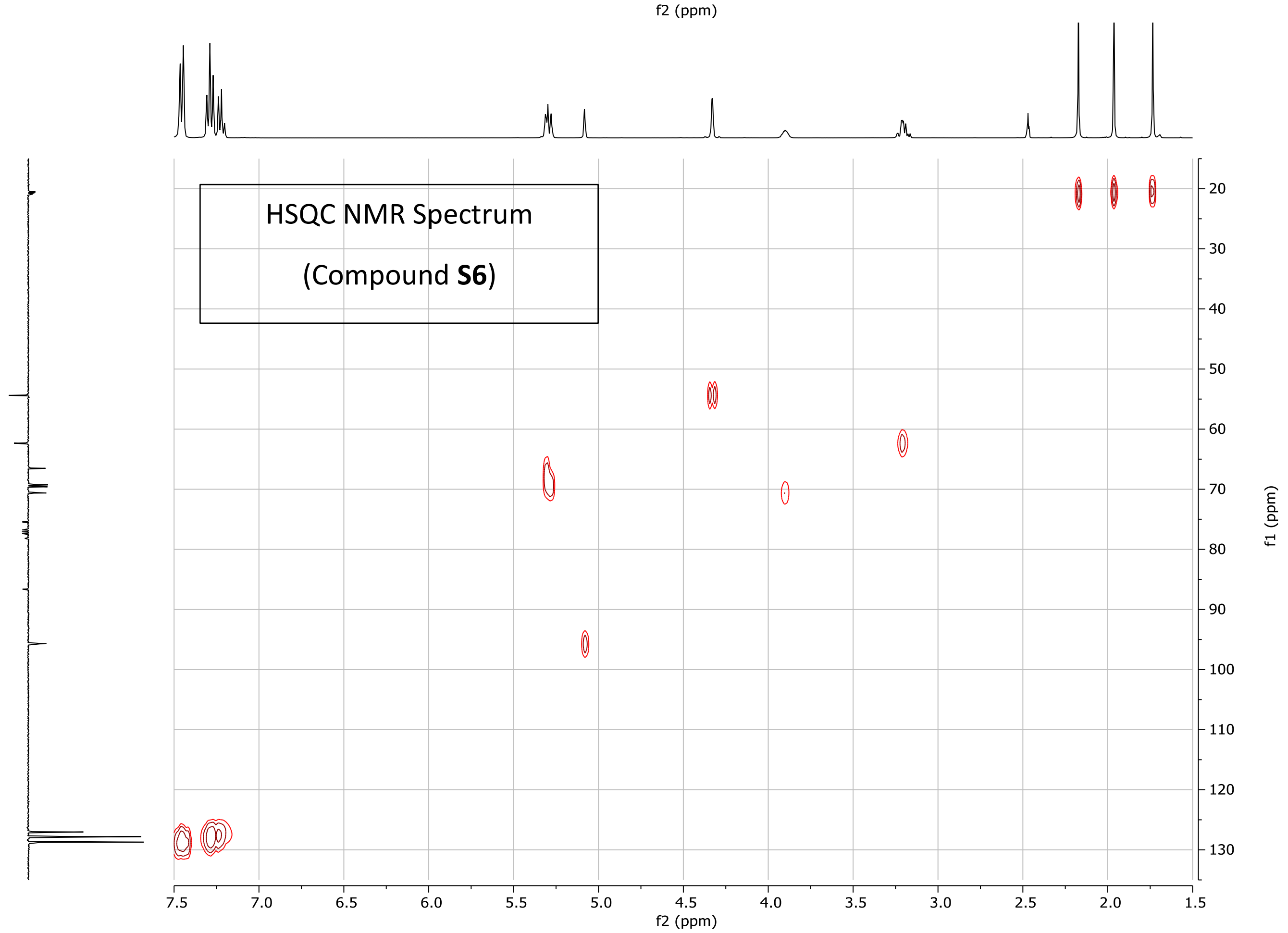
HSQC NMR Spectrum
(Compound **S6**)
f2 (ppm)
f1 (ppm)

9

^{1}H NMR Spectrum

(Compound **9**)

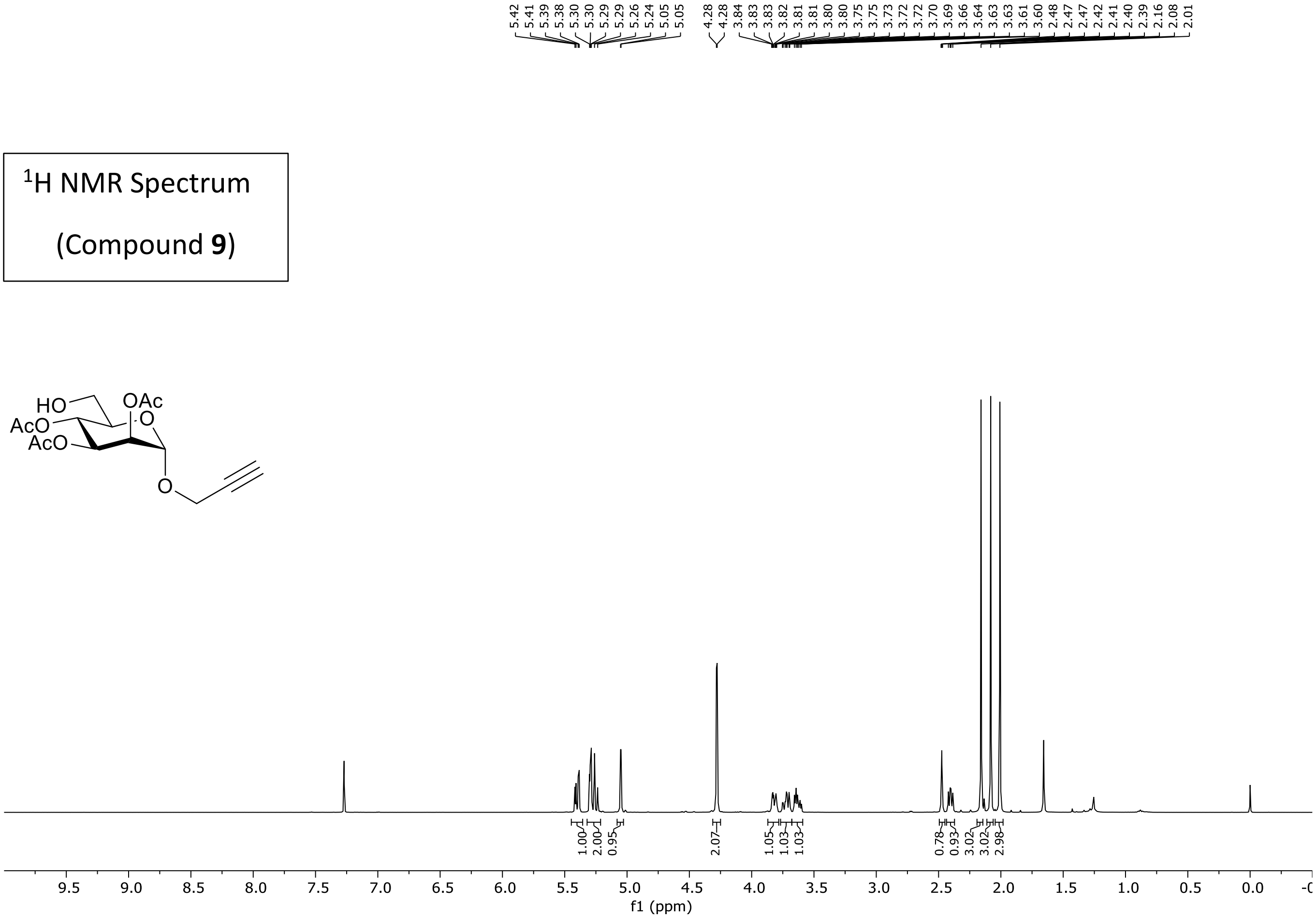

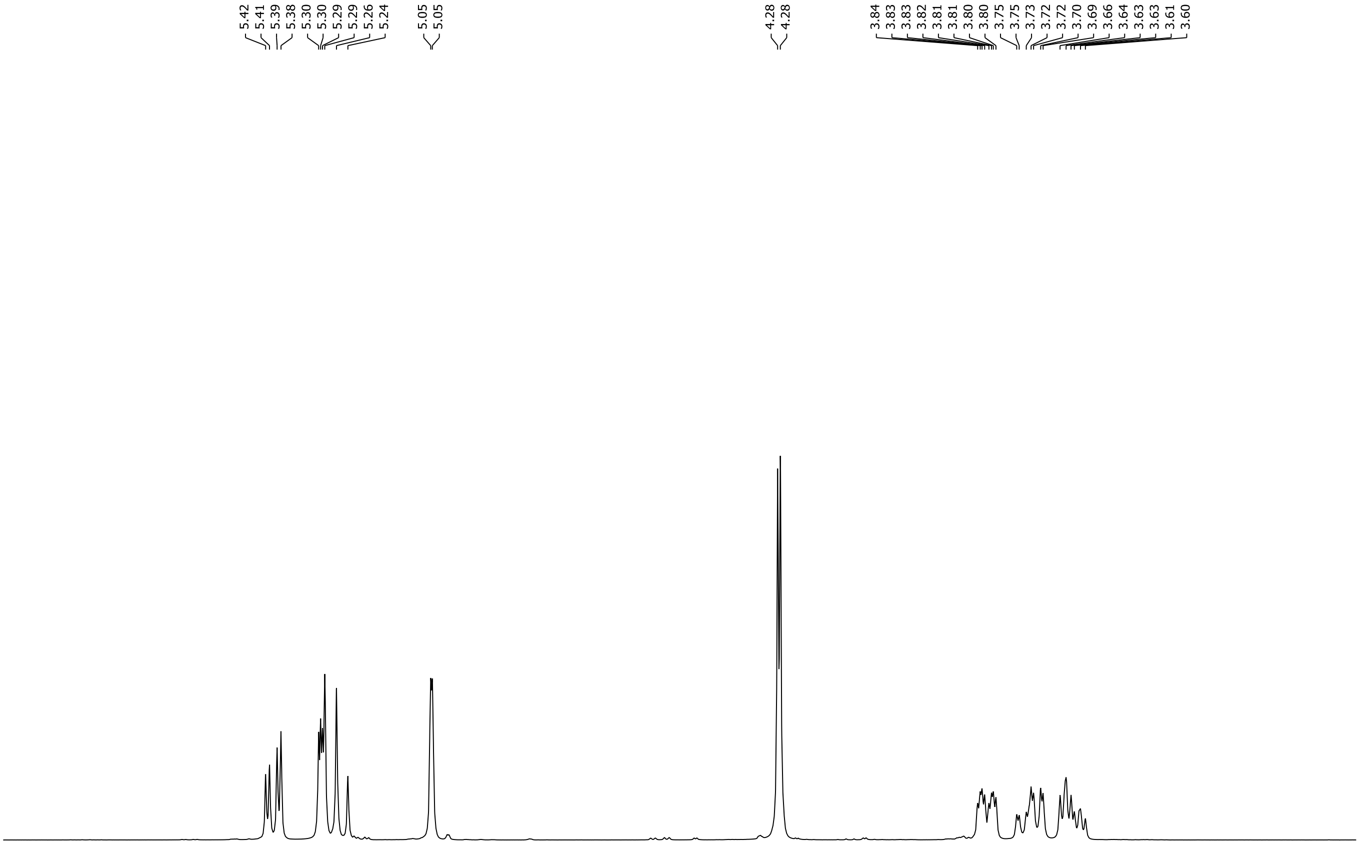

^{13}C APT NMR Spectrum

(Compound **9**)

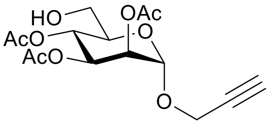

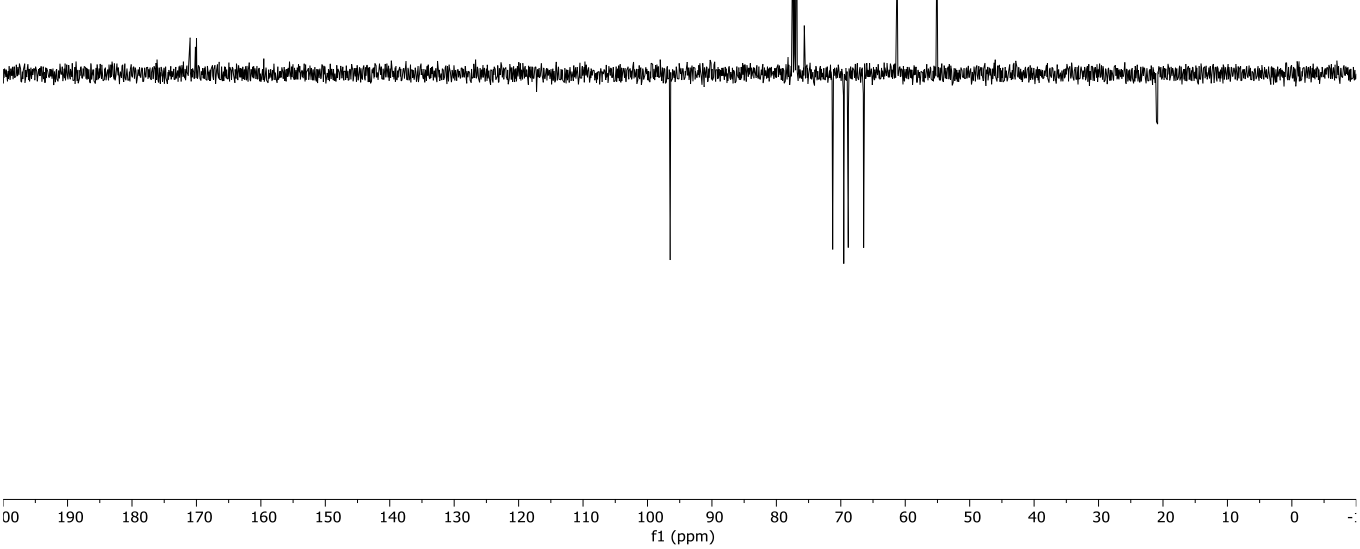

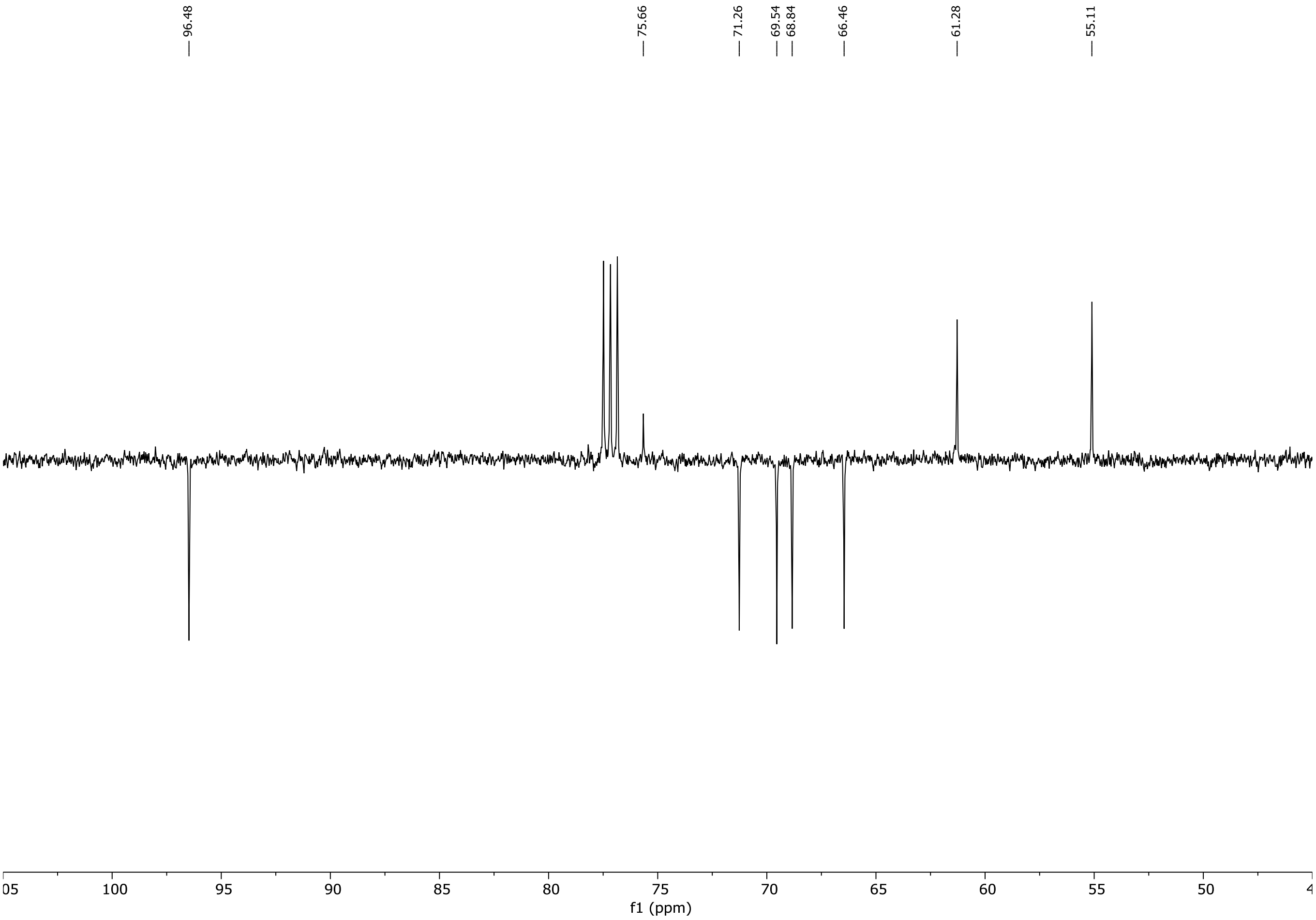

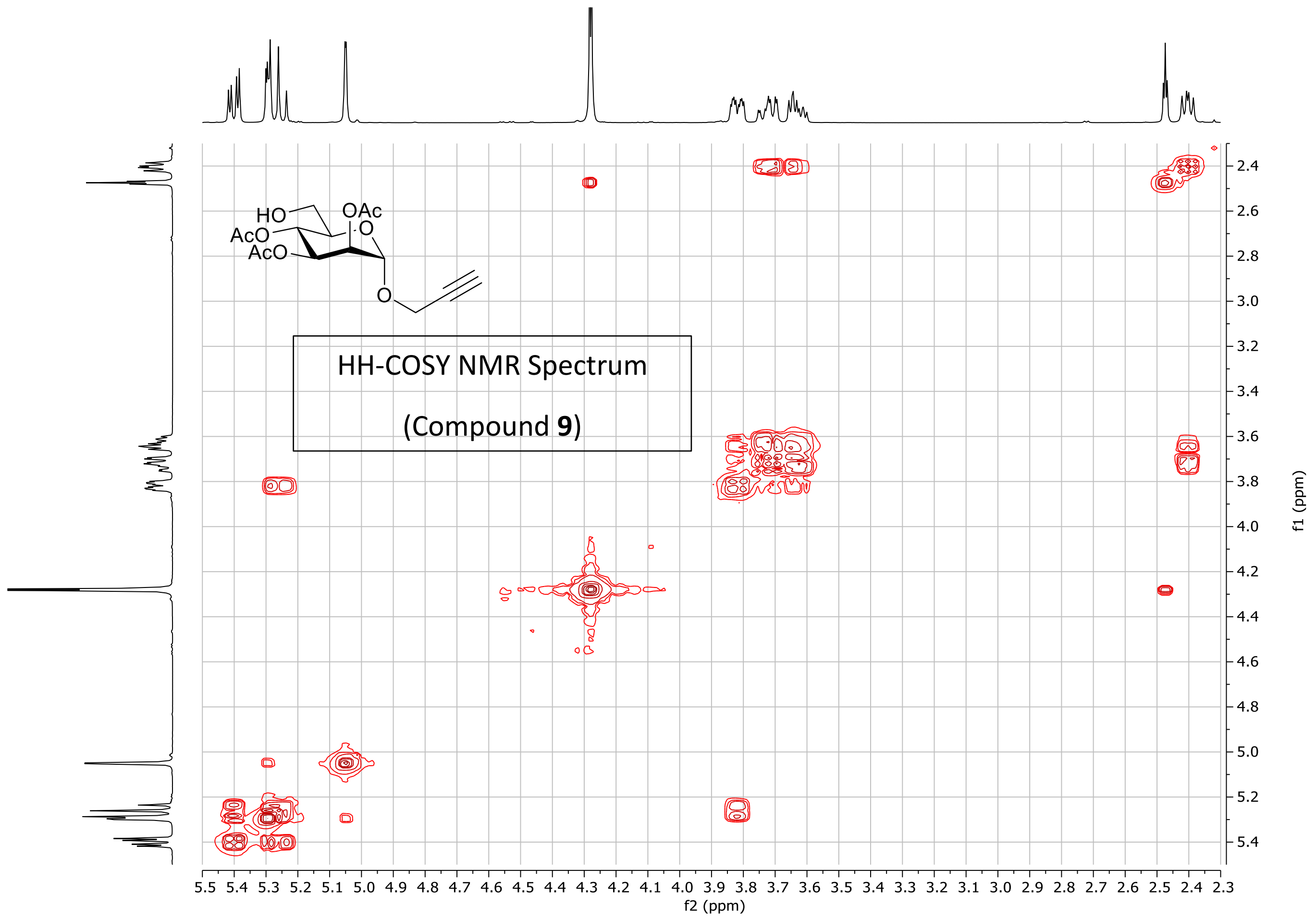
HO
OAc
AcO
O
AcO
O
HH-COSY NMR Spectrum
(Compound **9**)
f1 (ppm)
f2 (ppm)

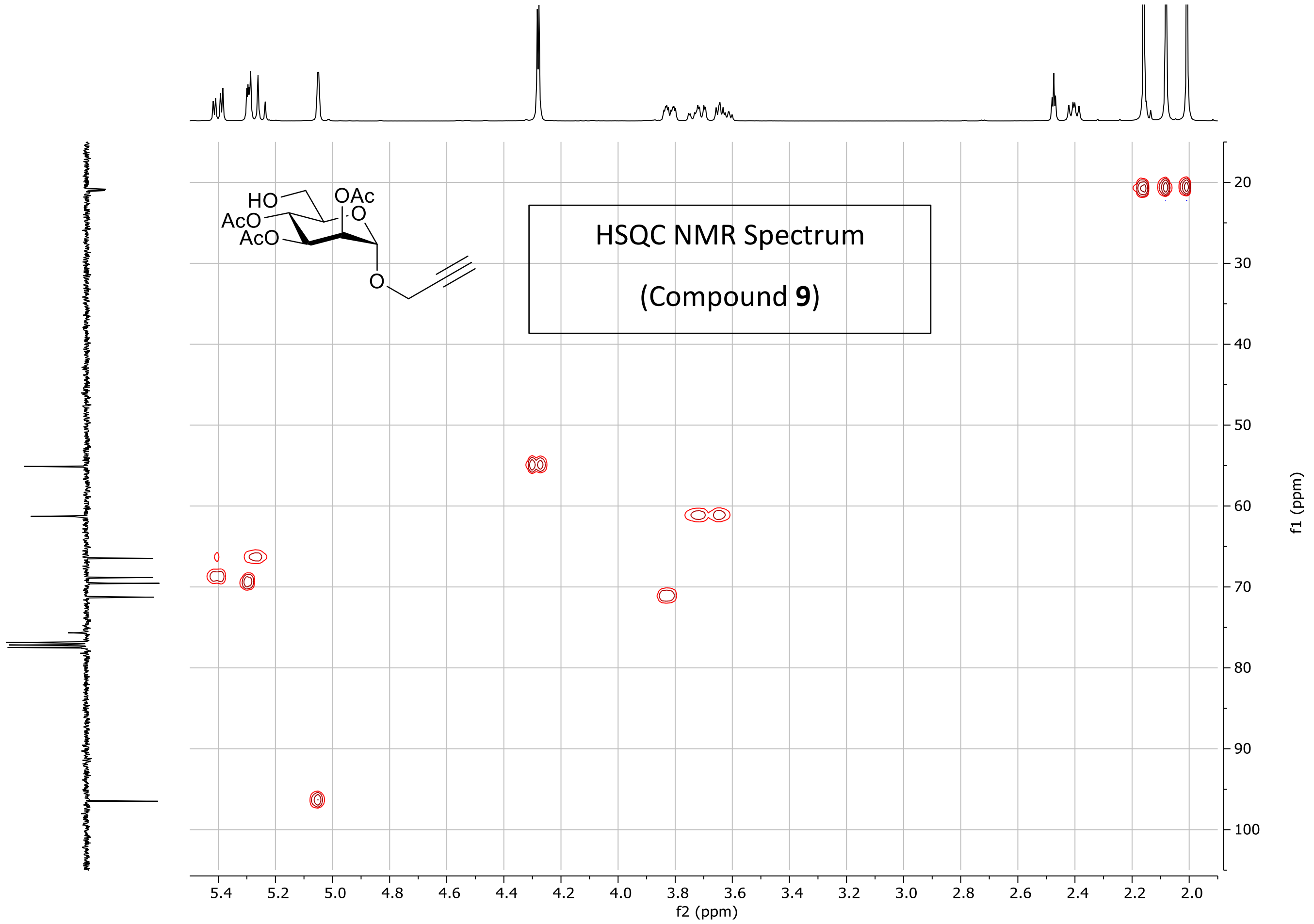
HSQC NMR Spectrum
(Compound 9)
HO
OAc
O
AcO
AcO
O
f2 (ppm)
f1 (ppm)

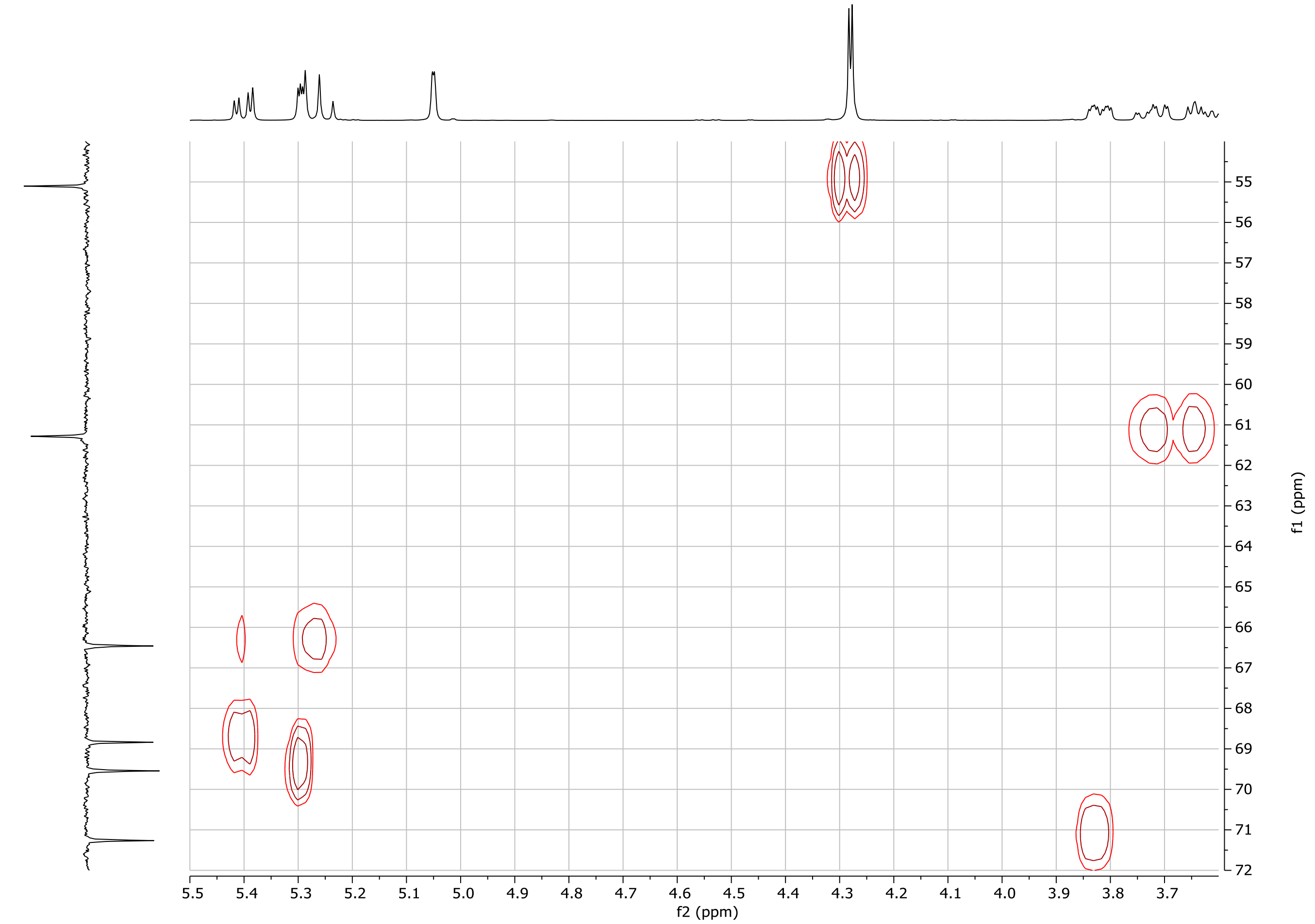
f2 (ppm)
f1 (ppm)

10

^{1}H NMR Spectrum

(Compound **10**)

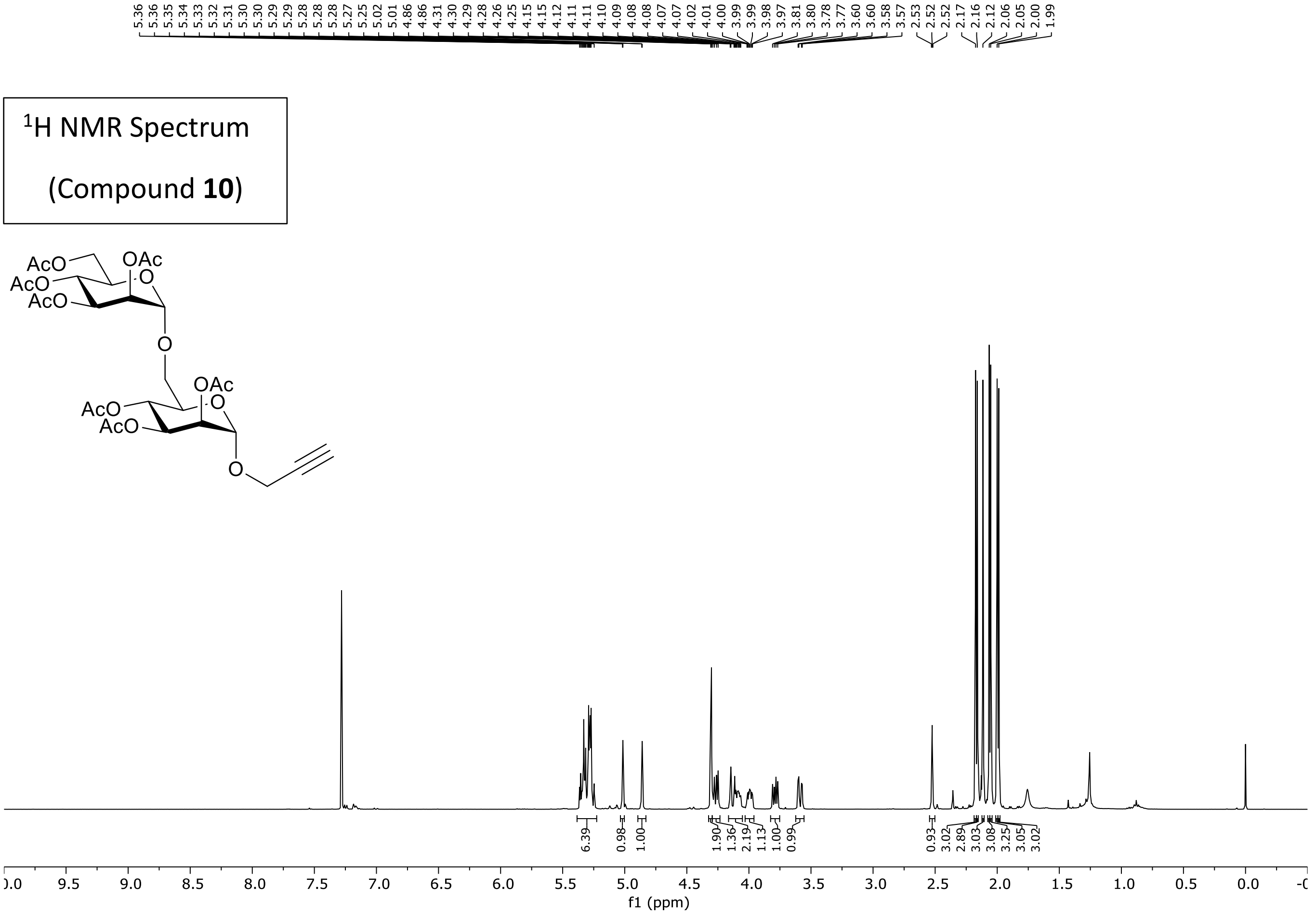

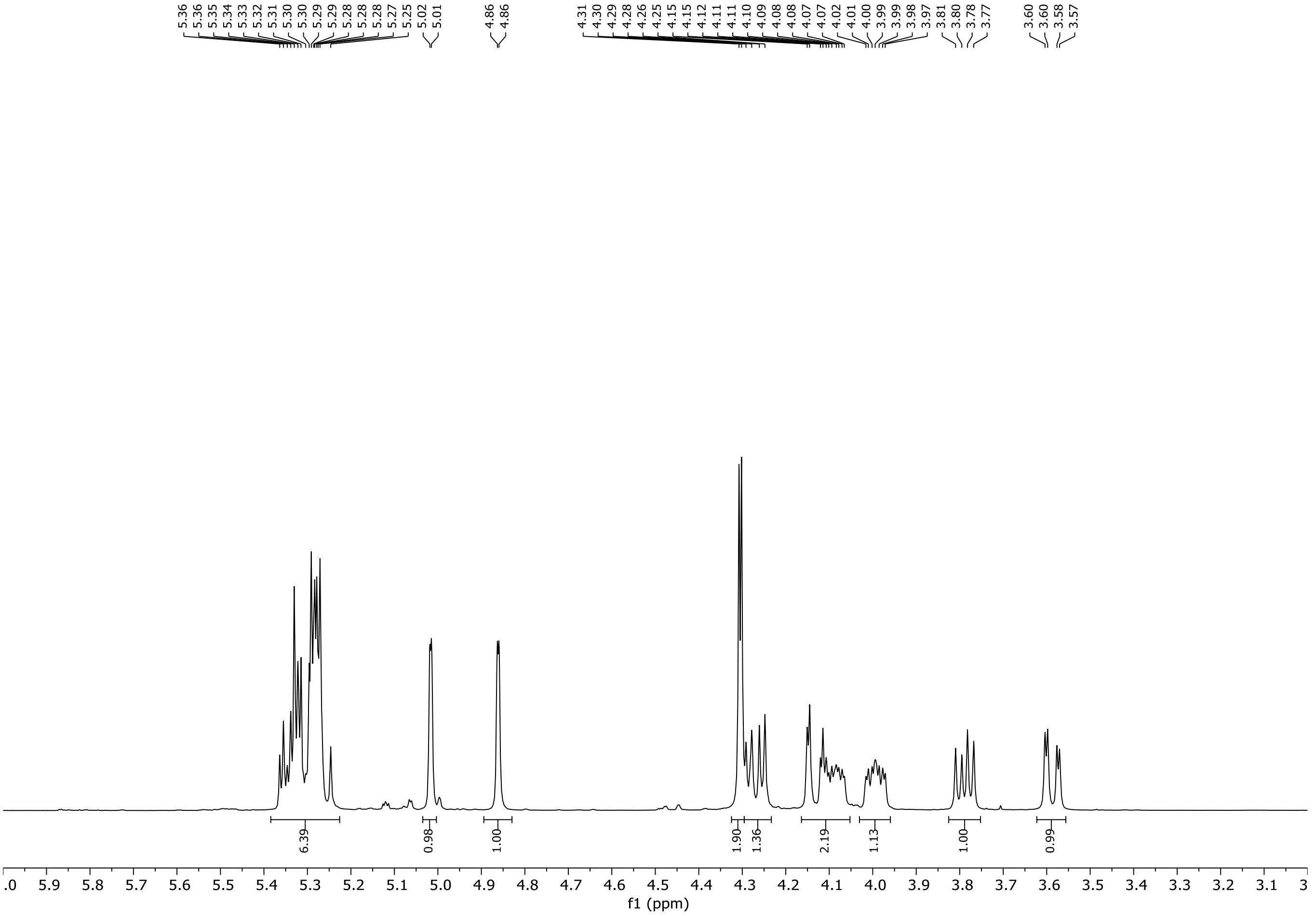

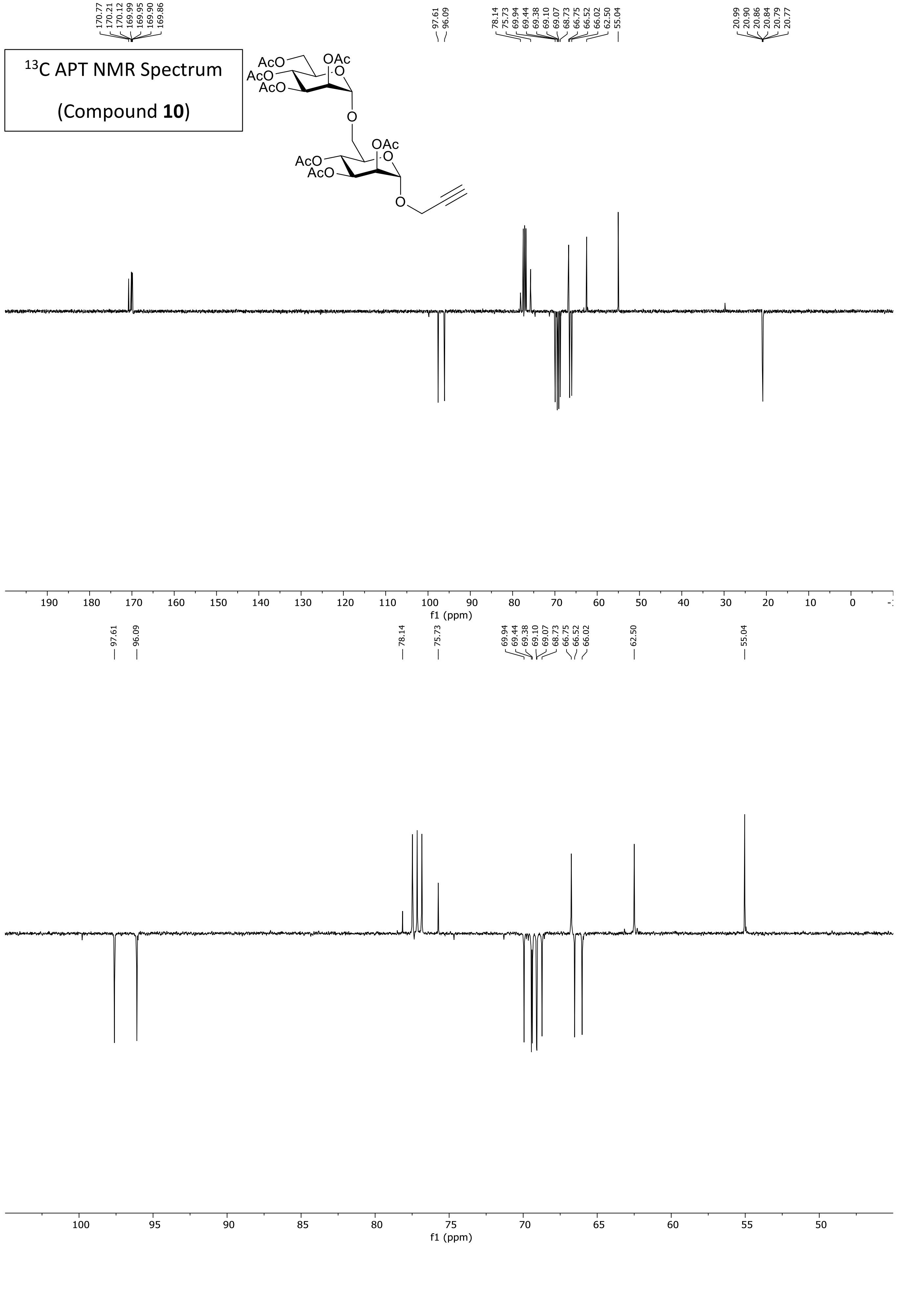
^{13}C APT NMR Spectrum
(Compound **10**)
AcO
AcO
AcO
OAc
O
O
OAc
AcO
AcO
O
O
170.77
170.21
170.12
169.99
169.95
169.90
169.86
97.61
96.09
78.14
75.73
69.94
69.44
69.38
69.10
69.07
68.73
66.75
66.52
66.02
62.50
55.04
20.99
20.90
20.86
20.84
20.79
20.77
f1 (ppm)
97.61
96.09
78.14
75.73
69.94
69.44
69.38
69.10
69.07
68.73
66.75
66.52
66.02
62.50
55.04
f1 (ppm)

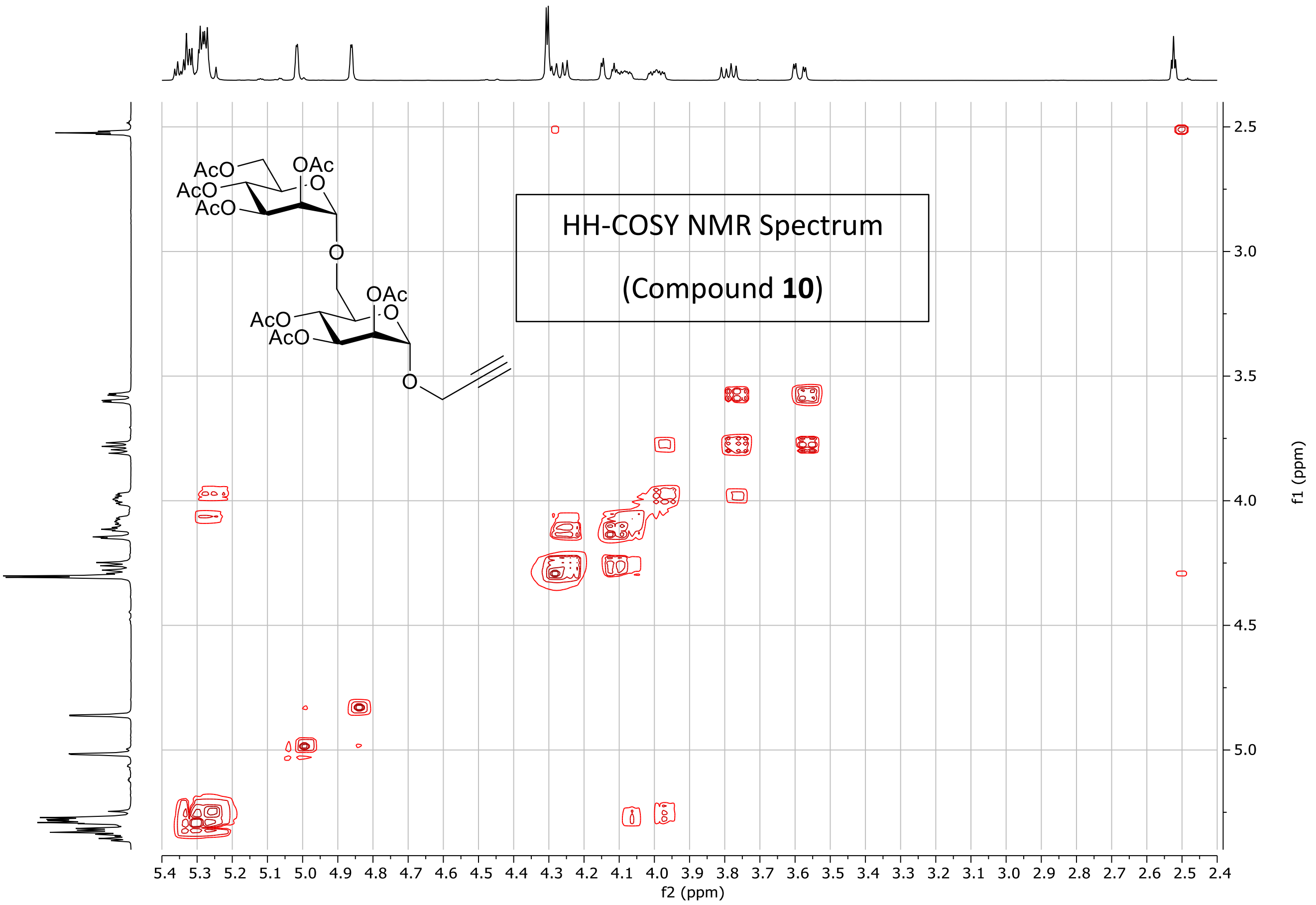
AcO
OAc
AcO
O
AcO
O
OAc
AcO
O
AcO
O
HH-COSY NMR Spectrum
(Compound **10**)
f2 (ppm)
f1 (ppm)

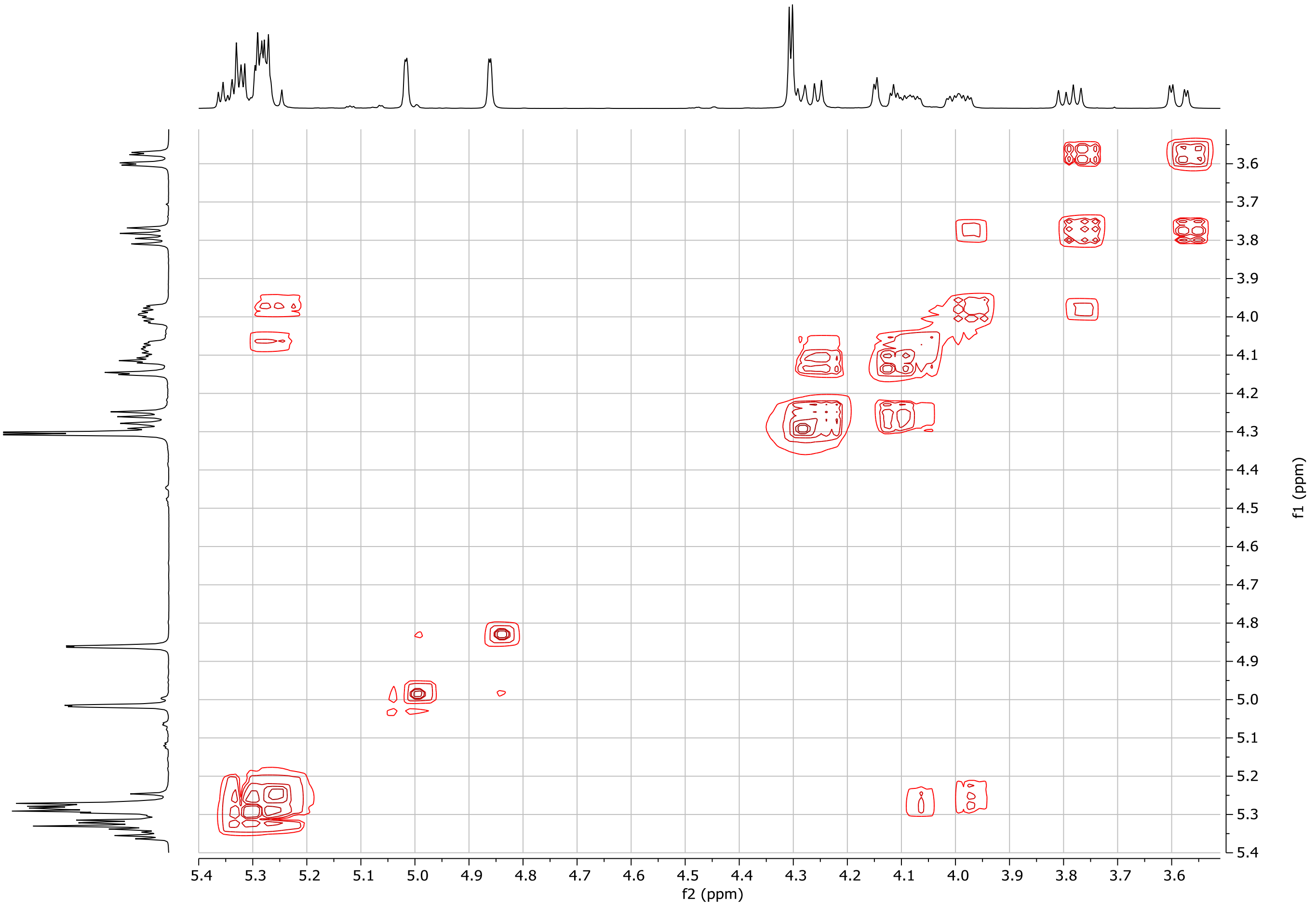
f2 (ppm)
f1 (ppm)

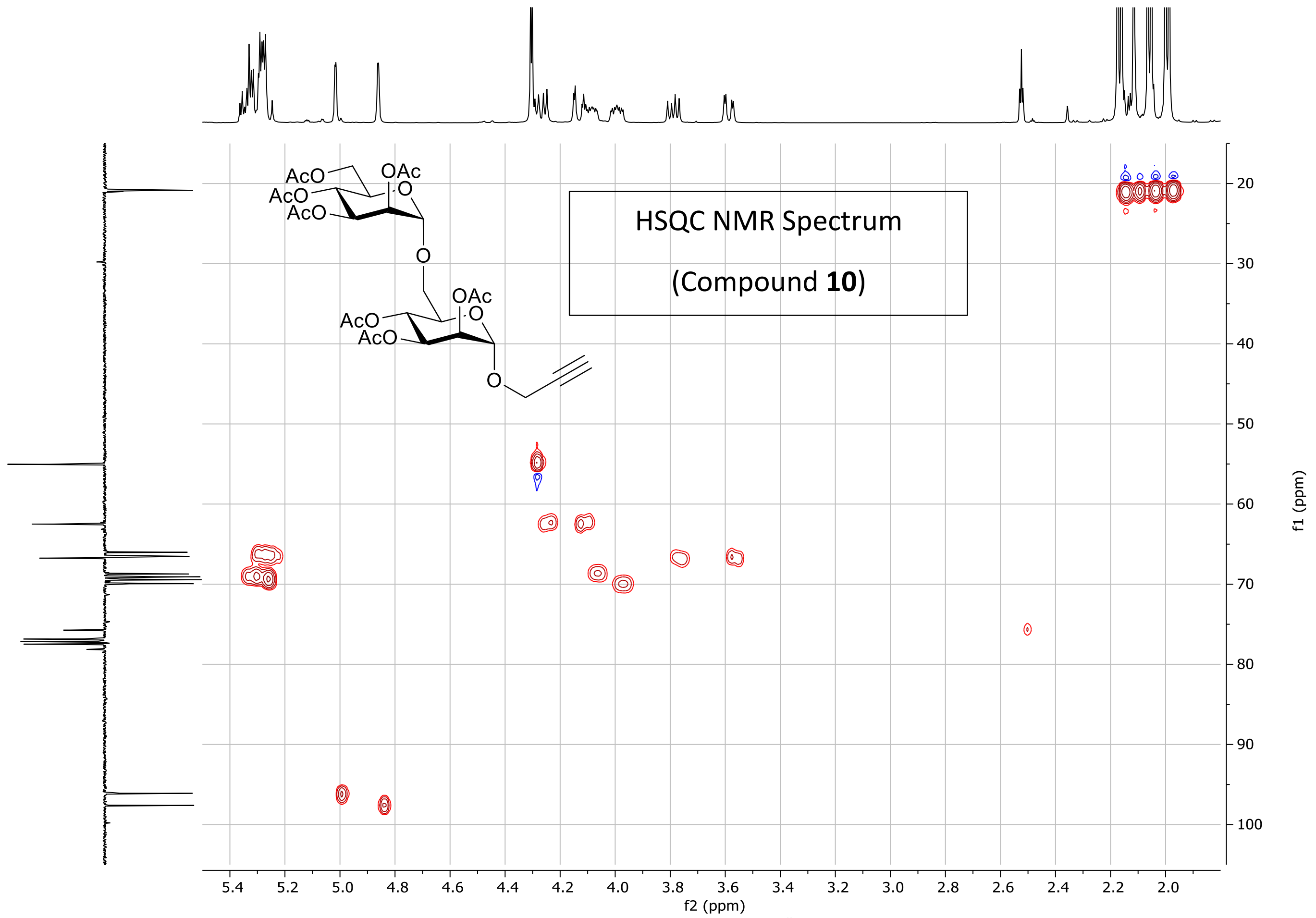

HSQC NMR Spectrum
(Compound **10**)
AcO
OAc
O
f2 (ppm)
f1 (ppm)

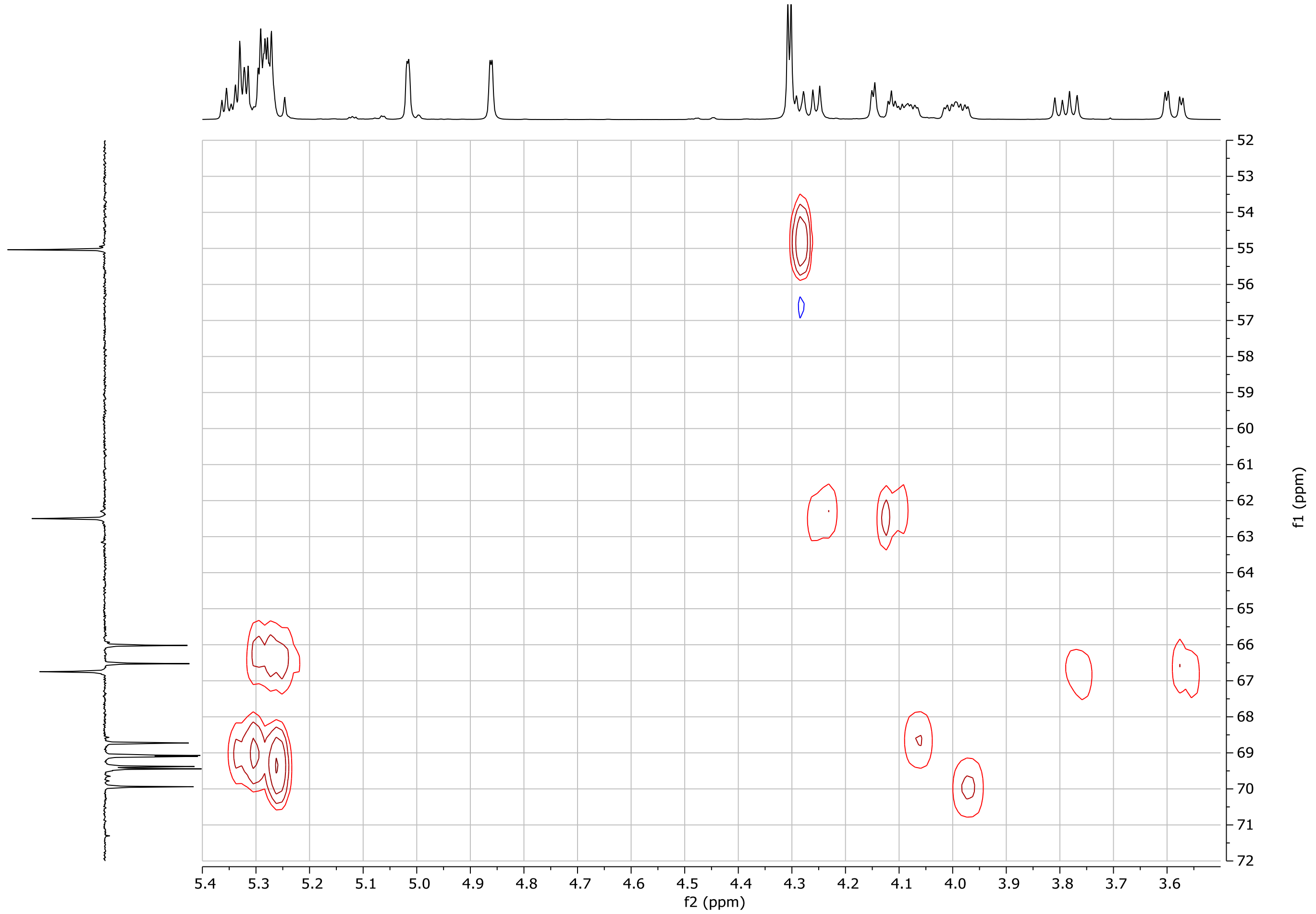

f2 (ppm)
f1 (ppm)

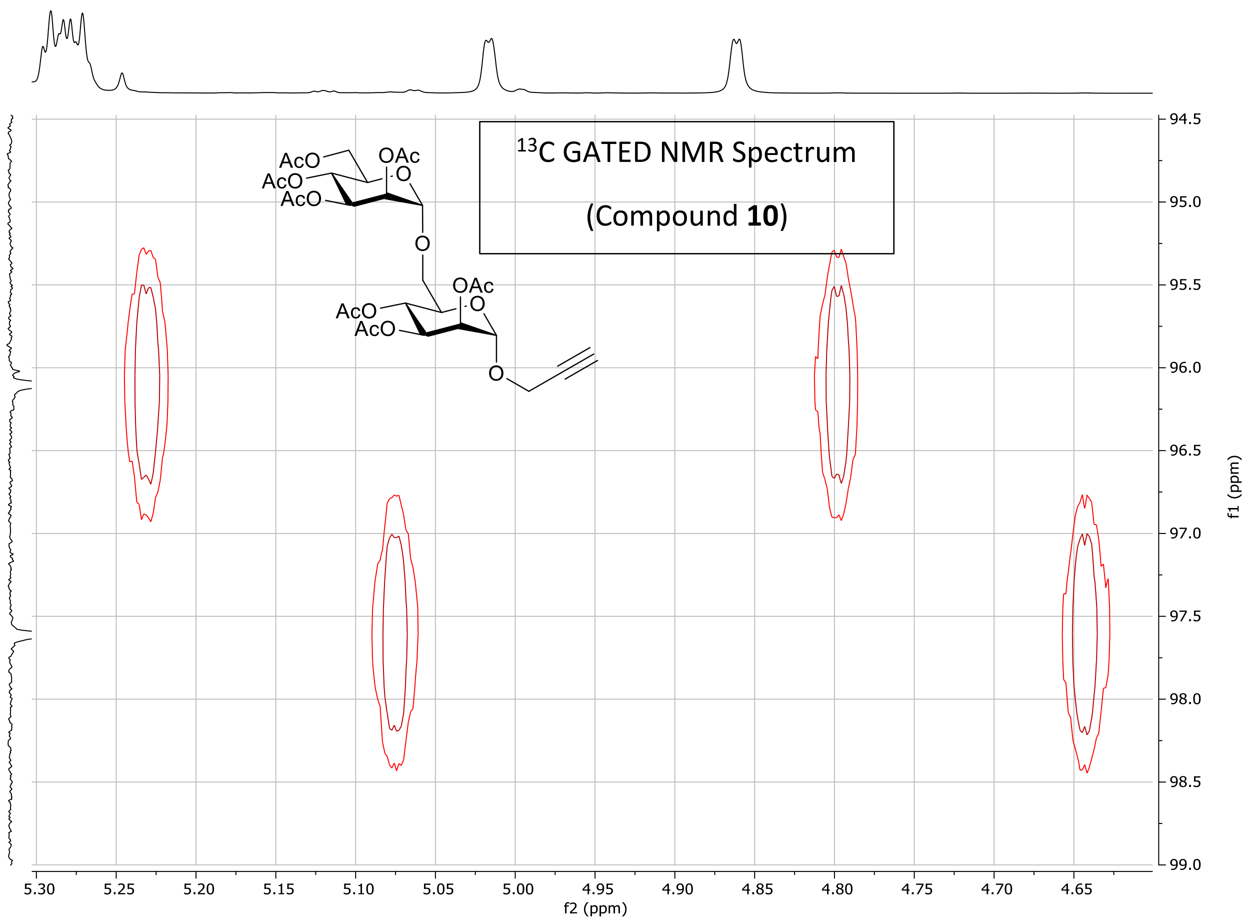

^{13}C GATED NMR Spectrum
(Compound **10**)
AcO
AcO
AcO
OAc
O
O
OAc
O
AcO
AcO
O
94.5
95.0
95.5
96.0
96.5
97.0
97.5
98.0
98.5
99.0
f1 (ppm)
5.30
5.25
5.20
5.15
5.10
5.05
5.00
4.95
4.90
4.85
4.80
4.75
4.70
4.65
f2 (ppm)

11

^{1}H NMR Spectrum

(Compound **11**)

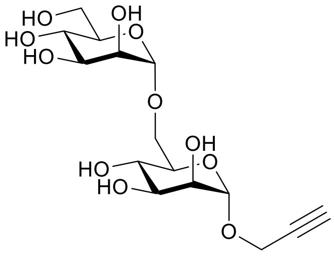

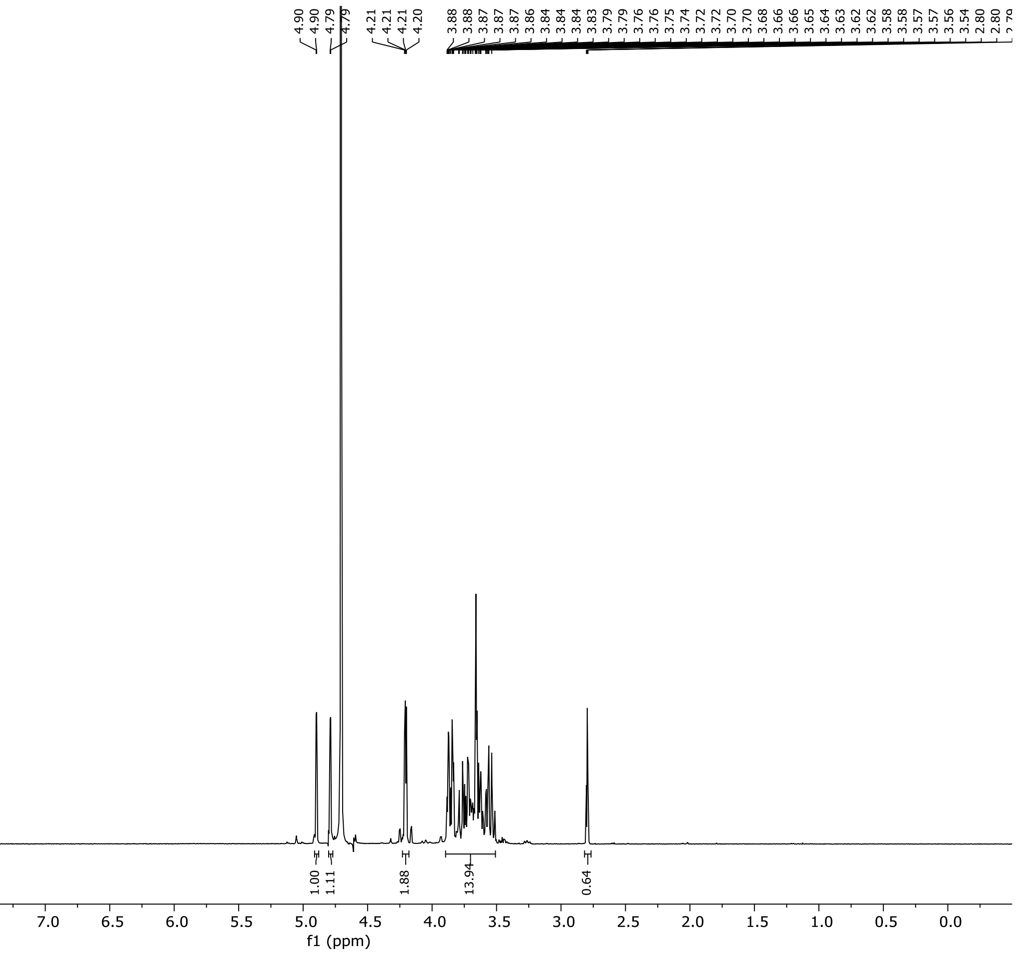

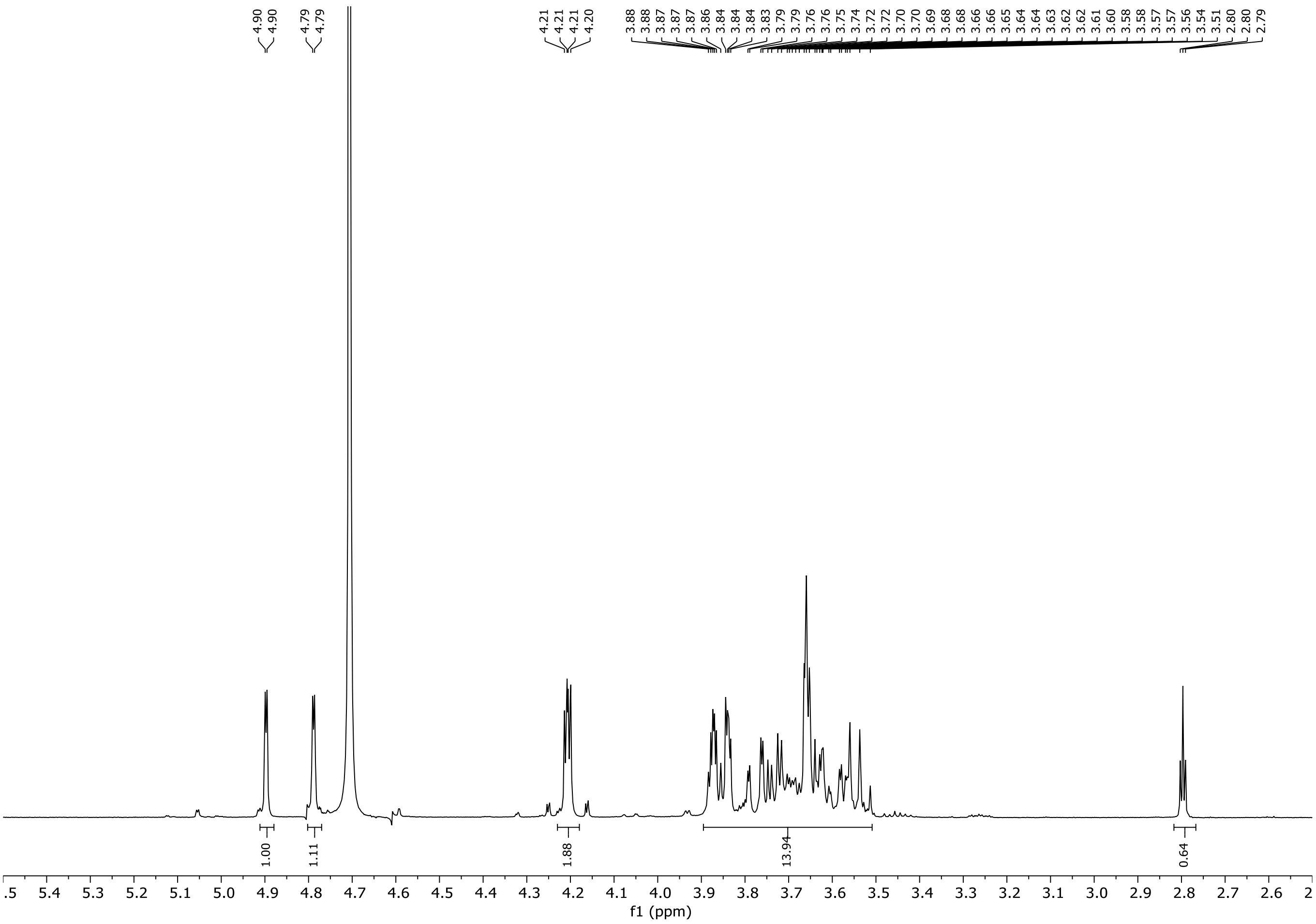

^{13}C APT NMR Spectrum

(Compound **11**)

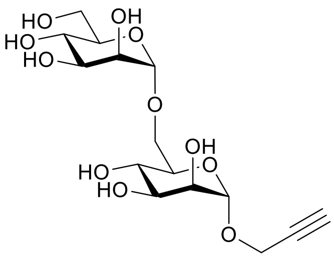

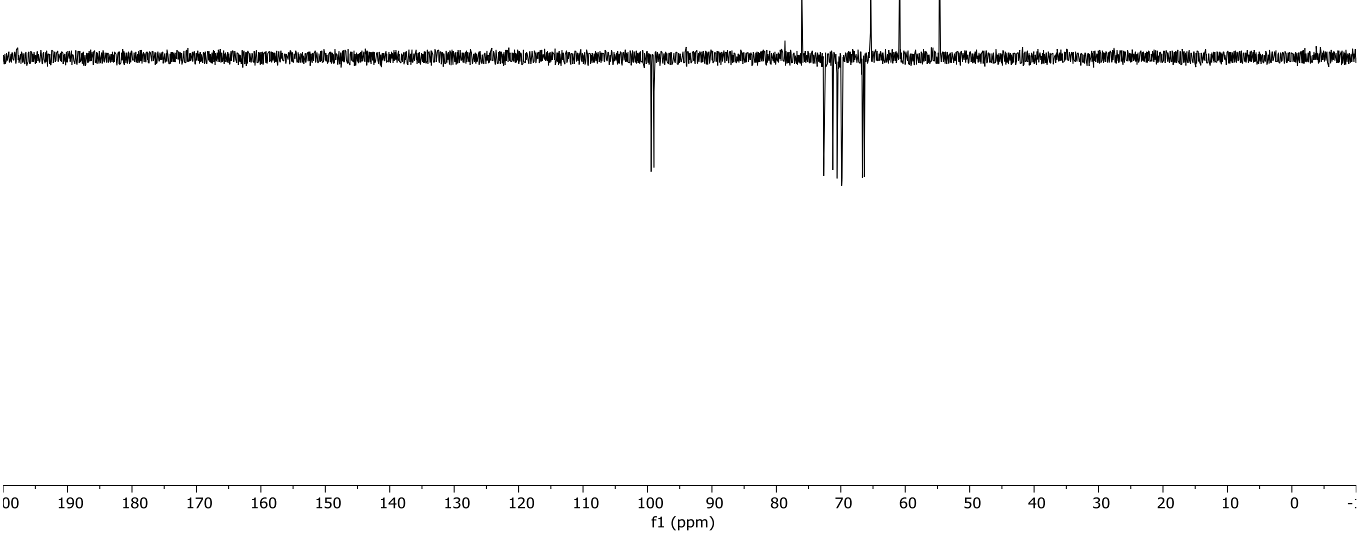

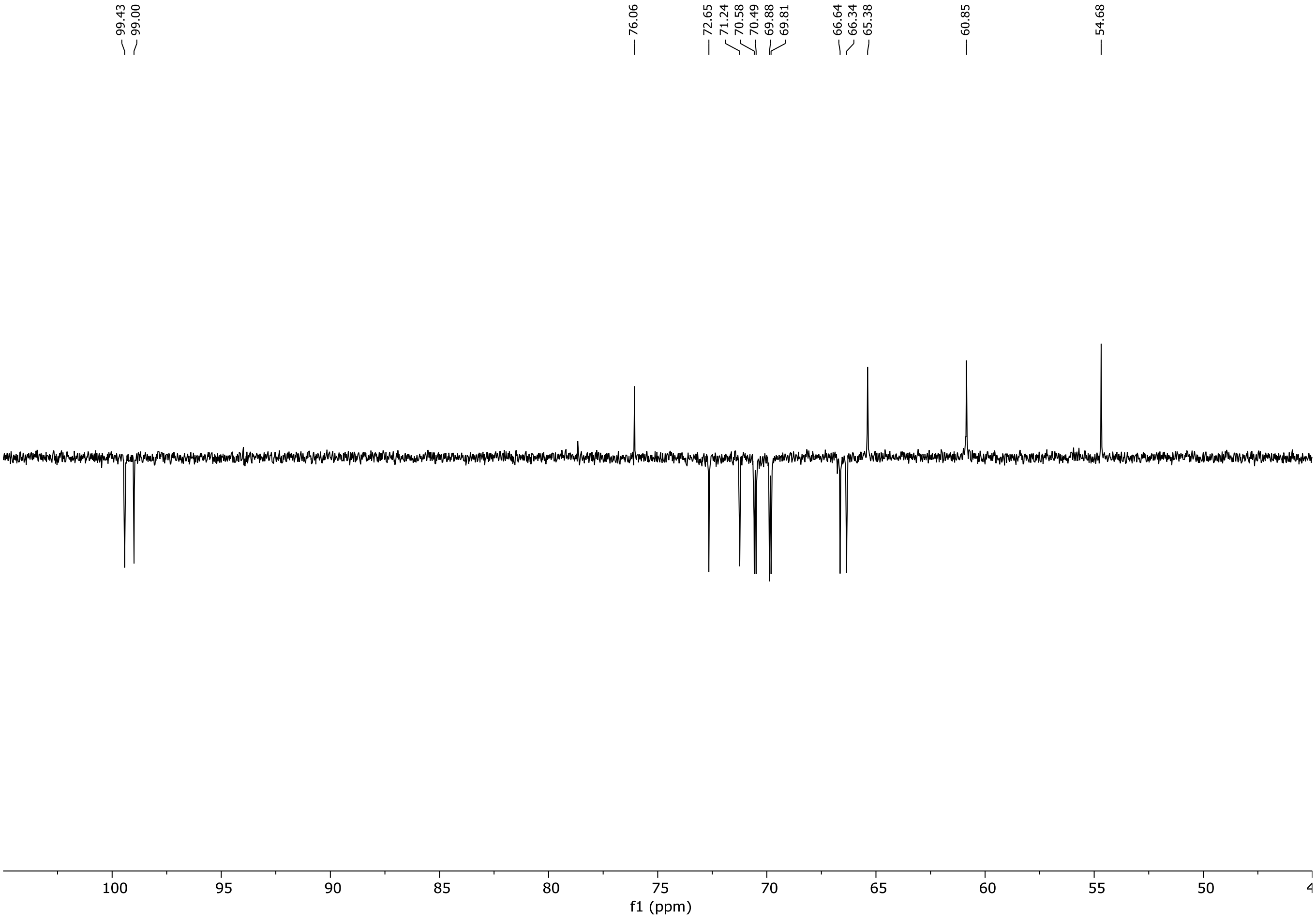

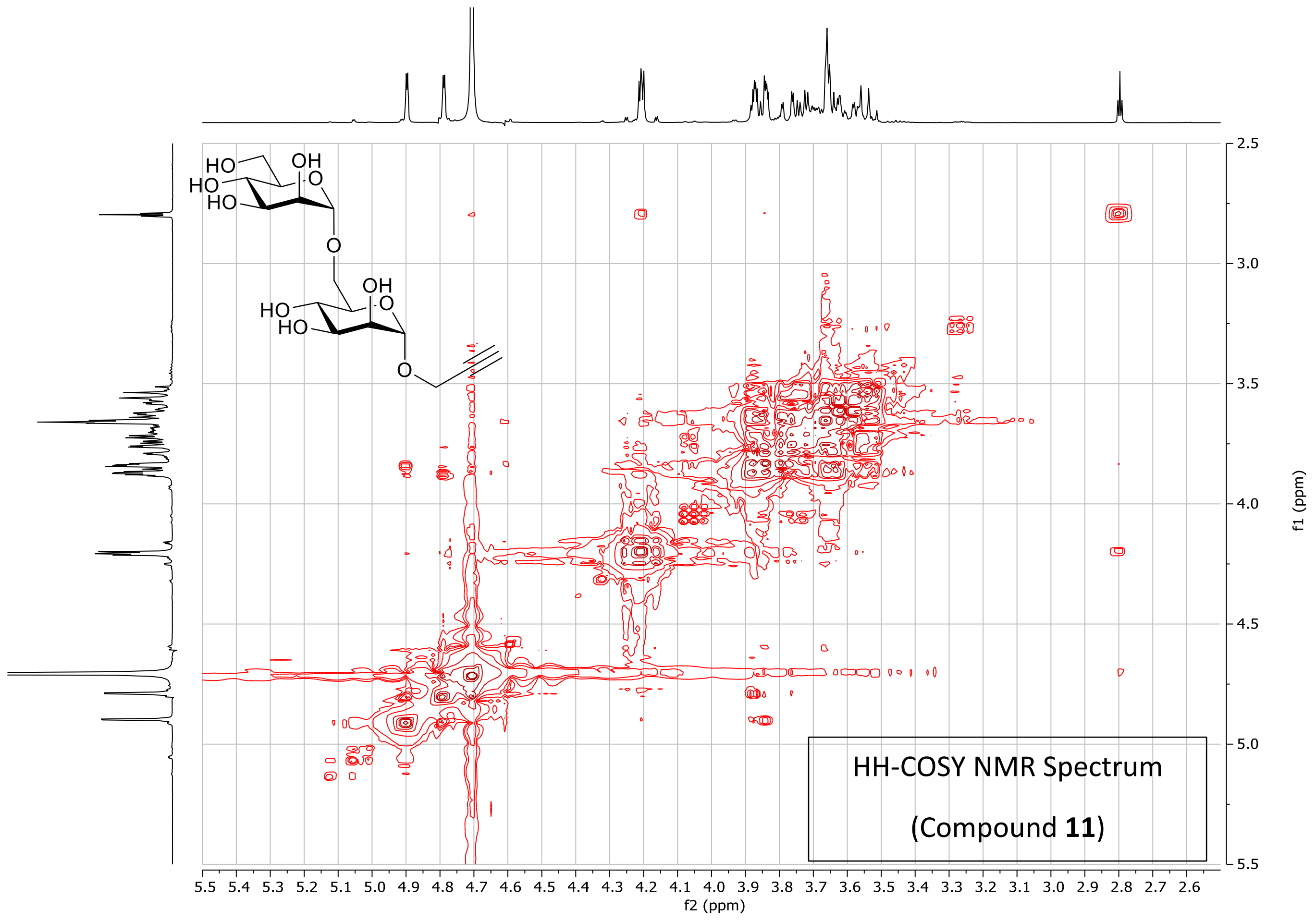
HH-COSY NMR Spectrum
(Compound **11**)
f2 (ppm)
f1 (ppm)

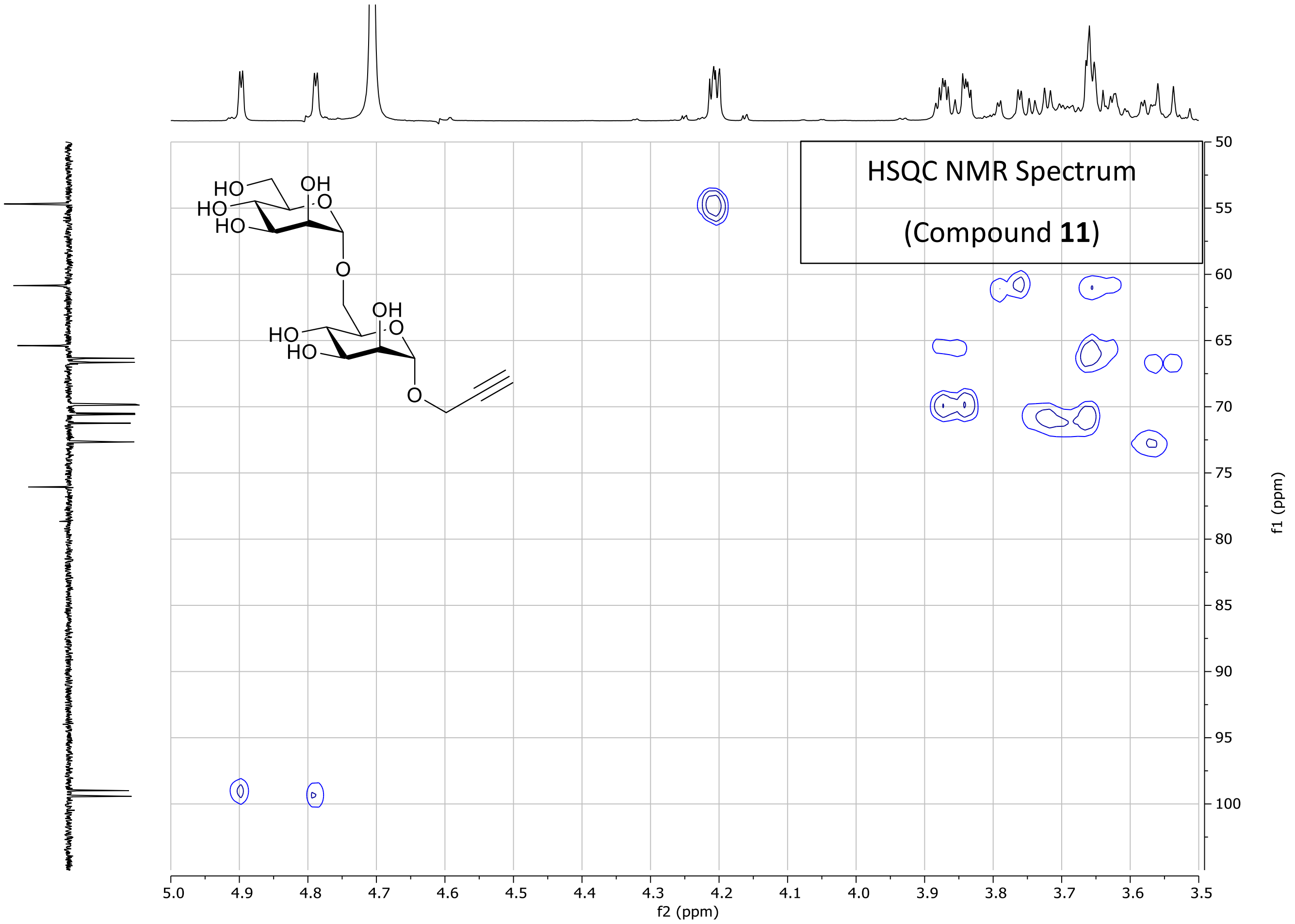
HSQC NMR Spectrum
(Compound **11**)
HO
HO
HO
OH
O
O
OH
HO
HO
O
O
f2 (ppm)
f1 (ppm)

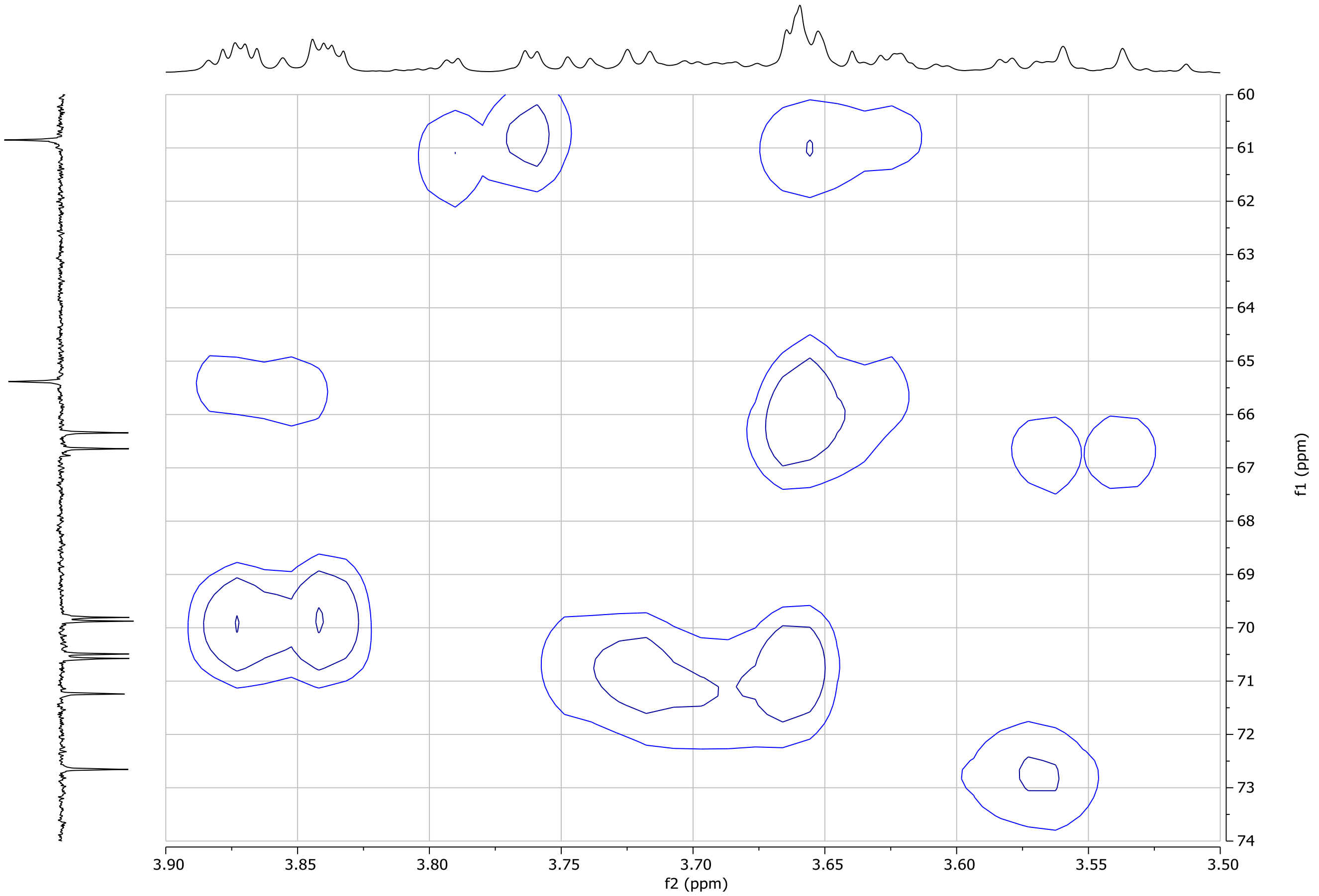
f2 (ppm)
f1 (ppm)

14

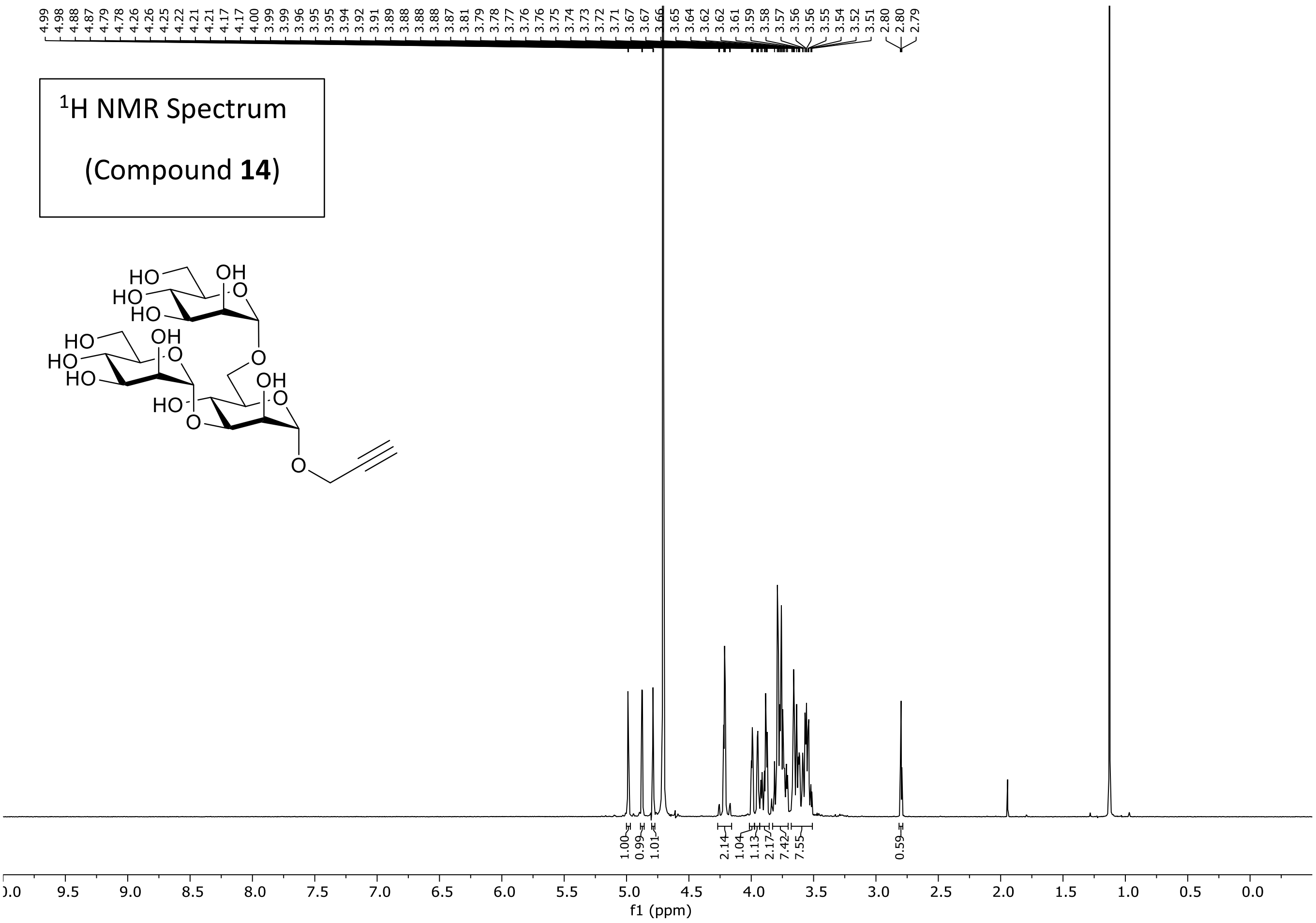

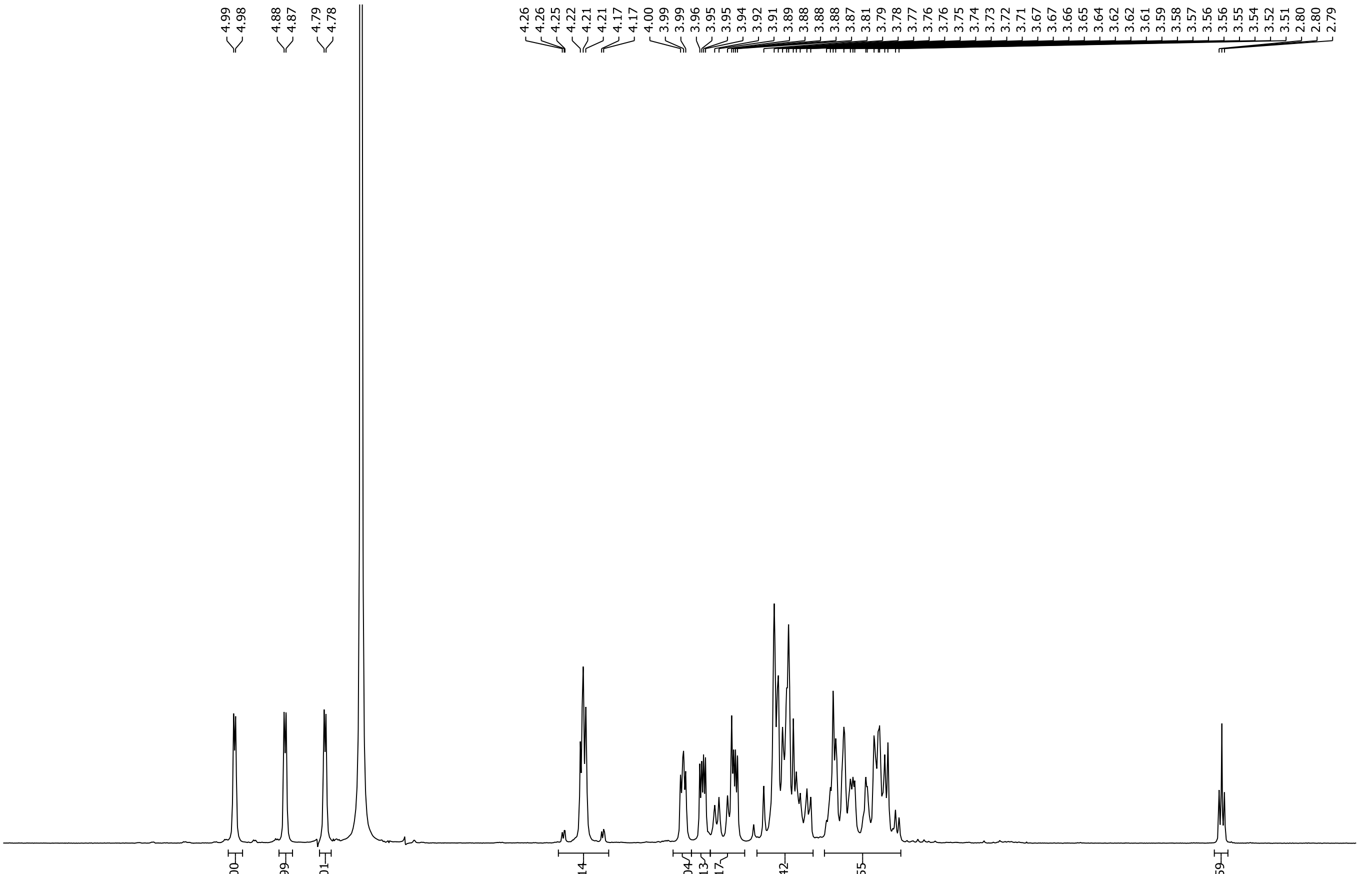

^{13}C APT NMR Spectrum

(Compound **14**)

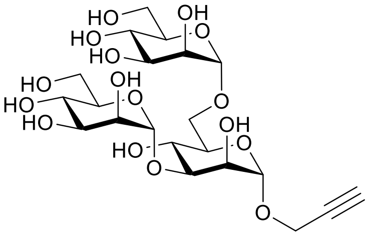

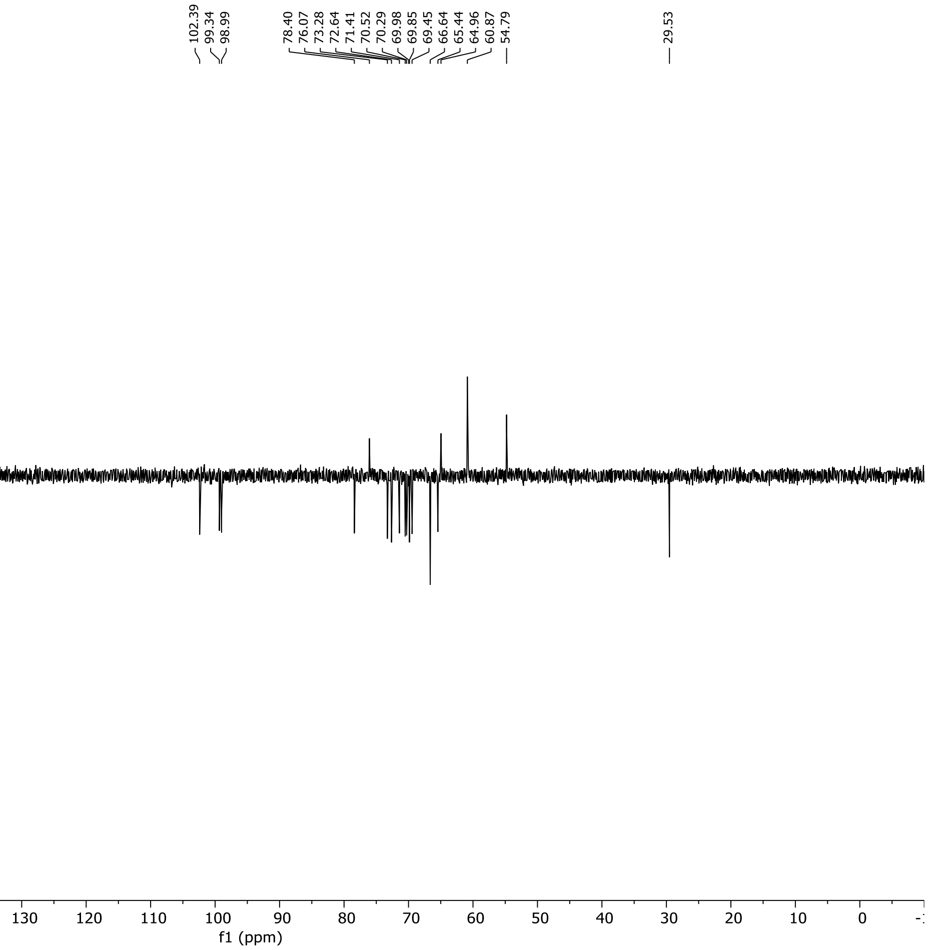

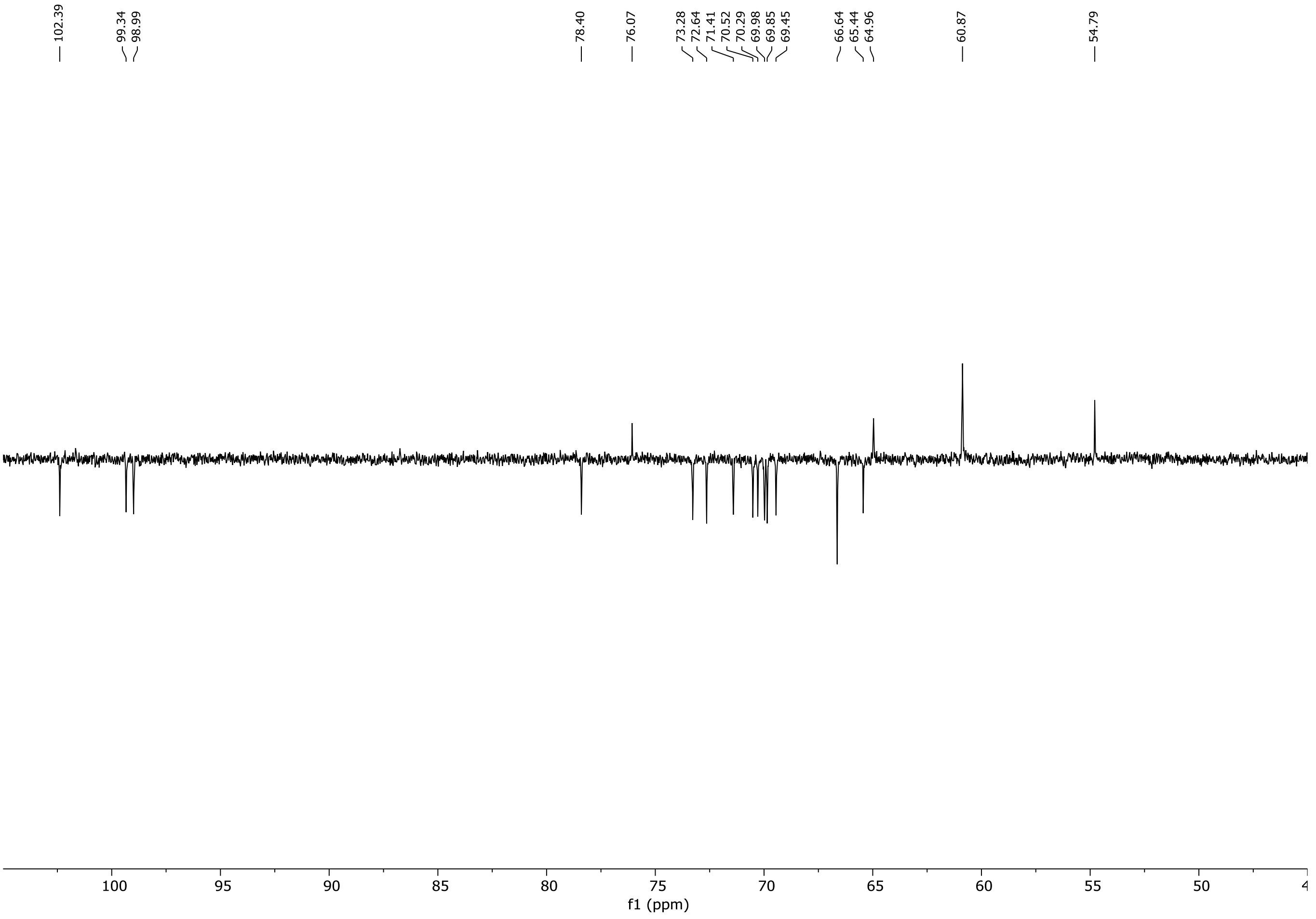

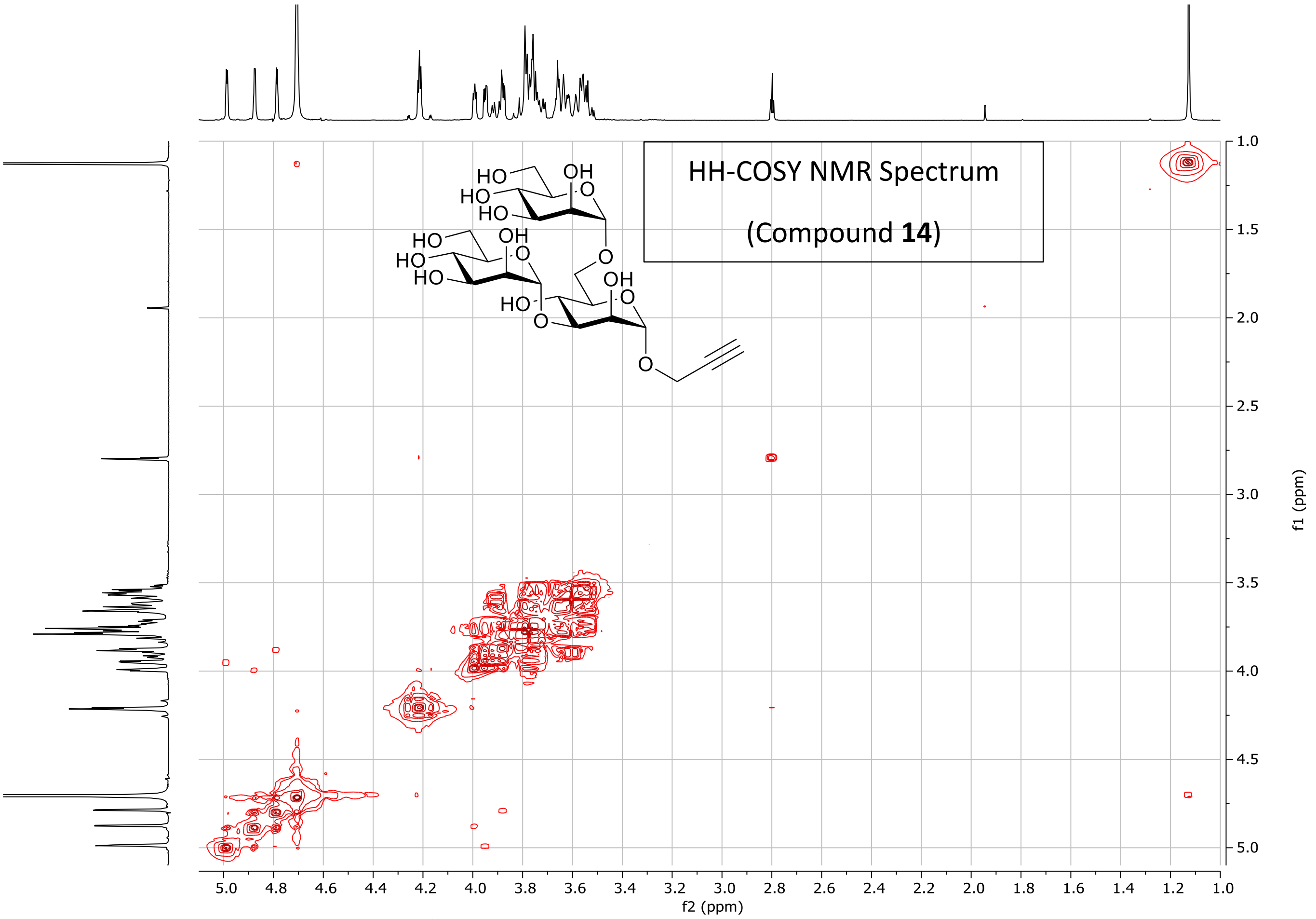
HH-COSY NMR Spectrum
(Compound **14**)
f2 (ppm)
f1 (ppm)

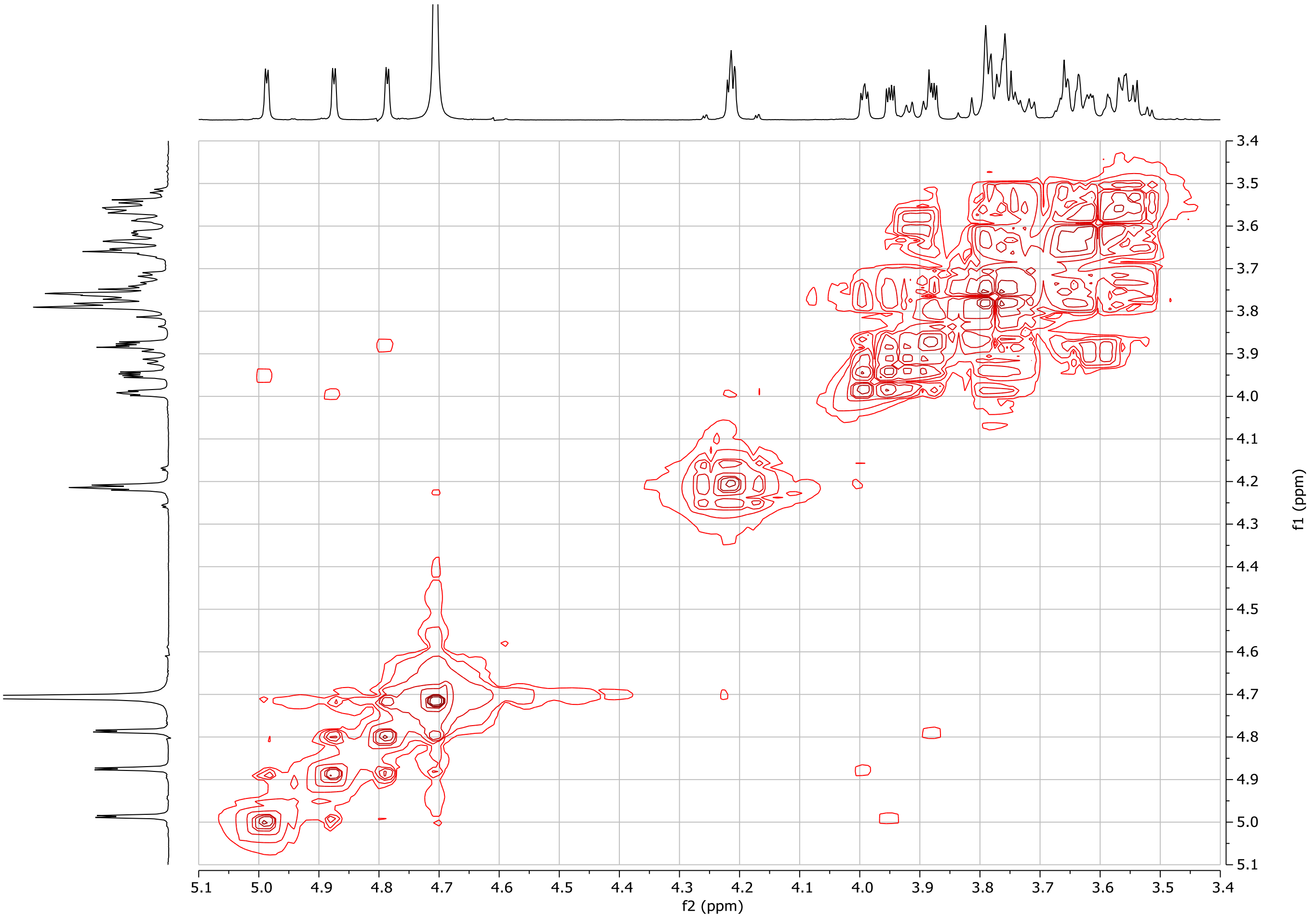
f2 (ppm)
f1 (ppm)

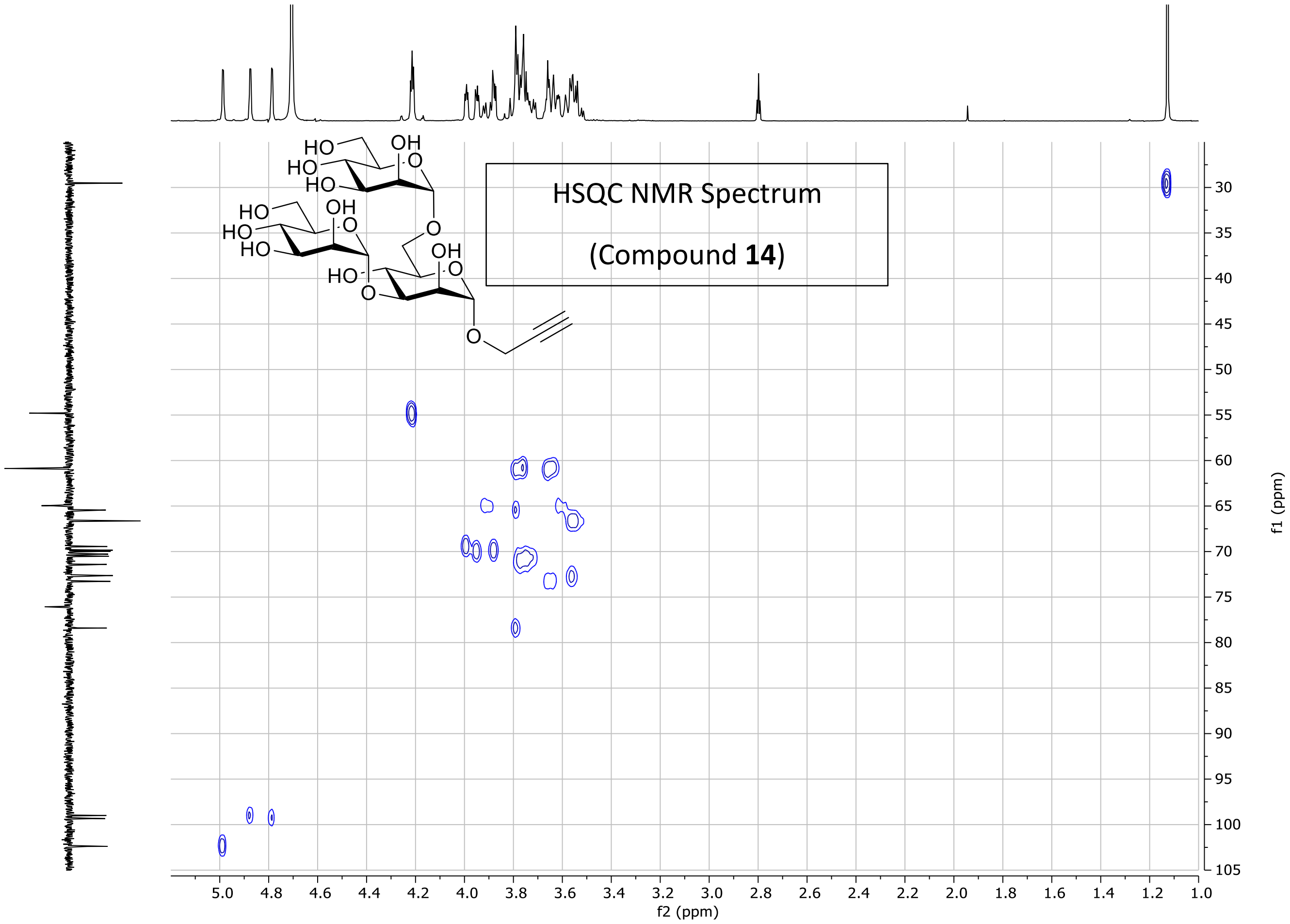
HSQC NMR Spectrum
(Compound **14**)
f2 (ppm)
f1 (ppm)

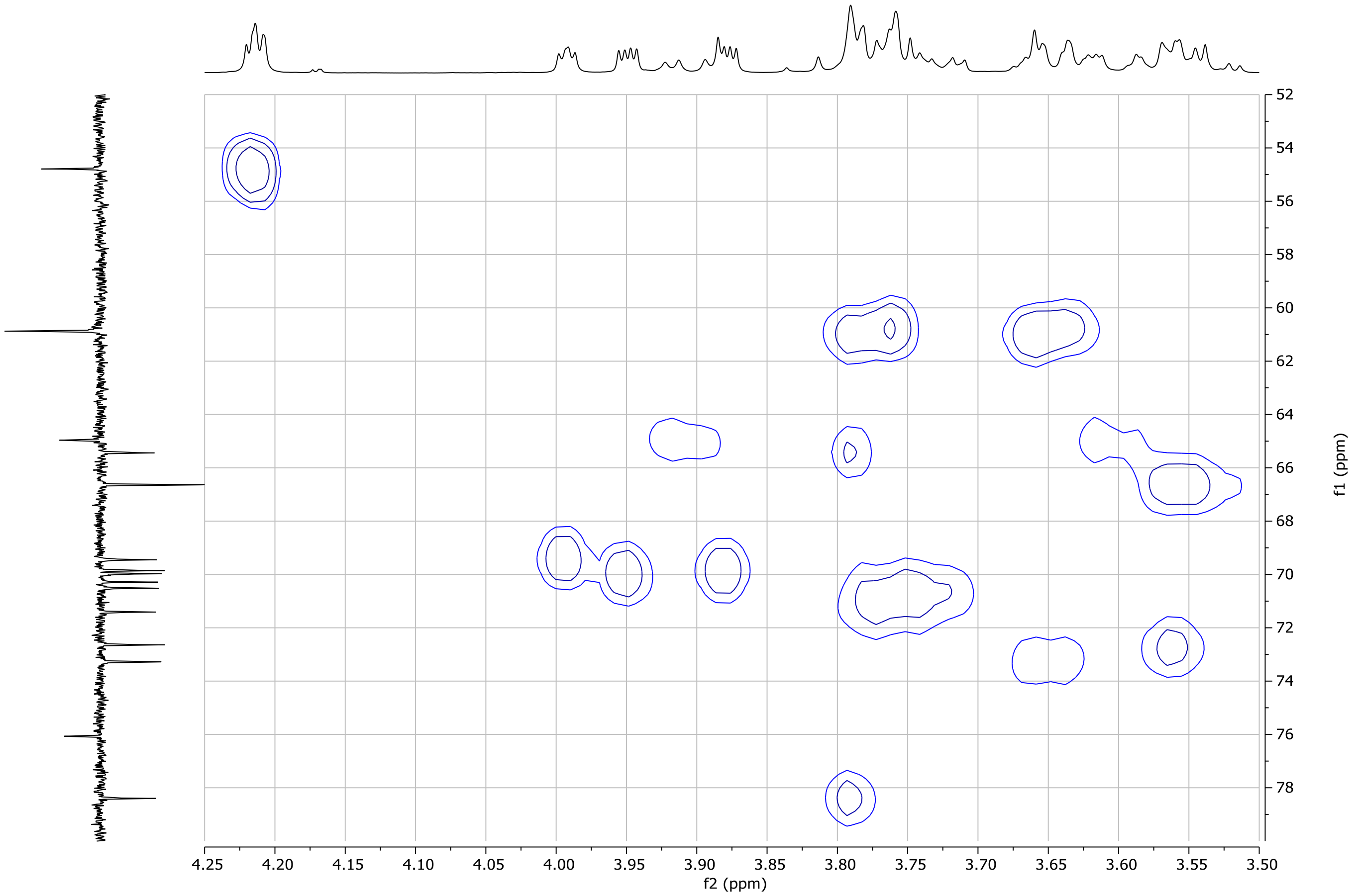
f2 (ppm)
f1 (ppm)

S8

^{1}H NMR Spectrum

(Compound **S8**)

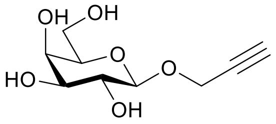

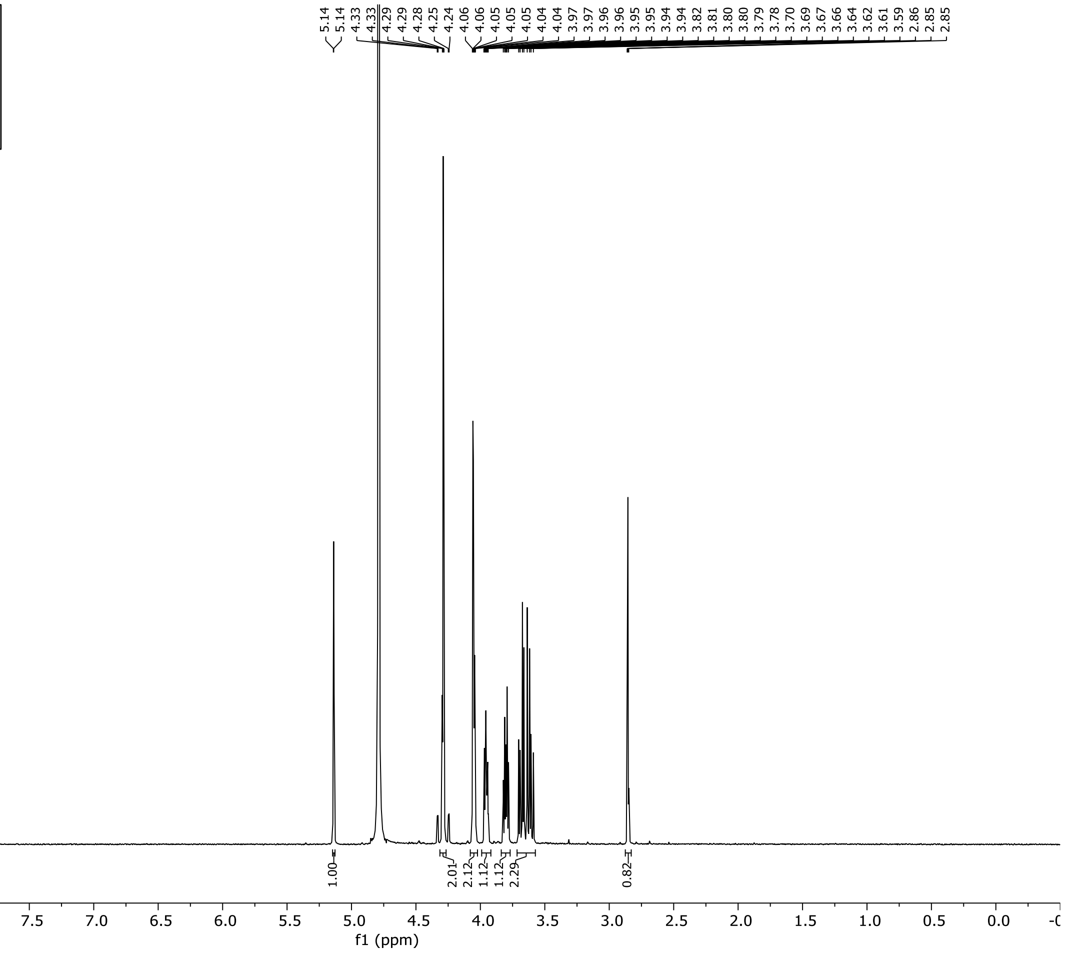

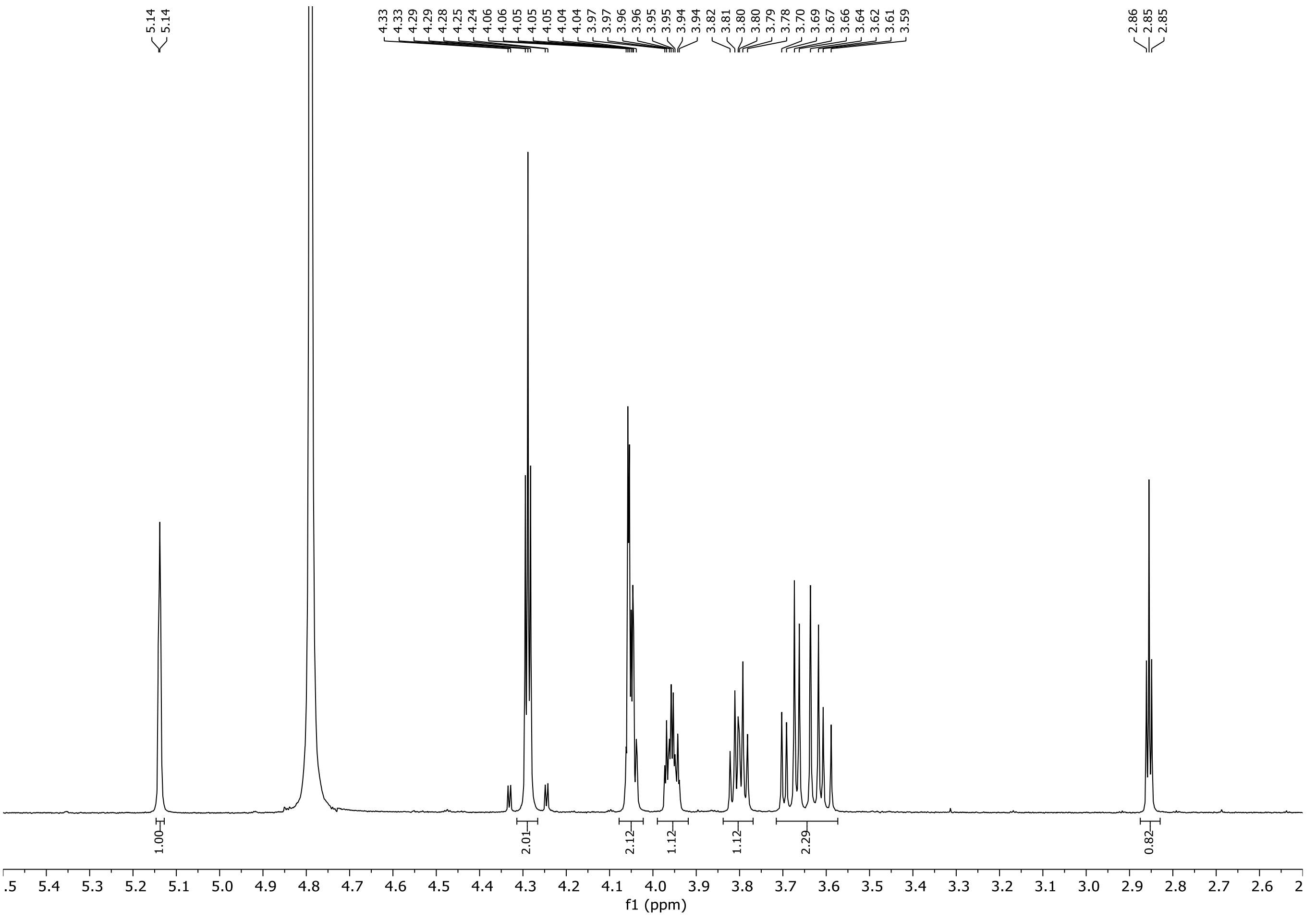

^{13}C APT NMR Spectrum

(Compound **S8**)

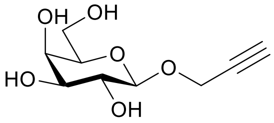

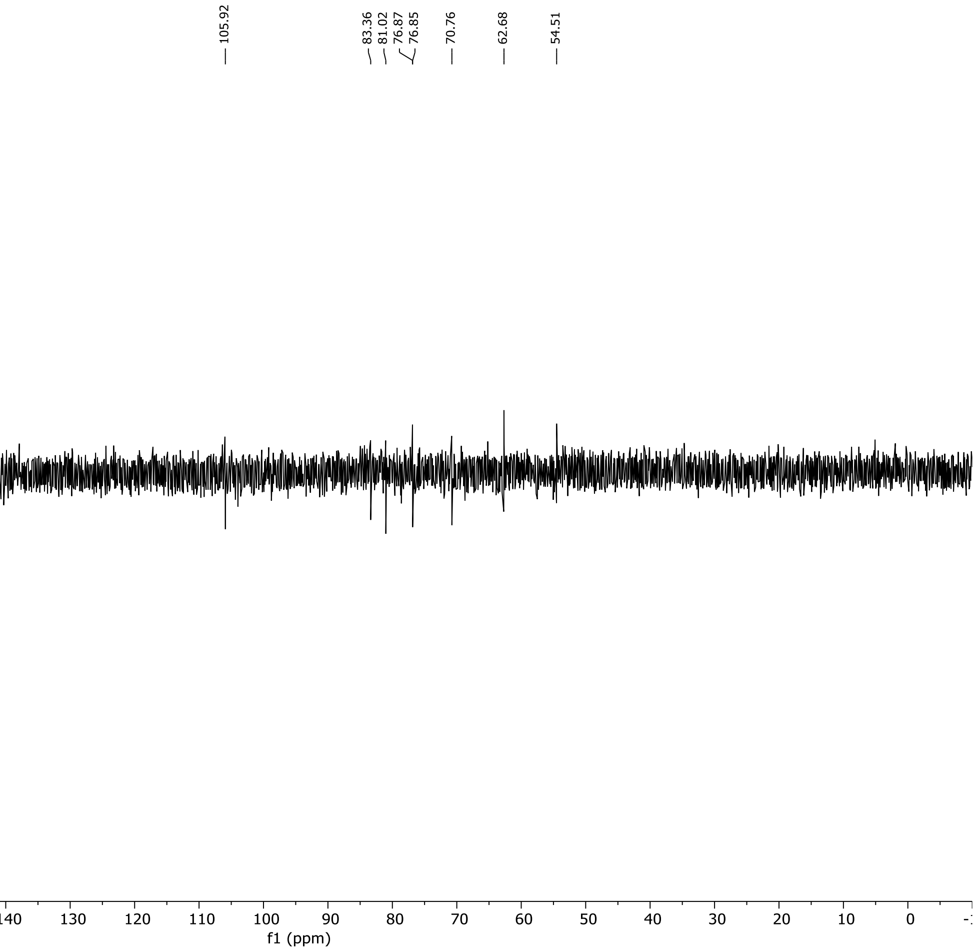

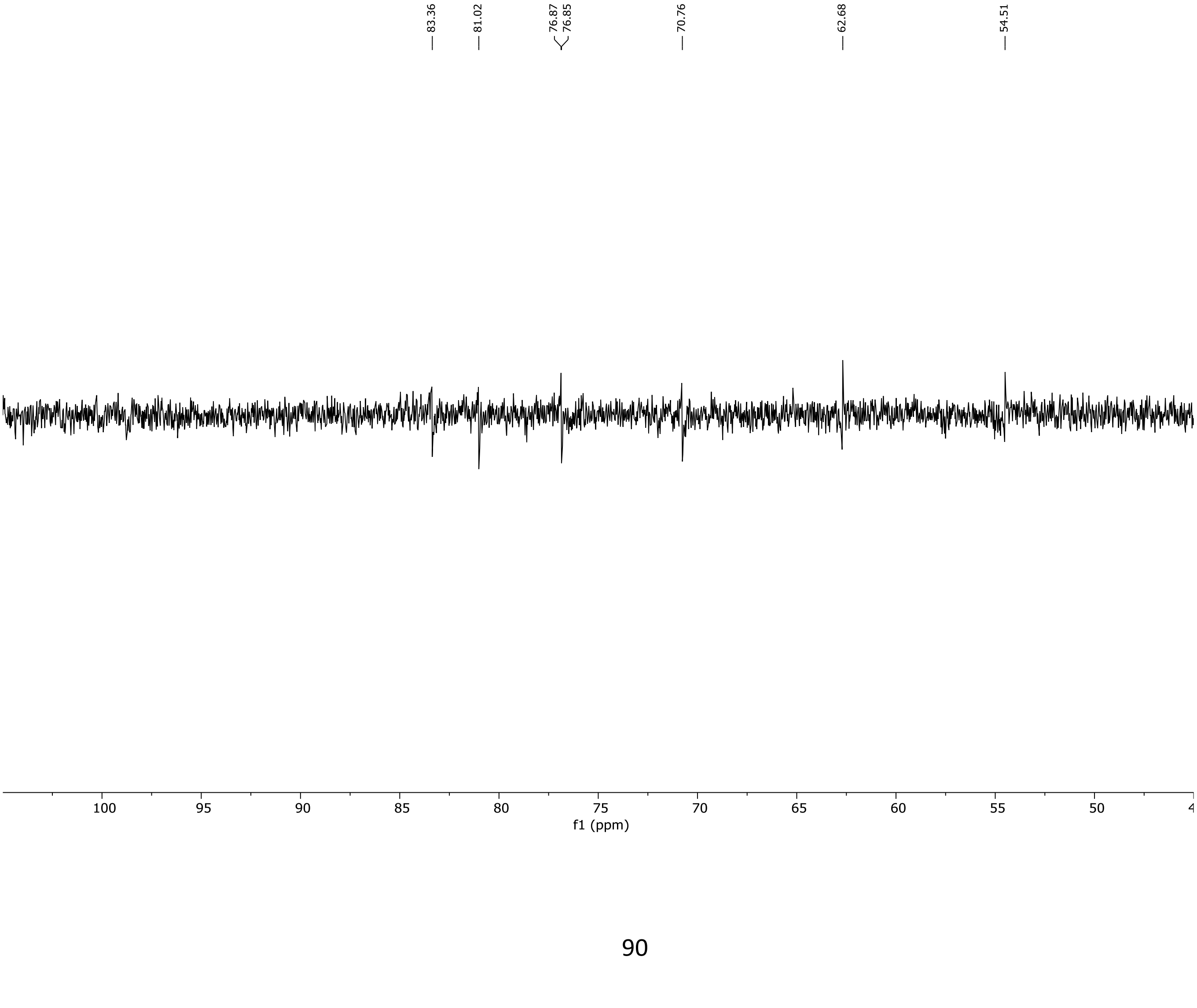

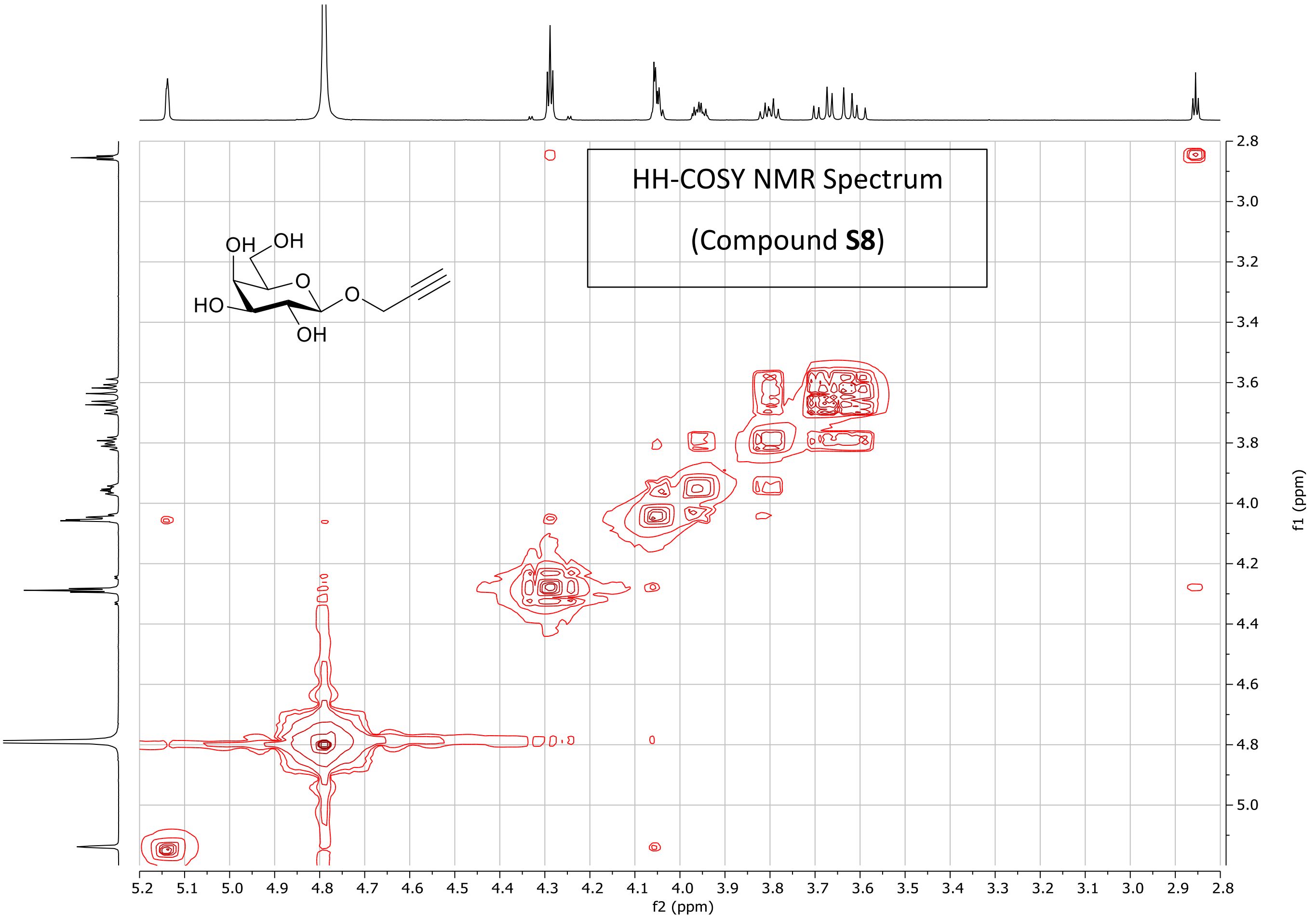

HH-COSY NMR Spectrum
(Compound **S8**)
OH
OH
O
O
HO
OH
f1 (ppm)
f2 (ppm)

Spectra of azido-backbones:

15

^{1}H NMR Spectrum

(Compound **15**)

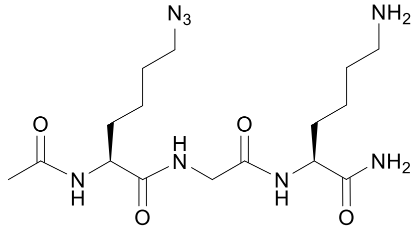

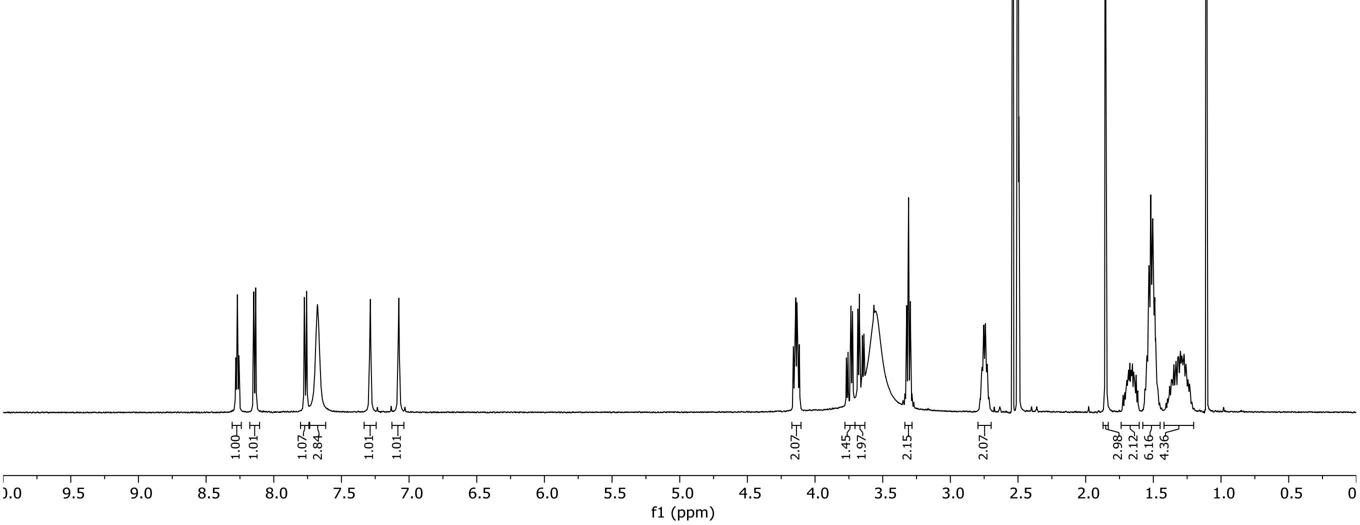

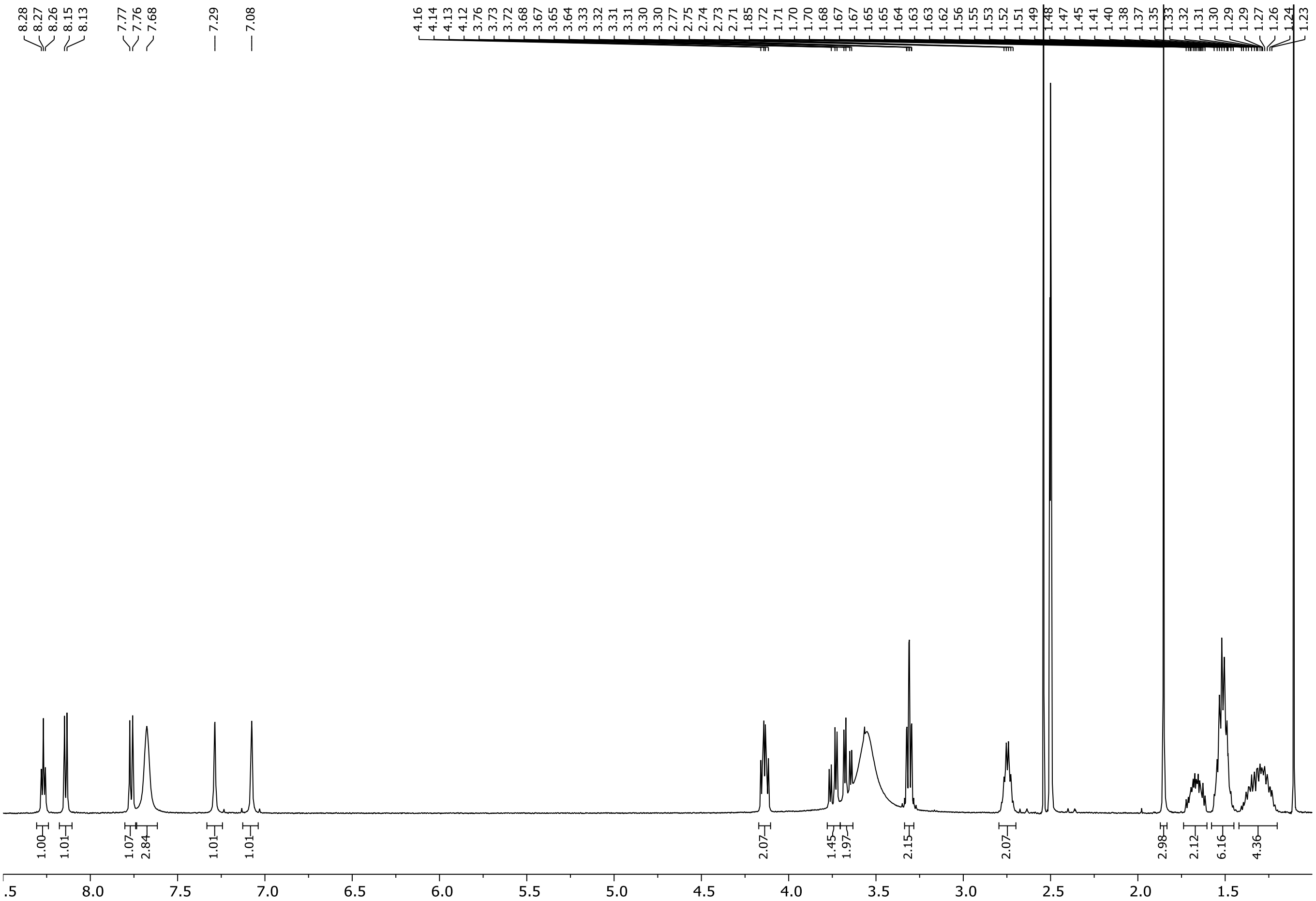

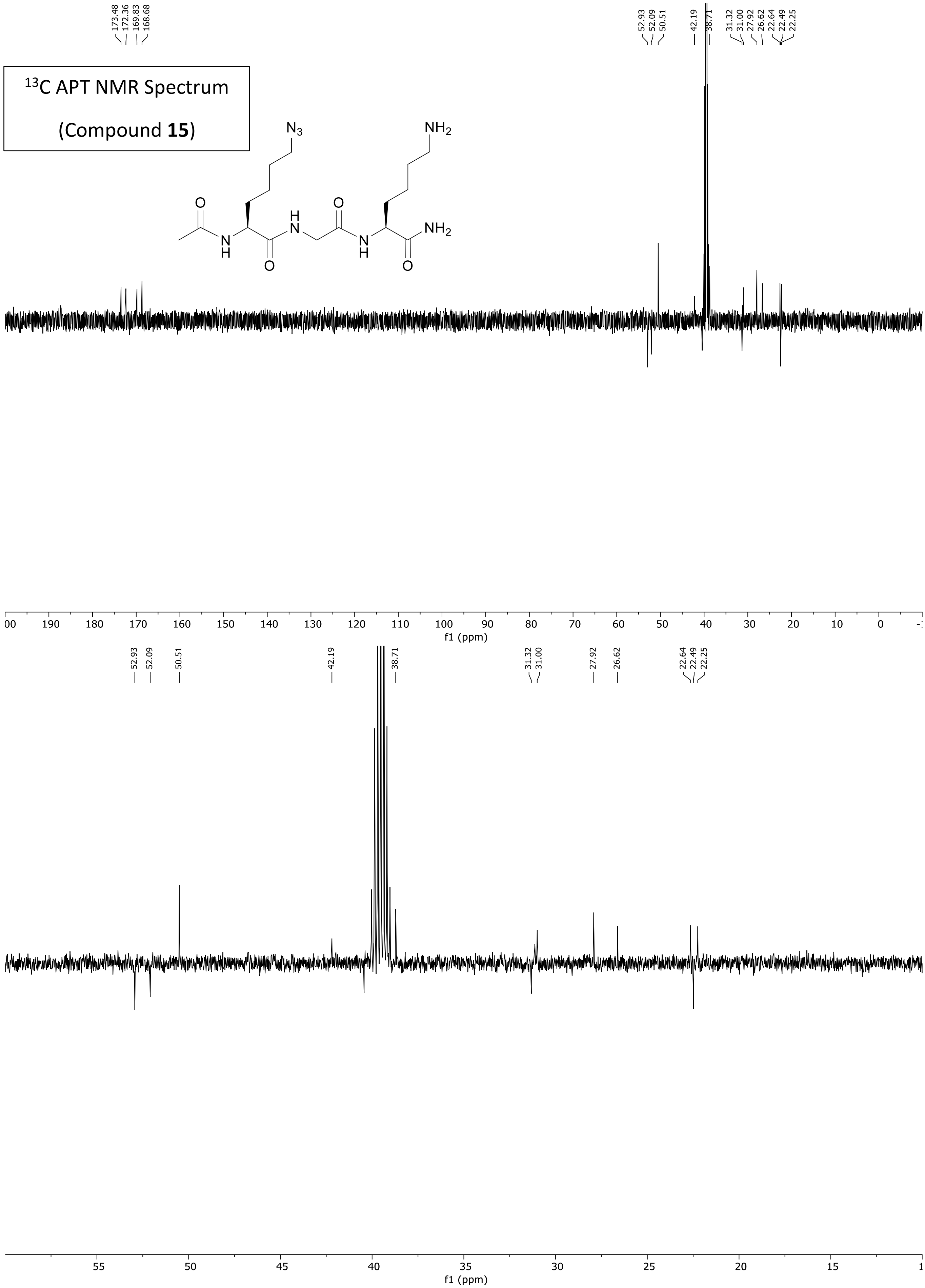
13C APT NMR Spectrum
(Compound 15)
N3
NH2
O
H
N
NH2
173.48
172.36
169.83
168.68
52.93
52.09
50.51
42.19
38.71
31.32
31.00
27.92
26.62
22.64
22.49
22.25
f1 (ppm)

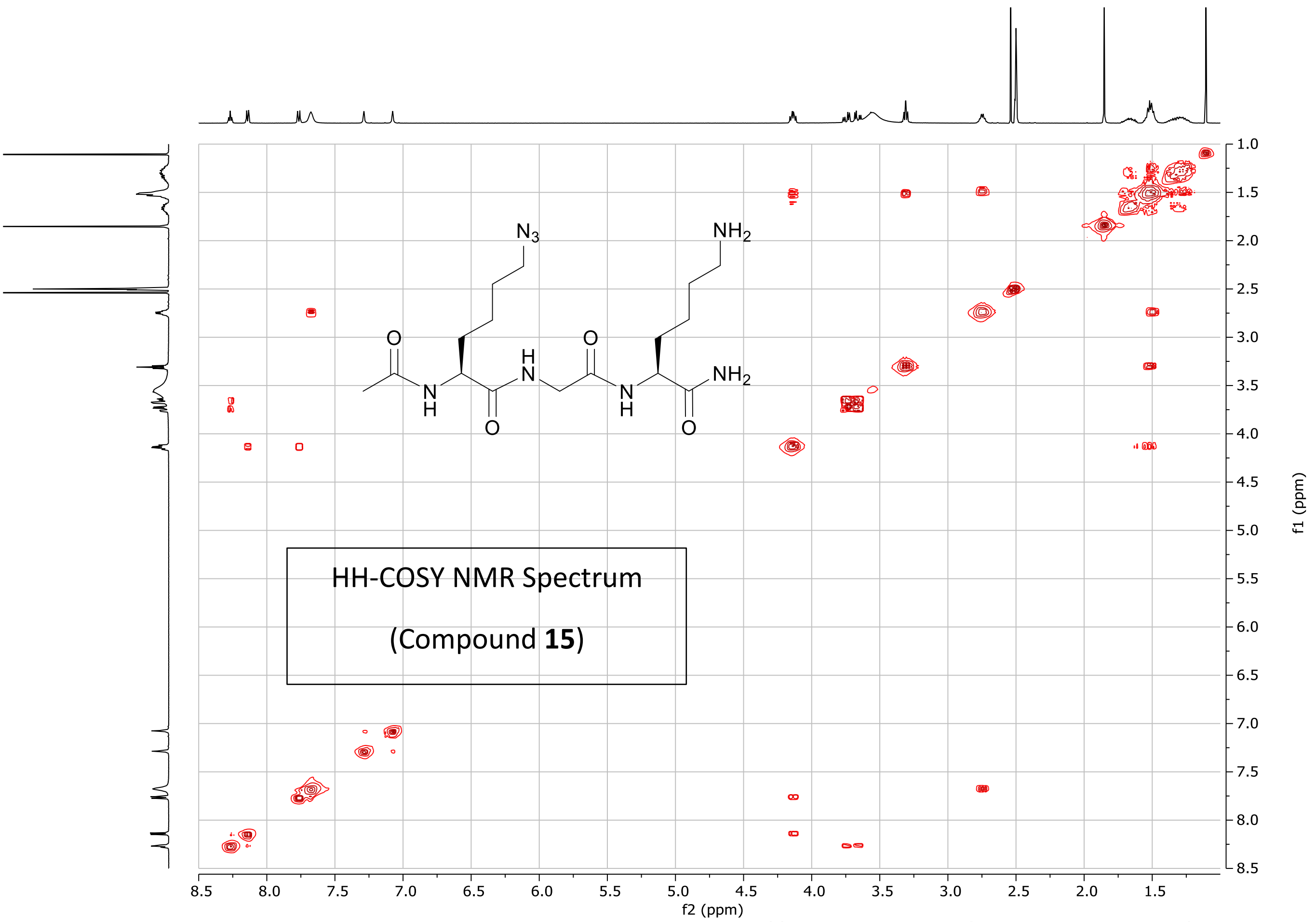
N3
NH2
O
H
N
O
NH2
N
H
O
N
H
O
HH-COSY NMR Spectrum
(Compound 15)
f1 (ppm)
f2 (ppm)

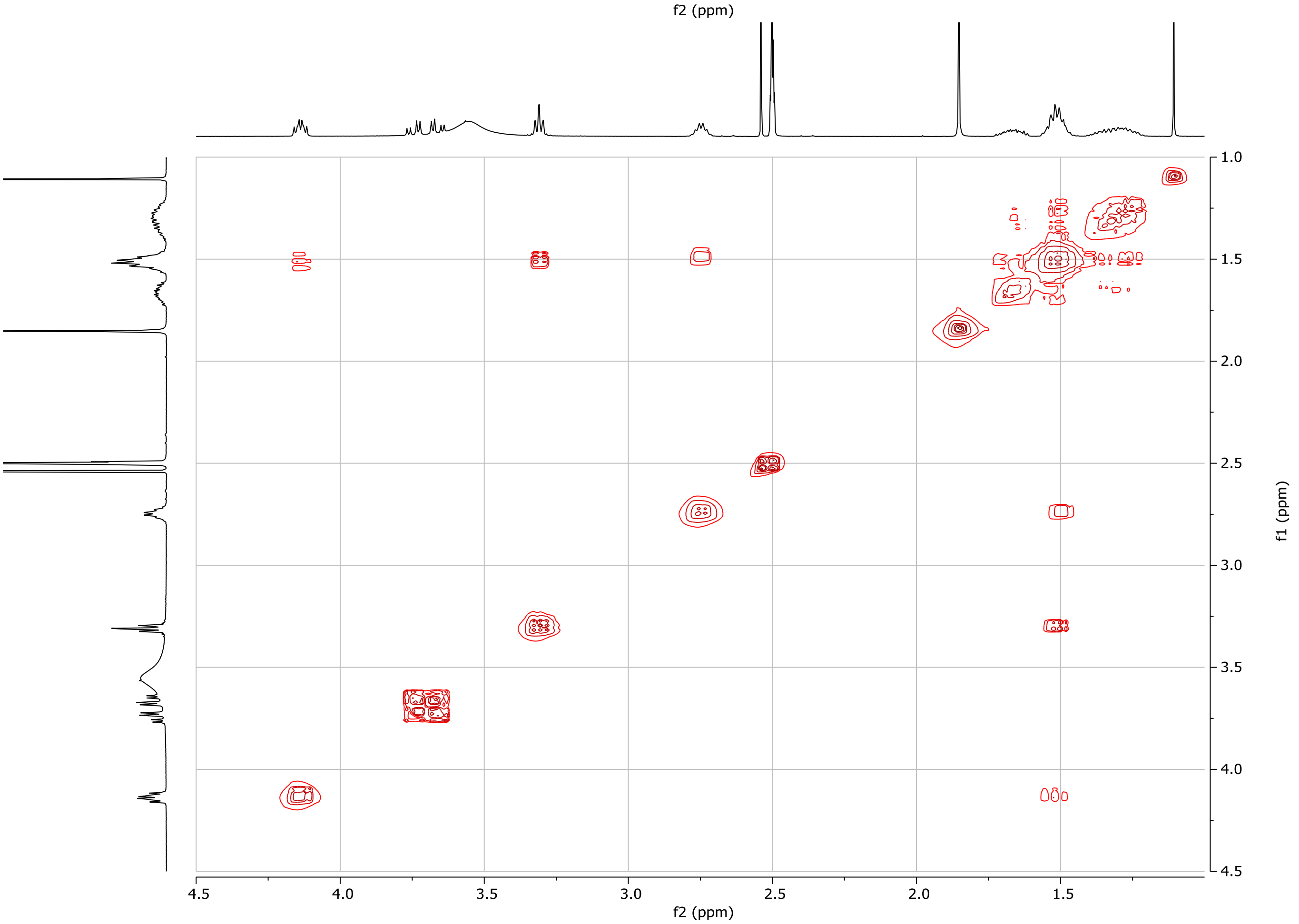
f1 (ppm)
f2 (ppm)

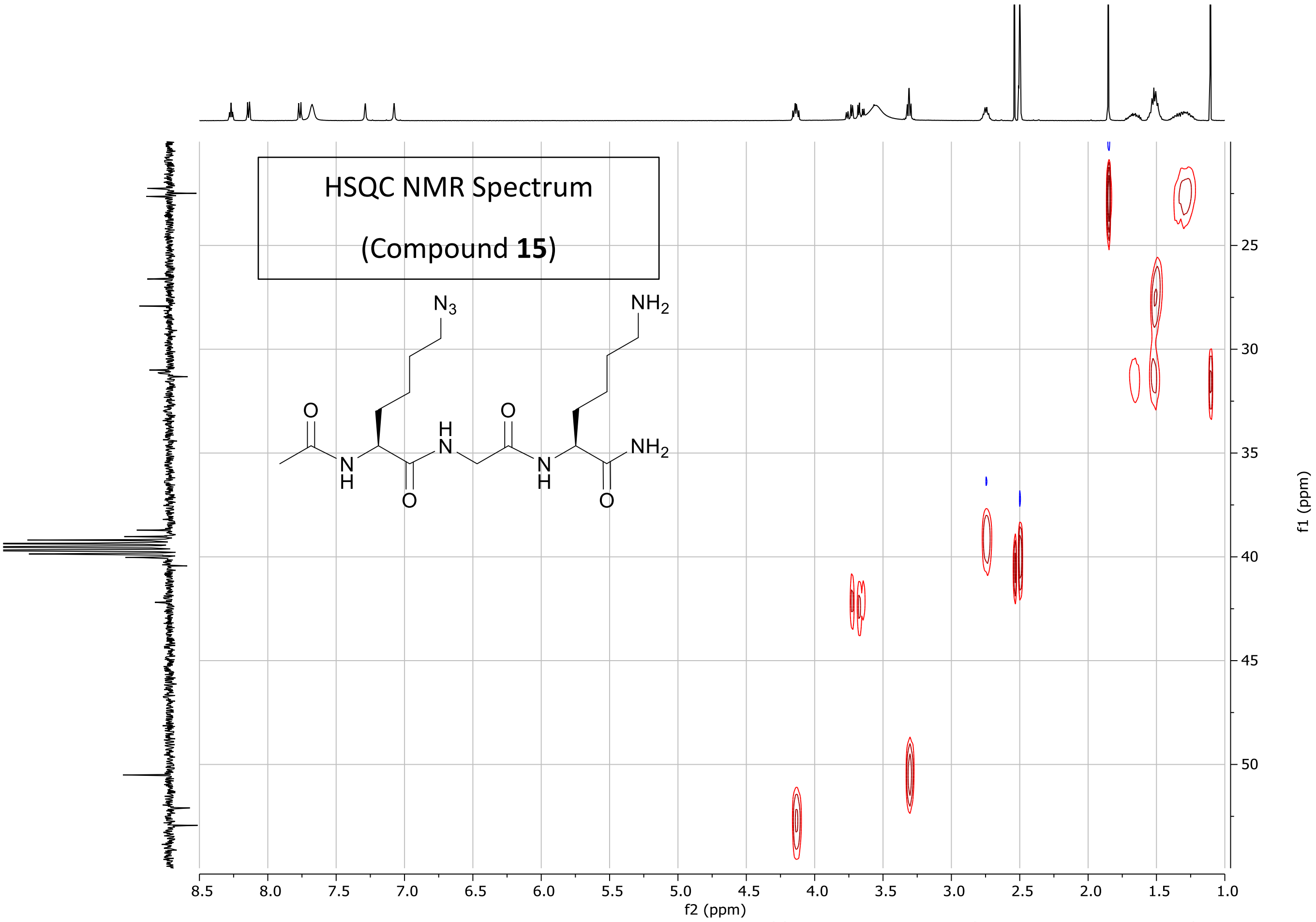
HSQC NMR Spectrum
(Compound **15**)
N3
NH2
O
H
N
NH2
f2 (ppm)
f1 (ppm)

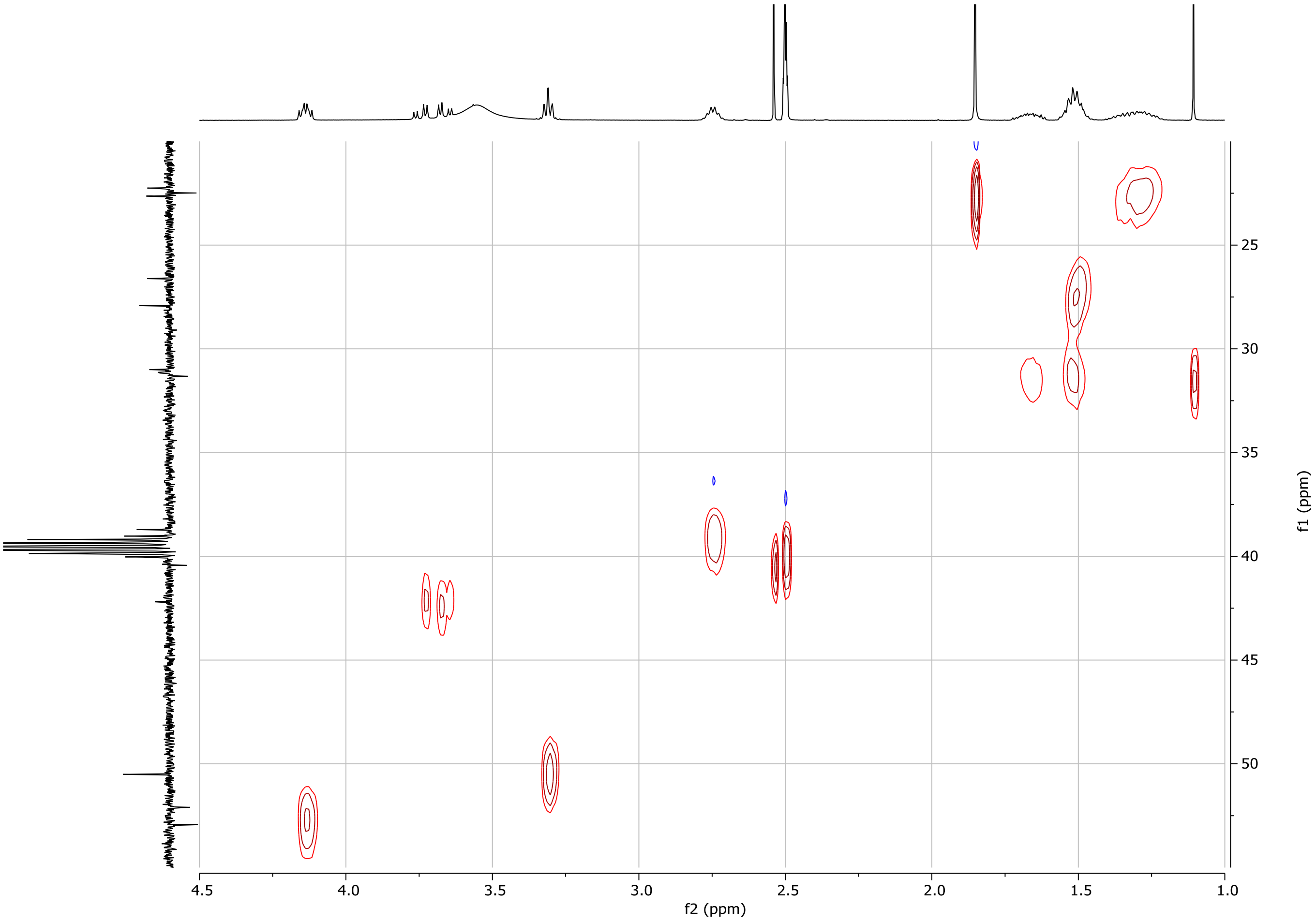
f2 (ppm)
f1 (ppm)

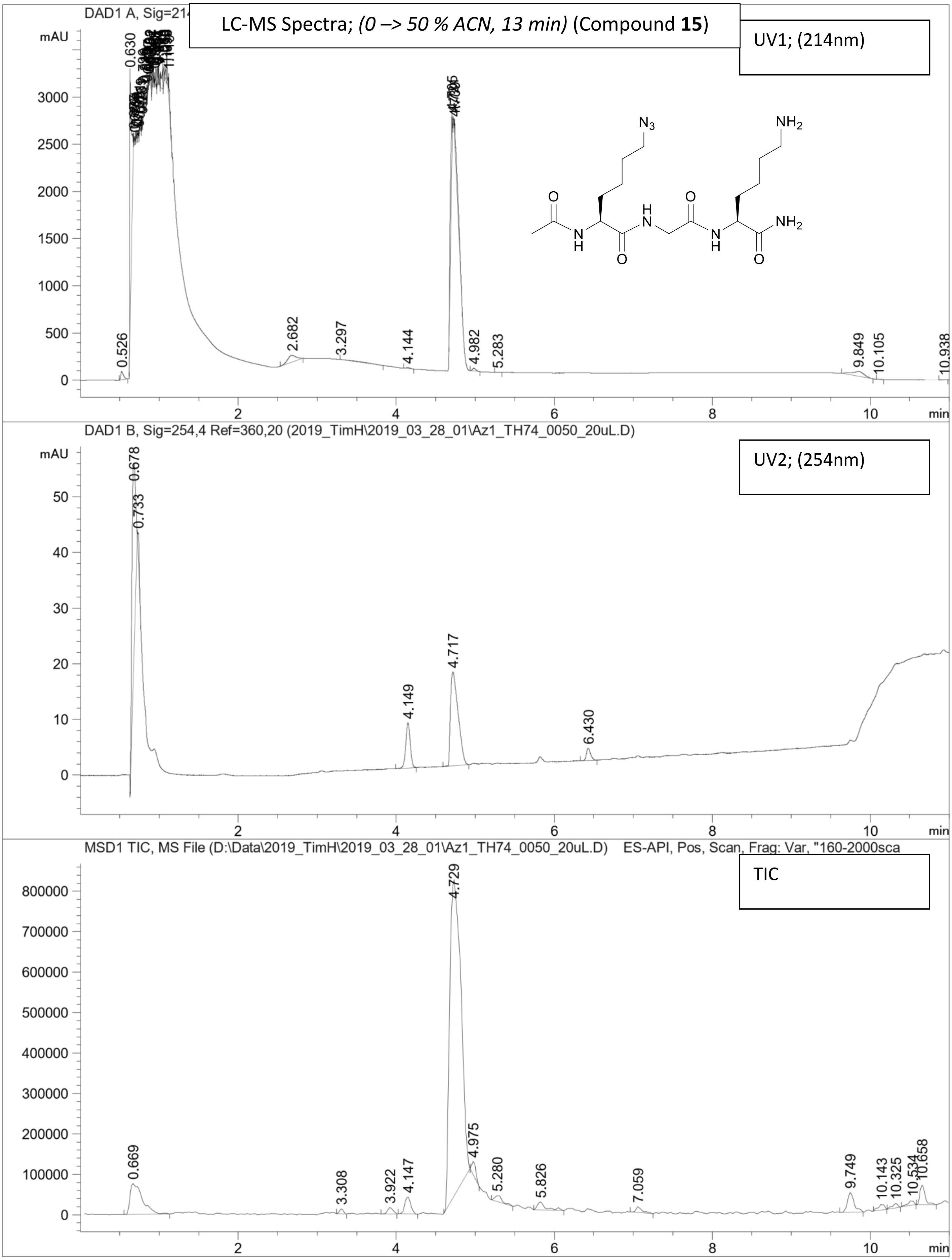
LC-MS Spectra; (0 –> 50 % ACN, 13 min) (Compound 15)
UV1; (214nm)
DAD1 A, Sig=214
mAU
0.630
0.526
2.682
3.297
4.144
4.982
5.283
9.849
10.105
10.938
min
N3
NH2
O
H
N
NH2
UV2; (254nm)
DAD1 B, Sig=254,4 Ref=360,20 (2019_TimH\2019_03_28_01\Az1_TH74_0050_20uL.D)
0.678
0.733
4.149
4.717
6.430
TIC
MSD1 TIC, MS File (D:\Data\2019_TimH\2019_03_28_01\Az1_TH74_0050_20uL.D) ES-API, Pos, Scan, Frag: Var, "160-2000sca
4.729
0.669
3.308
3.922
4.147
4.975
5.280
5.826
7.059
9.749
10.143
10.325
10.534
10.658

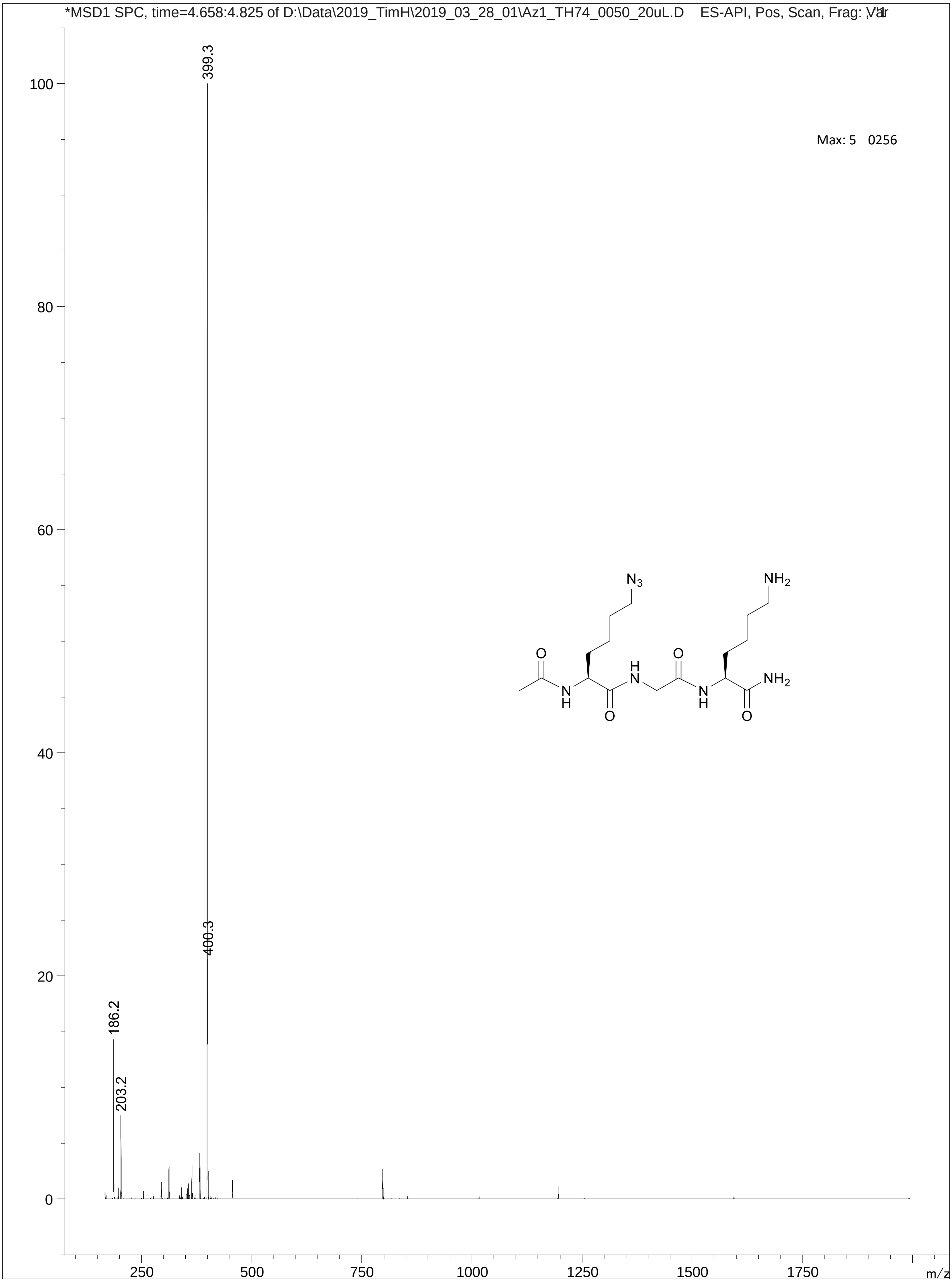
*MSD1 SPC, time=4.658:4.825 of D:\Data\2019_TimH\2019_03_28_01\Az1_TH74_0050_20uL.D ES-API, Pos, Scan, Frag: Var
Max: 5 0256
399.3
400.3
186.2
203.2
0
20
40
60
80
100
250
500
750
1000
1250
1500
1750
m/z
N3
NH2
O
H
N
NH2
O

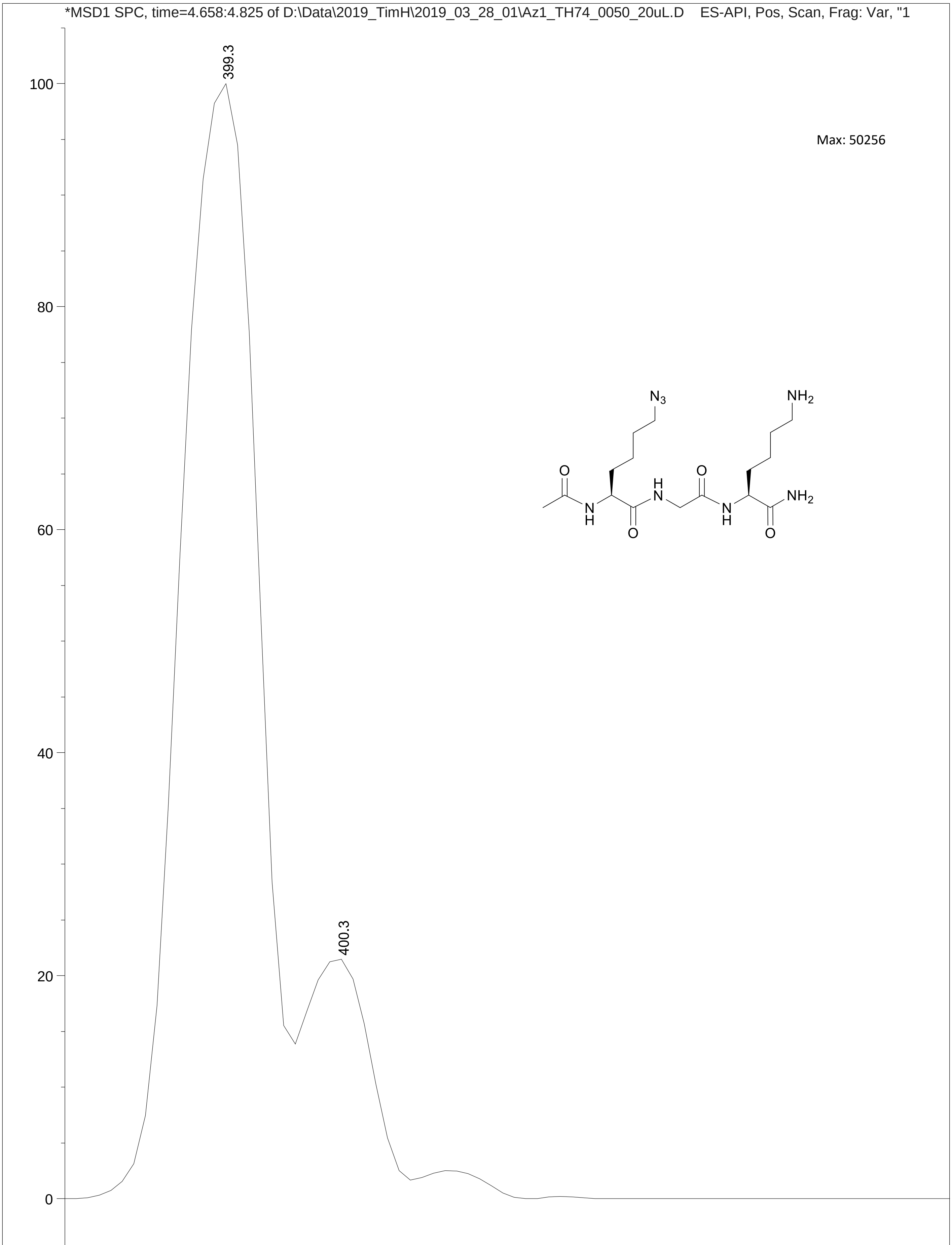
*MSD1 SPC, time=4.658:4.825 of D:\Data\2019_TimH\2019_03_28_01\Az1_TH74_0050_20uL.D ES-API, Pos, Scan, Frag: Var, "1
399.3
400.3
Max: 50256
100
80
60
40
20
0
398
399
400
401
402
403
404
405
m/z
N3
NH2
O
H
N
N
H
O
O
N
H
NH2
O

16

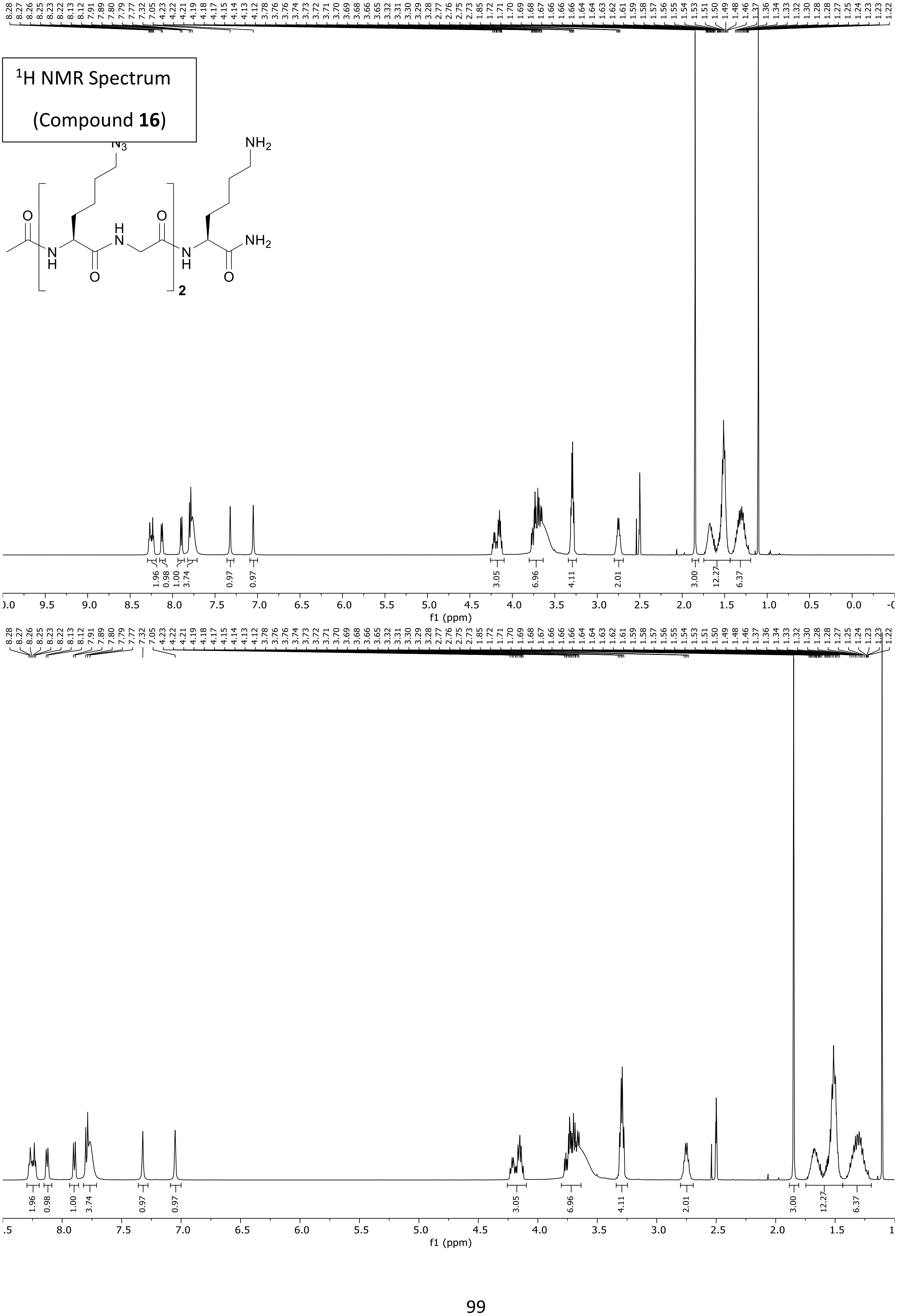

^{1}H NMR Spectrum
(Compound **16**)
N3
NH2
O
H
N
O
N
H
O
N
H
O
NH2
2
1.96
0.98
1.00
3.74
0.97
0.97
3.05
6.96
4.11
2.01
3.00
12.27
6.37
f1 (ppm)

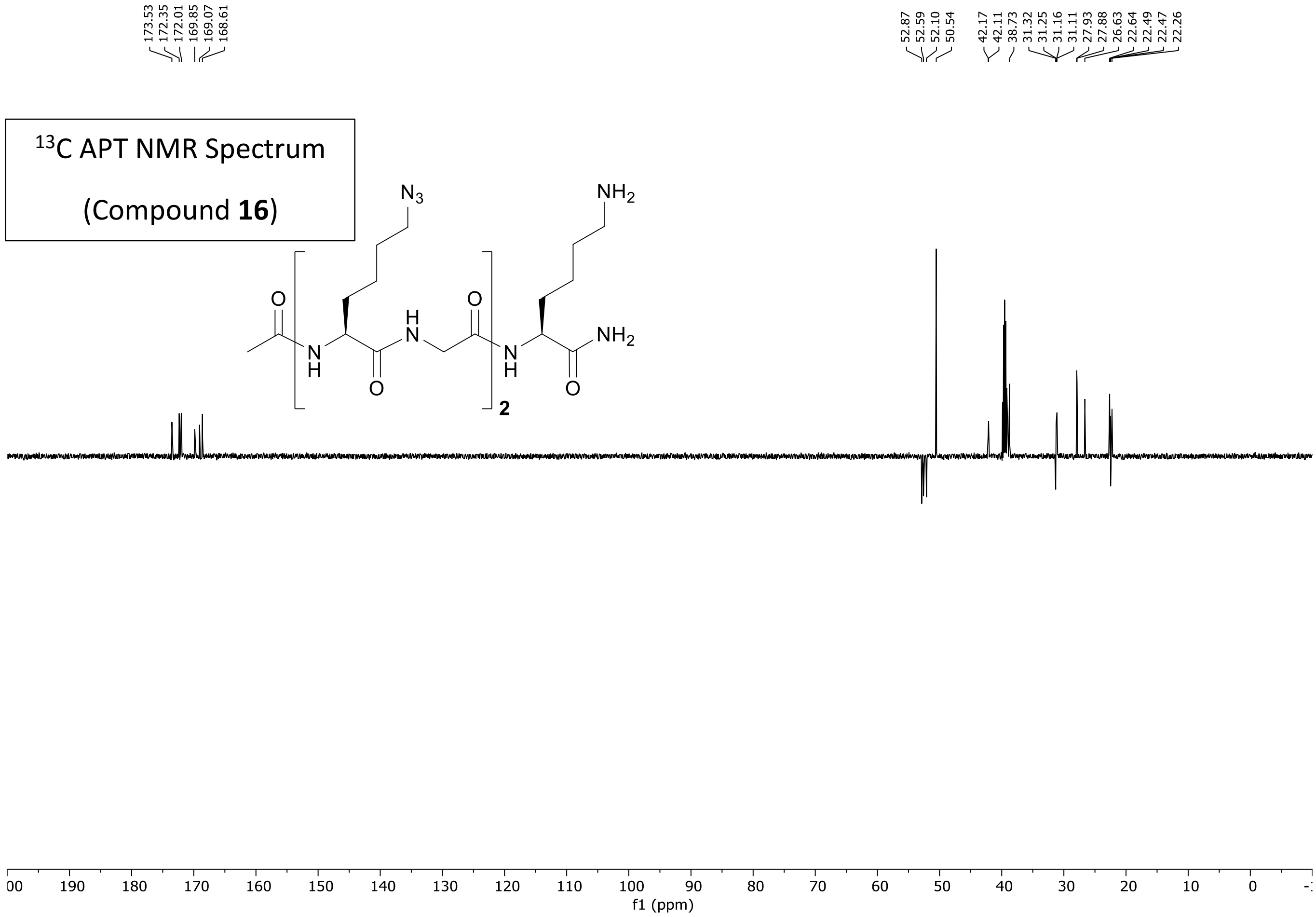
13C APT NMR Spectrum
(Compound 16)
173.53 172.35 172.01 169.85 169.07 168.61
52.87 52.59 52.10 50.54 42.17 42.11 38.73 31.32 31.25 31.16 31.11 27.93 27.88 26.63 22.64 22.49 22.47 22.26
N3
NH2
O
H
N
NH2
2
00 190 180 170 160 150 140 130 120 110 100 90 80 70 60 50 40 30 20 10 0
f1 (ppm)

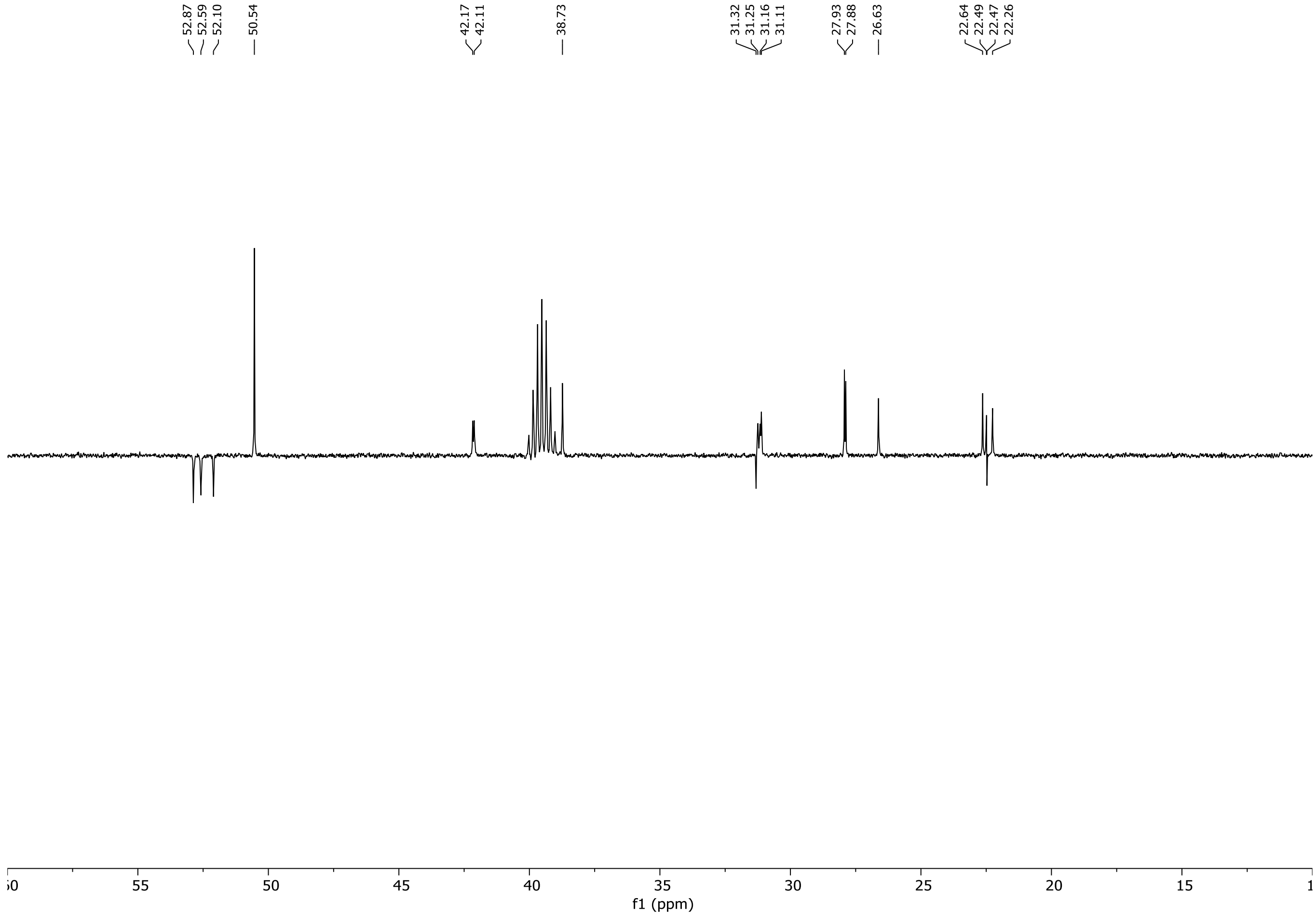
52.87 52.59 52.10 50.54 42.17 42.11 38.73 31.32 31.25 31.16 31.11 27.93 27.88 26.63 22.64 22.49 22.47 22.26
55 50 45 40 35 30 25 20 15
f1 (ppm)

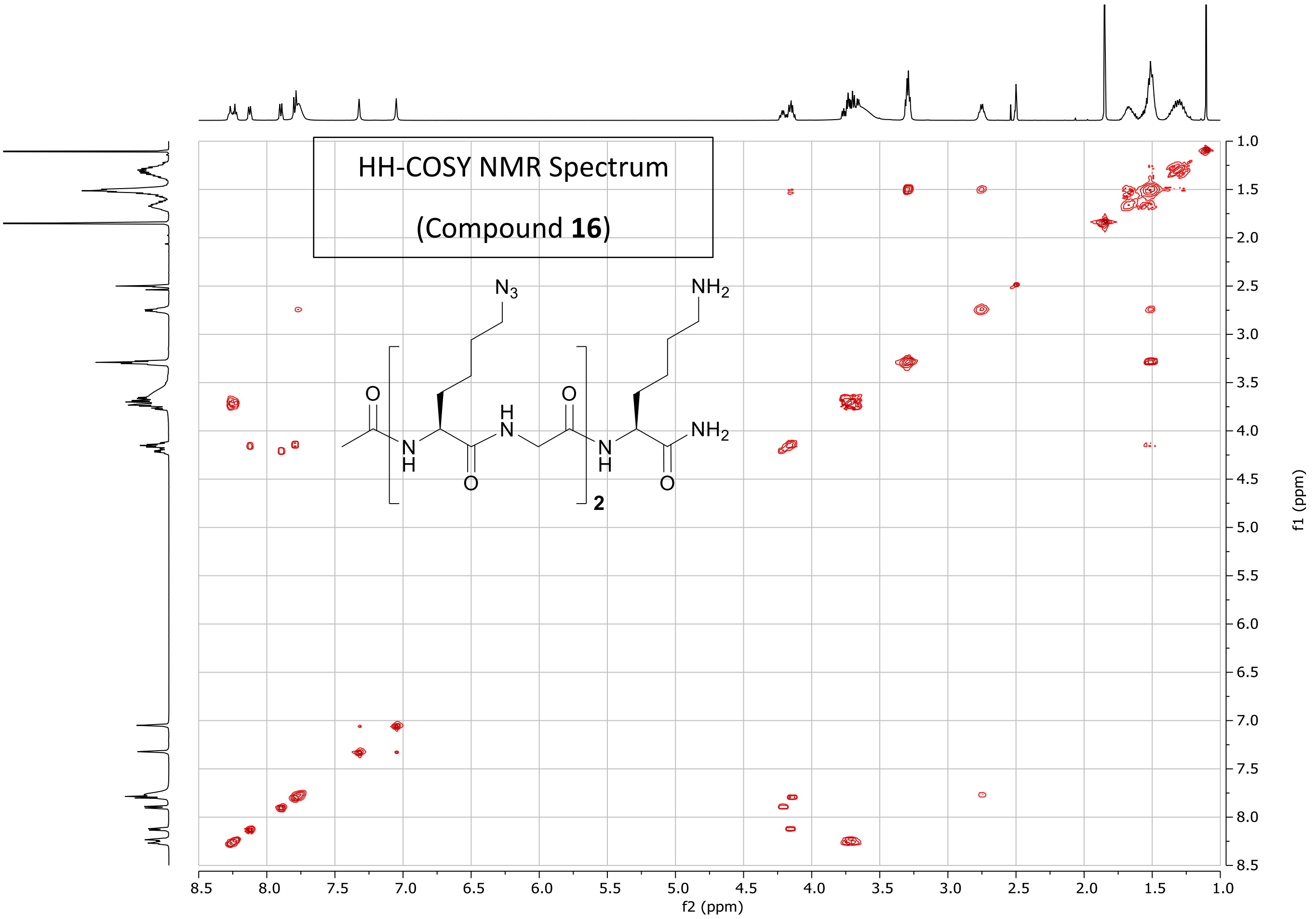
HH-COSY NMR Spectrum
(Compound 16)
N3
NH2
O
H
N
NH2
2
f2 (ppm)
f1 (ppm)

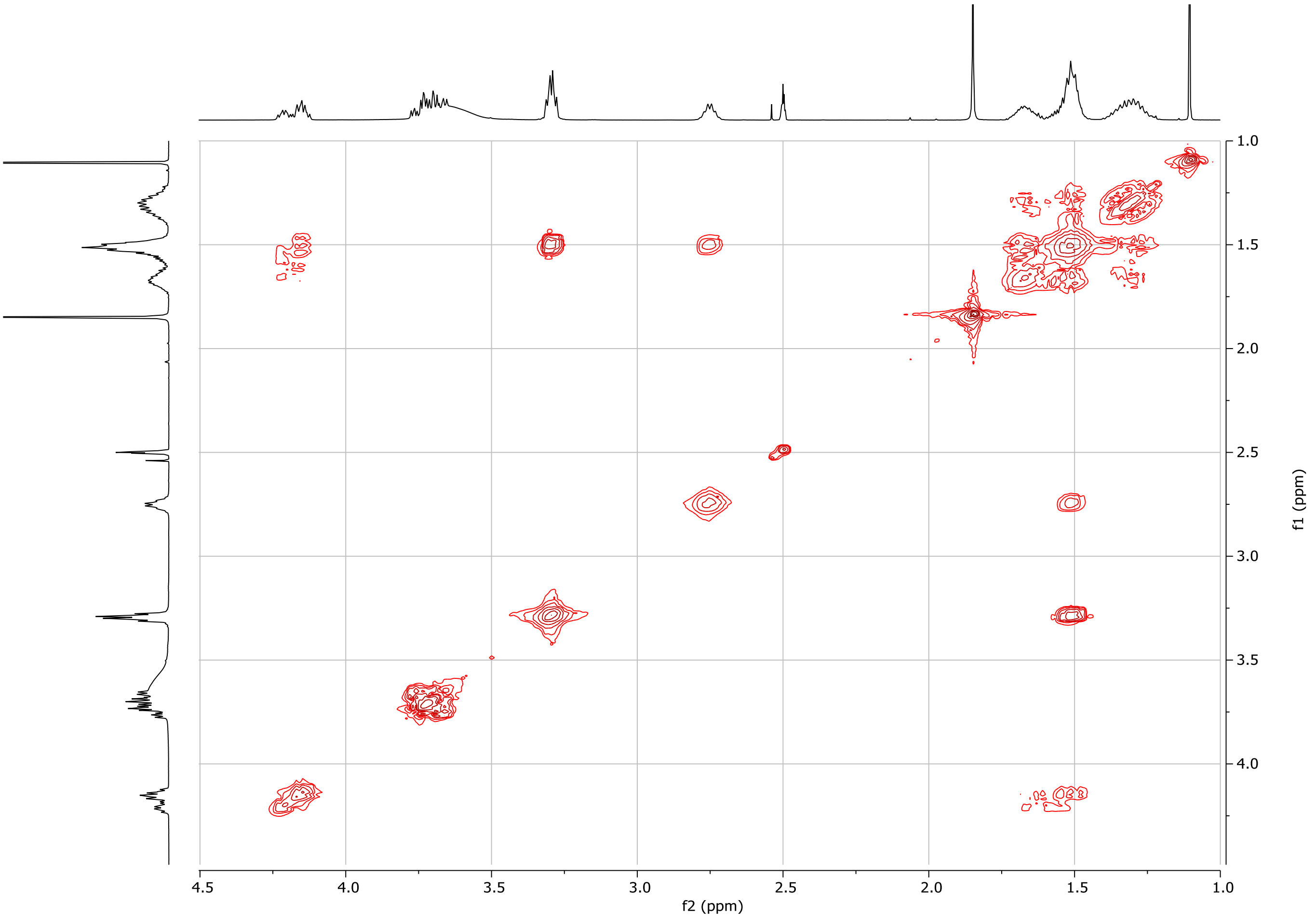
f2 (ppm)
f1 (ppm)

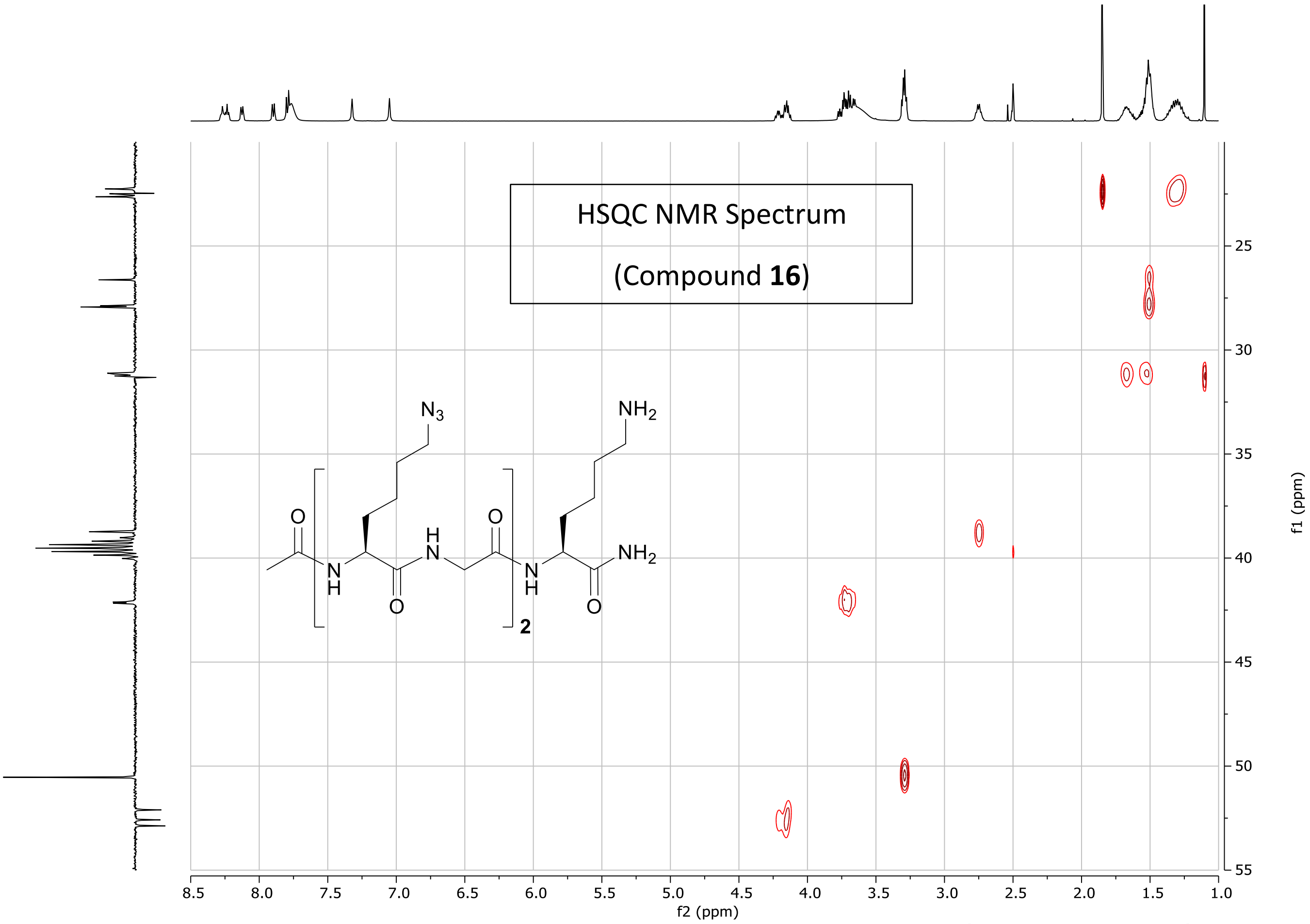
HSQC NMR Spectrum
(Compound 16)
N3
NH2
O
N
H
H
N
O
O
N
H
NH2
O
2
f2 (ppm)
f1 (ppm)

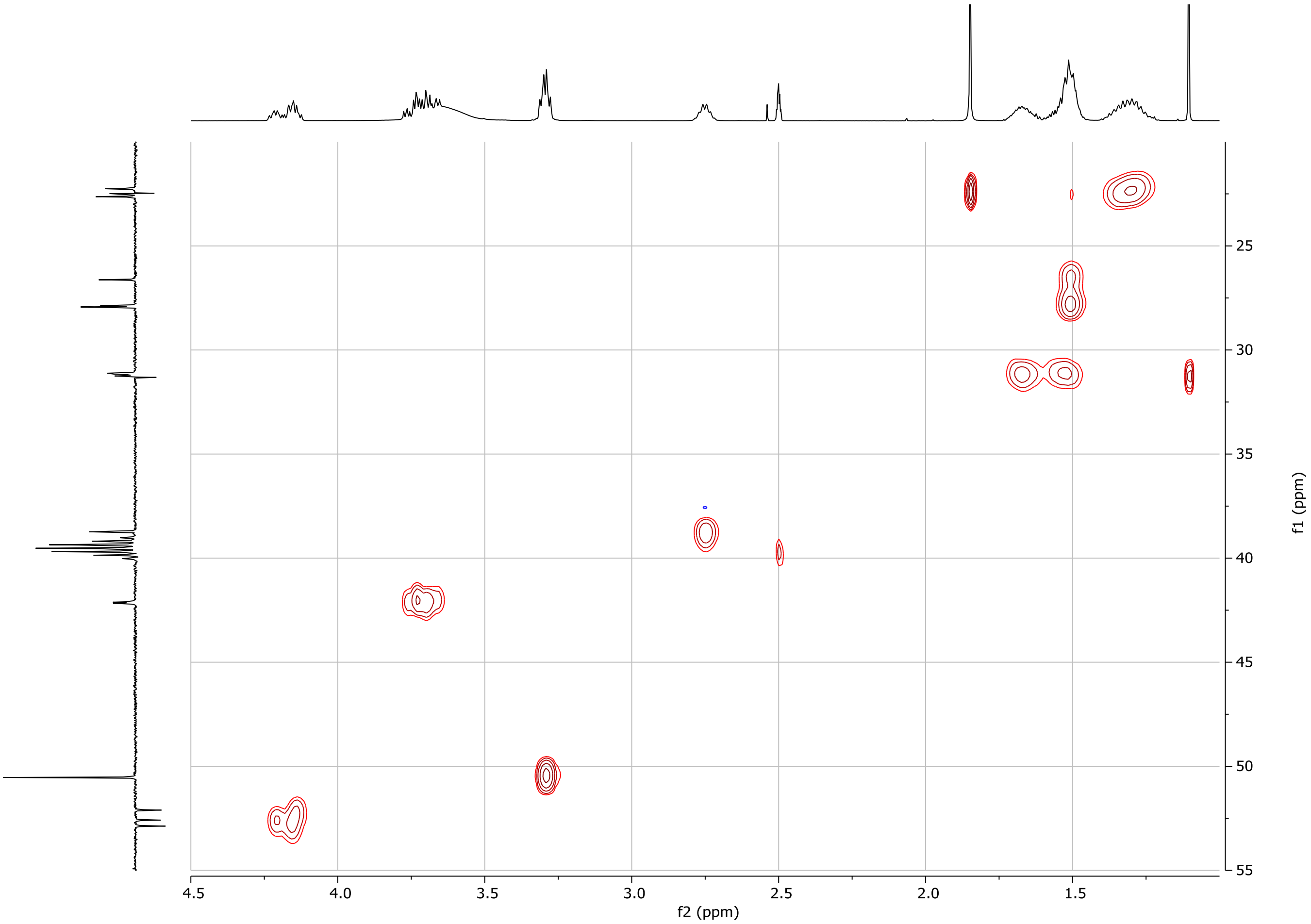
f2 (ppm)
f1 (ppm)

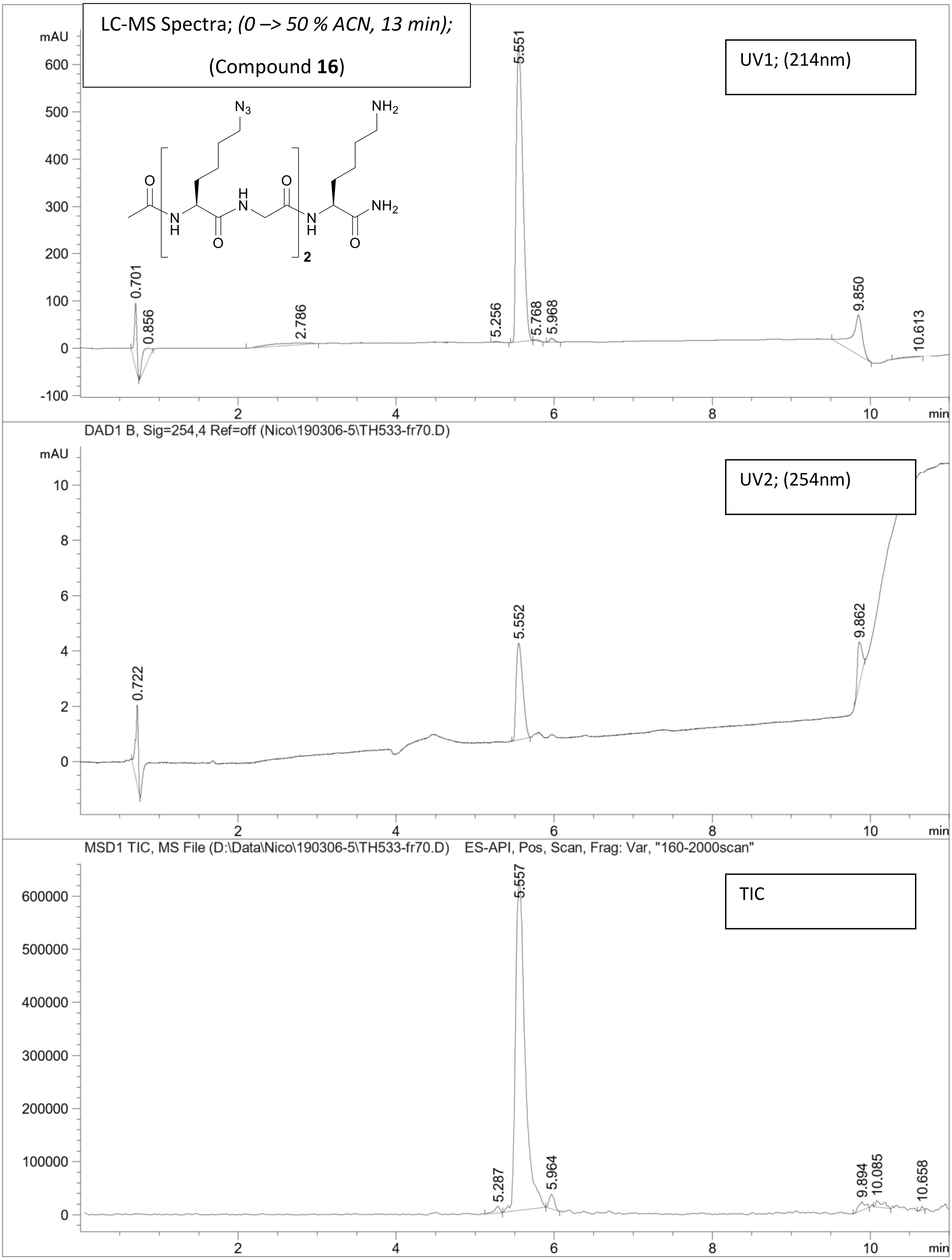
LC-MS Spectra; (0 –> 50 % ACN, 13 min);
(Compound 16)
UV1; (214nm)
mAU
5.551
0.701
0.856
2.786
5.256
5.768
5.968
9.850
10.613
min
DAD1 B, Sig=254,4 Ref=off (Nico\190306-5\TH533-fr70.D)
UV2; (254nm)
0.722
5.552
9.862
MSD1 TIC, MS File (D:\Data\Nico\190306-5\TH533-fr70.D) ES-API, Pos, Scan, Frag: Var, "160-2000scan"
TIC
5.557
5.287
5.964
9.894
10.085
10.658

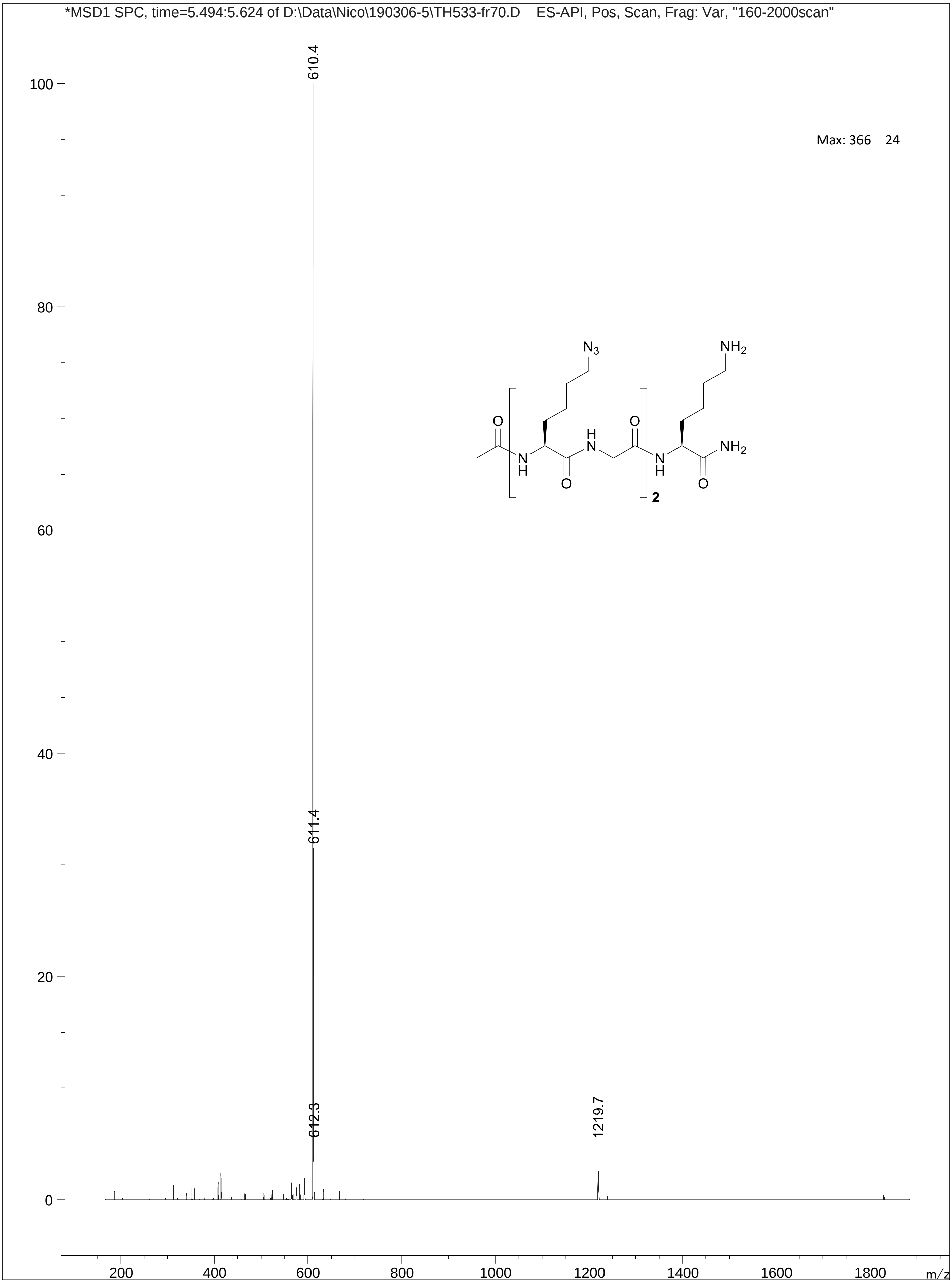
*MSD1 SPC, time=5.494:5.624 of D:\Data\Nico\190306-5\TH533-fr70.D   ES-API, Pos, Scan, Frag: Var, "160-2000scan"
Max: 366   24
610.4
611.4
612.3
1219.7
m/z
N3
NH2
O
H
N
2

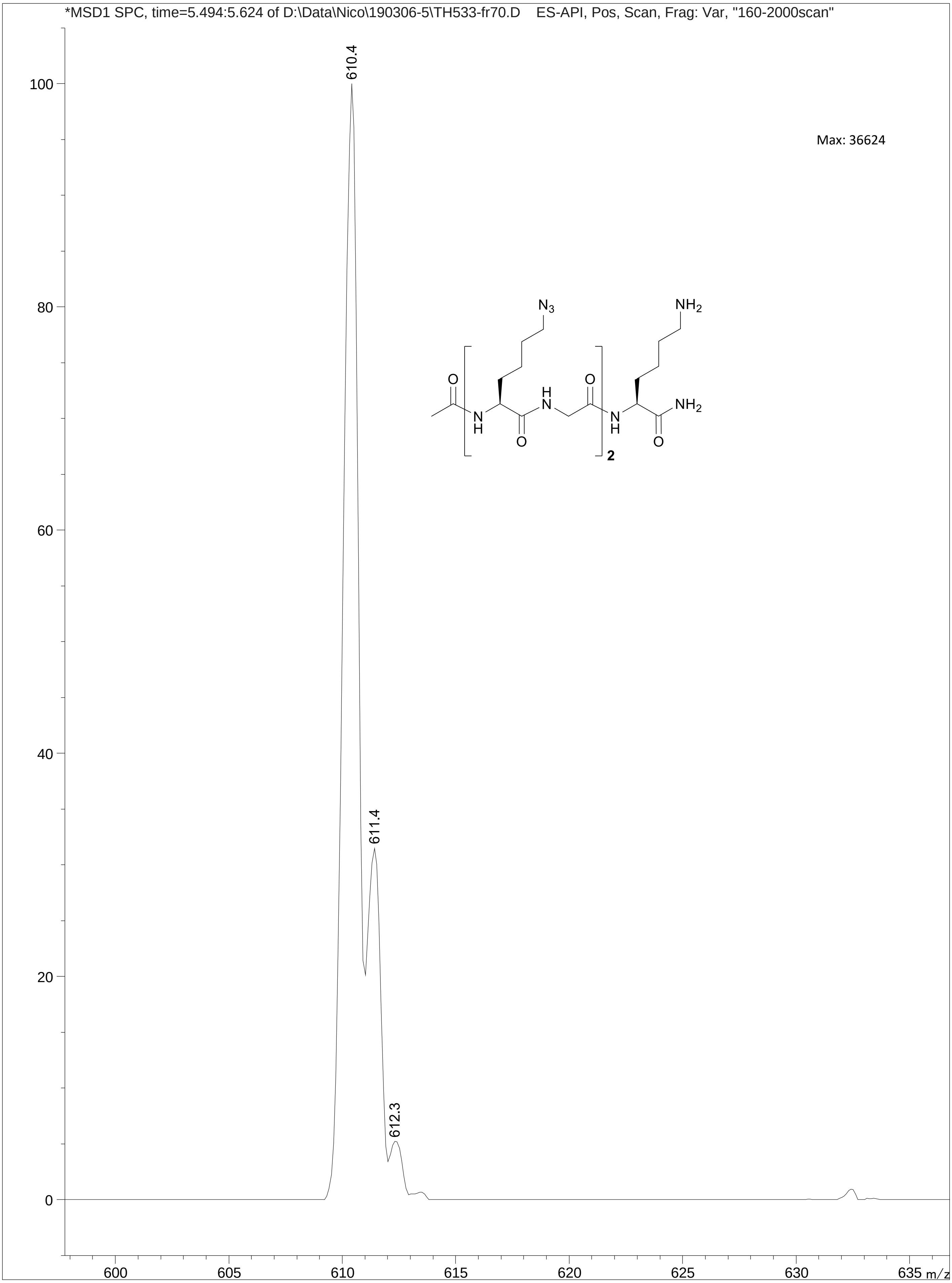
*MSD1 SPC, time=5.494:5.624 of D:\Data\Nico\190306-5\TH533-fr70.D ES-API, Pos, Scan, Frag: Var, "160-2000scan"
Max: 36624
610.4
611.4
612.3
m/z
N3
NH2
2

17

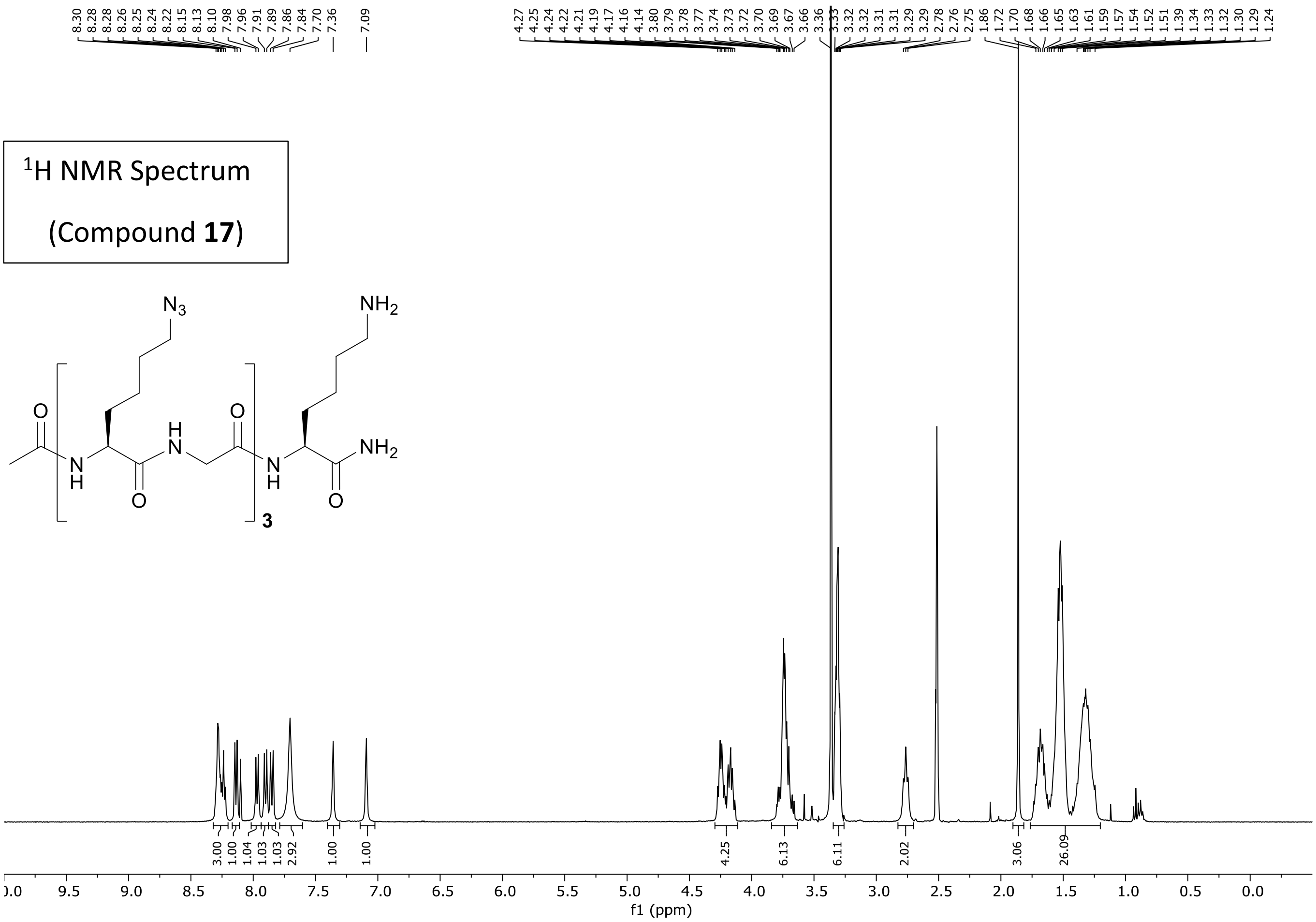

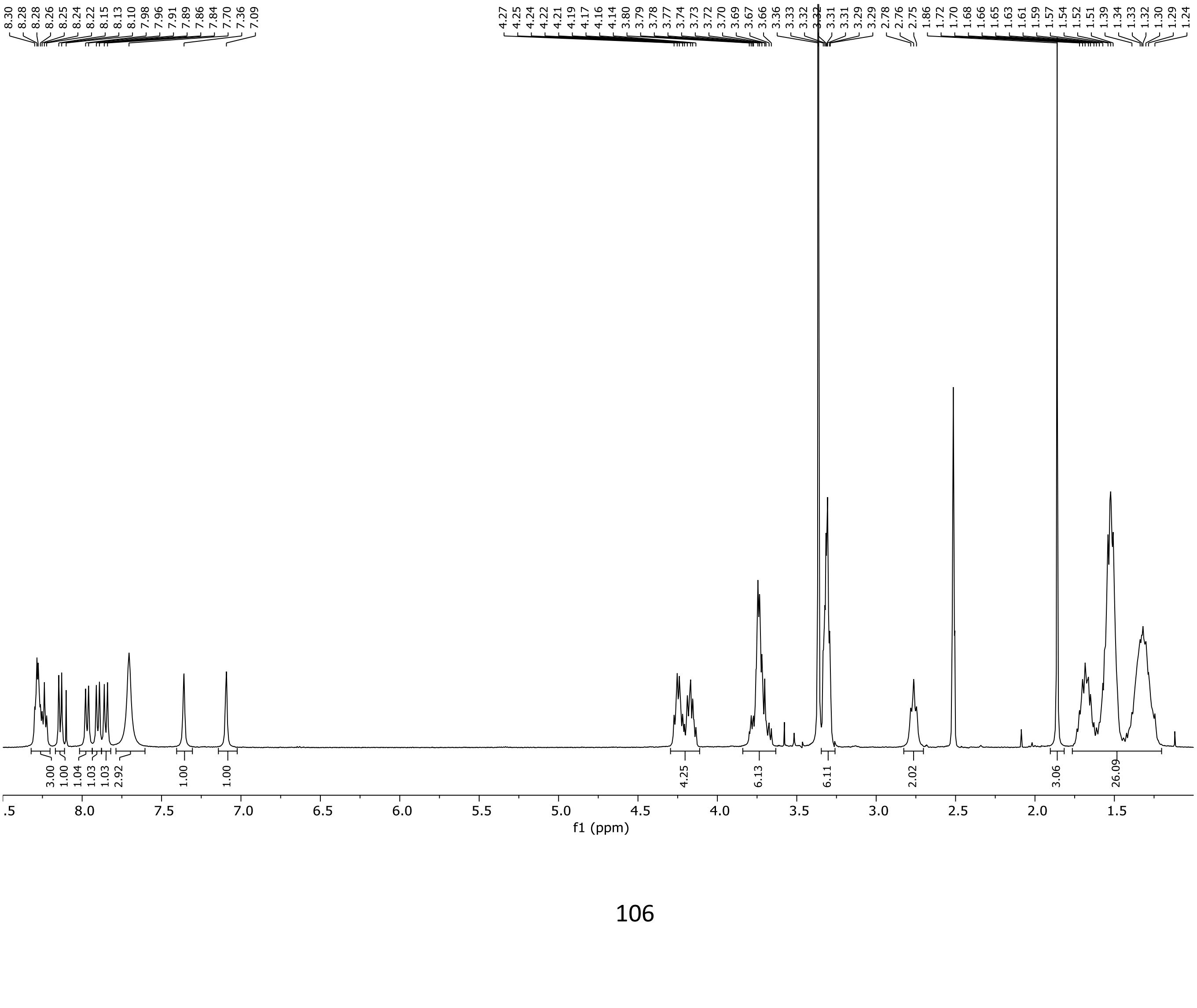

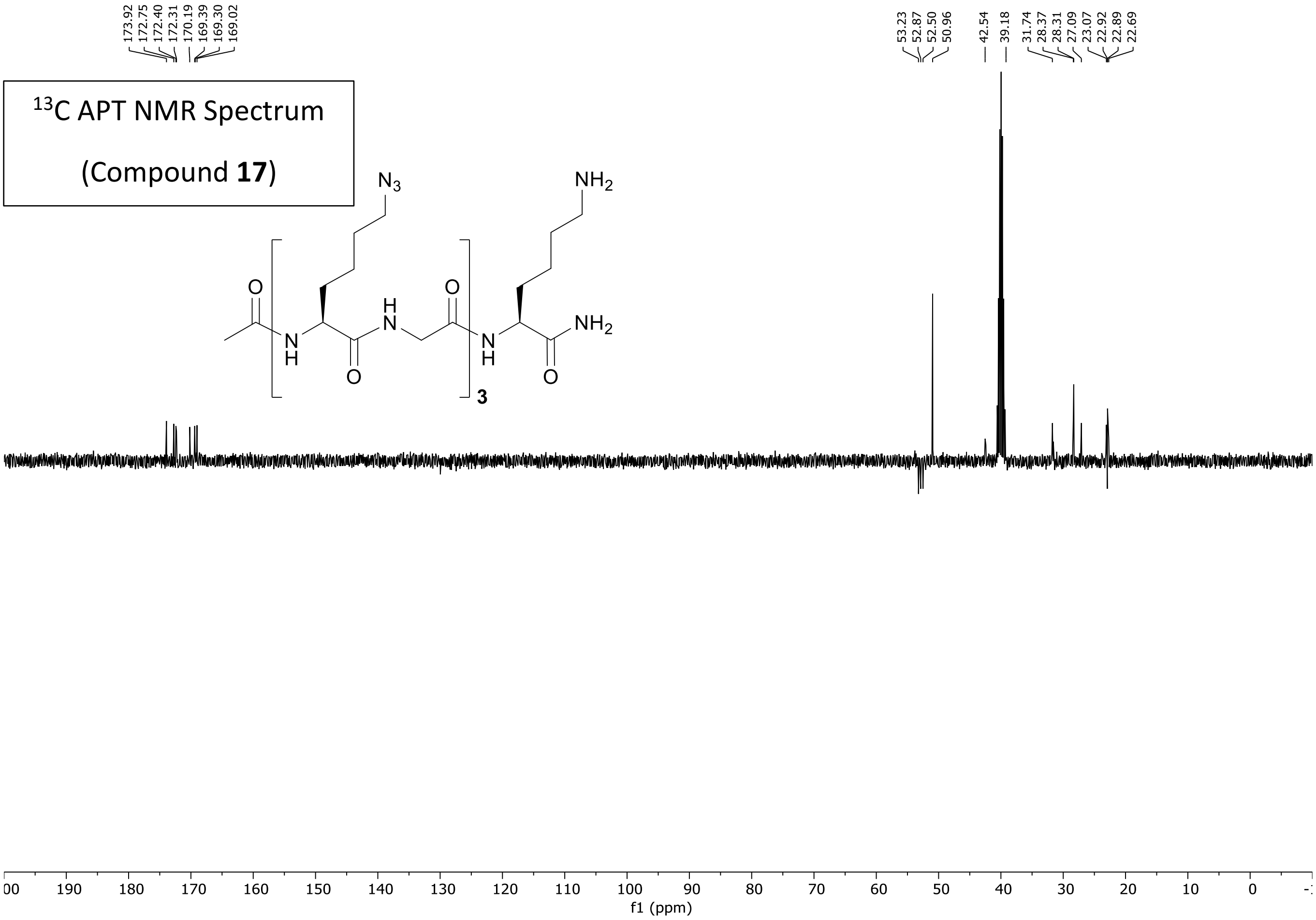
13C APT NMR Spectrum
(Compound 17)
N3
NH2
O
H
N
H
O
N
H
O
N
H
NH2
O
3
173.92
172.75
172.40
172.31
170.19
169.39
169.30
169.02
53.23
52.87
52.50
50.96
42.54
39.18
31.74
28.37
28.31
27.09
23.07
22.92
22.89
22.69
190
180
170
160
150
140
130
120
110
100
90
80
70
60
50
40
30
20
10
0
f1 (ppm)

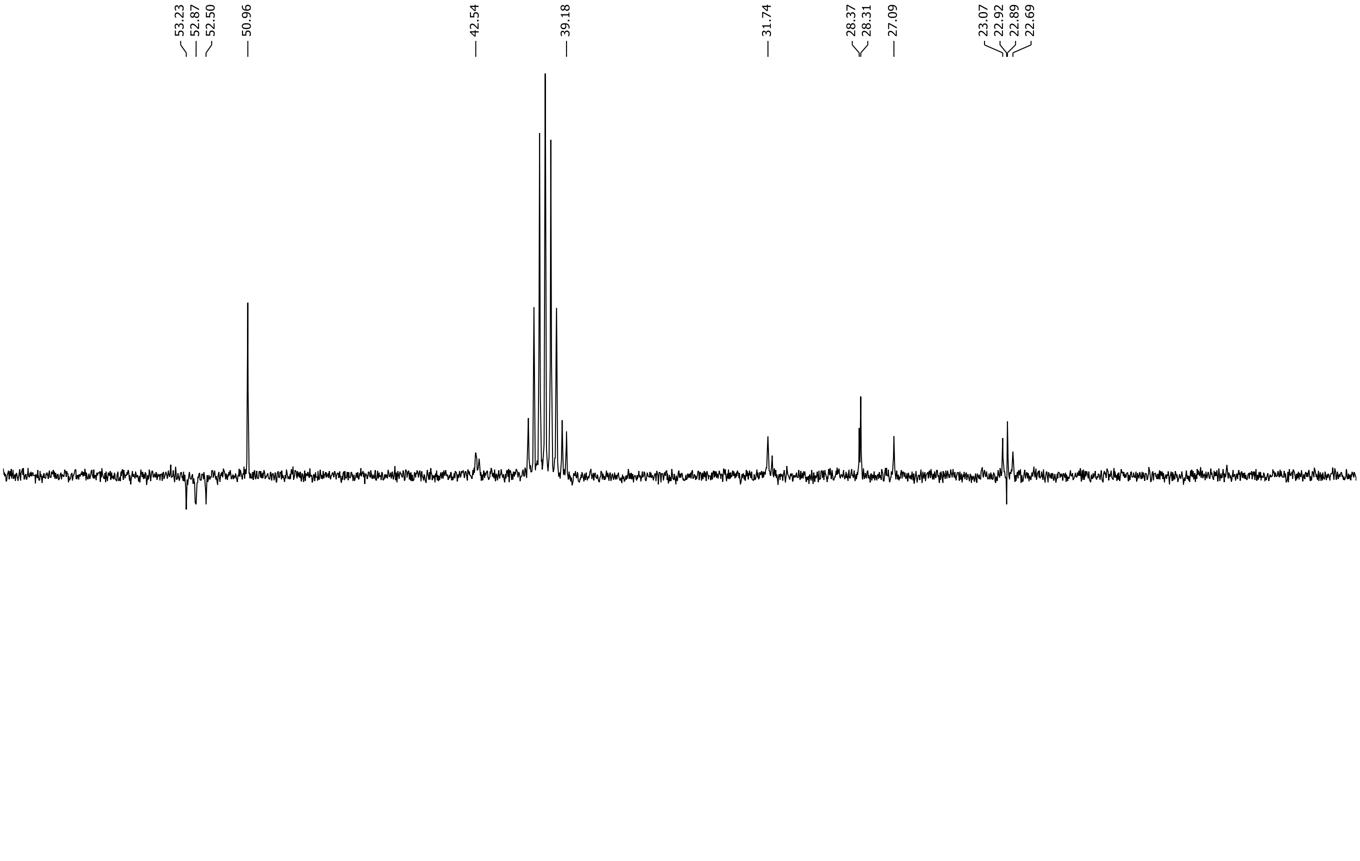
53.23
52.87
52.50
50.96
42.54
39.18
31.74
28.37
28.31
27.09
23.07
22.92
22.89
22.69
55
50
45
40
35
30
25
20
15
f1 (ppm)

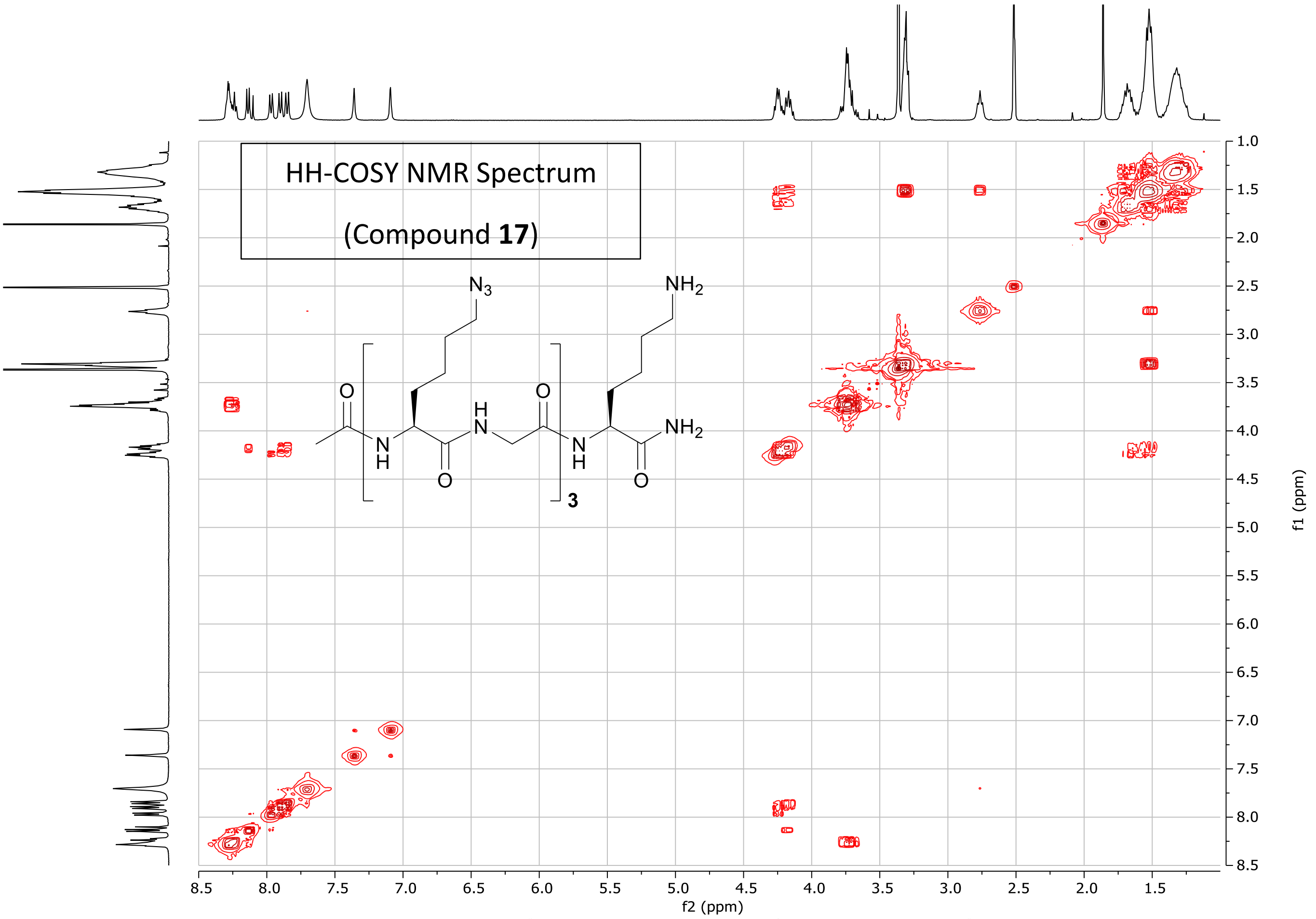
HH-COSY NMR Spectrum
(Compound 17)
N3
NH2
O
N
H
3
f2 (ppm)
f1 (ppm)

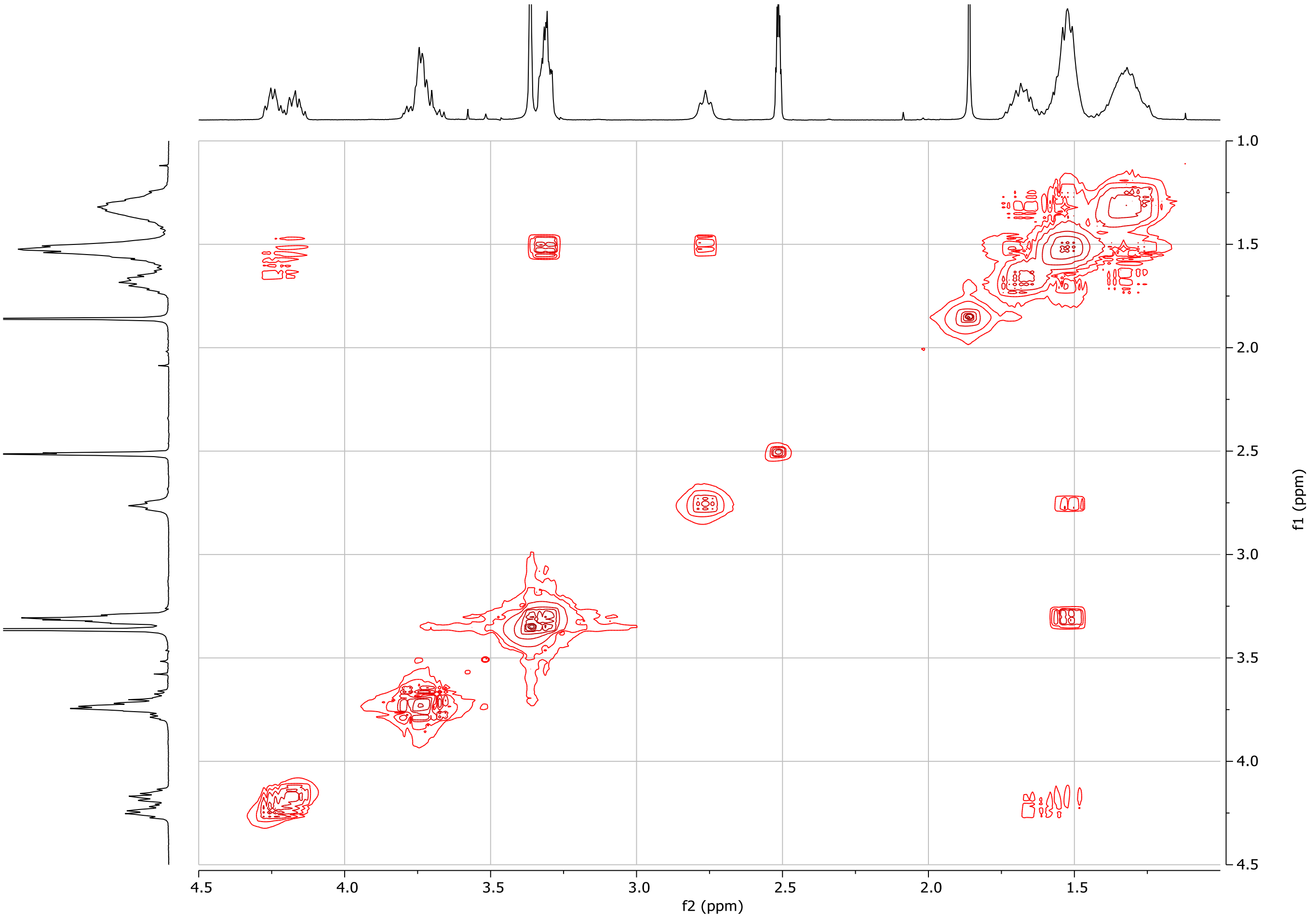
f2 (ppm)
f1 (ppm)

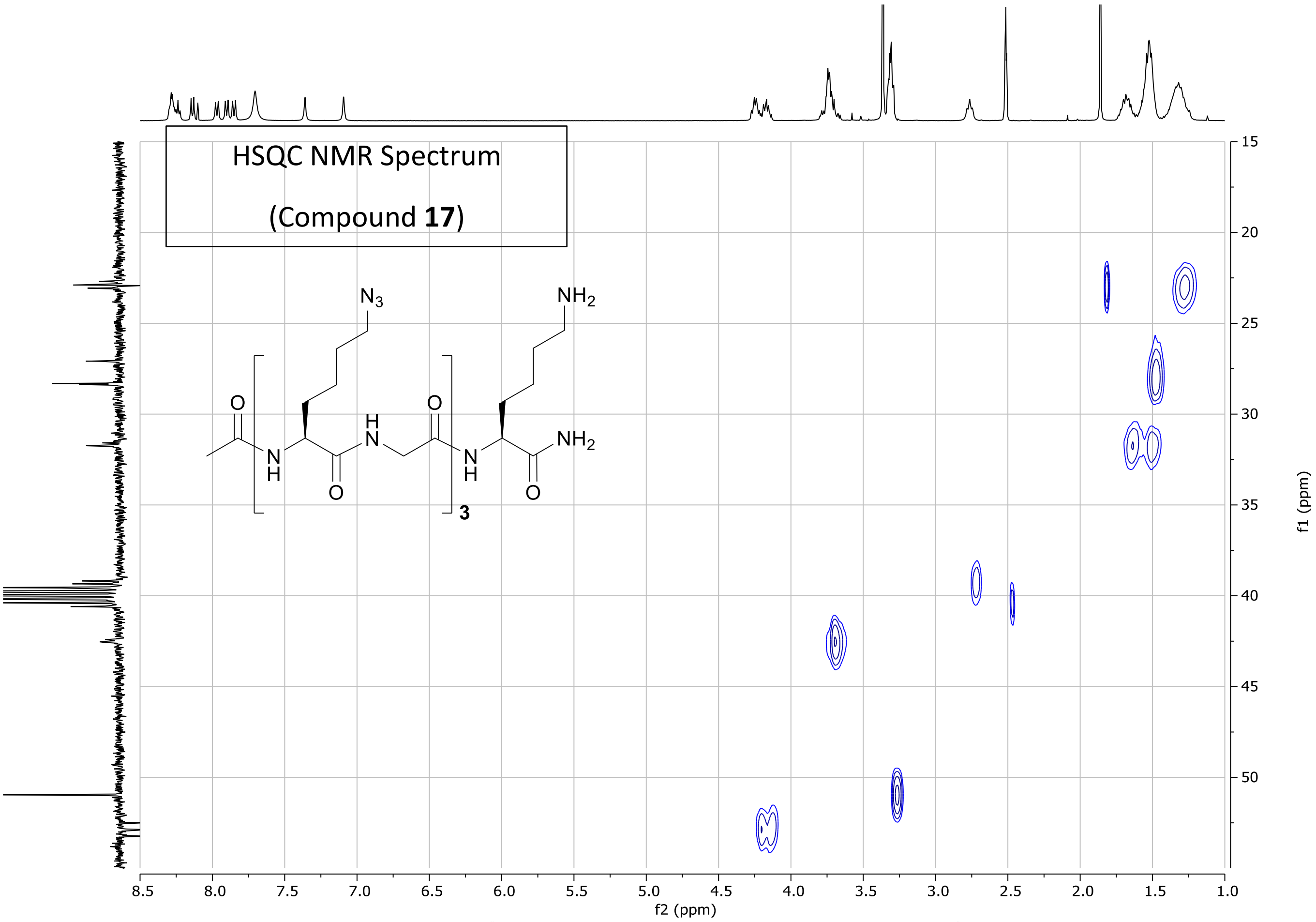
HSQC NMR Spectrum
(Compound **17**)
N3
NH2
O
H
N
O
N
H
O
N
H
O
NH2
O
3
f2 (ppm)
f1 (ppm)

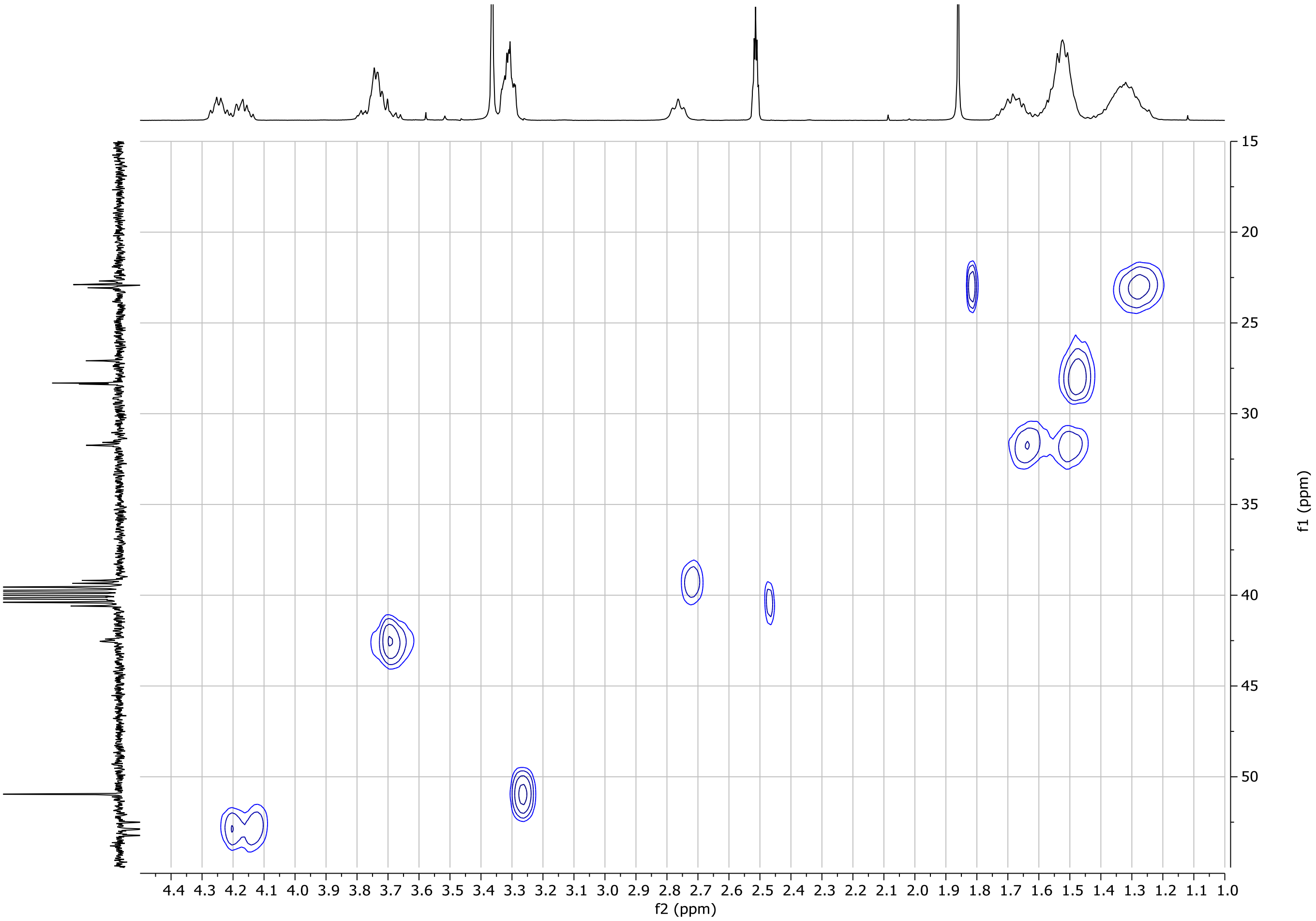
f2 (ppm)
f1 (ppm)

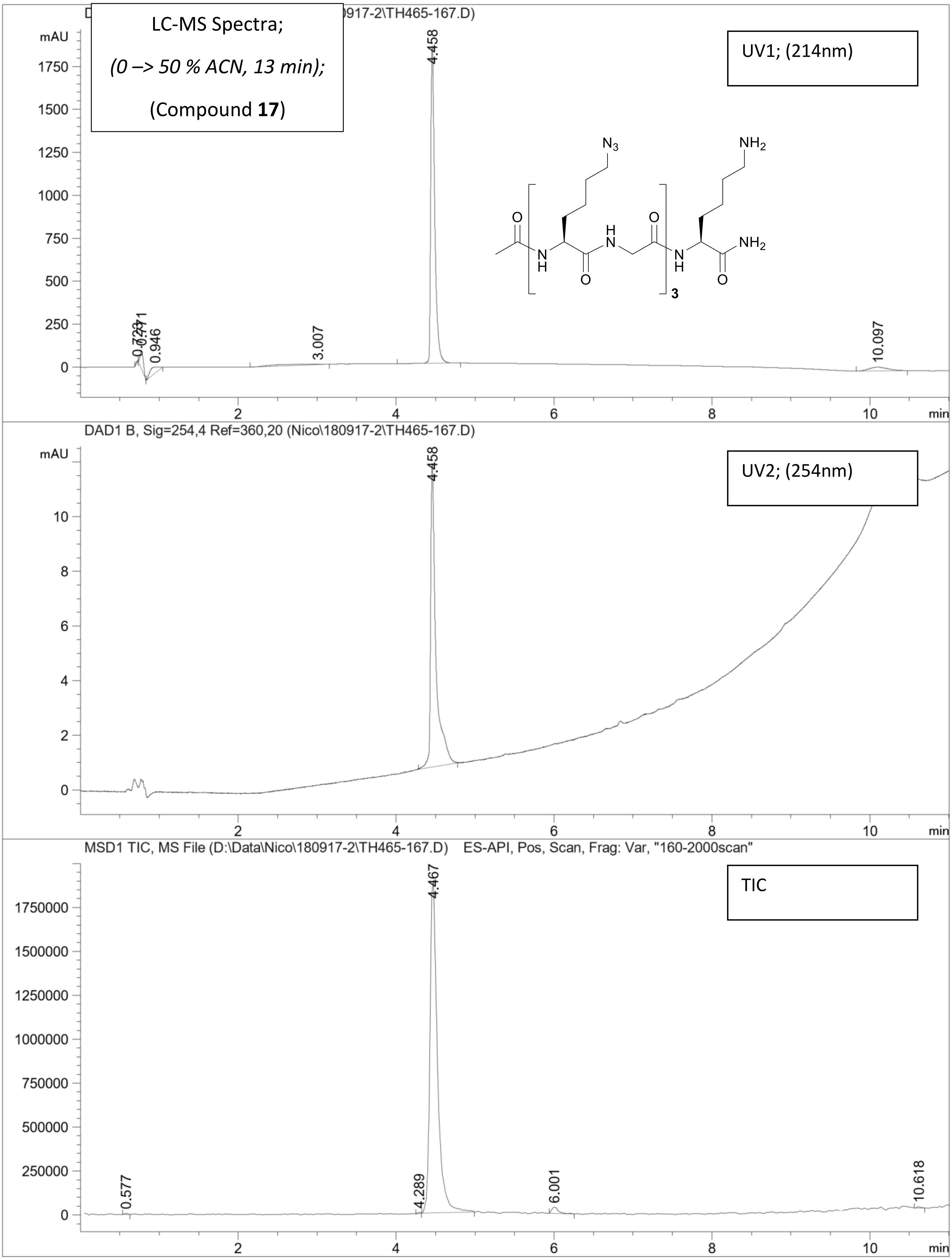
LC-MS Spectra;
(0 –> 50 % ACN, 13 min);
(Compound 17)
UV1; (214nm)
UV2; (254nm)
TIC
DAD1 B, Sig=254,4 Ref=360,20 (Nico\180917-2\TH465-167.D)
MSD1 TIC, MS File (D:\Data\Nico\180917-2\TH465-167.D) ES-API, Pos, Scan, Frag: Var, "160-2000scan"
mAU
min
4.458
3.007
10.097
0.946
4.467
4.289
0.577
6.001
10.618
N3
NH2
O
N
H
3

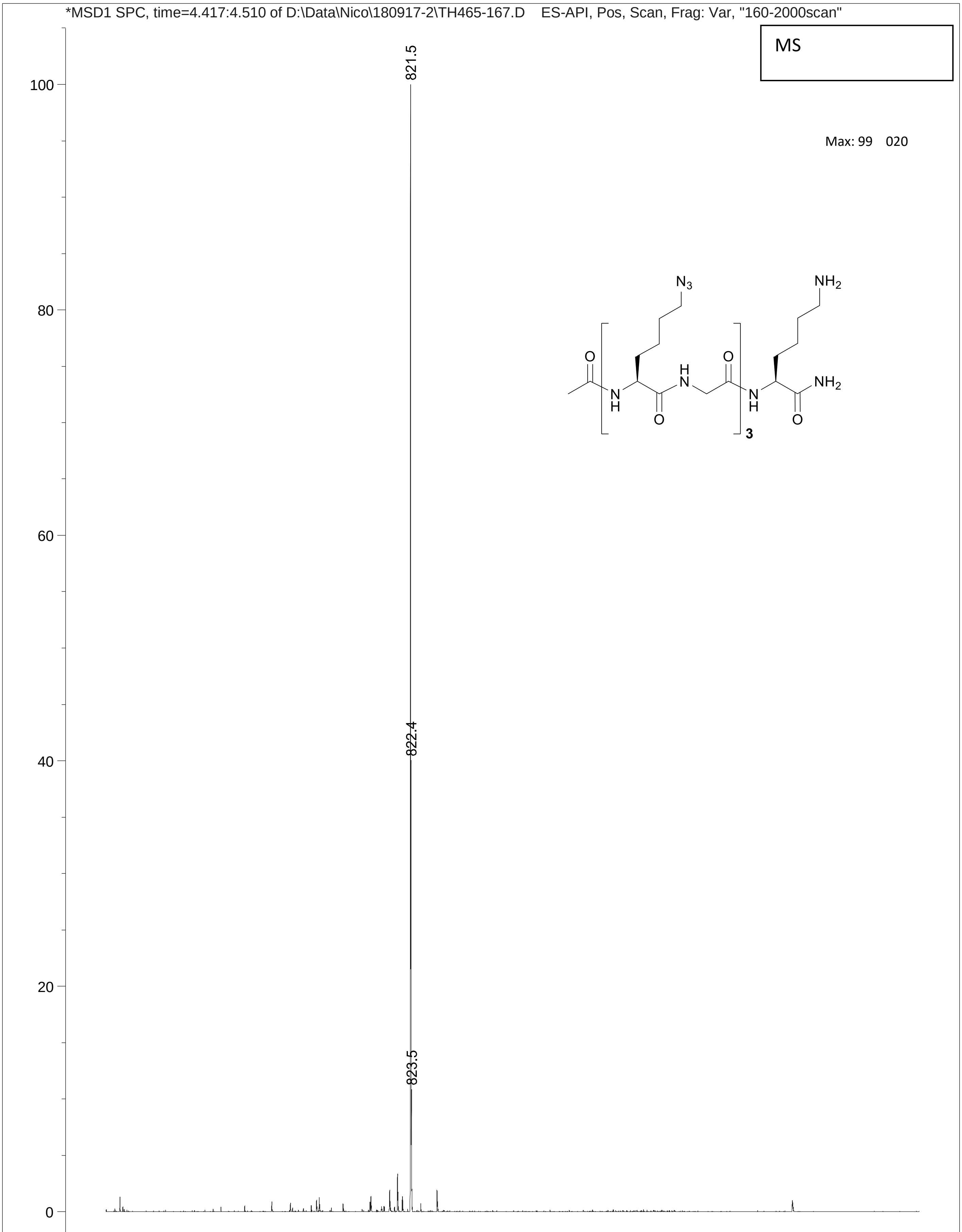
*MSD1 SPC, time=4.417:4.510 of D:\Data\Nico\180917-2\TH465-167.D ES-API, Pos, Scan, Frag: Var, "160-2000scan"
MS
Max: 99 020
821.5
822.4
823.5
m/z
N3
NH2
3

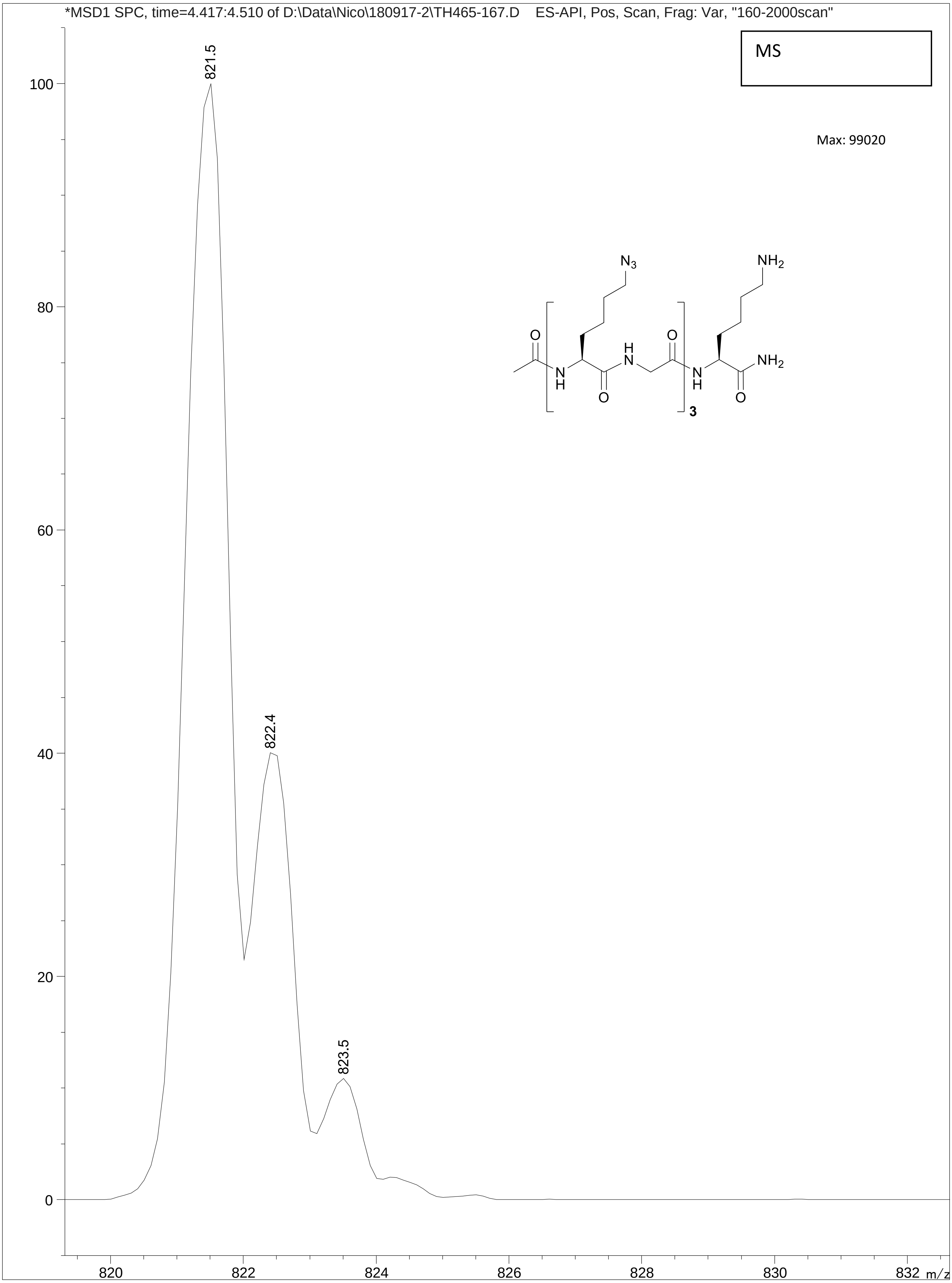
*MSD1 SPC, time=4.417:4.510 of D:\Data\Nico\180917-2\TH465-167.D ES-API, Pos, Scan, Frag: Var, "160-2000scan"
MS
Max: 99020
821.5
822.4
823.5
100
80
60
40
20
0
820
822
824
826
828
830
832
m/z
N3
NH2
O
H
N
NH2
3

18

^{1}H NMR Spectrum

(Compound **18**)

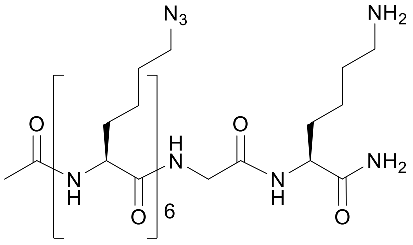

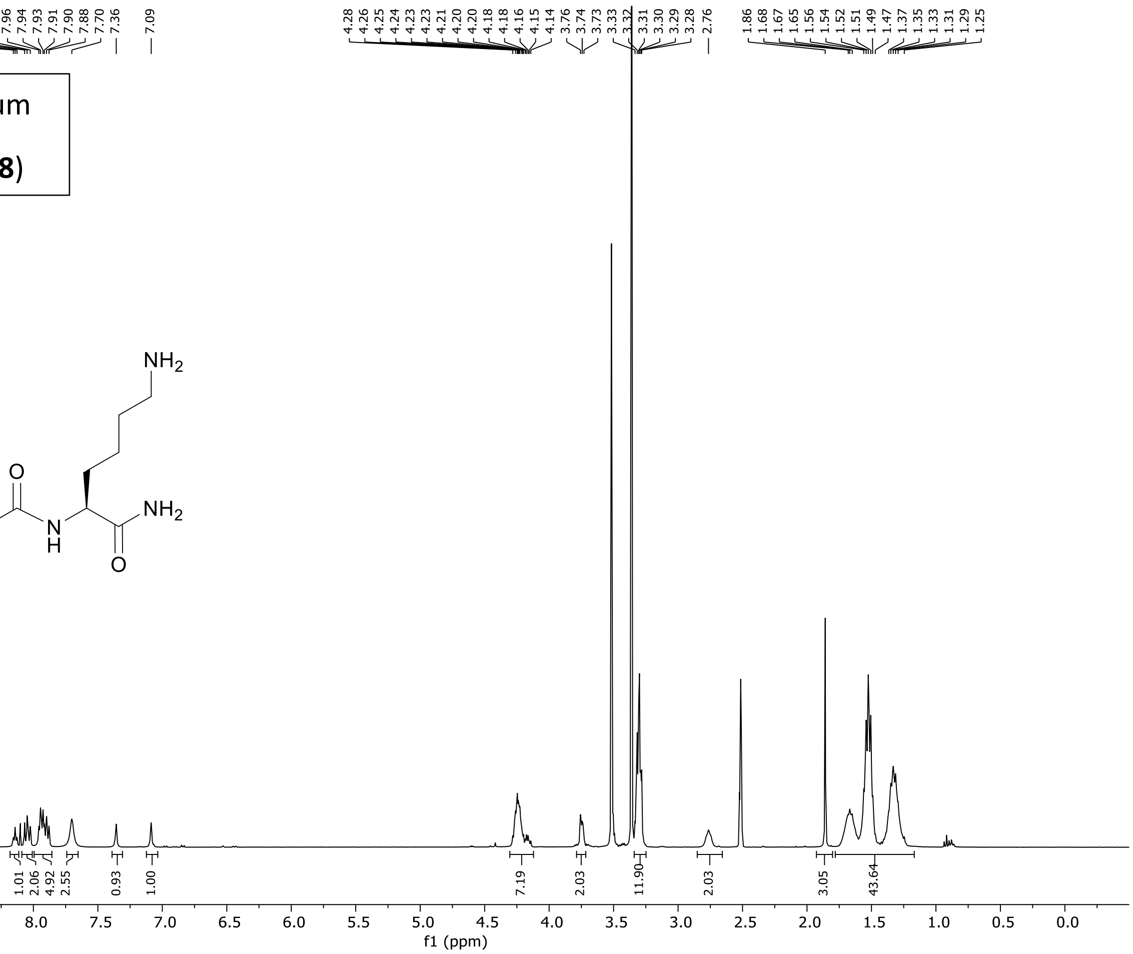

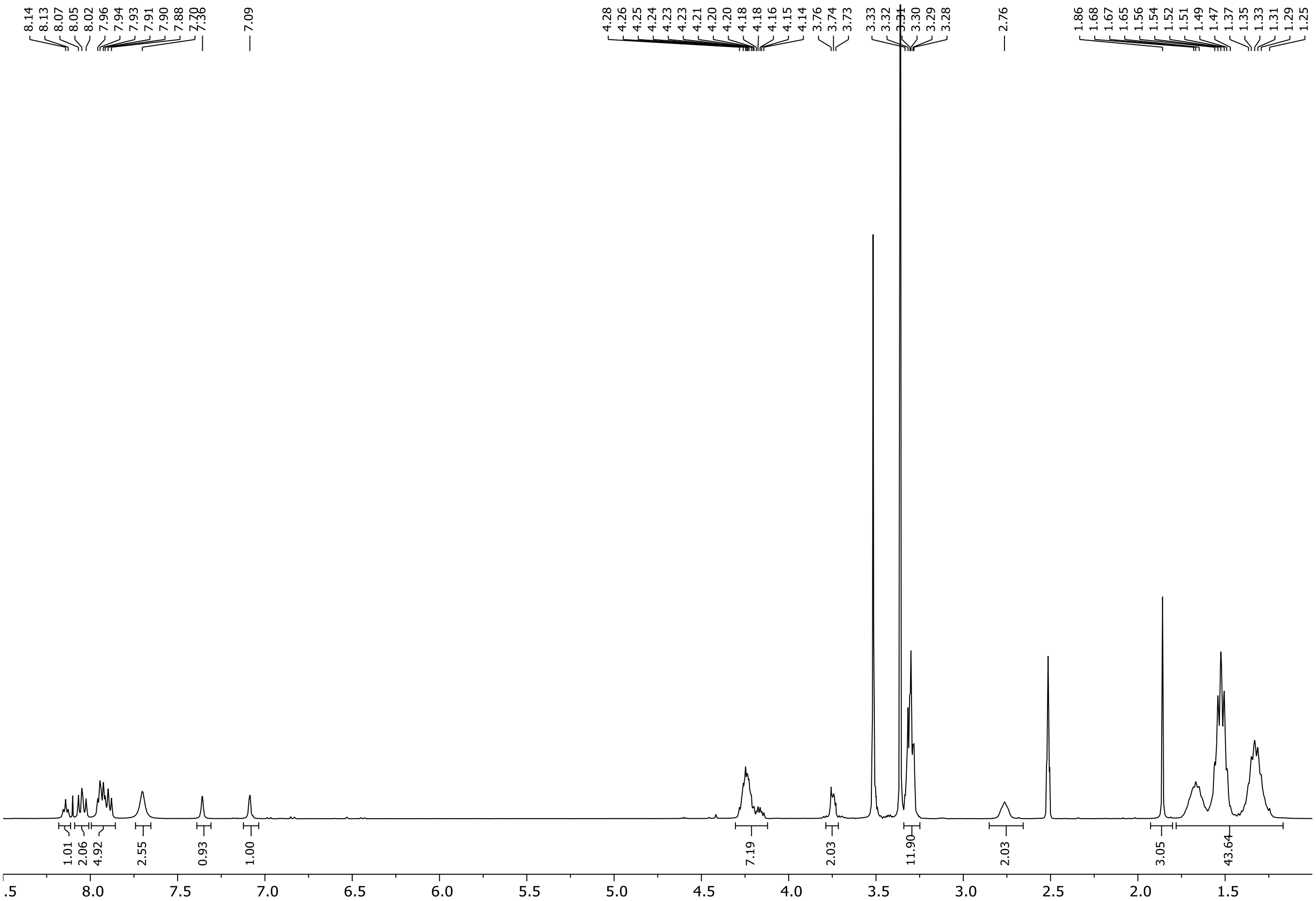

^{13}C APT NMR Spectrum

(Compound **18**)

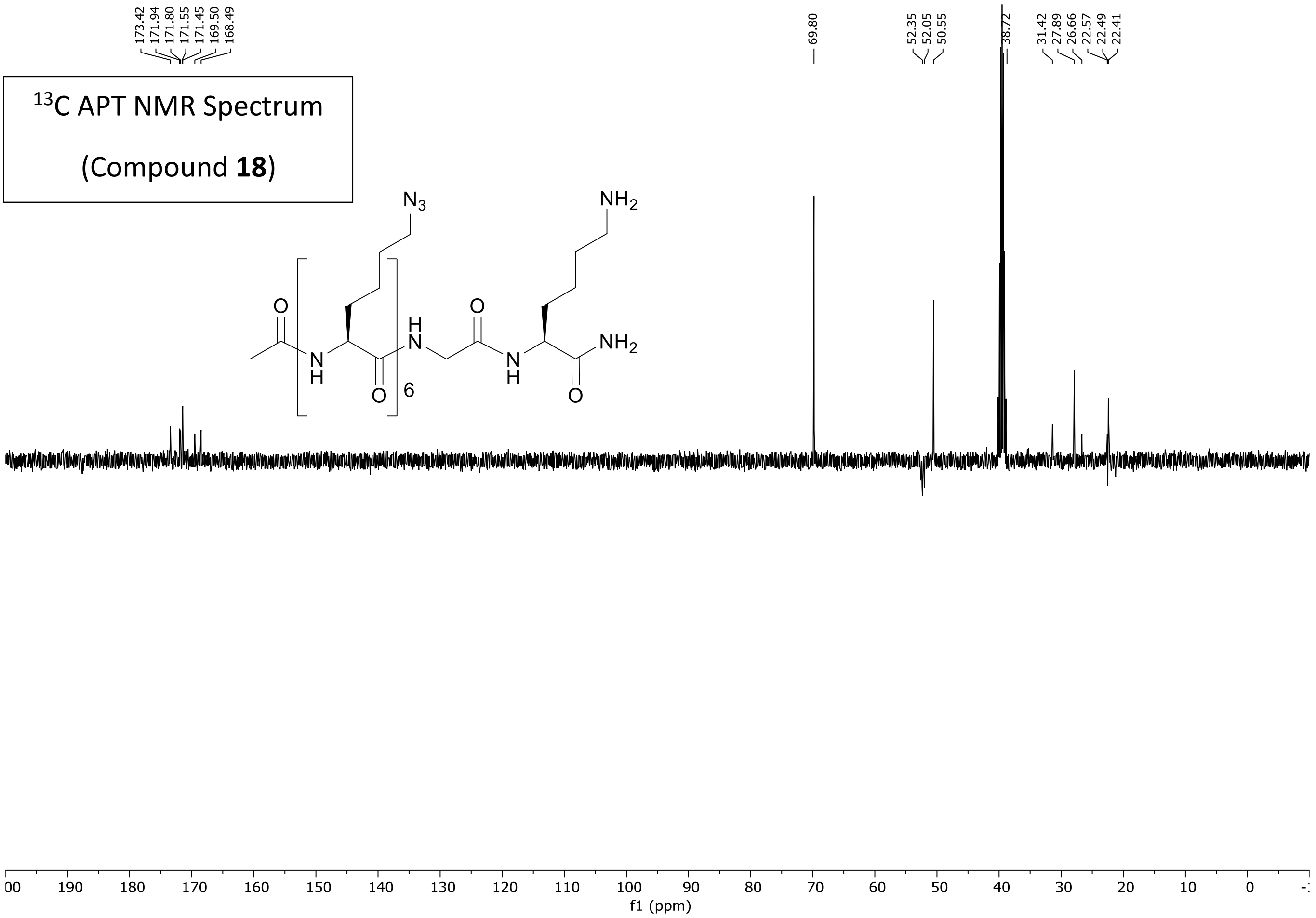

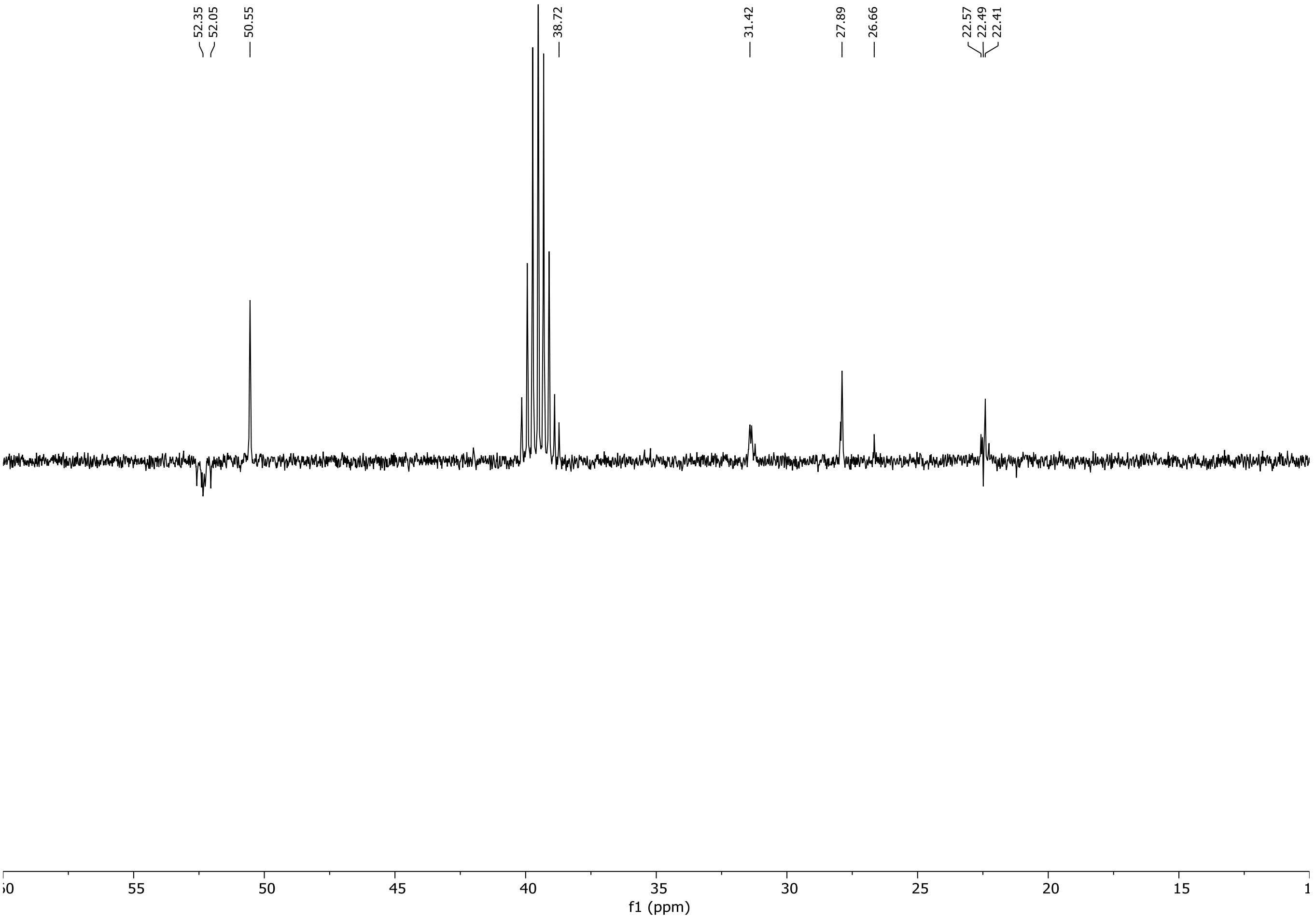

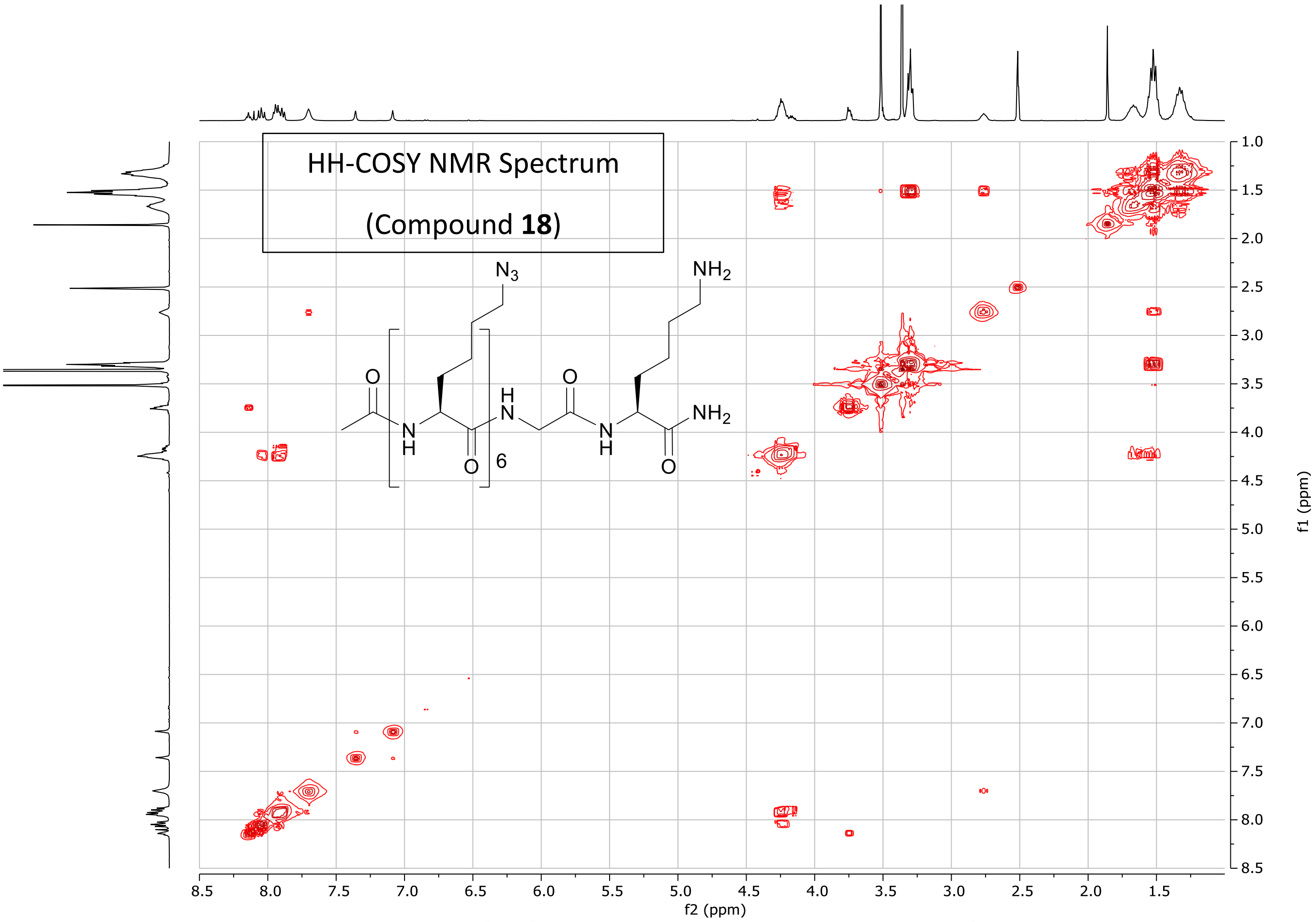
HH-COSY NMR Spectrum
(Compound 18)
N3
NH2
NH2
6
f2 (ppm)
f1 (ppm)

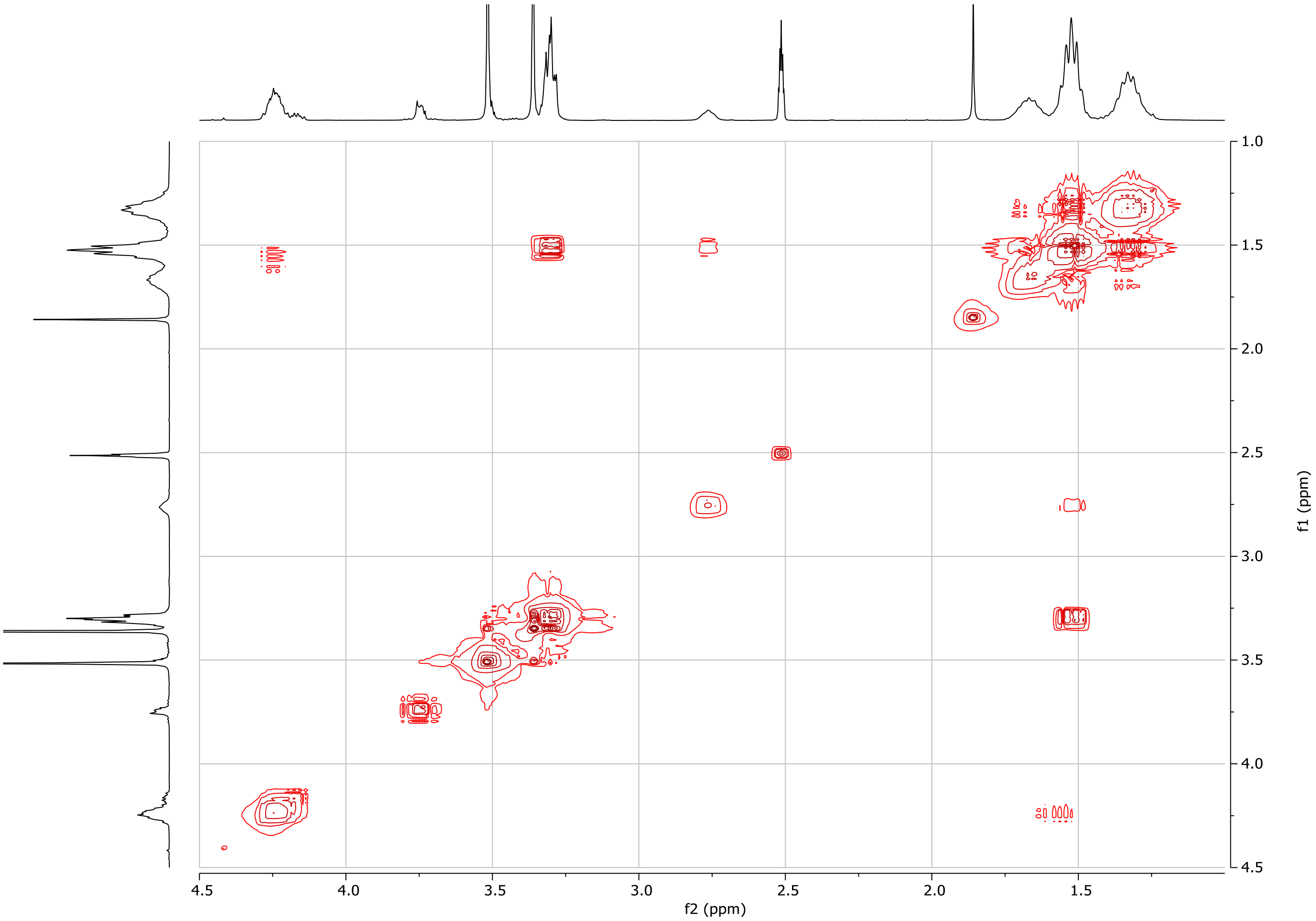
f2 (ppm)
f1 (ppm)

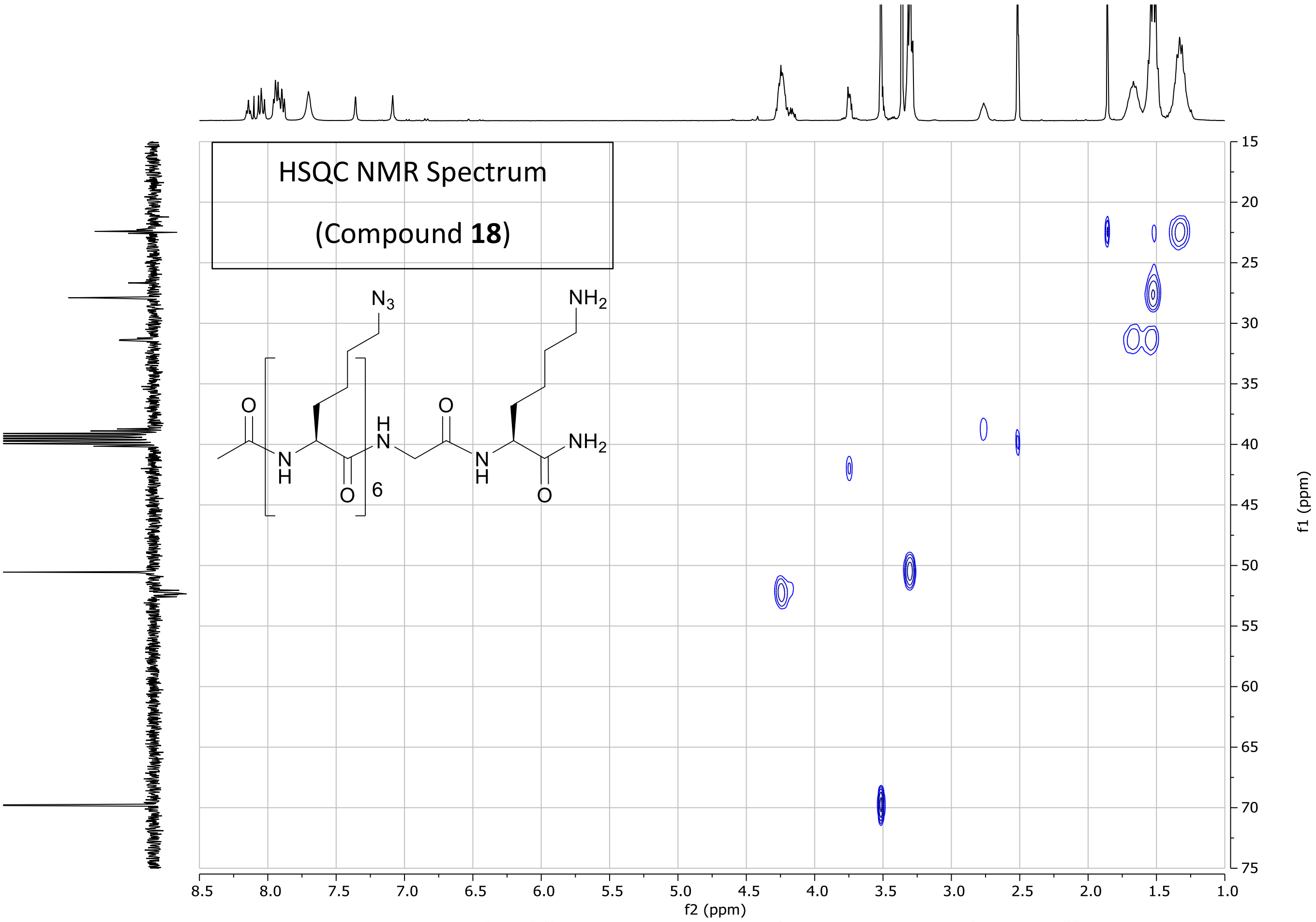

HSQC NMR Spectrum
(Compound 18)
f1 (ppm)
f2 (ppm)

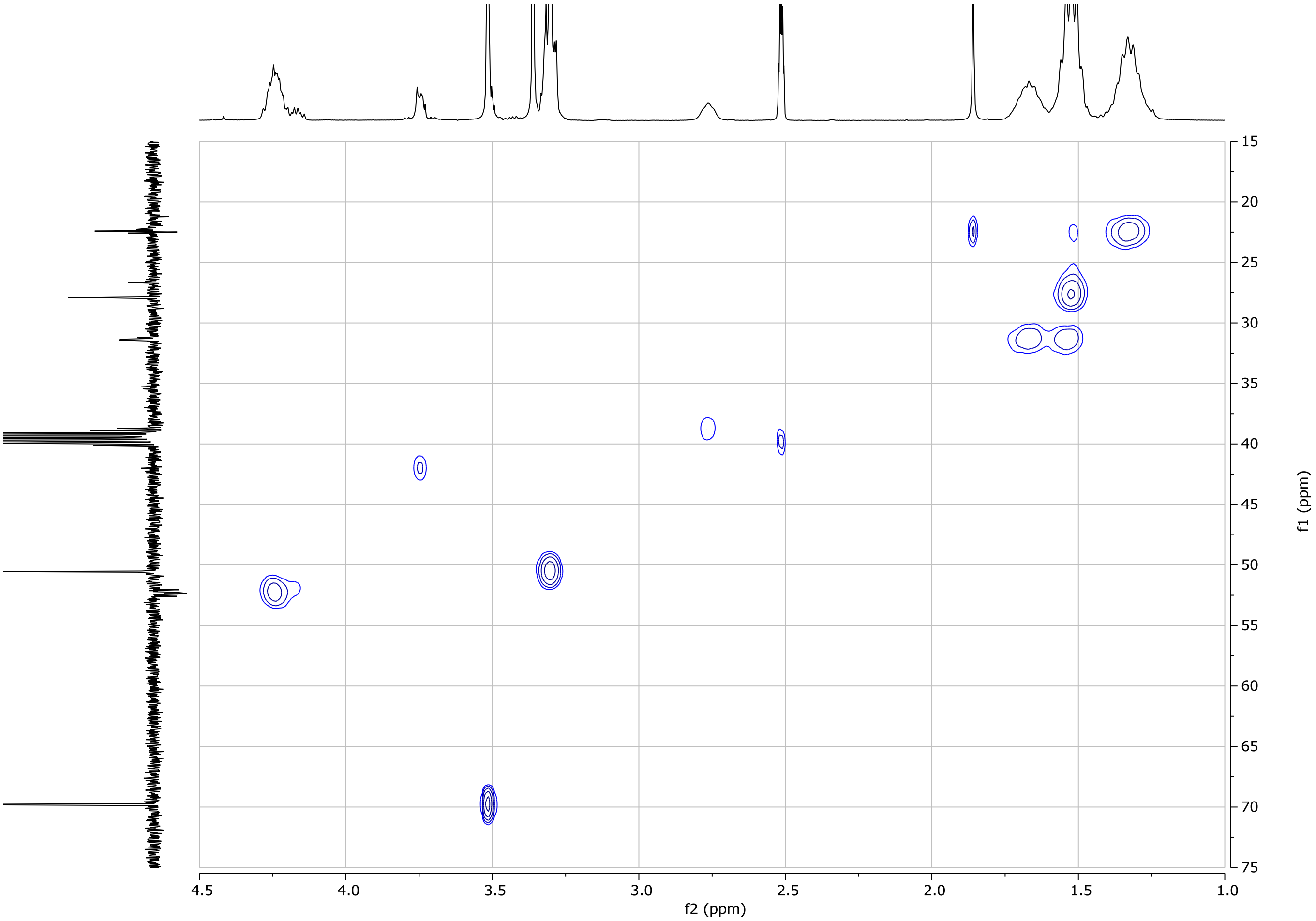

f1 (ppm)
f2 (ppm)

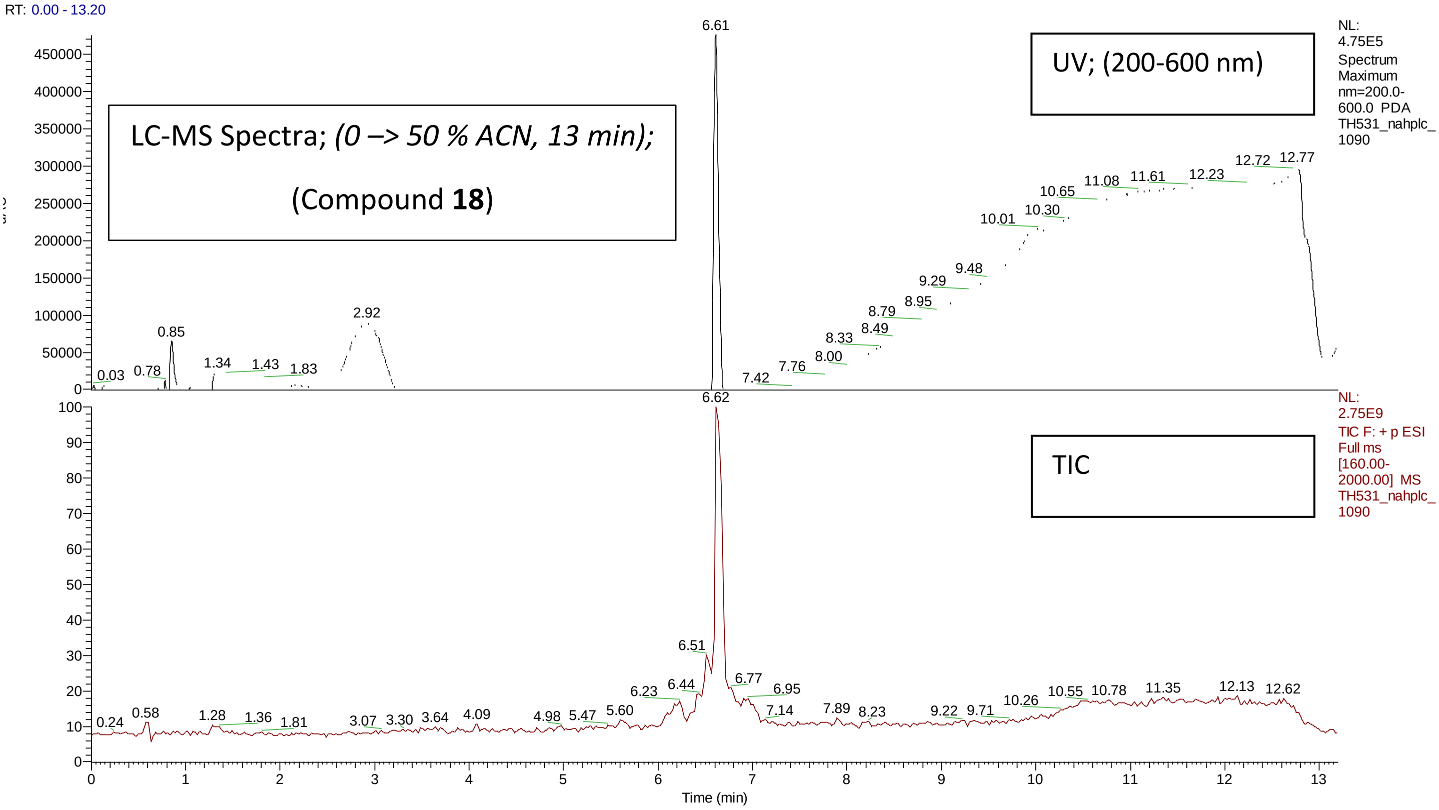
RT: 0.00 - 13.20
LC-MS Spectra; (0 –> 50 % ACN, 13 min);
(Compound 18)
UV; (200-600 nm)
NL: 4.75E5 Spectrum Maximum nm=200.0-600.0 PDA TH531_nahplc_1090
6.61
TIC
NL: 2.75E9 TIC F: + p ESI Full ms [160.00-2000.00] MS TH531_nahplc_1090
6.62
Time (min)

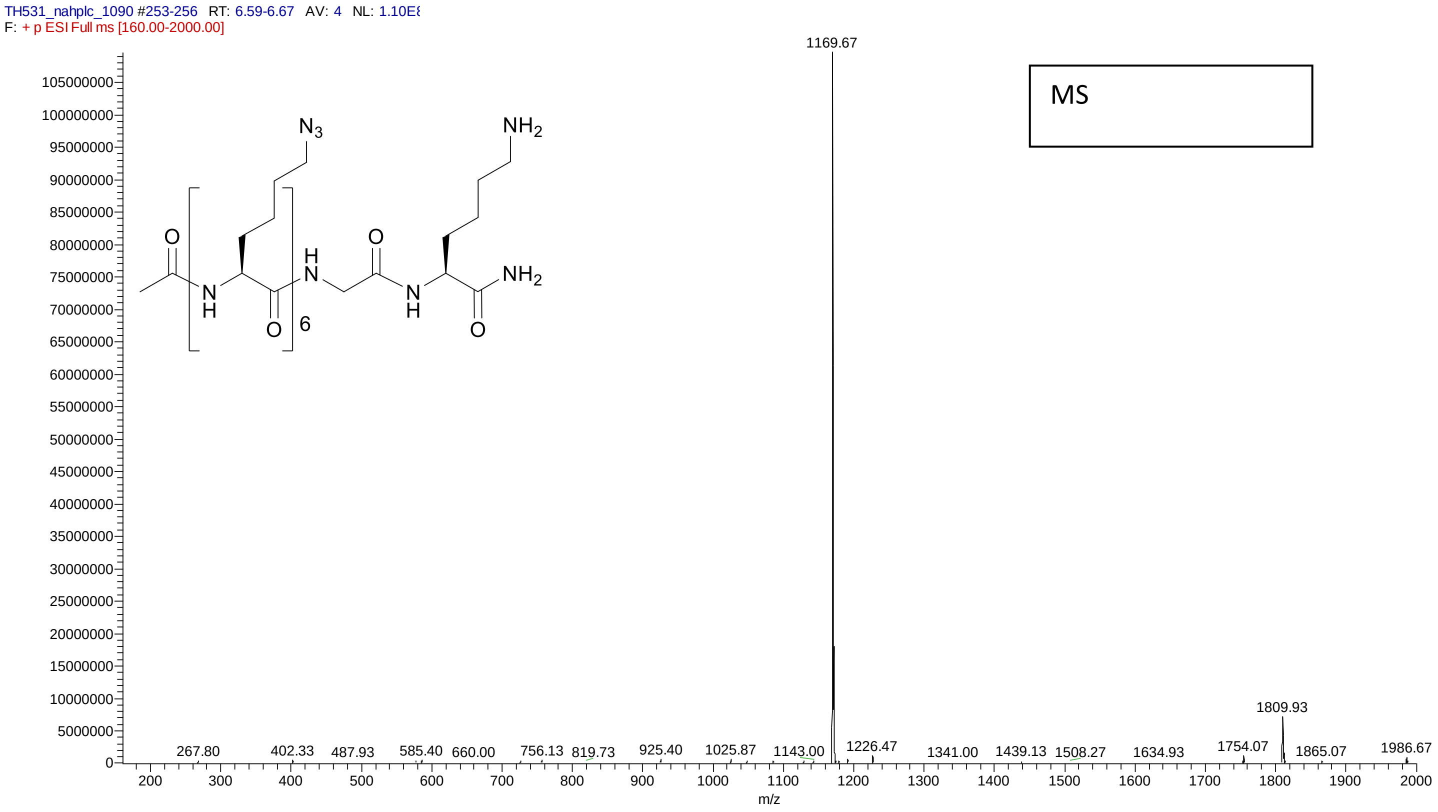
TH531_nahplc_1090 #253-256 RT: 6.59-6.67 AV: 4 NL: 1.10E8
F: + p ESI Full ms [160.00-2000.00]
MS
1169.67
1809.93
m/z

Spectra of CLR ligands, free amine:

A1

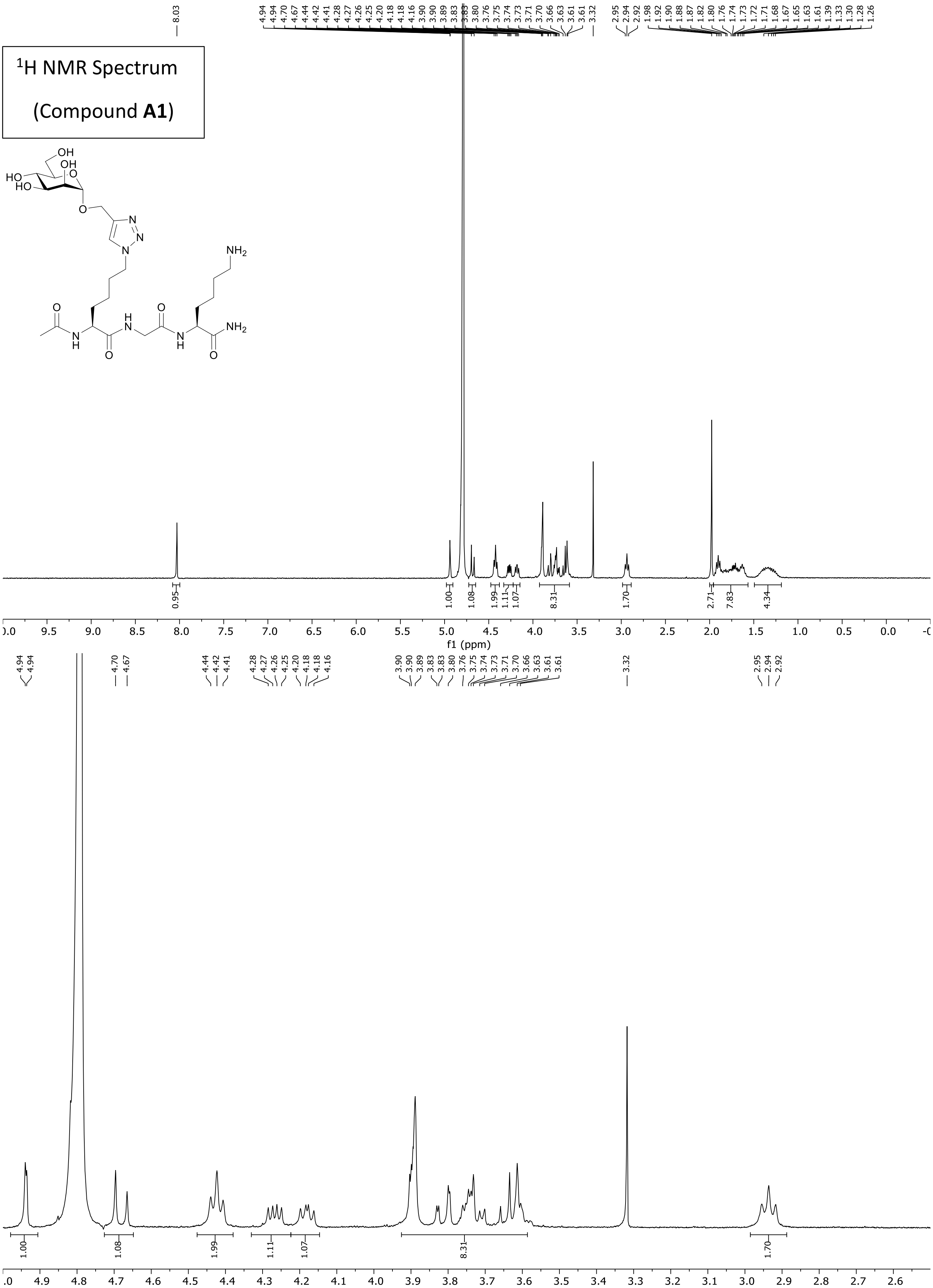

LC-MS Spectra; *(0 –> 50 % ACN, 13 min)* (Compound **A1**)

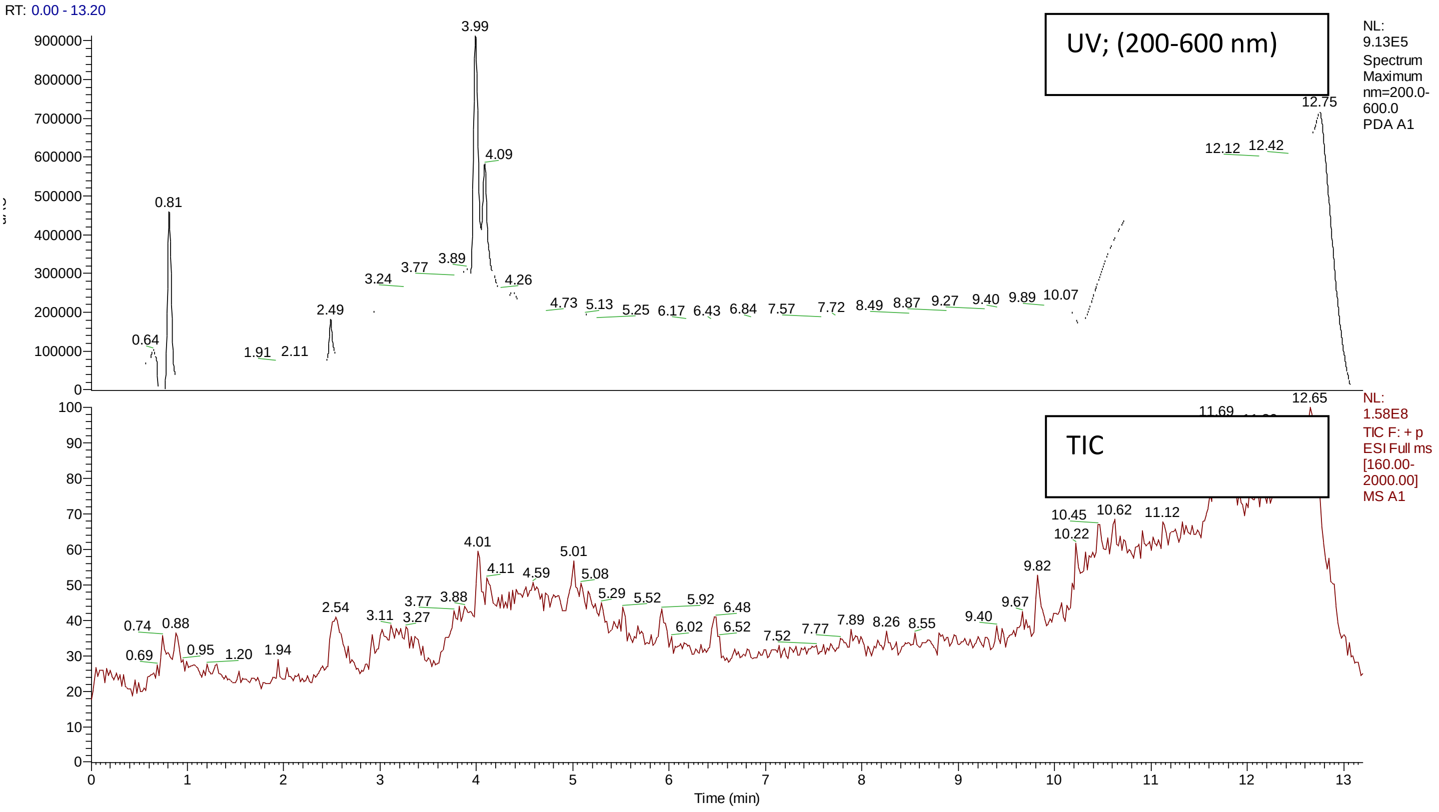

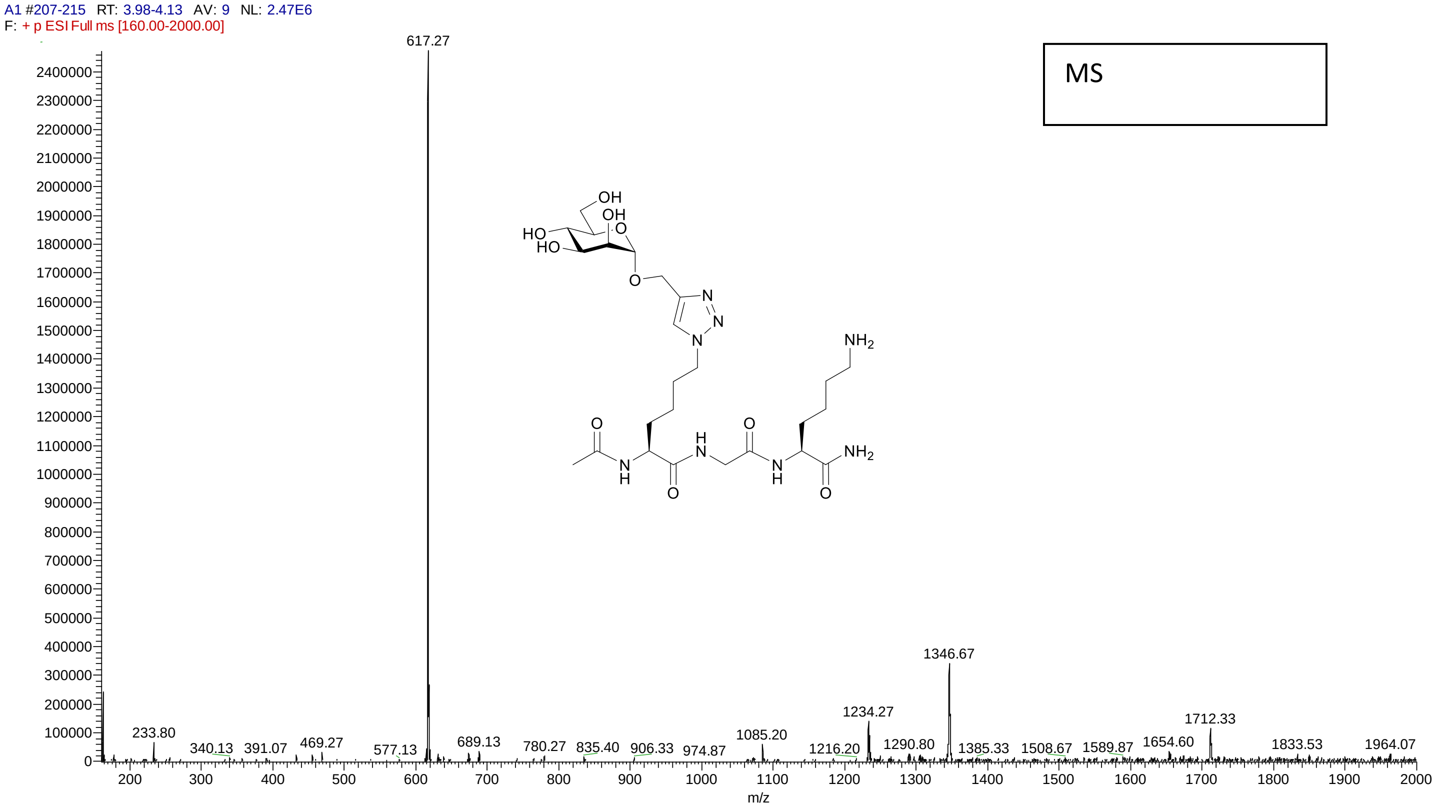

A2

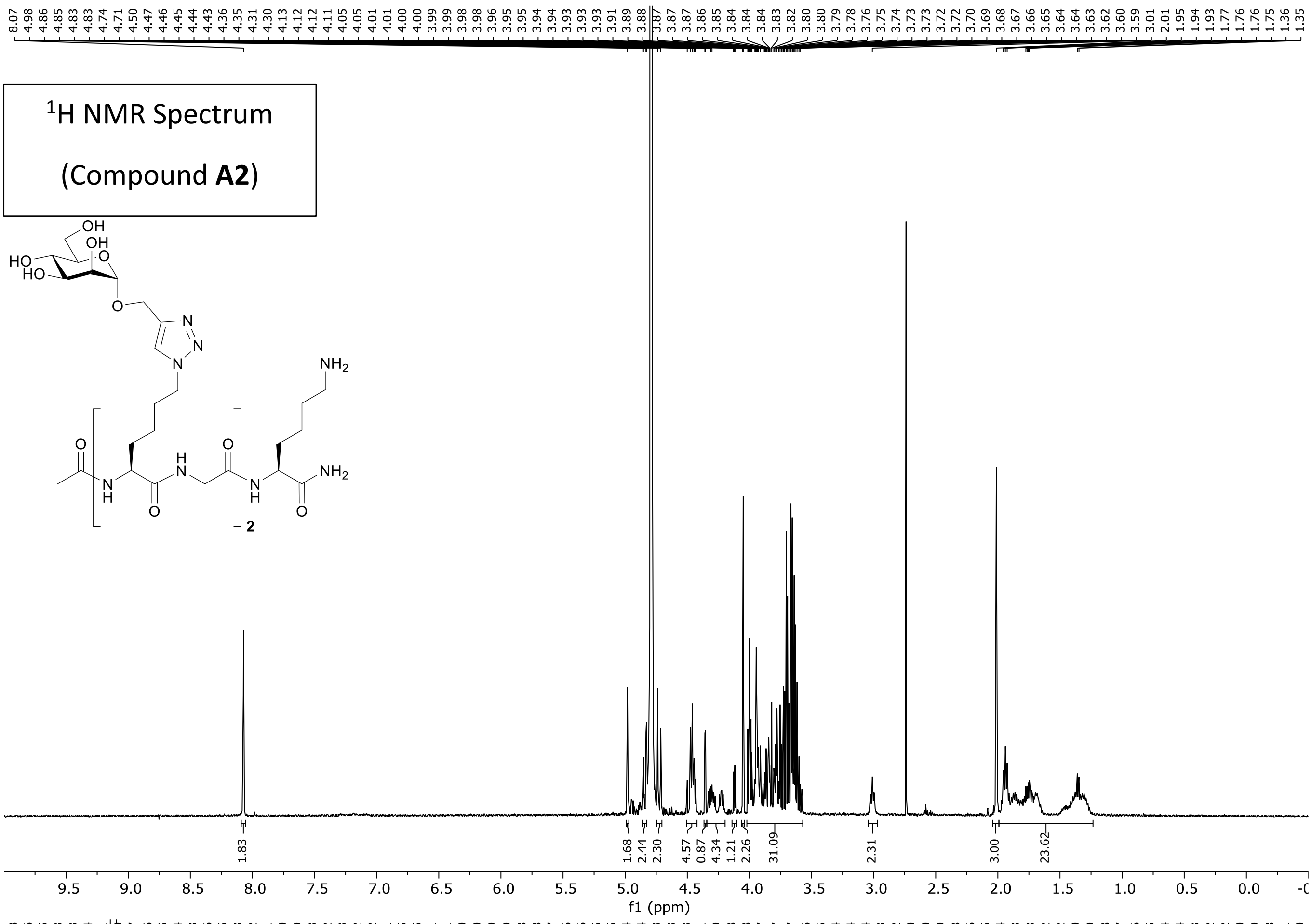

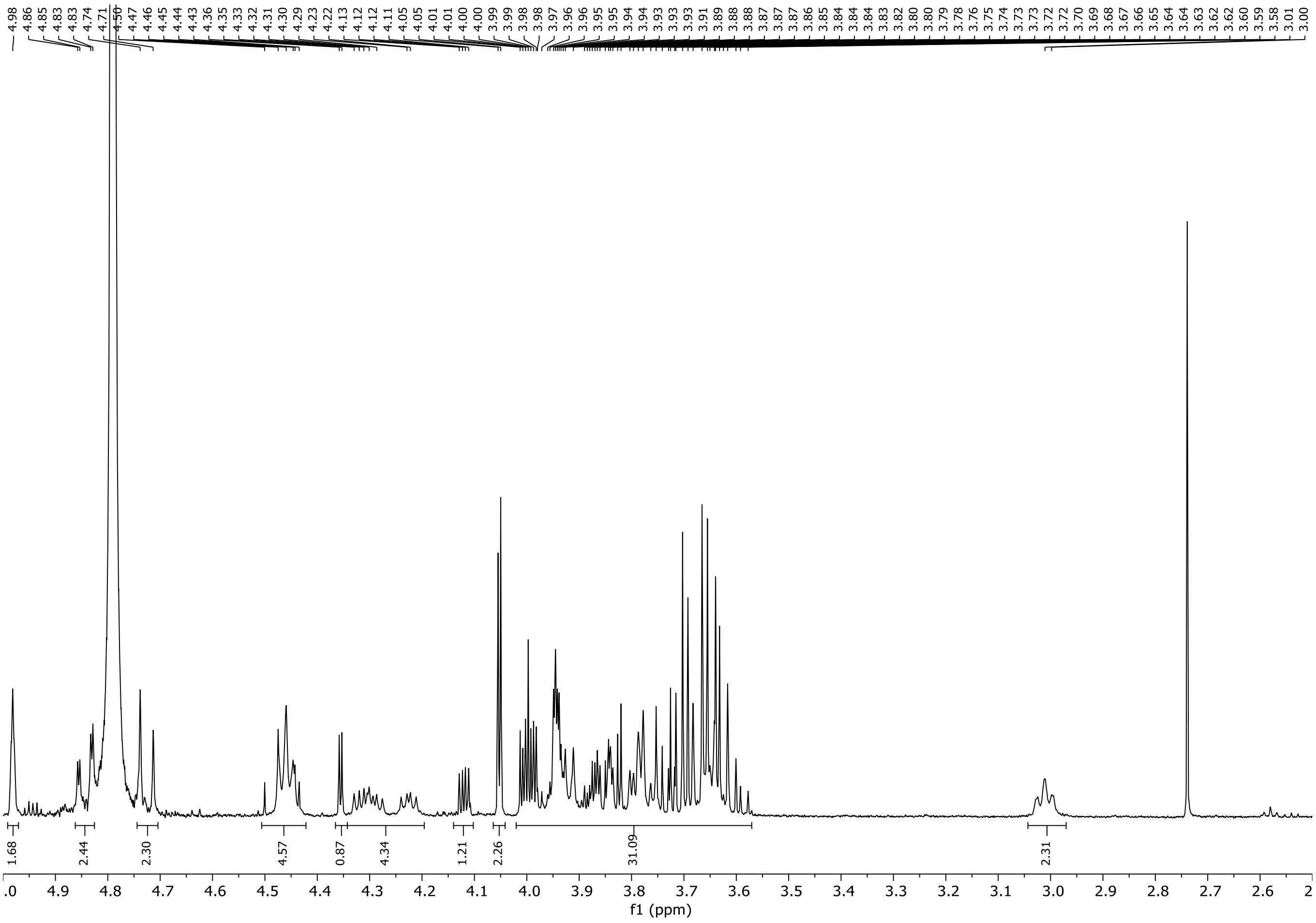

LC-MS Spectra; *(0 –> 50 % ACN, 13 min);* (Compound **A2**)

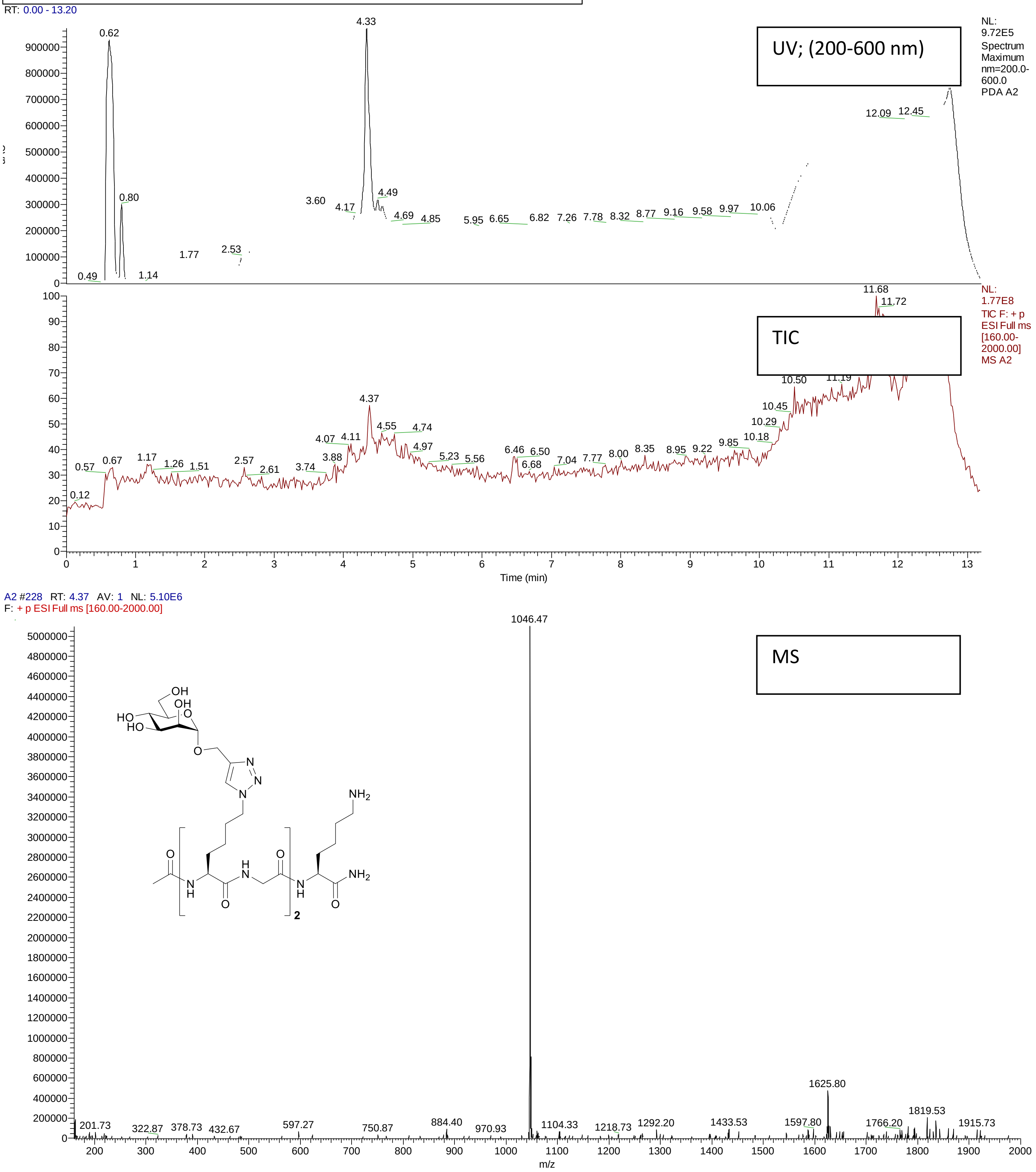

A3

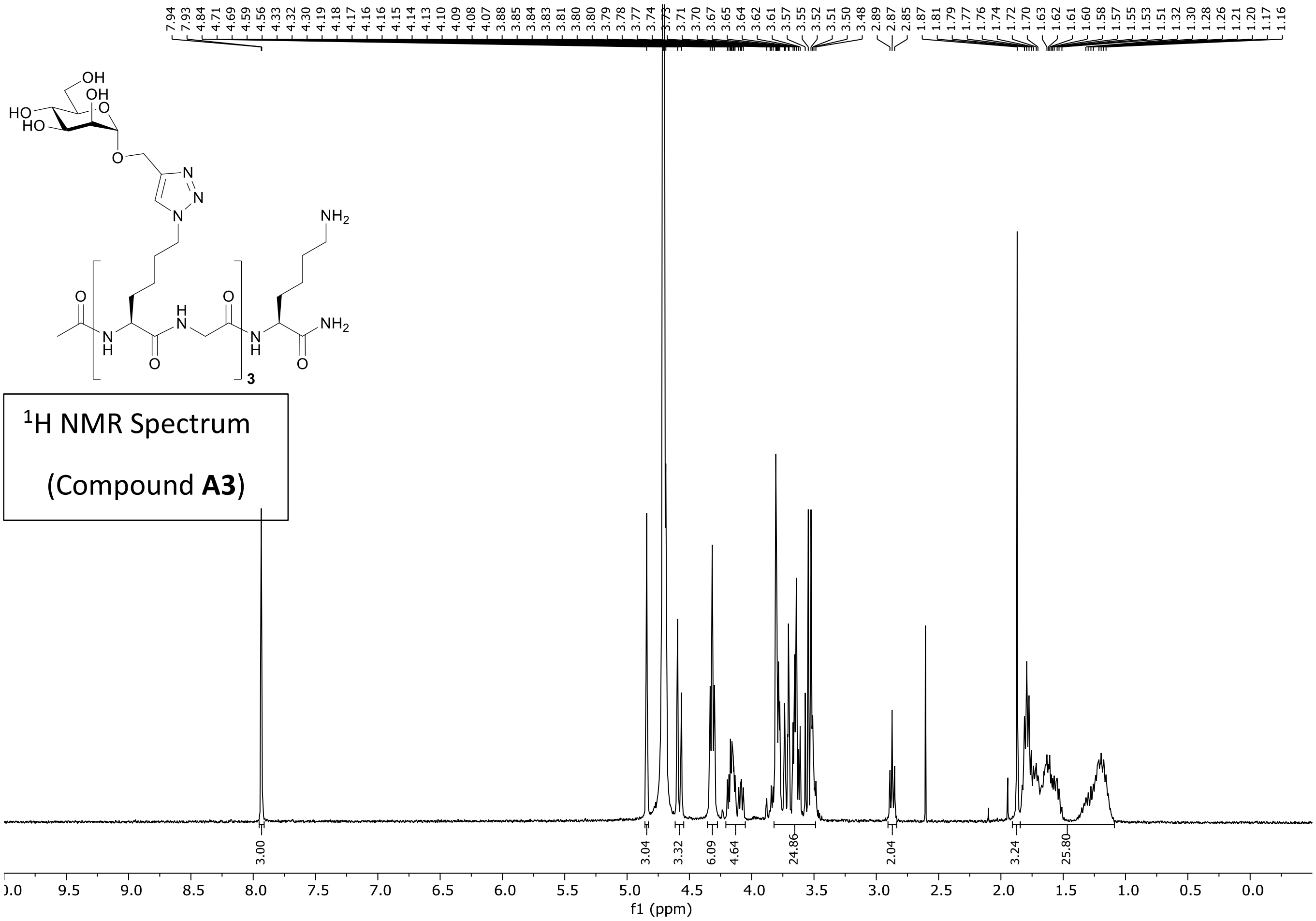

^{1}H NMR Spectrum

(Compound **A3**)

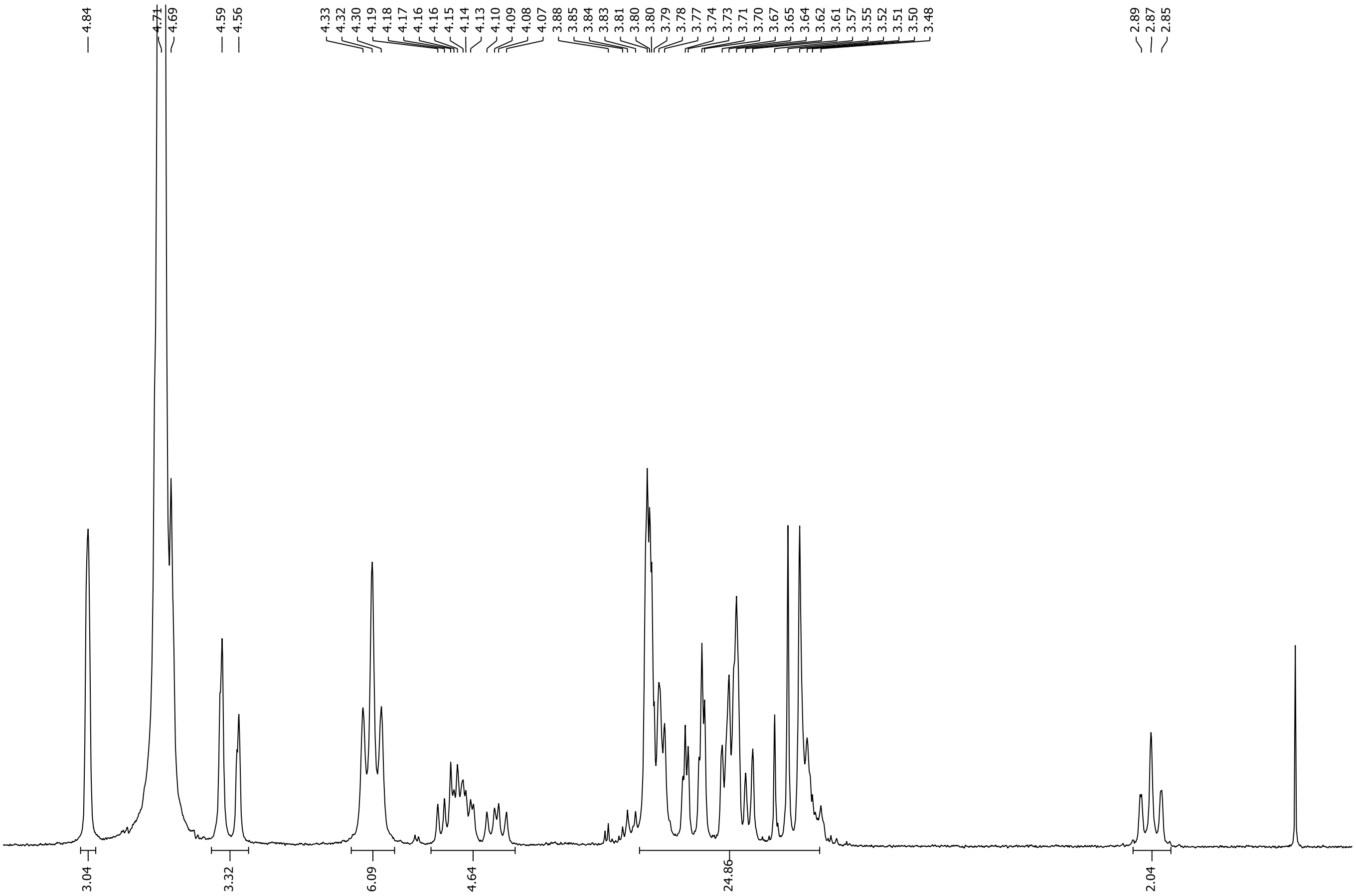

^{13}C APT NMR Spectrum

(Compound **A3**)

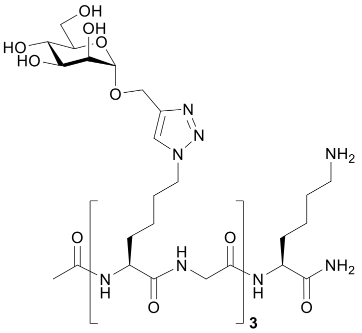

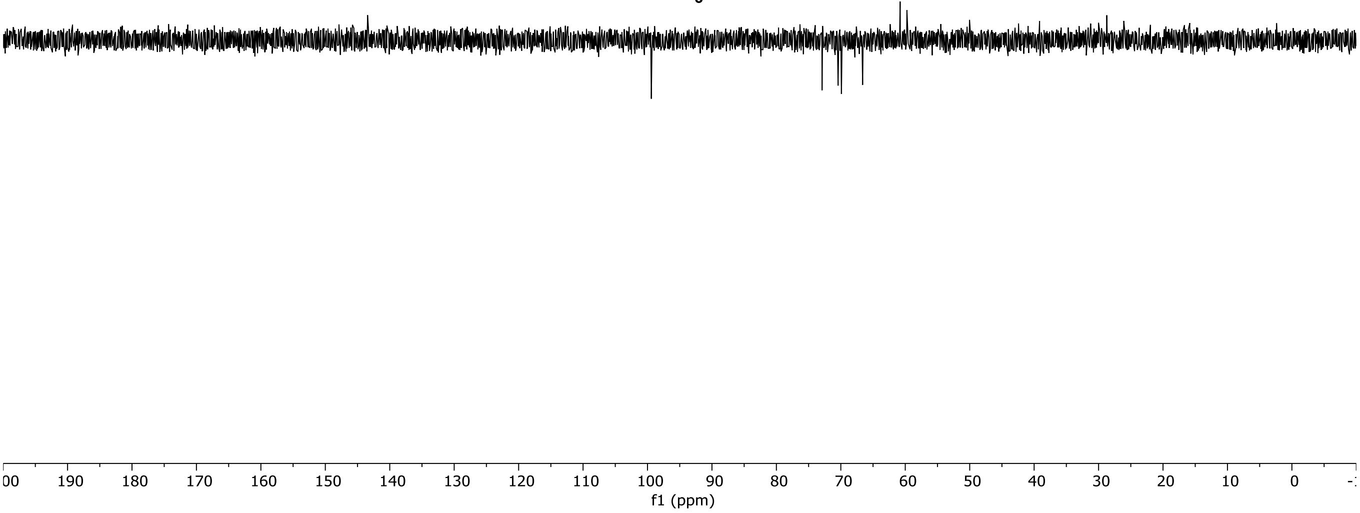

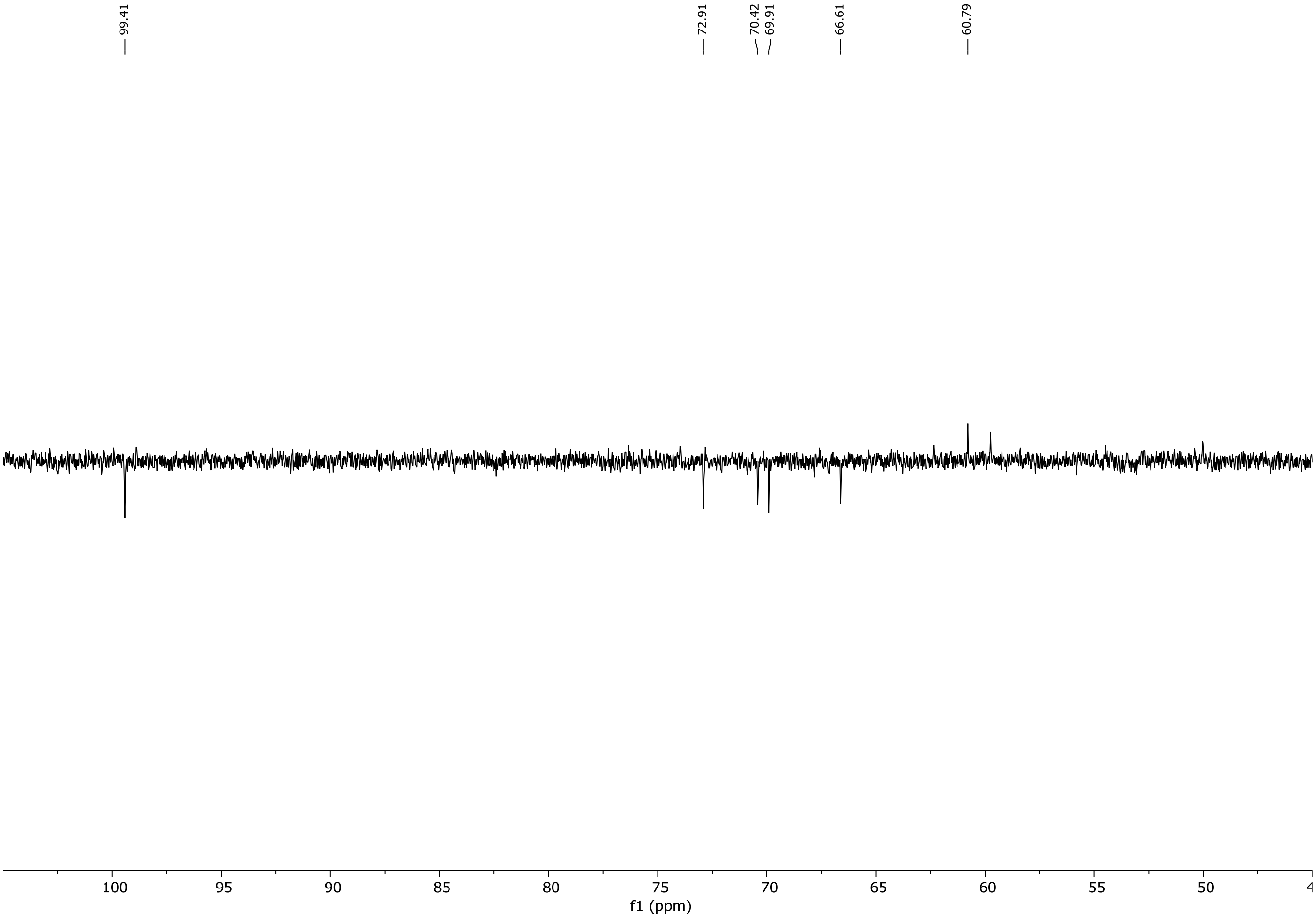

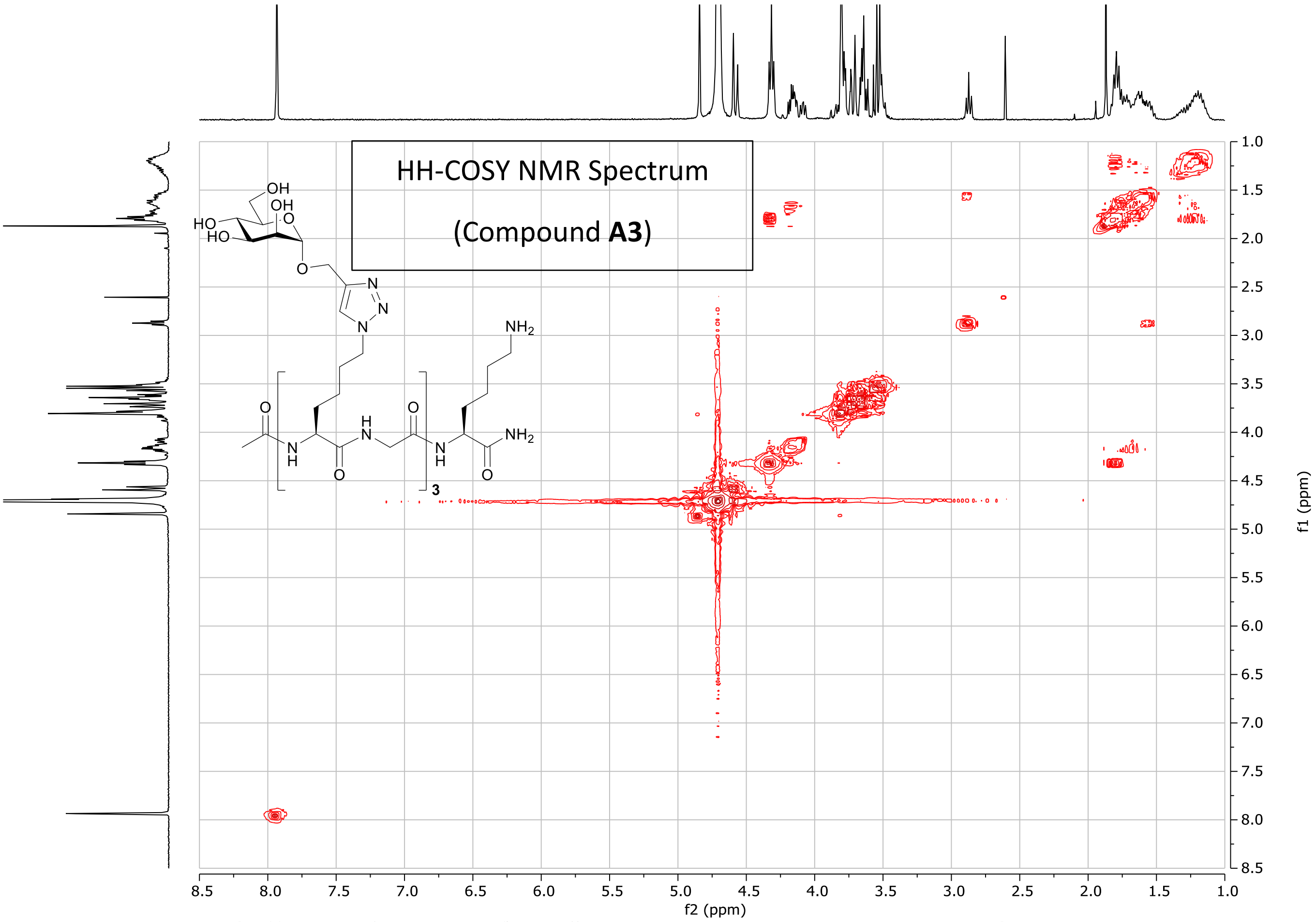
HH-COSY NMR Spectrum
(Compound **A3**)
f2 (ppm)
f1 (ppm)

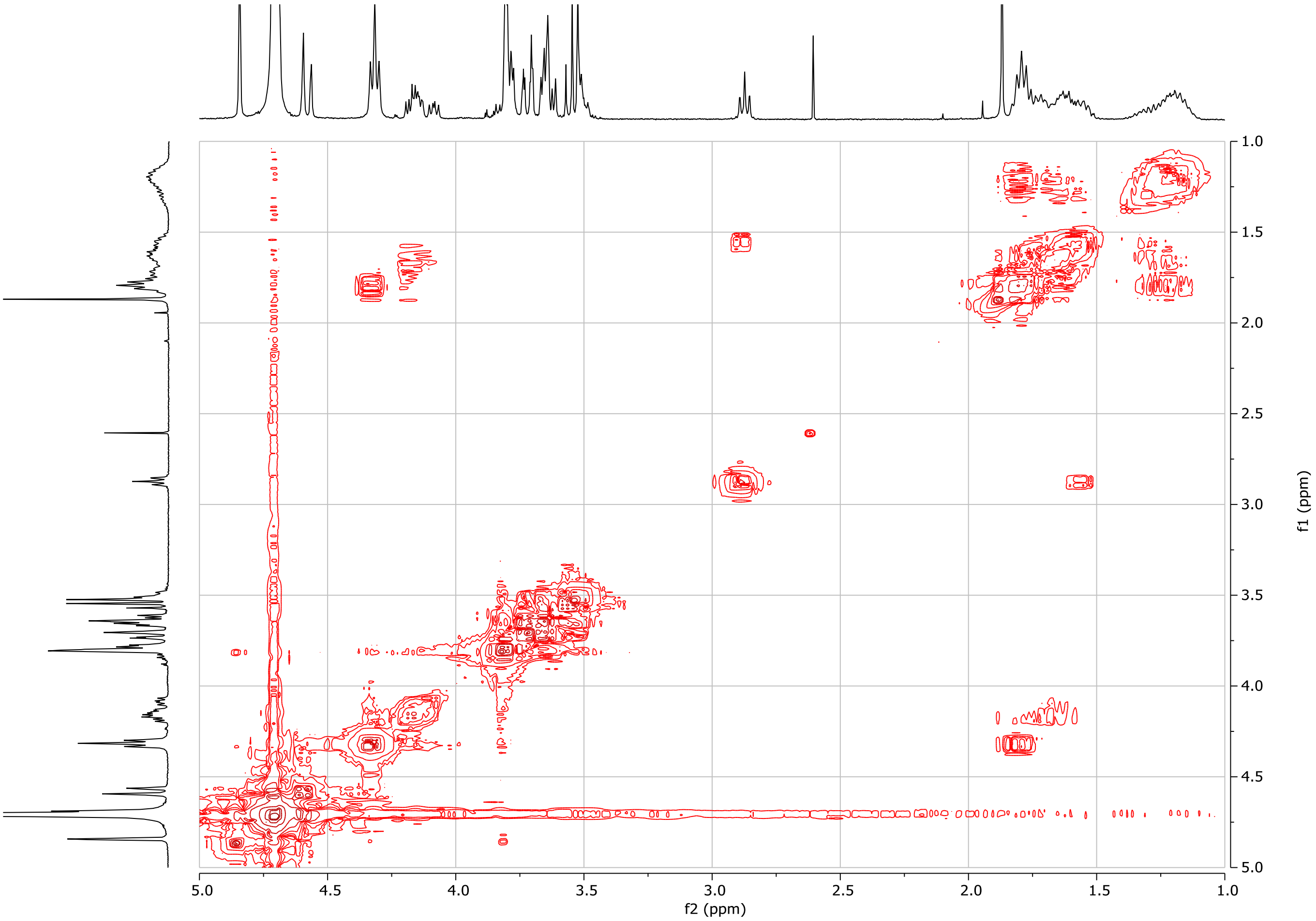
f2 (ppm)
f1 (ppm)

LC-MS Spectra; *(0 –> 50 % ACN, 13 min)*; (Compound **A3**)

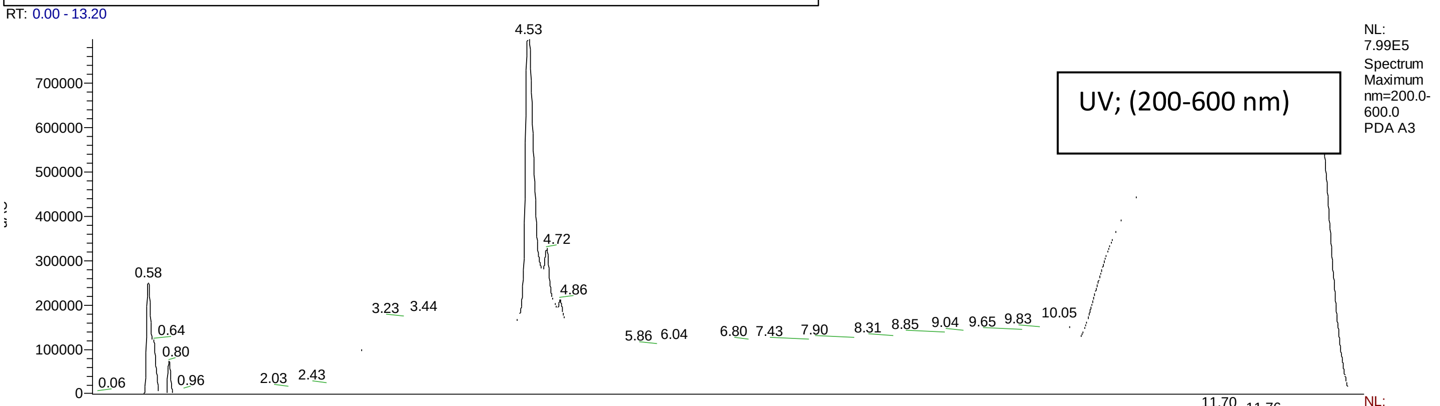

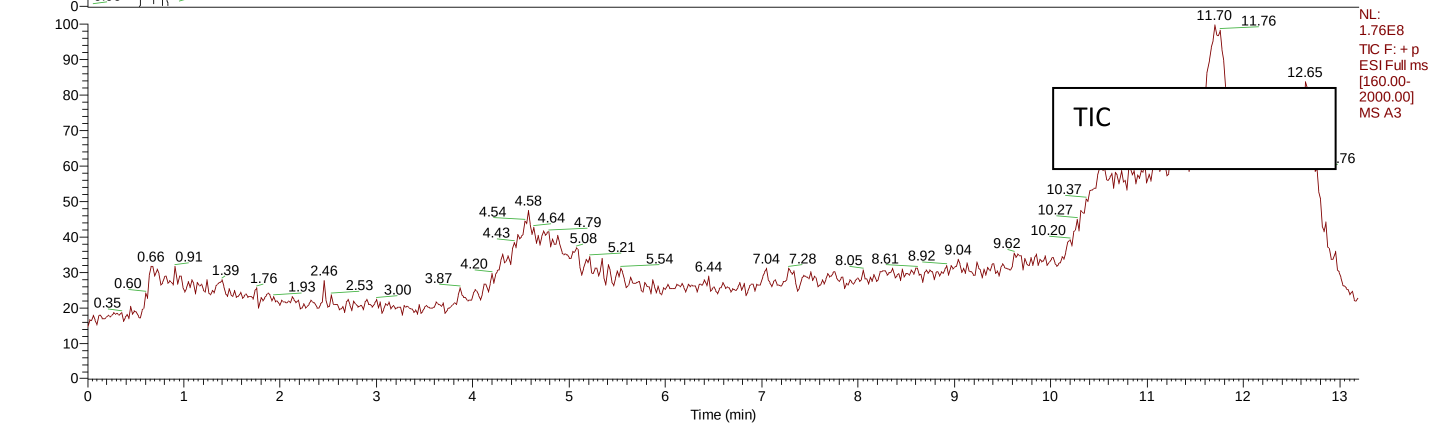

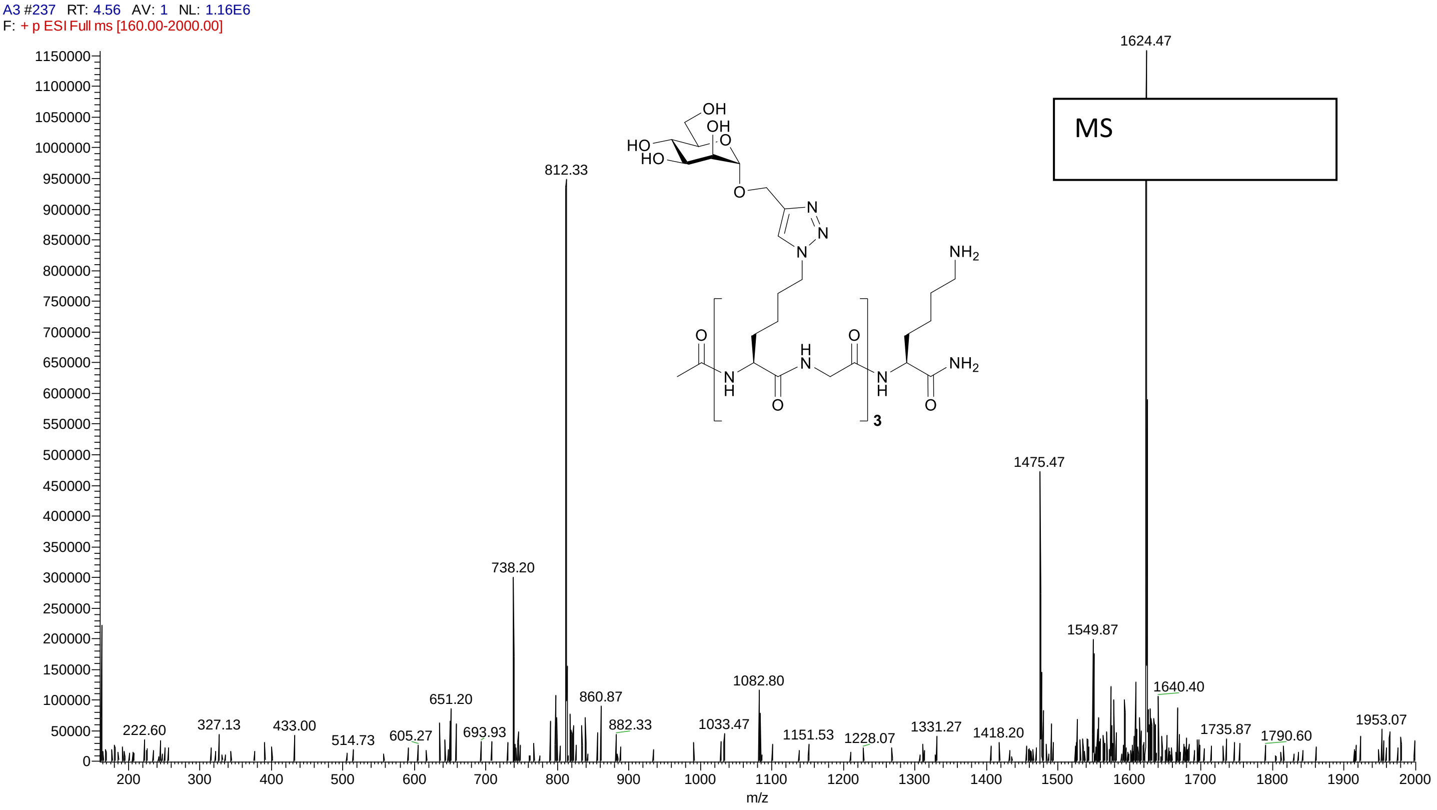

A6

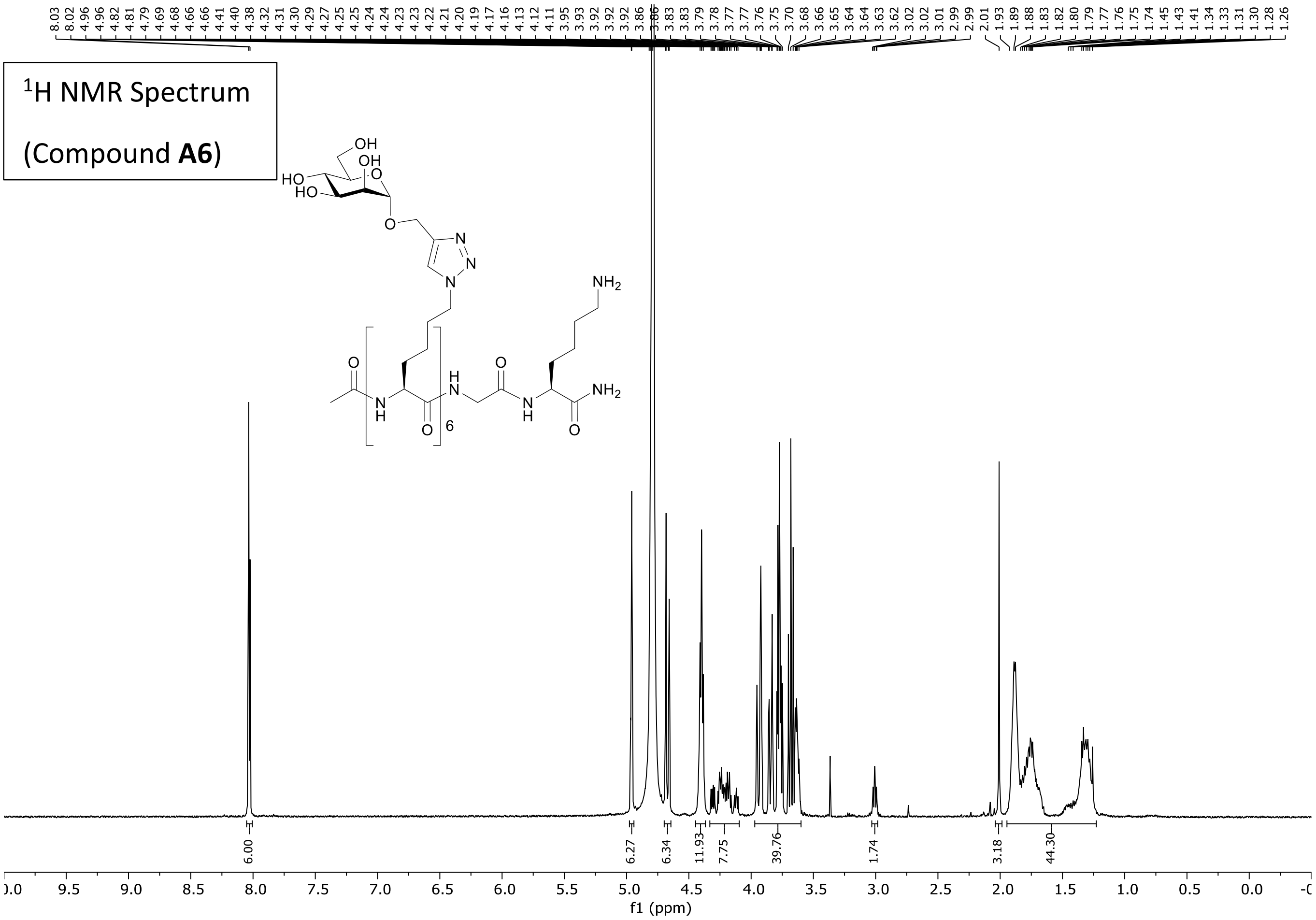

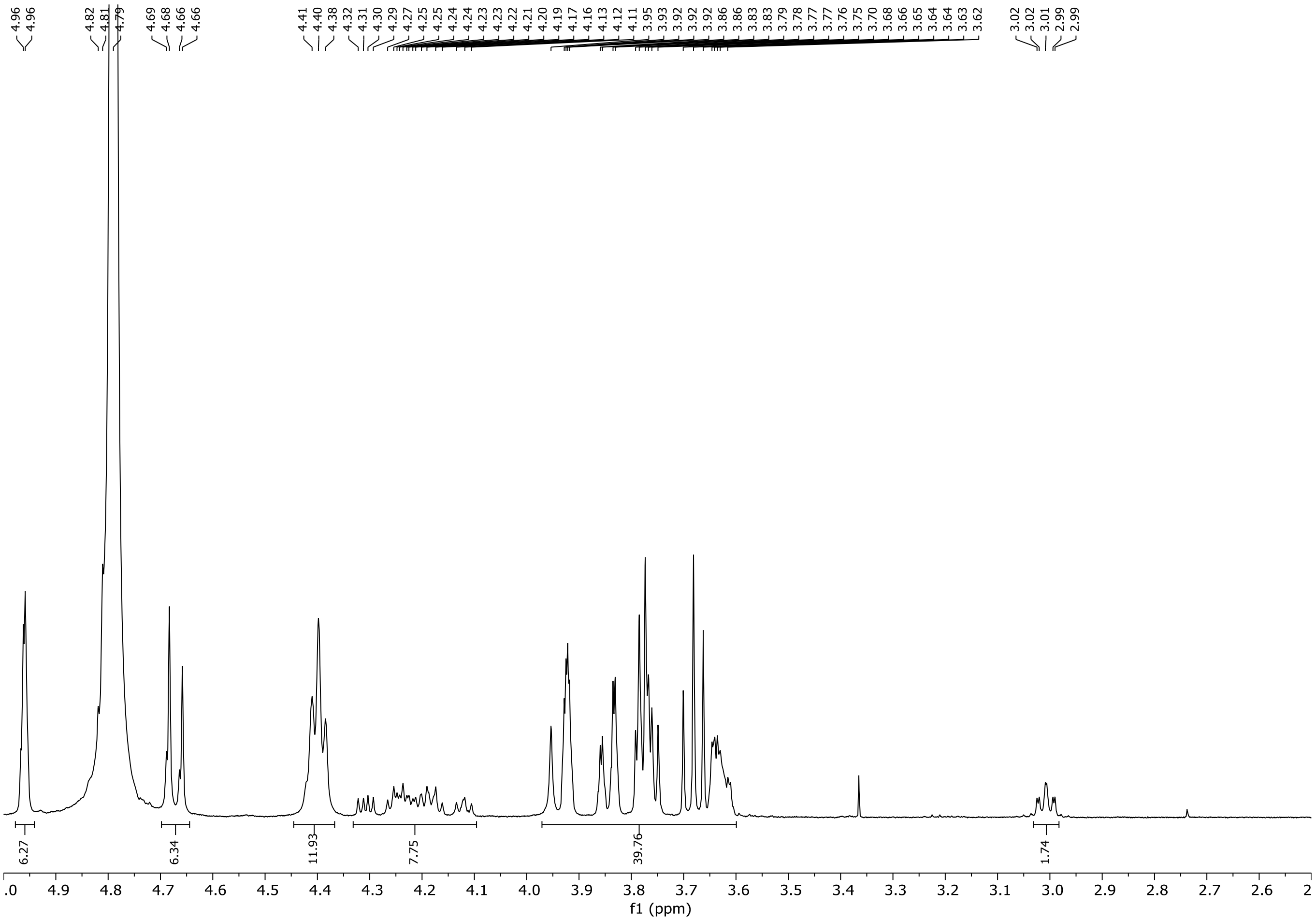

^{13}C APT NMR Spectrum

(Compound **A6**)

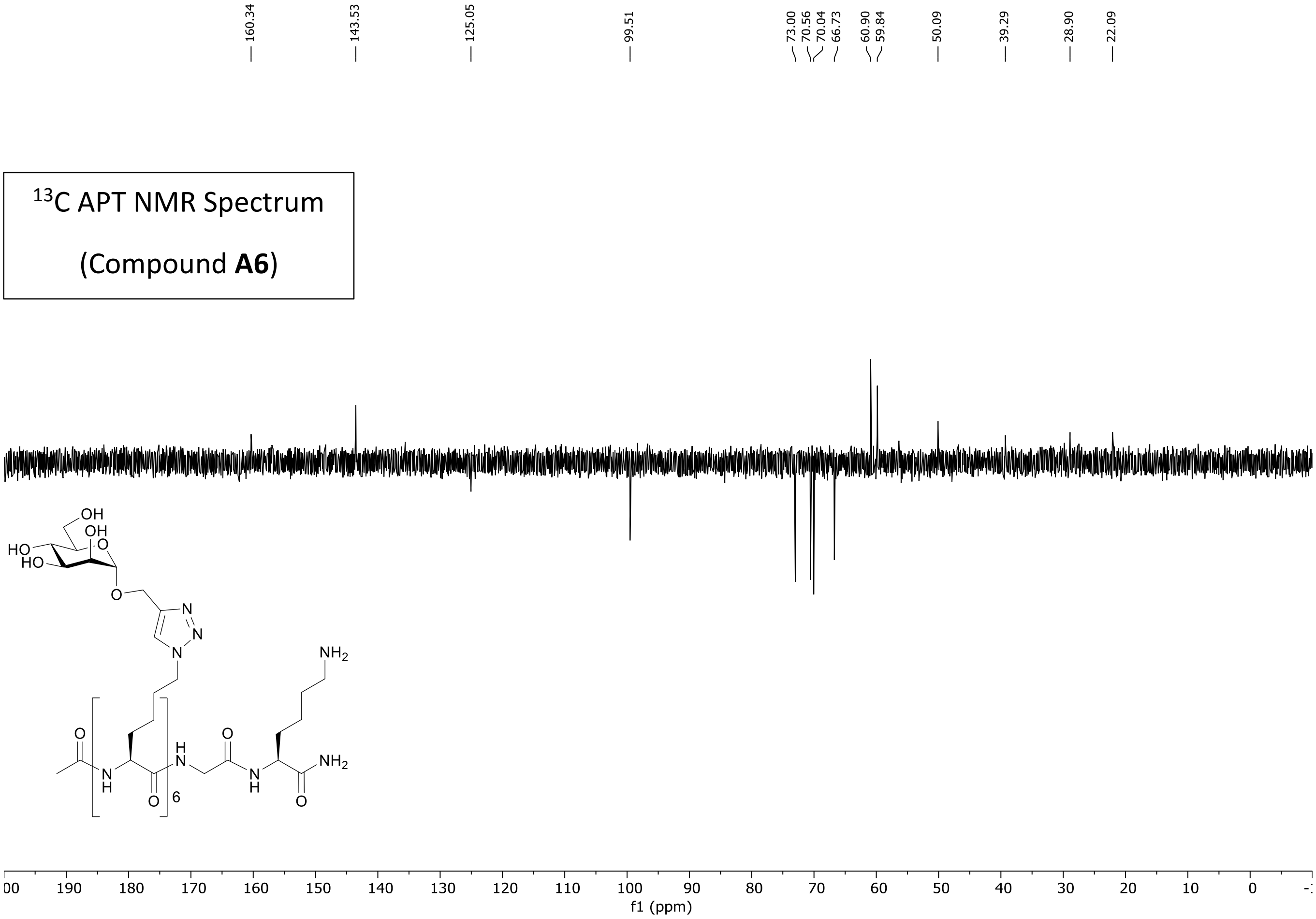

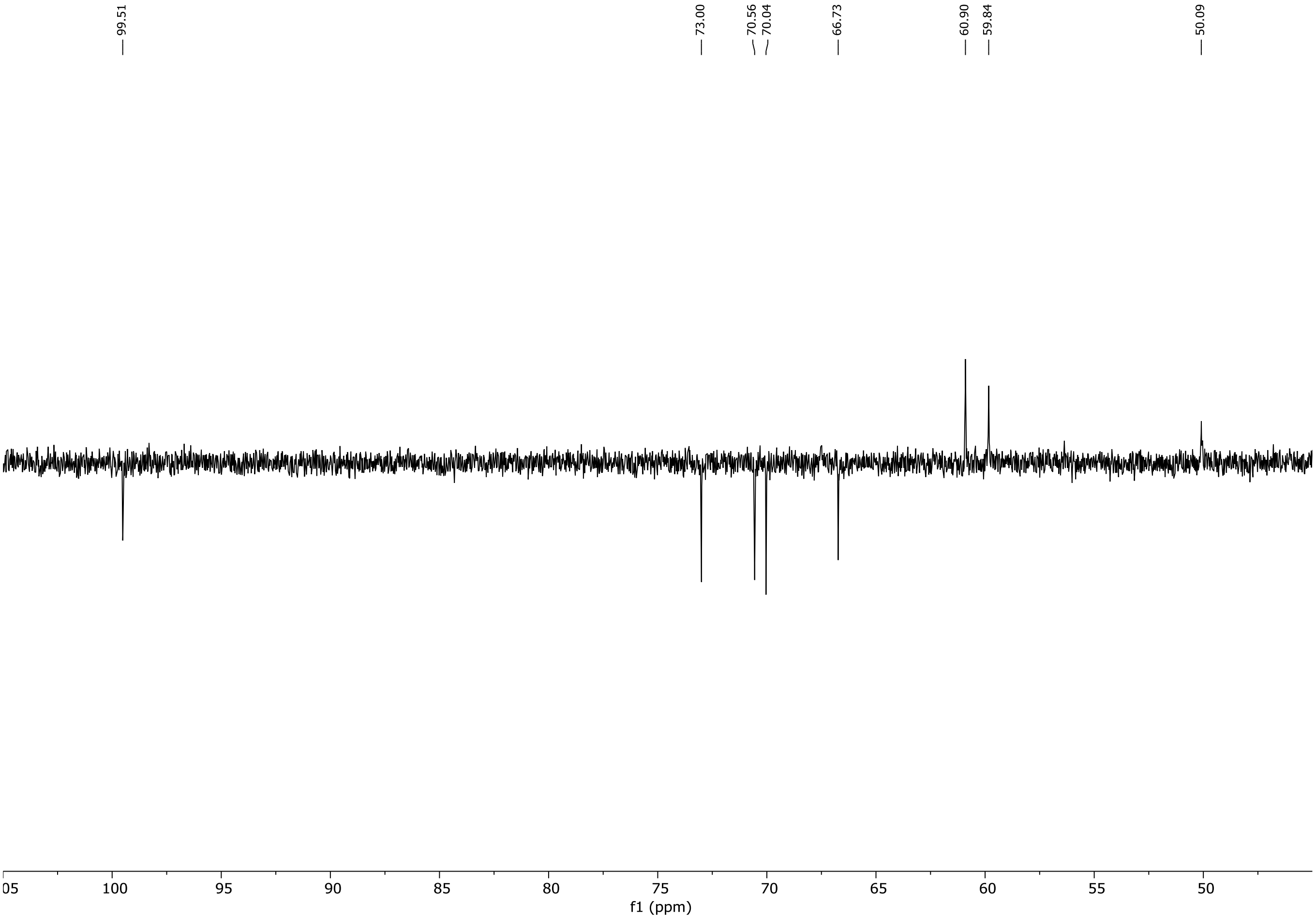

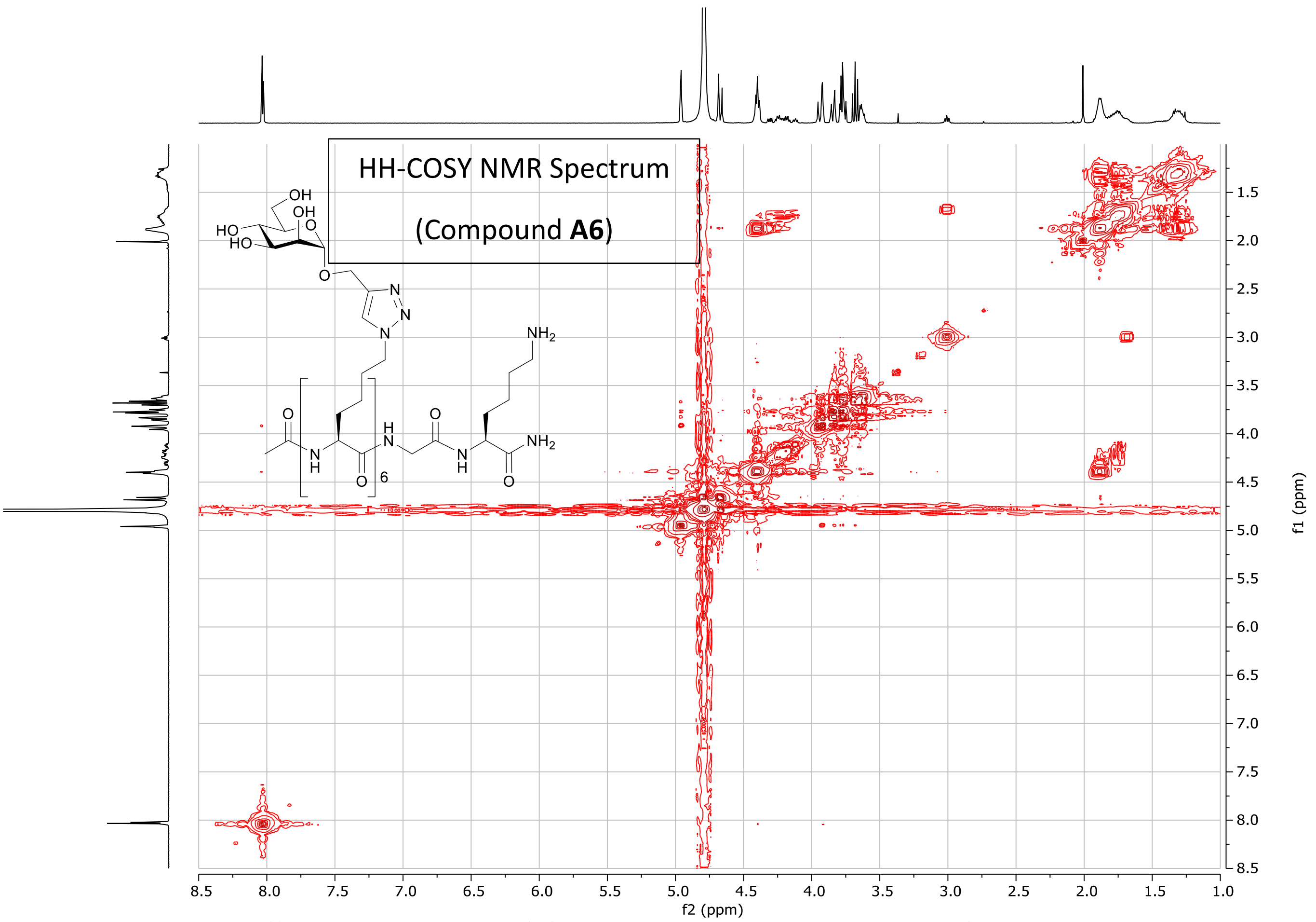
HH-COSY NMR Spectrum
(Compound **A6**)
f2 (ppm)
f1 (ppm)

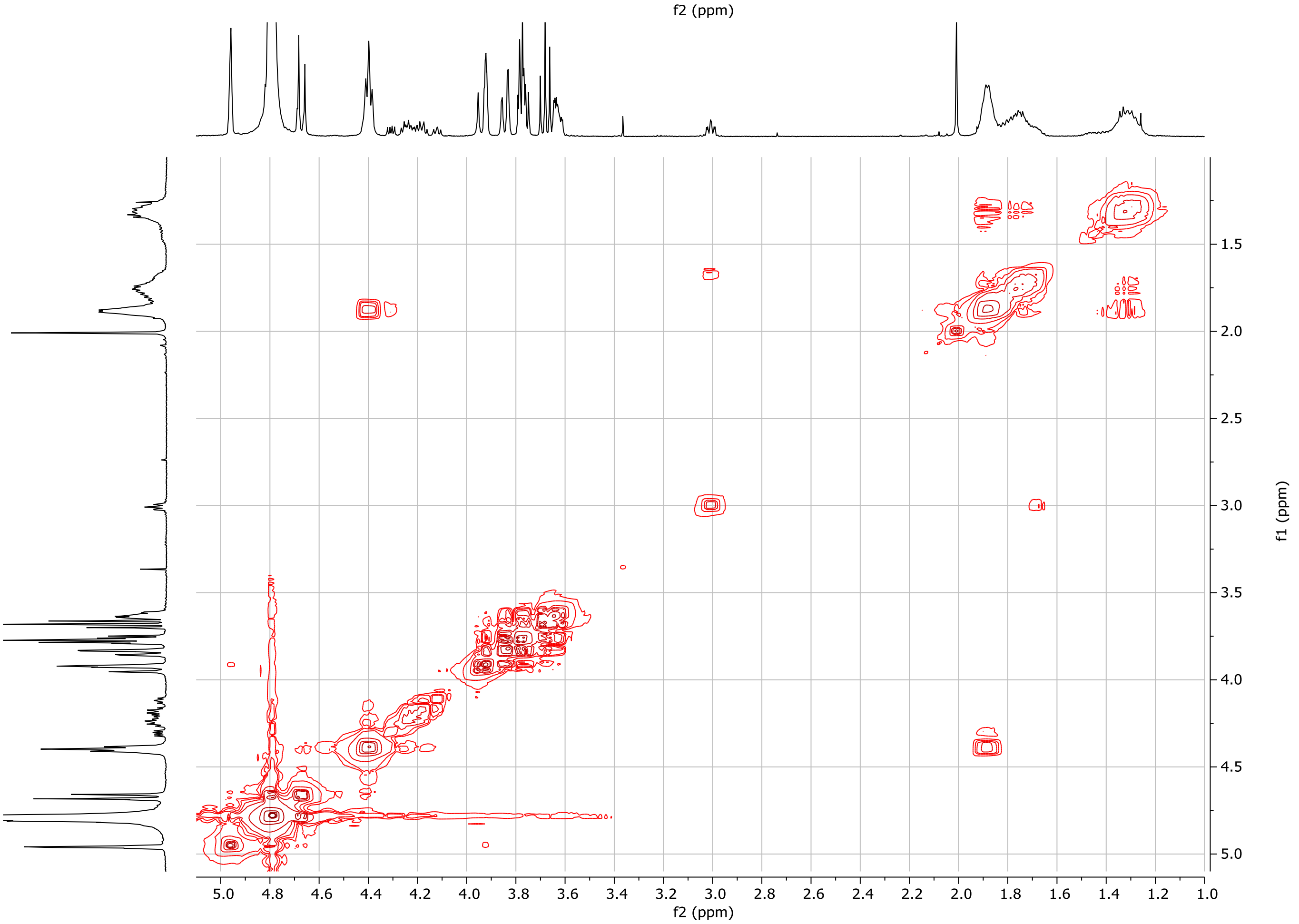
f2 (ppm)
f1 (ppm)

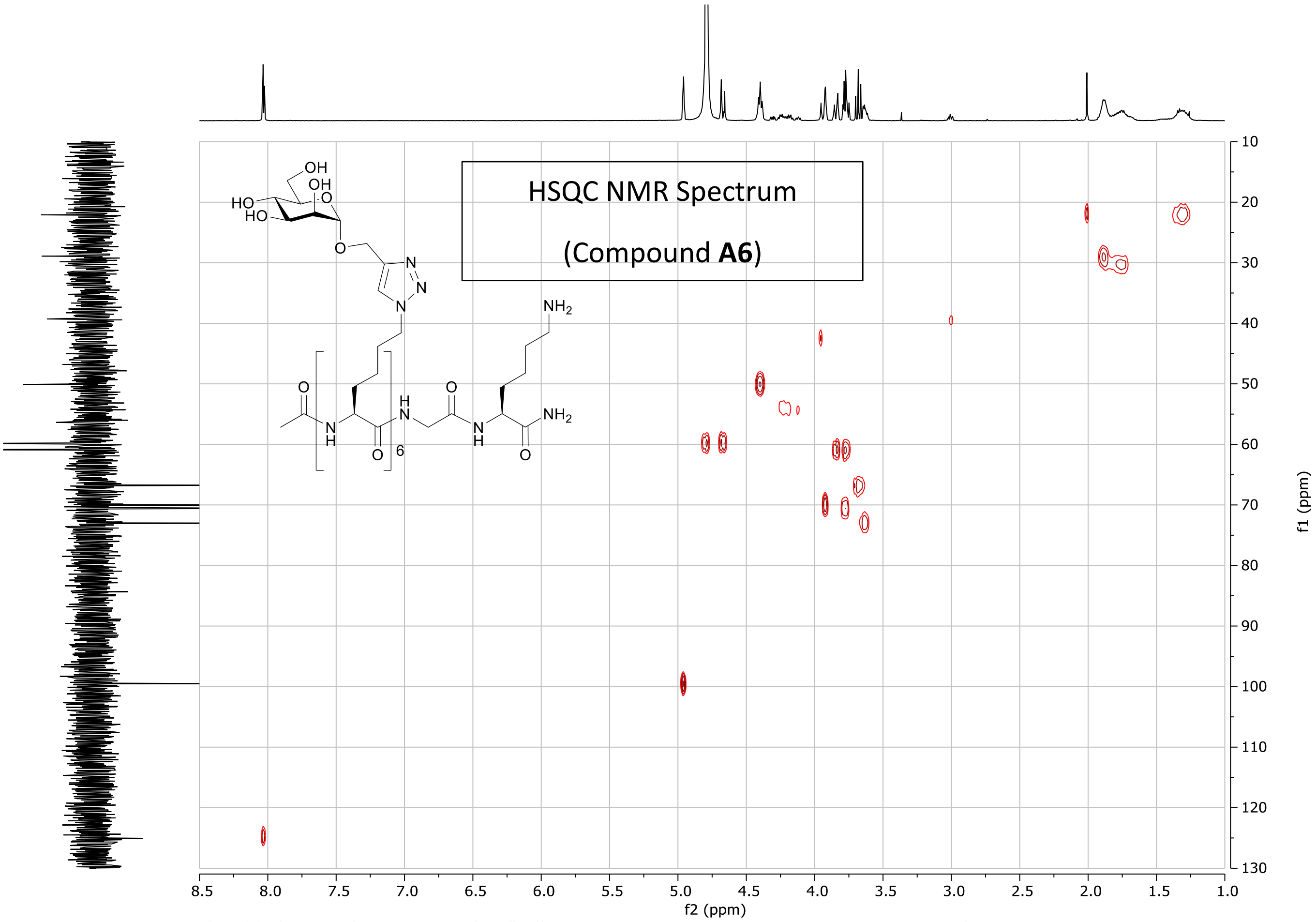
HSQC NMR Spectrum
(Compound A6)
OH
OH
HO
HO
O
O
N
N
N
NH2
O
N
H
O
6
H
N
O
N
H
NH2
O
f1 (ppm)
f2 (ppm)

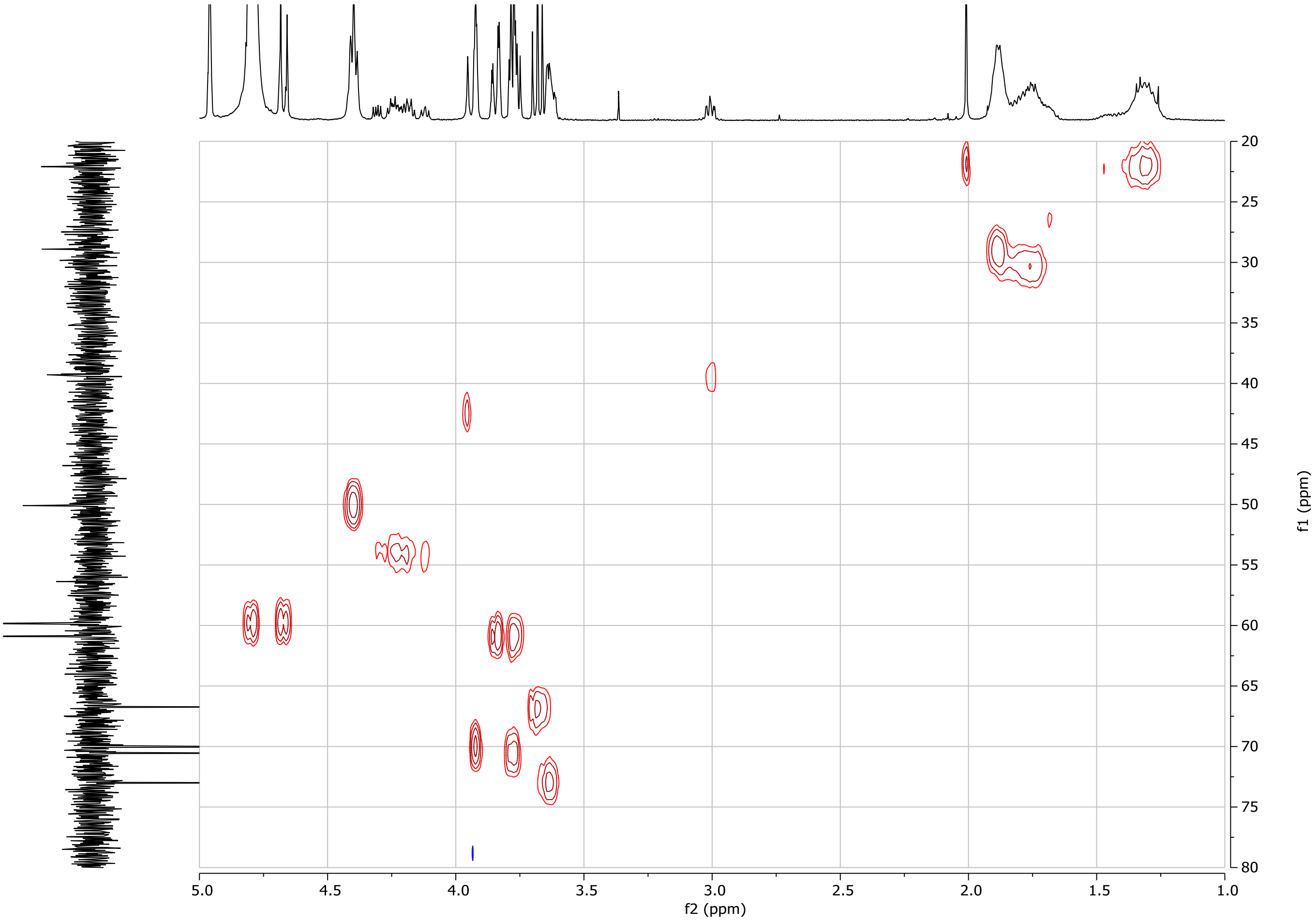
f1 (ppm)
f2 (ppm)

LC-MS Spectra; *(0 –> 50 % ACN, 13 min)*; (Compound **A6**)

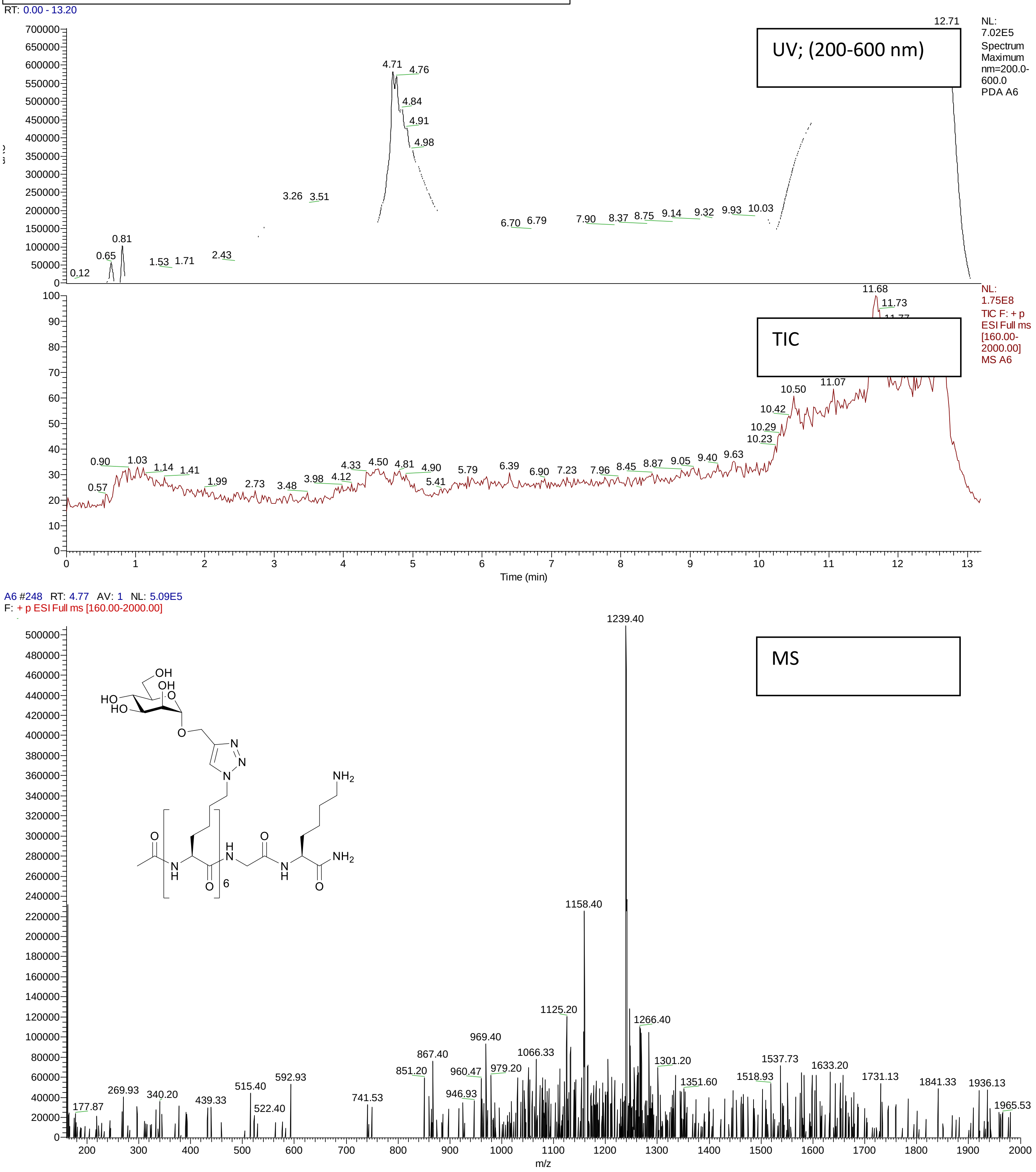

B1

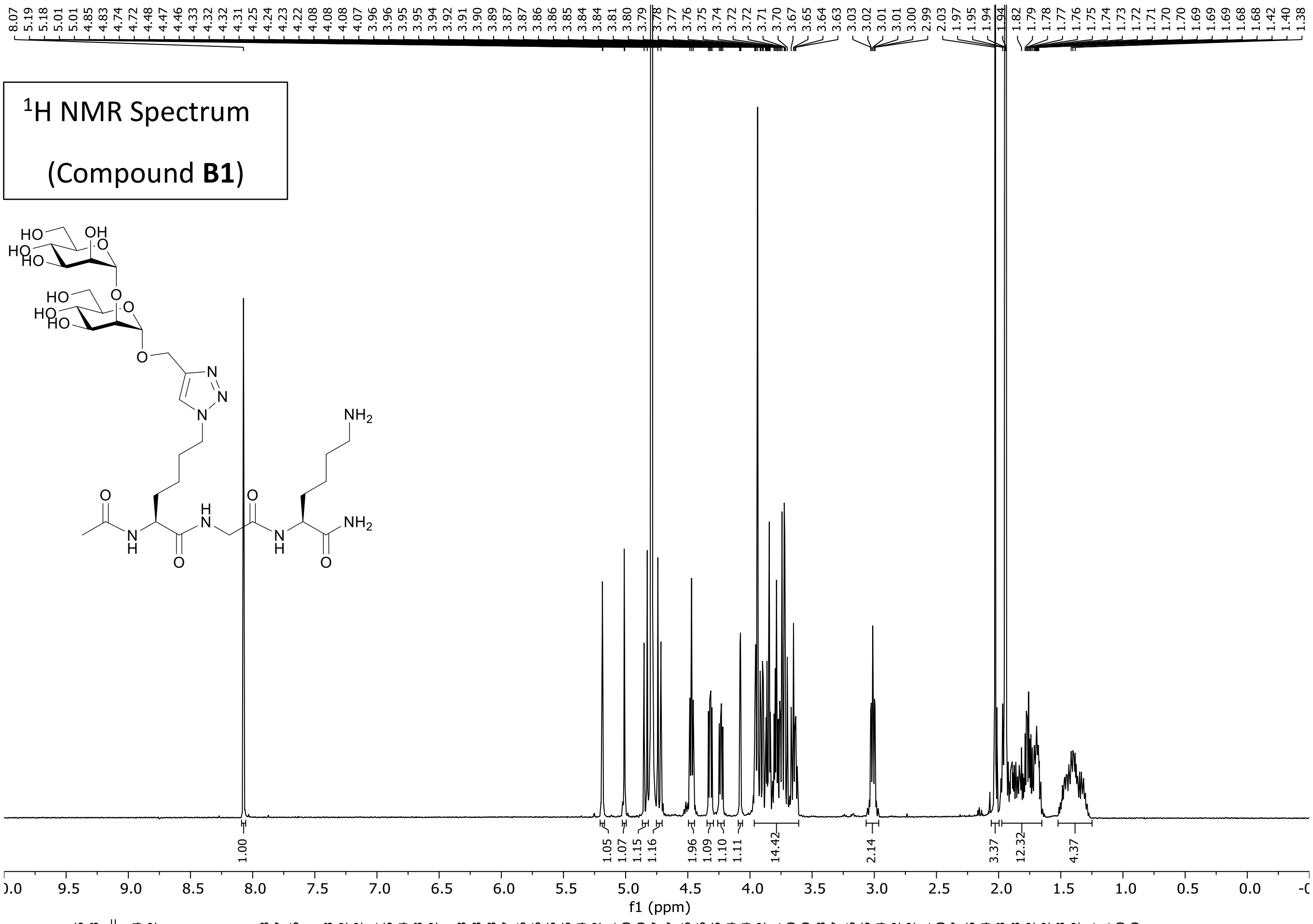

^{1}H NMR Spectrum
(Compound **B1**)
HO
OH
HO
HO
O
O
HO
HO
HO
O
O
N
N
N
NH2
O
H
N
NH
O
NH2
f1 (ppm)

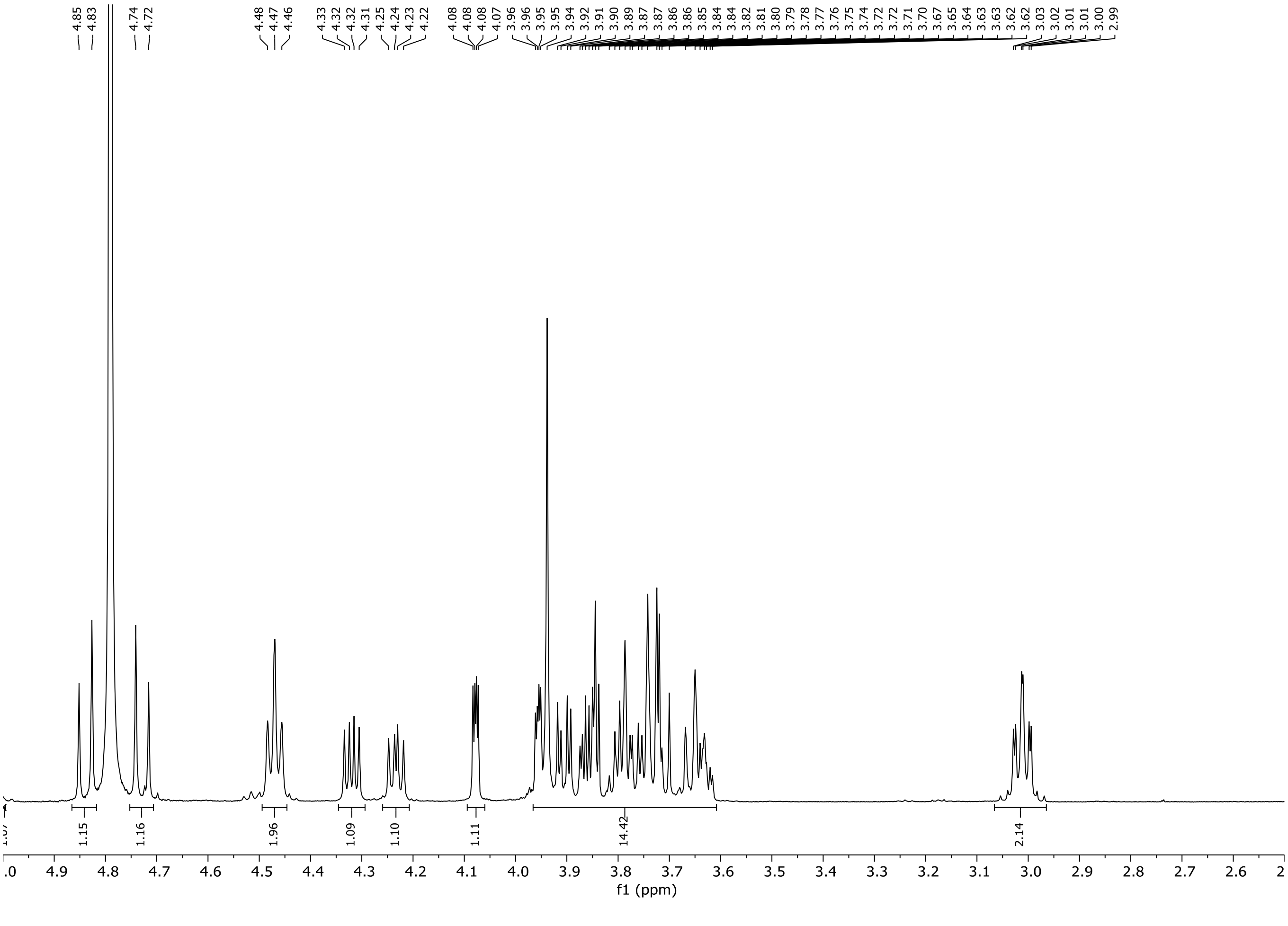

f1 (ppm)

LC-MS Spectra; *(0 –> 50 % ACN, 13 min);* (Compound **B1**)

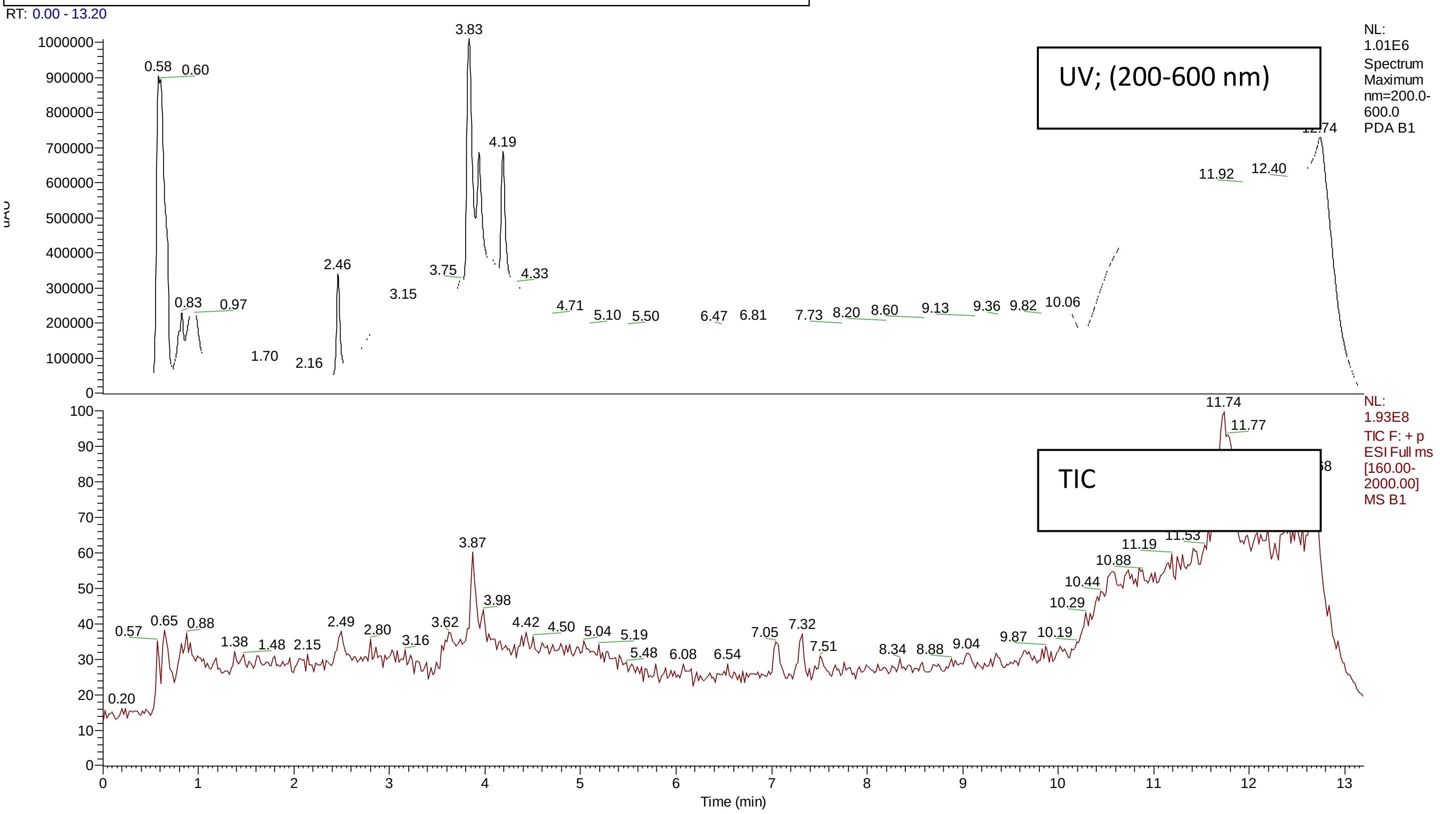

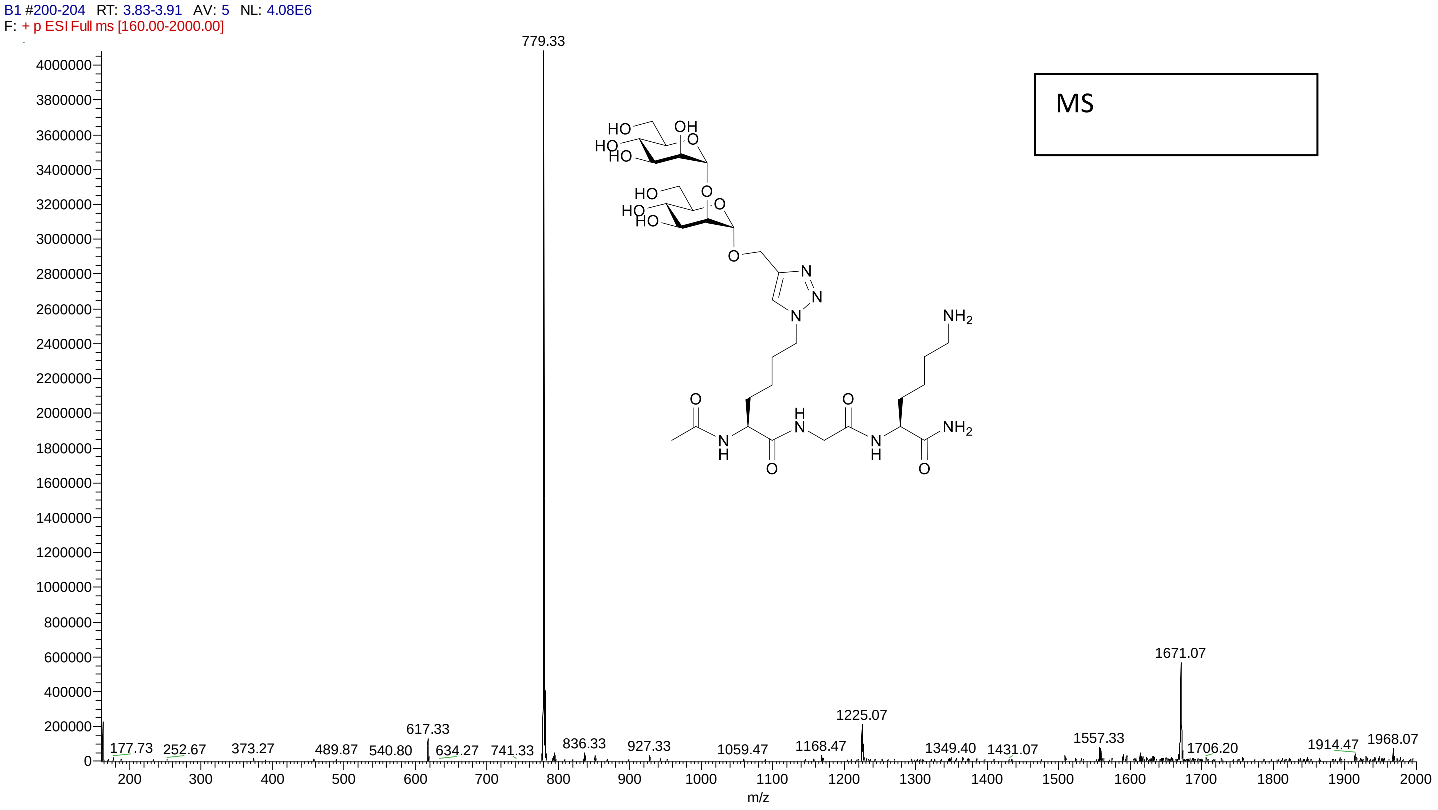

B2

LC-MS Spectra; *(0 –> 50 % ACN, 13 min);* (Compound **B2**)

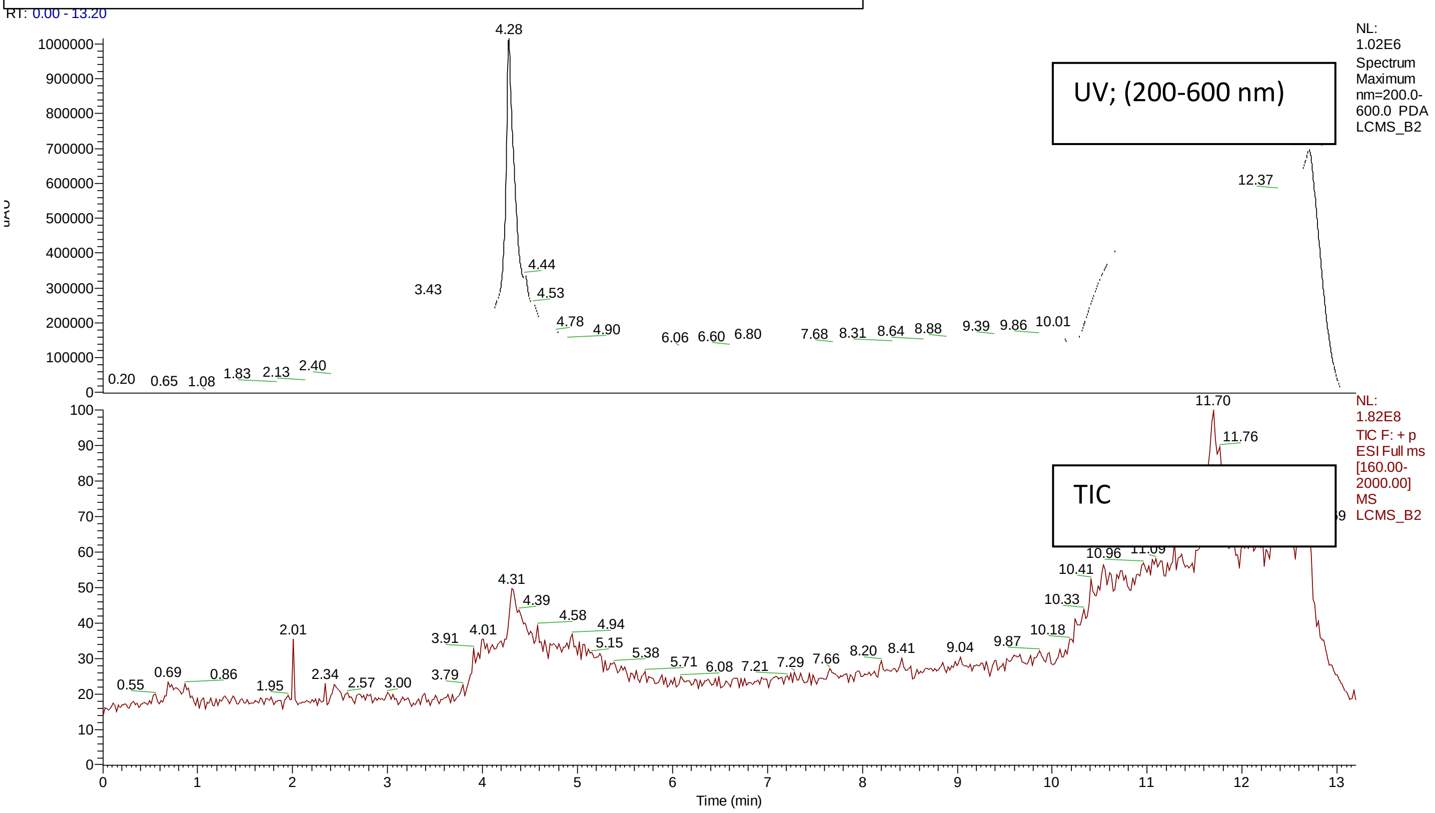

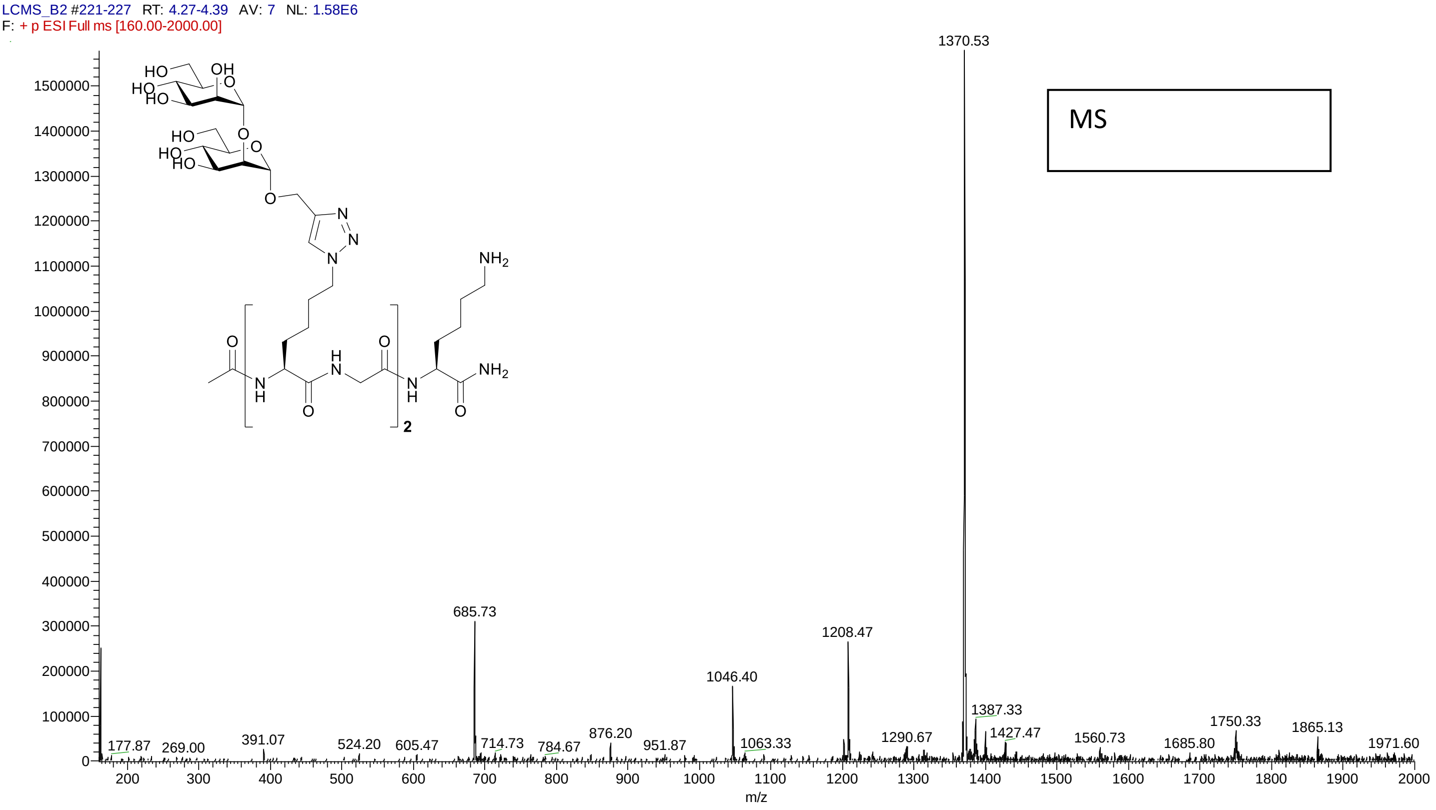

B3

^{1}H NMR Spectrum

(Compound **B3**)

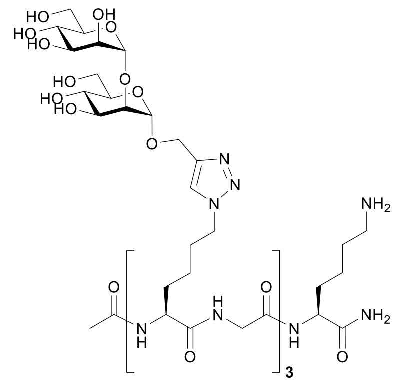

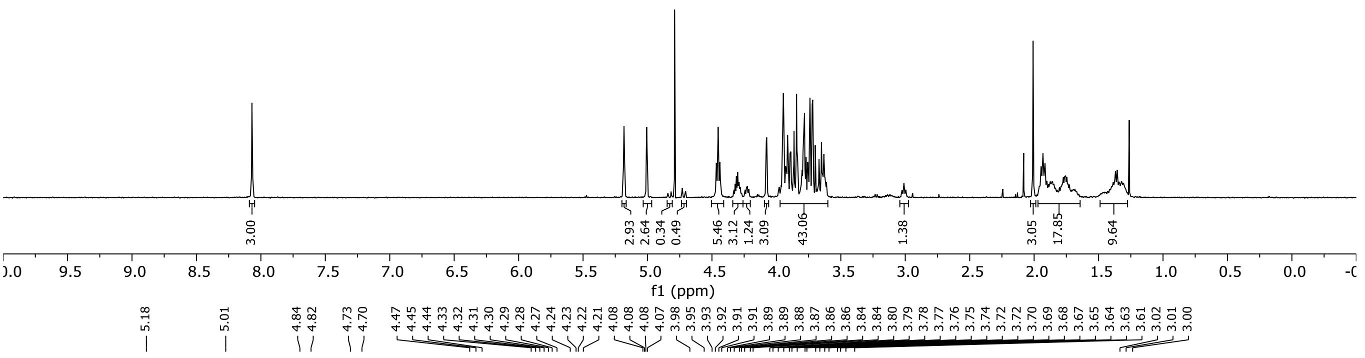

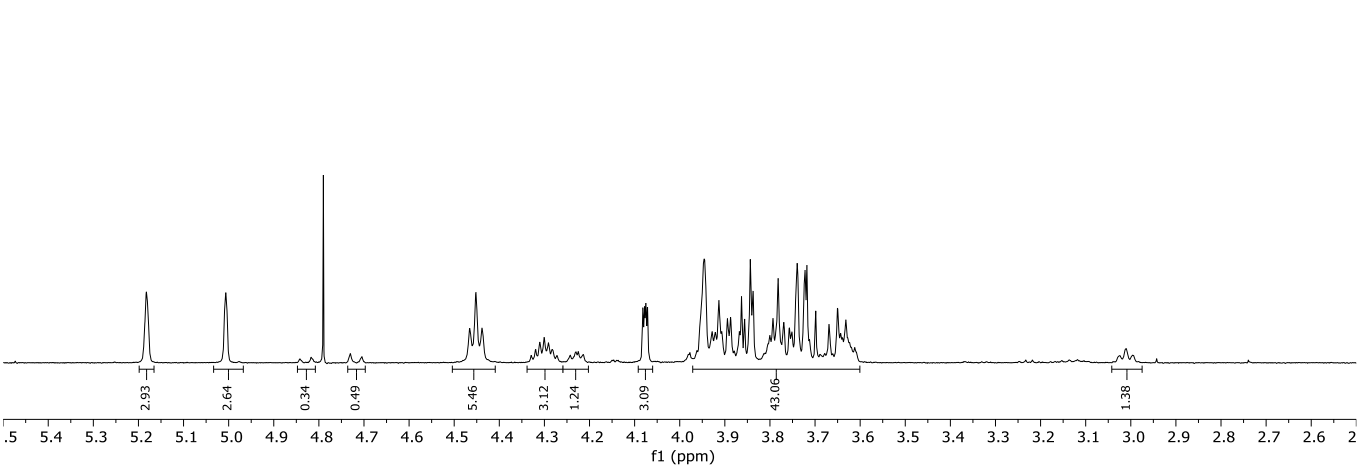

LC-MS Spectra; *(0 –> 50 % ACN, 13 min)*; (Compound **B3**)

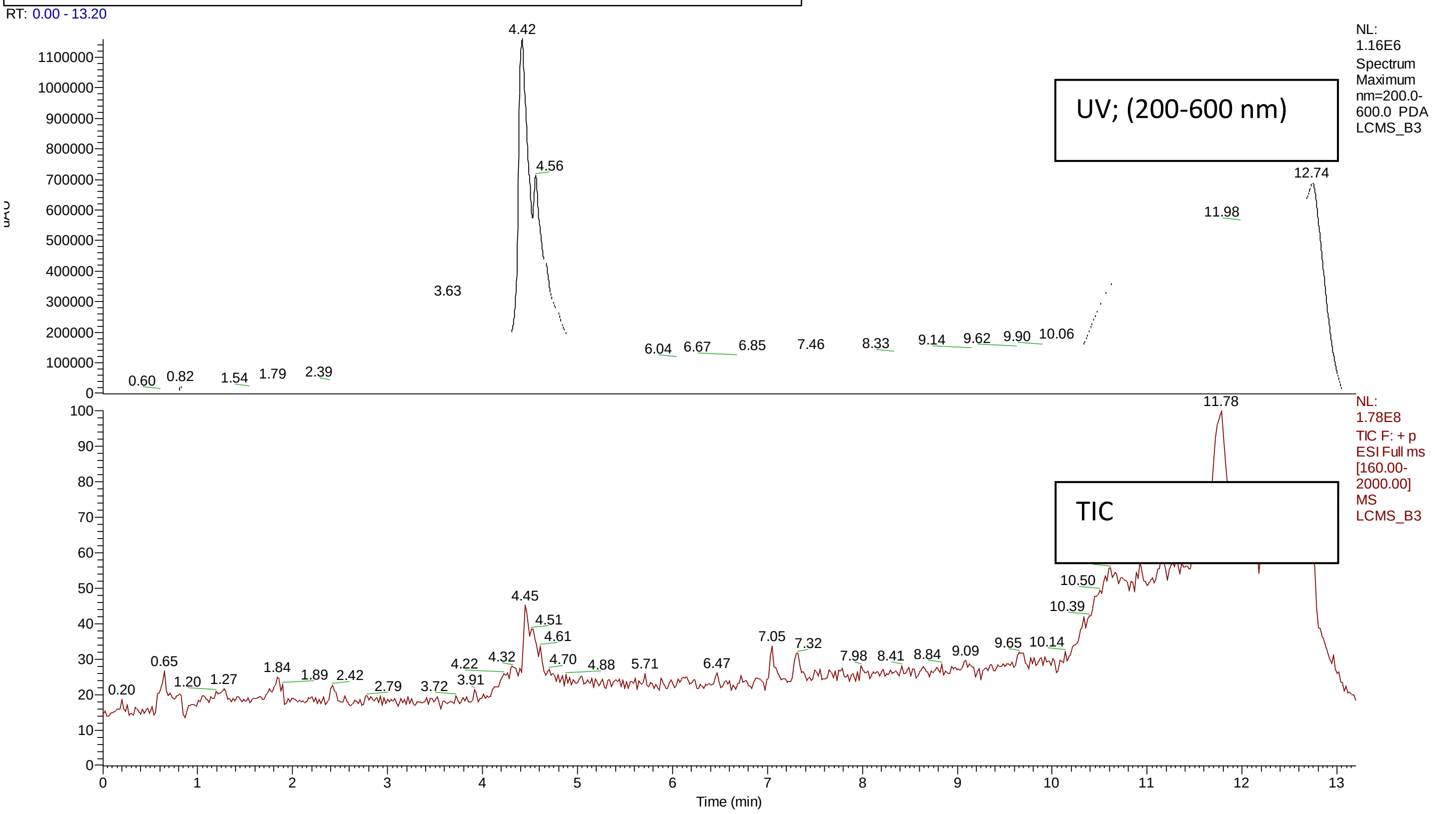

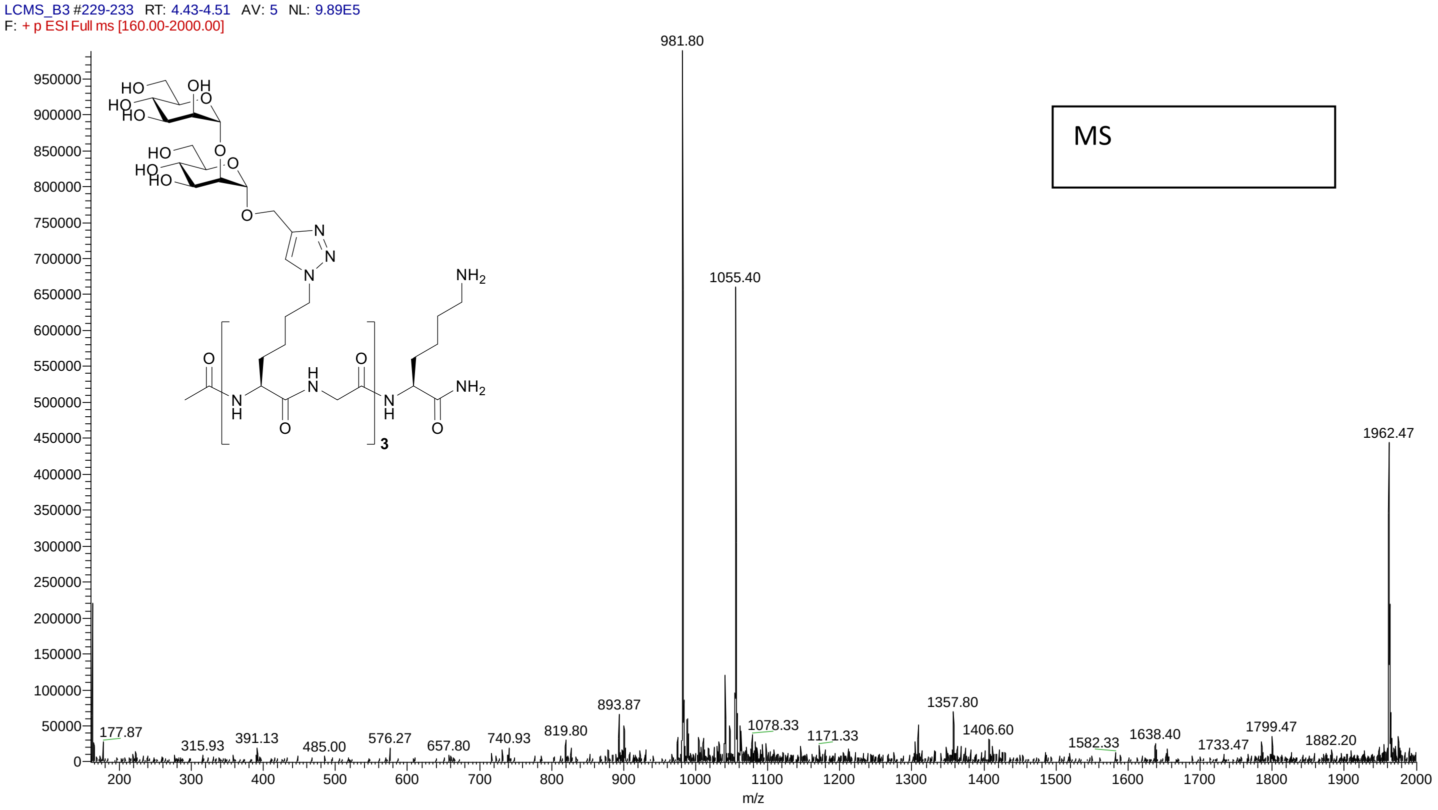

B6

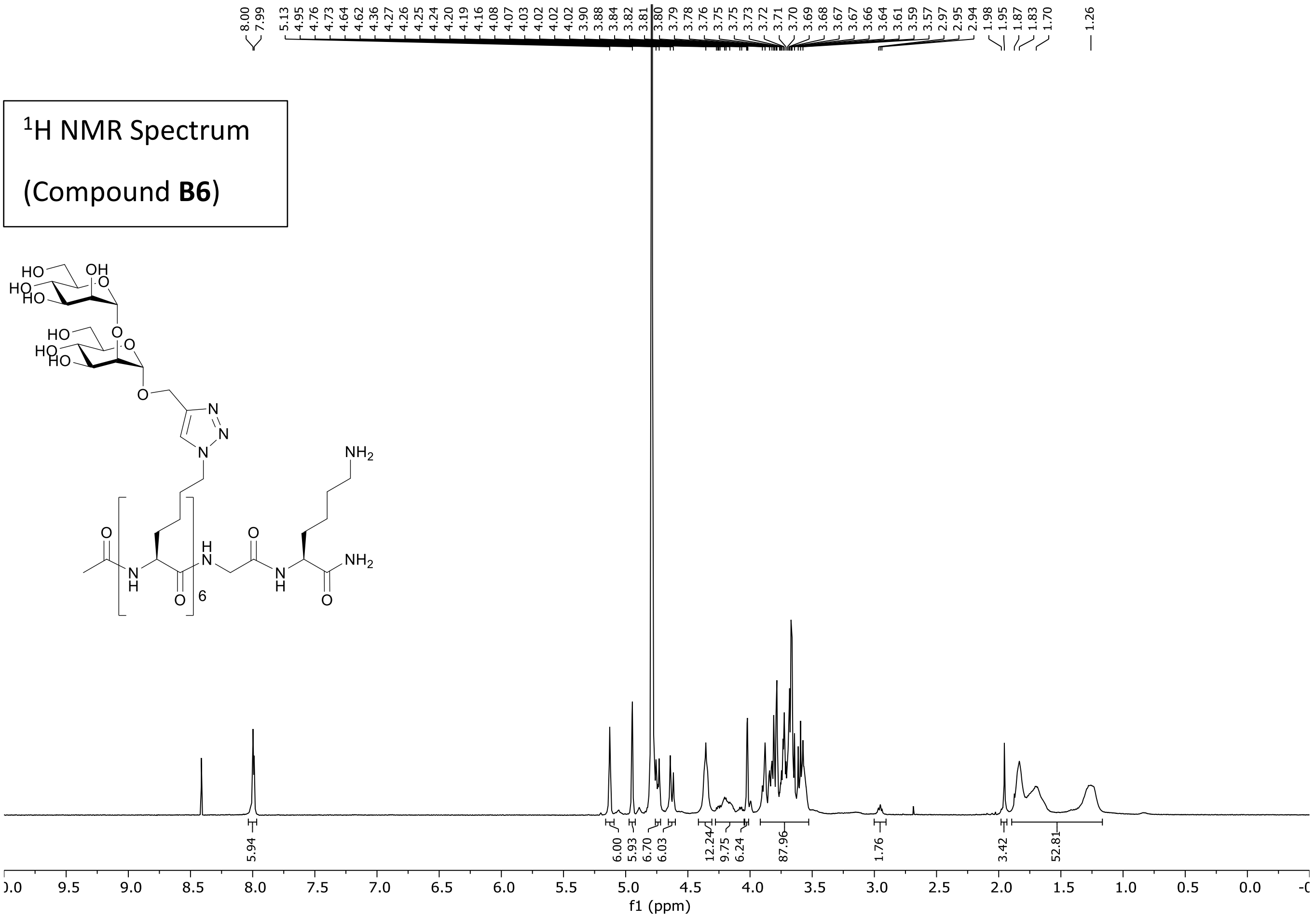

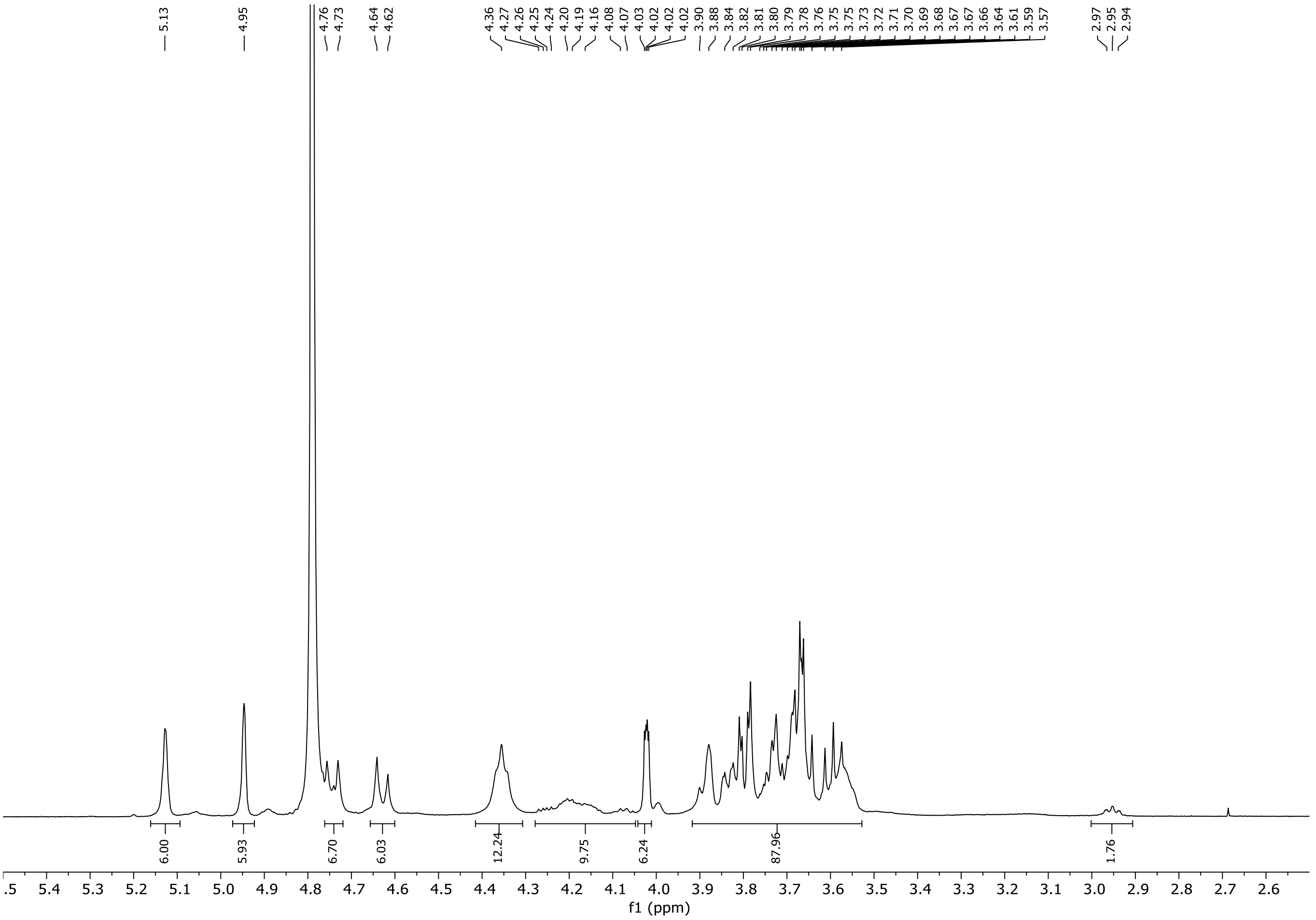

^{13}C APT NMR Spectrum

(Compound **B6**)

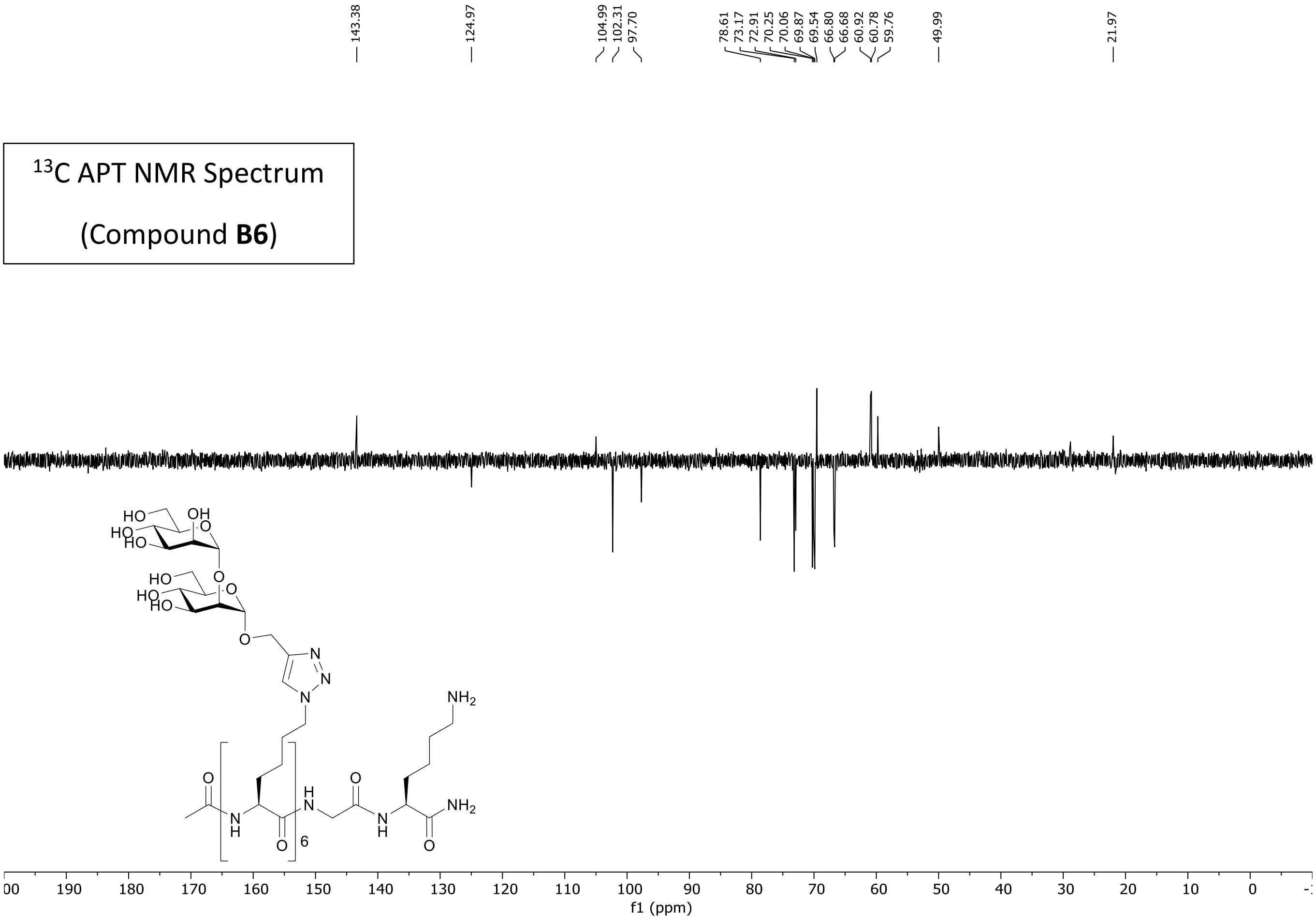

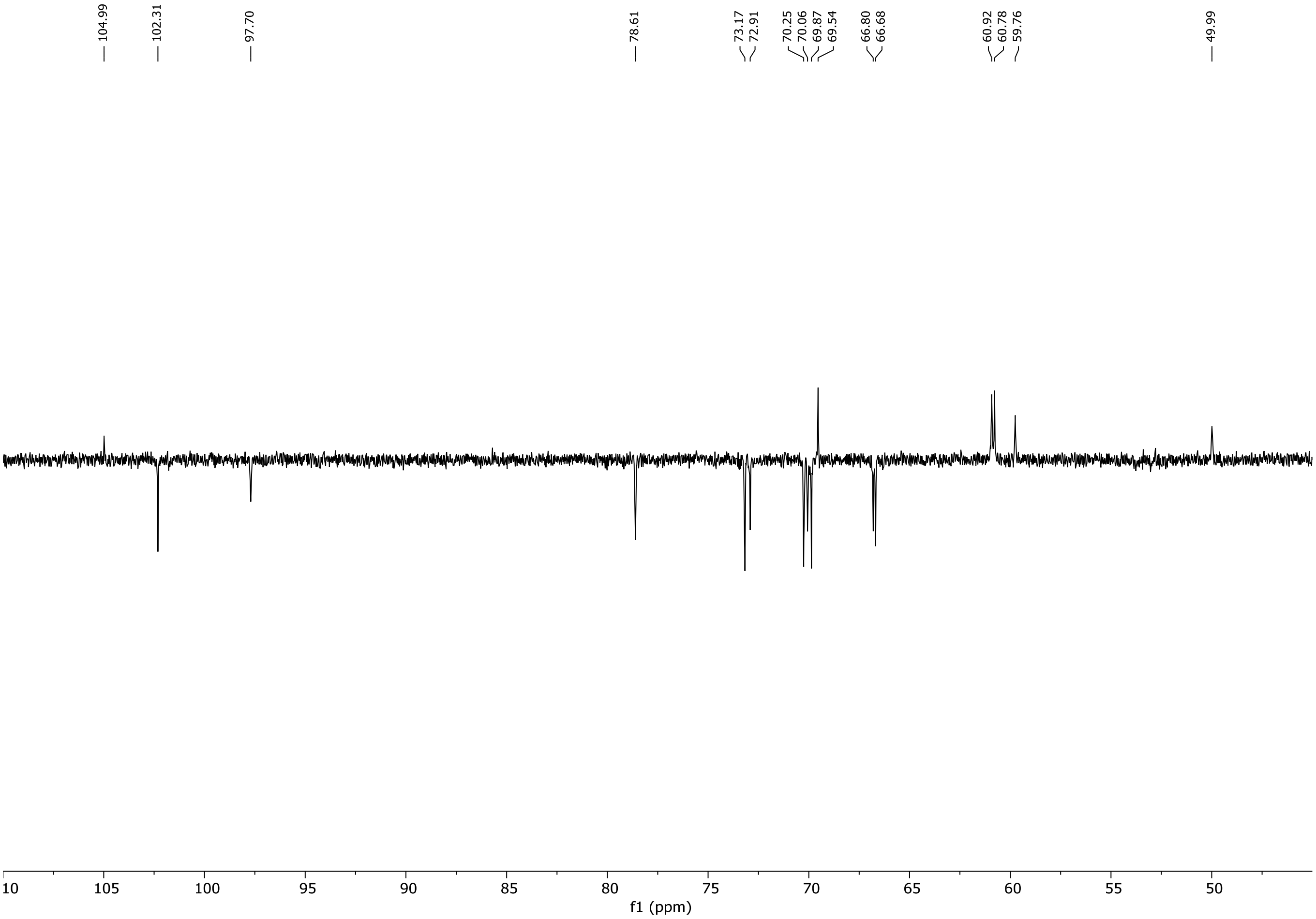

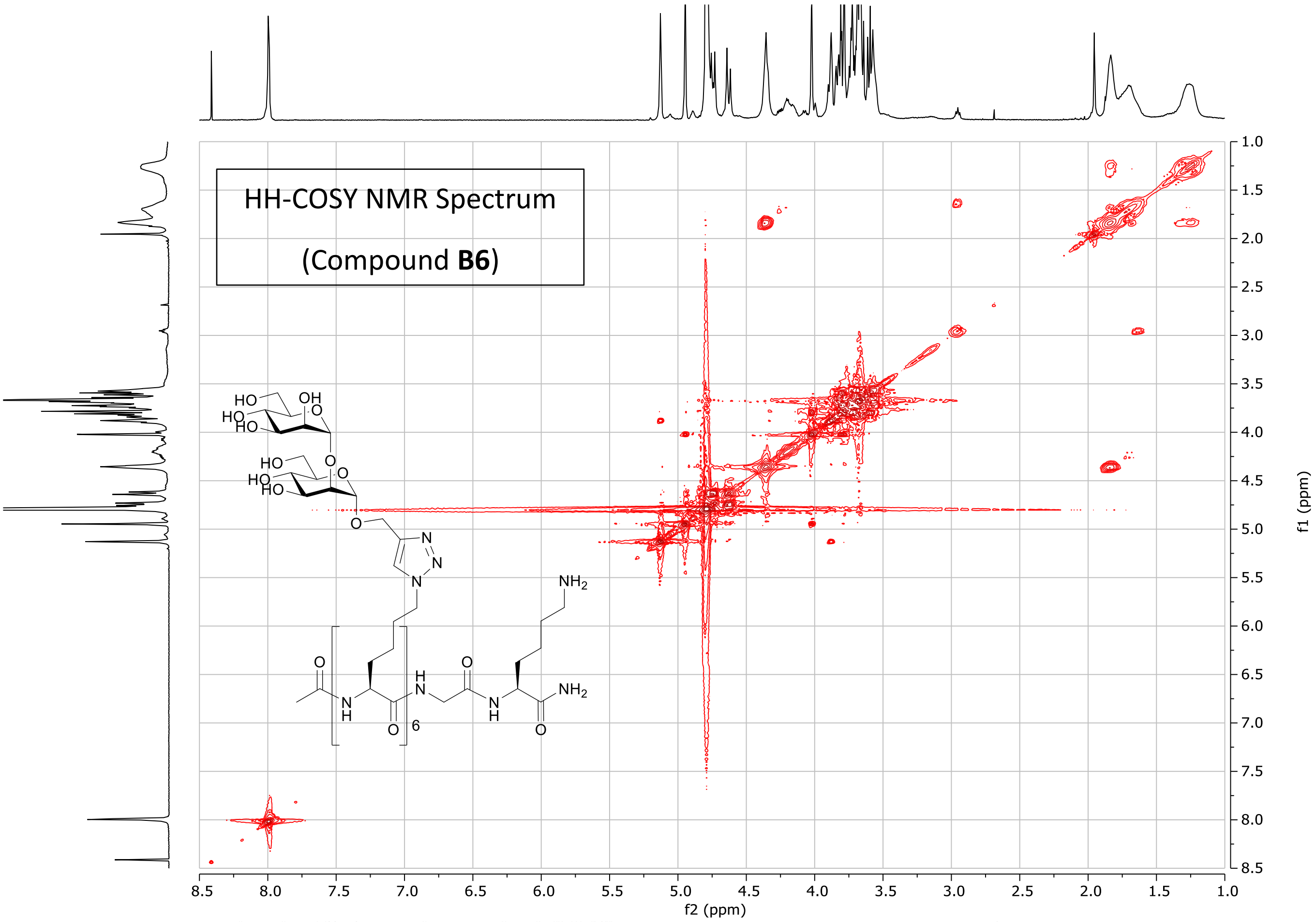
HH-COSY NMR Spectrum
(Compound **B6**)
f2 (ppm)
f1 (ppm)

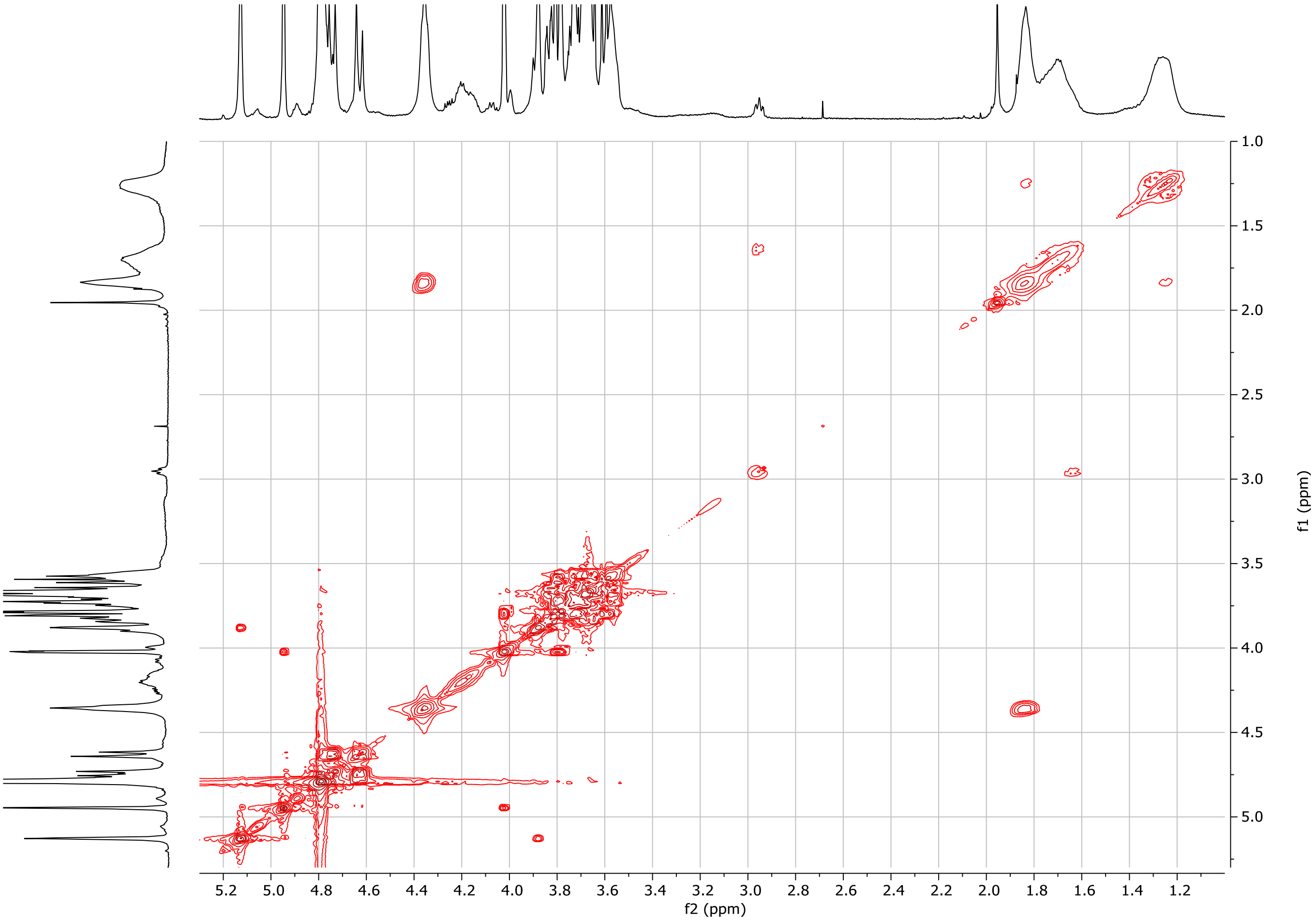
f2 (ppm)
f1 (ppm)

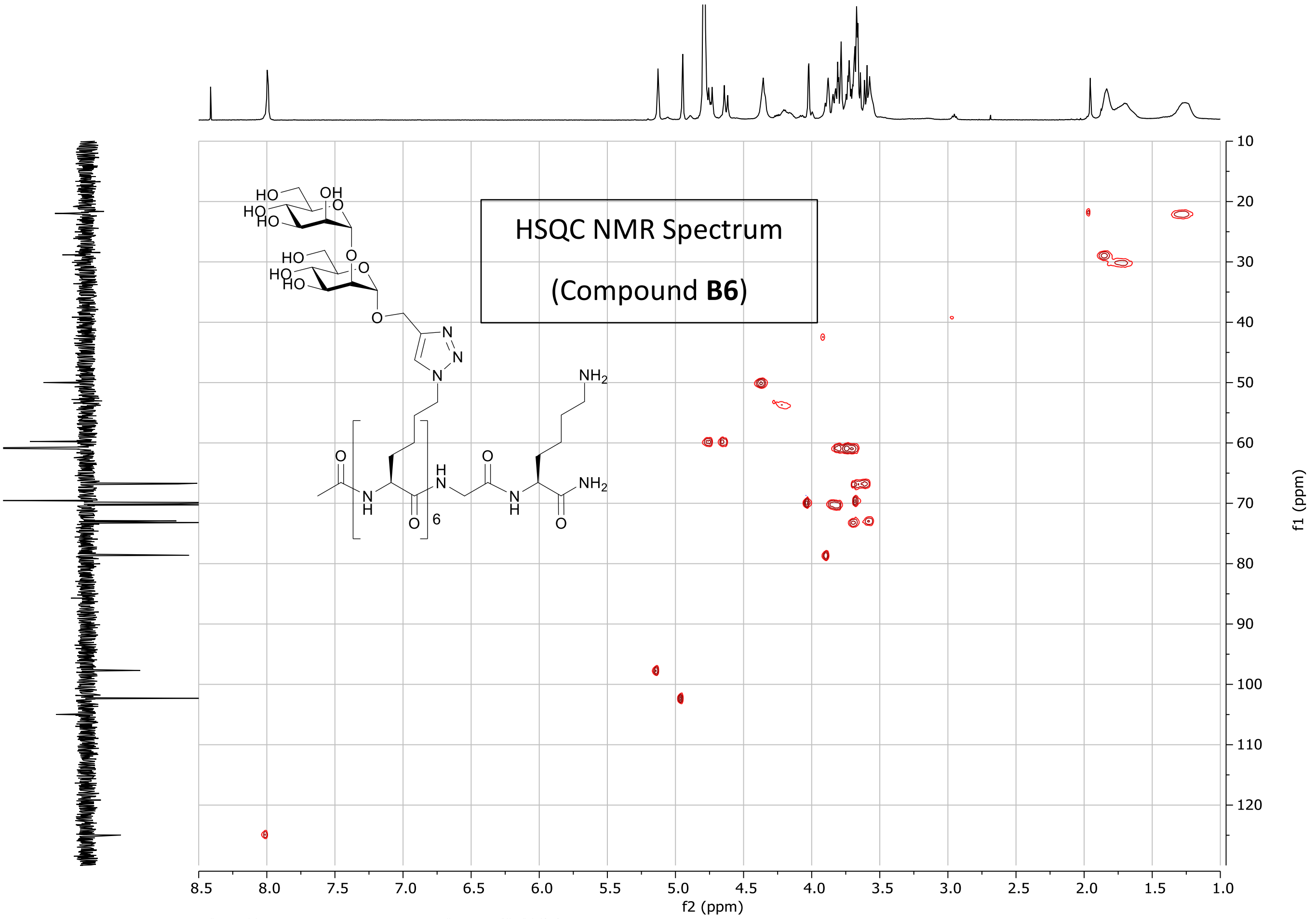
HSQC NMR Spectrum
(Compound **B6**)
f2 (ppm)
f1 (ppm)

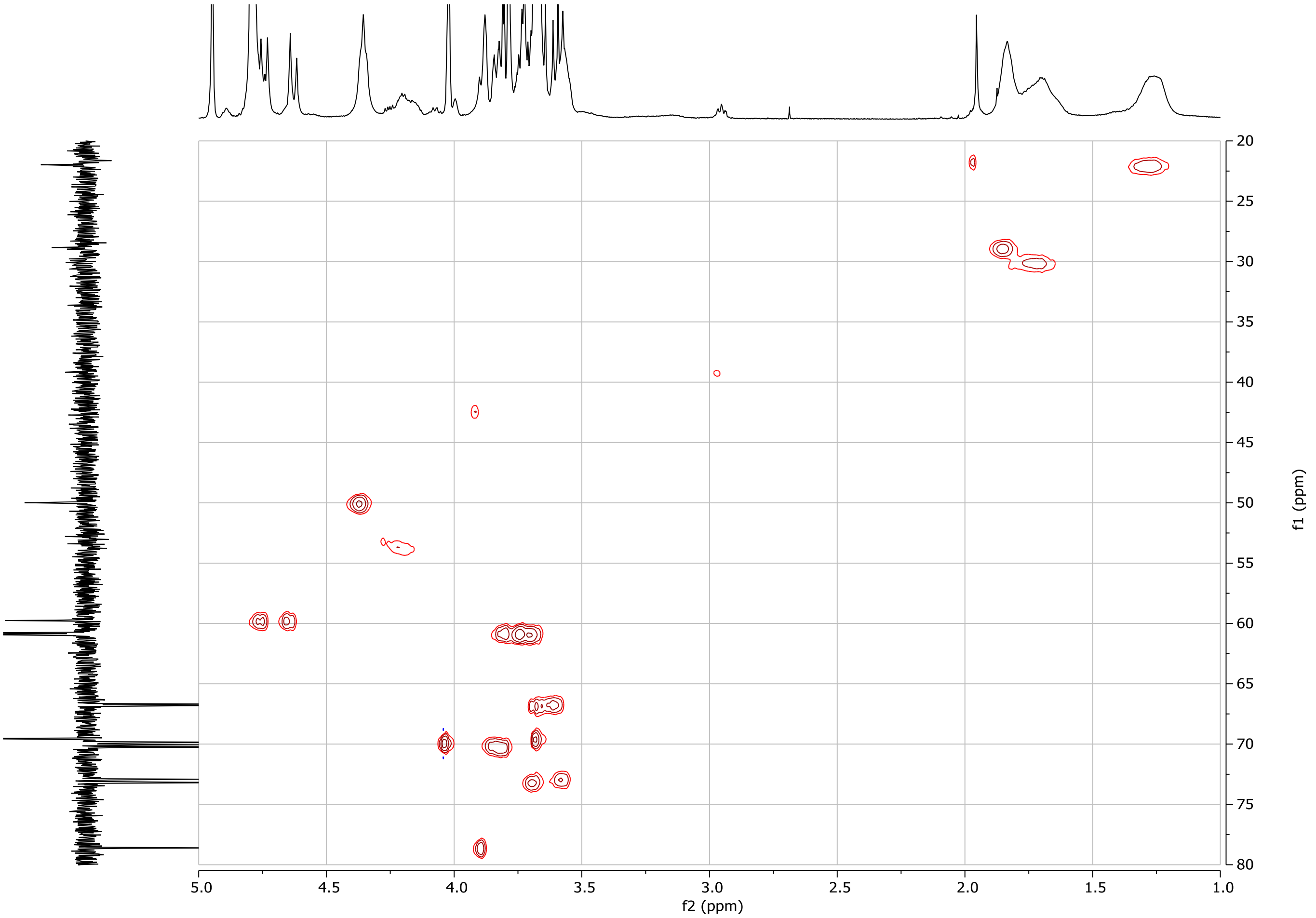
f2 (ppm)
f1 (ppm)

LC-MS Spectra; *(0 –> 50 % ACN, 13 min);* (Compound **B6**)

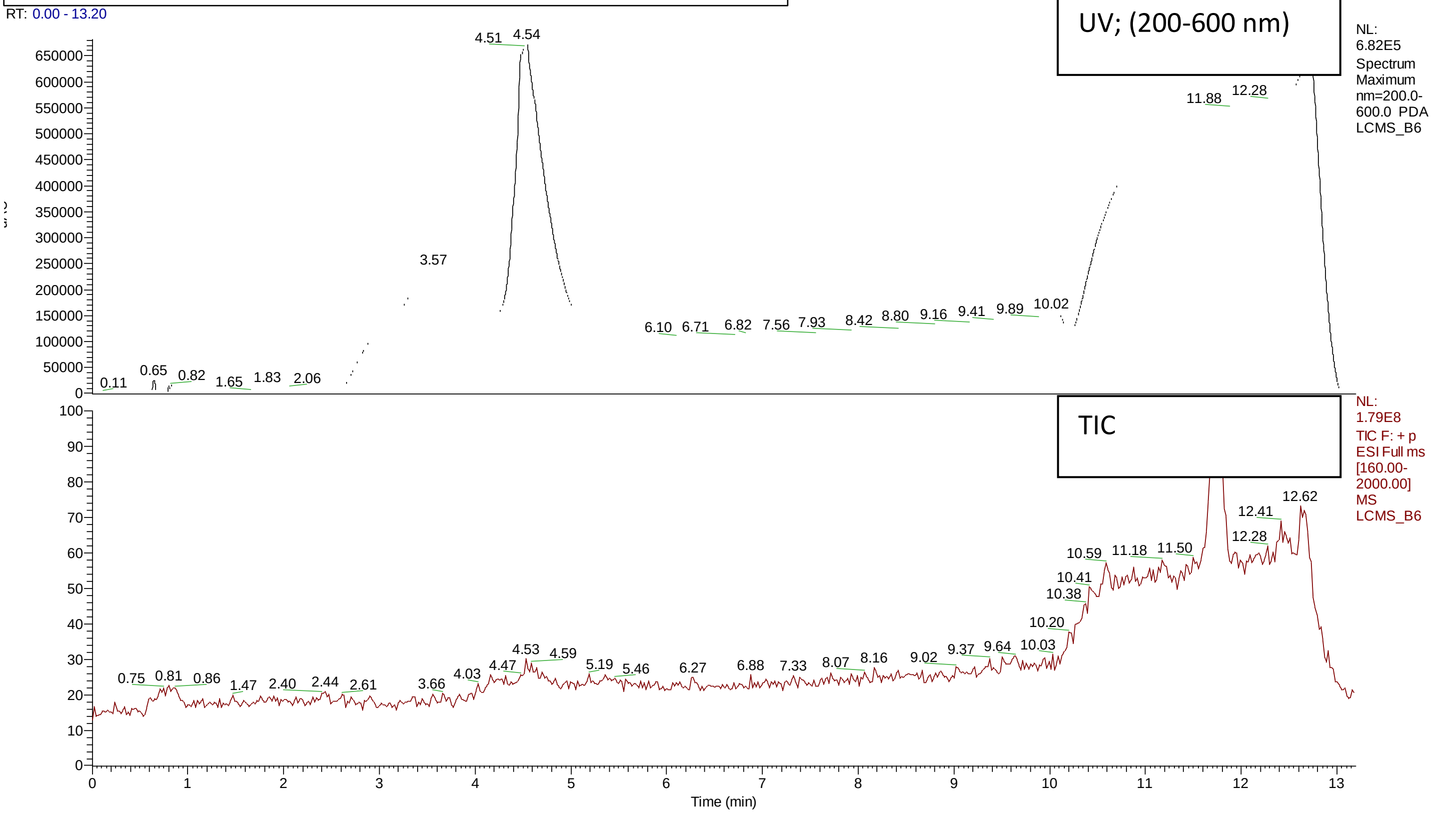

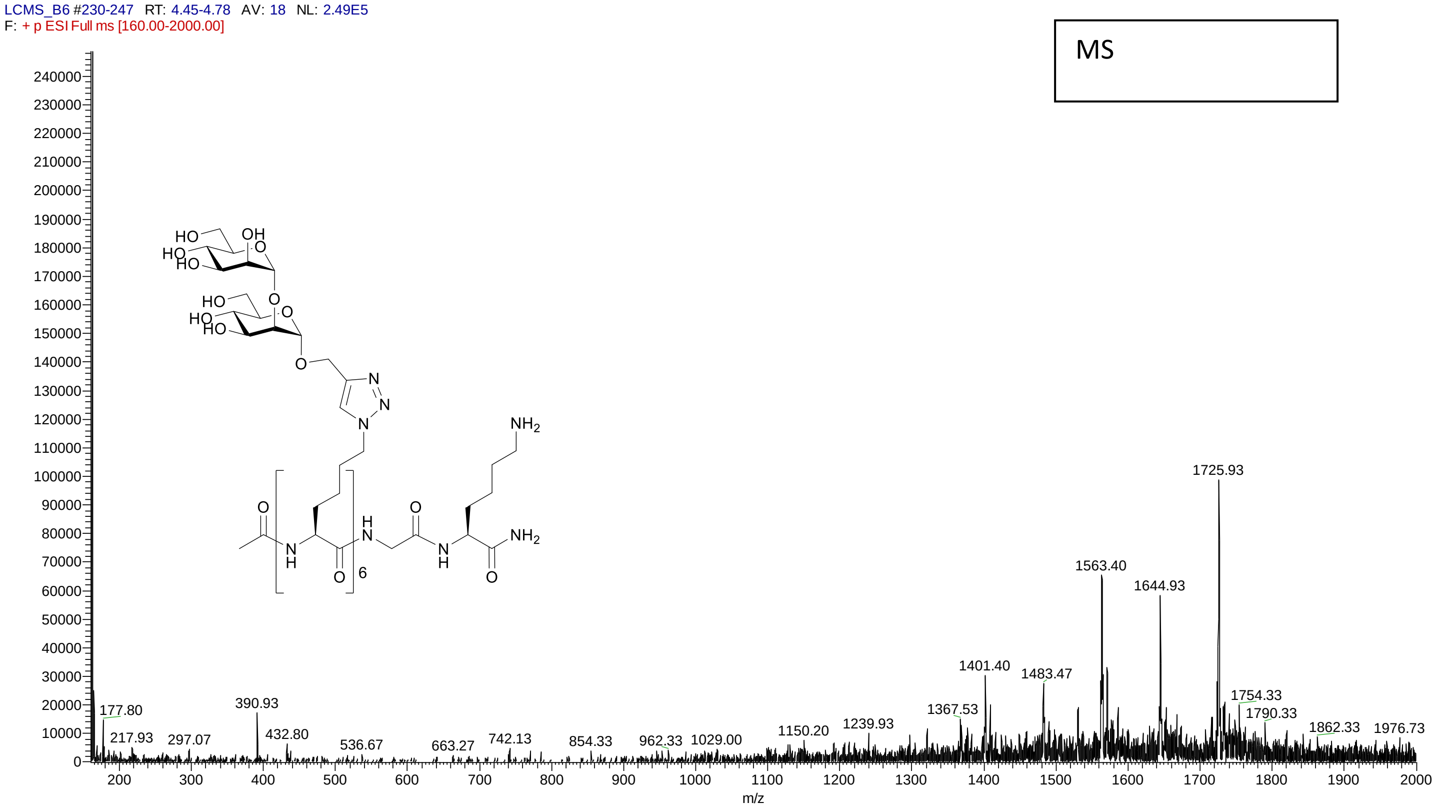

C1

^{1}H NMR Spectrum; (Compound **C1**)

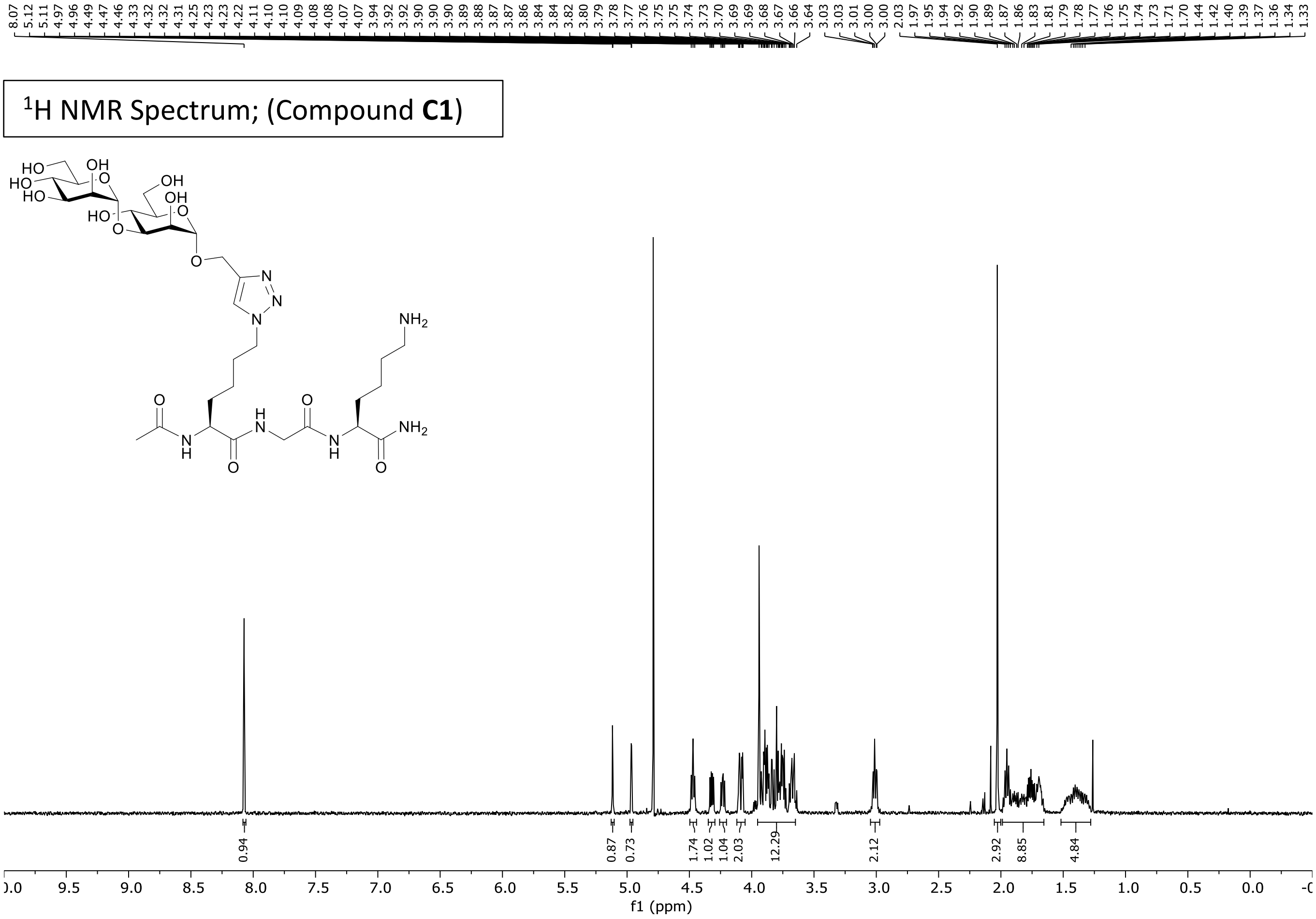

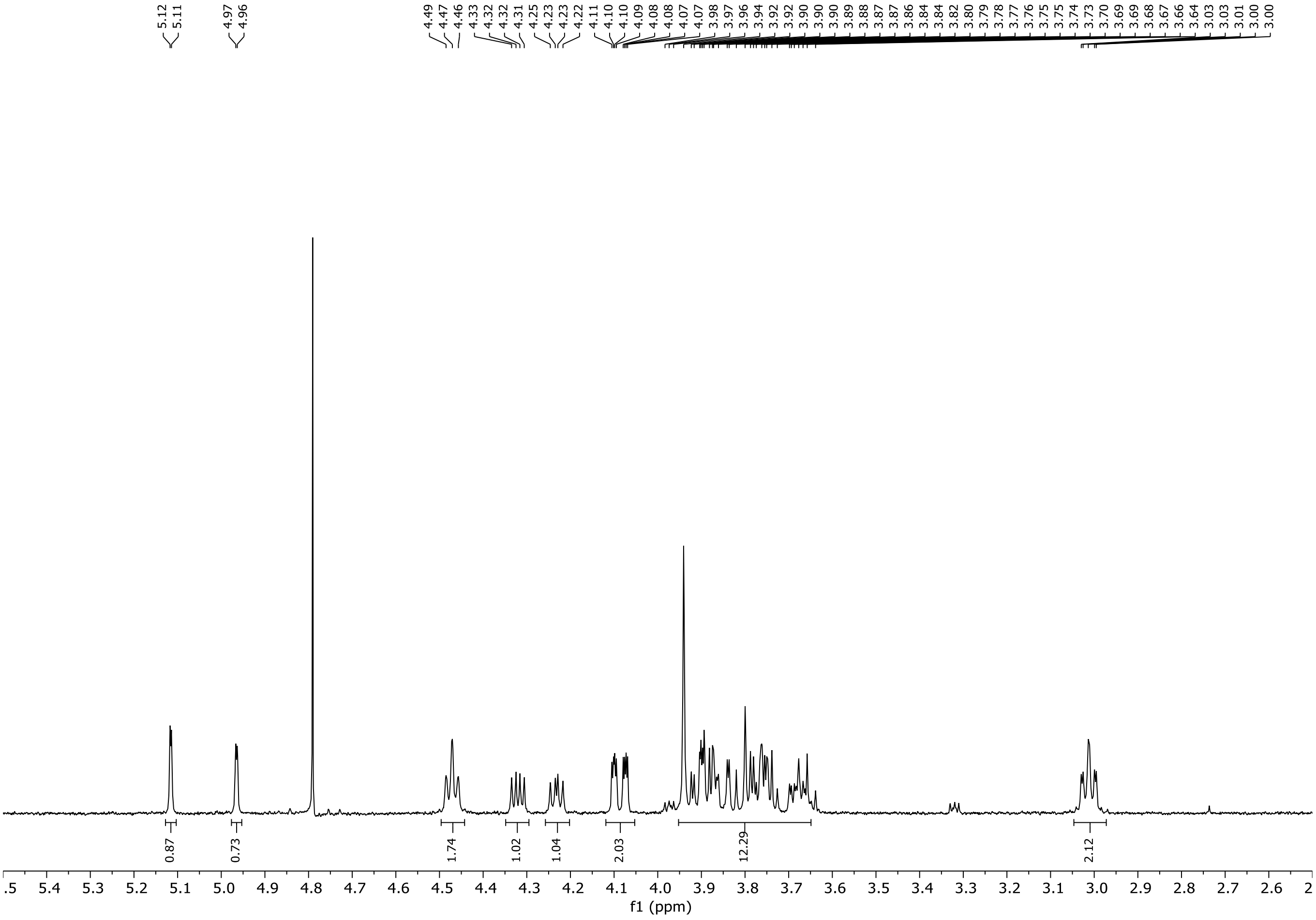

LC-MS Spectra; *(0 –> 50 % ACN, 13 min);* (Compound **C1**)

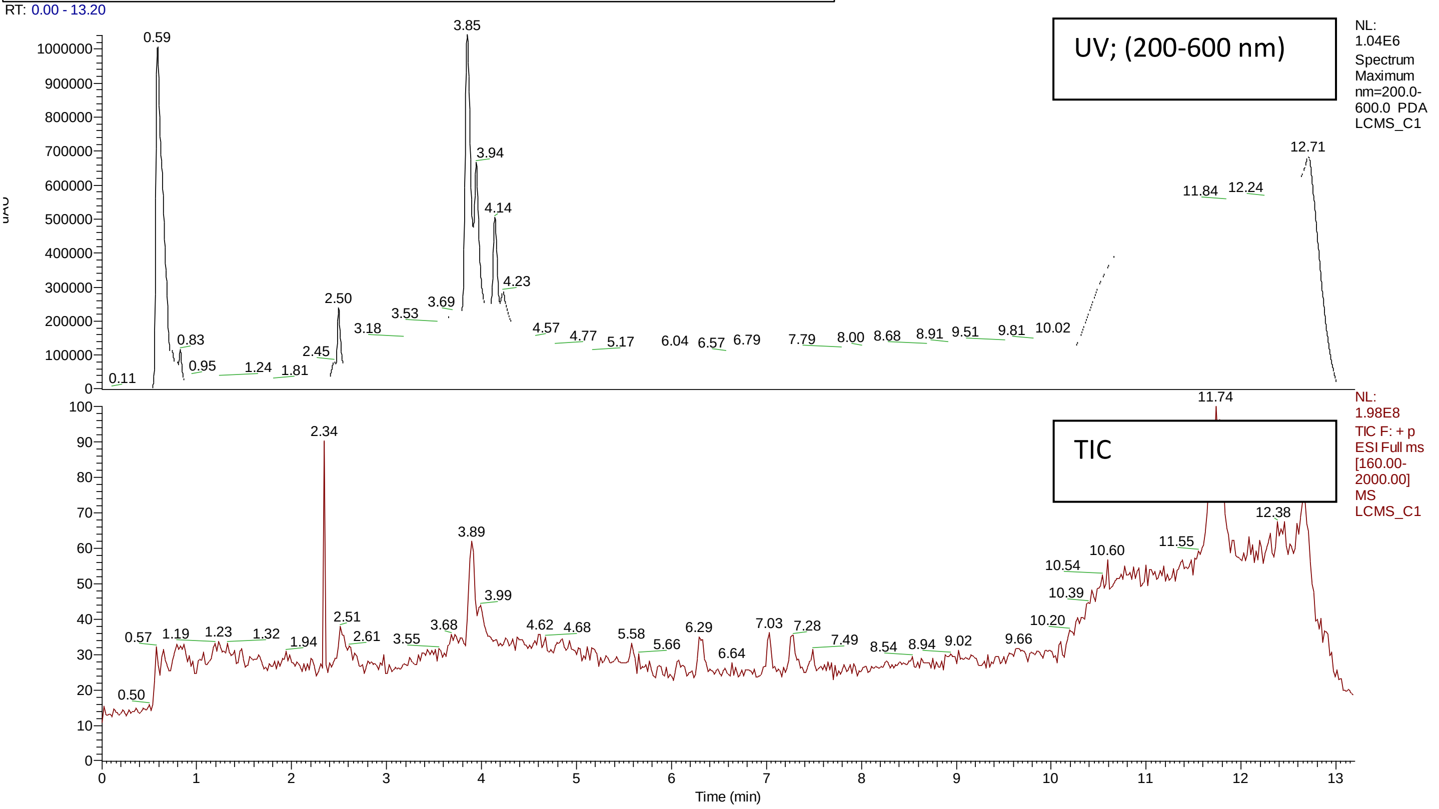

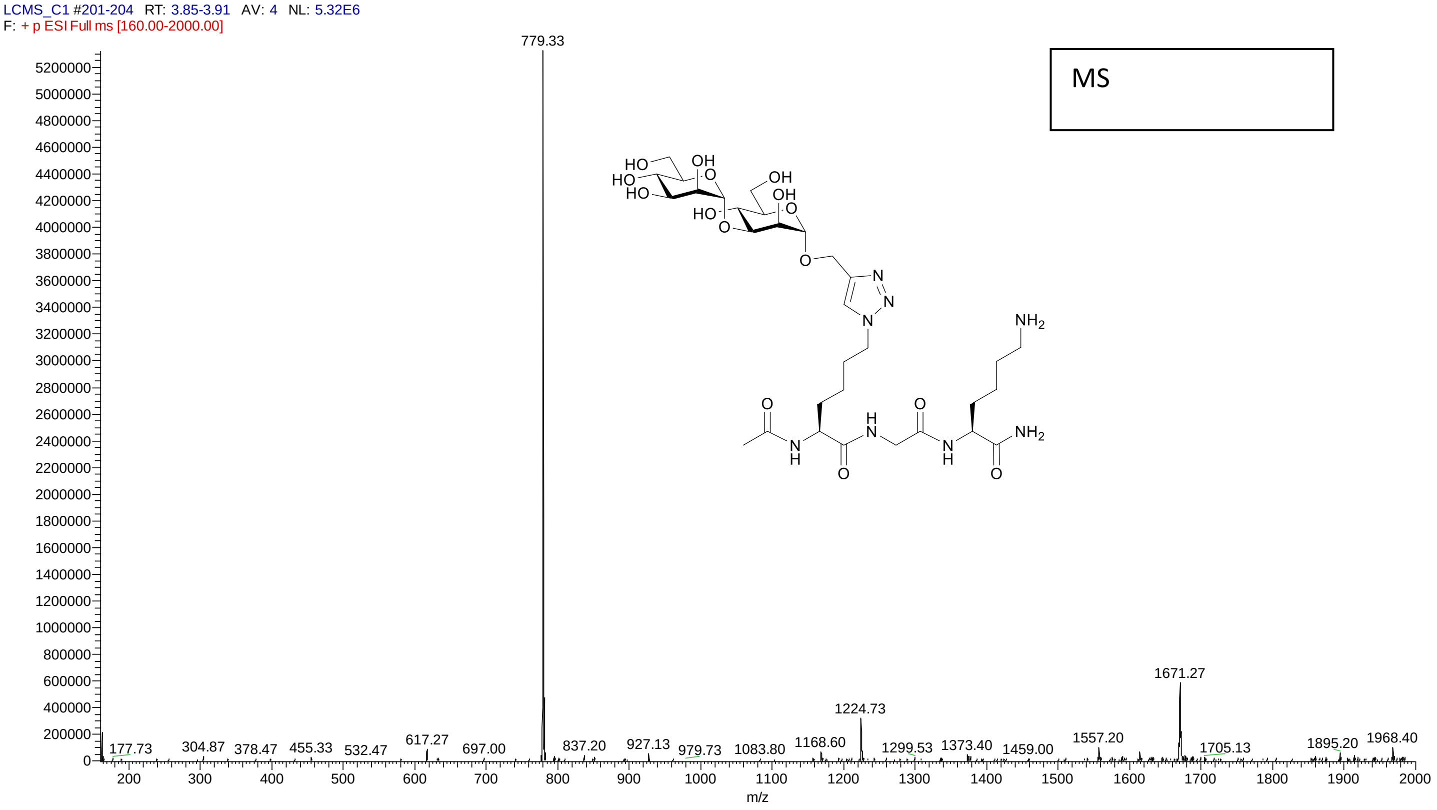

C2

LC-MS Spectra; *(0 –> 50 % ACN, 13 min);* (Compound **C2**)

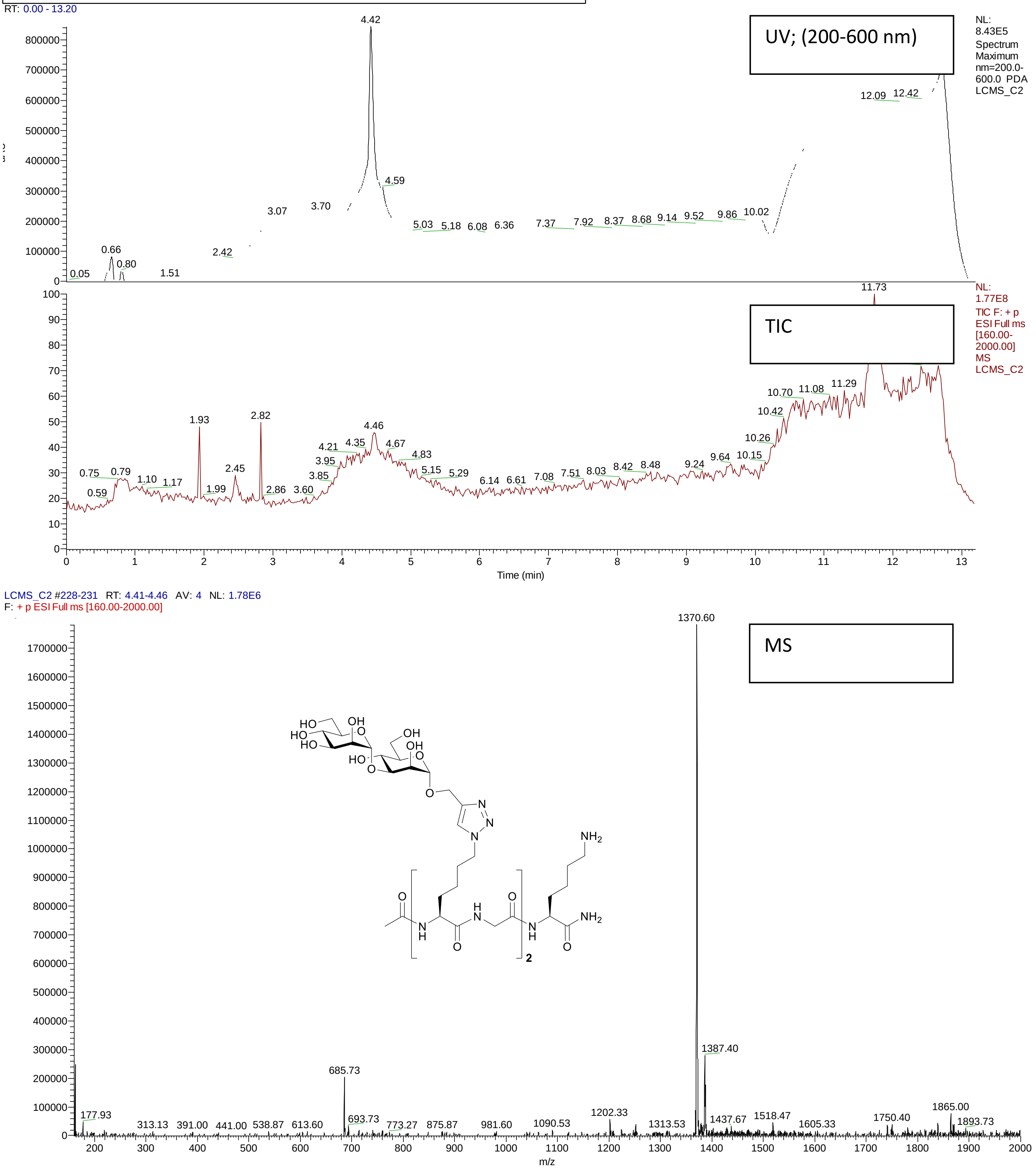

C3

^{1}H NMR Spectrum

(Compound **C3**)

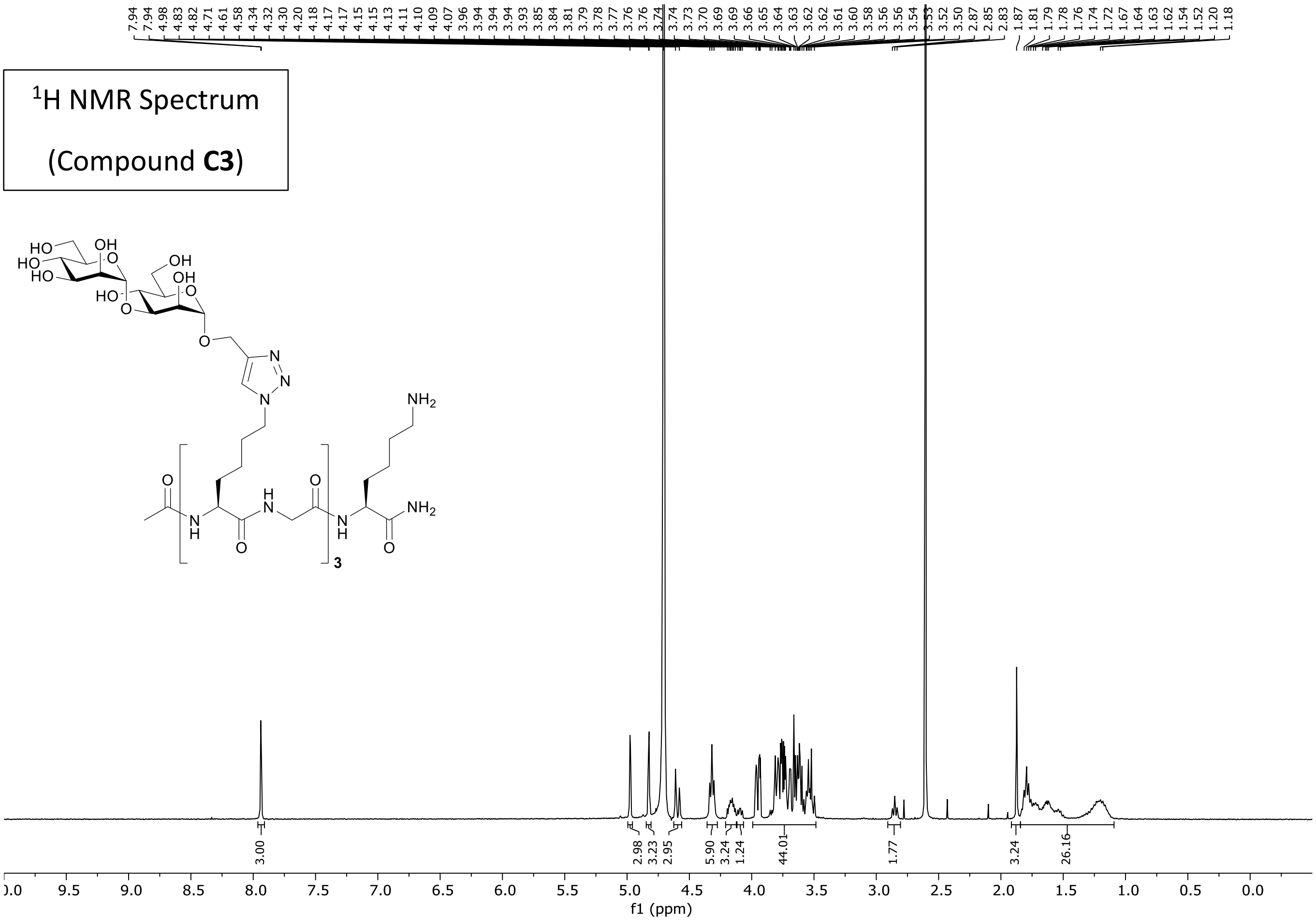

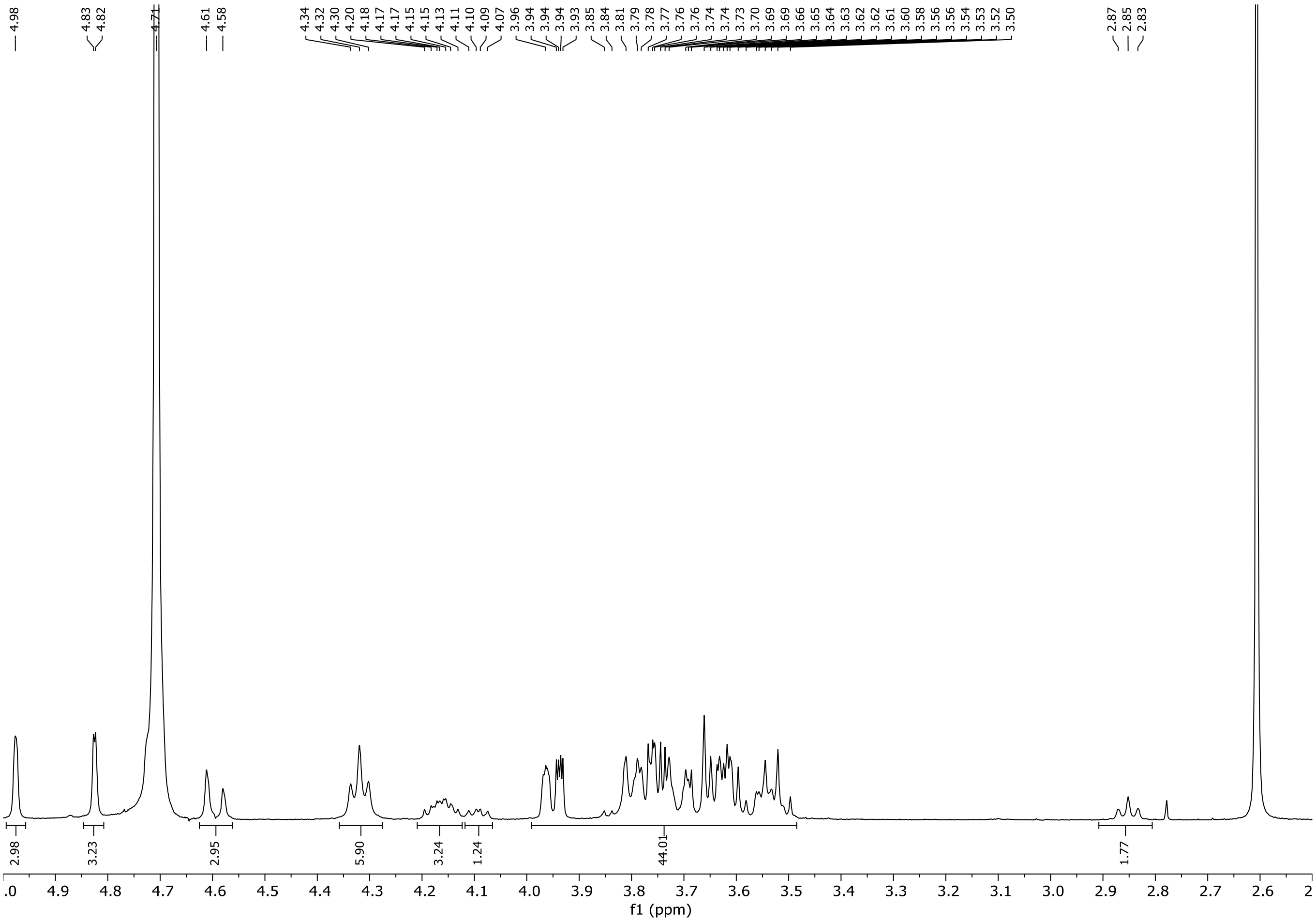

^{13}C APT NMR Spectrum

(Compound **C3**)

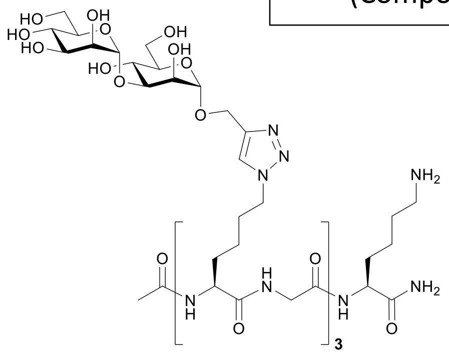

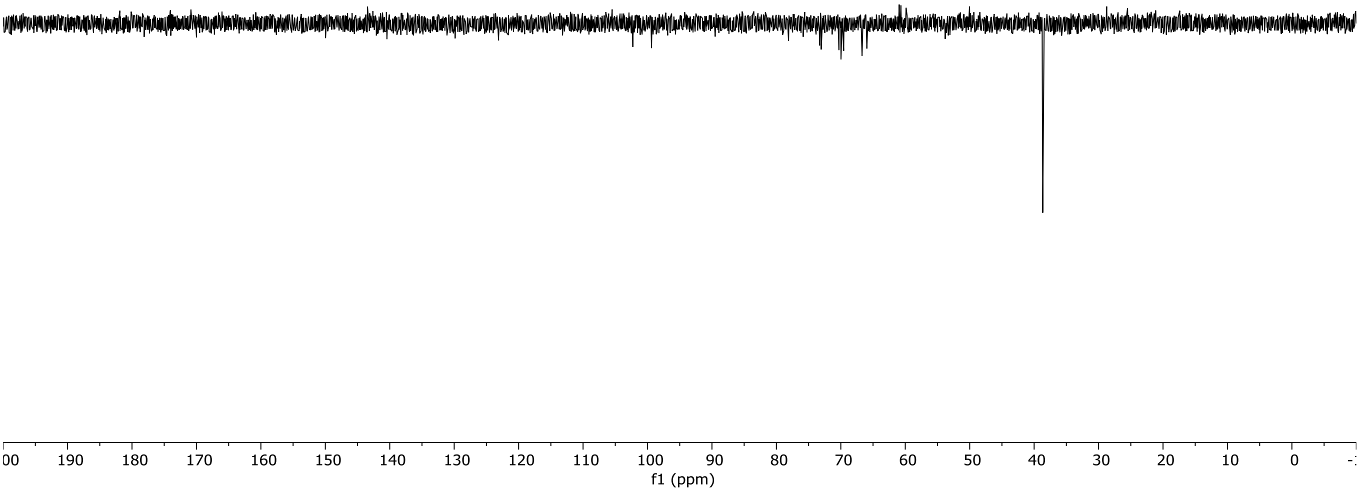

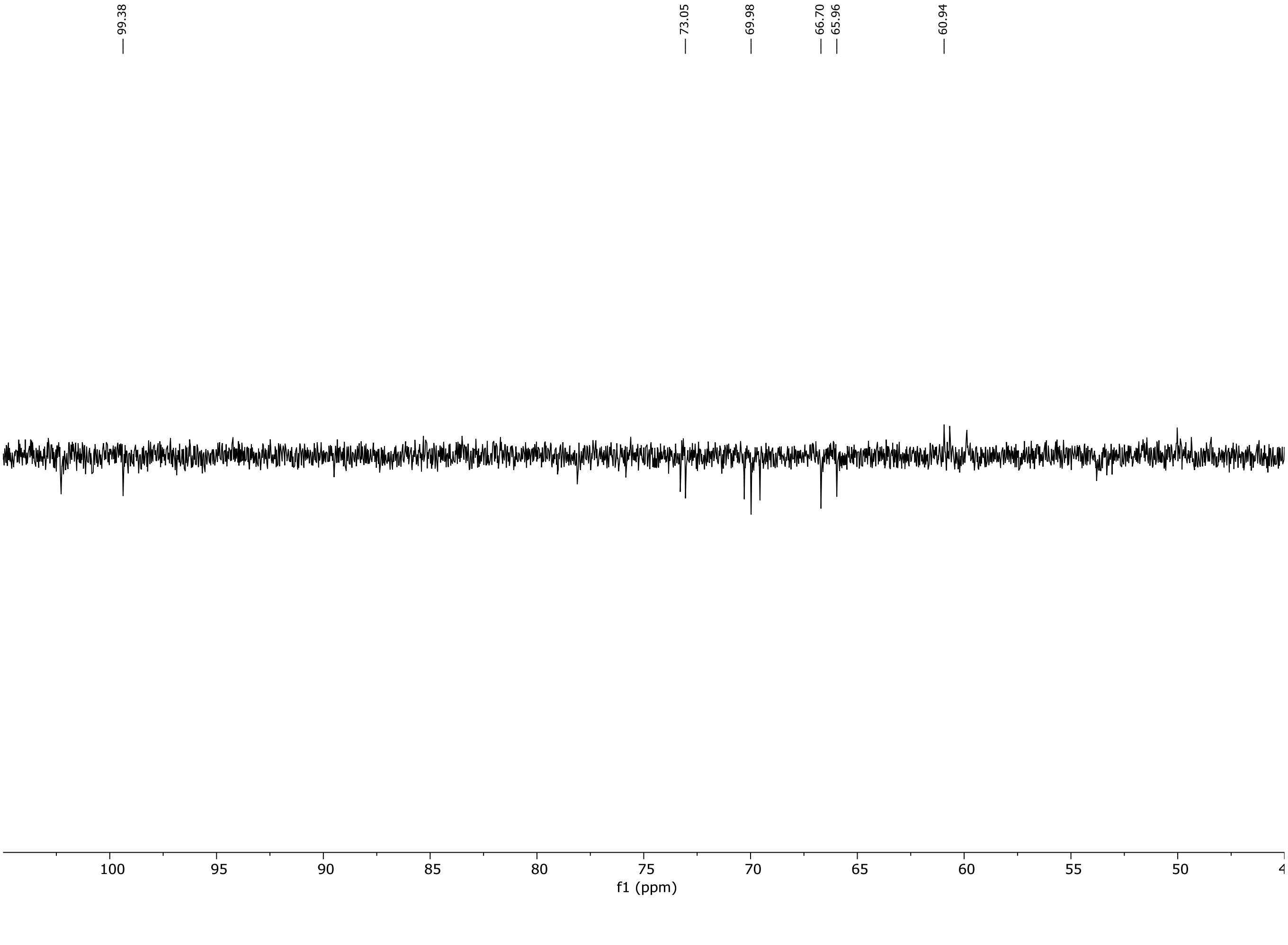

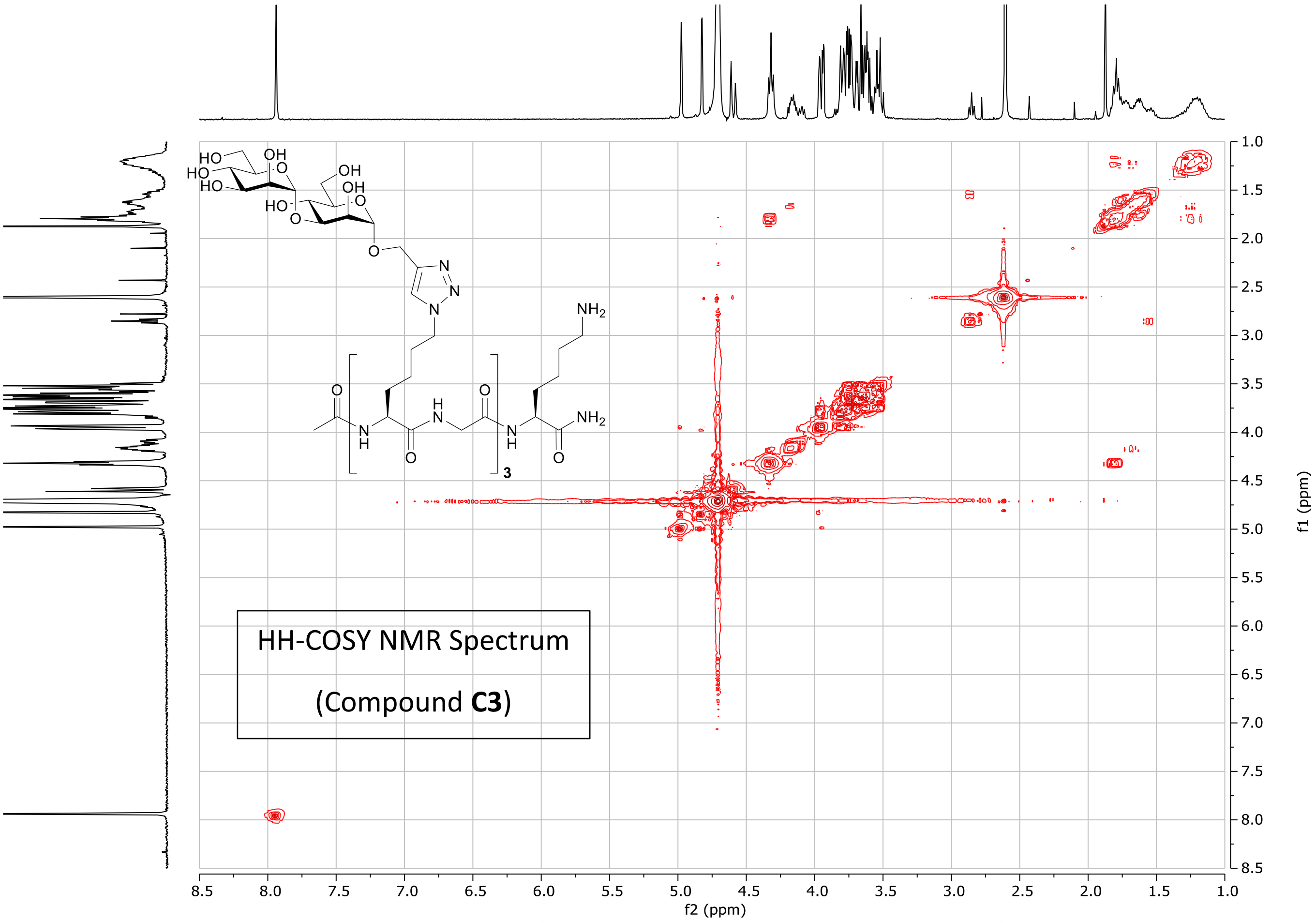
HH-COSY NMR Spectrum
(Compound **C3**)
f2 (ppm)
f1 (ppm)

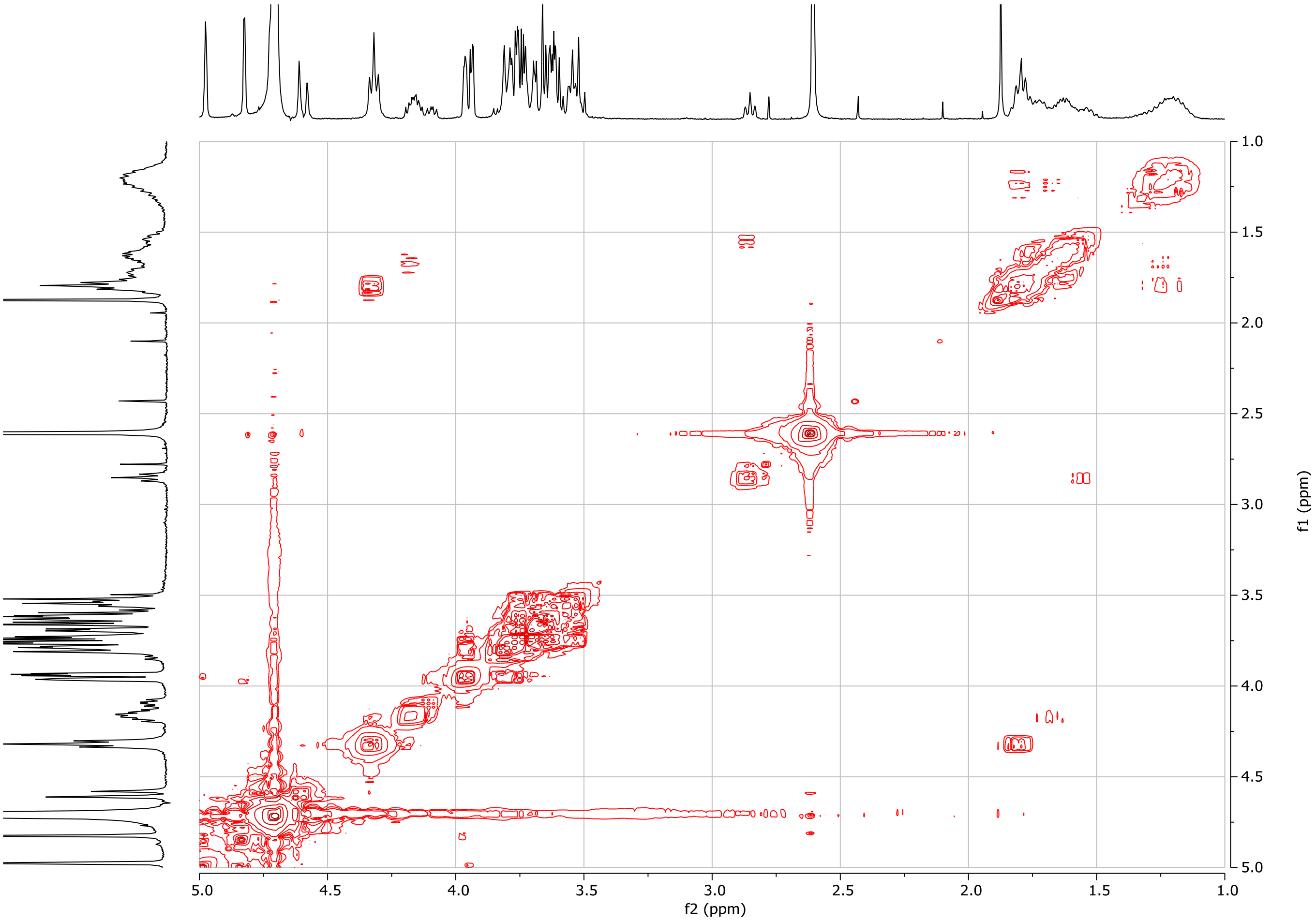
f2 (ppm)
f1 (ppm)

LC-MS Spectra; *(0 –> 50 % ACN, 13 min);* (Compound **C3**)

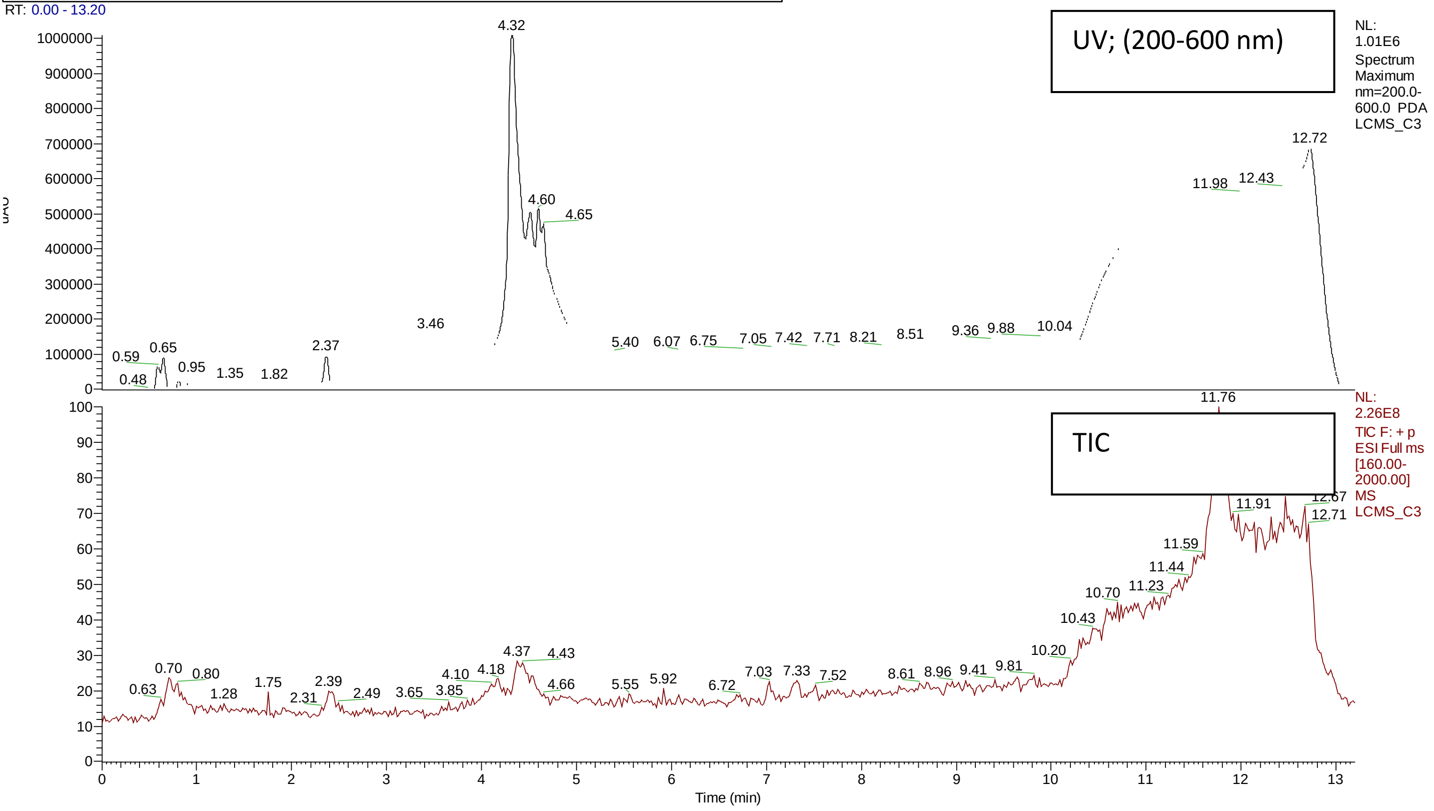

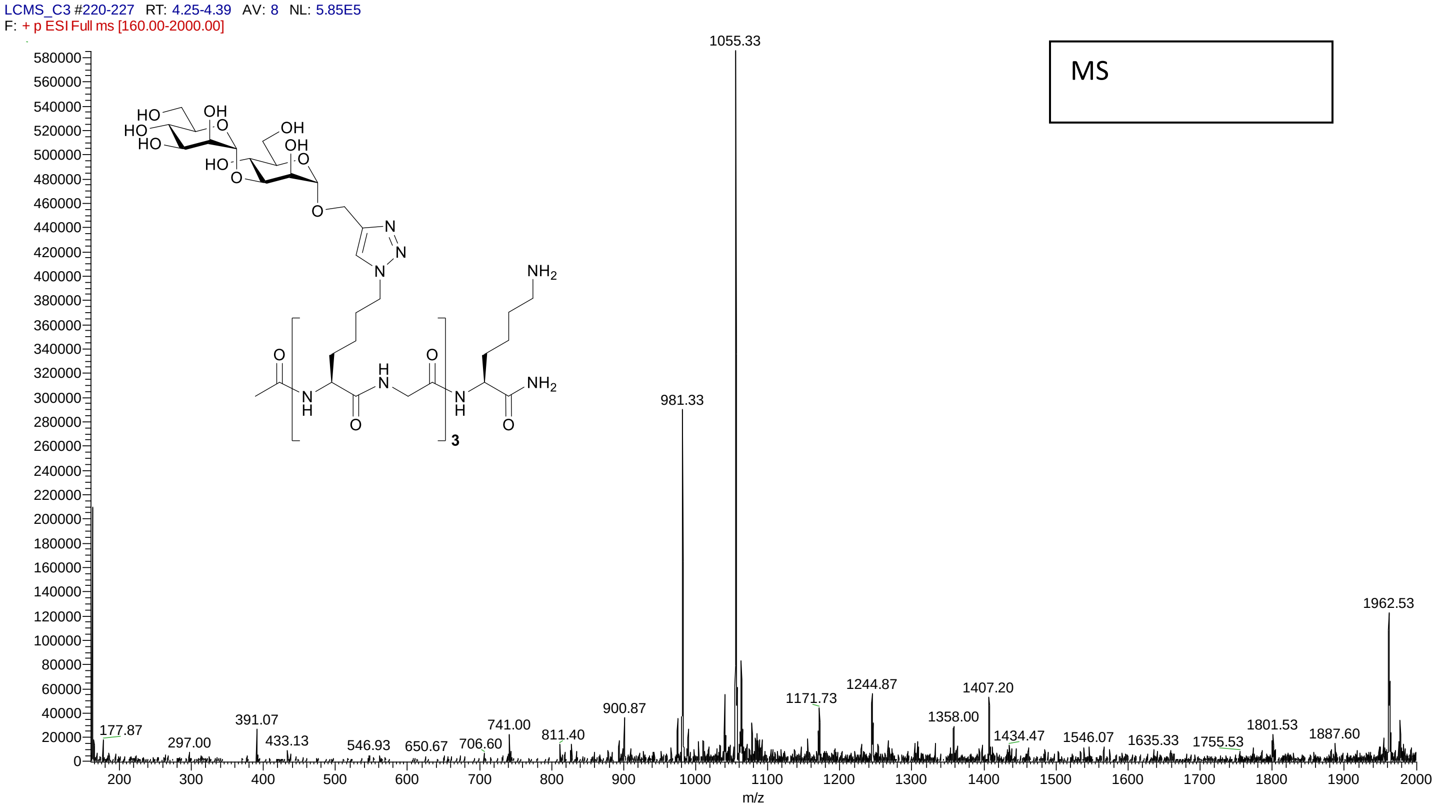

C6

^{1}H NMR Spectrum

(Compound **C6**)

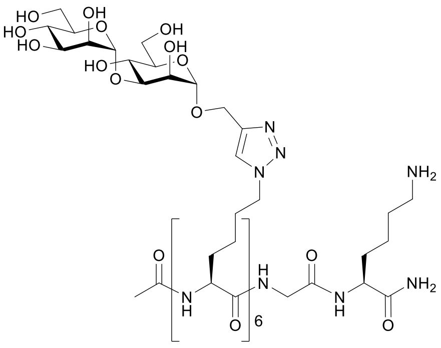

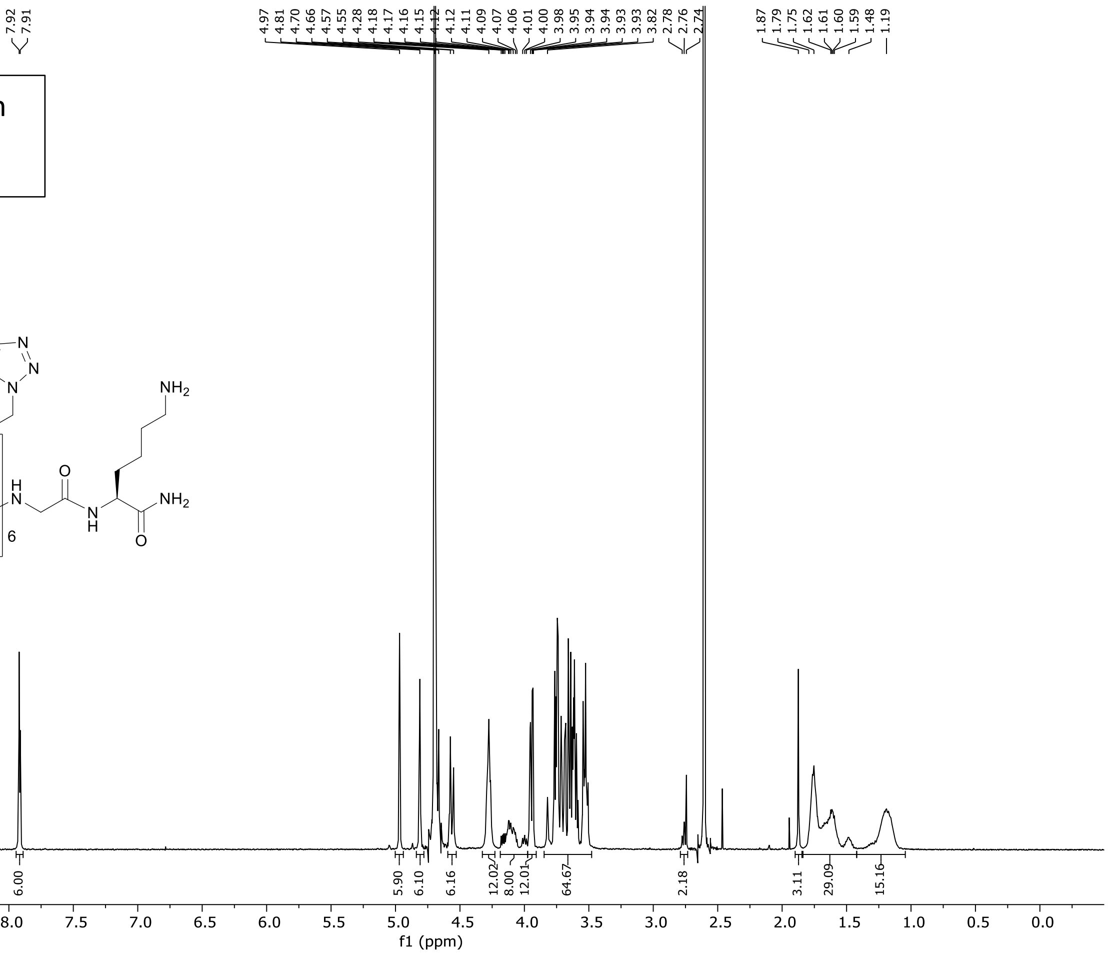

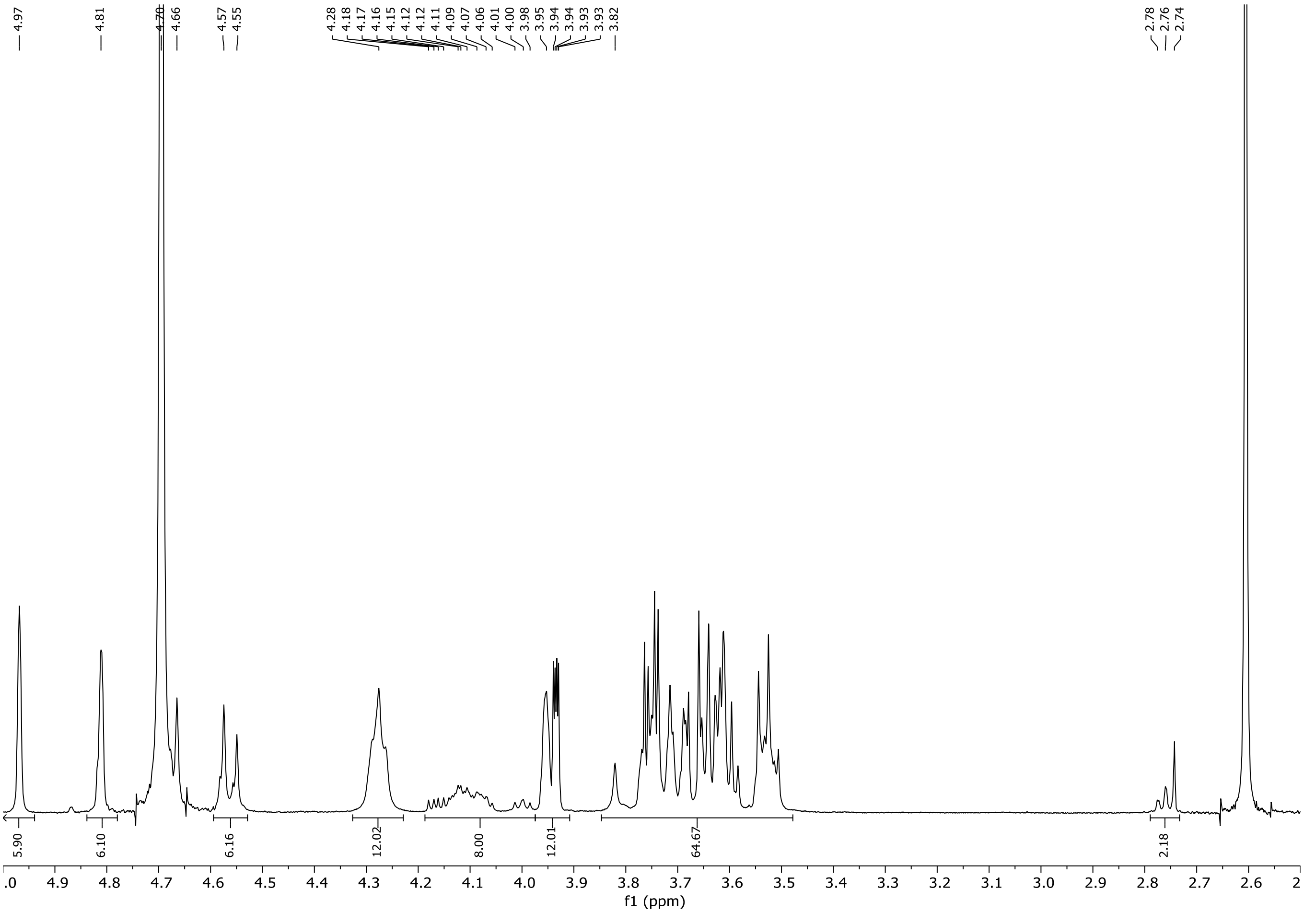

^{13}C APT NMR Spectrum

(Compound **C6**)

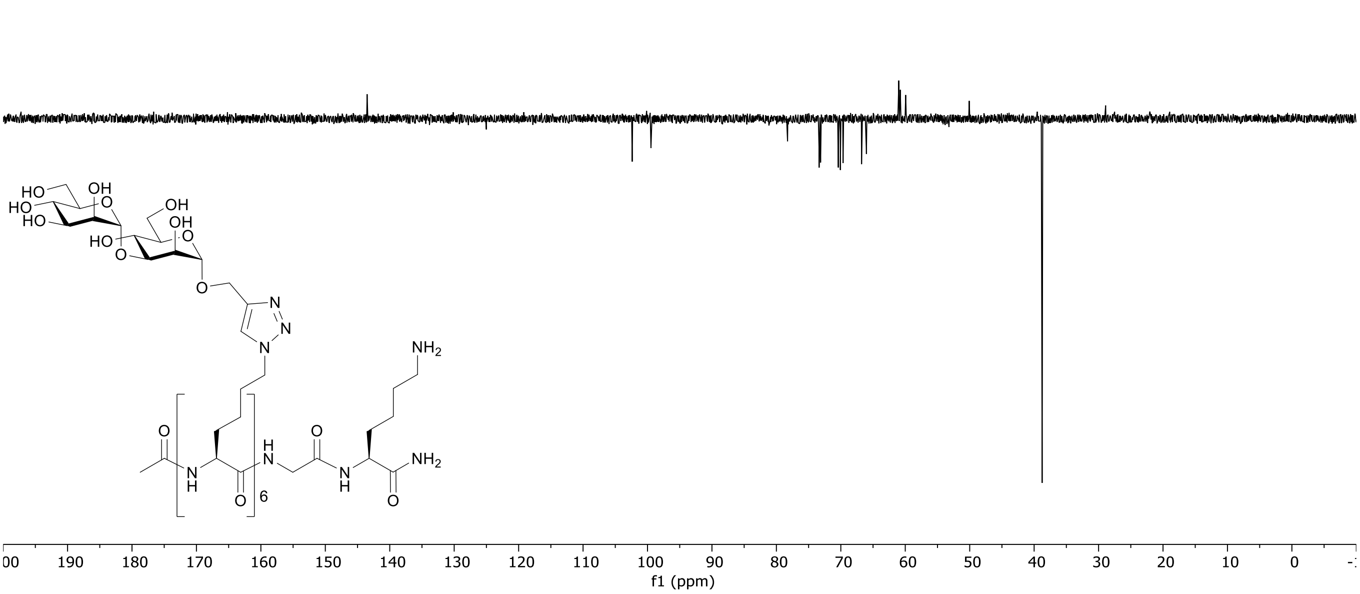

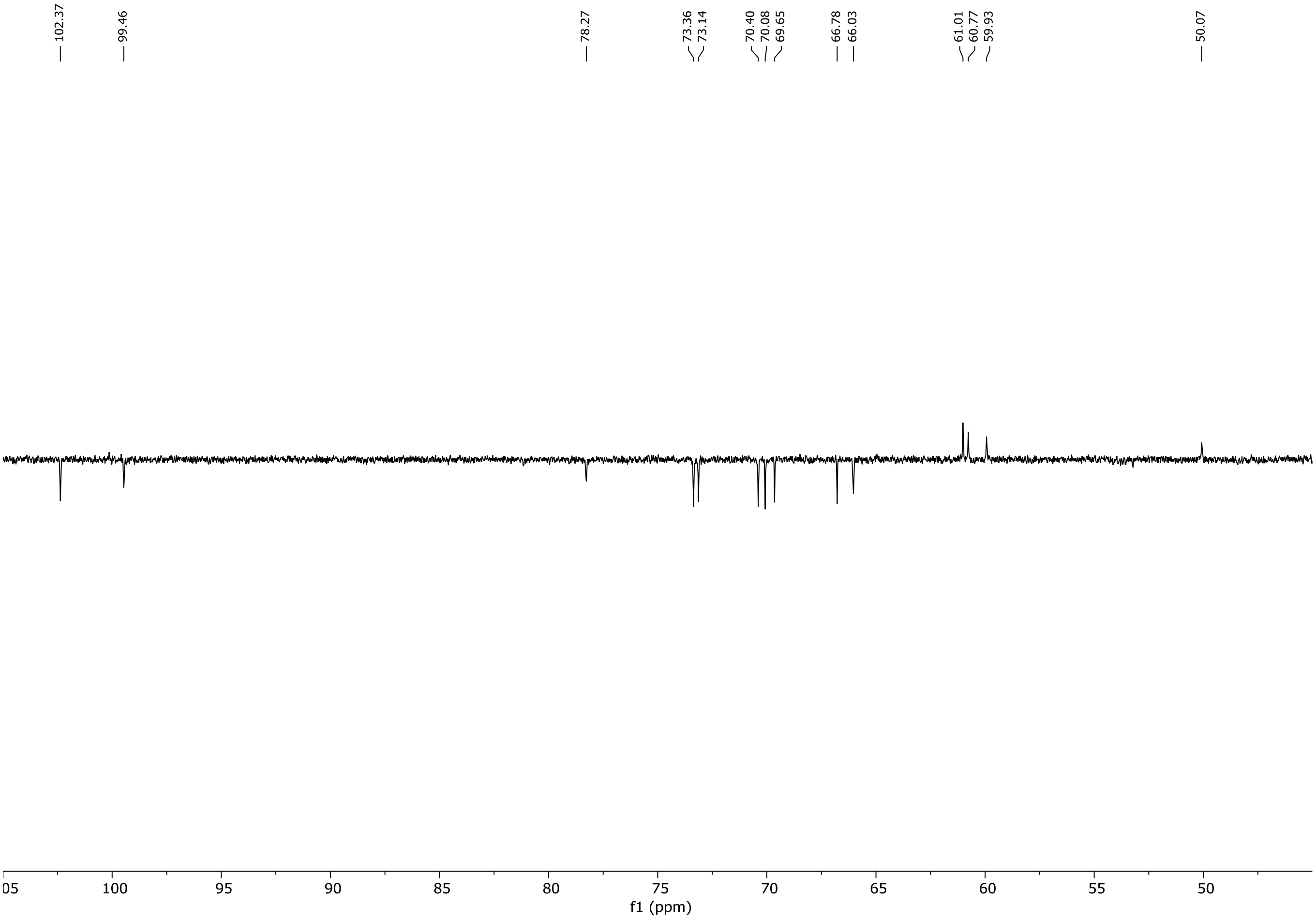

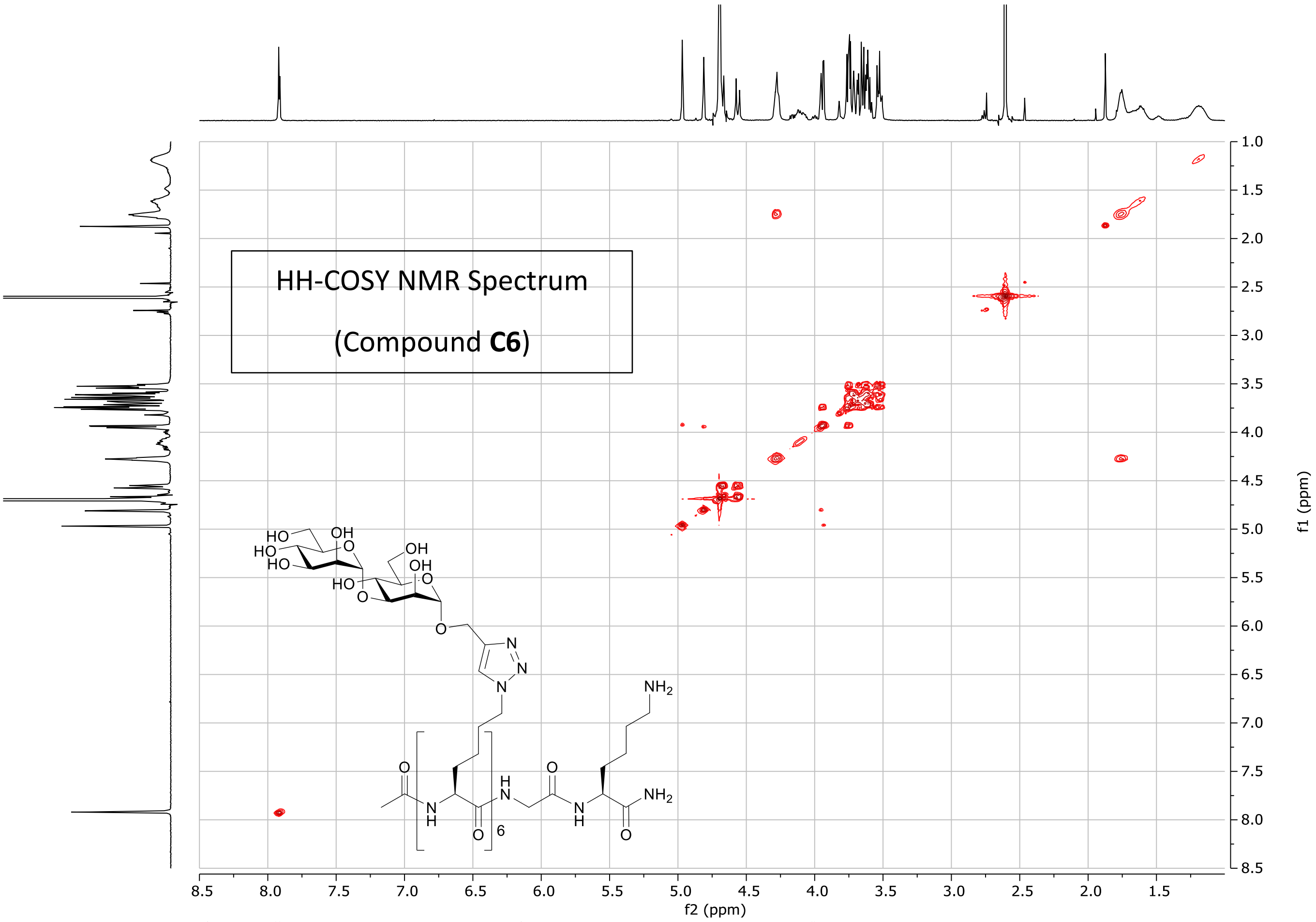
HH-COSY NMR Spectrum
(Compound **C6**)
f2 (ppm)
f1 (ppm)

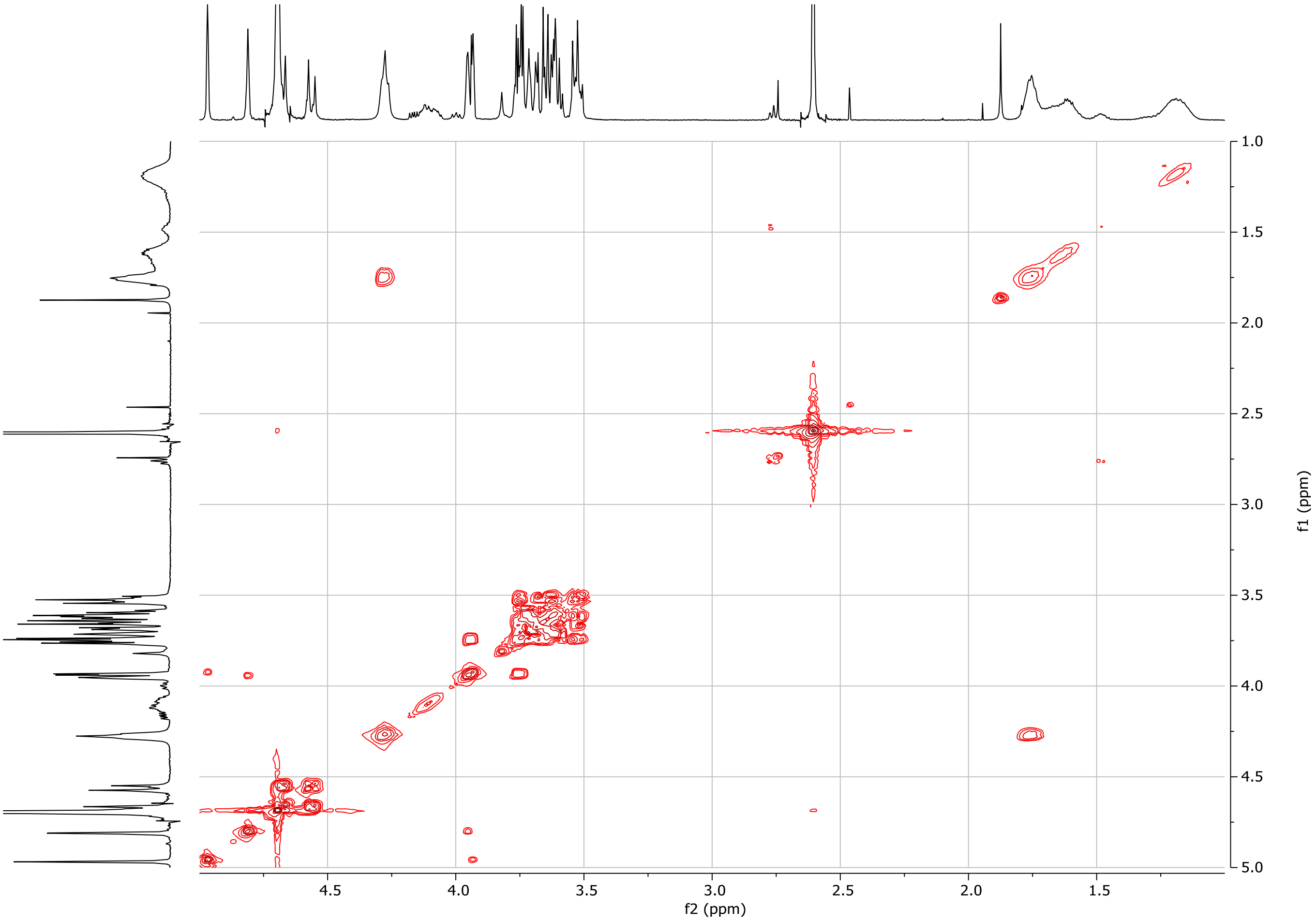
f2 (ppm)
f1 (ppm)

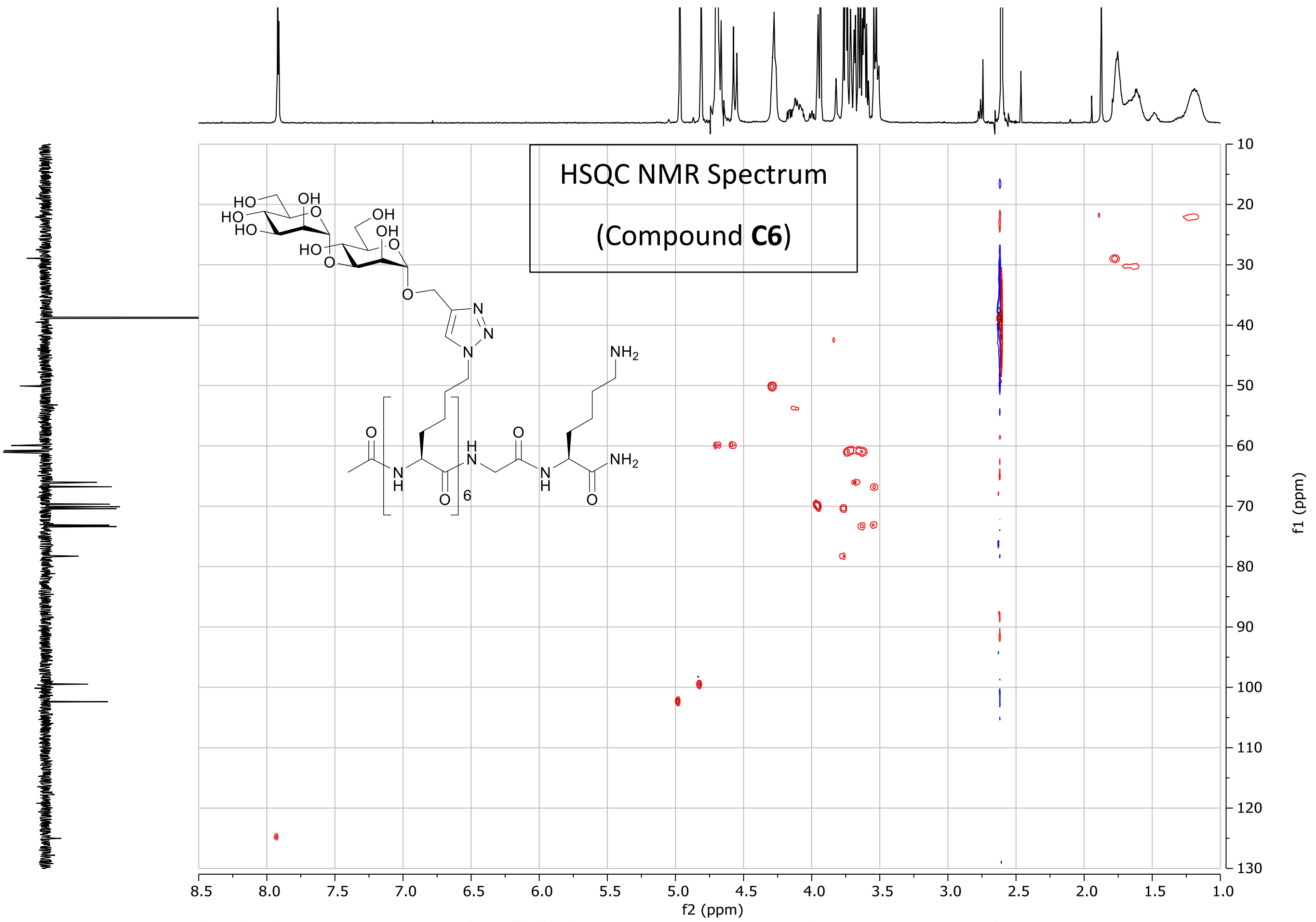
HSQC NMR Spectrum
(Compound **C6**)
f1 (ppm)
f2 (ppm)

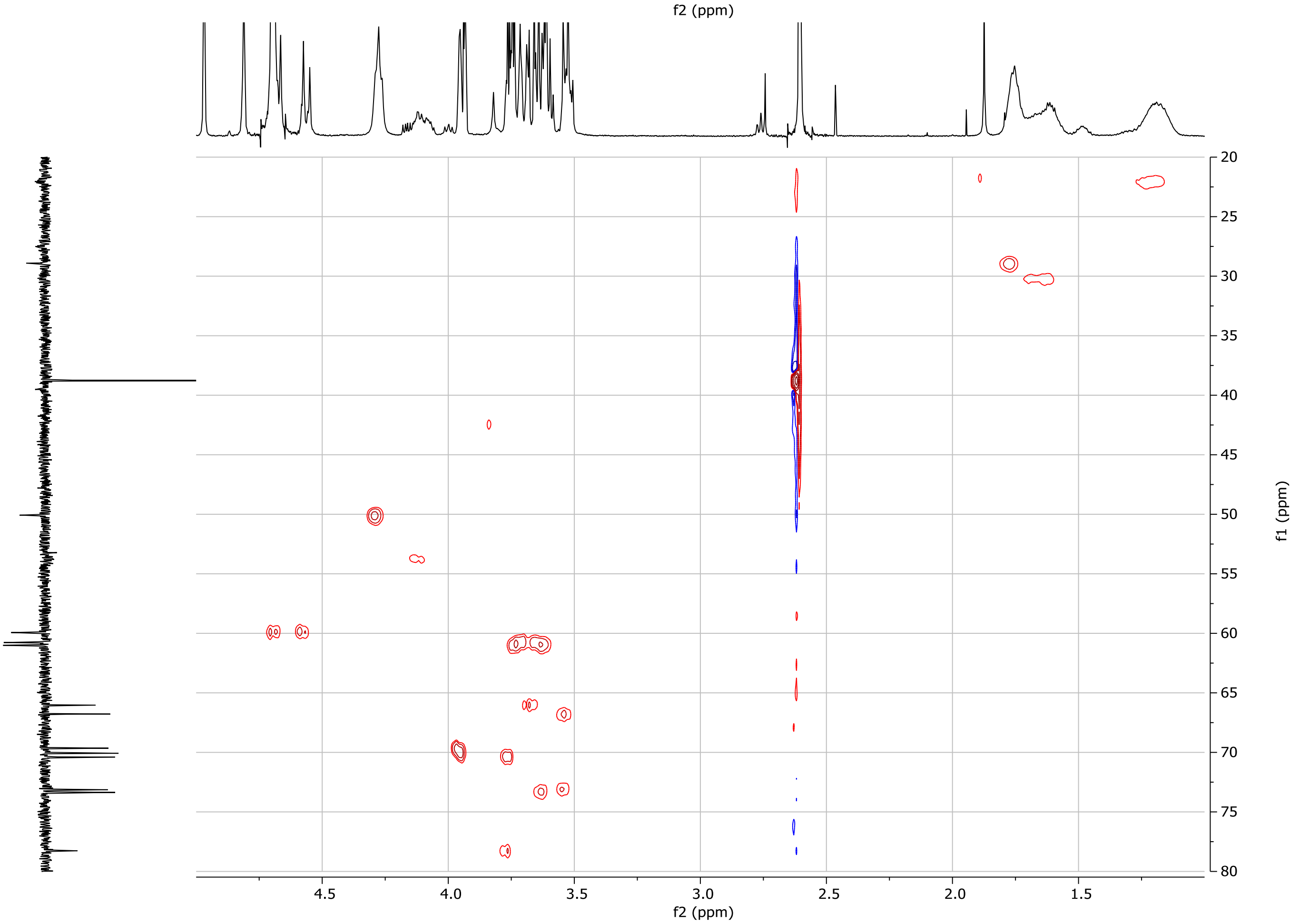
f1 (ppm)
f2 (ppm)

LC-MS Spectra; *(0 –> 50 % ACN, 13 min);* (Compound **C6**)

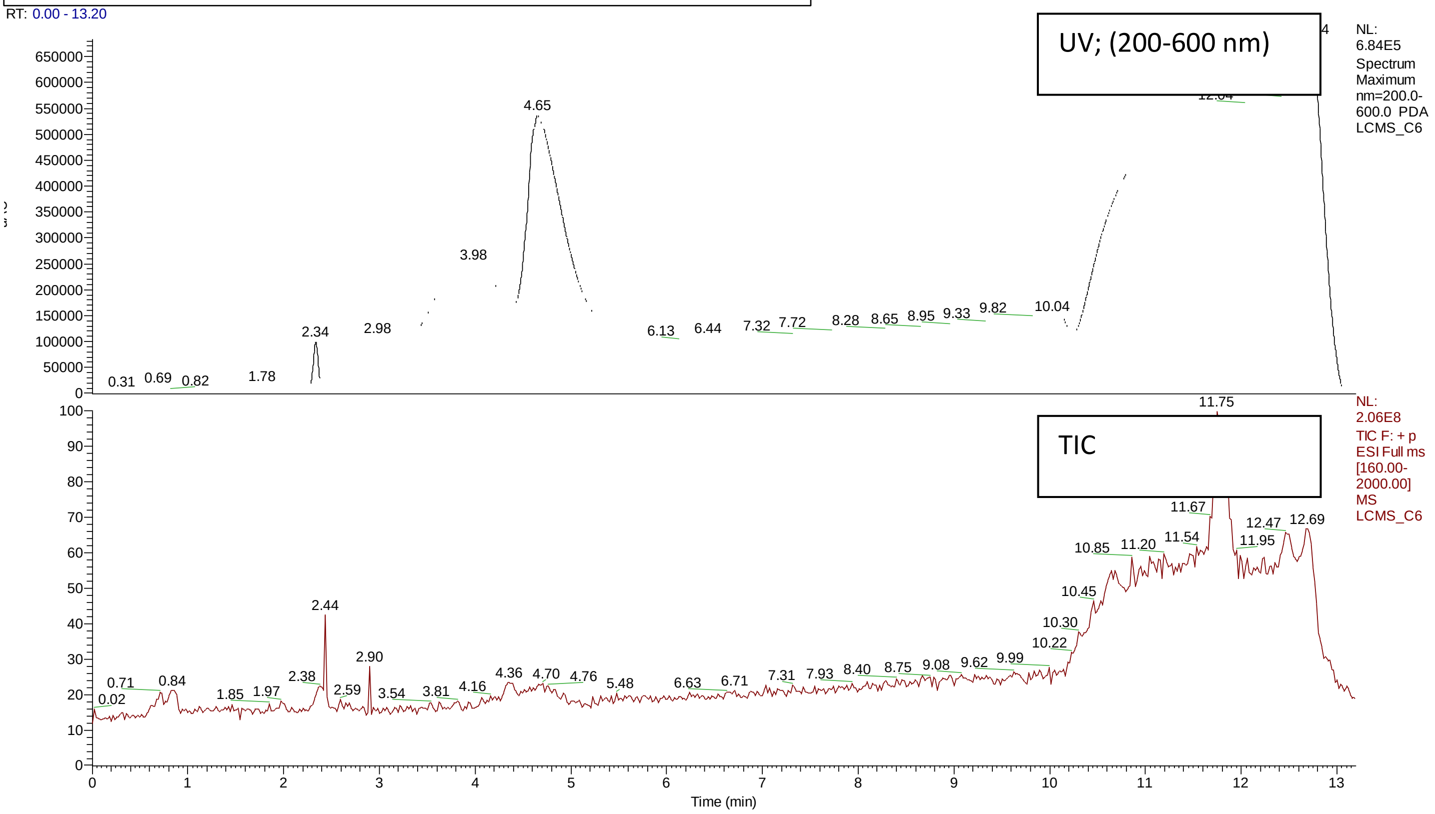

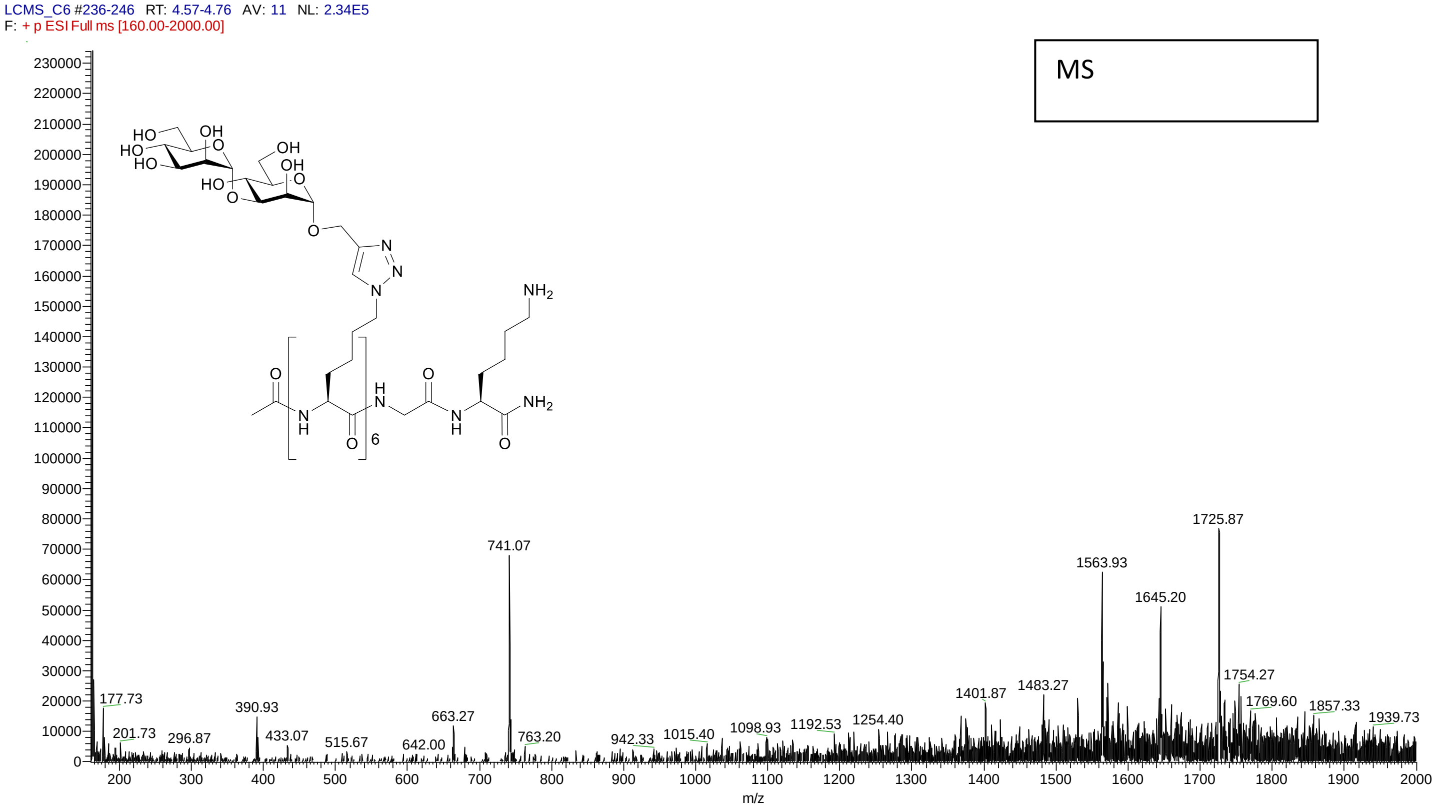

D1

LC-MS Spectra; *(0 –> 50 % ACN, 13 min);* (Compound **D1**)

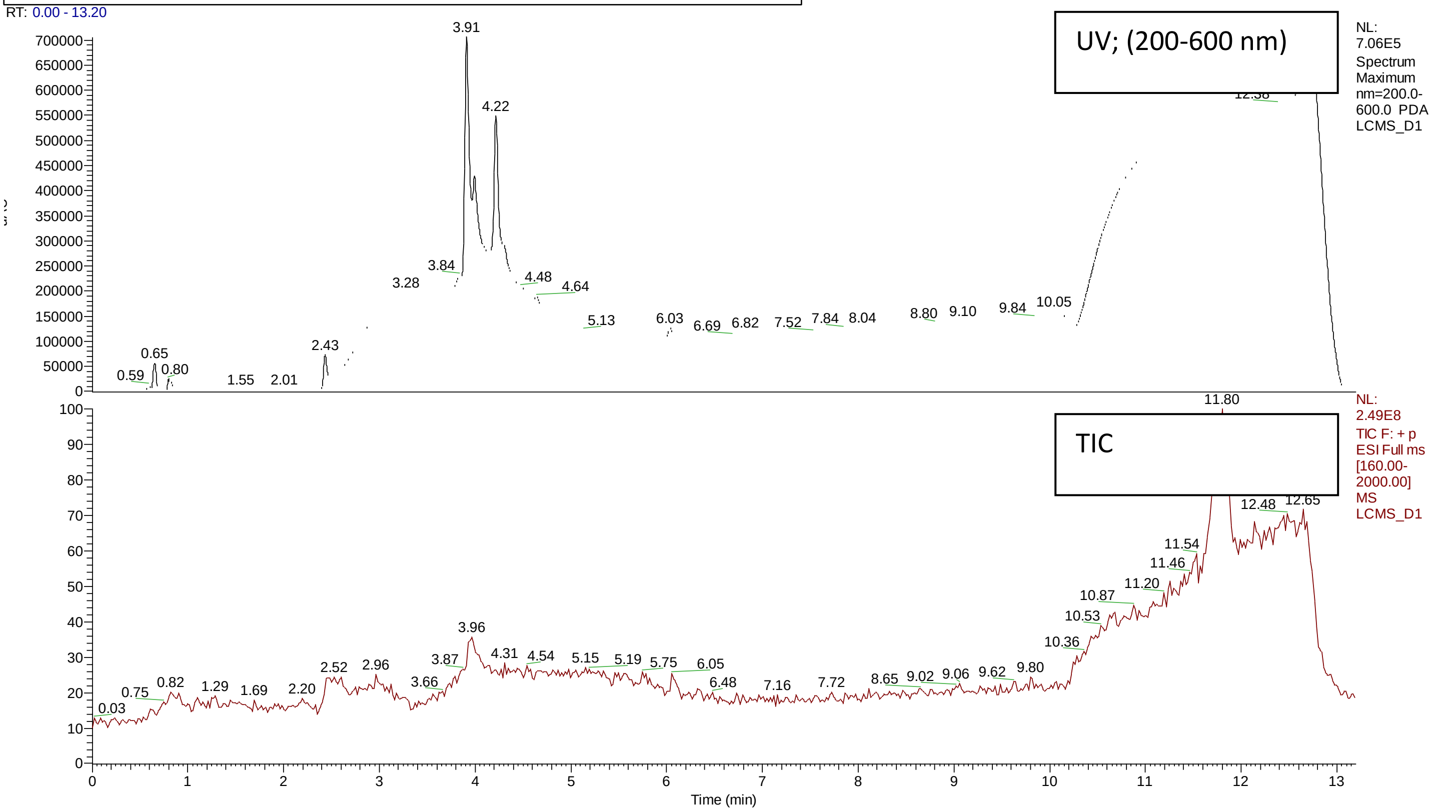

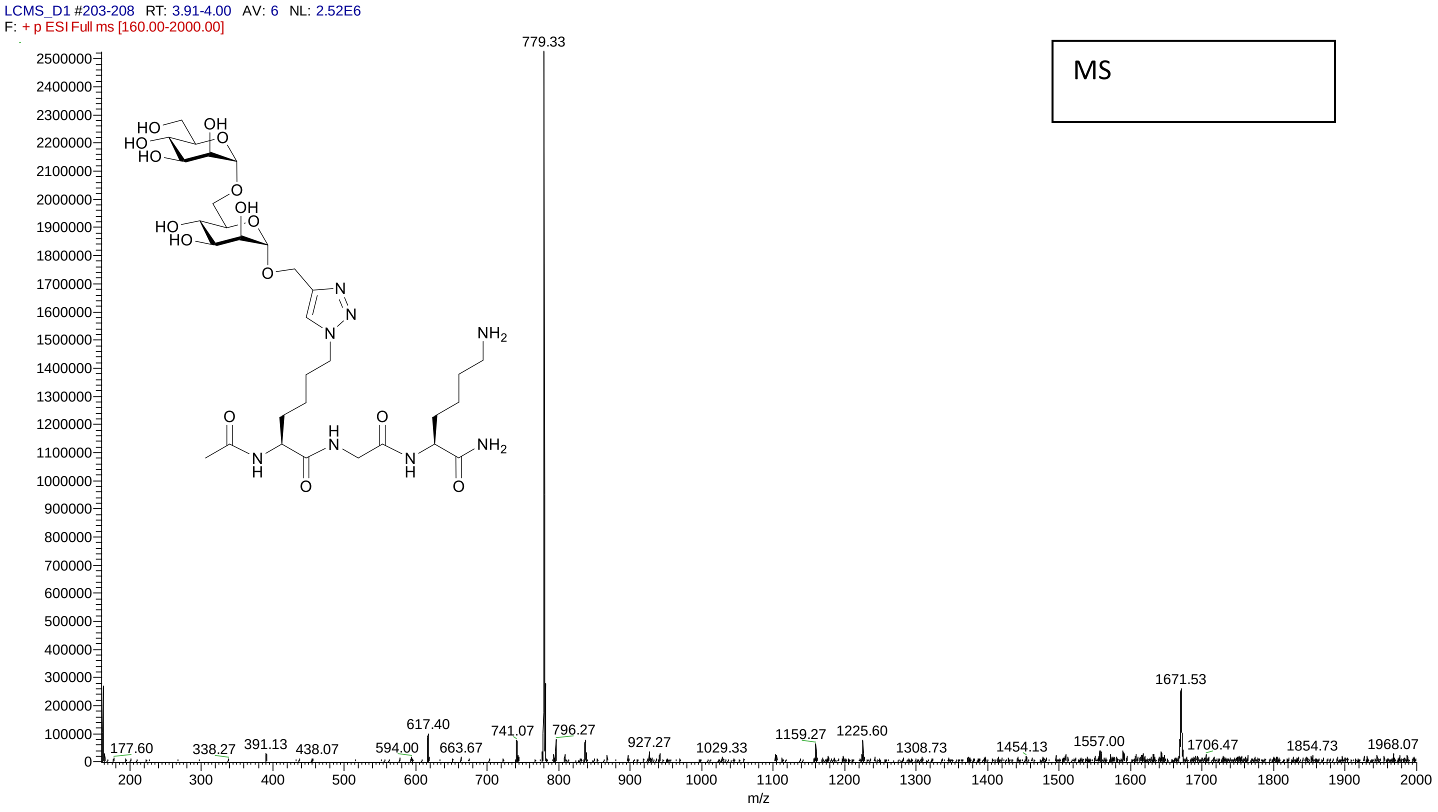

D2

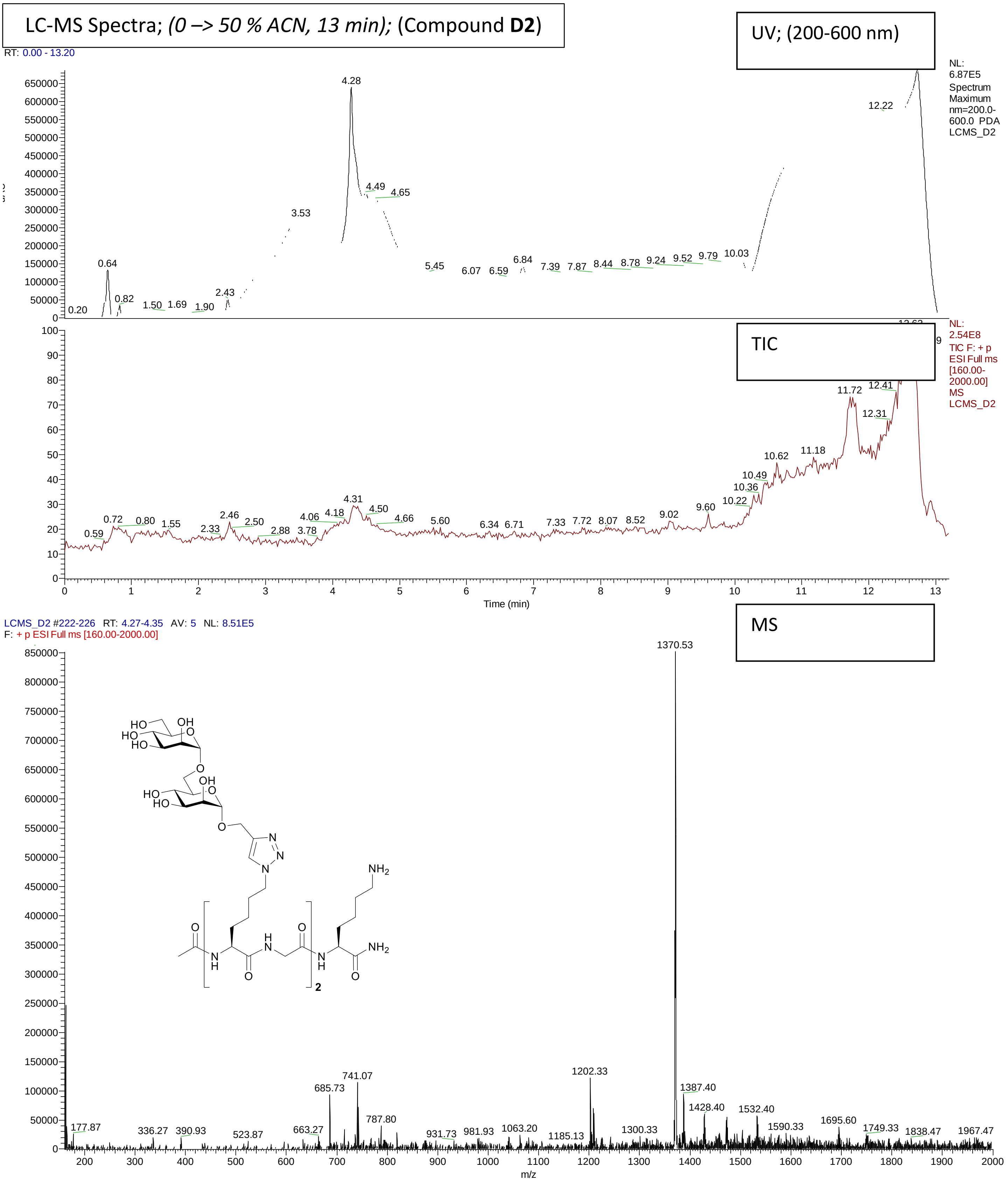
LC-MS Spectra; (0 –> 50 % ACN, 13 min); (Compound D2)
RT: 0.00 - 13.20
UV; (200-600 nm)
NL: 6.87E5 Spectrum Maximum nm=200.0-600.0 PDA LCMS_D2
4.28
0.64
12.22
TIC
NL: 2.54E8 TIC F: + p ESI Full ms [160.00-2000.00] MS LCMS_D2
Time (min)
MS
LCMS_D2 #222-226 RT: 4.27-4.35 AV: 5 NL: 8.51E5
F: + p ESI Full ms [160.00-2000.00]
1370.53
1202.33
741.07
685.73
1387.40
m/z
HO
OH
O
NH2
N
H
2

D3

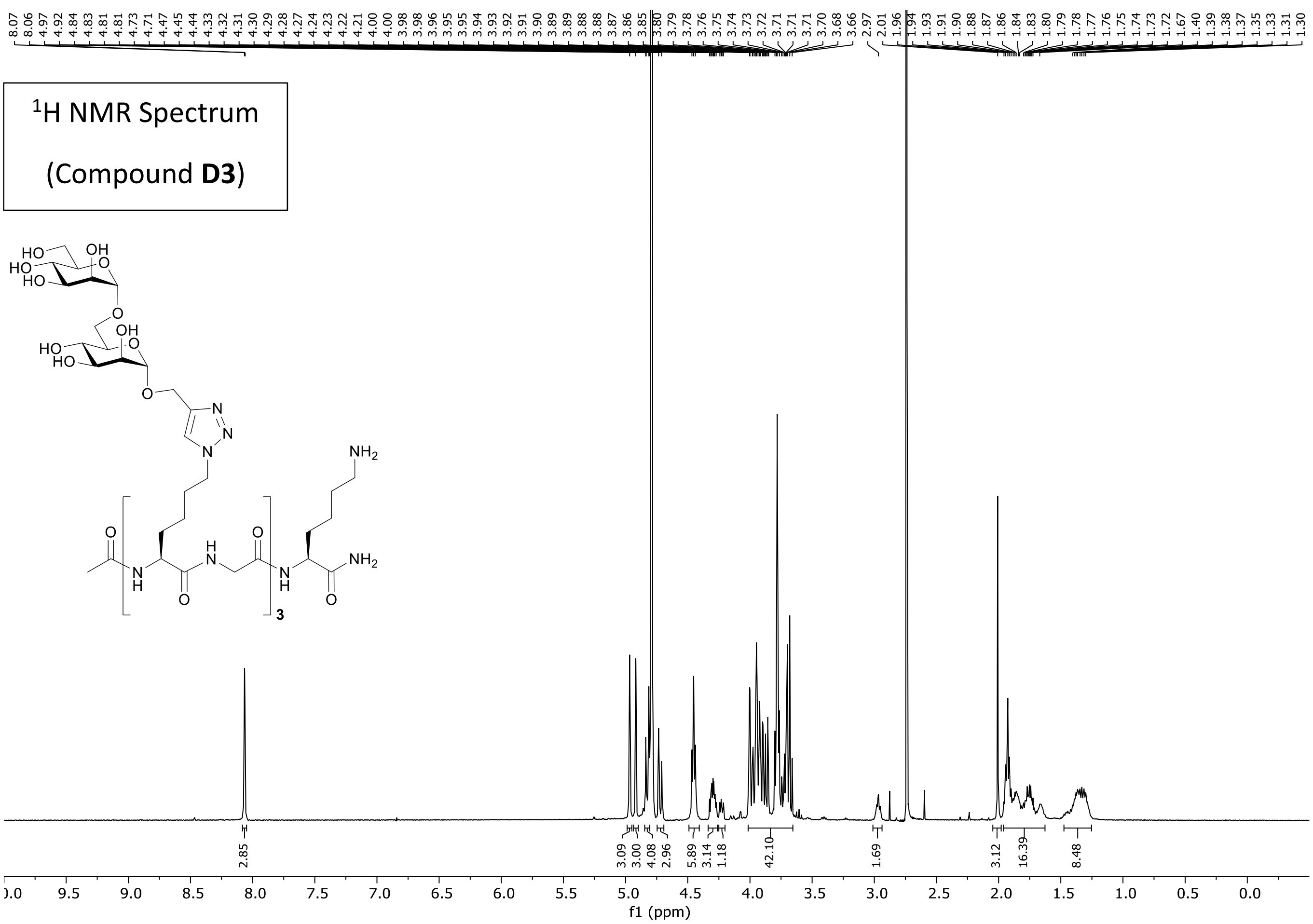

^{1}H NMR Spectrum

(Compound **D3**)

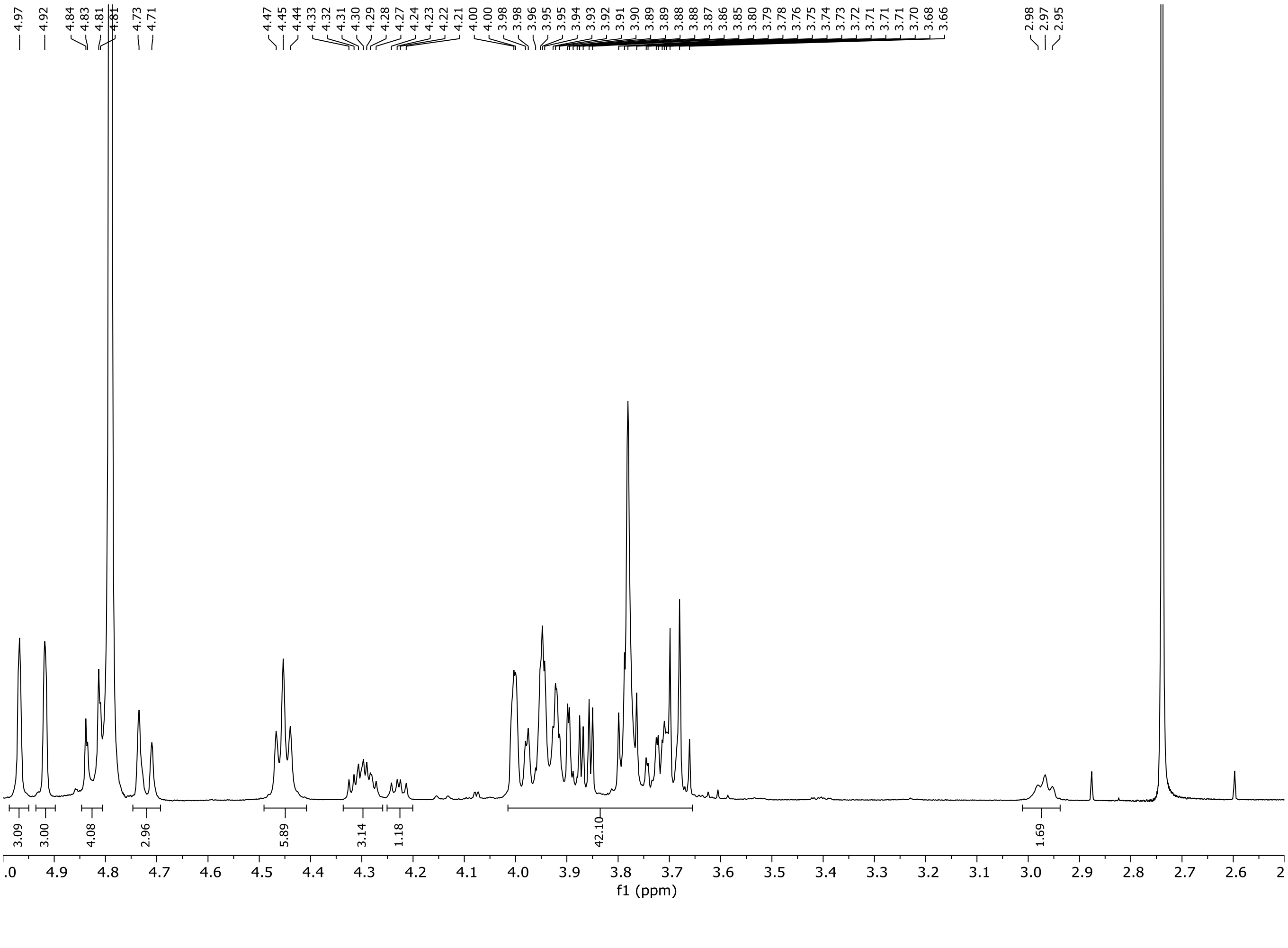

LC-MS Spectra; *(0 –> 50 % ACN, 13 min)*; (Compound **D3**)

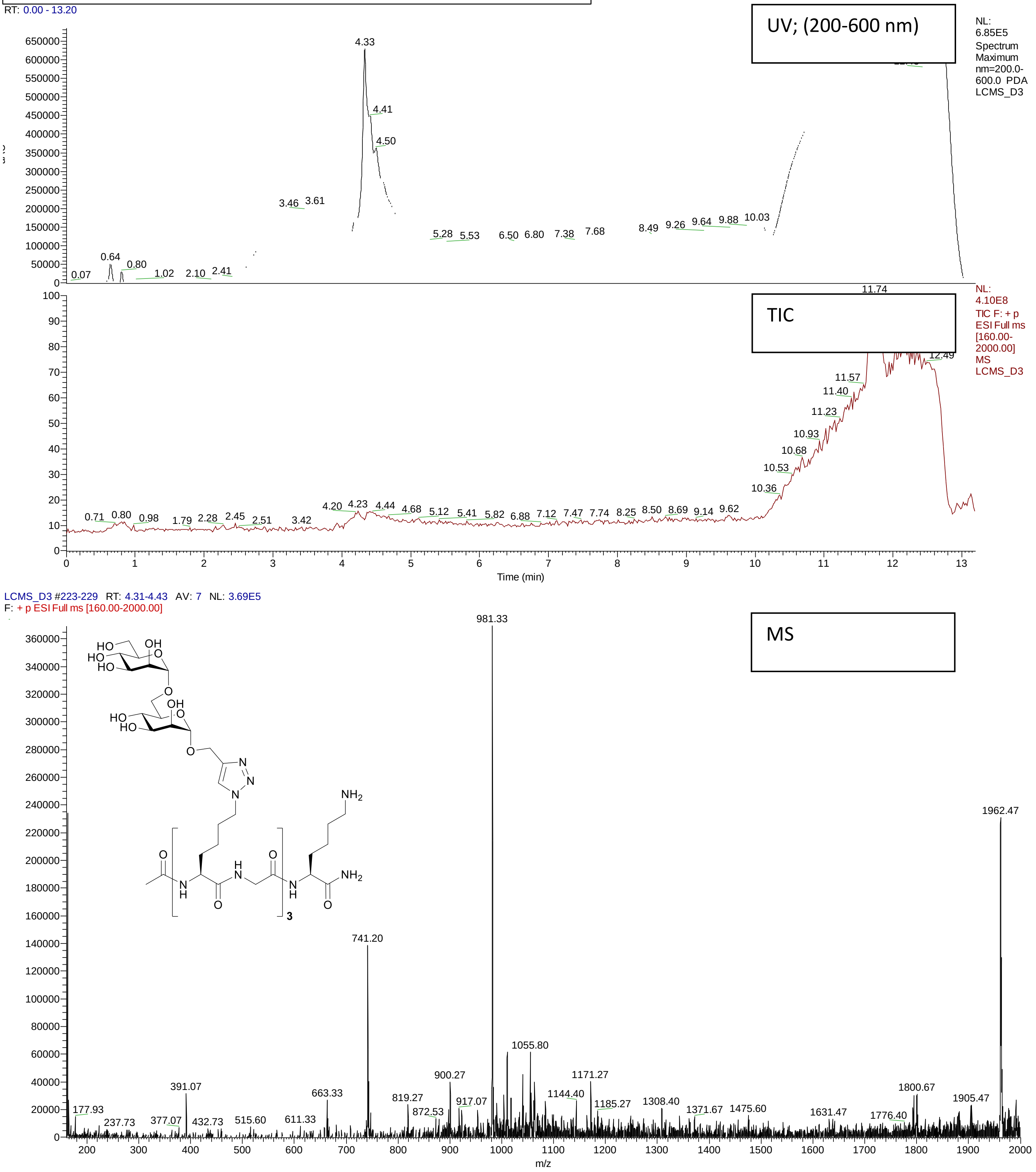

D6

LC-MS Spectra; *(0 –> 50 % ACN, 13 min);* (Compound **D6**)

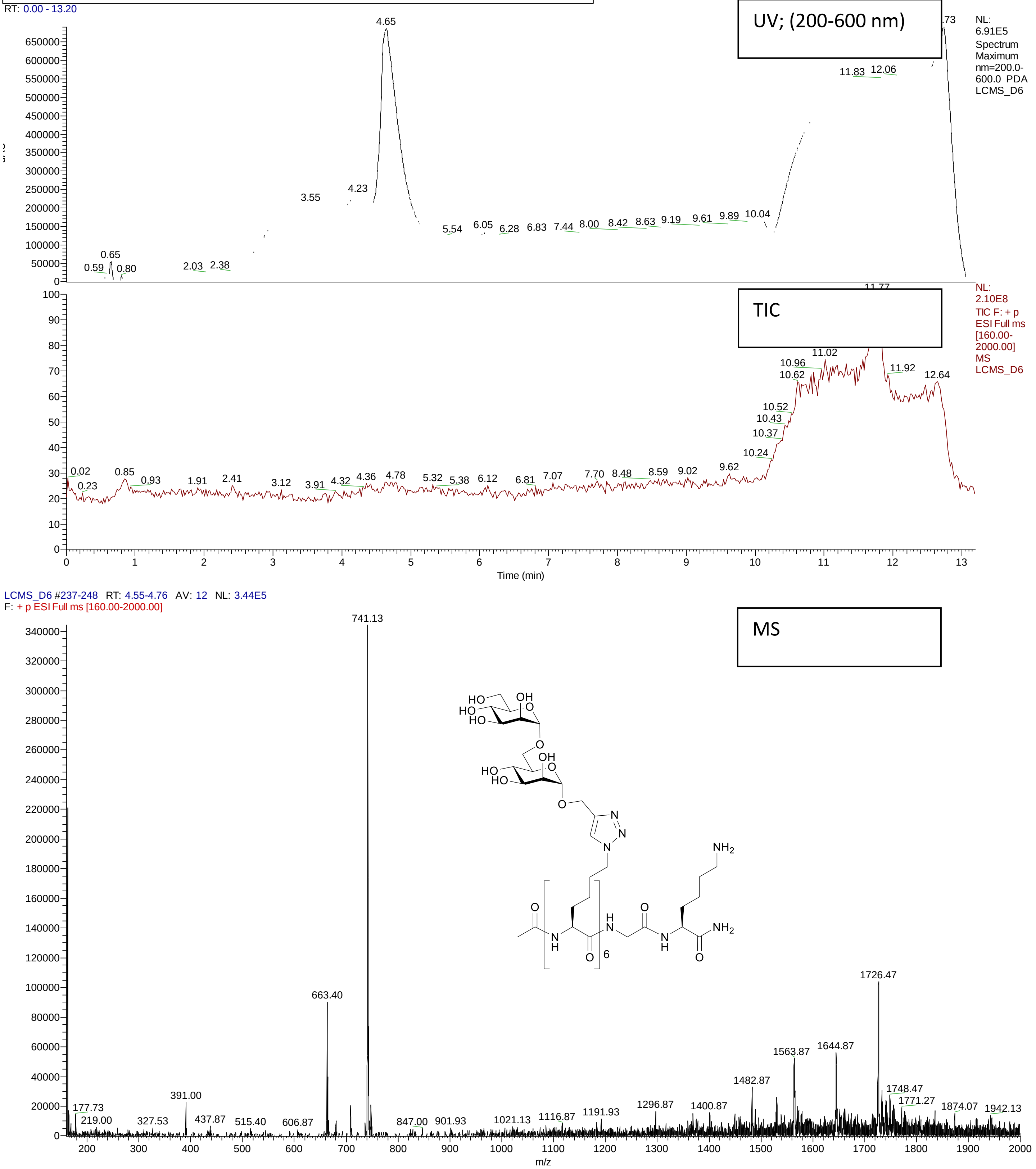

E1

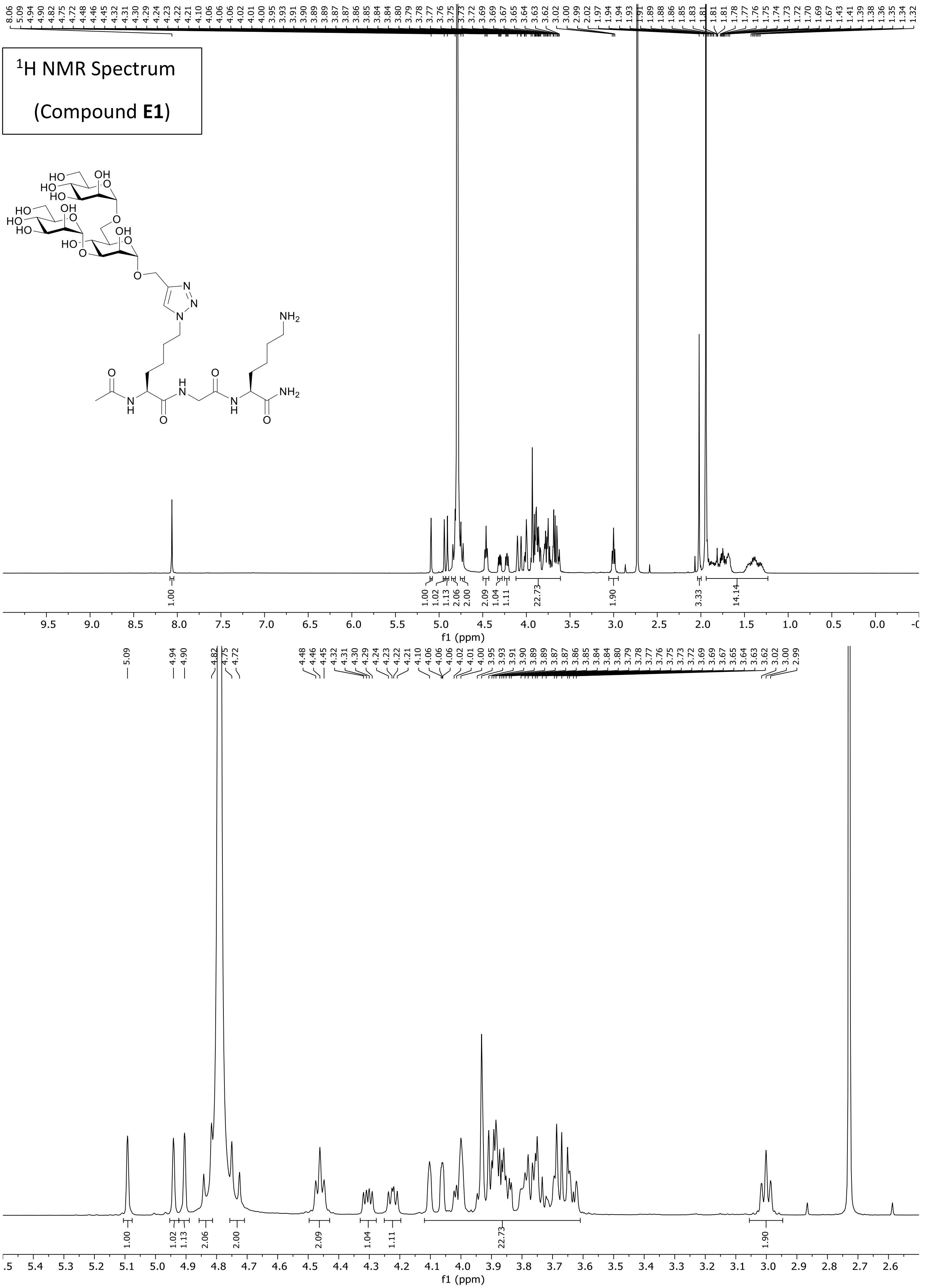

LC-MS Spectra; *(0 –> 50 % ACN, 13 min);* (Compound **E1**)

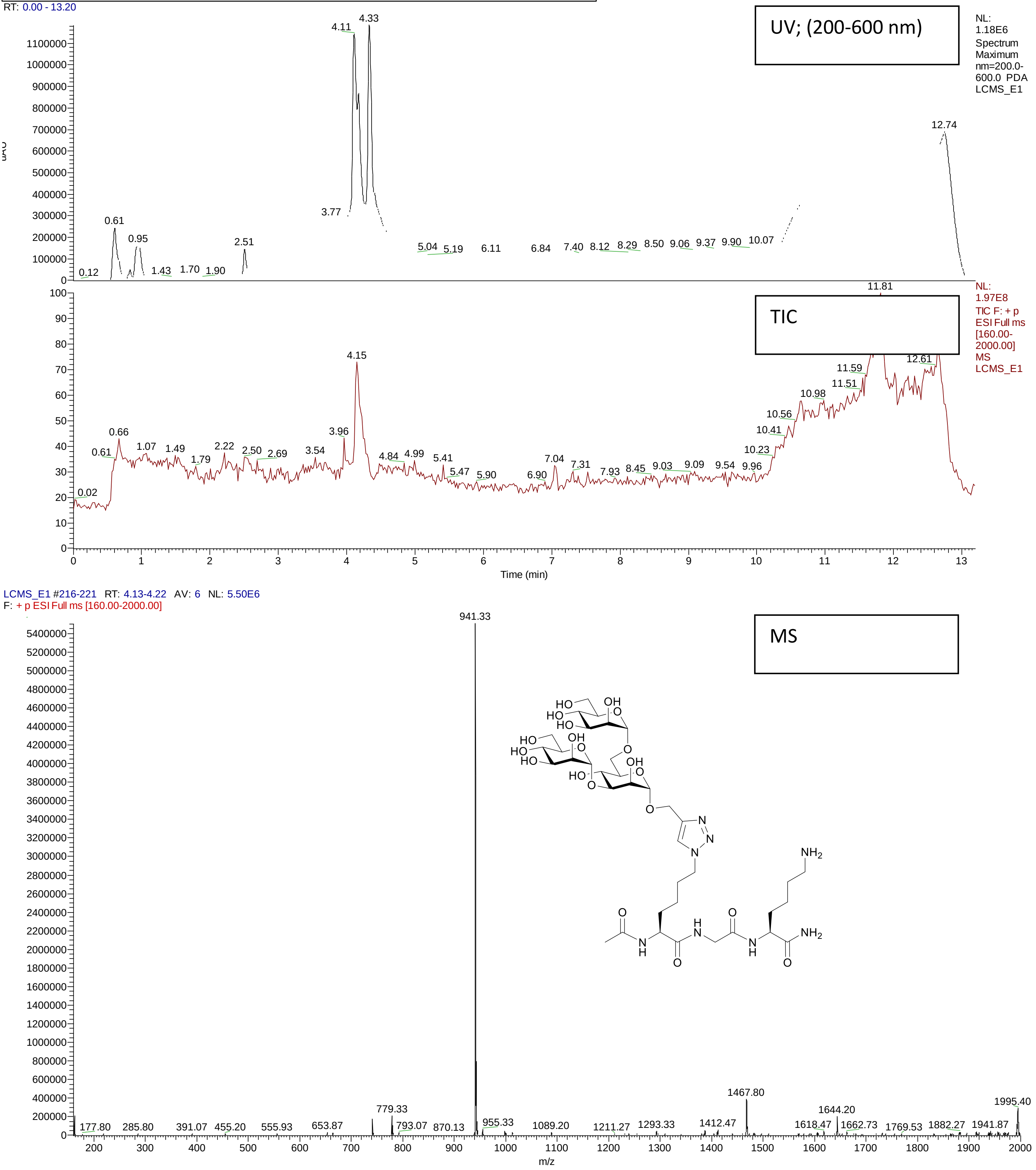

E2

LC-MS Spectra; *(0 –> 50 % ACN, 13 min);* (Compound **E2**)

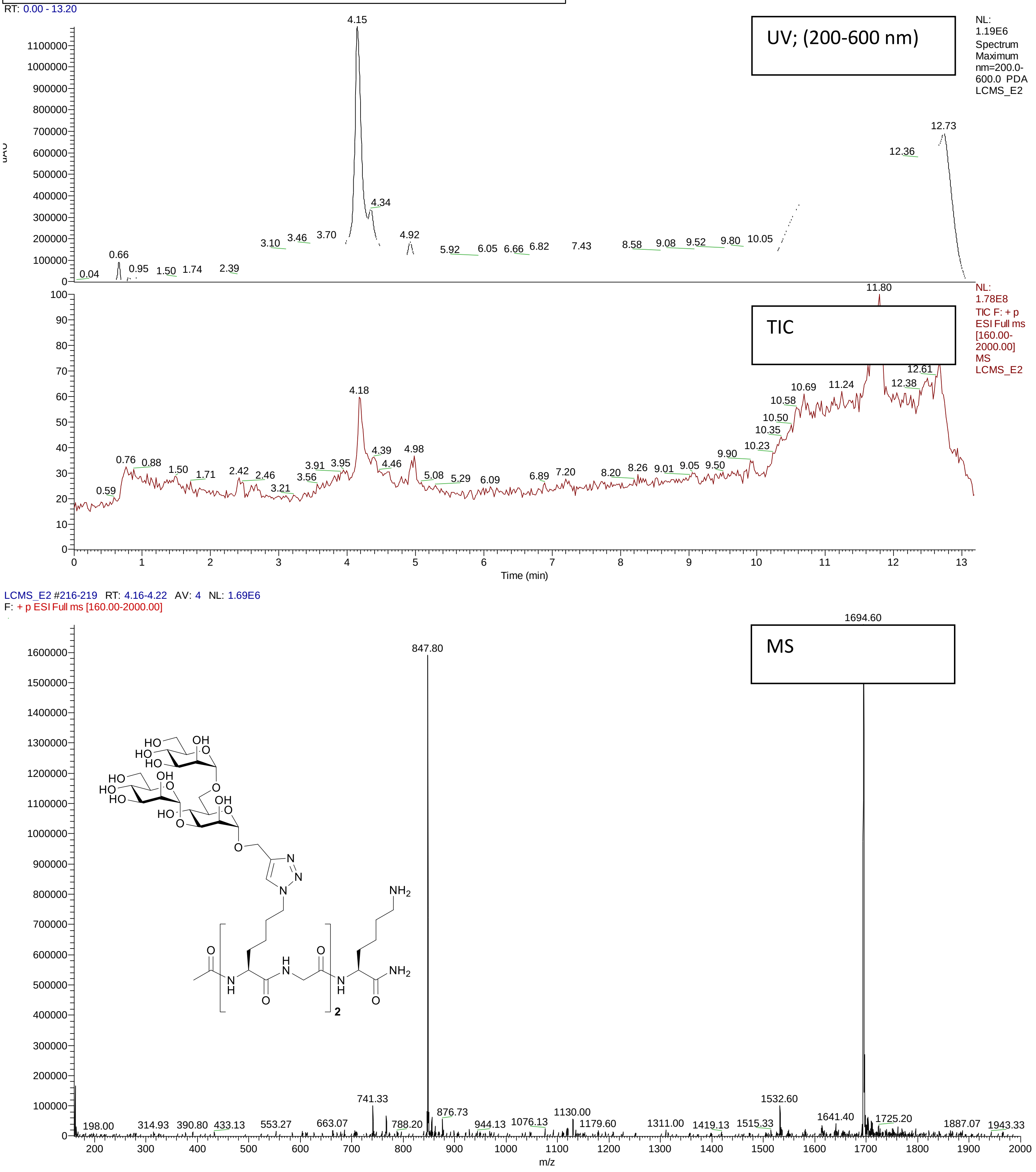

E3

^1H NMR Spectrum

(Compound **E3**)

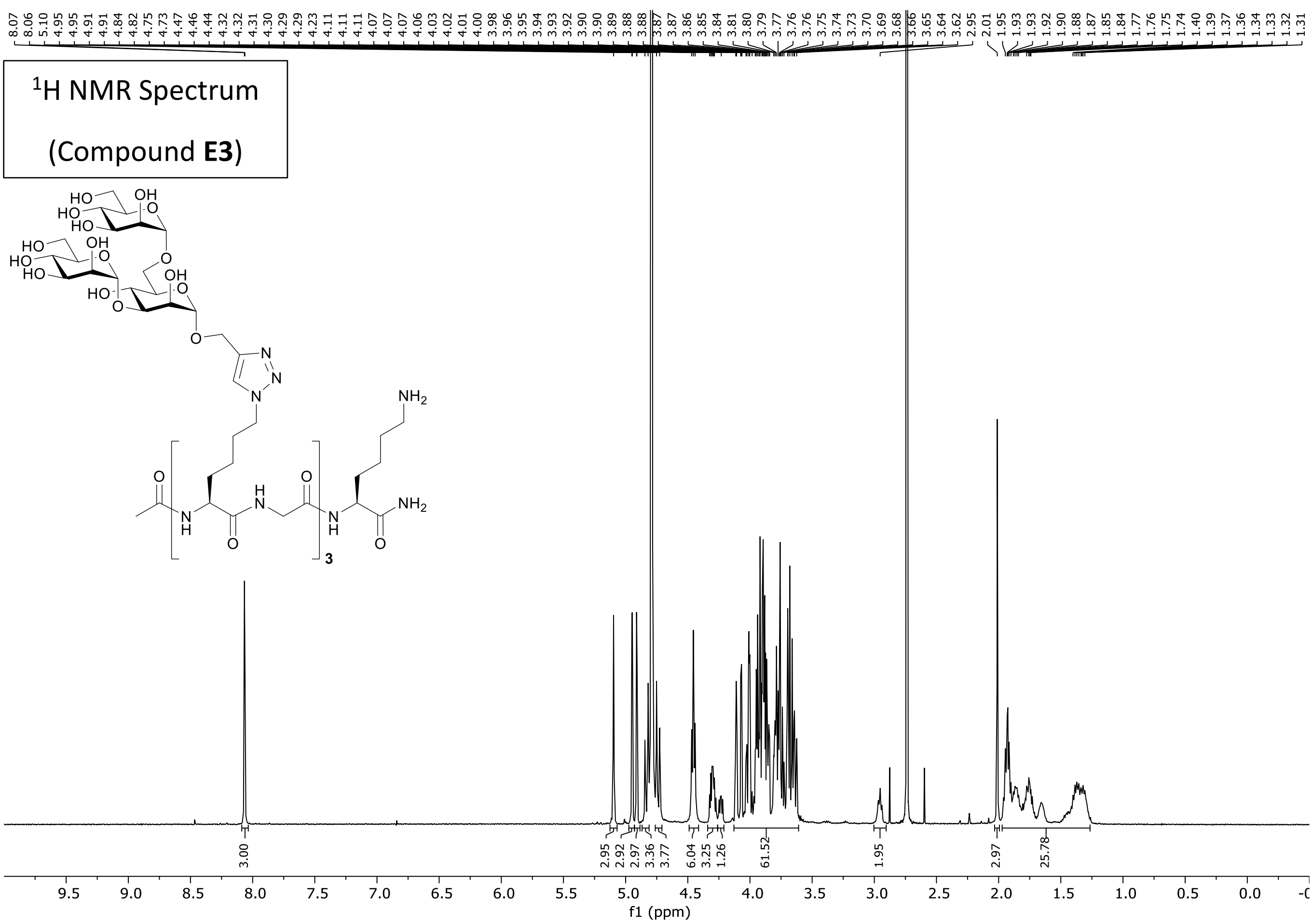

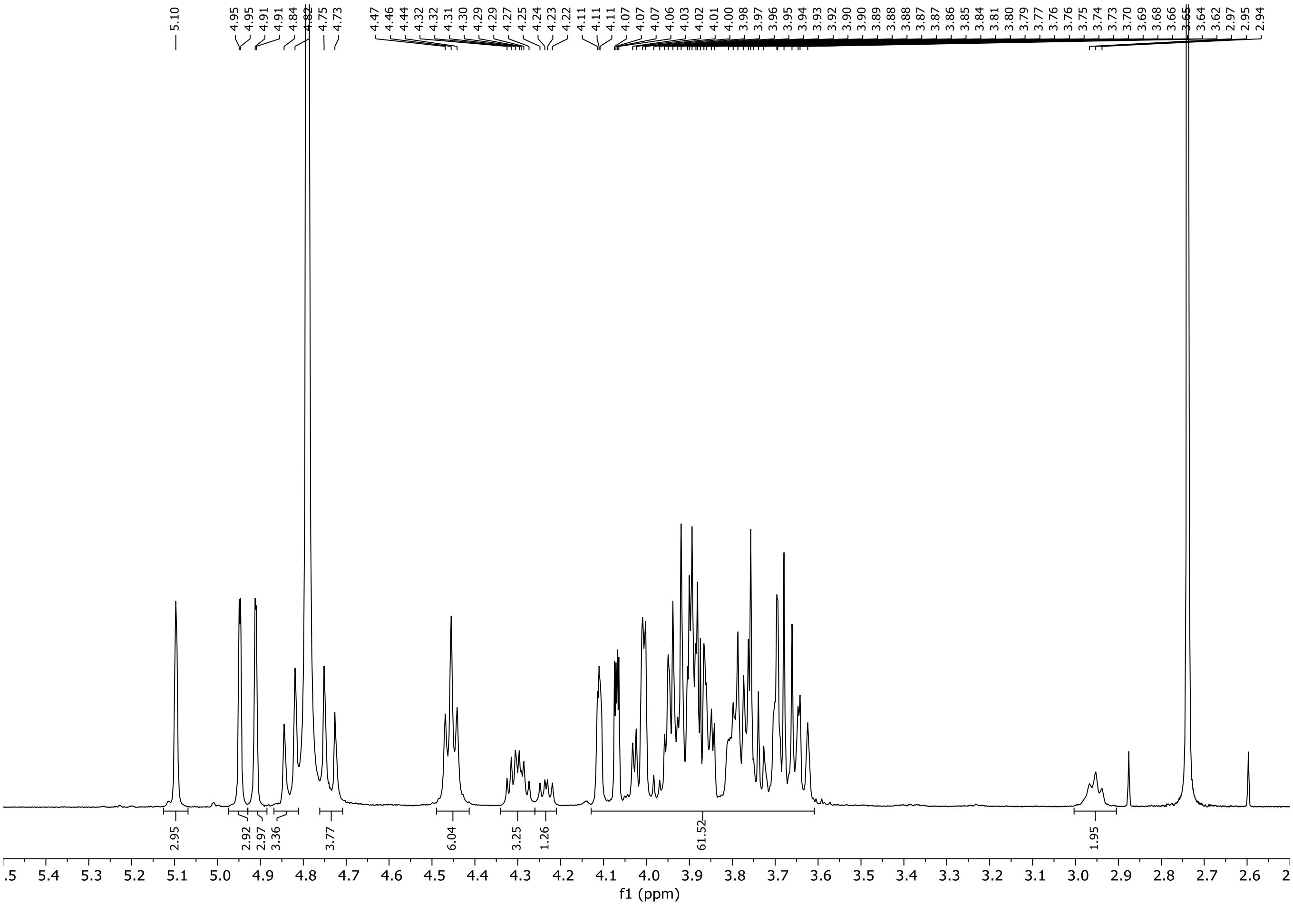

LC-MS Spectra; *(0 –> 50 % ACN, 13 min)*; (Compound **E3**)

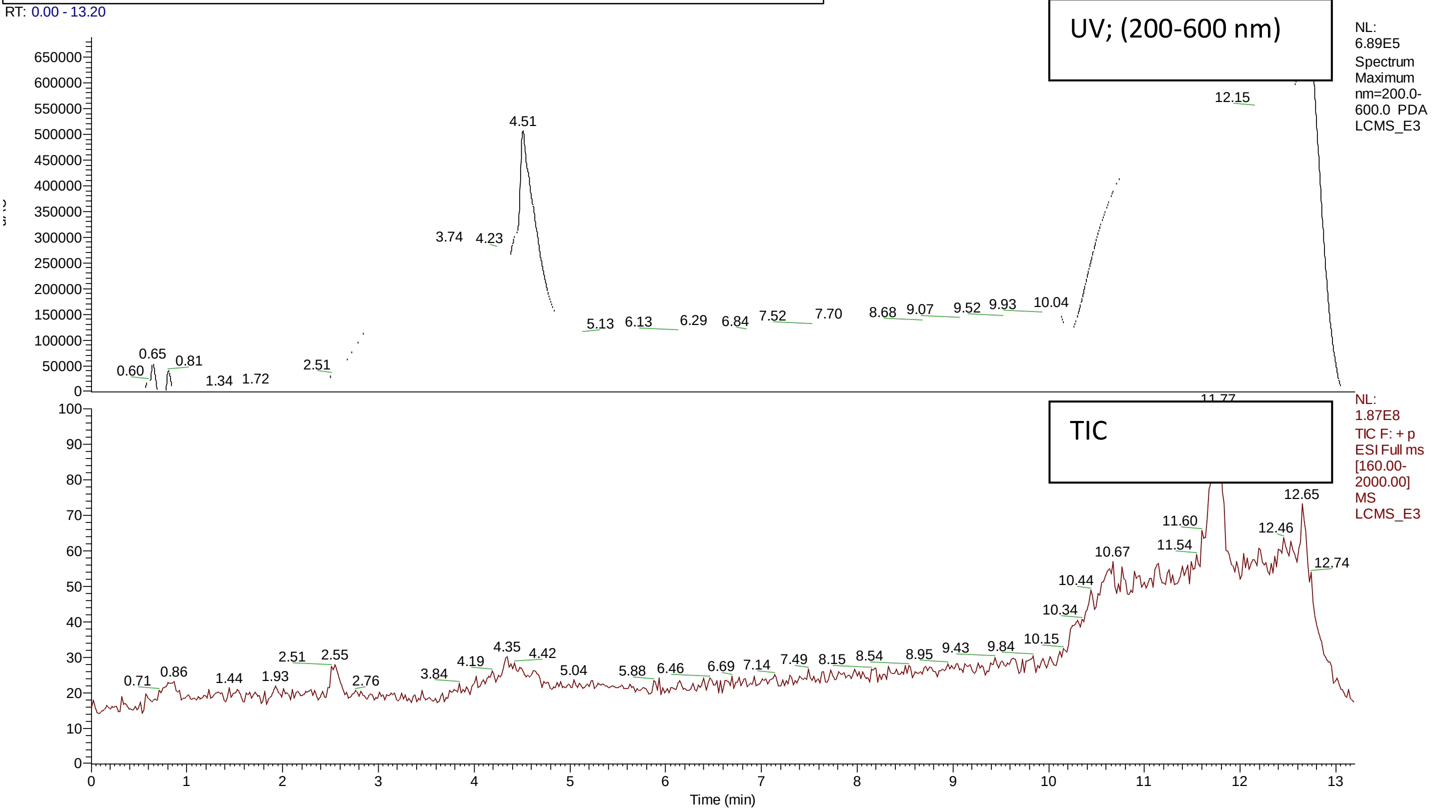

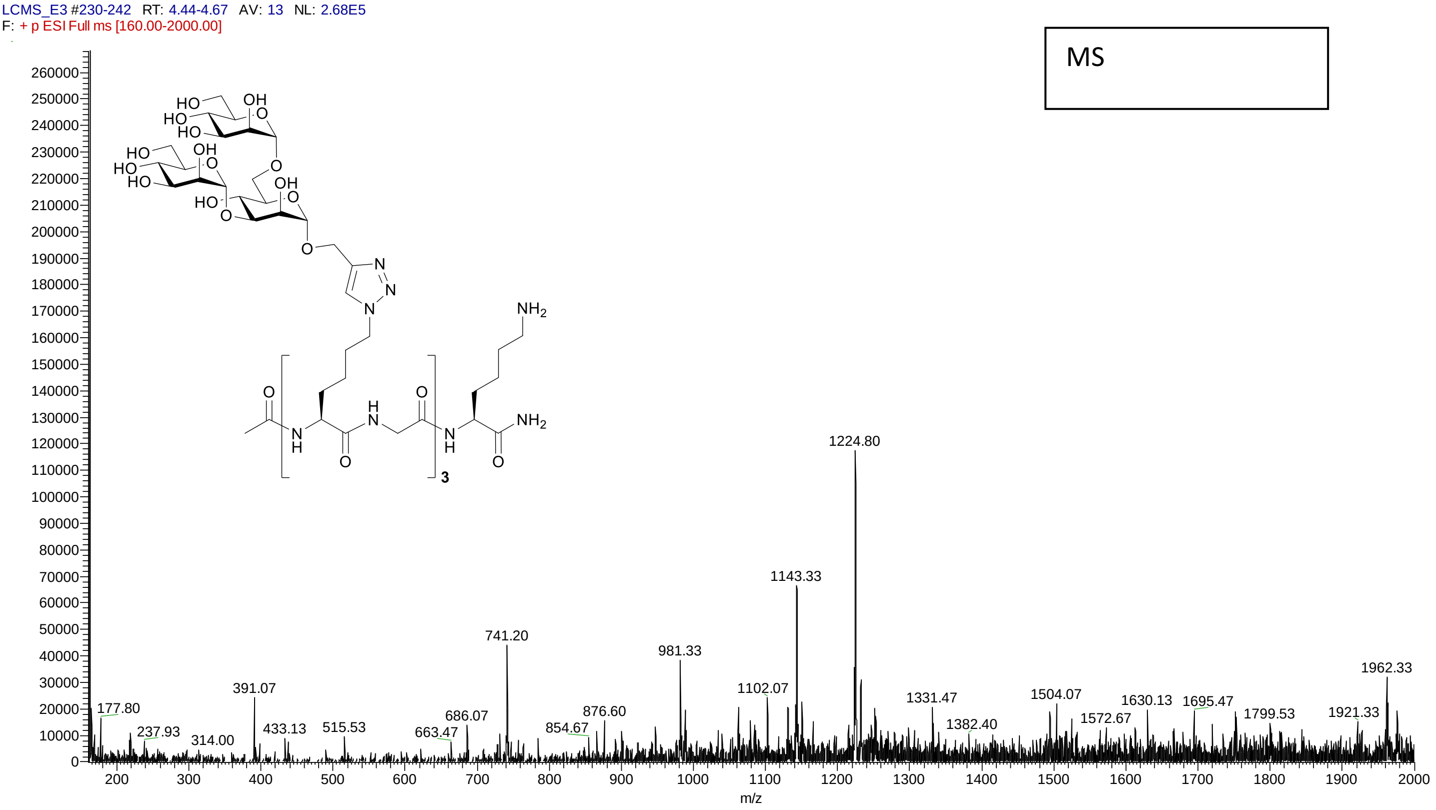

E6

^1H NMR Spectrum

(Compound **E6**)

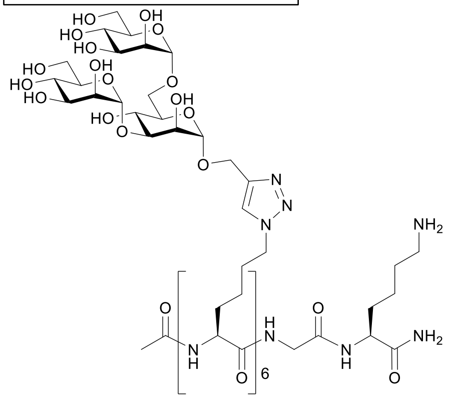

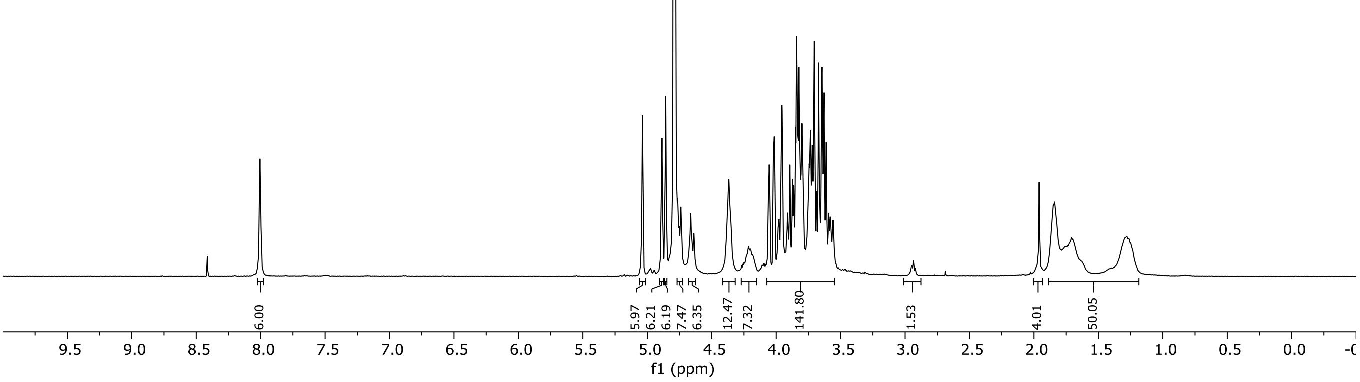

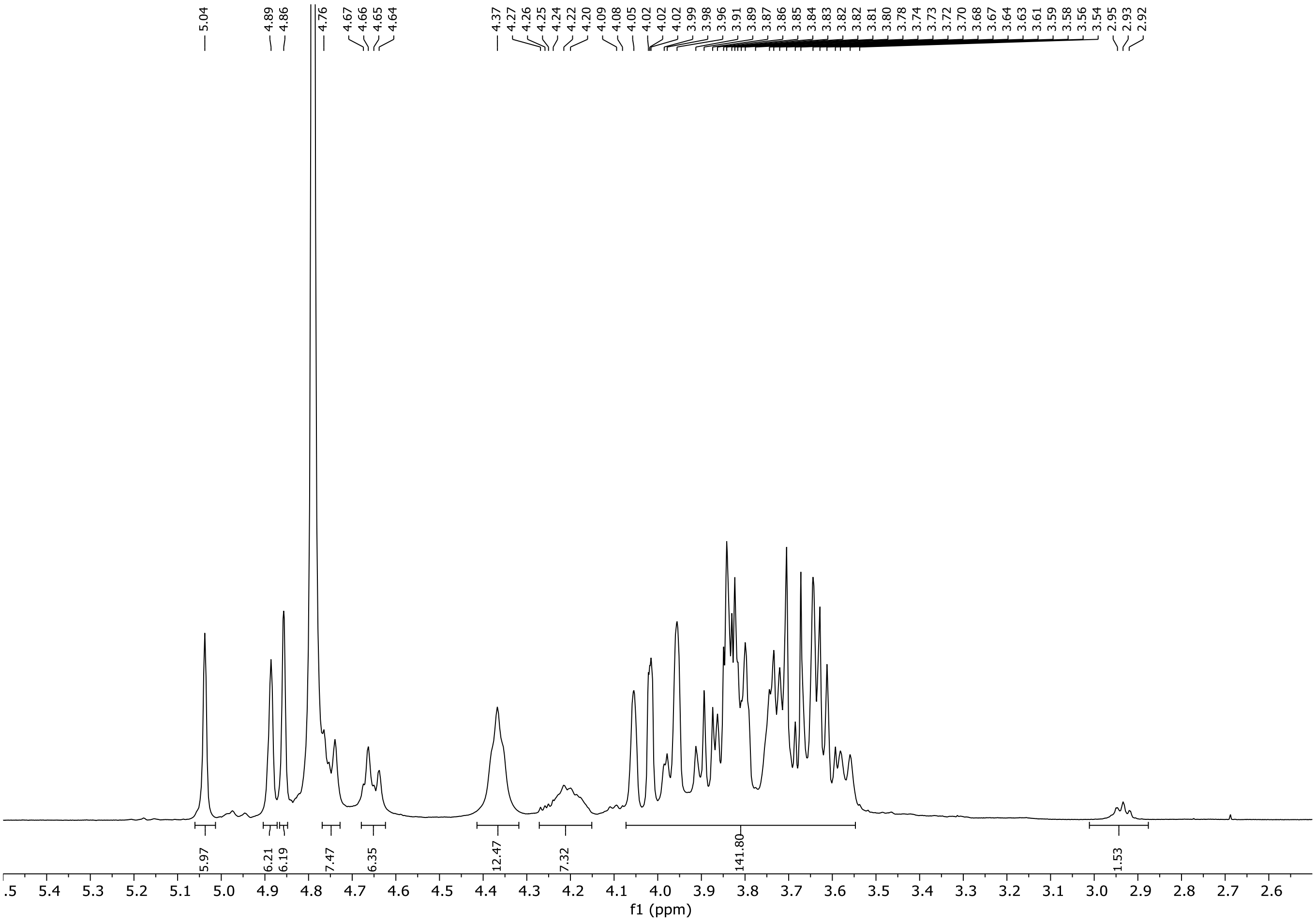

^{13}C APT NMR Spectrum

(Compound **E6**)

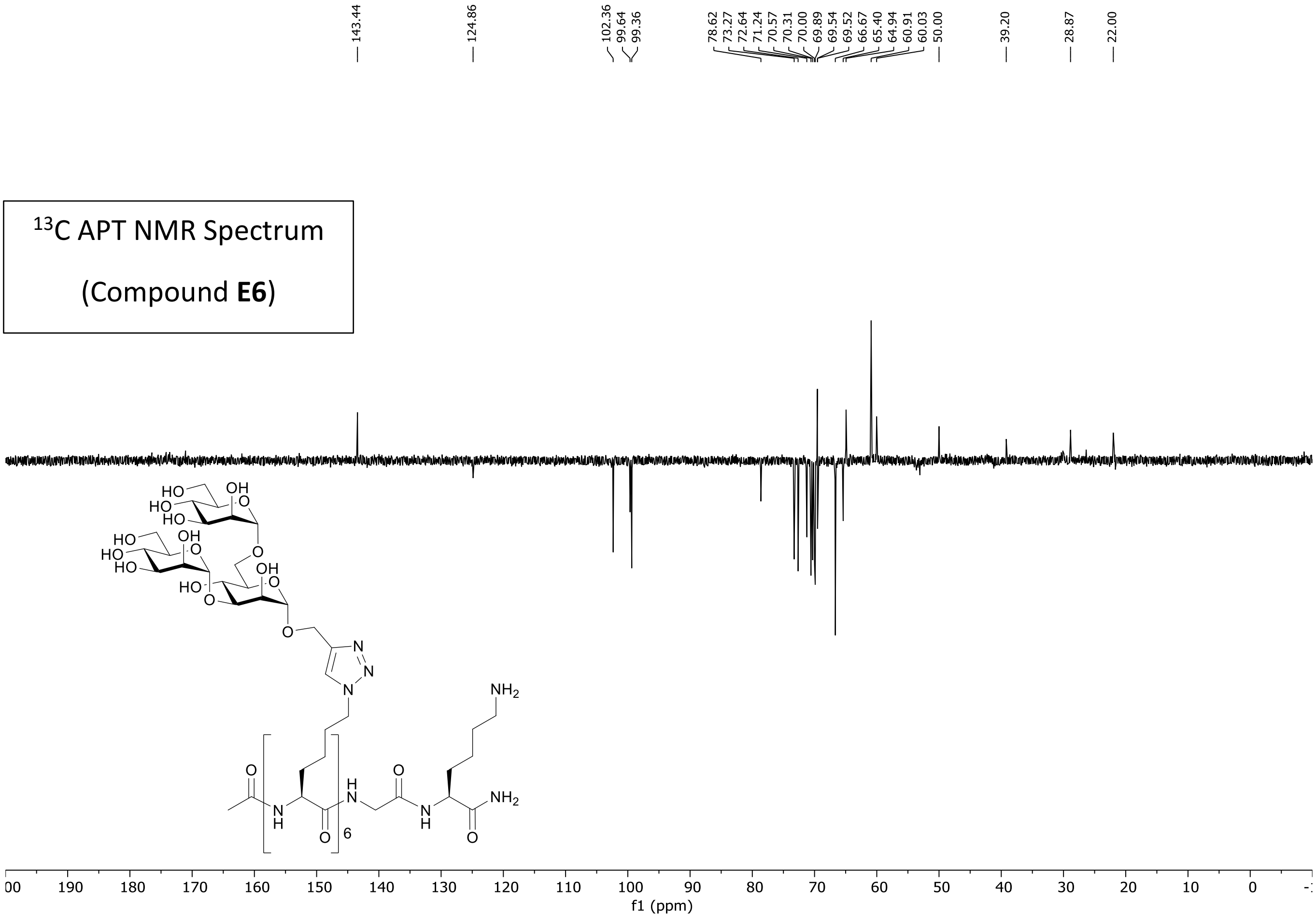

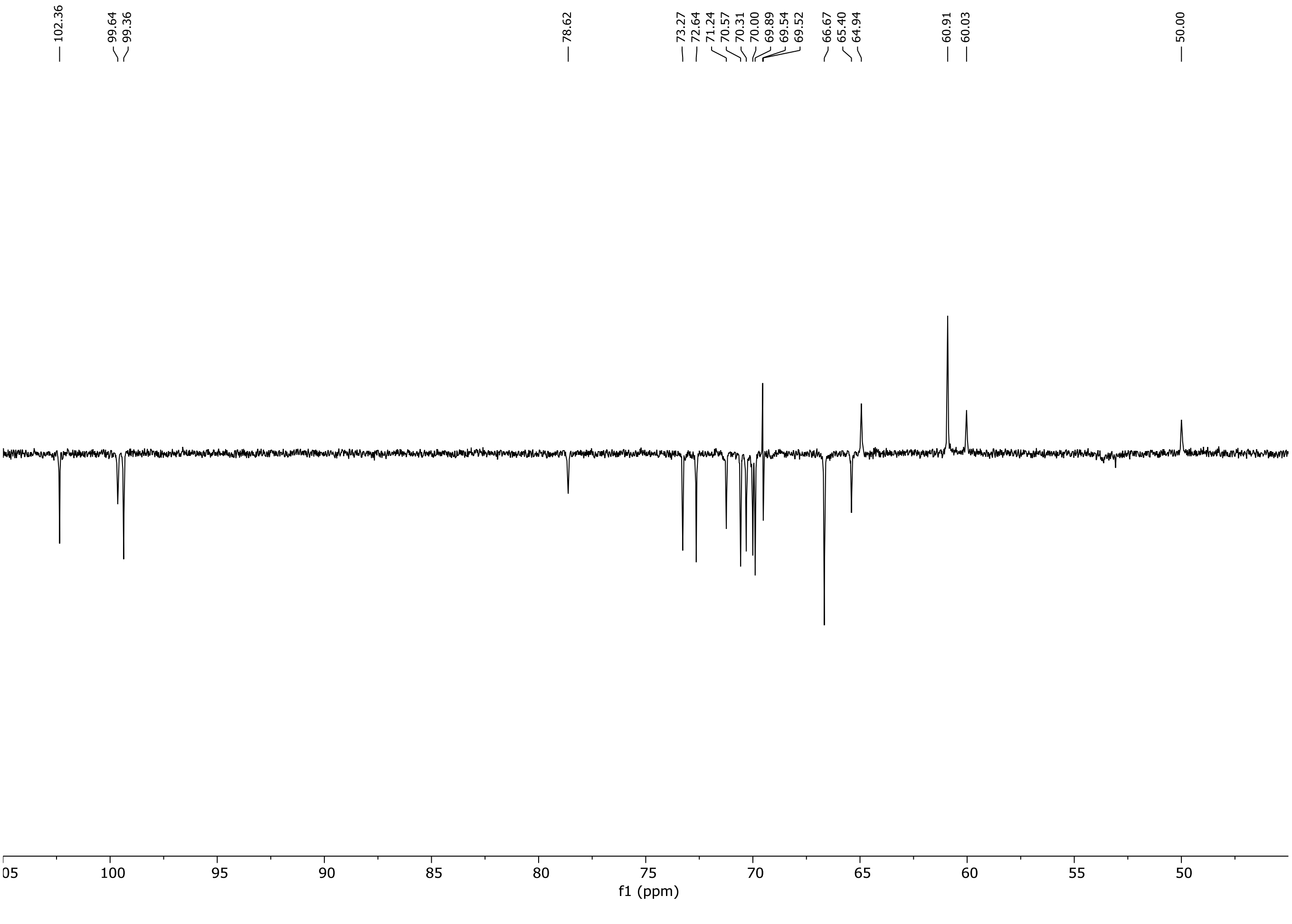

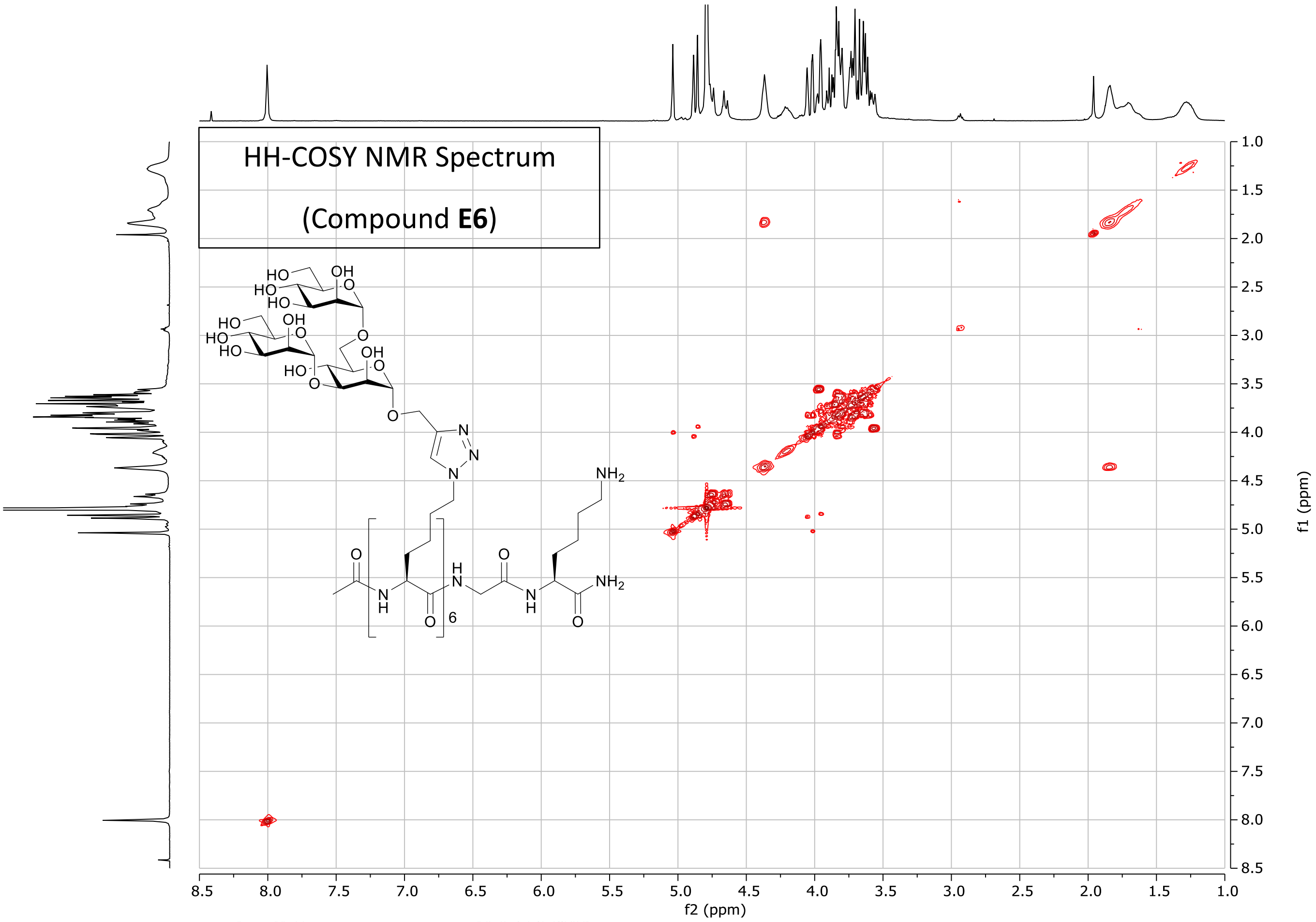
HH-COSY NMR Spectrum
(Compound **E6**)
HO
OH
HO
O
HO
HO
OH
O
O
HO
HO
OH
O
HO
O
O
O
N
N
N
NH2
O
O
N
H
H
N
O
6
N
H
NH2
O
f2 (ppm)
f1 (ppm)

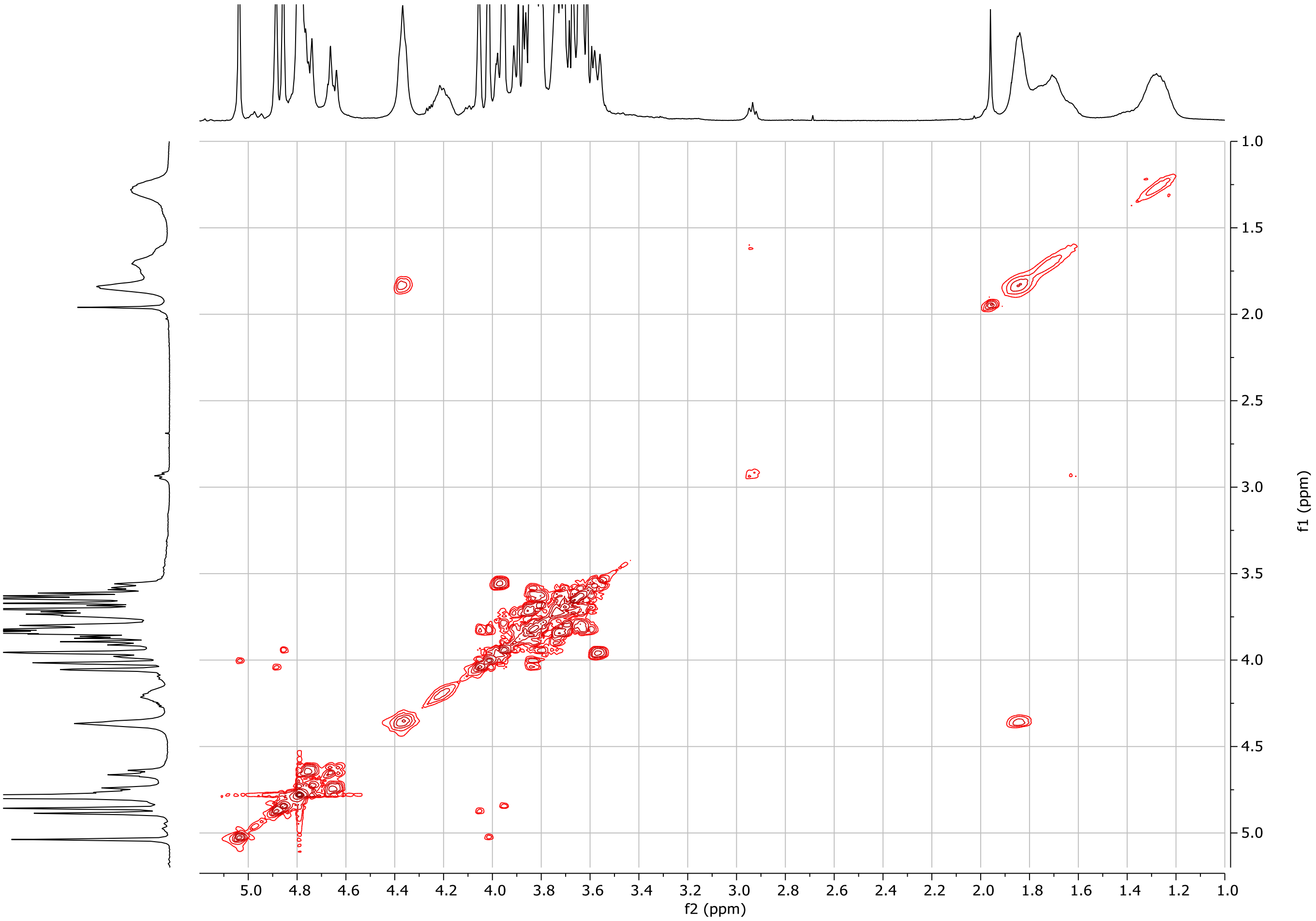
f2 (ppm)
f1 (ppm)

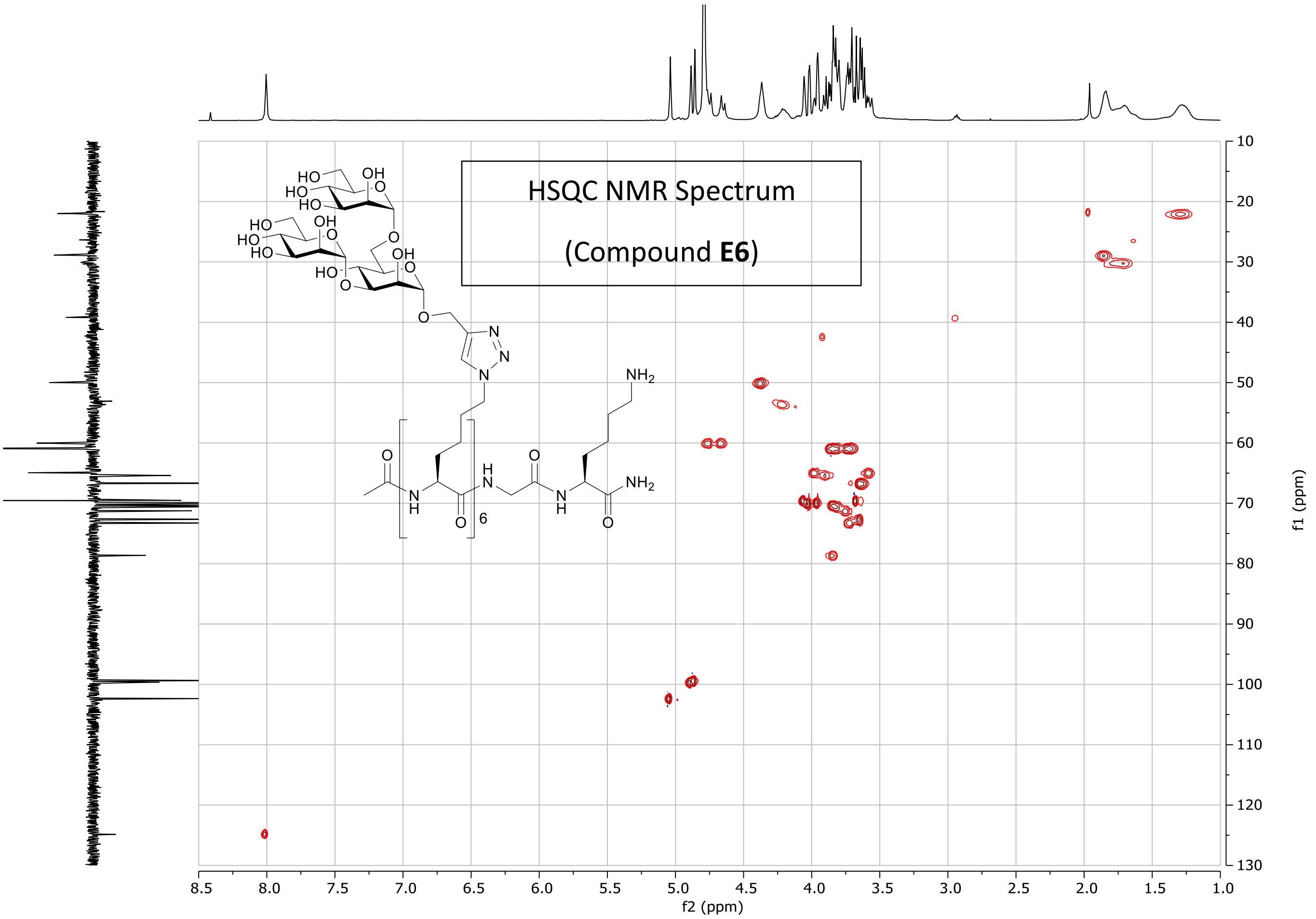
HSQC NMR Spectrum
(Compound E6)
f2 (ppm)
f1 (ppm)

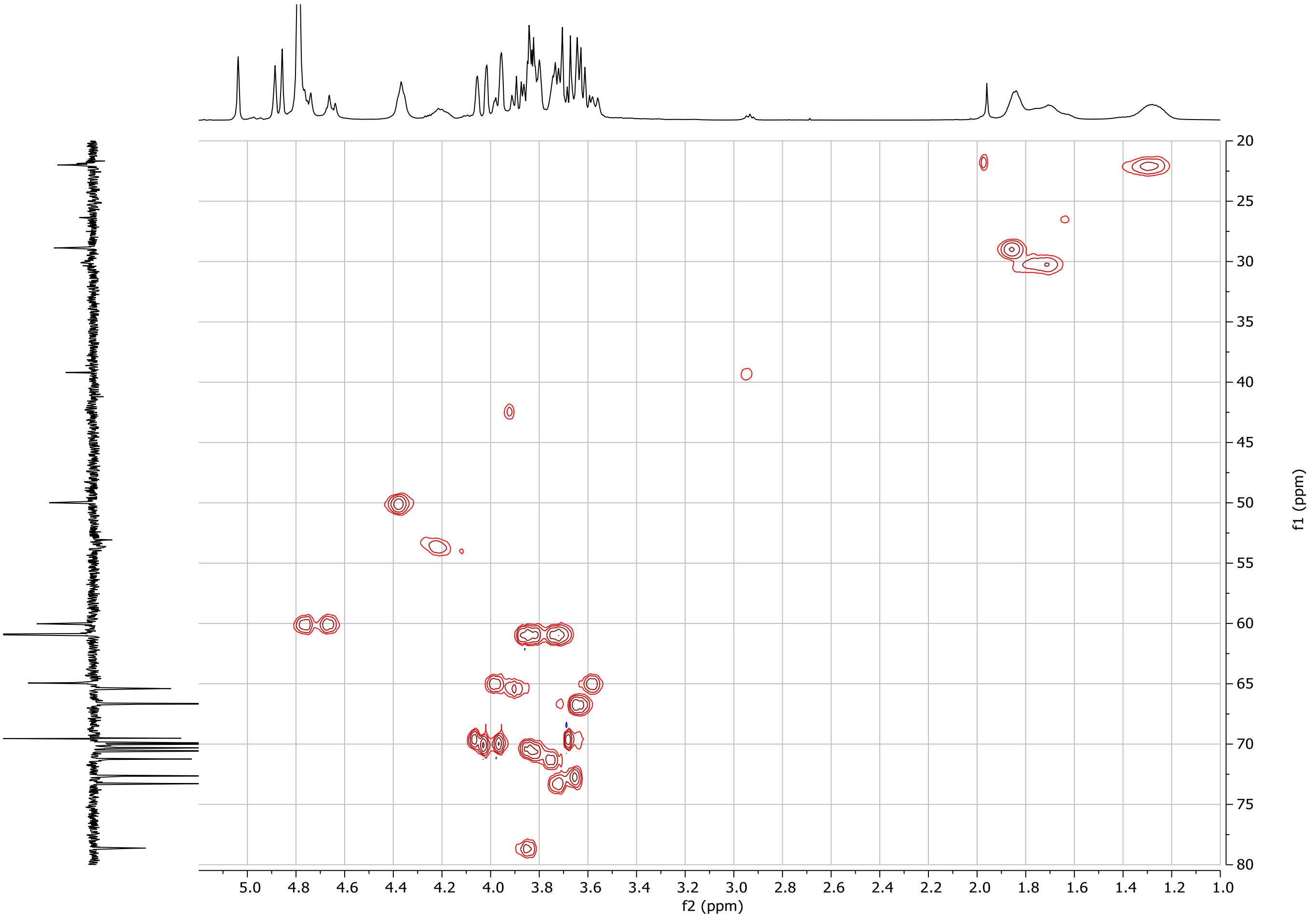
f2 (ppm)
f1 (ppm)

LC-MS Spectra; *(0 –> 50 % ACN, 13 min);* (Compound **E6**)

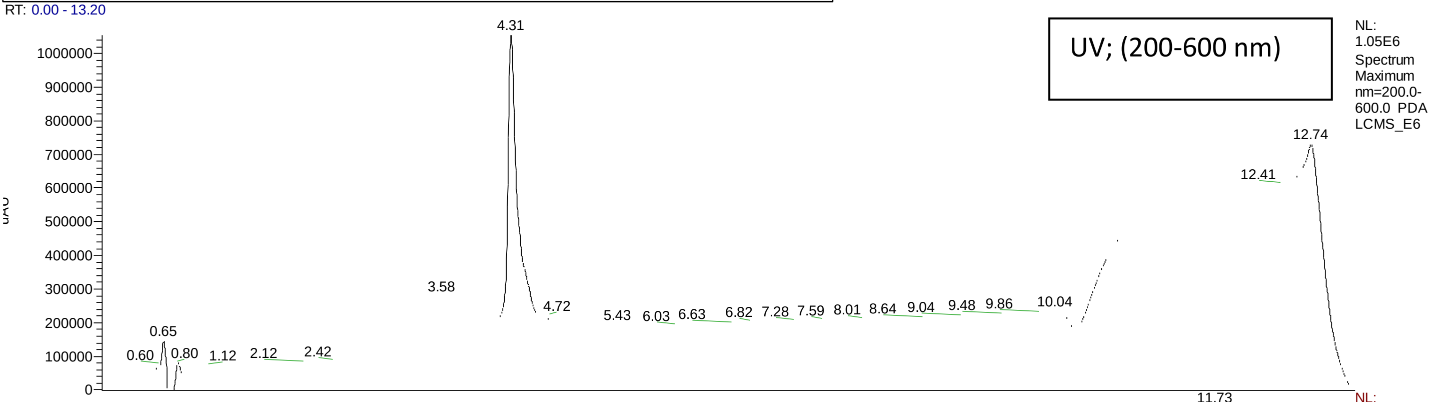

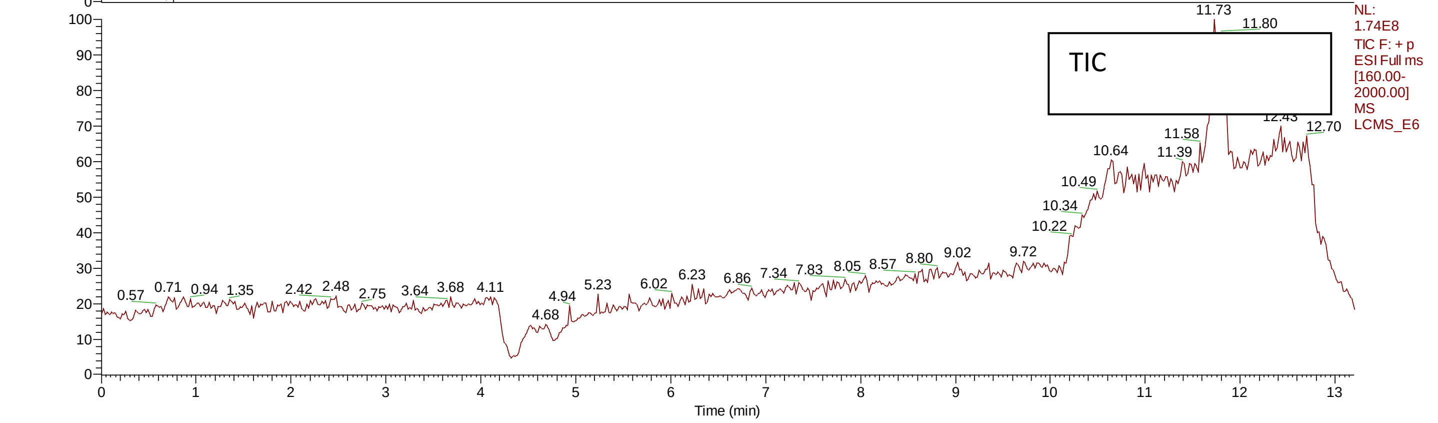

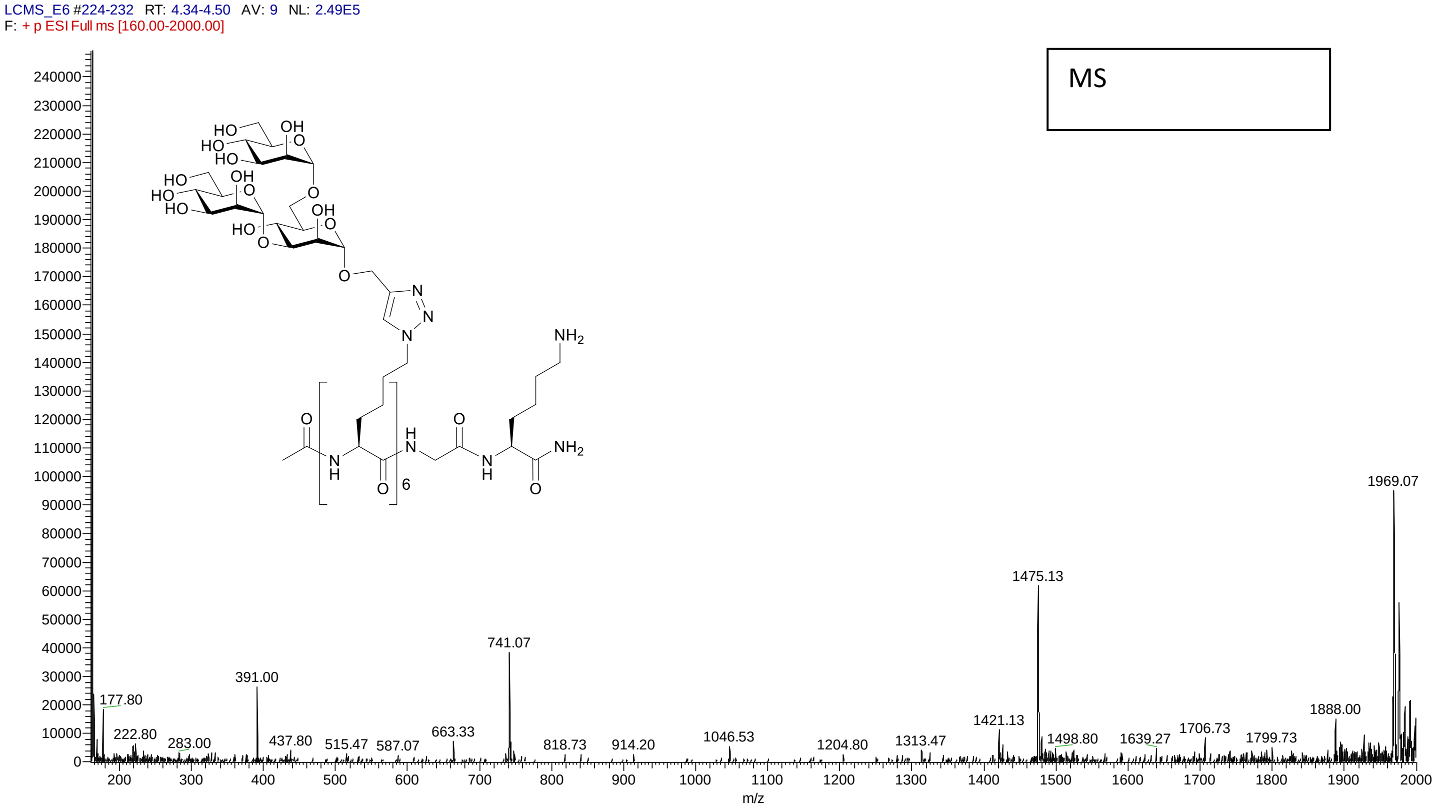

G1

^{1}H NMR Spectrum

(Compound **G1**)

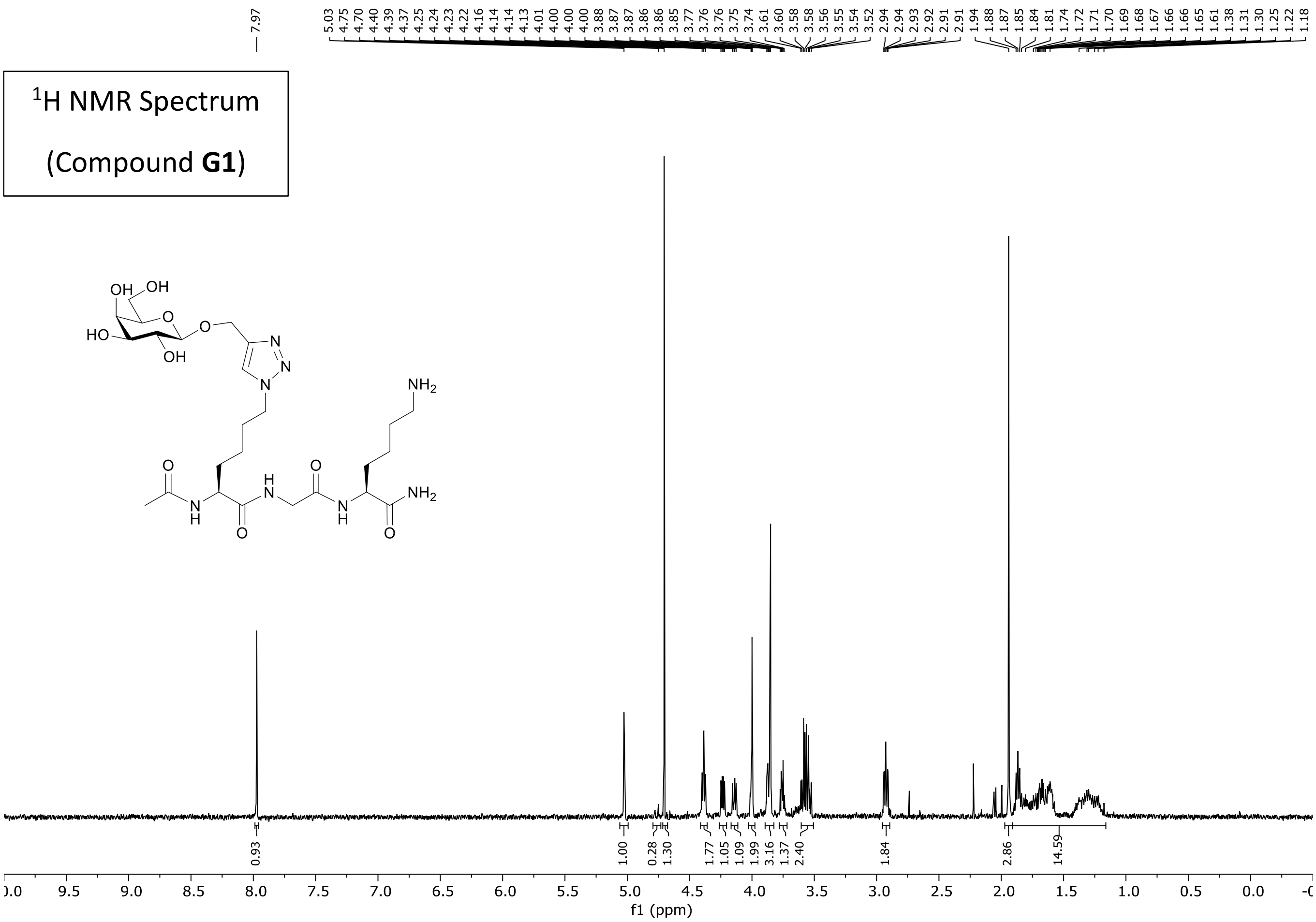

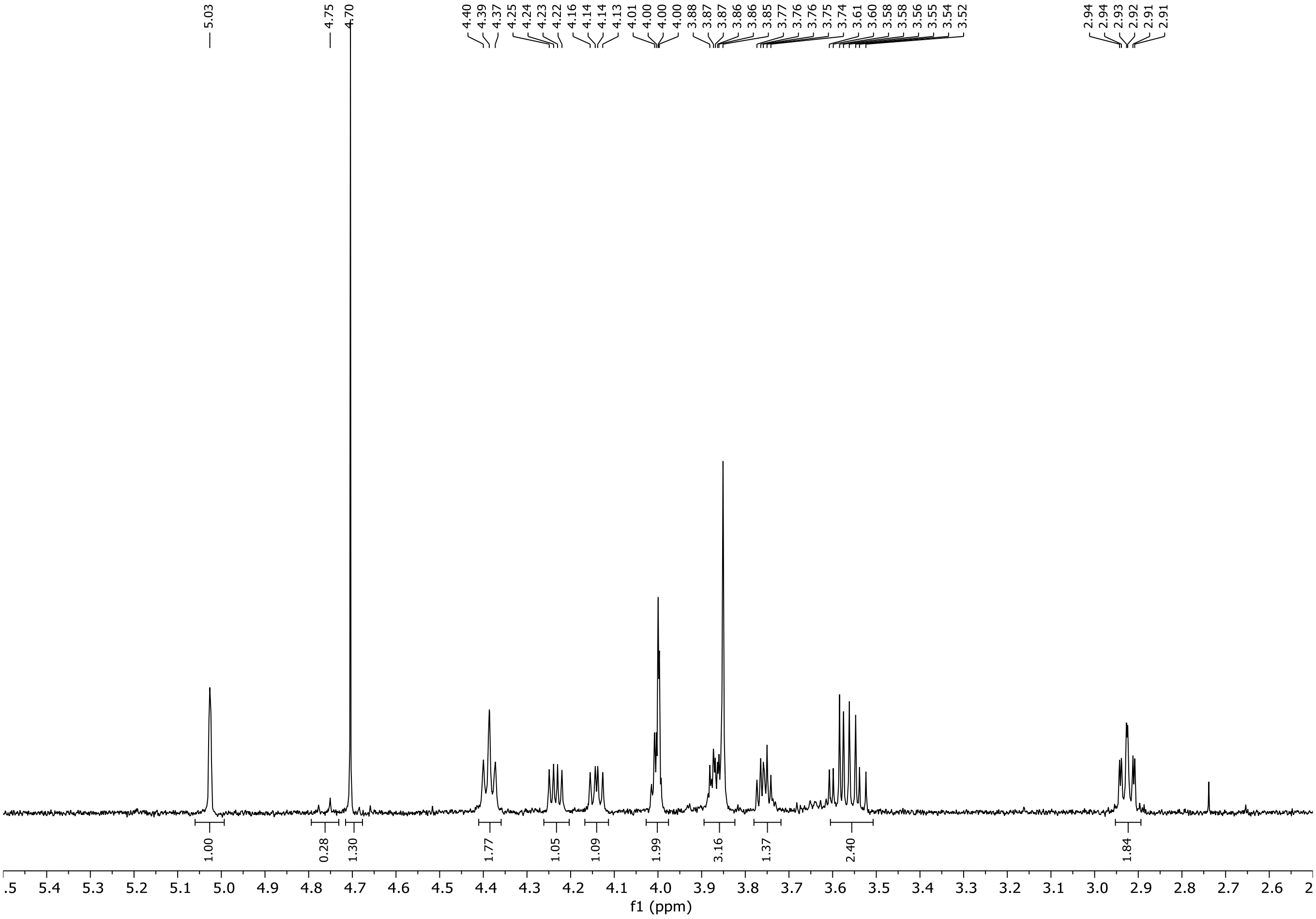

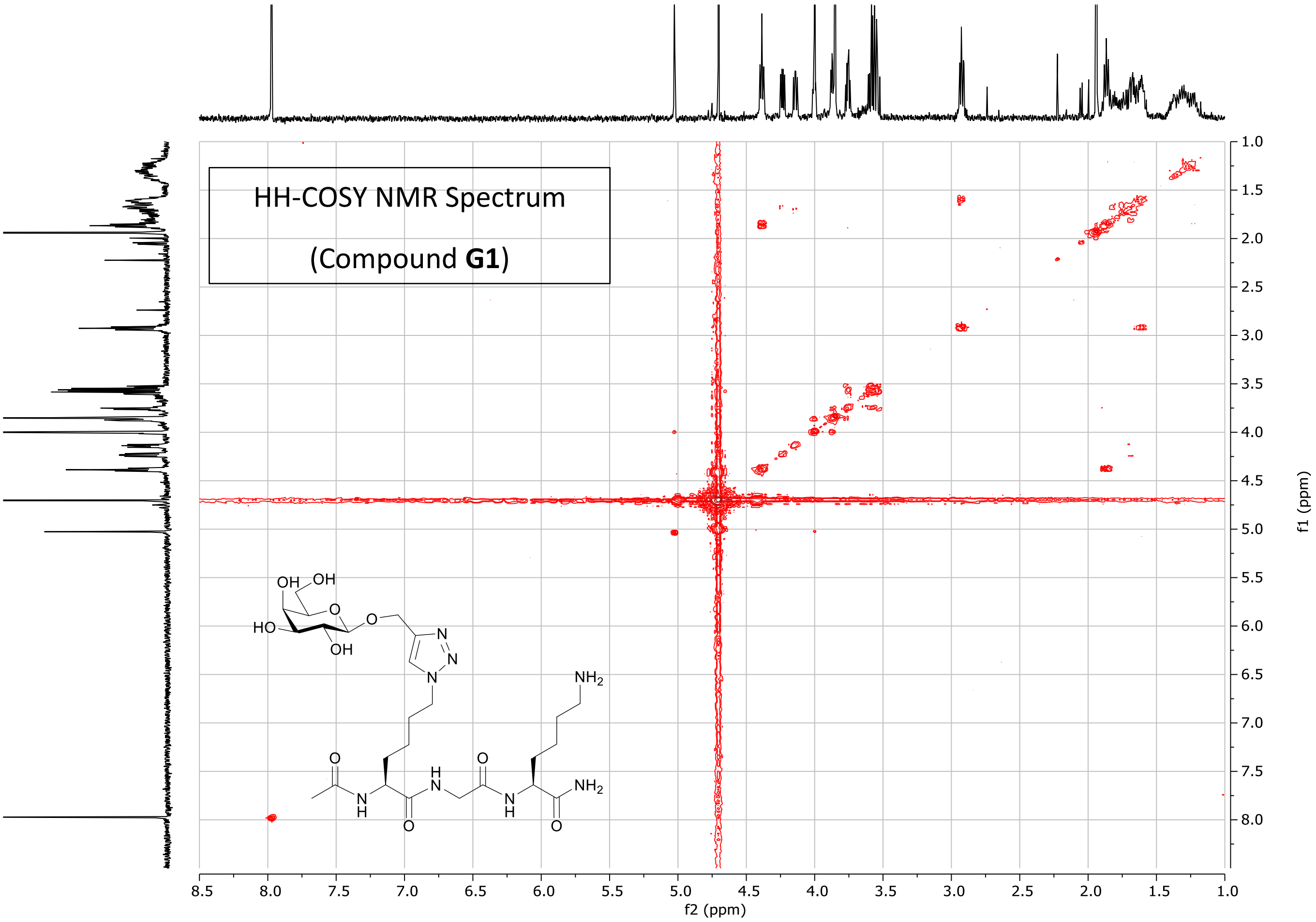
HH-COSY NMR Spectrum
(Compound G1)
f2 (ppm)
f1 (ppm)

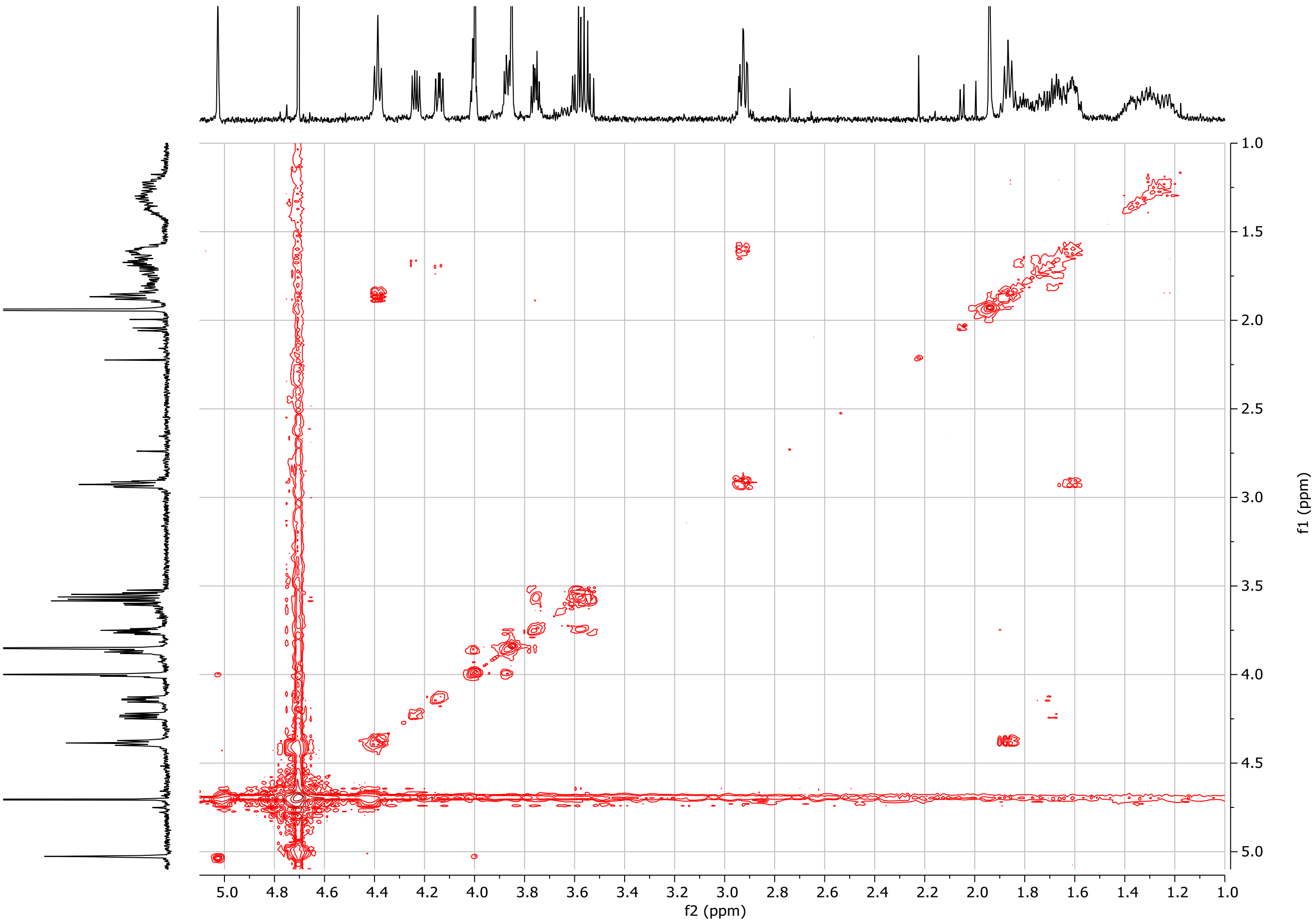
f2 (ppm)
f1 (ppm)

LC-MS Spectra; *(0 –> 50 % ACN, 13 min);* (Compound **G1**)

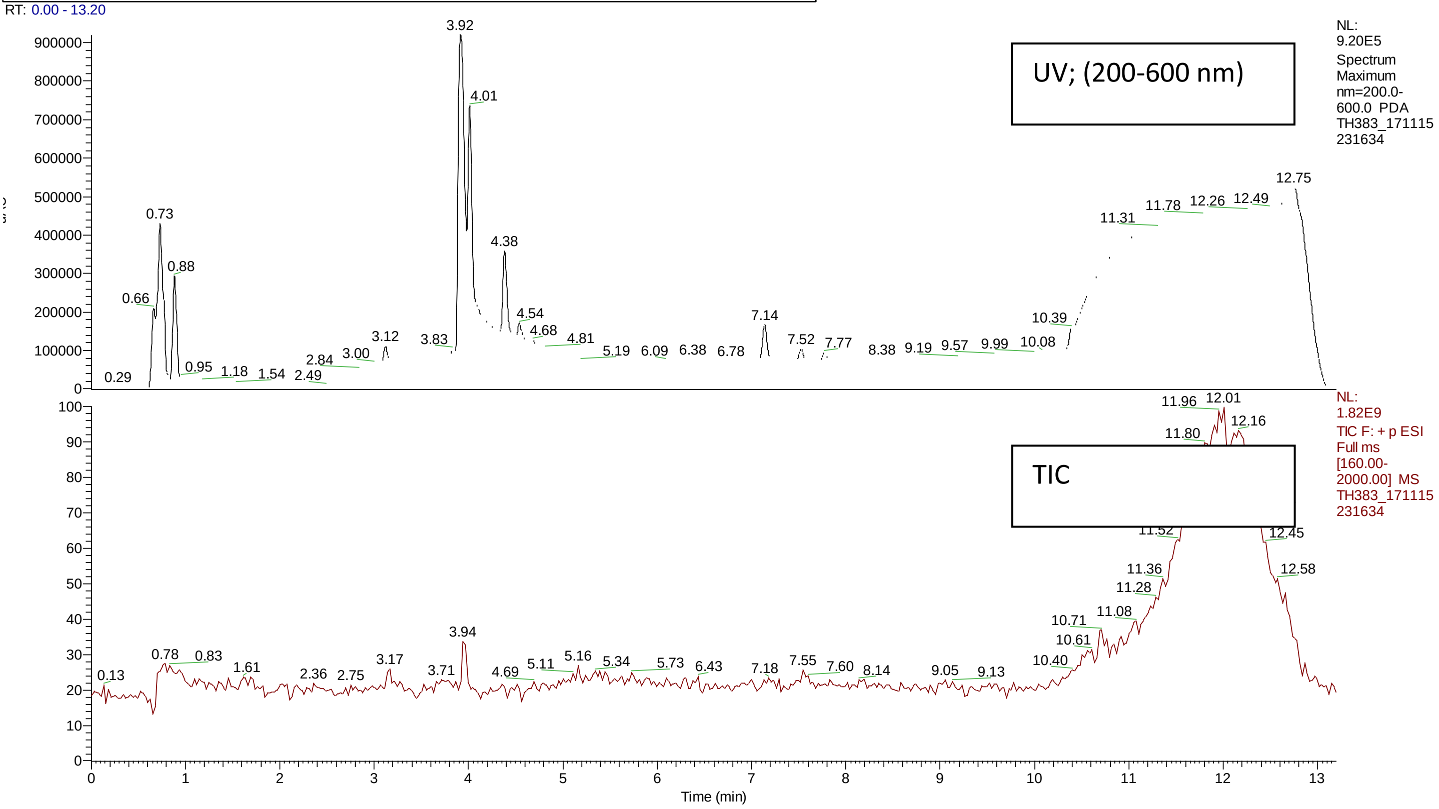

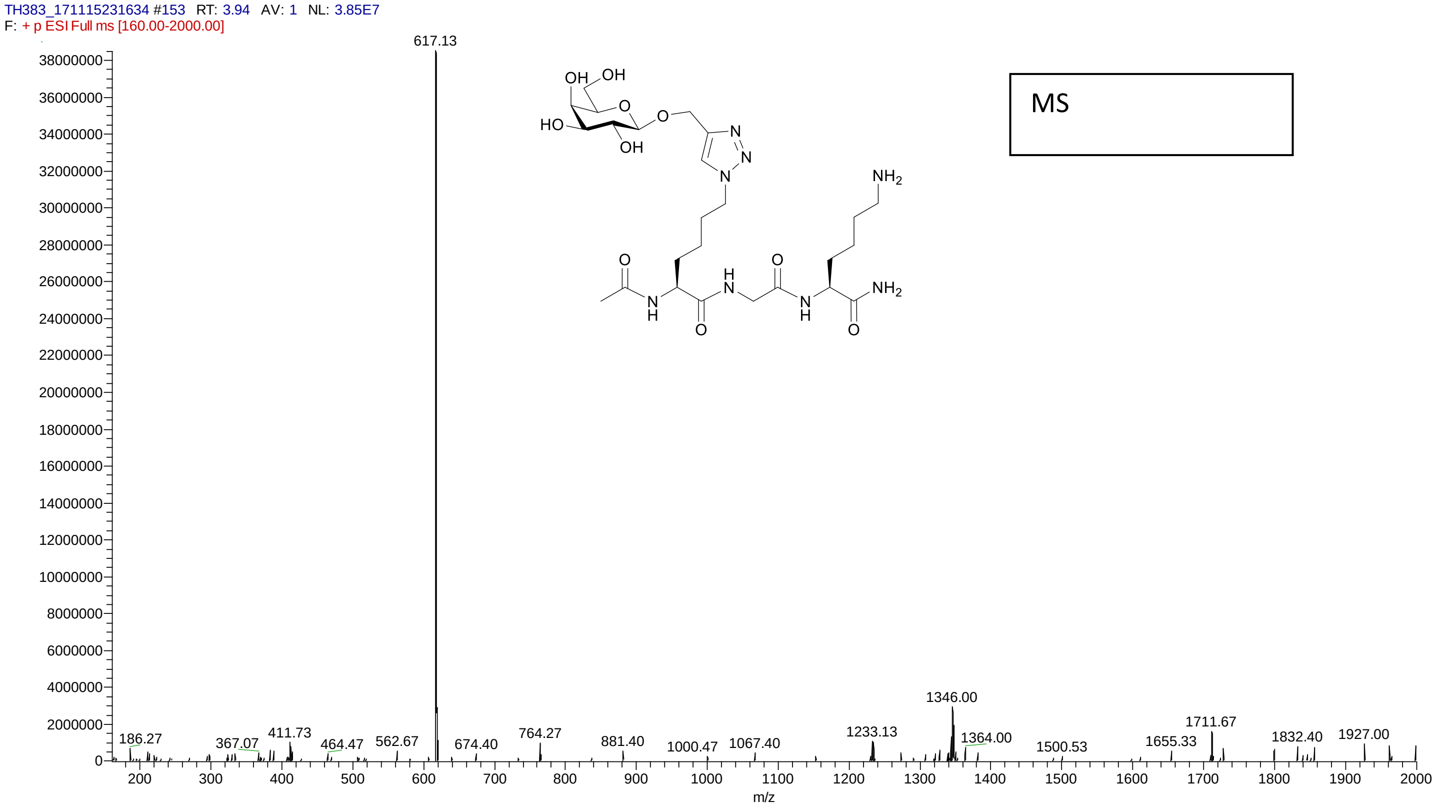

G2

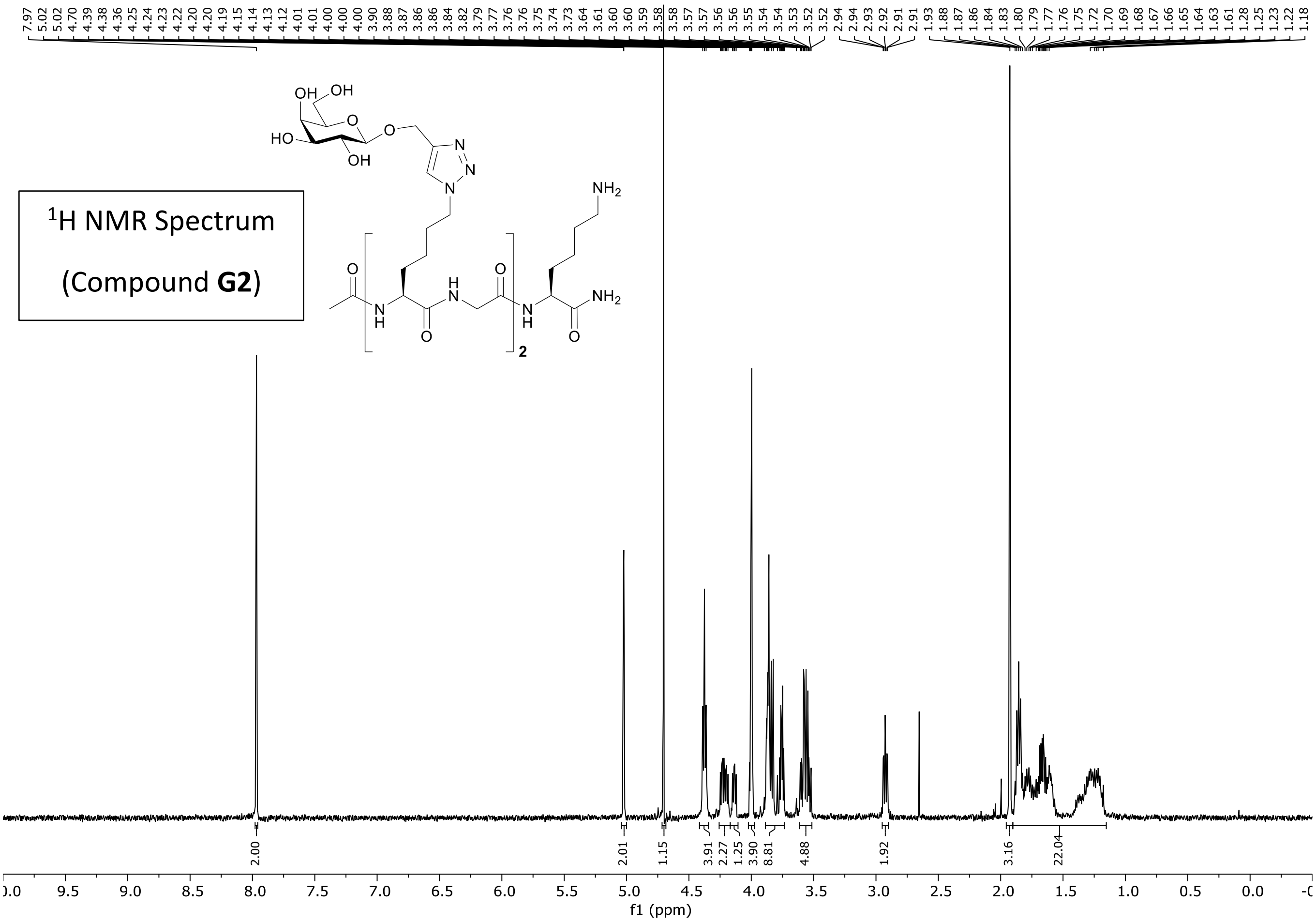

7.97 5.02 4.70 4.39 4.38 4.36 4.25 4.24 4.23 4.22 4.20 4.20 4.19 4.15 4.14 4.13 4.12 4.01 4.01 4.00 4.00 4.00 3.90 3.88 3.87 3.86 3.86 3.84 3.82 3.79 3.77 3.76 3.76 3.75 3.74 3.73 3.64 3.61 3.60 3.60 3.59 3.58 3.58 3.57 3.57 3.56 3.56 3.55 3.54 3.54 3.53 3.52 3.52 2.94 2.94 2.93 2.92 2.91 2.91 1.93 1.88 1.87 1.86 1.84 1.83 1.80 1.79 1.77 1.76 1.75 1.72 1.70 1.69 1.68 1.67 1.66 1.65 1.64 1.63 1.61 1.28 1.25 1.23 1.22 1.18
^{1}H NMR Spectrum
(Compound **G2**)
2.00 2.01 1.15 3.91 2.27 1.25 3.90 8.81 4.88 1.92 3.16 22.04
f1 (ppm)

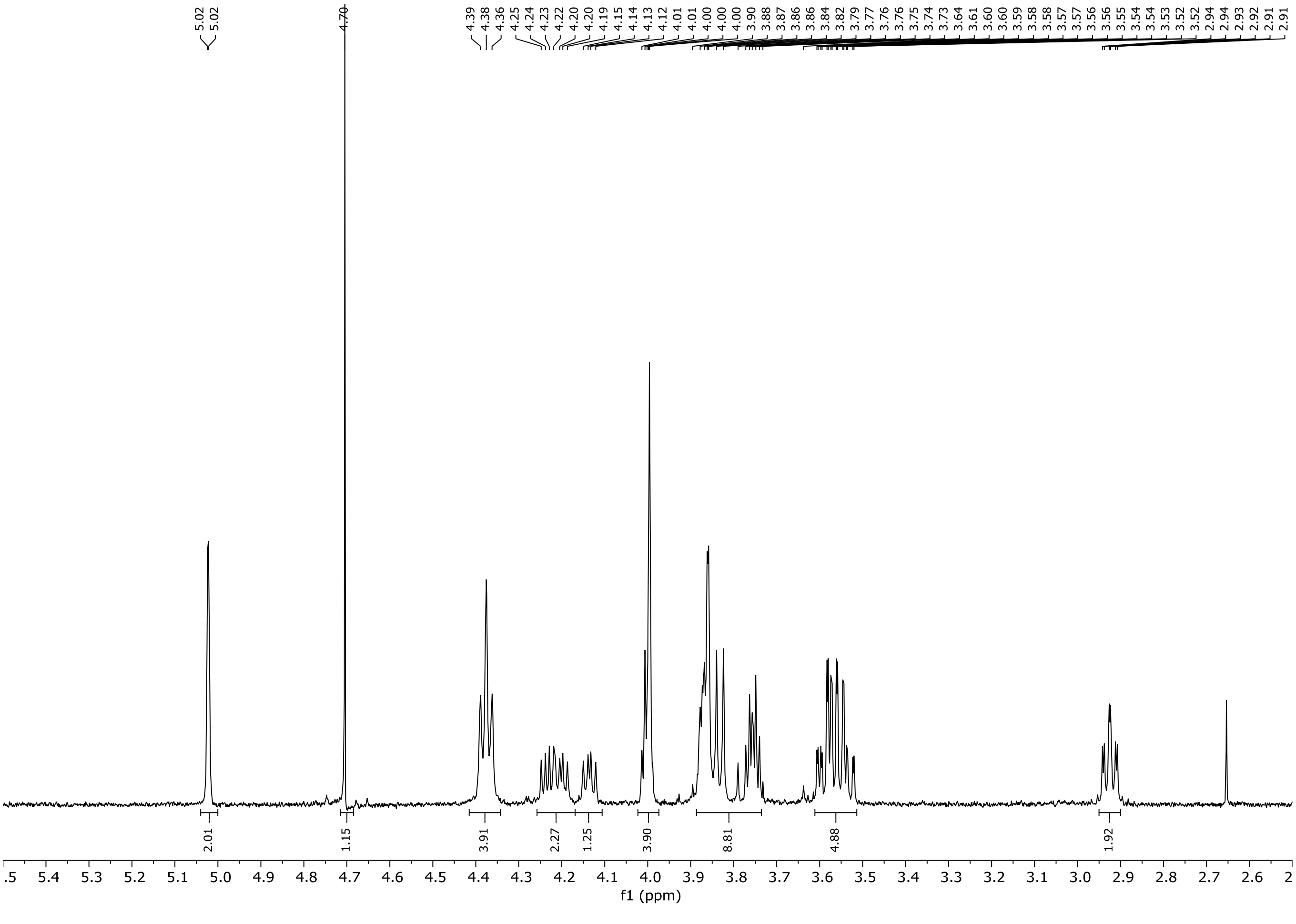

2.01 1.15 3.91 2.27 1.25 3.90 8.81 4.88 1.92
f1 (ppm)

LC-MS Spectra; *(0 –> 50 % ACN, 13 min);* (Compound **G2**)

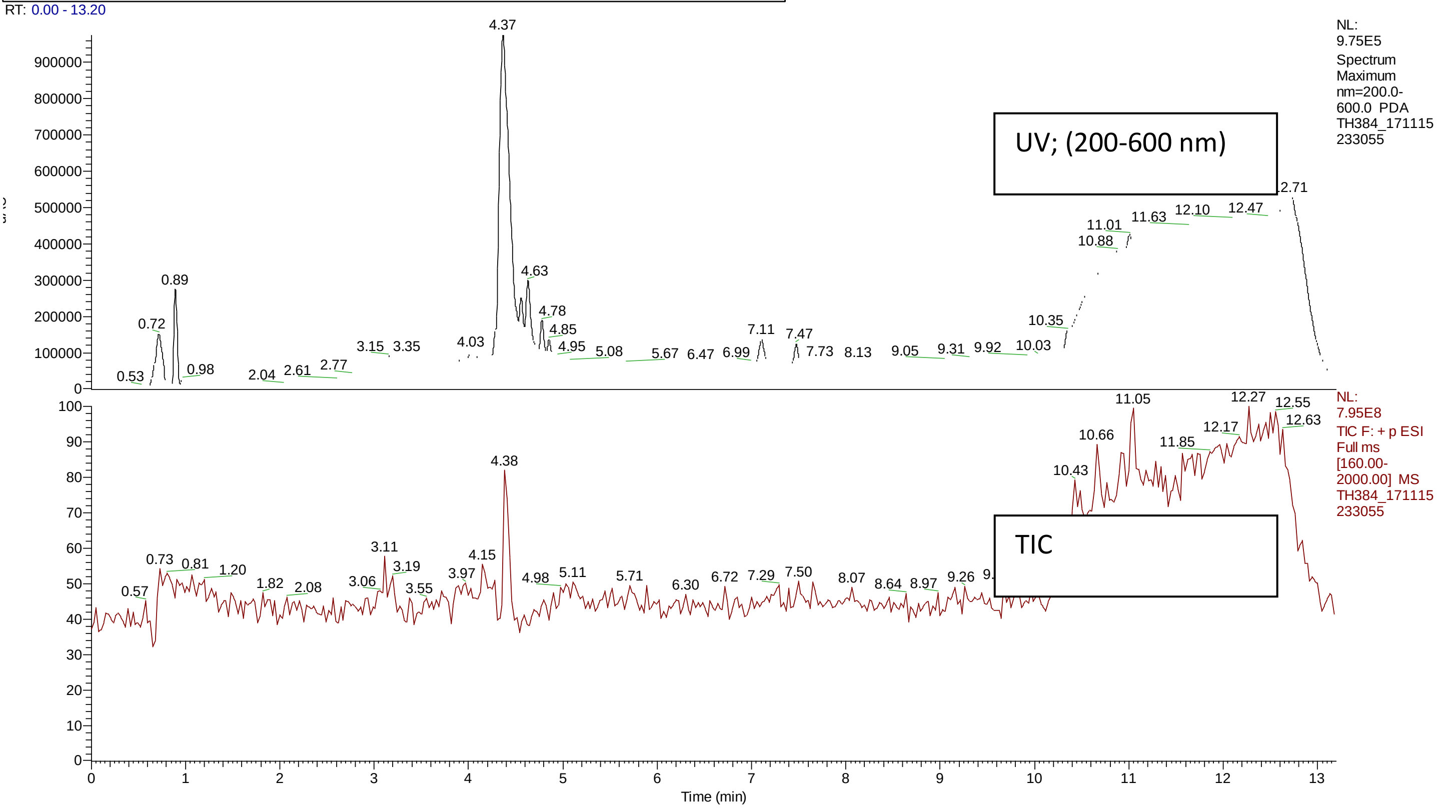

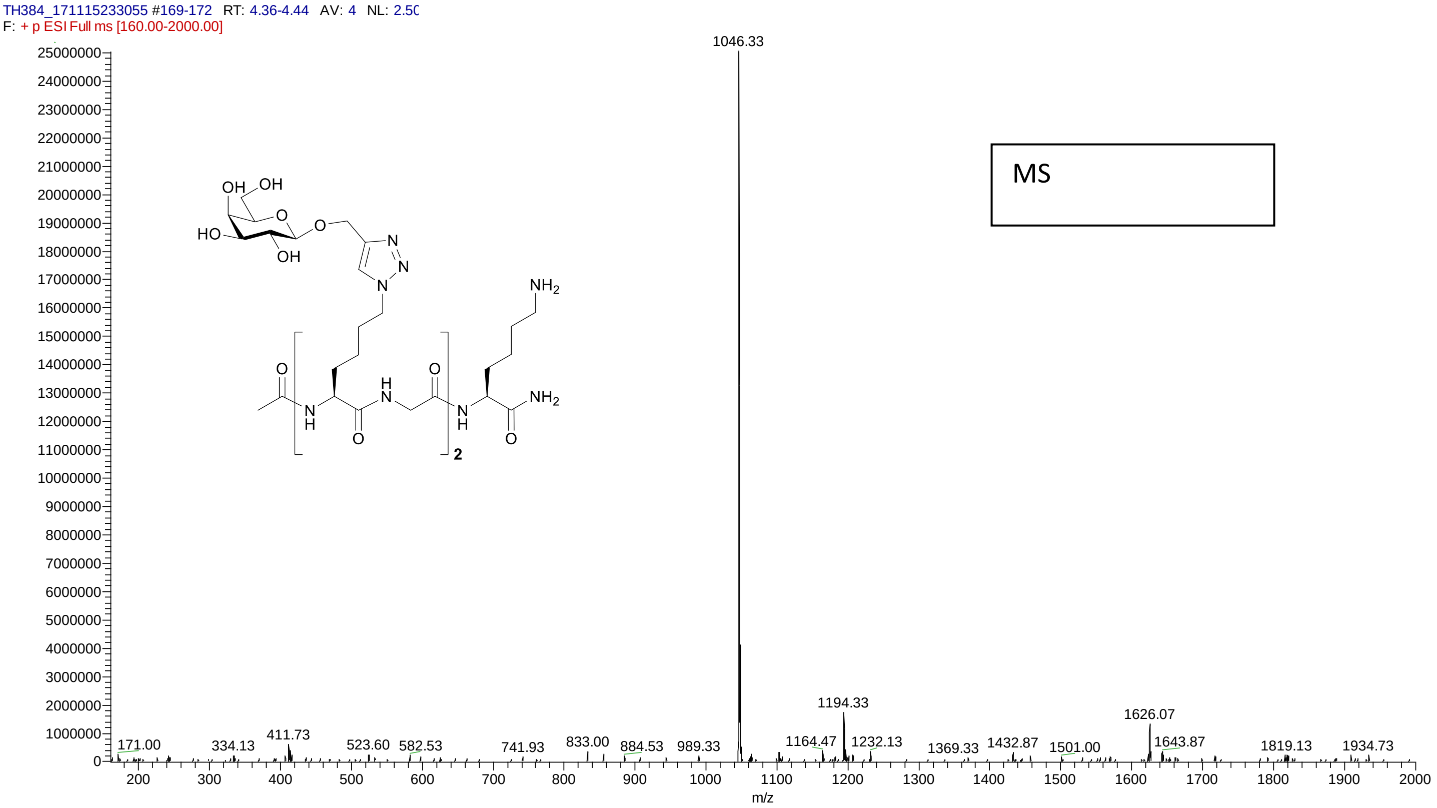

Spectra of CLR Ligands, biotin functionalised

Compound: a1

LC-MS Spectra; *(0 –> 50 % ACN, 13 min)*

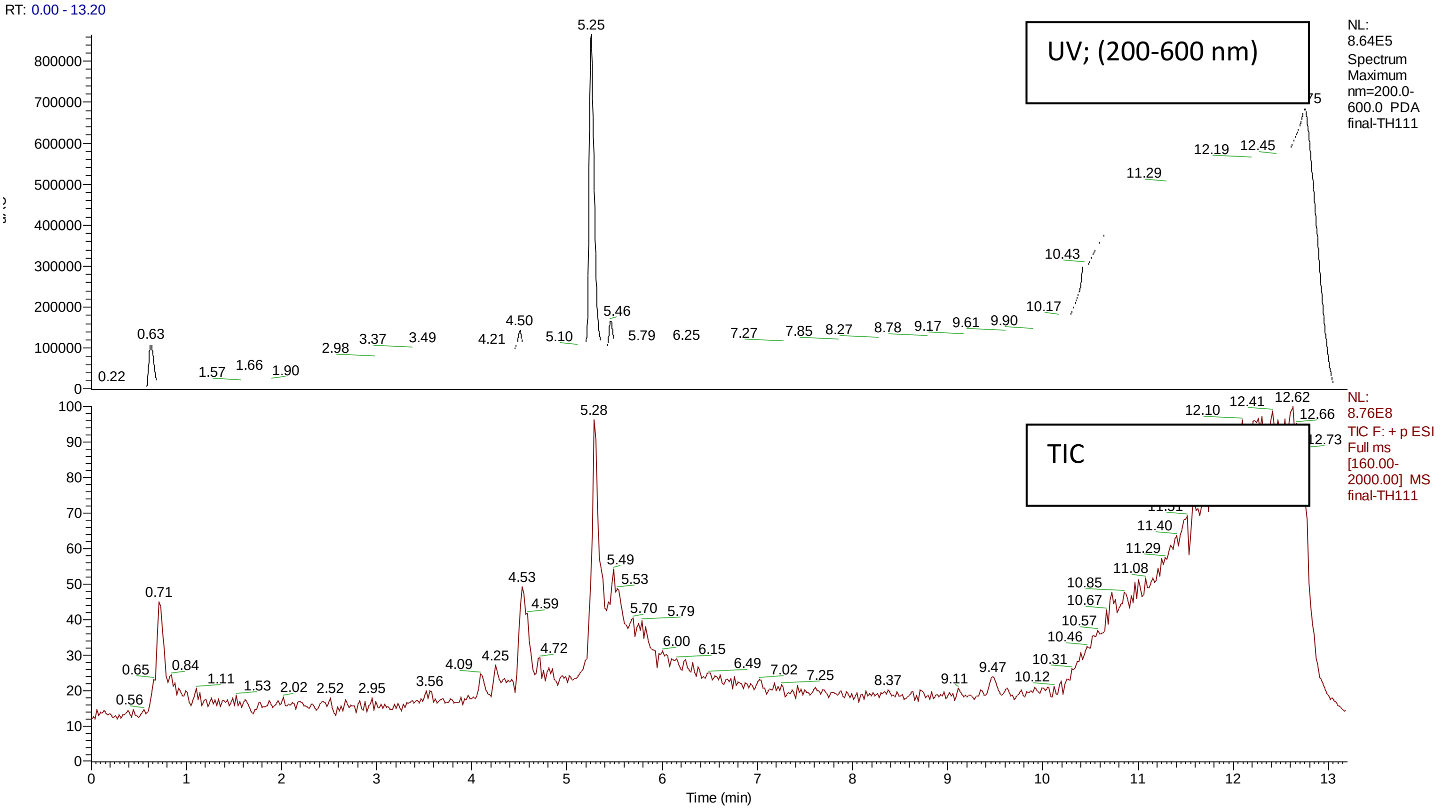

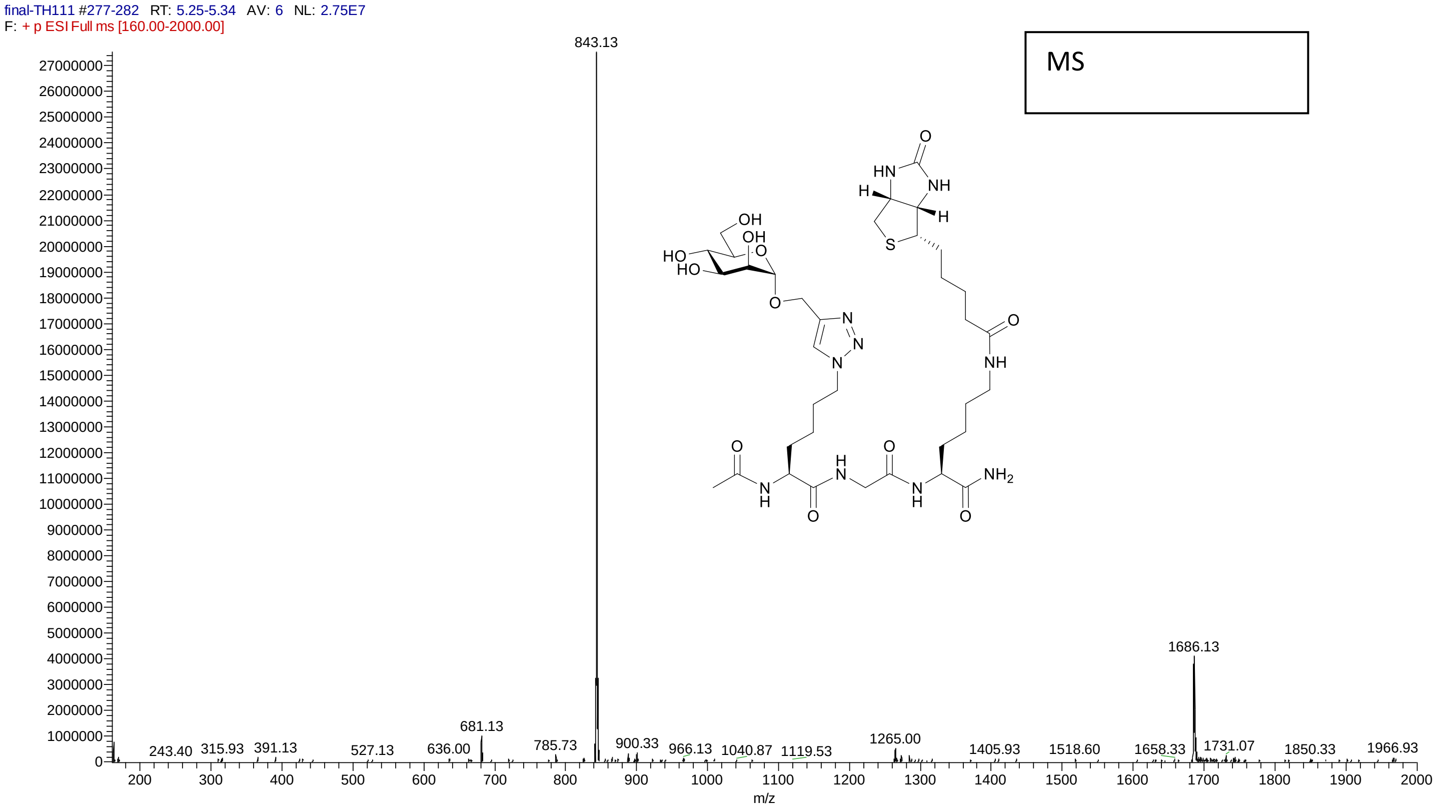

Compound a2

LC-MS Spectra; *(0 –> 50 % ACN, 13 min)*

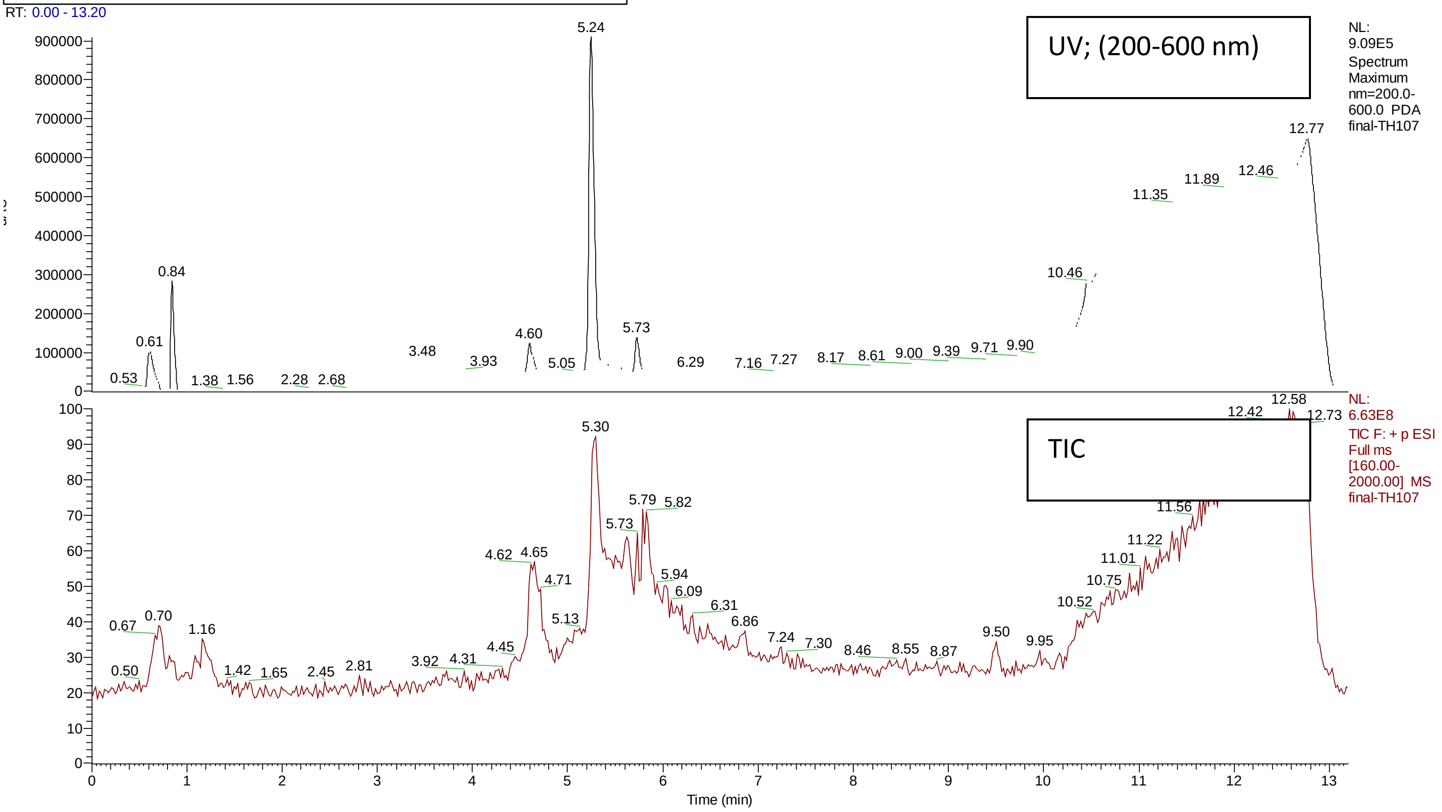

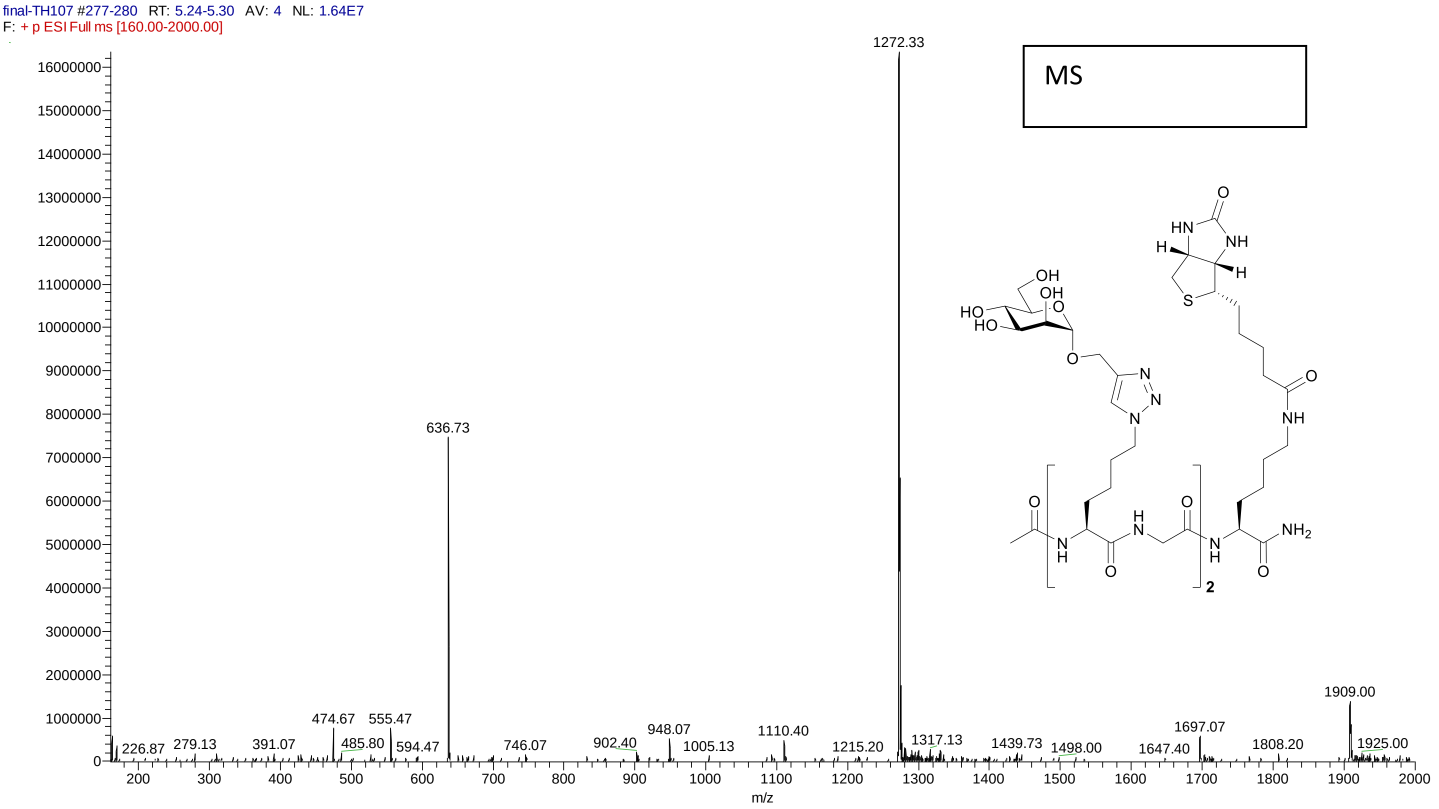

Compound a3

LC-MS Spectra; *(0 –> 50 % ACN, 13 min)*

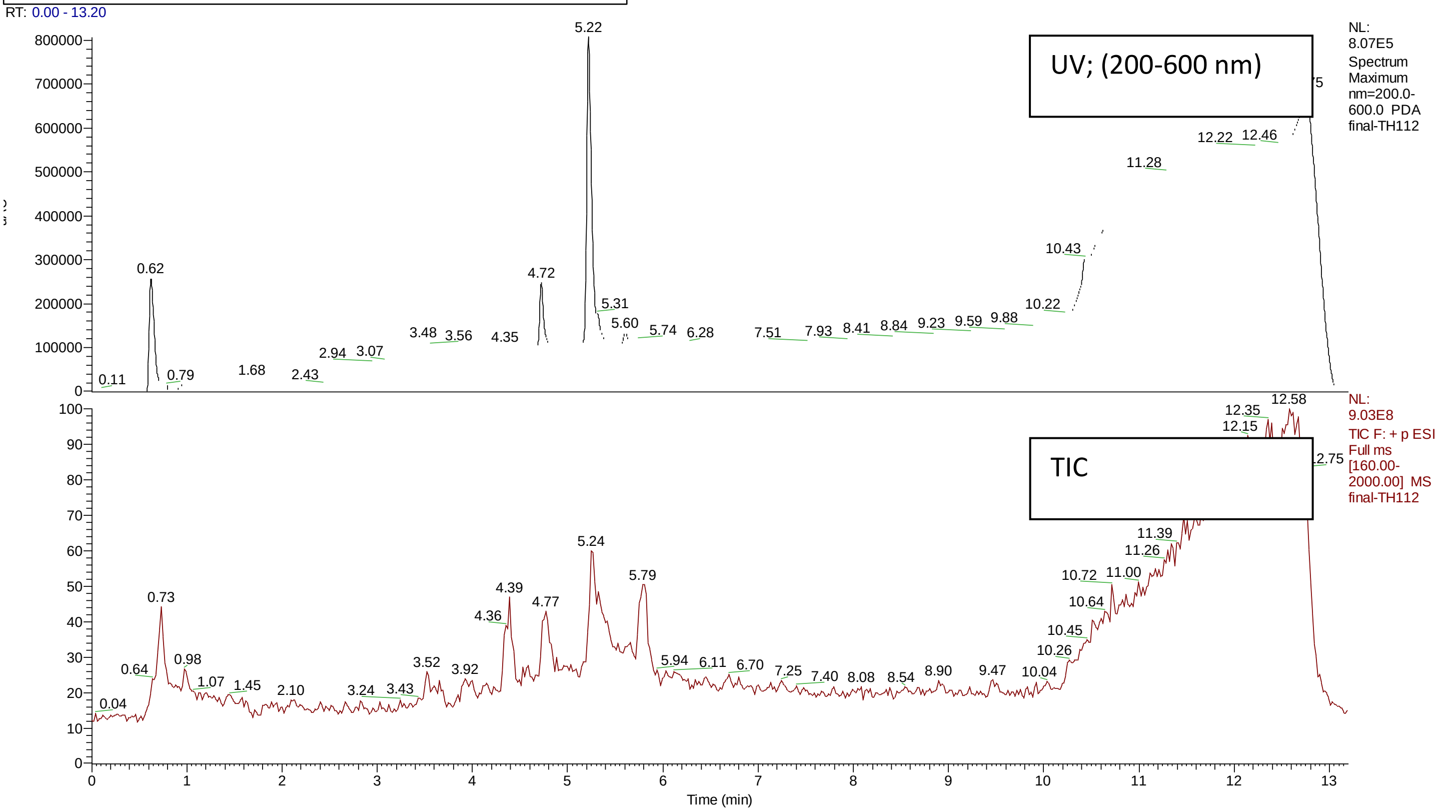

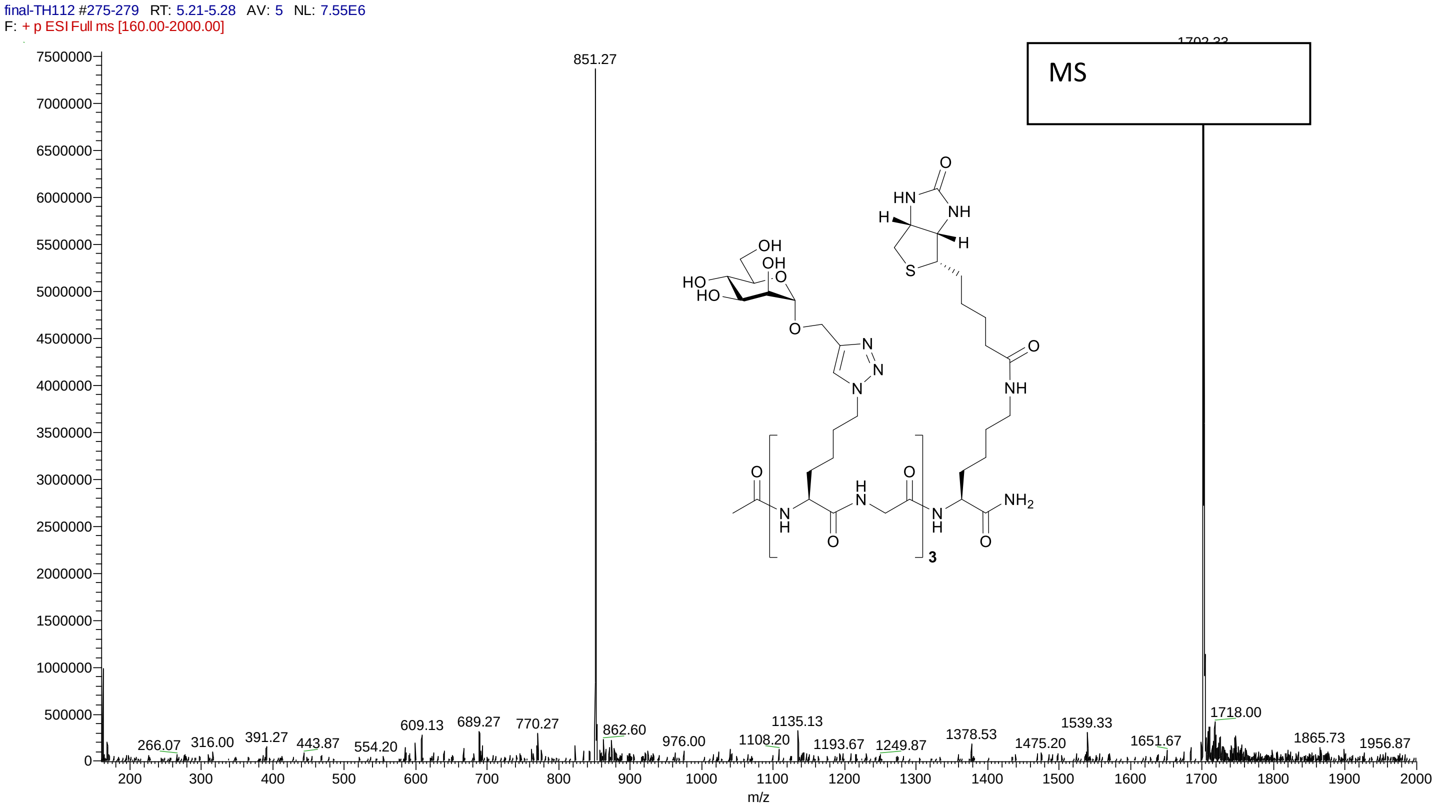

Compound a6

LC-MS Spectra; *(0 –> 50 % ACN, 13 min)*

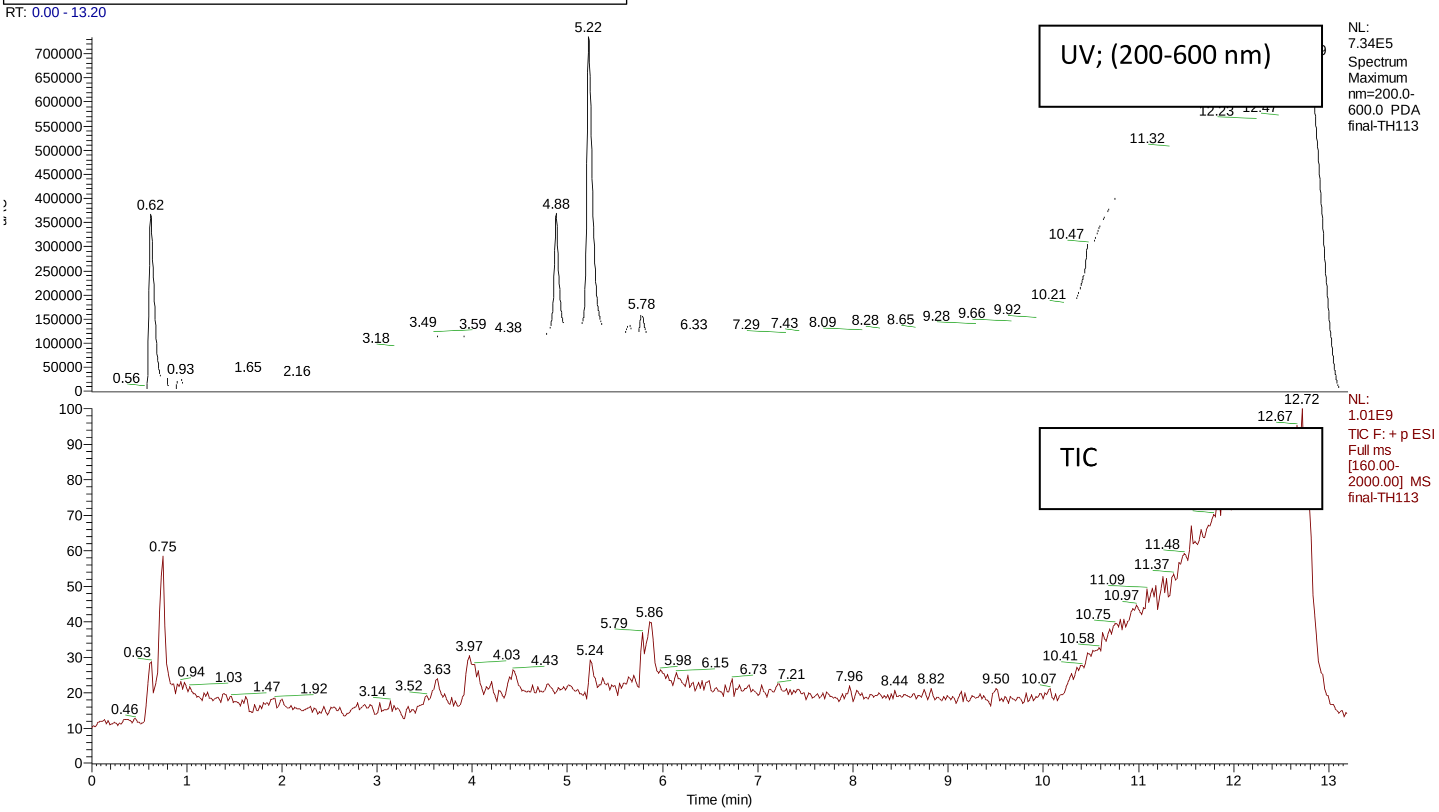

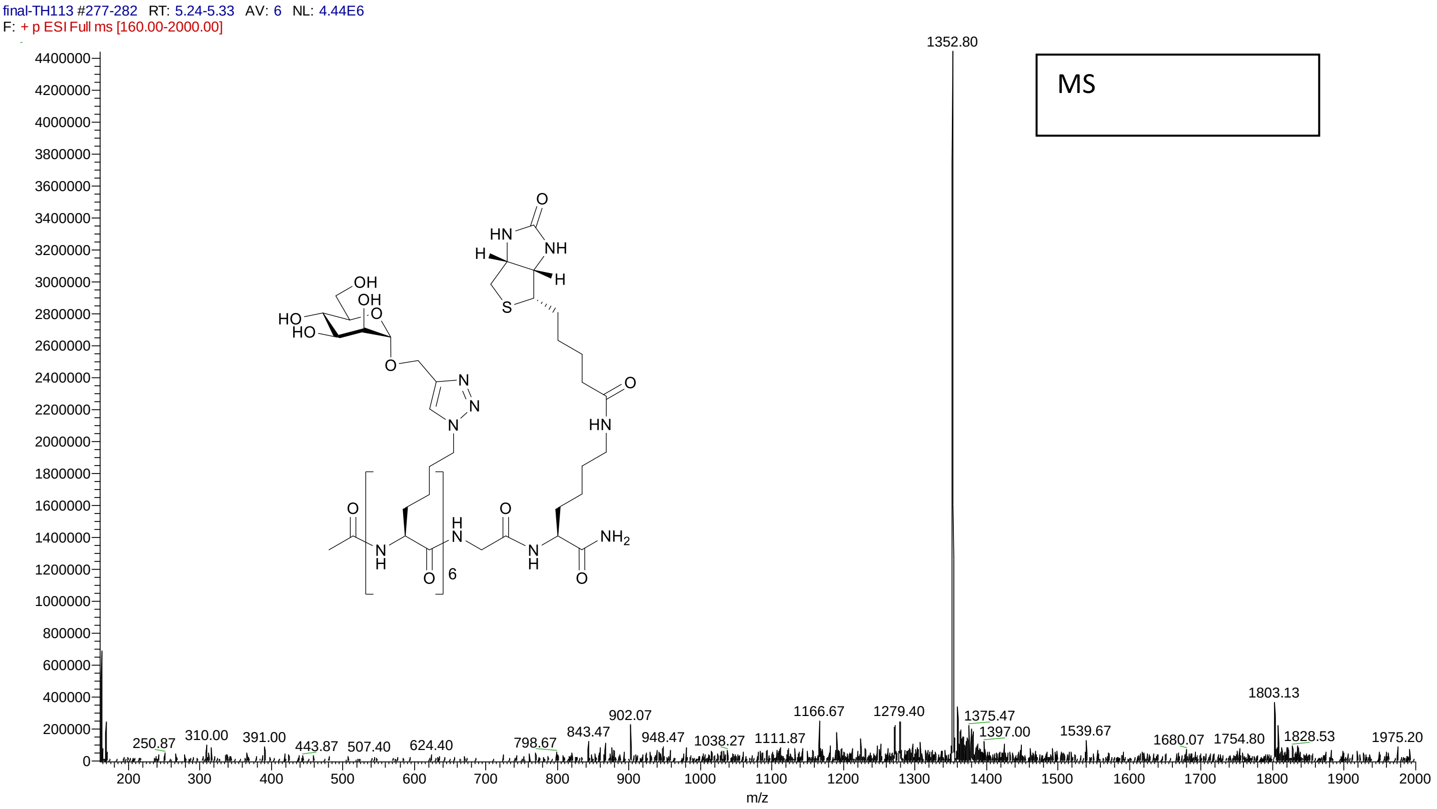

Compound b1

LC-MS Spectra; *(0 –> 50 % ACN, 13 min)*

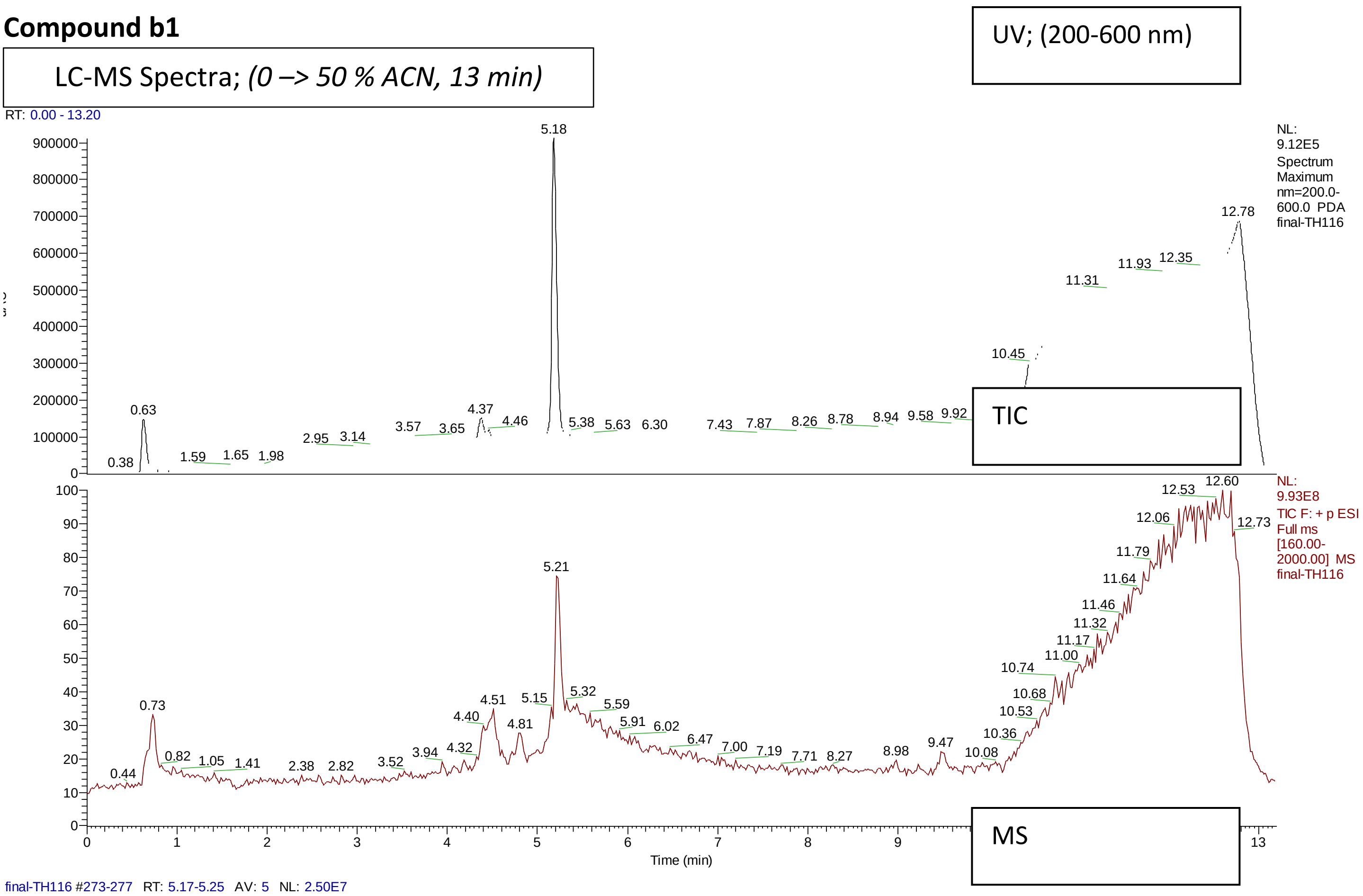

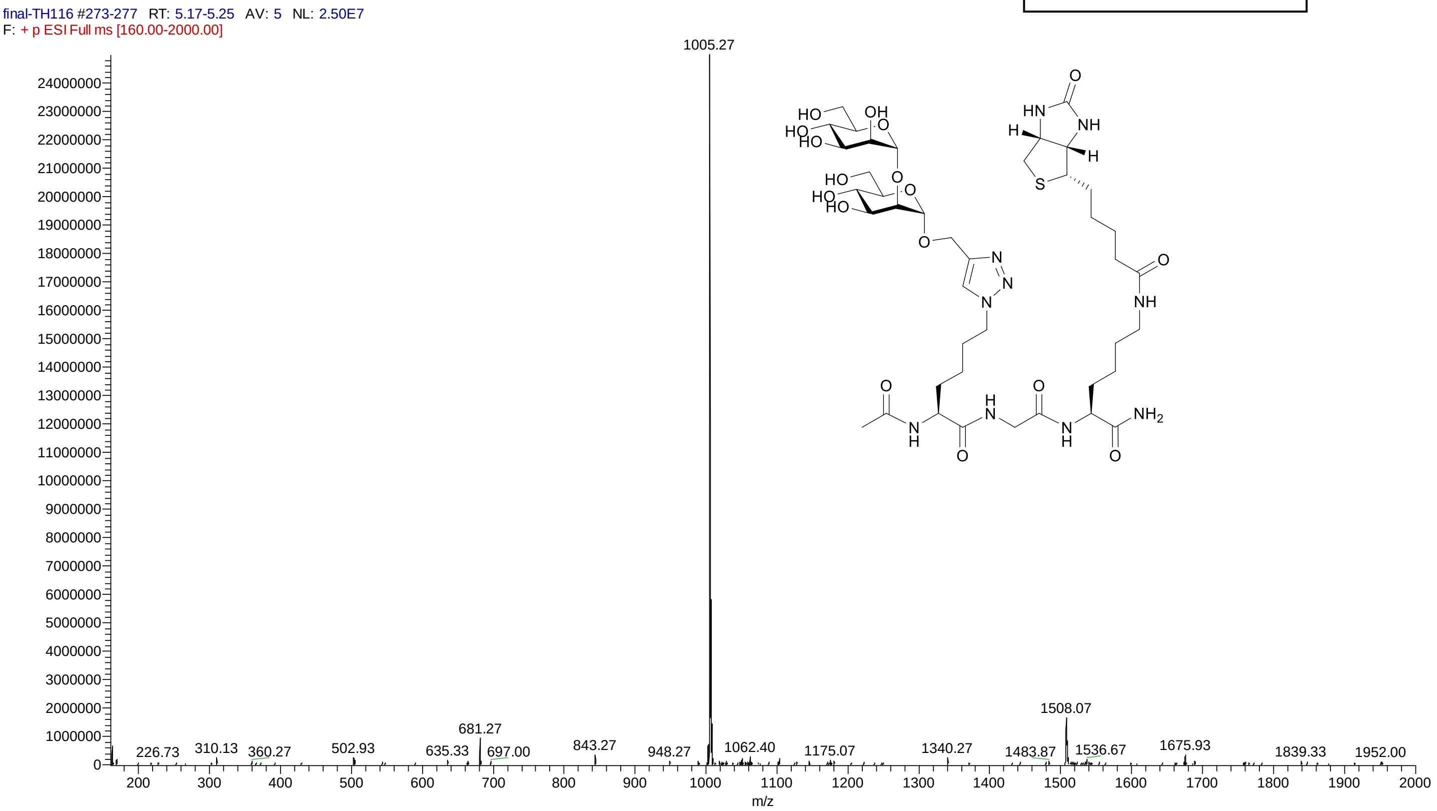

Compound b2

LC-MS Spectra; *(0 –> 50 % ACN, 13 min)*

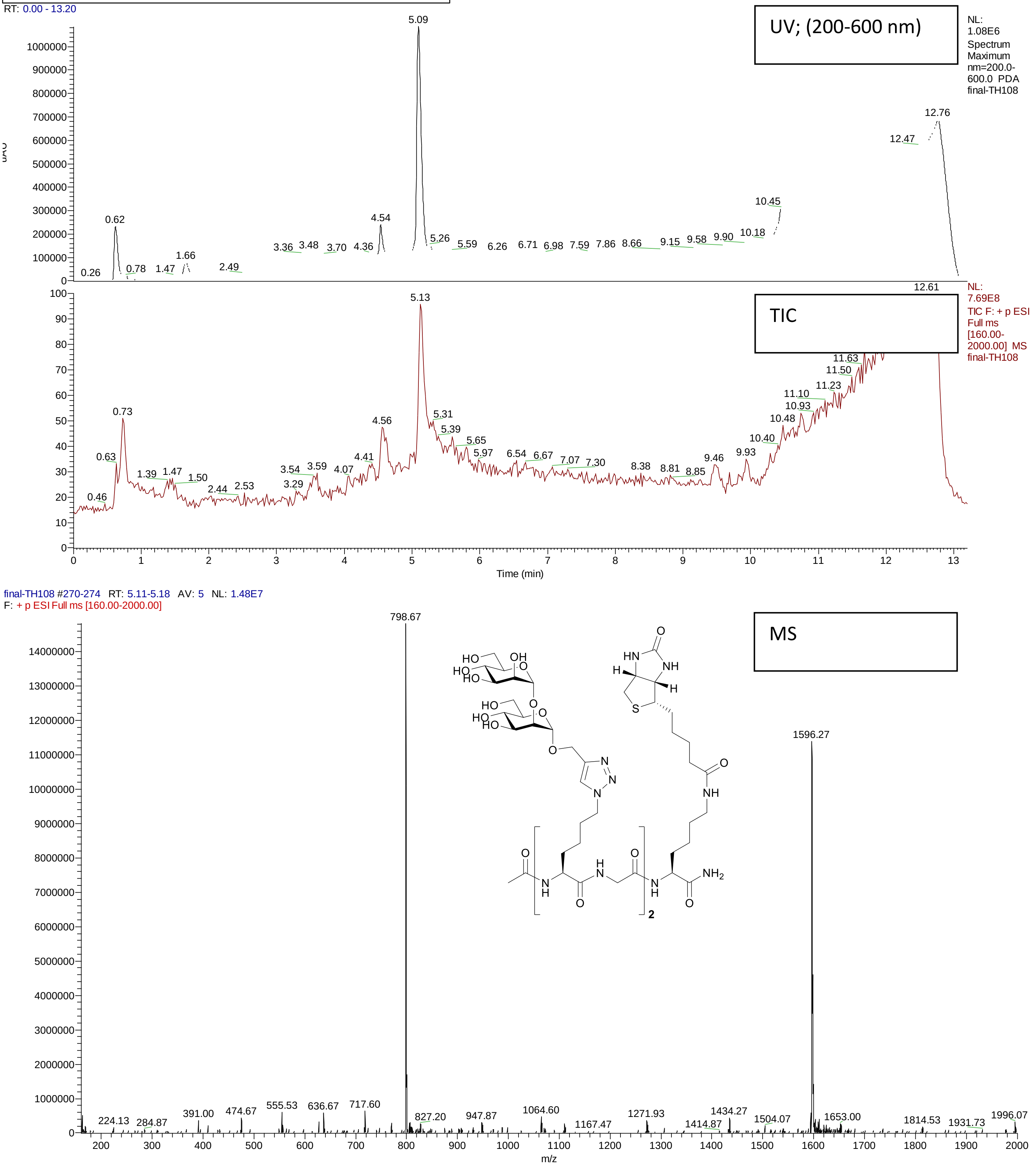

Compound b3

LC-MS Spectra; *(0 –> 50 % ACN, 13 min)*

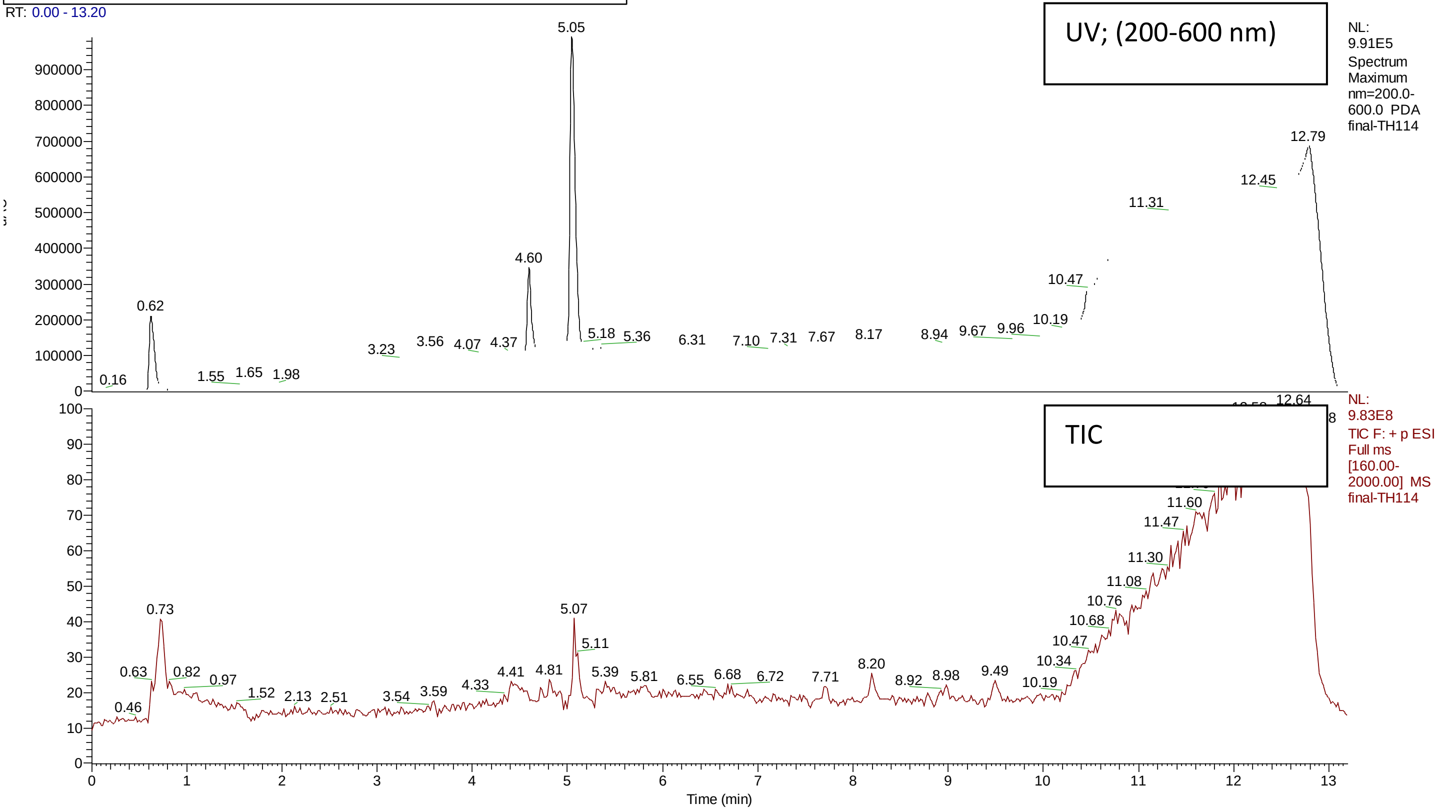

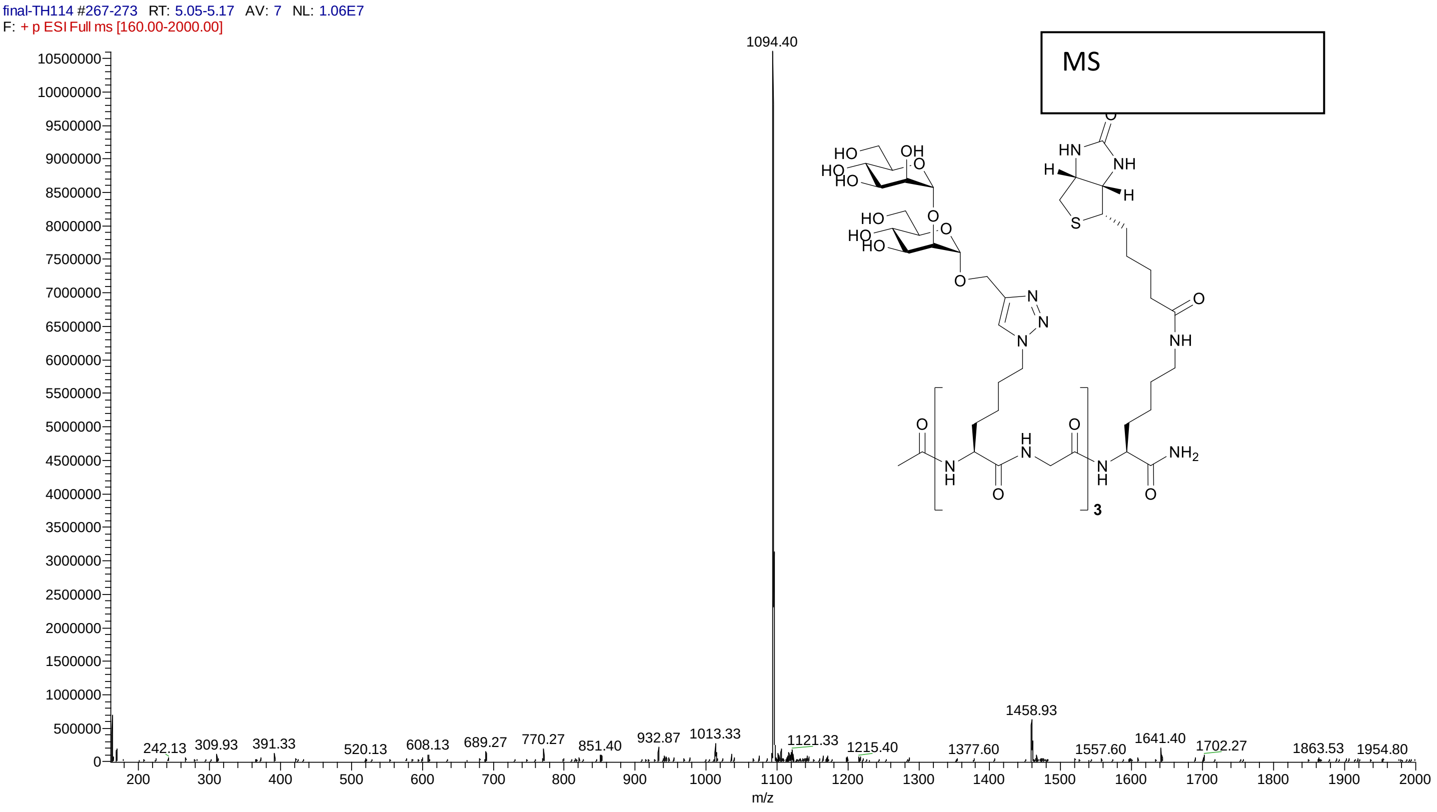

Compound b6

LC-MS Spectra; *(0 –> 50 % ACN, 13 min)*

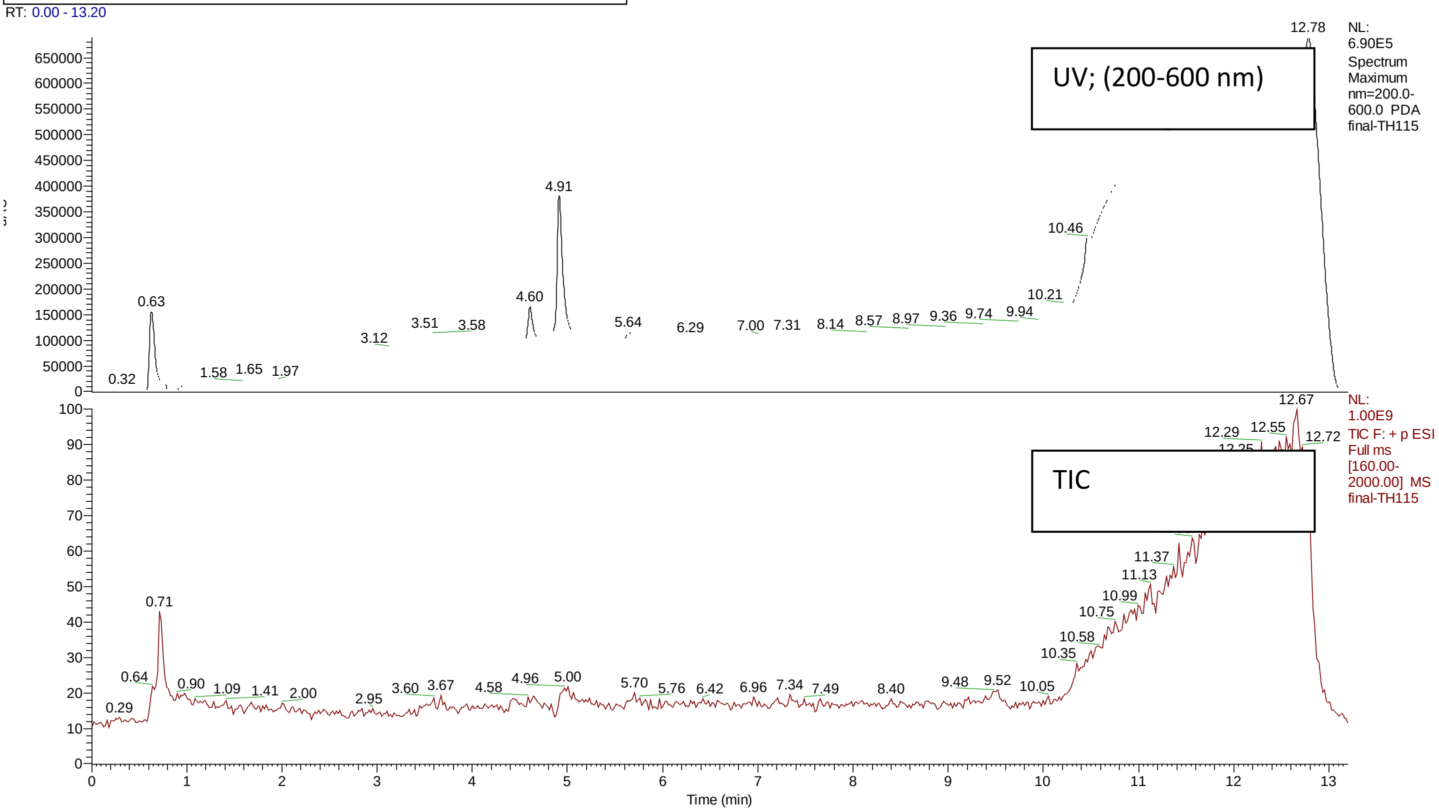

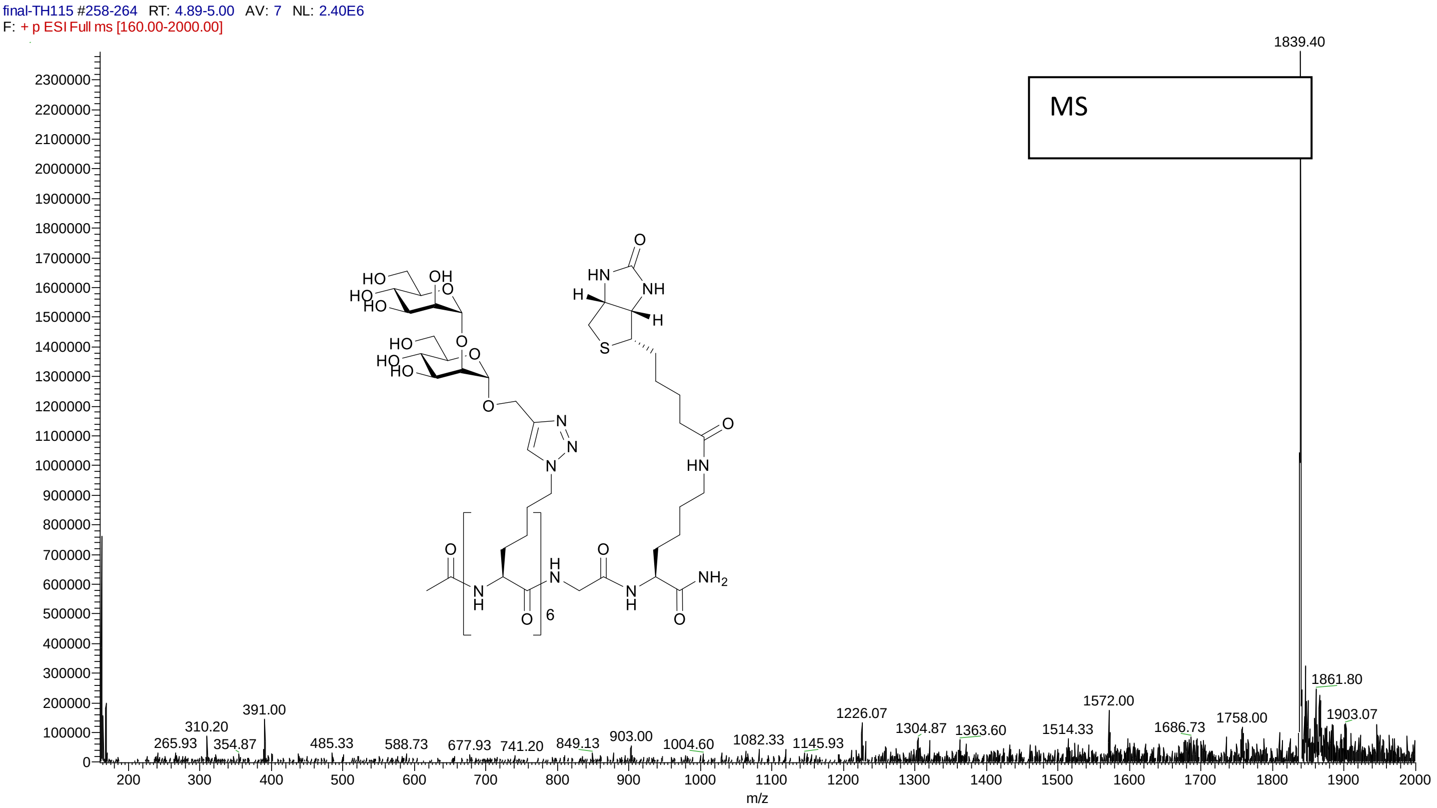

Compound c1

LC-MS Spectra; *(0 –> 50 % ACN, 13 min)*

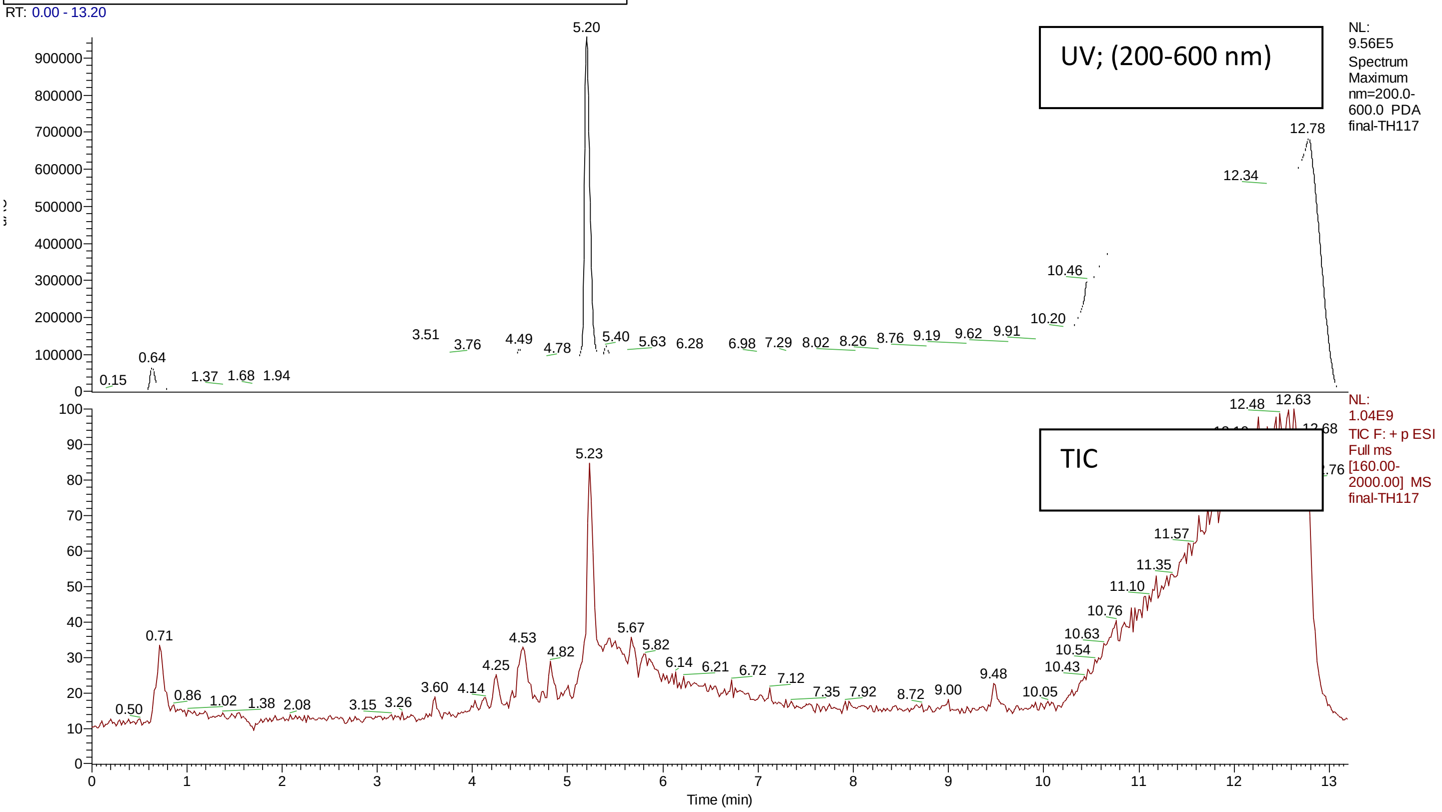

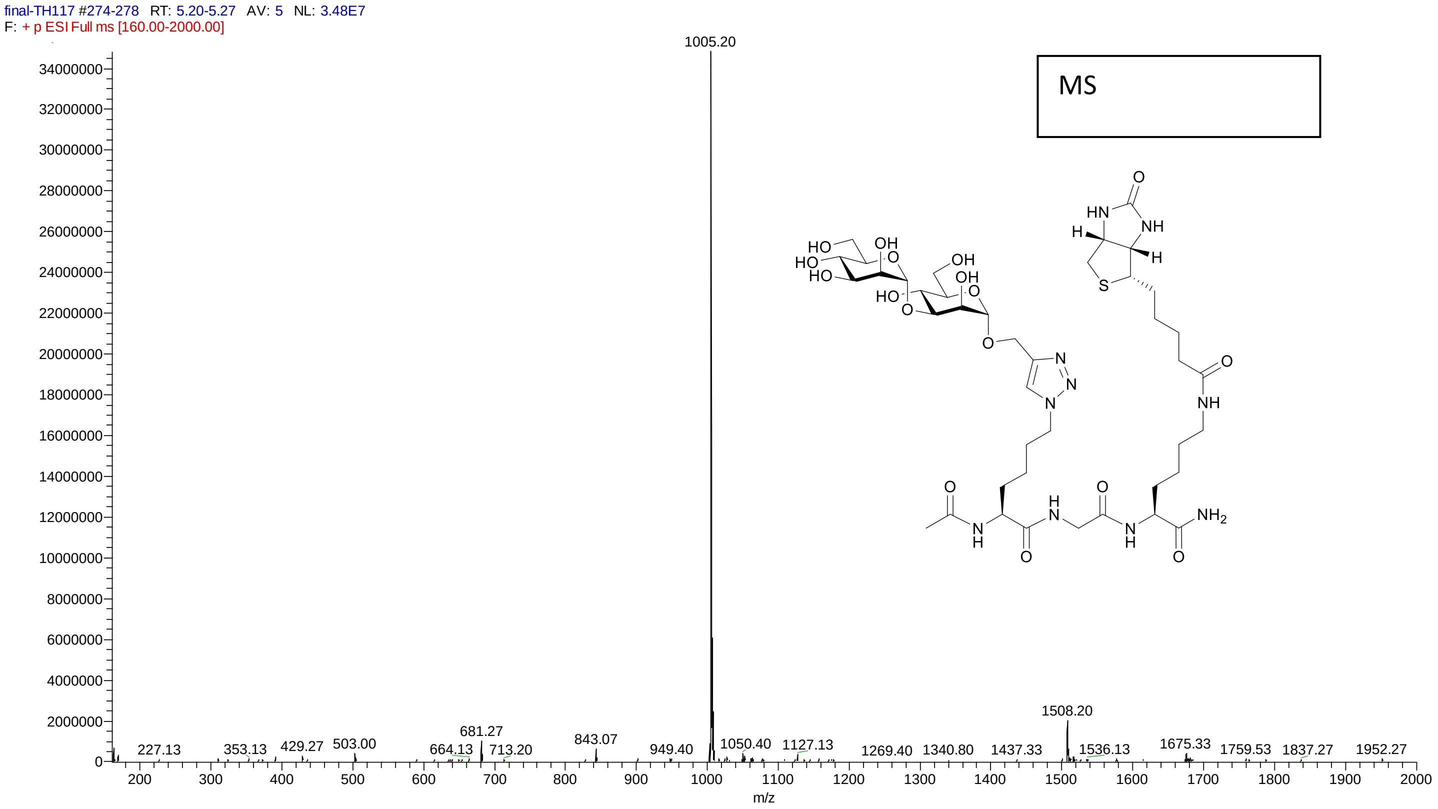

Compound c2

LC-MS Spectra; *(0 –> 50 % ACN, 13 min)*

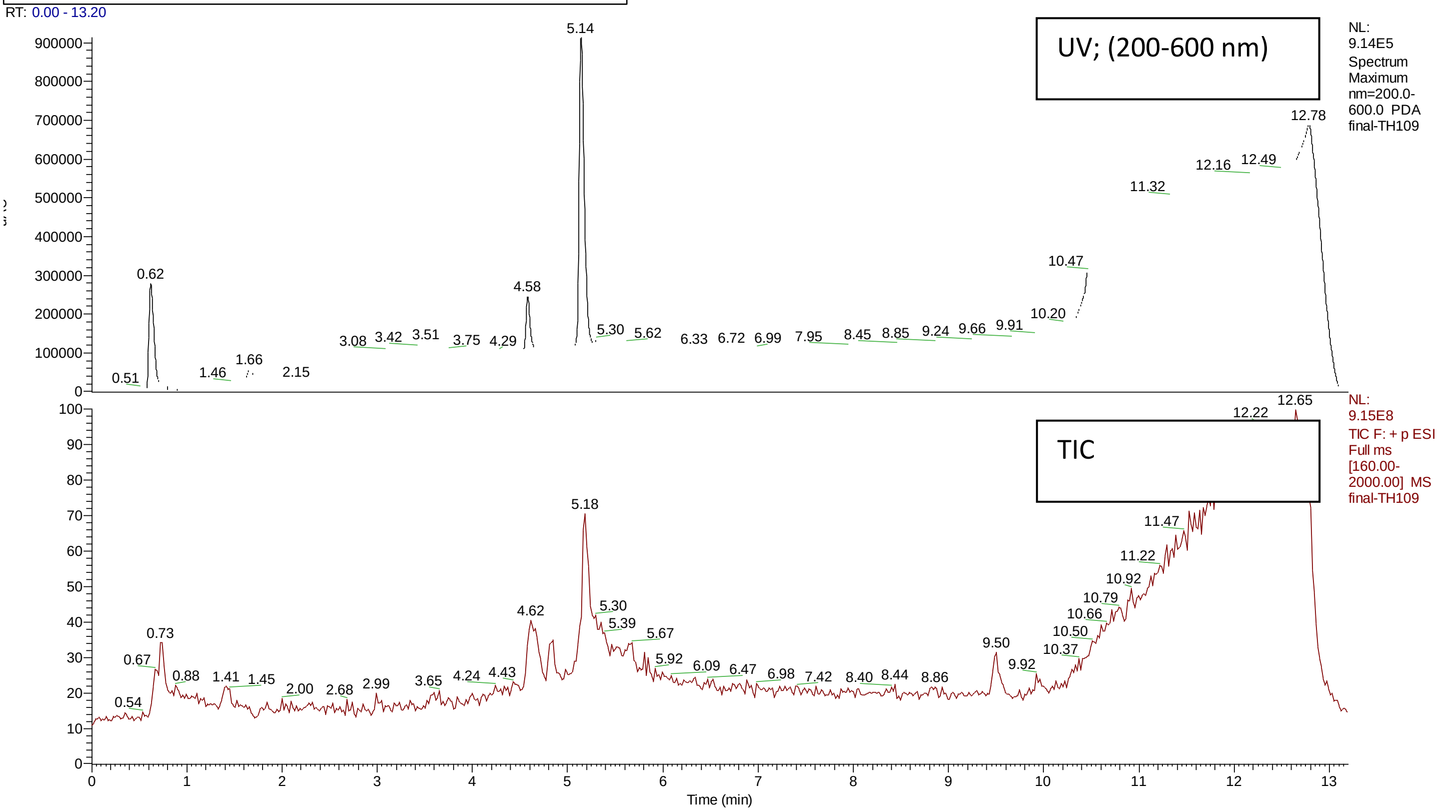

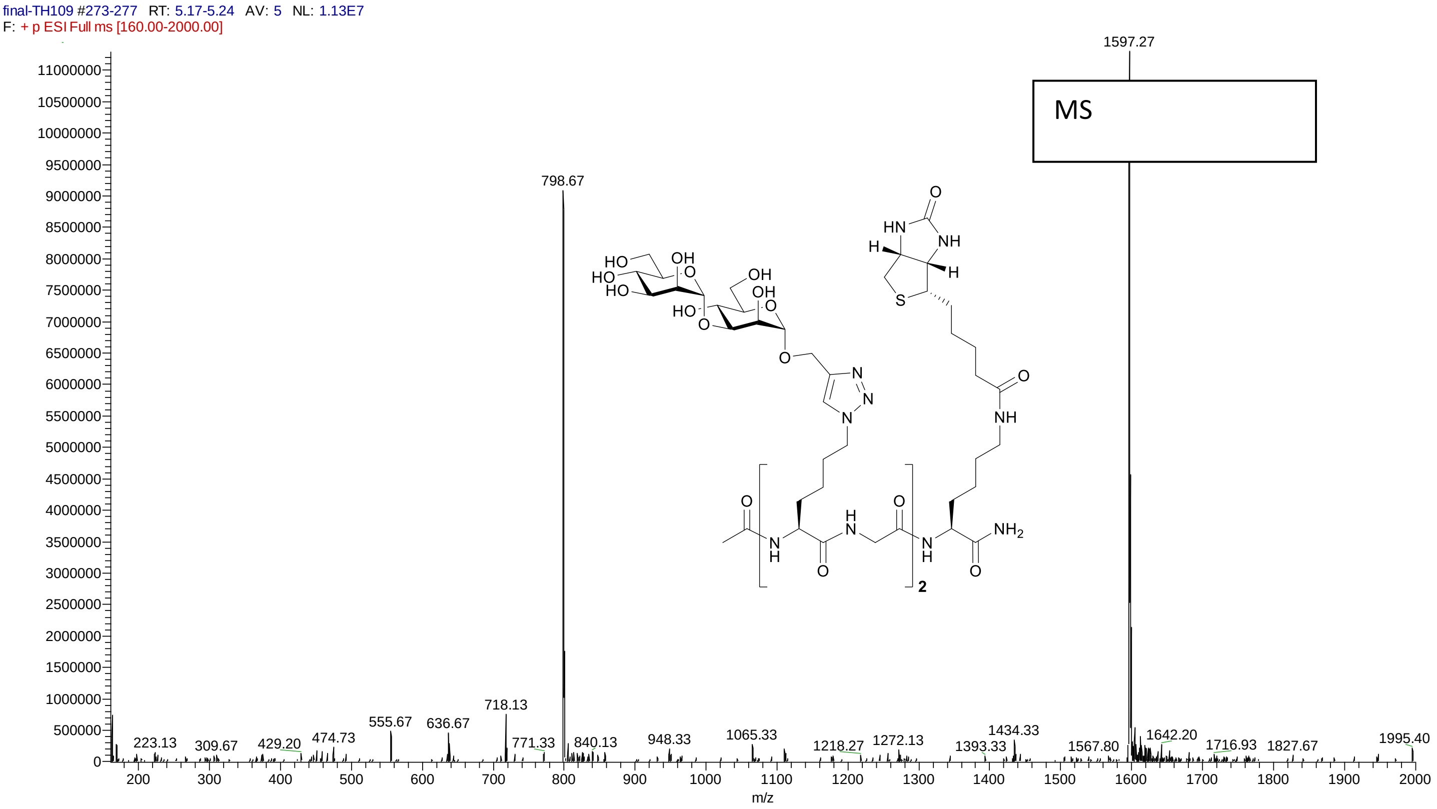

Compound c3

LC-MS Spectra; *(0 –> 50 % ACN, 13 min)*

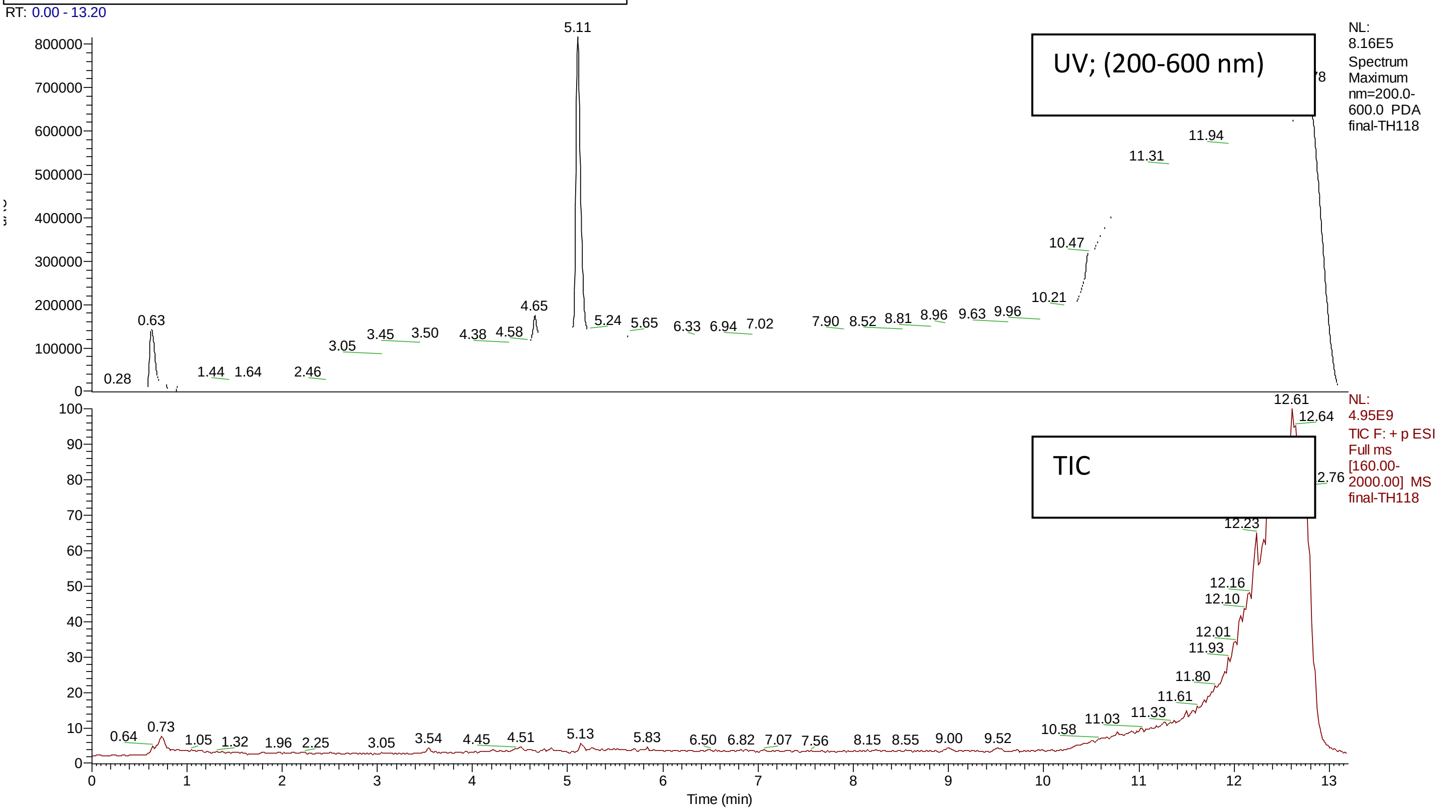

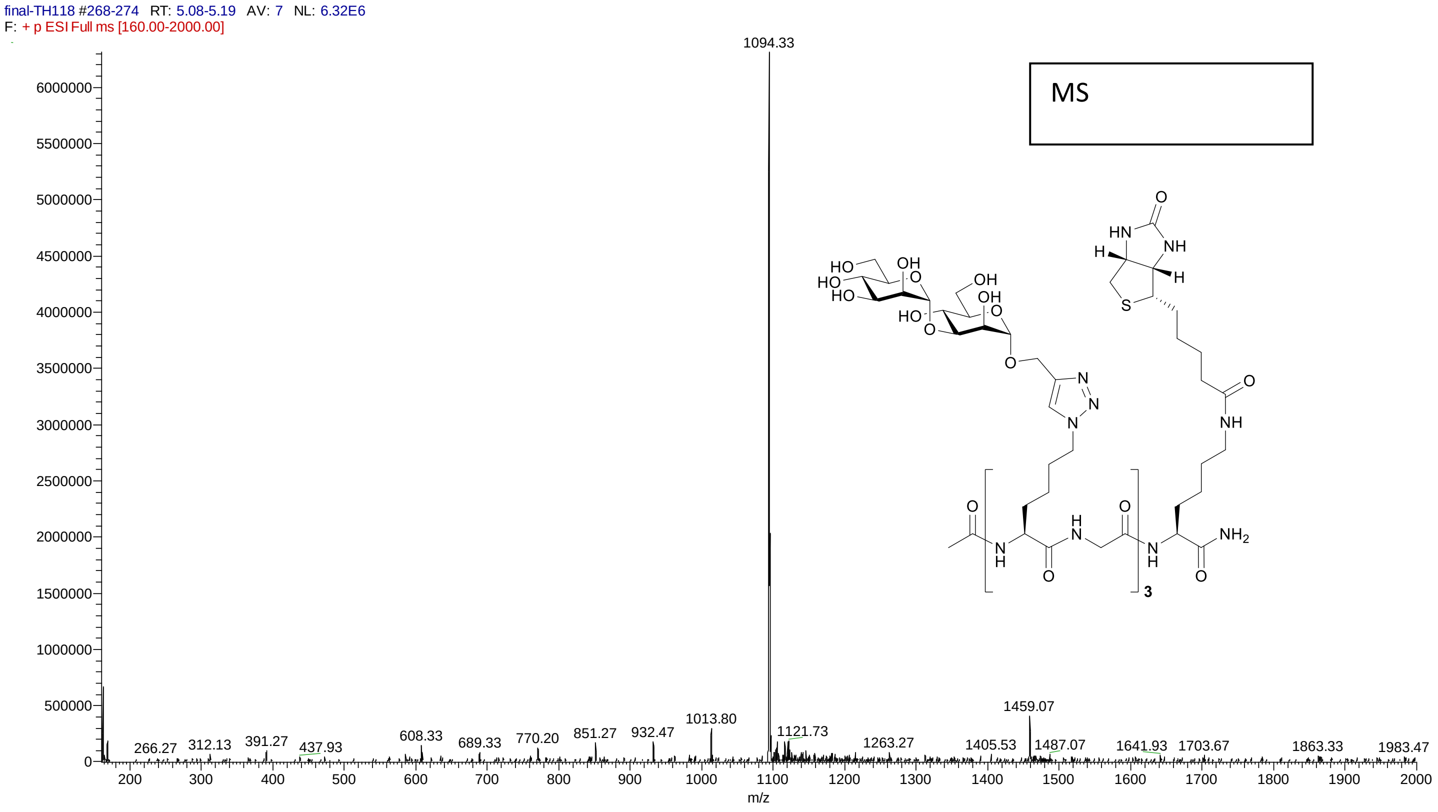

Compound c6

LC-MS Spectra; *(0 –> 50 % ACN, 13 min)*

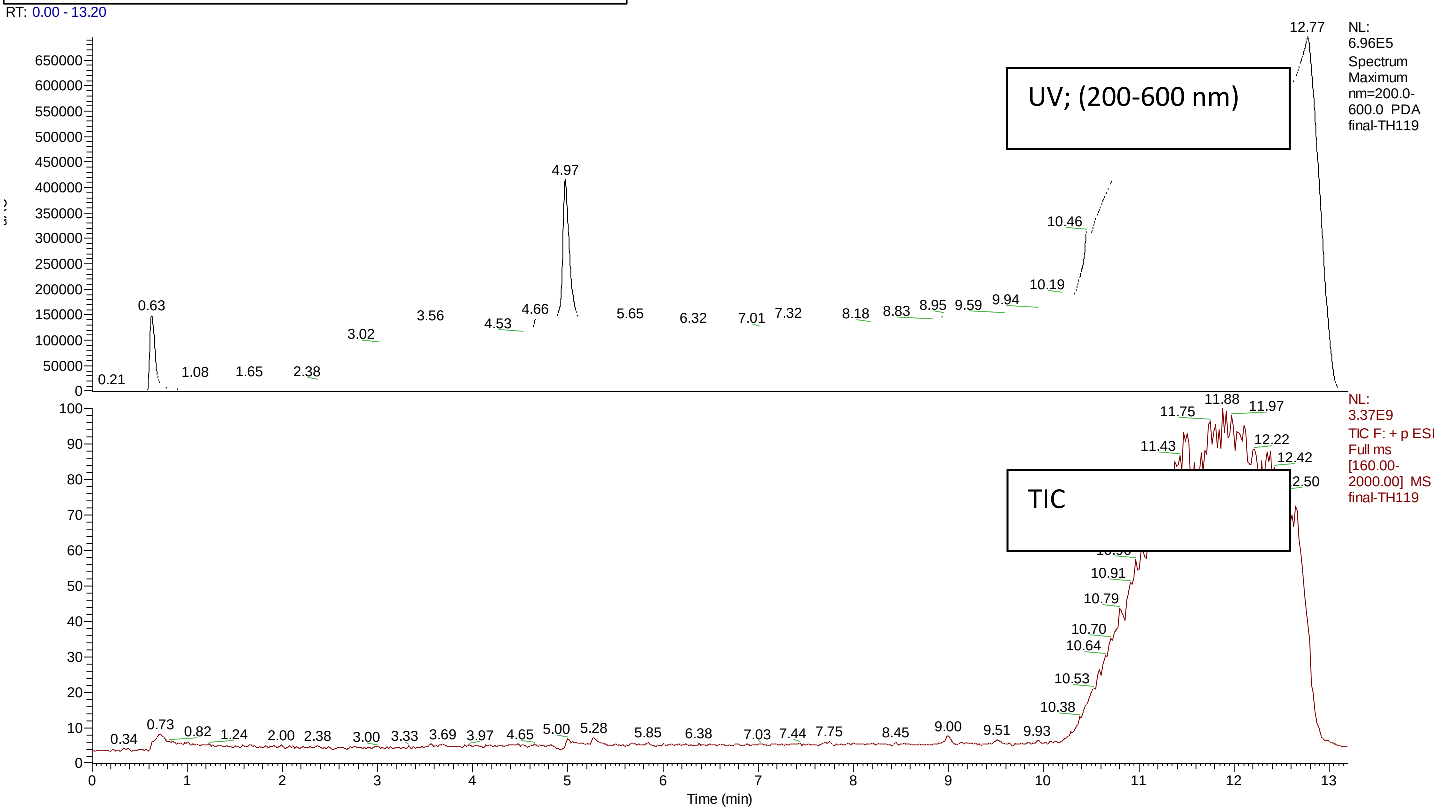

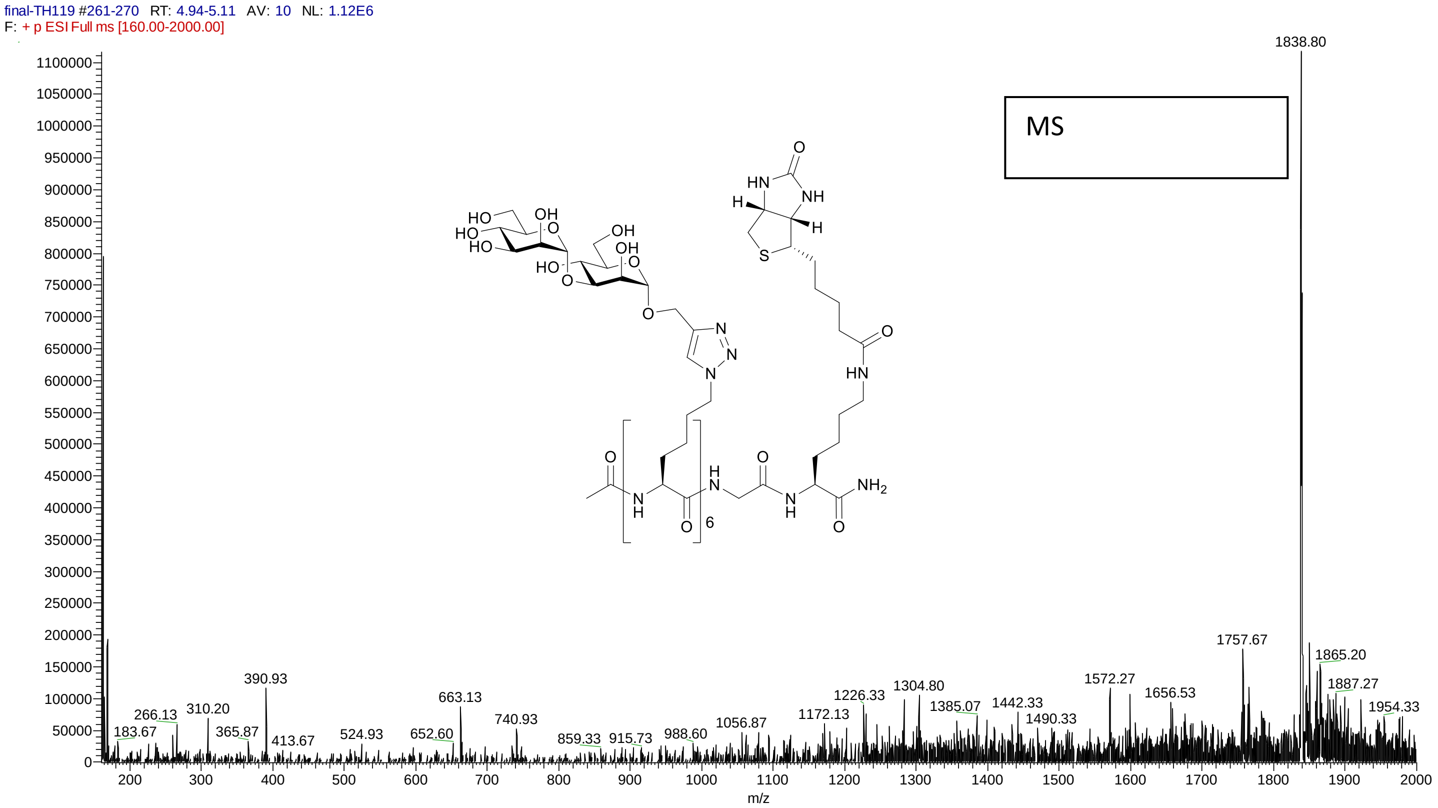

Compound d1

LC-MS Spectra; *(0 –> 50 % ACN, 13 min)*

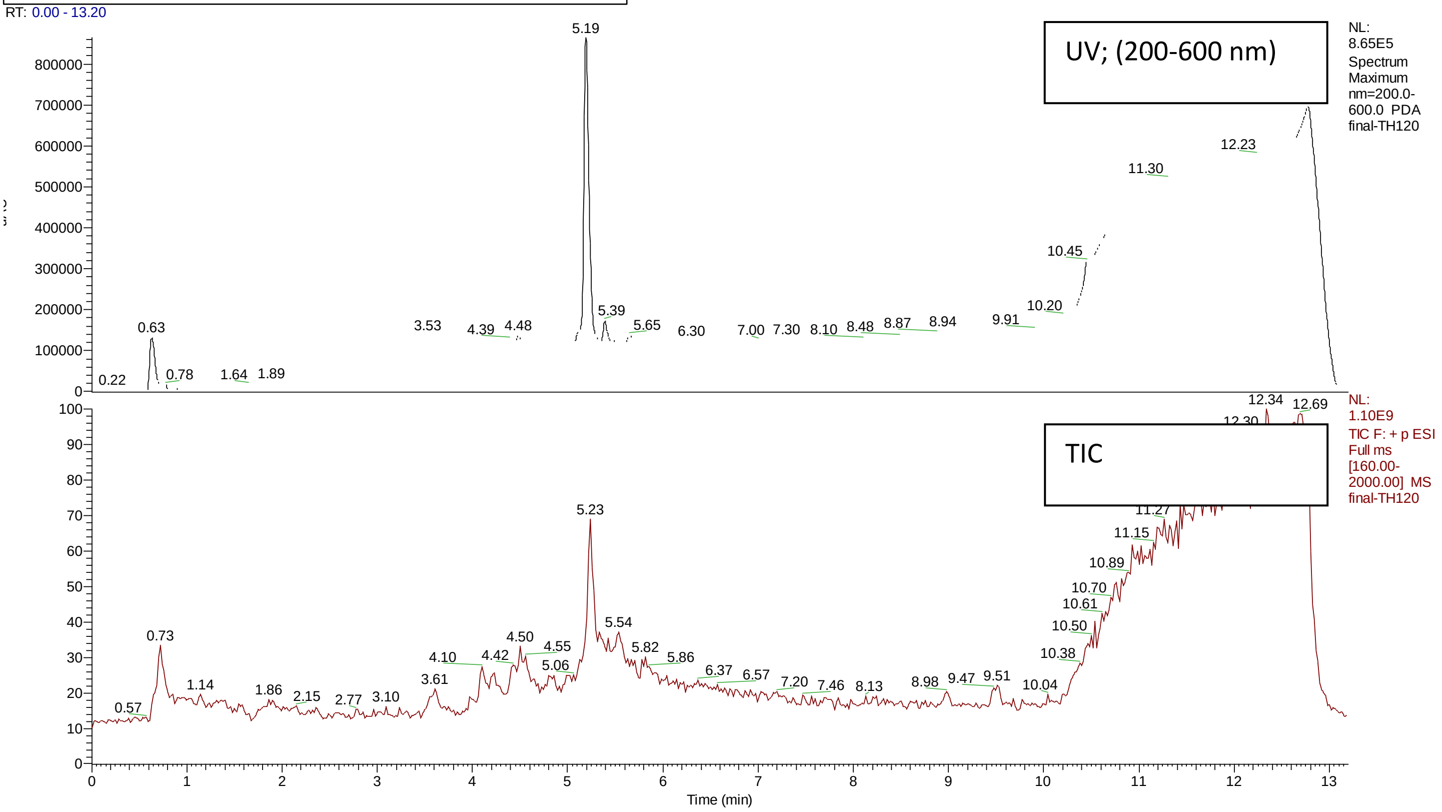

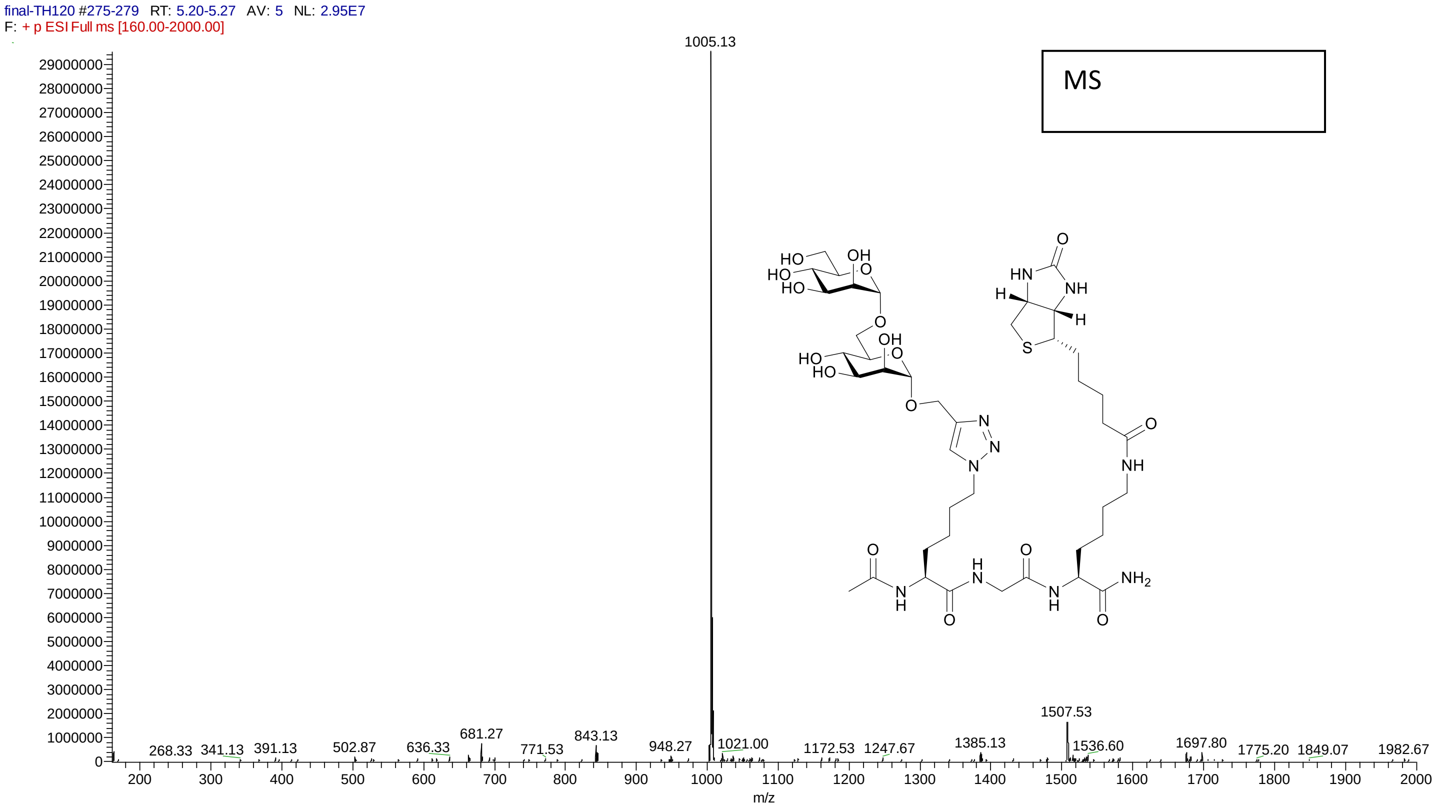

Compound d2

LC-MS Spectra; *(0 –> 50 % ACN, 13 min)*

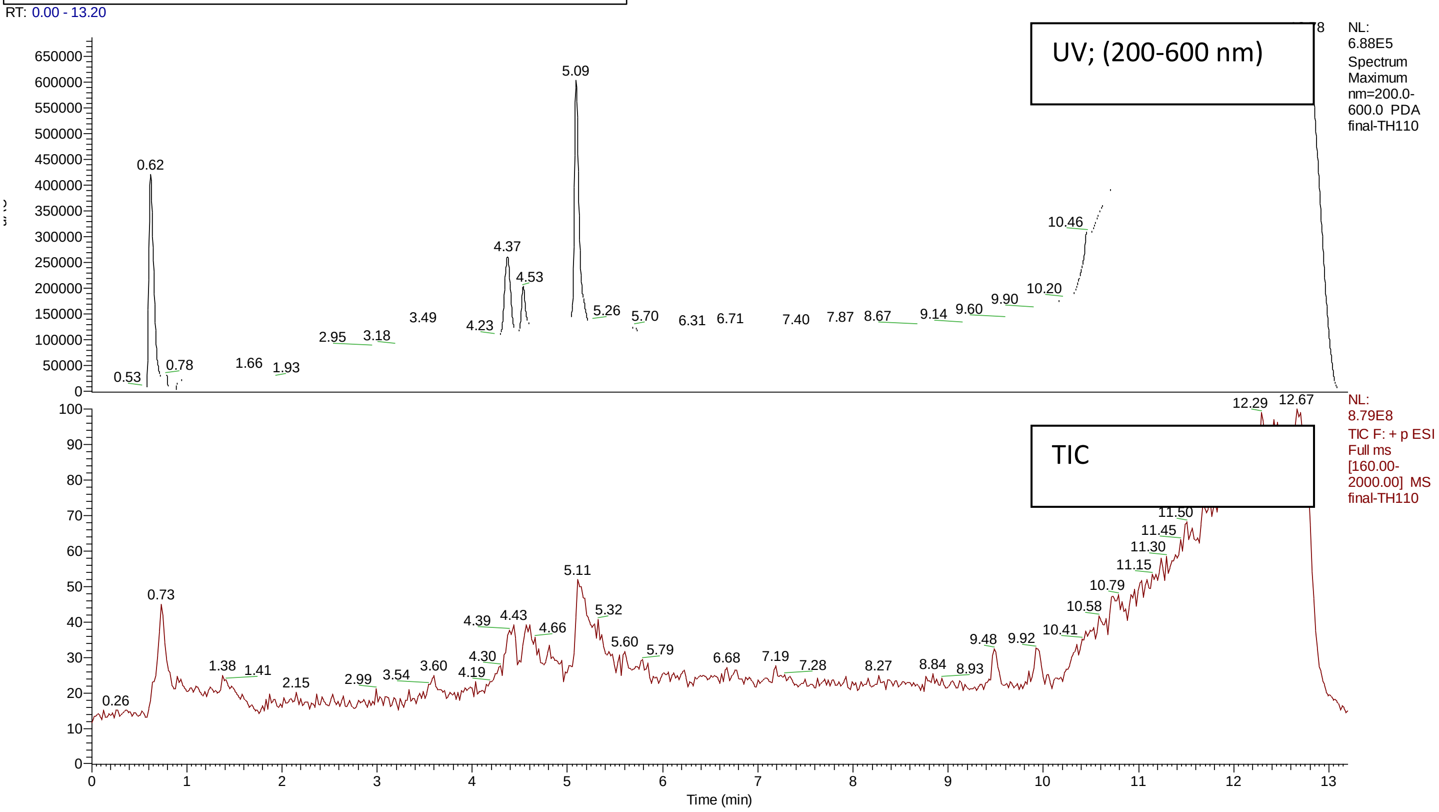

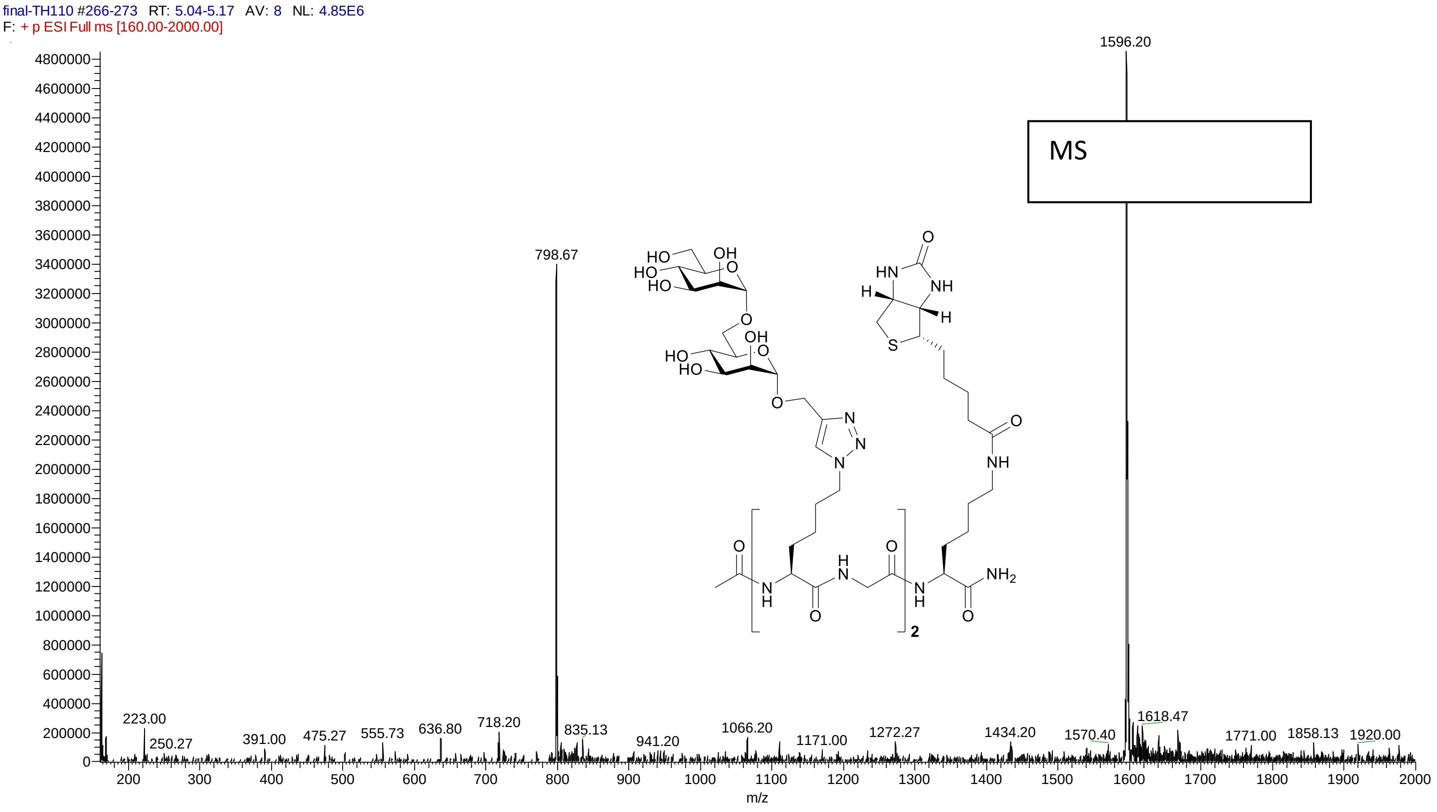

Compound d3

LC-MS Spectra; *(0 –> 50 % ACN, 13 min)*

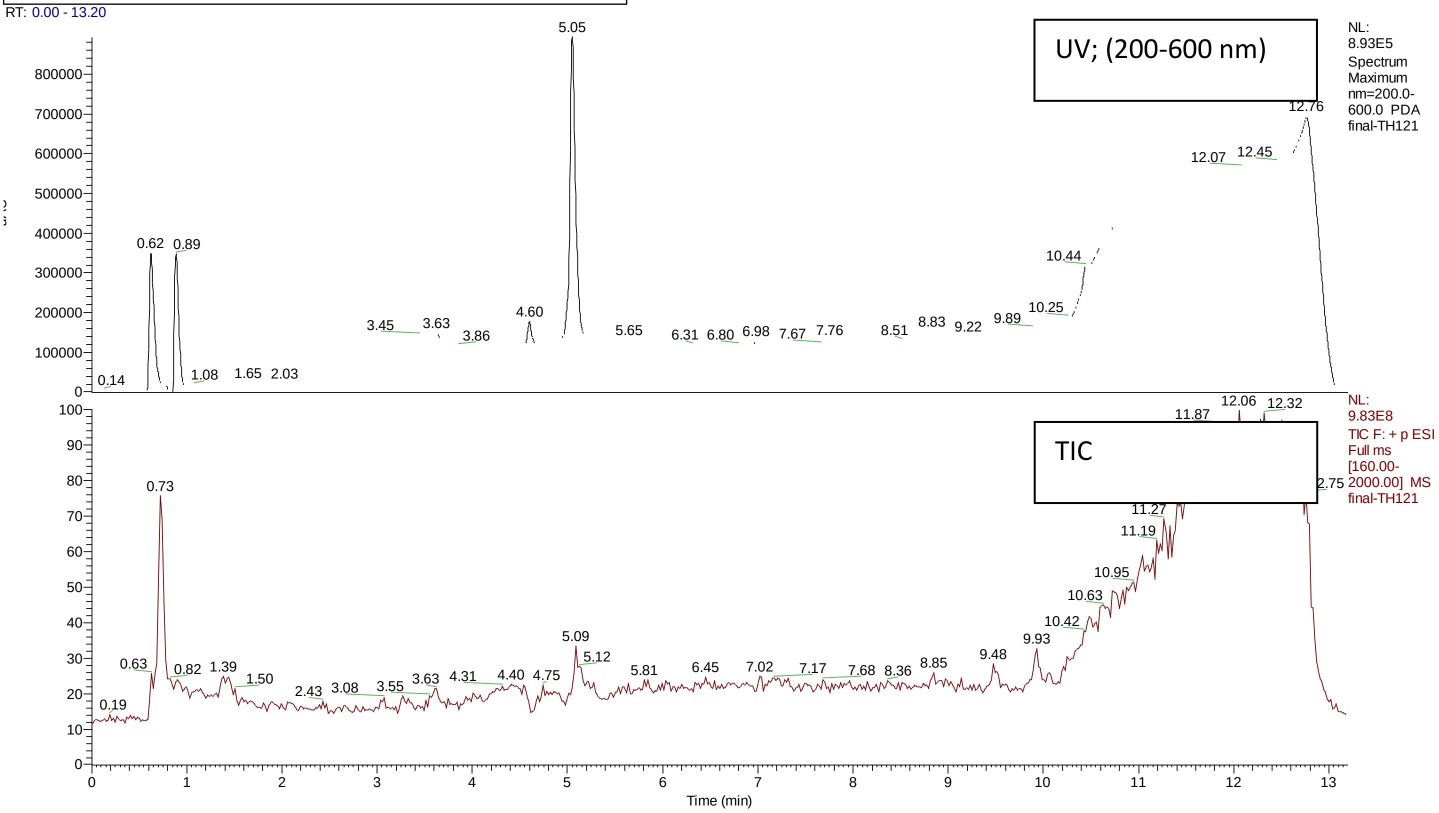

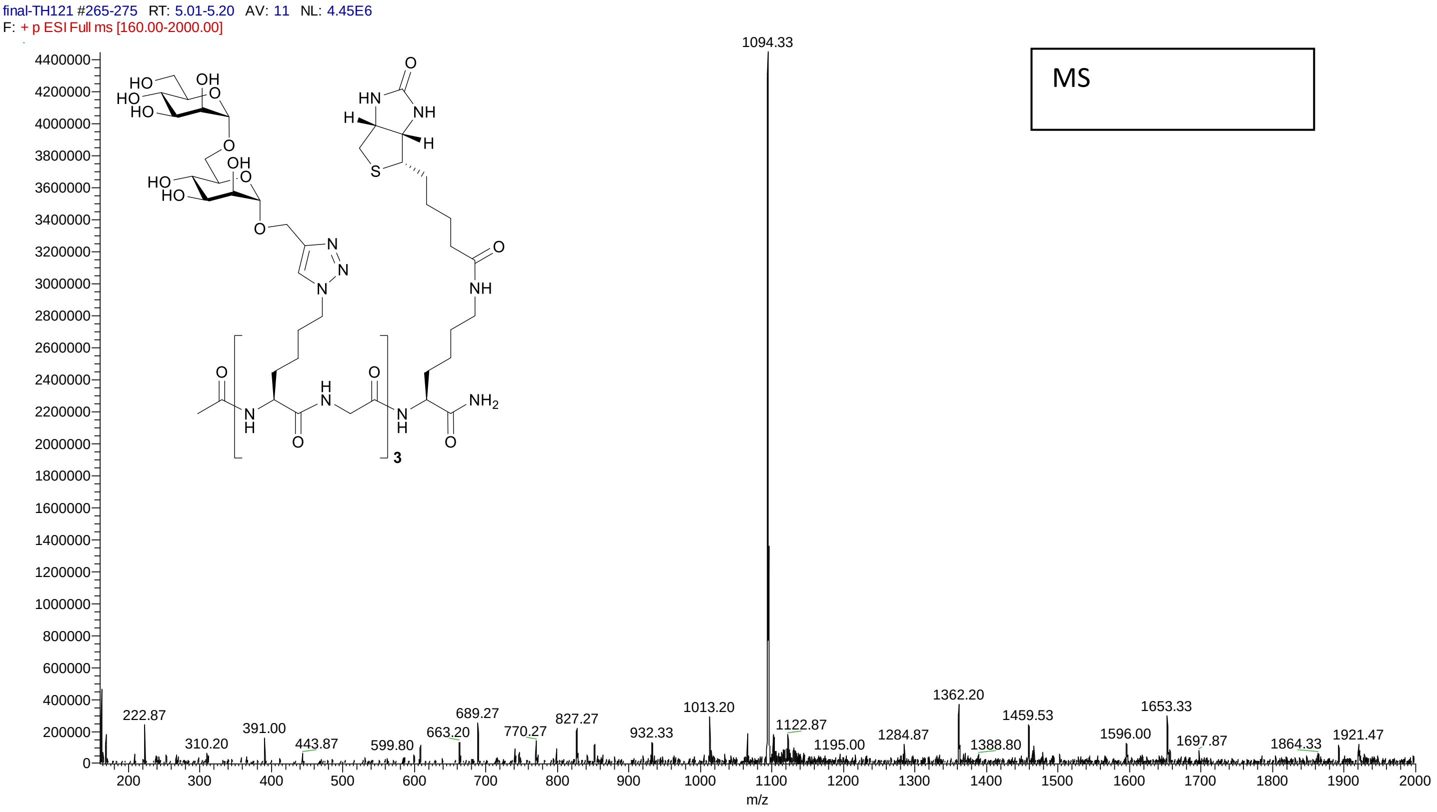

Compound d6

LC-MS Spectra; *(0 –> 50 % ACN, 13 min)*

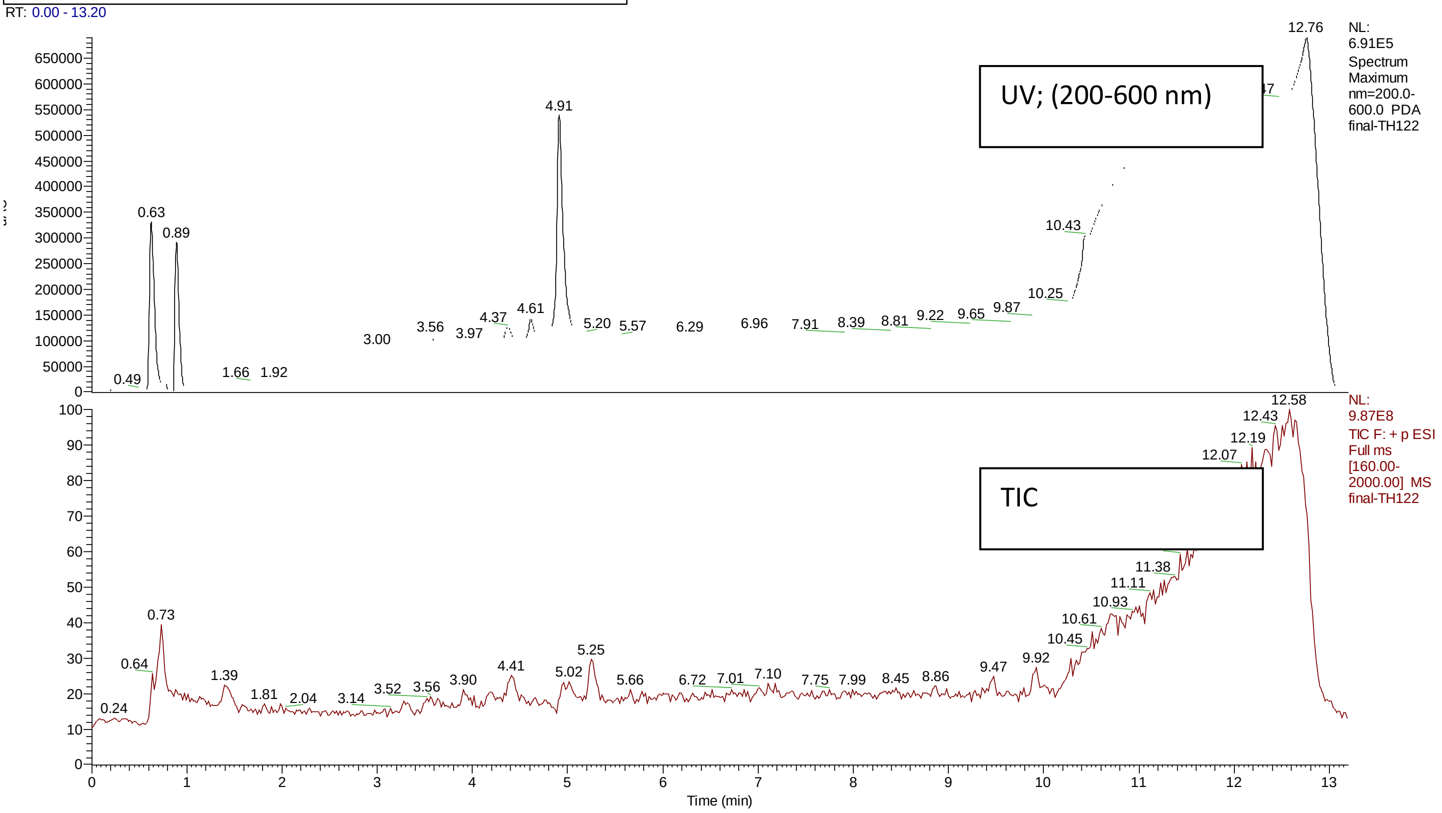

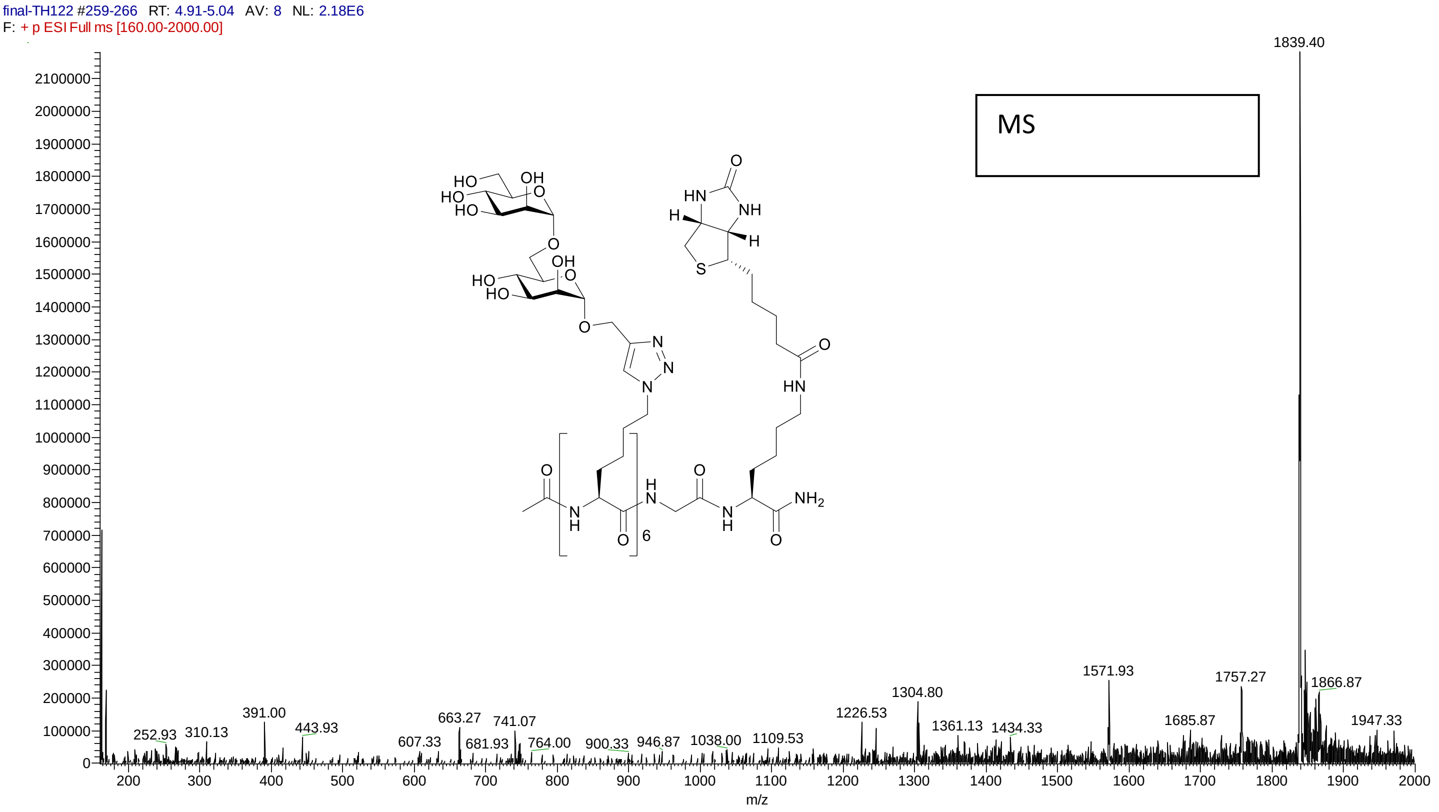

Compound e1

LC-MS Spectra; *(0 –> 50 % ACN, 13 min)*

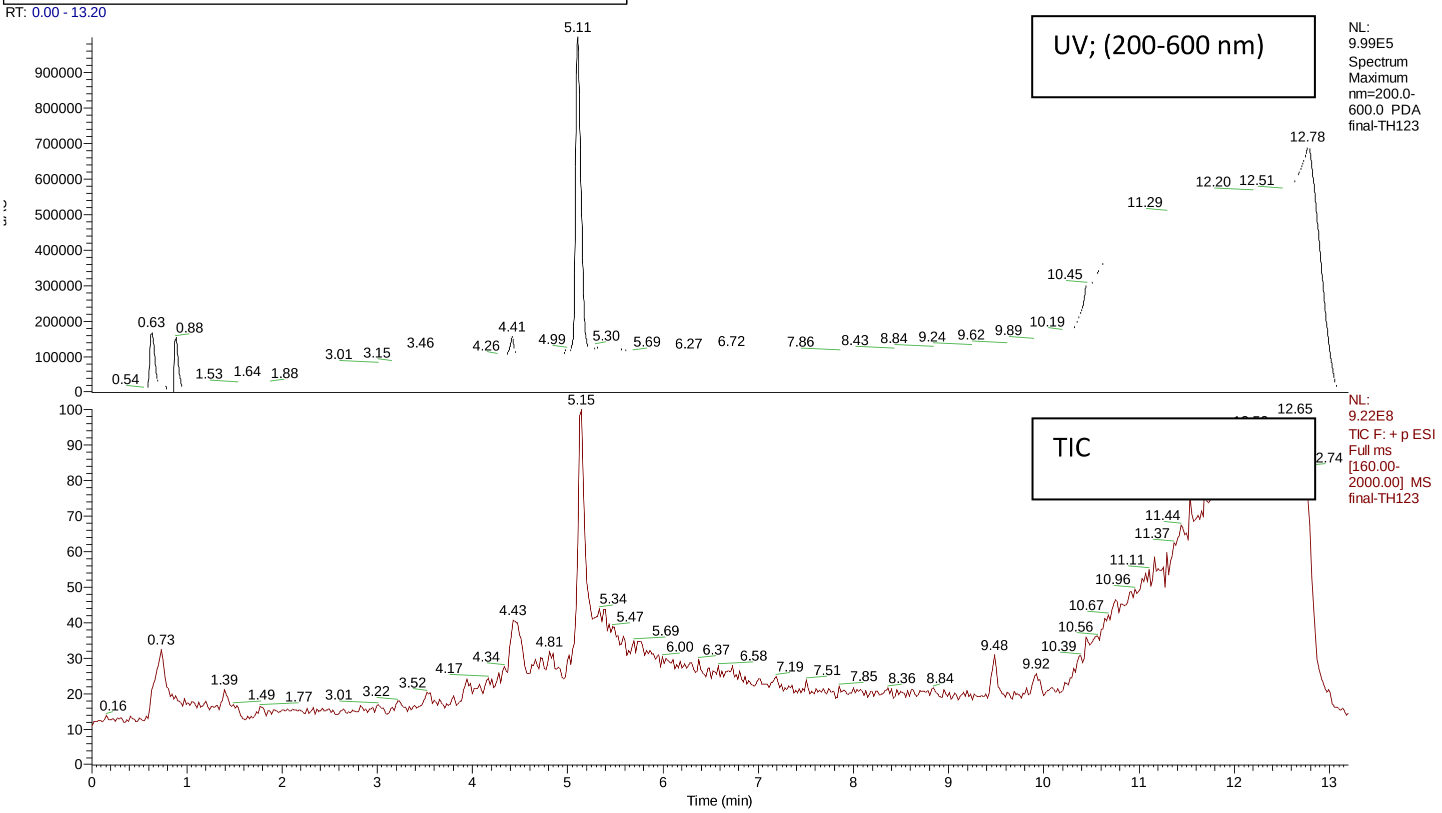

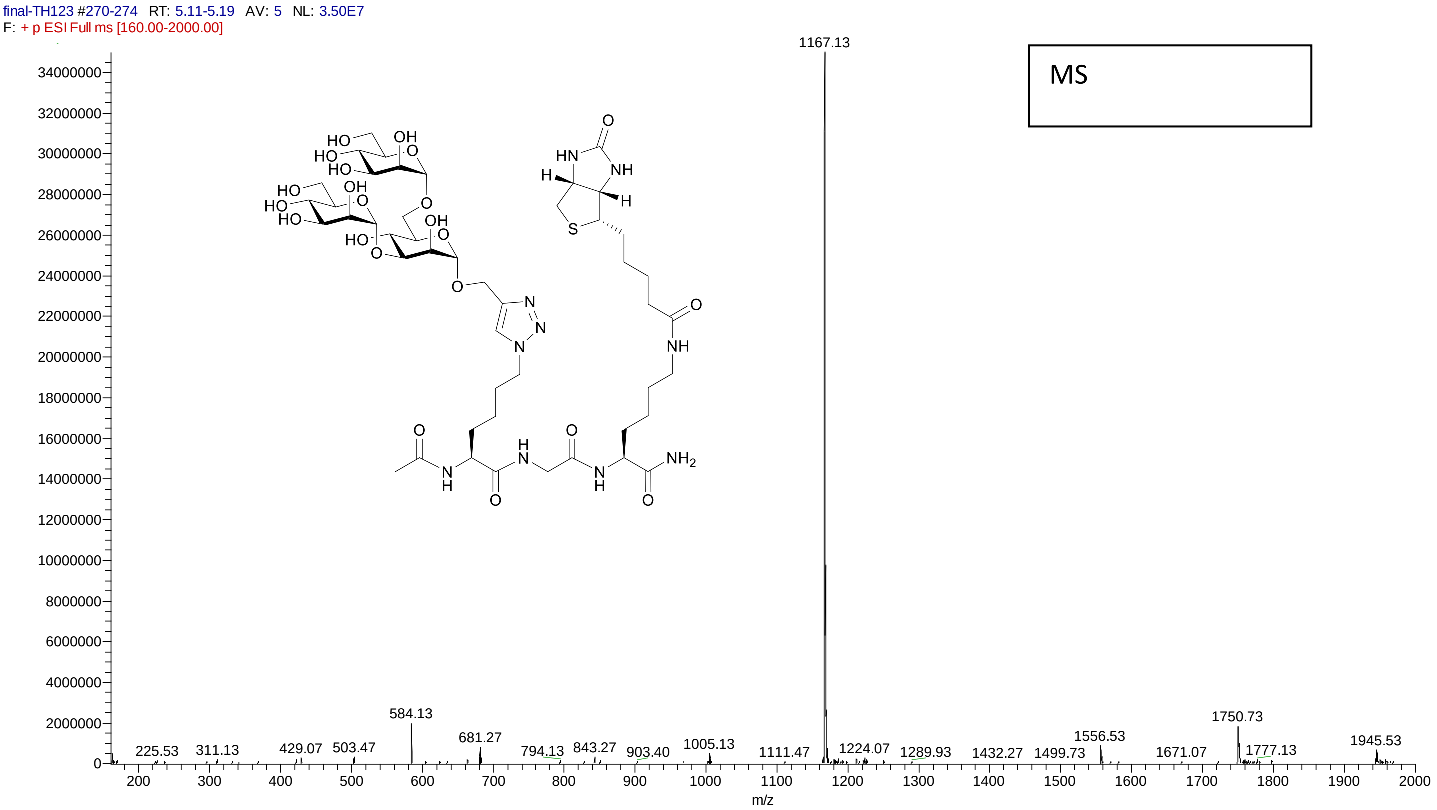

Compound e2

LC-MS Spectra; *(0 –> 50 % ACN, 13 min)*

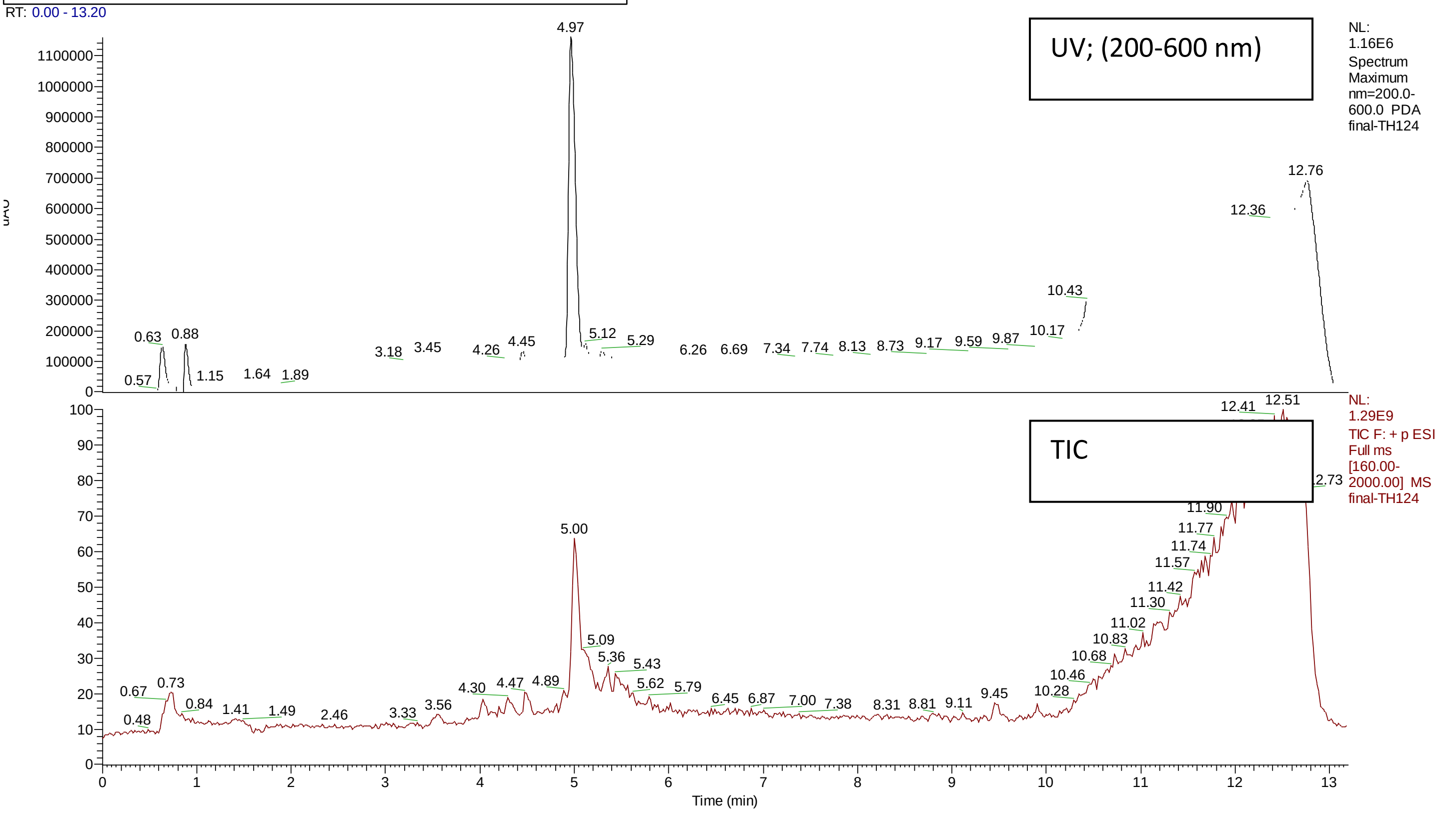

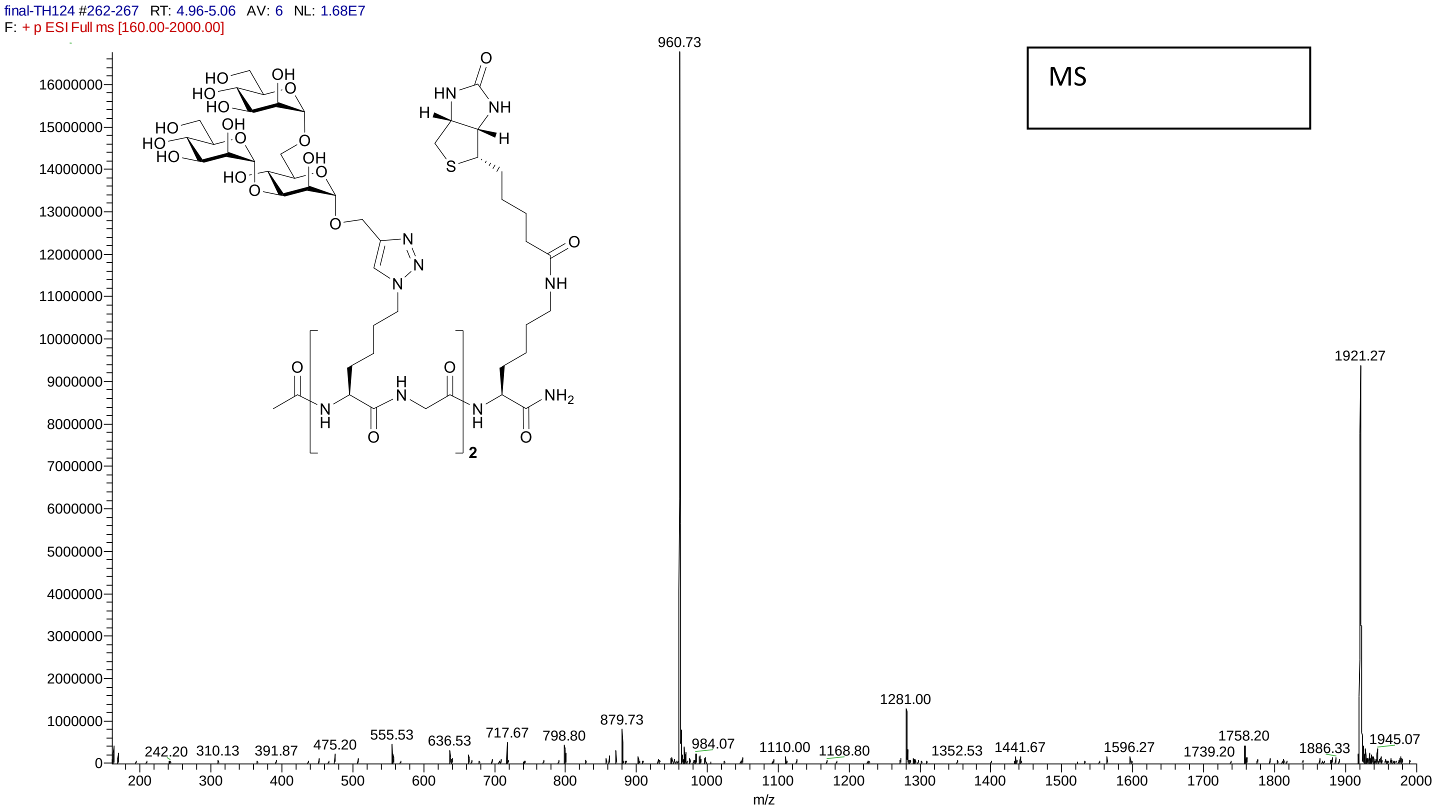

Compound e3

LC-MS Spectra; *(0 –> 50 % ACN, 13 min)*

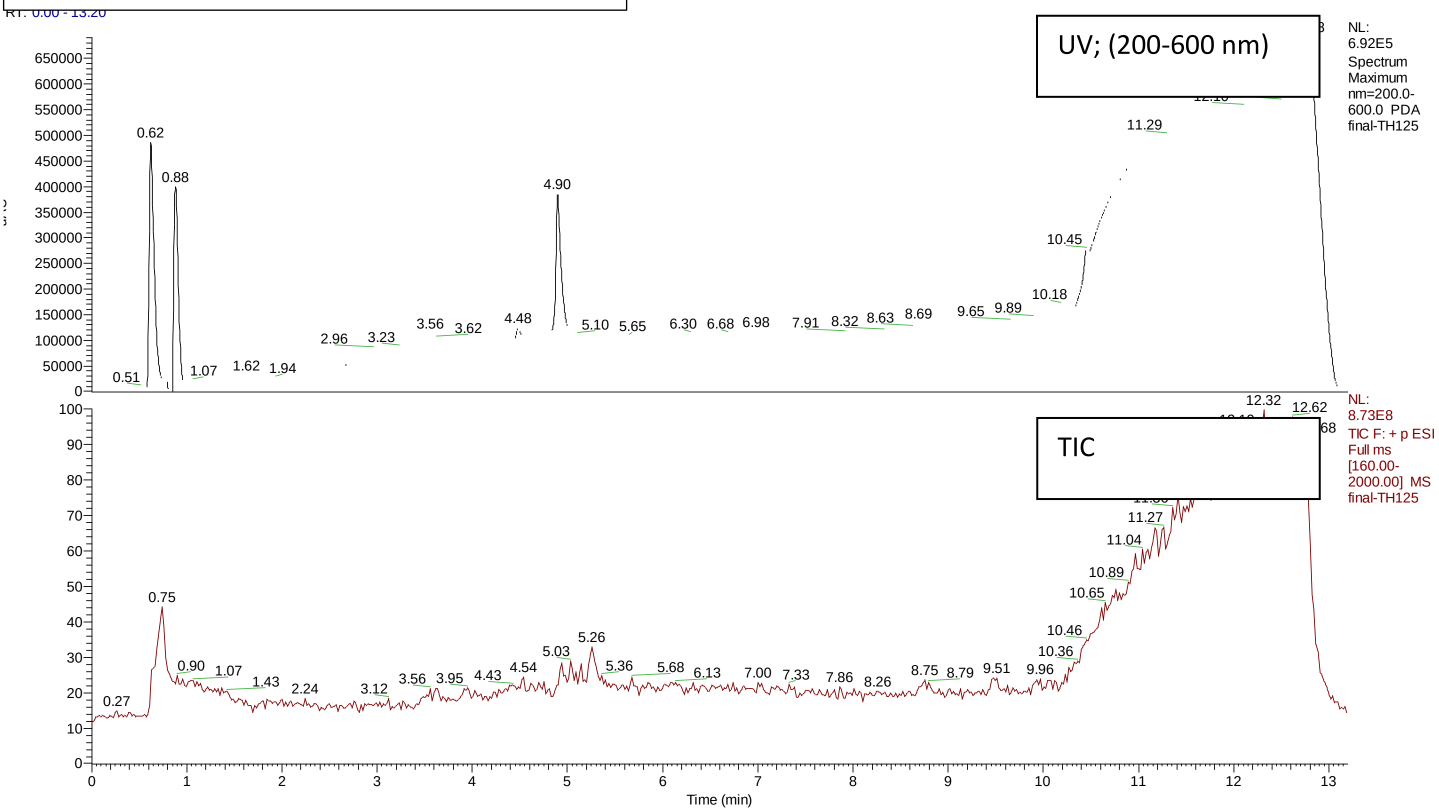

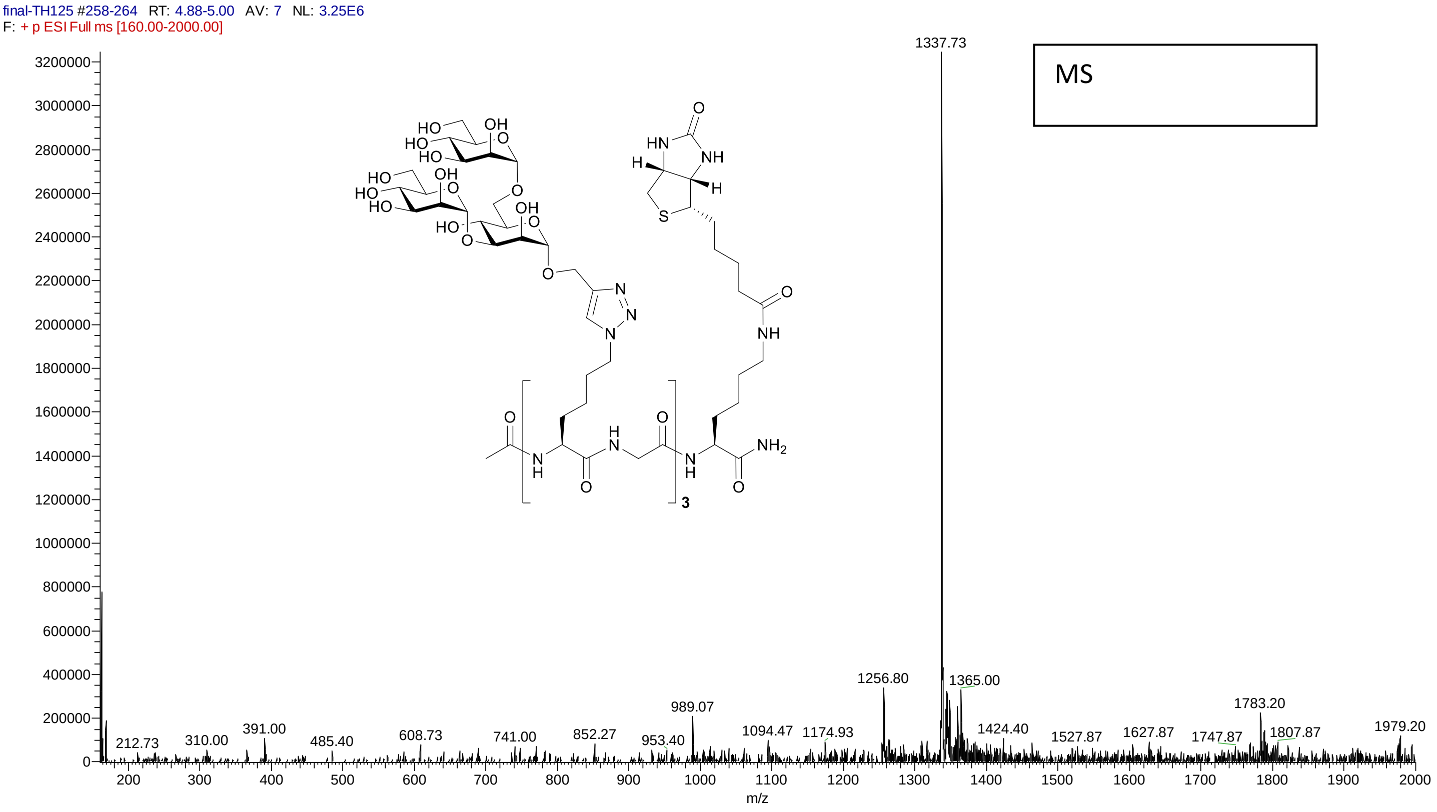

Compound e6

LC-MS Spectra; *(0 –> 50 % ACN, 13 min)*

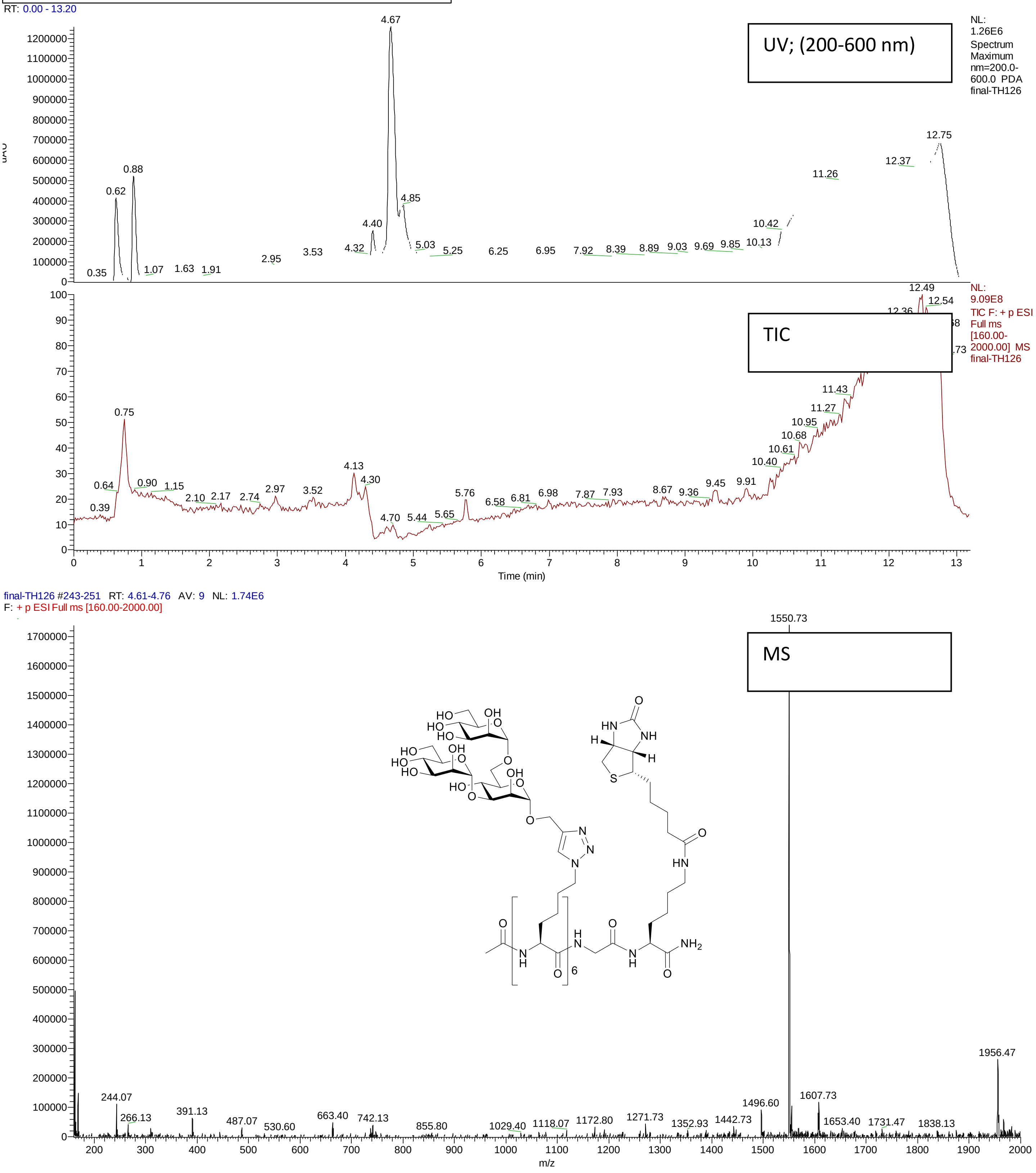

Spectra of generated building blocks

S9

^{1}H NMR Spectrum

(Compound **S9**)

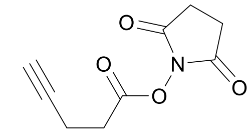

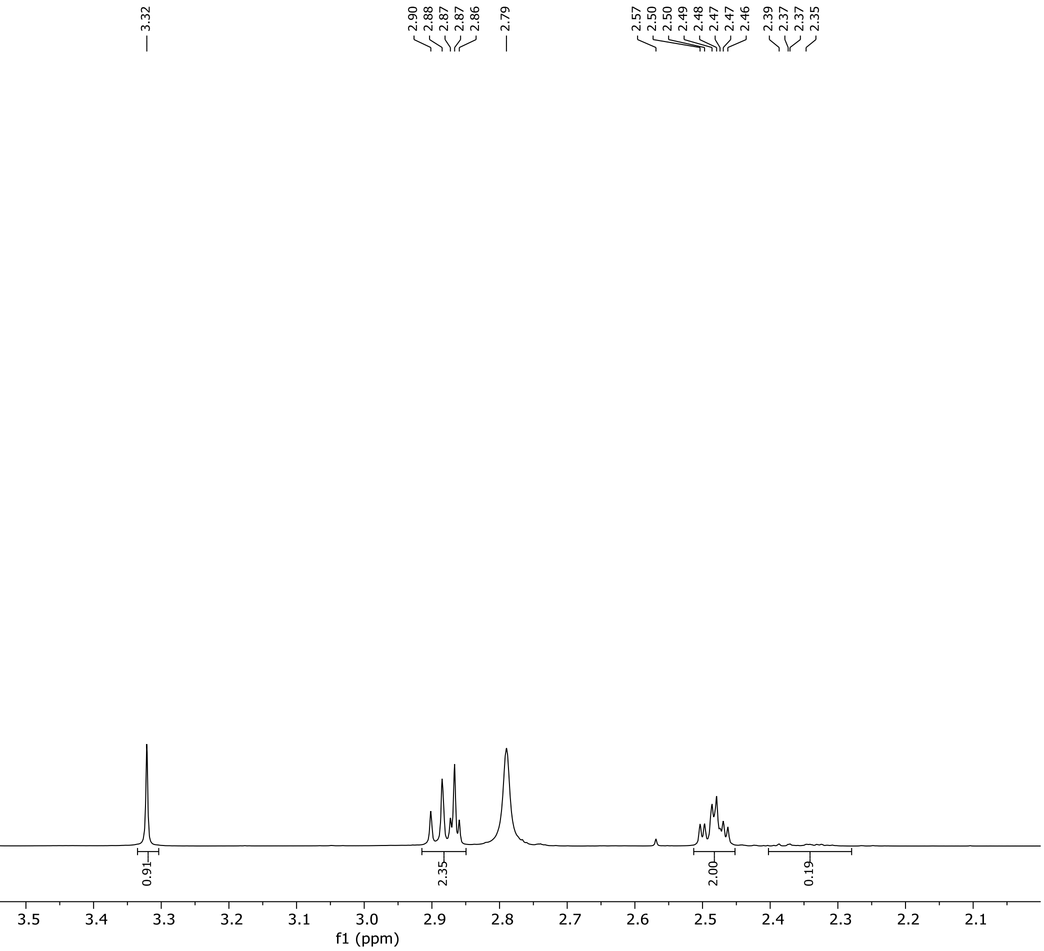

^{13}C APT NMR Spectrum

(Compound **S9**)

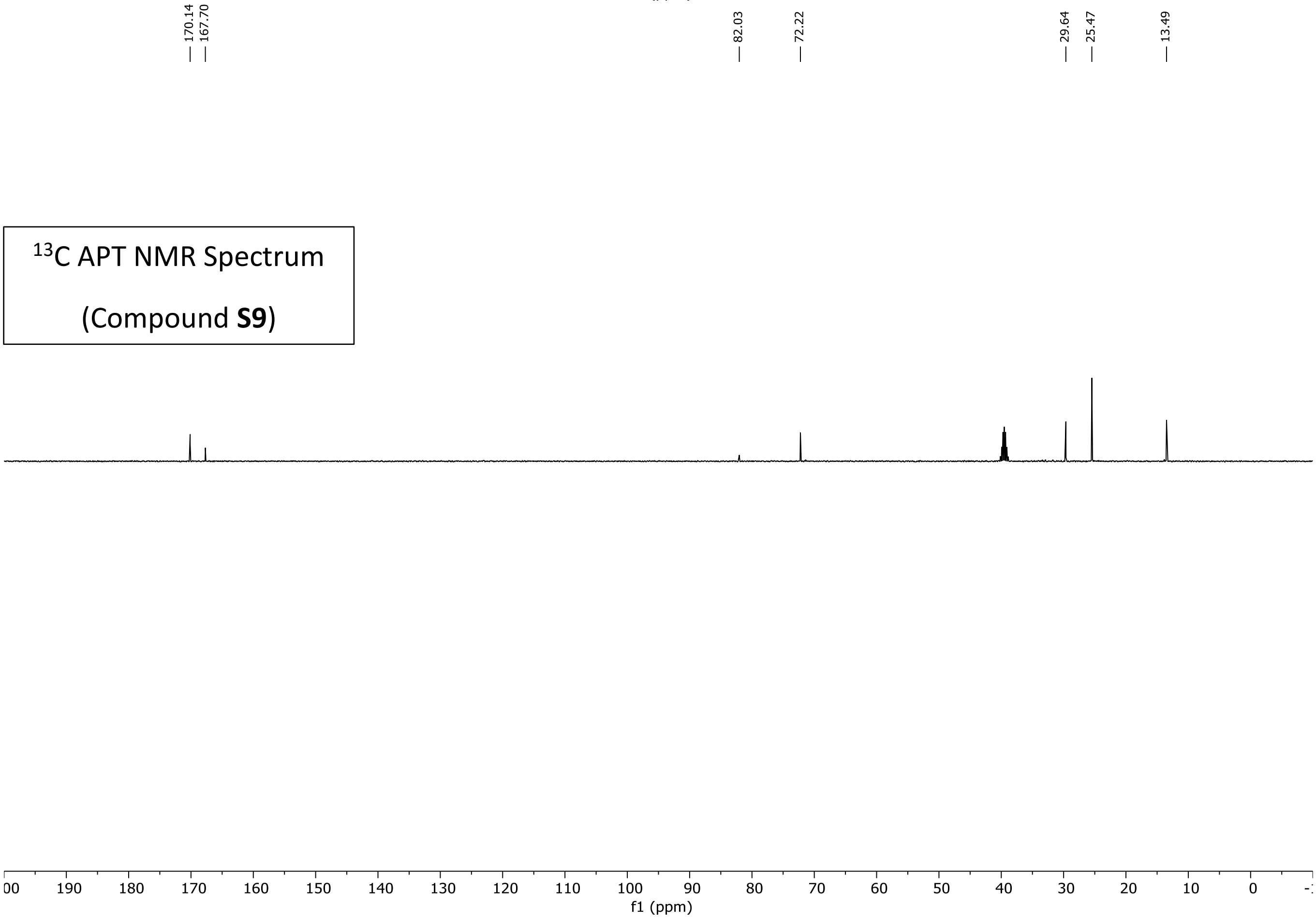

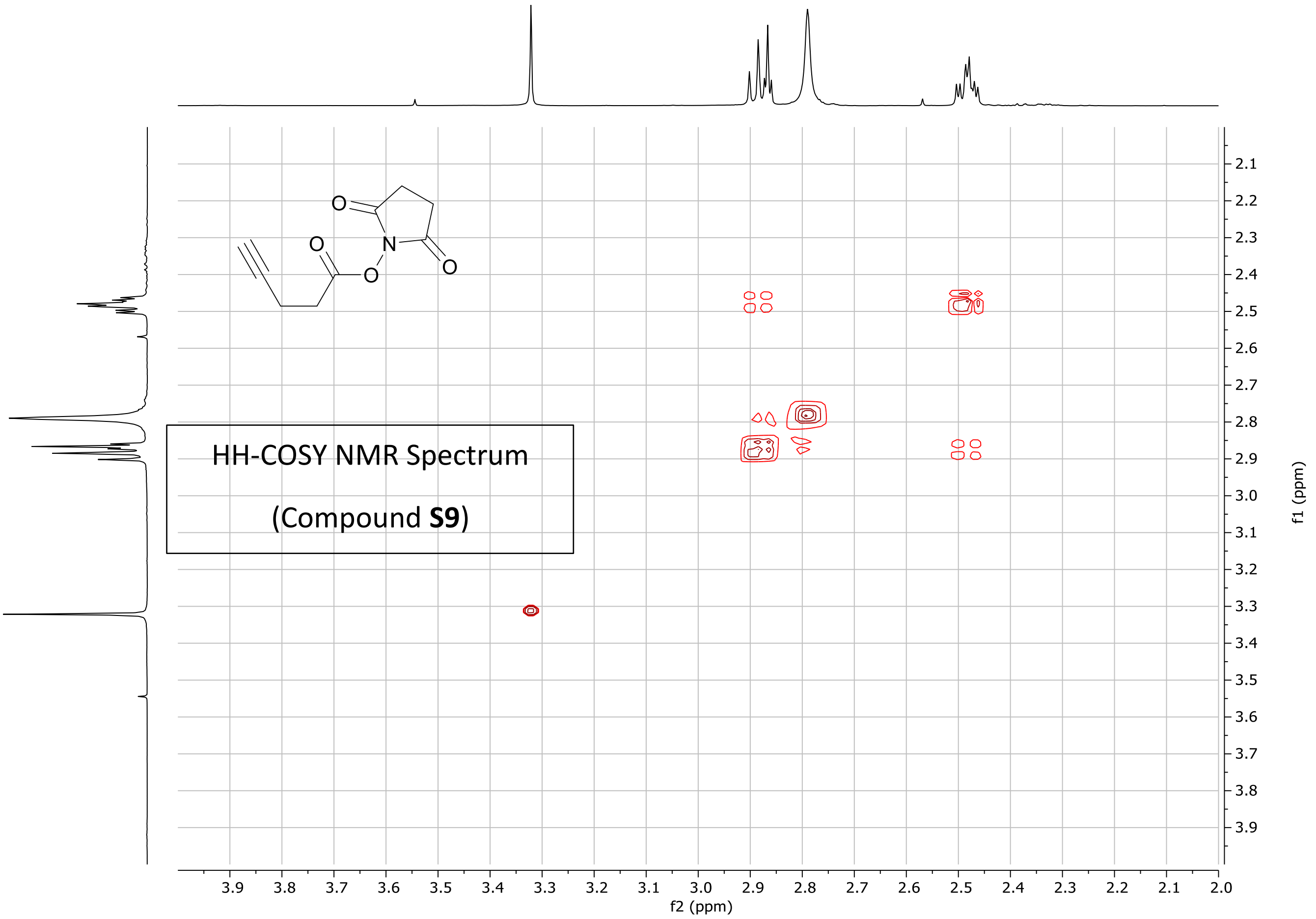

HH-COSY NMR Spectrum
(Compound **S9**)
f2 (ppm)
f1 (ppm)

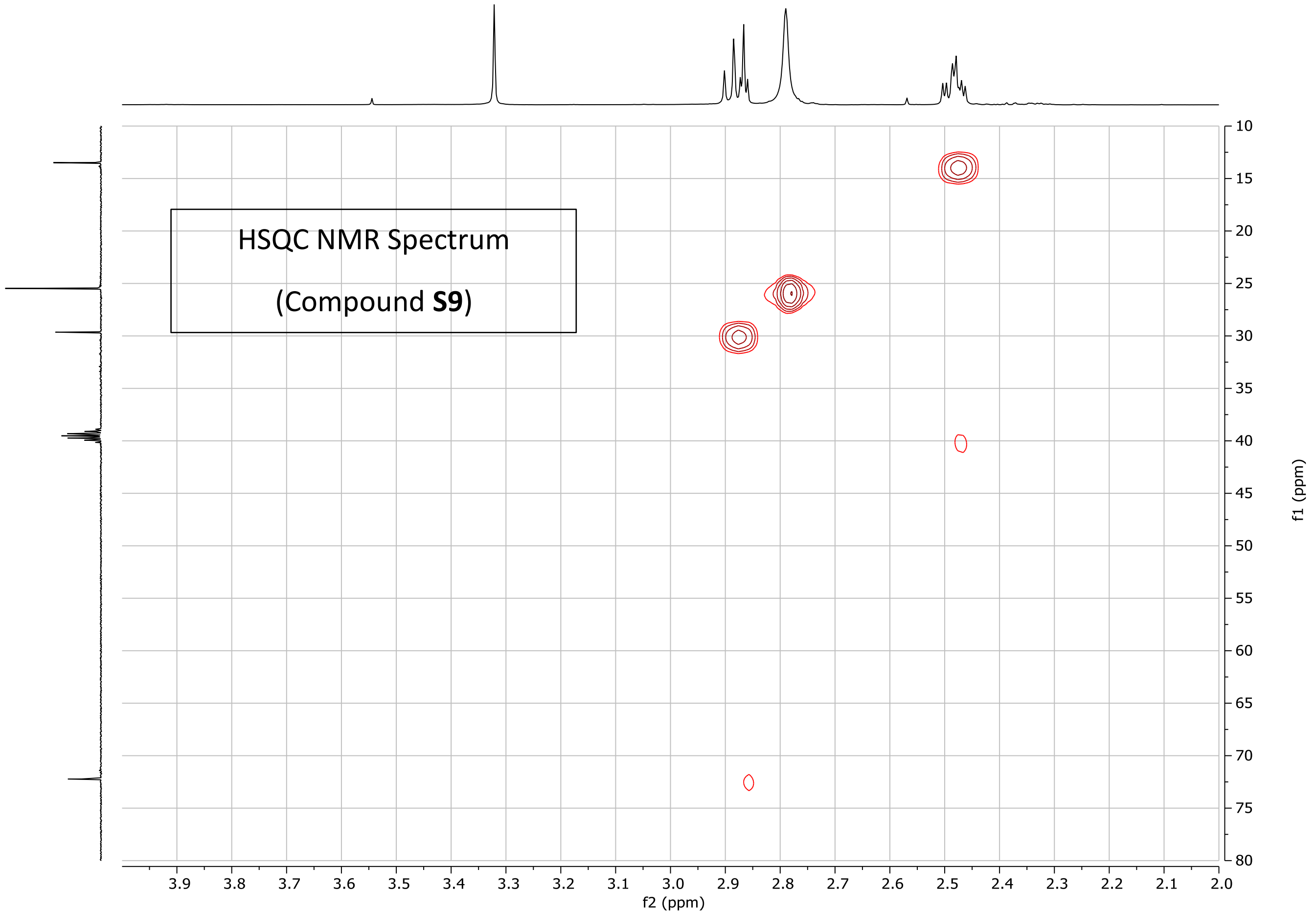

HSQC NMR Spectrum
(Compound **S9**)
f2 (ppm)
f1 (ppm)

23

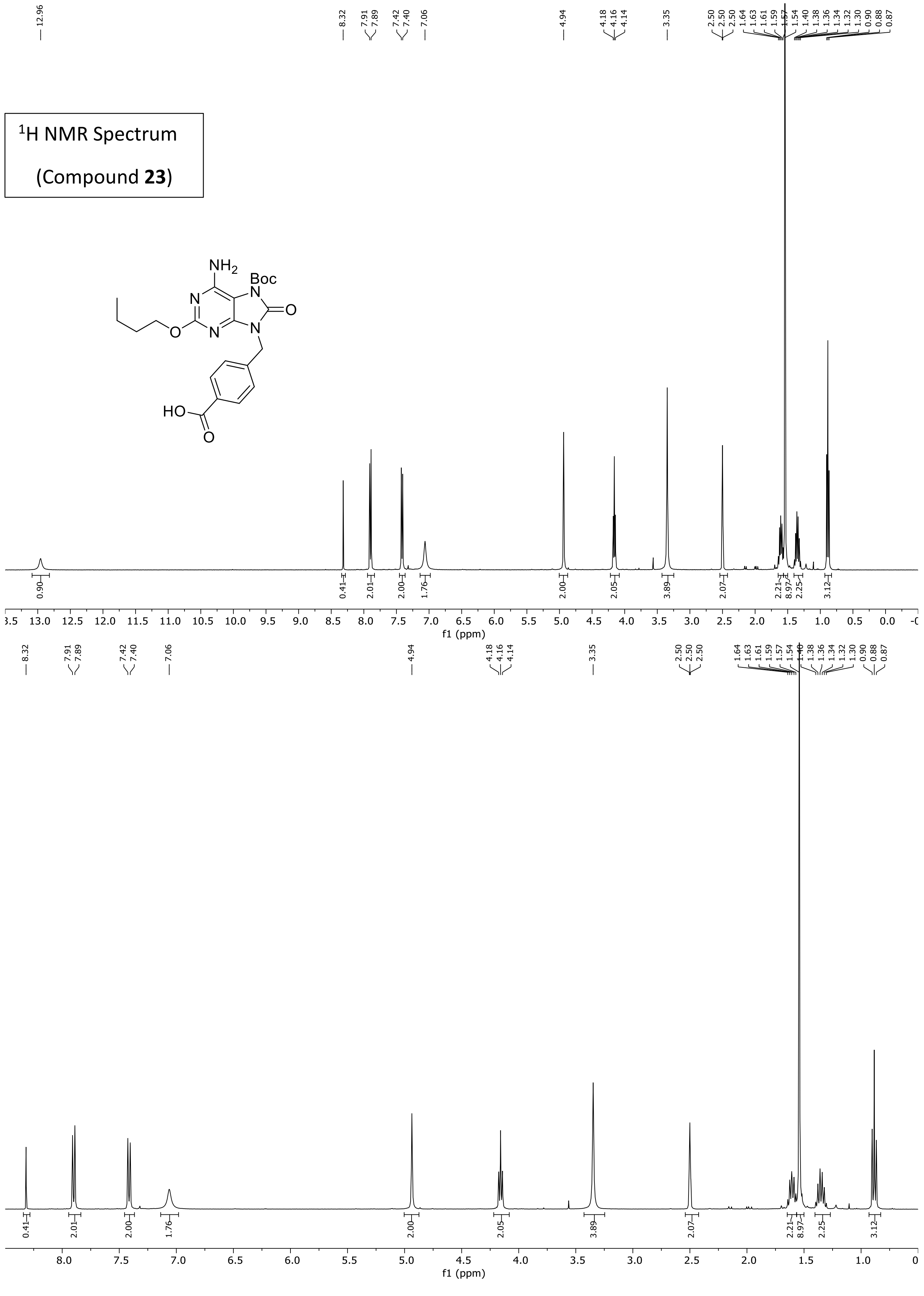

^{13}C APT NMR Spectrum

(Compound **23**)

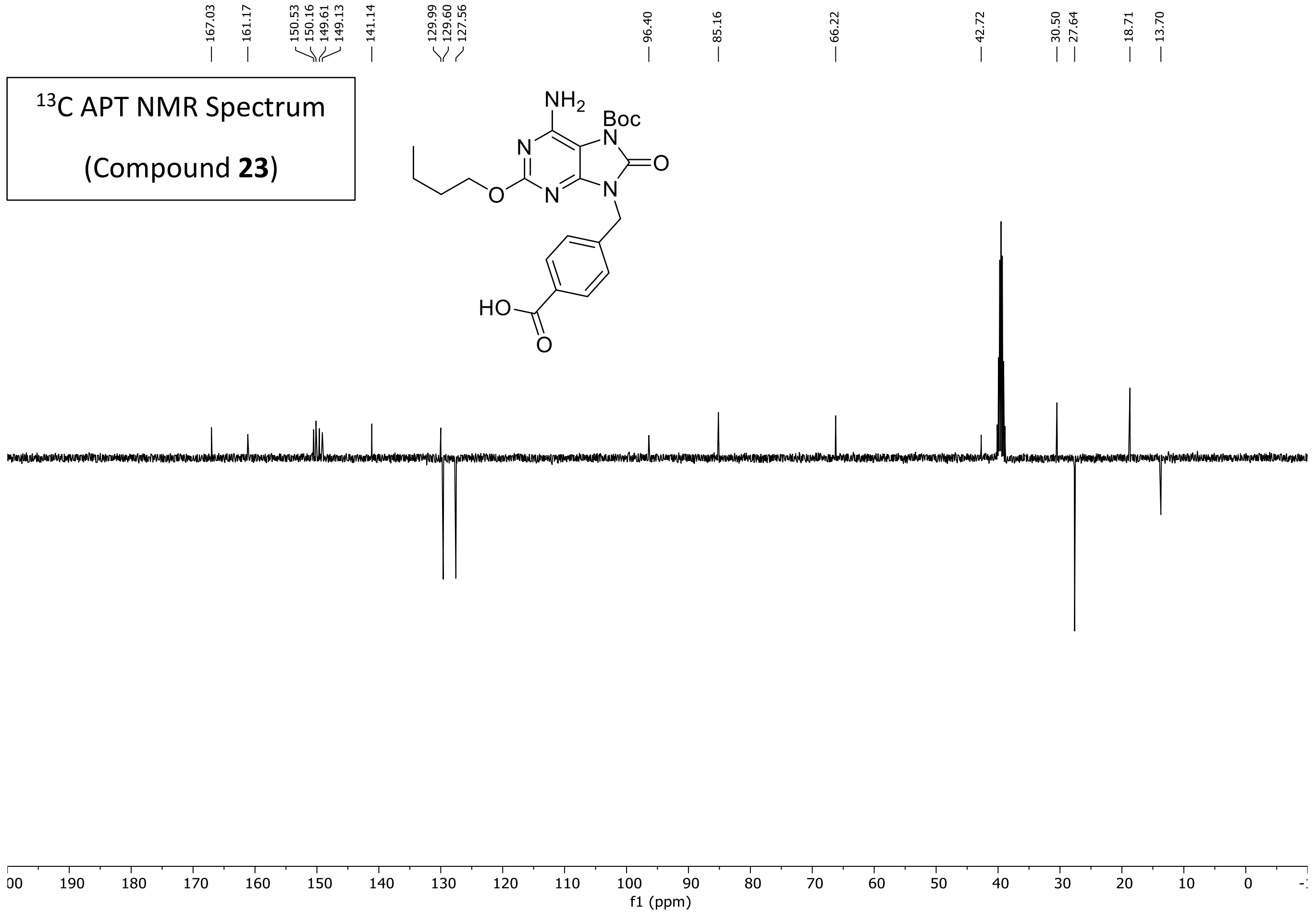

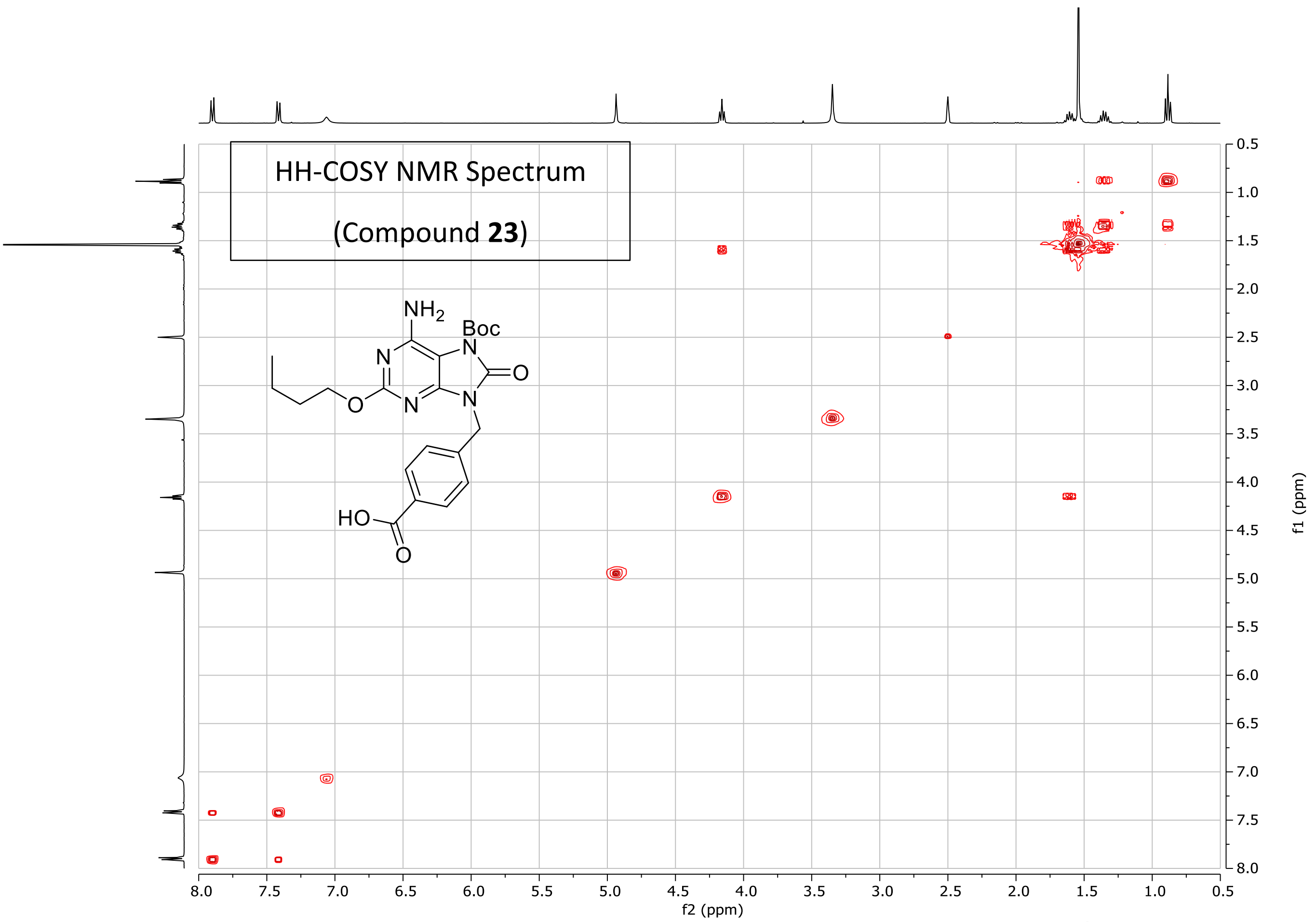
HH-COSY NMR Spectrum
(Compound **23**)
NH_2
Boc
N
N
N
N
O
O
HO
O
f2 (ppm)
f1 (ppm)

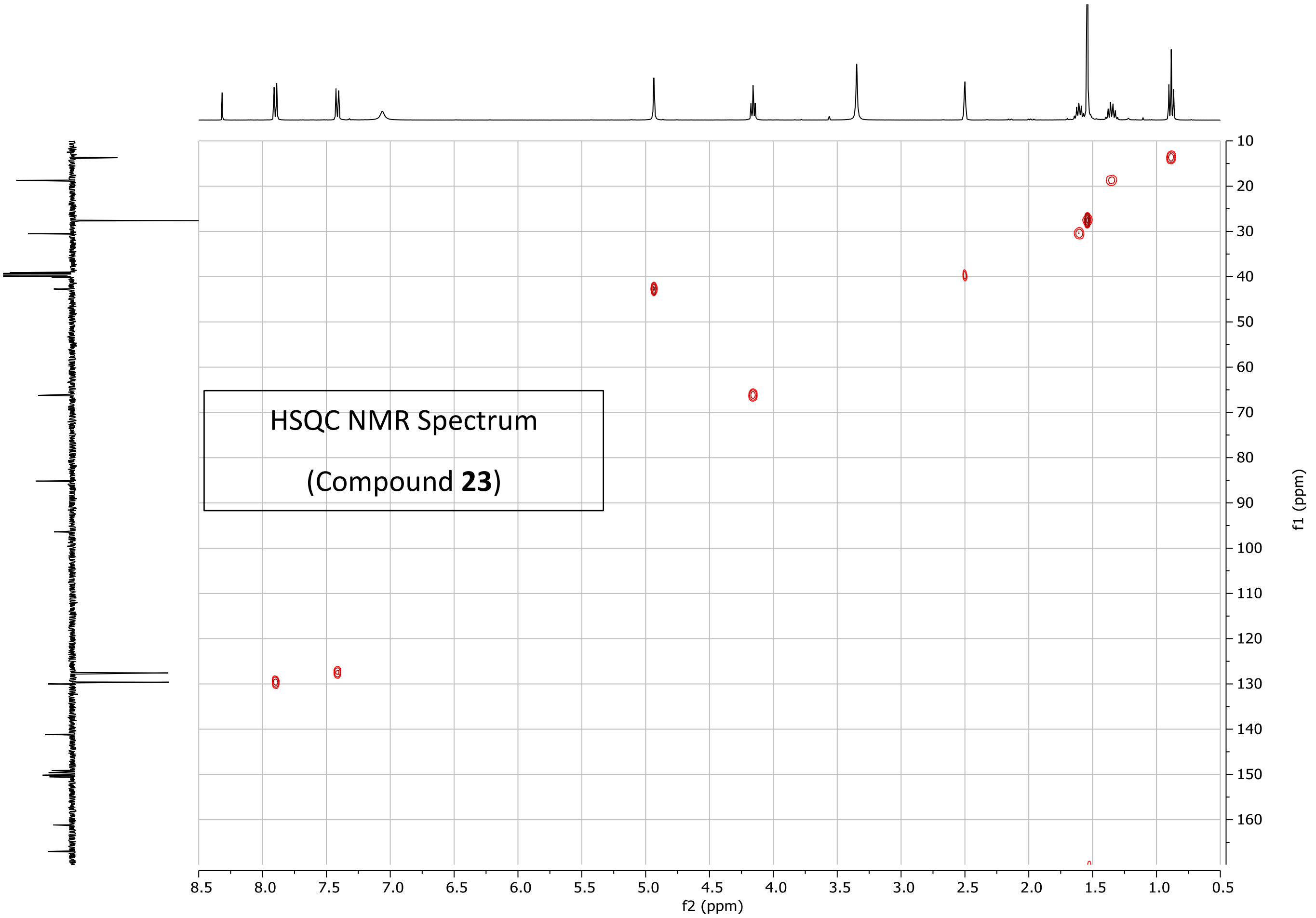
HSQC NMR Spectrum
(Compound **23**)
f2 (ppm)
f1 (ppm)

Spectra of CLR Ligands with Alkyne

19

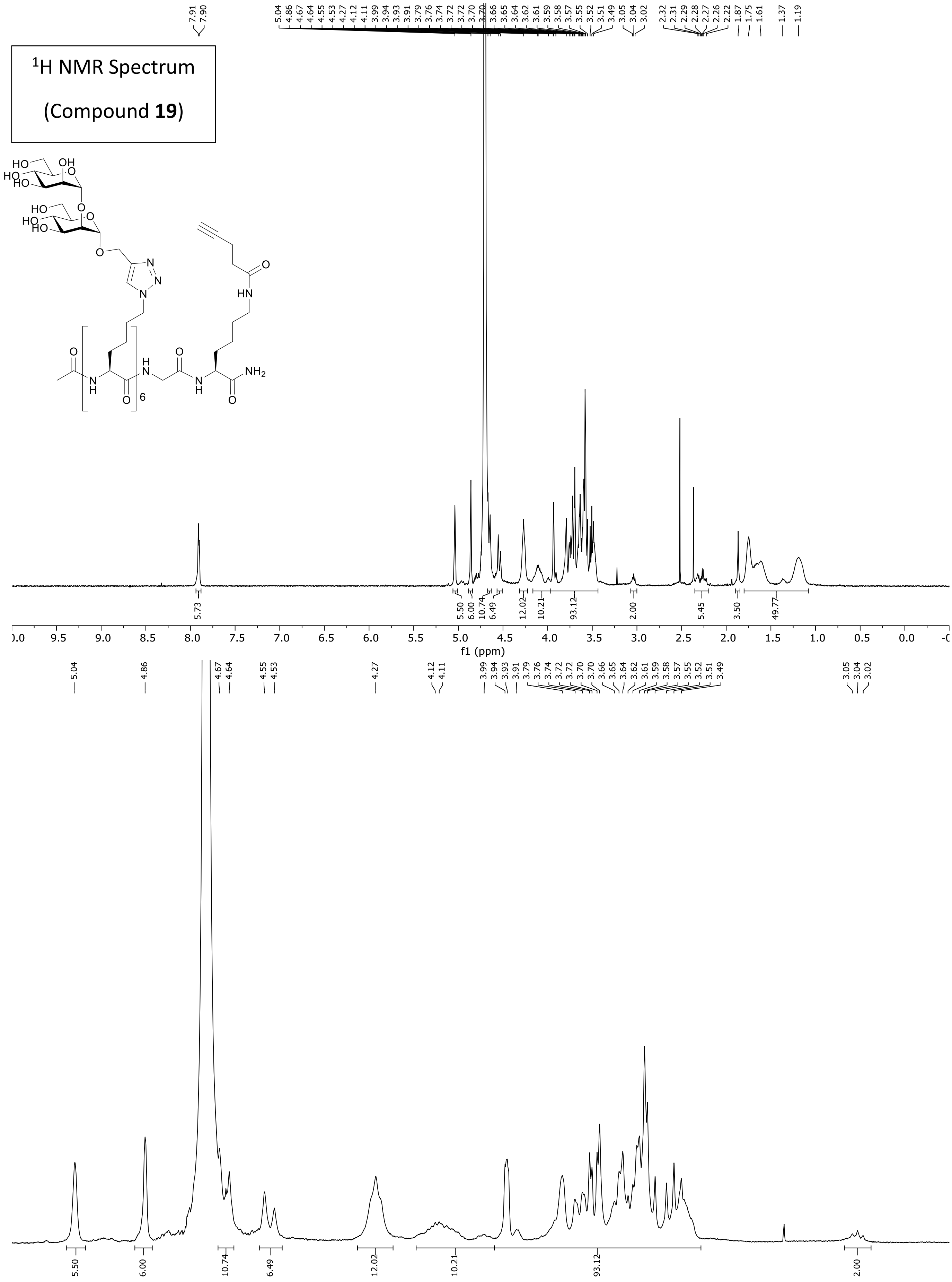

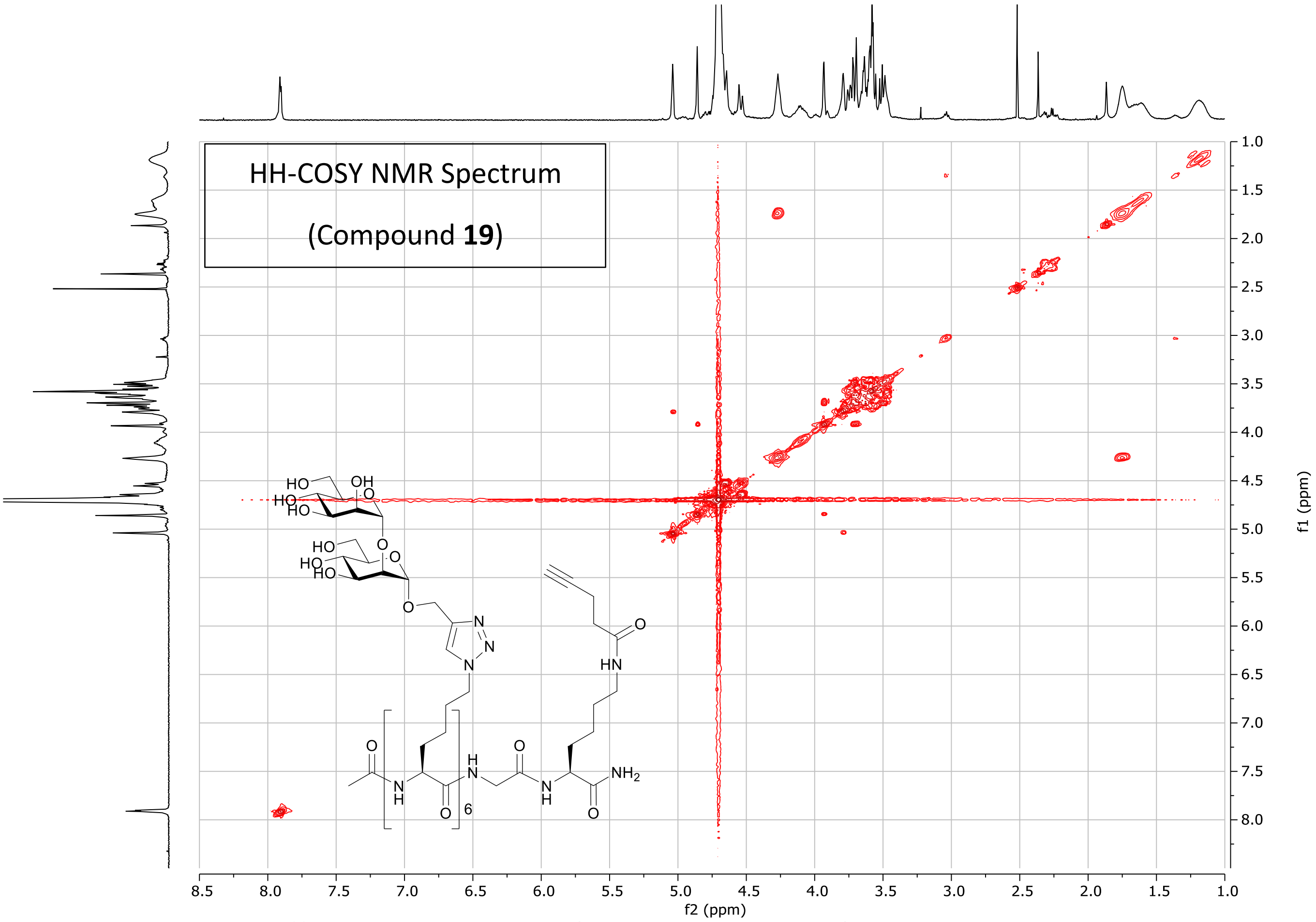
HH-COSY NMR Spectrum
(Compound **19**)
f2 (ppm)
f1 (ppm)

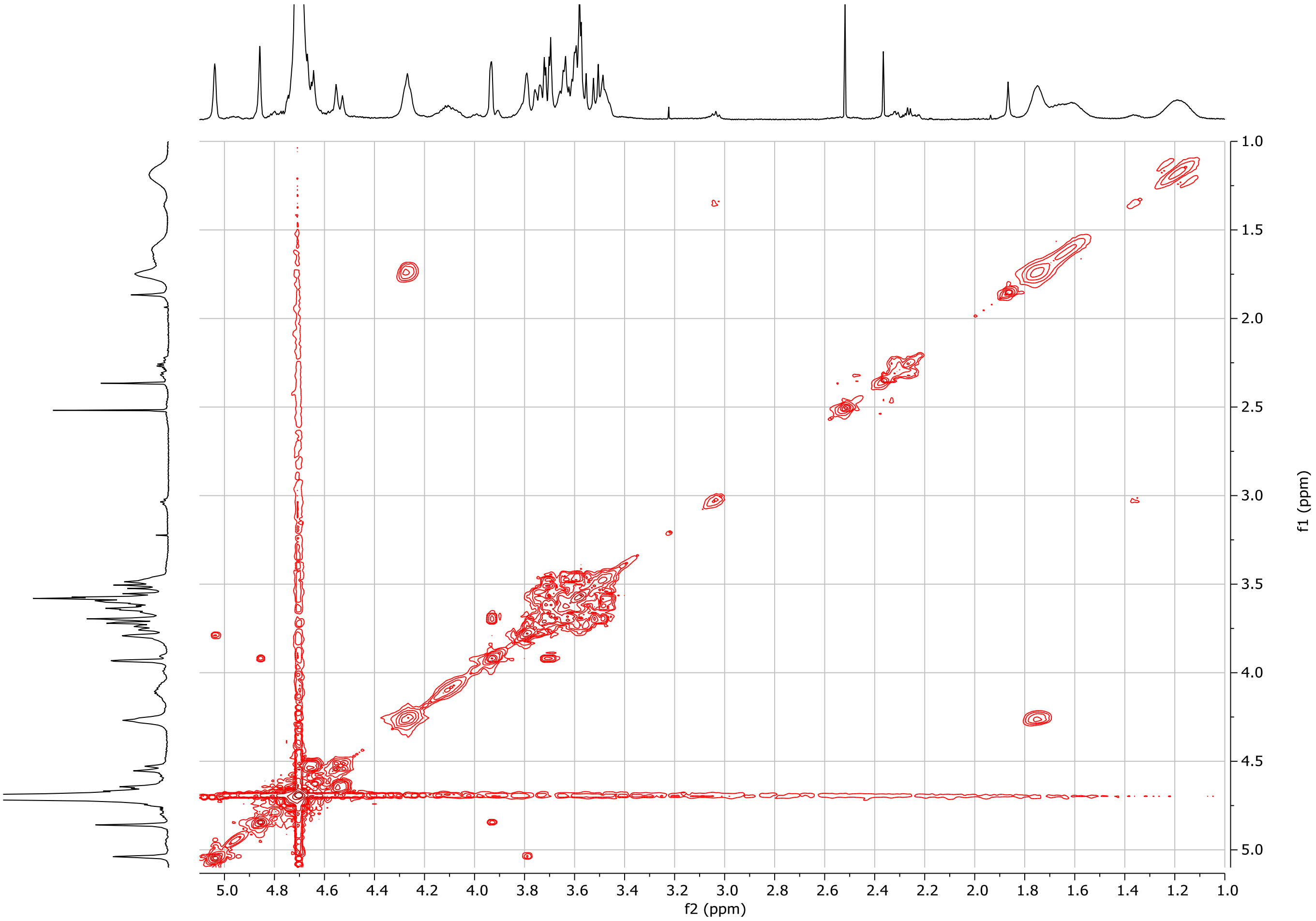
f2 (ppm)
f1 (ppm)

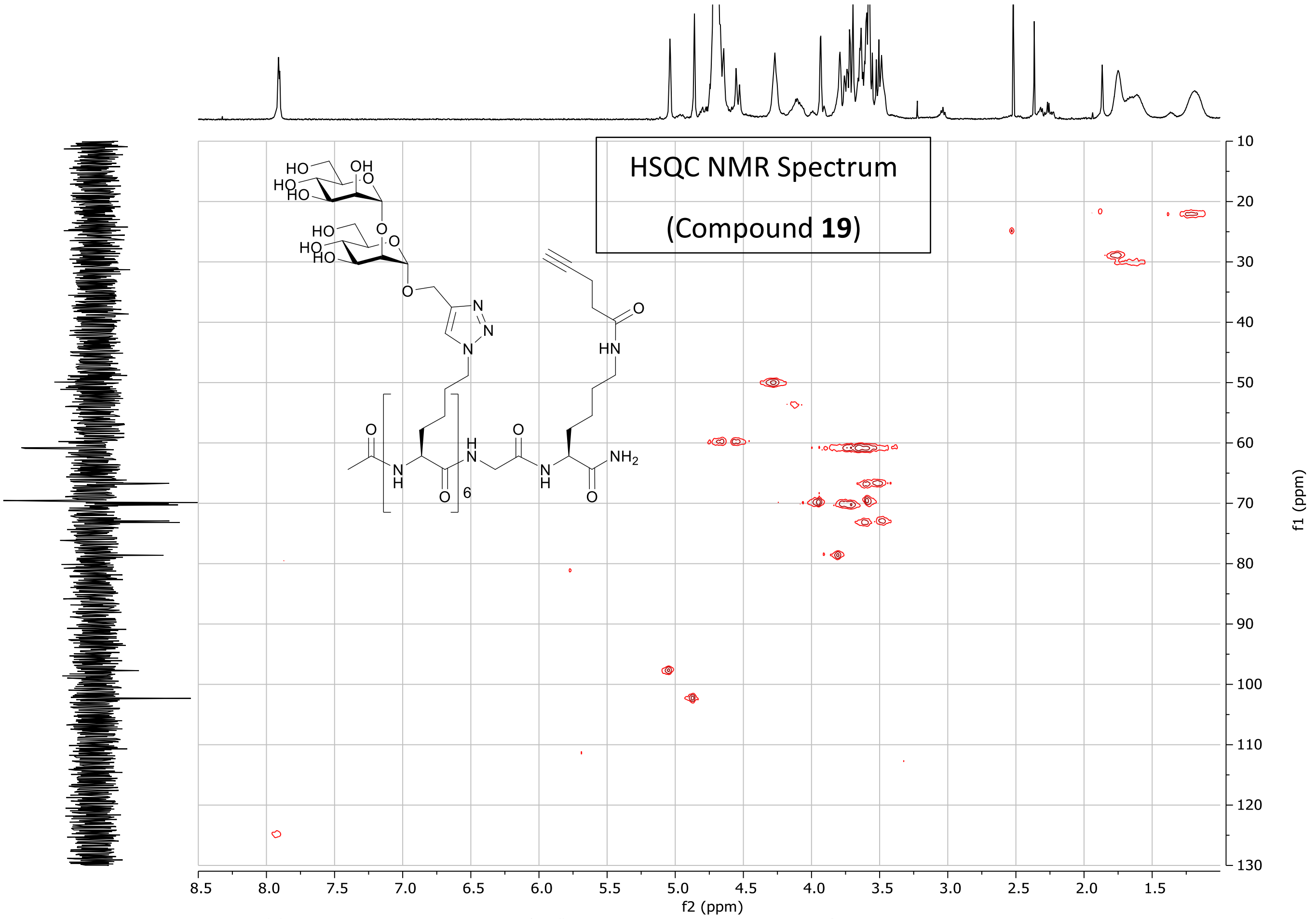
HSQC NMR Spectrum
(Compound **19**)
f1 (ppm)
f2 (ppm)

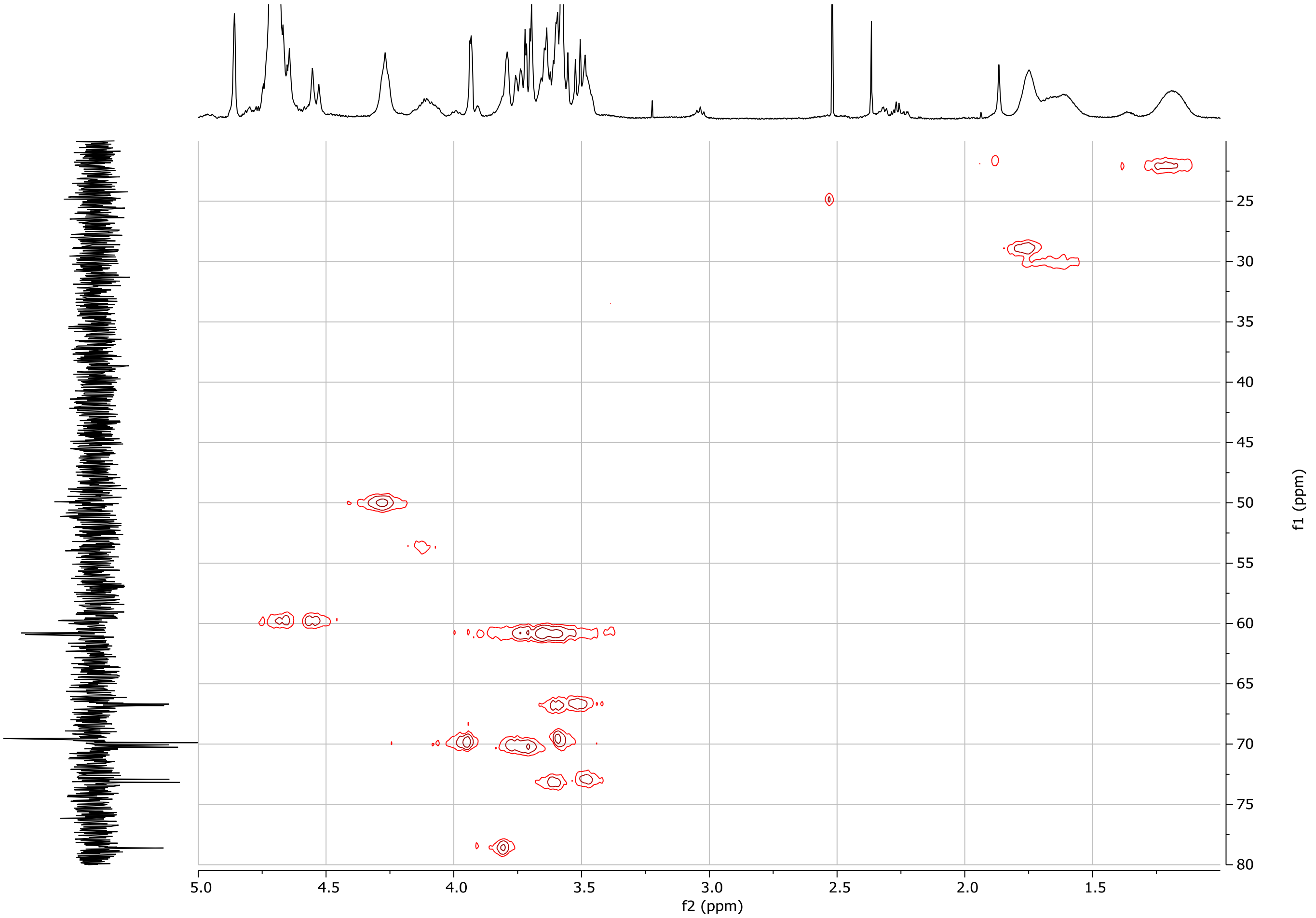
f1 (ppm)
f2 (ppm)

LC-MS Spectra; *(0 –> 50 % ACN, 13 min)*; (Compound **19**)

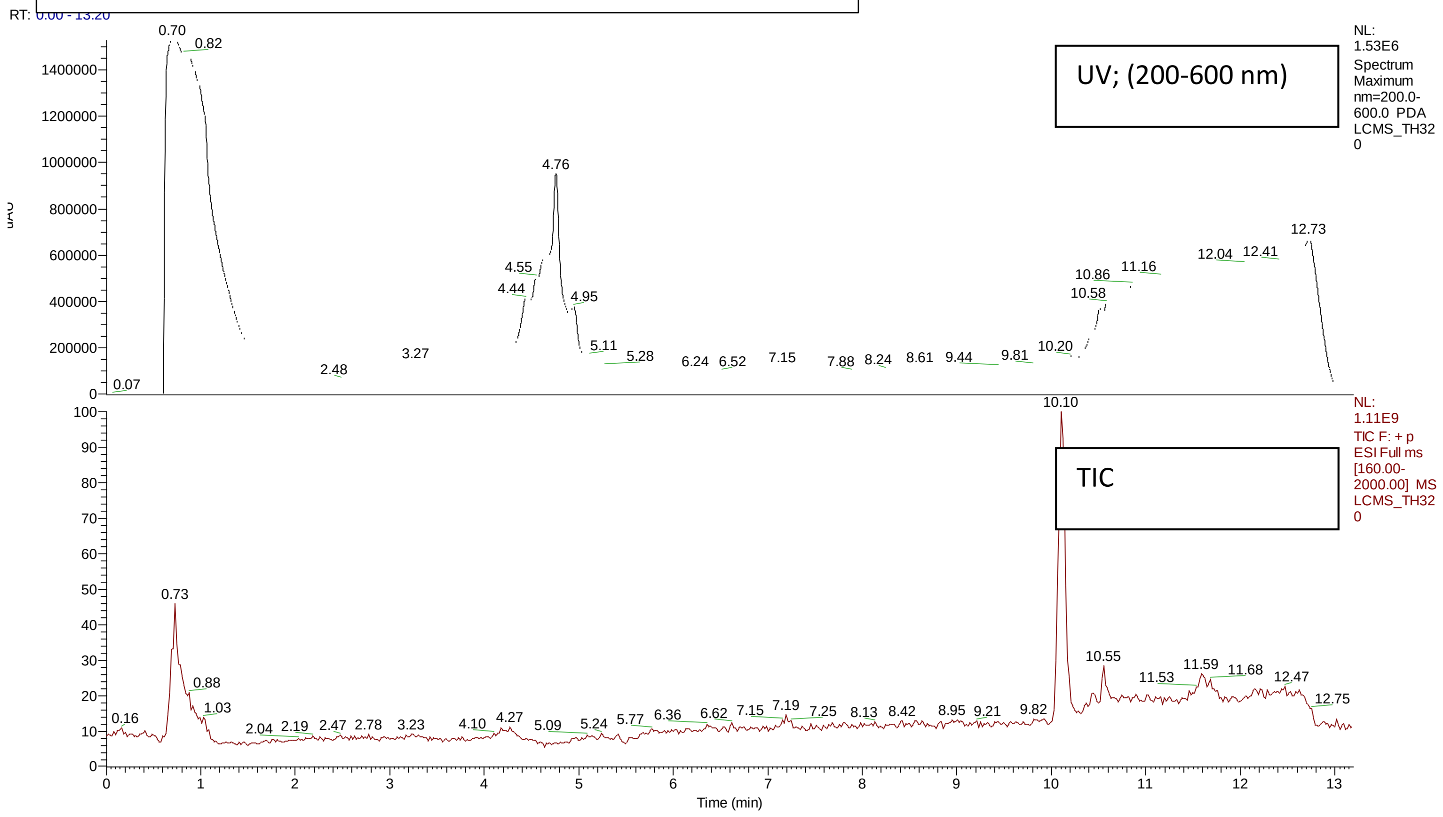

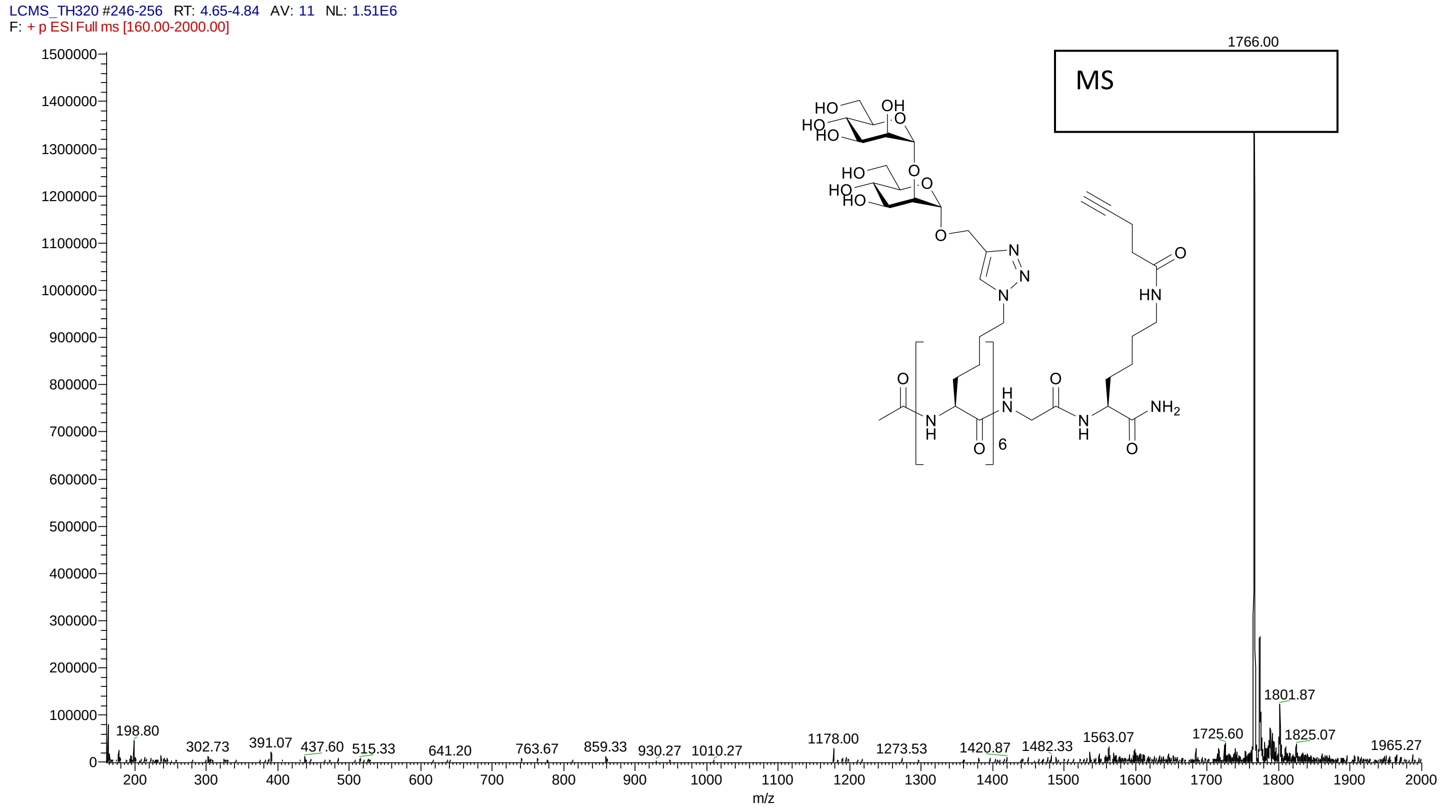

20

^{1}H NMR Spectrum

(Compound **20**)

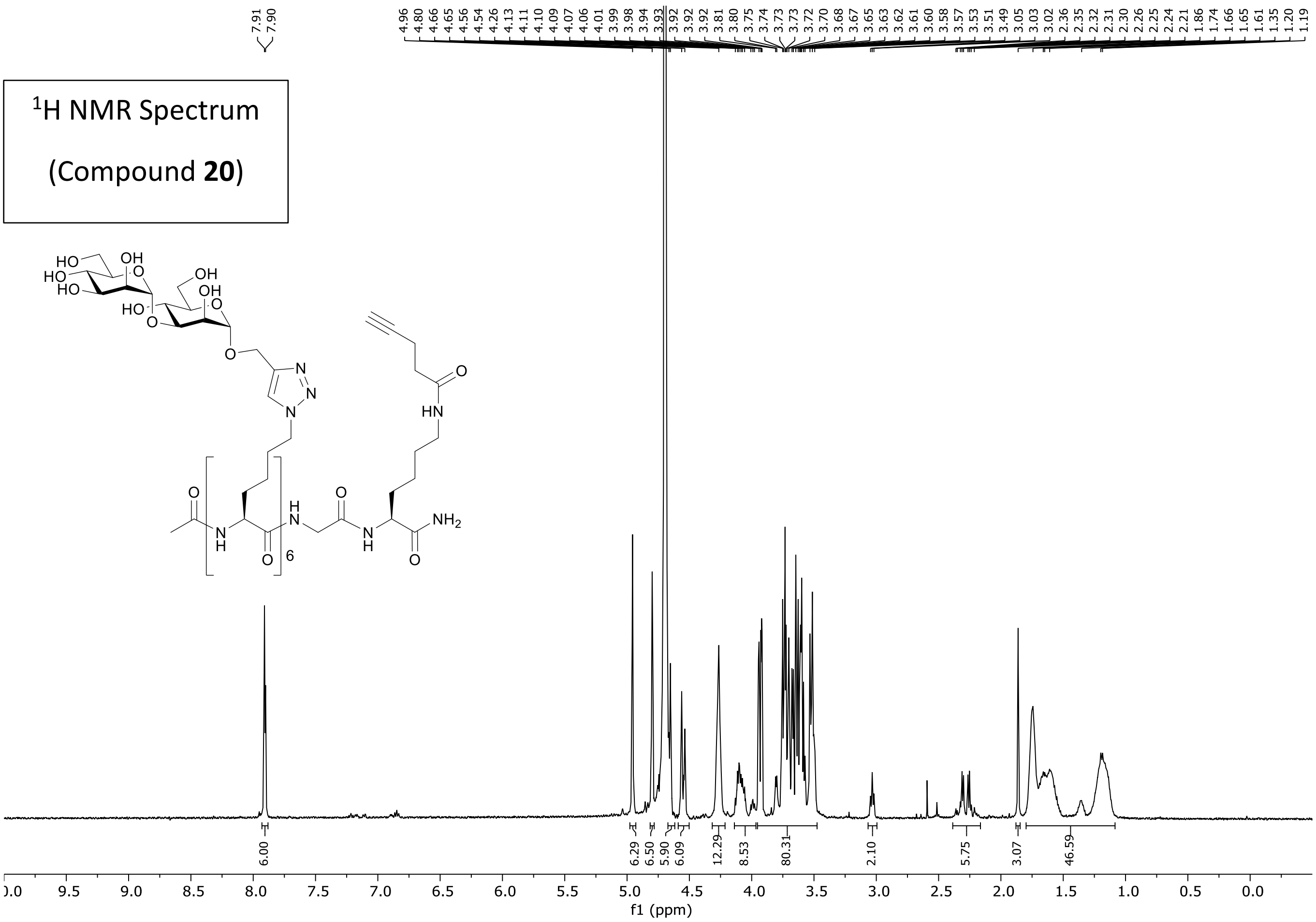

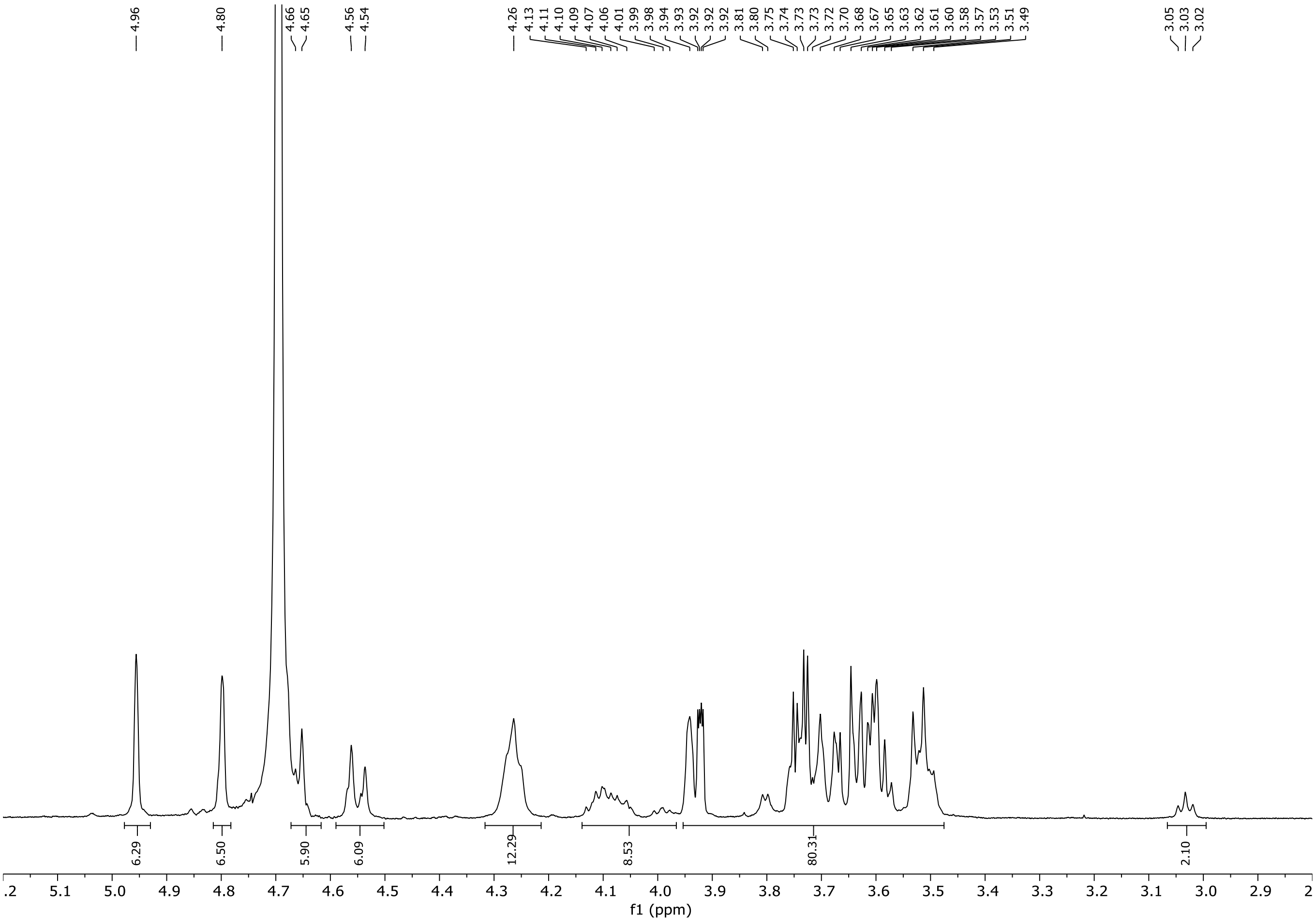

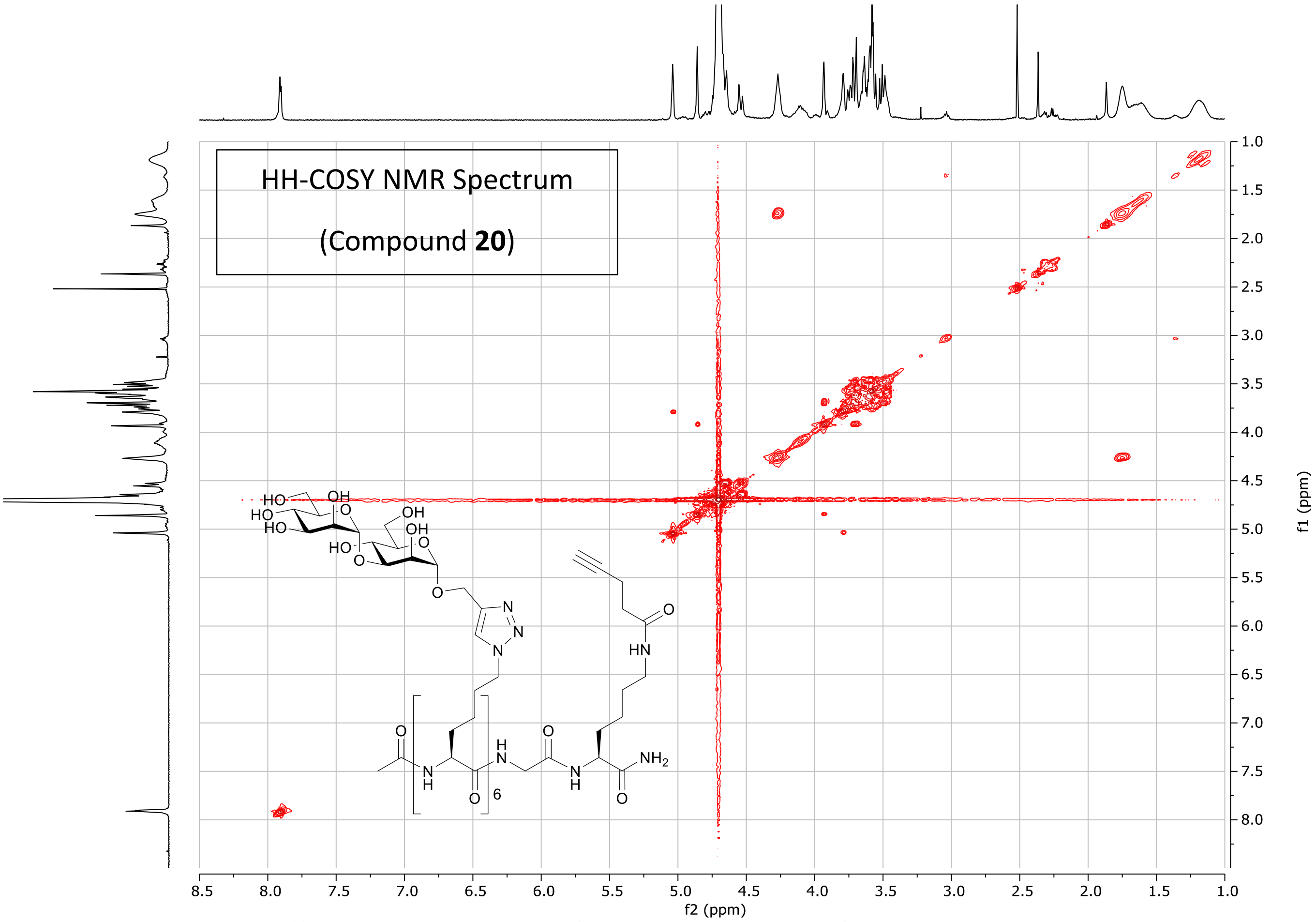
HH-COSY NMR Spectrum
(Compound 20)
f2 (ppm)
f1 (ppm)

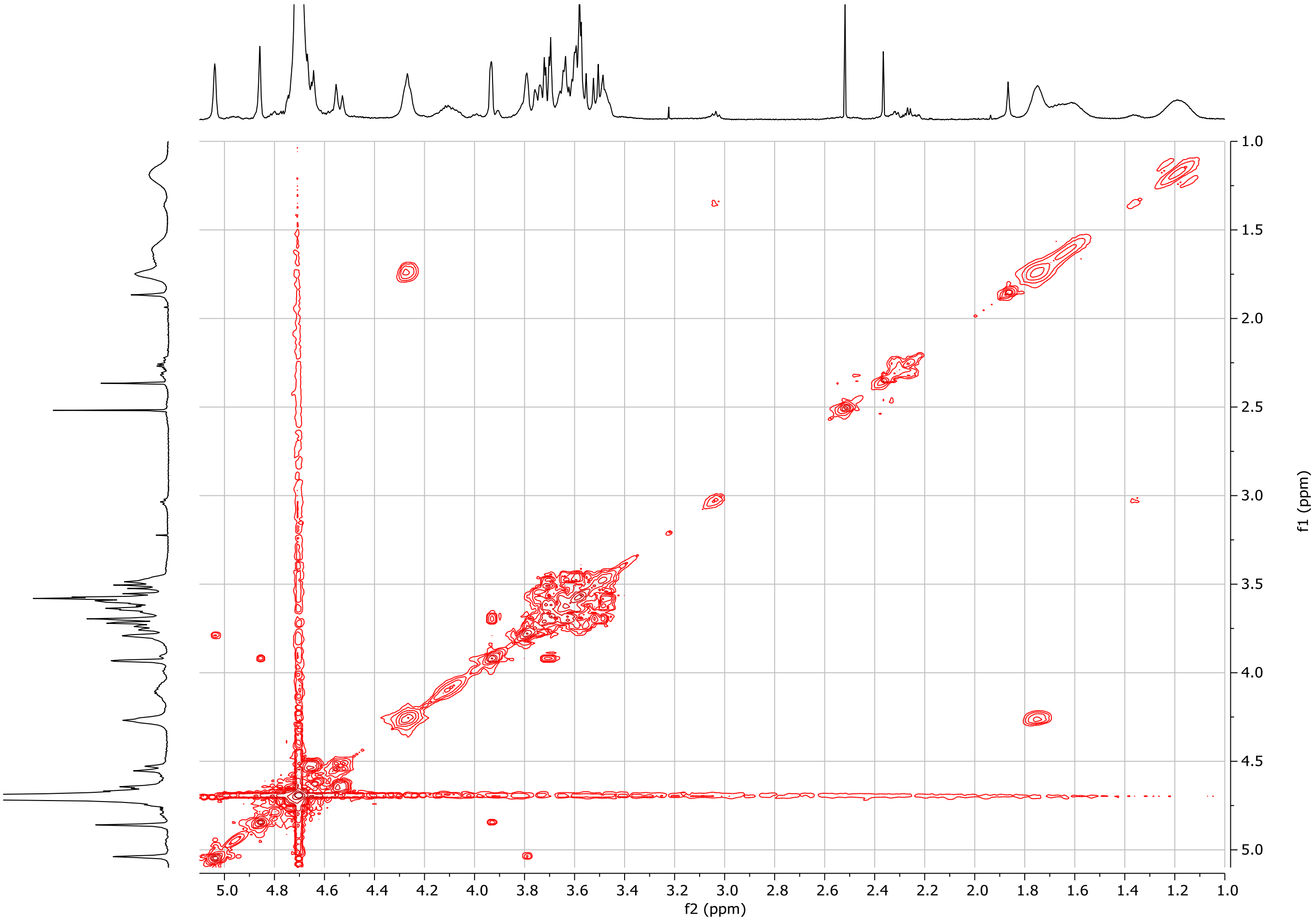
f2 (ppm)
f1 (ppm)

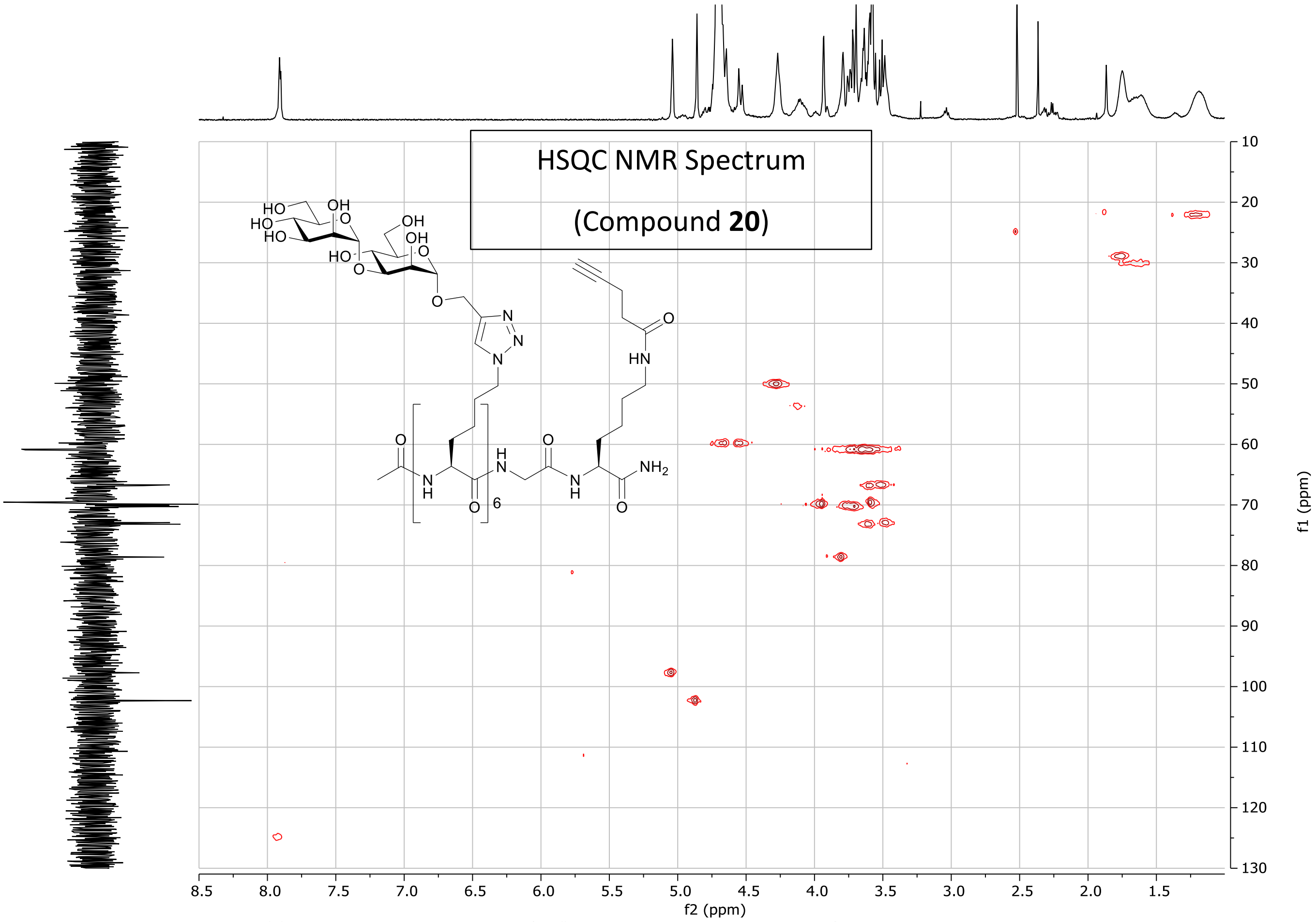
HSQC NMR Spectrum
(Compound **20**)
f1 (ppm)
f2 (ppm)

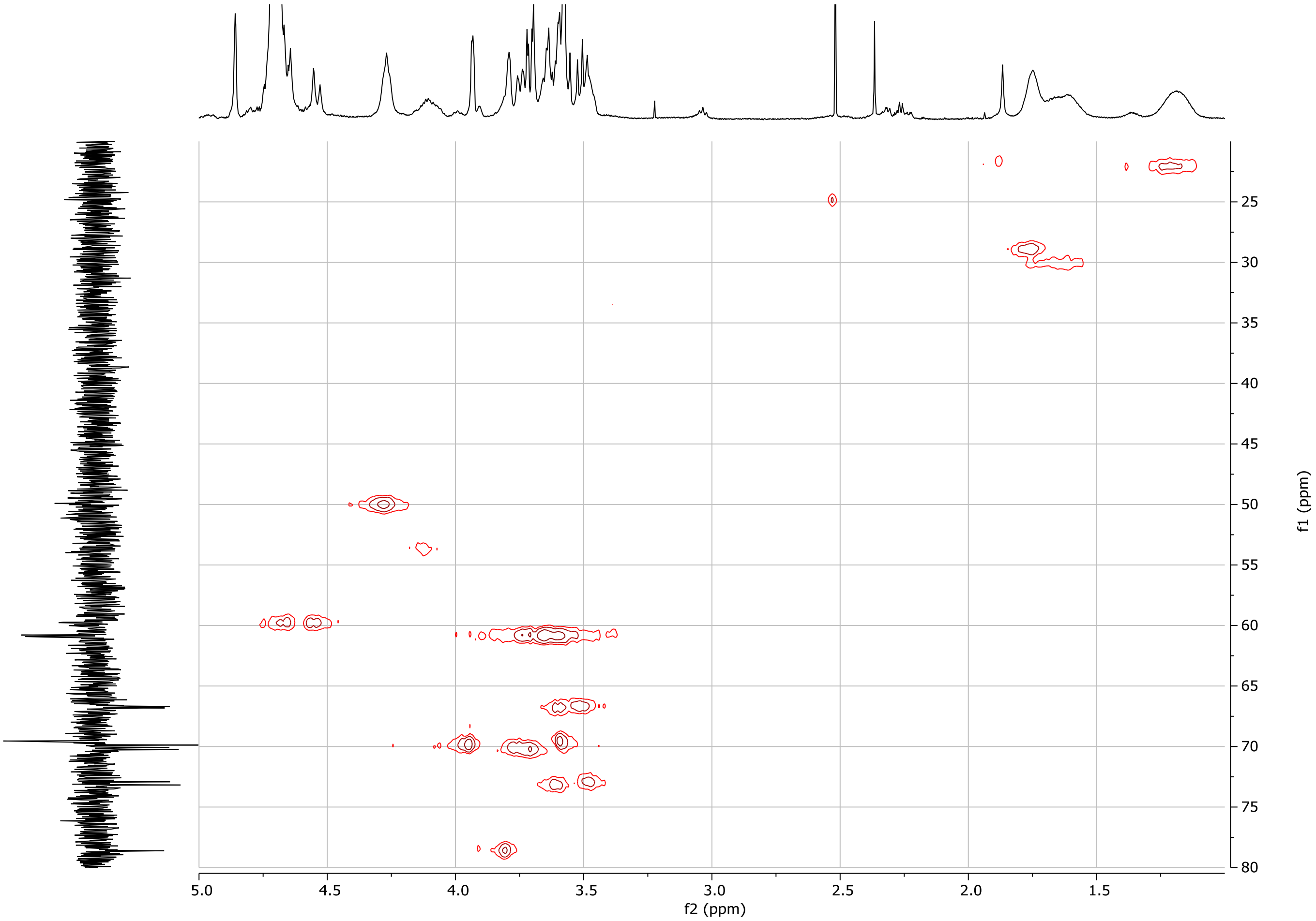
f1 (ppm)
f2 (ppm)

LC-MS Spectra; *(0 –> 50 % ACN, 13 min)*; (Compound **20**)

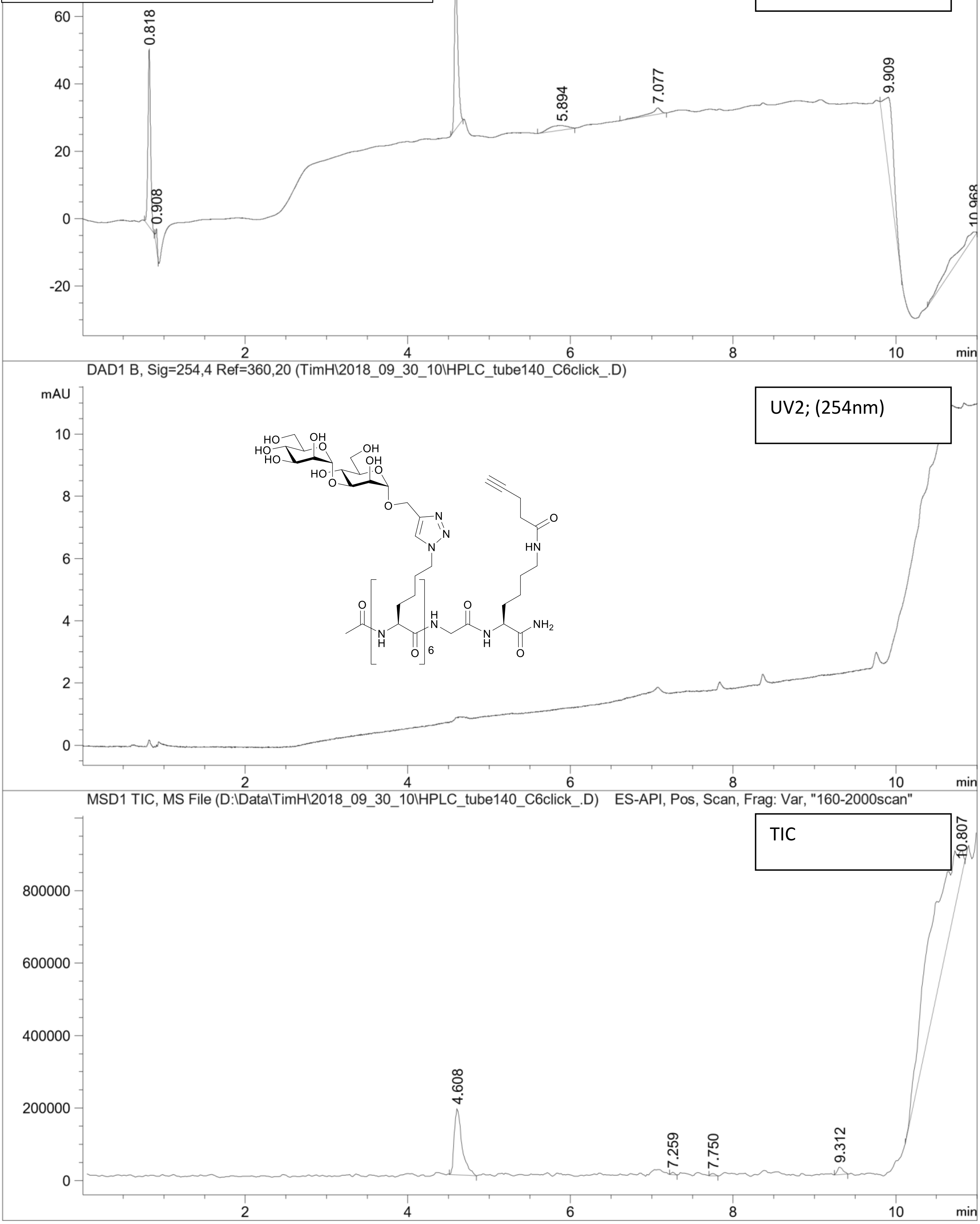

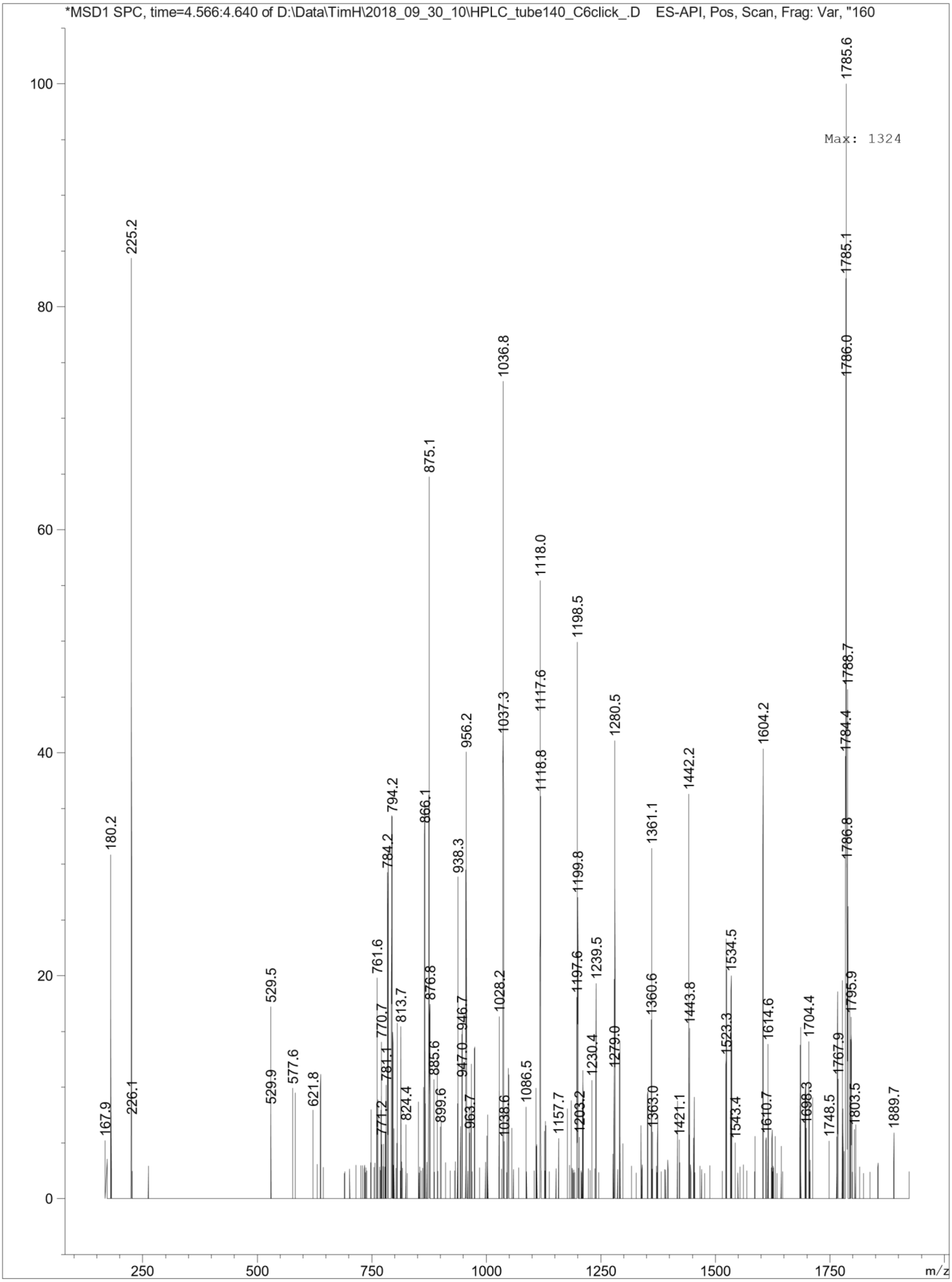
*MSD1 SPC, time=4.566:4.640 of D:\Data\TimH\2018_09_30_10\HPLC_tube140_C6click_.D ES-API, Pos, Scan, Frag: Var, "160
Max: 1324
1785.6
225.2
1785.1
1786.0
1036.8
875.1
1118.0
1198.5
1788.7
1117.6
1280.5
1037.3
956.2
1784.4
1604.2
1442.2
1118.8
794.2
866.1
1361.1
180.2
784.2
1786.8
938.3
1199.8
1534.5
1239.5
761.6
876.8
1197.6
1795.9
529.5
1028.2
1360.6
1443.8
813.7
946.7
1614.6
1704.4
770.7
1523.3
1230.4
947.0
885.6
1279.0
781.1
1767.9
577.6
621.8
529.9
226.1
1363.0
1698.3
1803.5
1889.7
1086.5
167.9
771.2
824.4
899.6
963.7
1038.6
1157.7
1203.2
1421.1
1543.4
1610.7
1748.5
100
80
60
40
20
0
250
500
750
1000
1250
1500
1750
m/z

21

LC-MS Spectra; *(0 –> 50 % ACN, 13 min);* (Compound **21**)

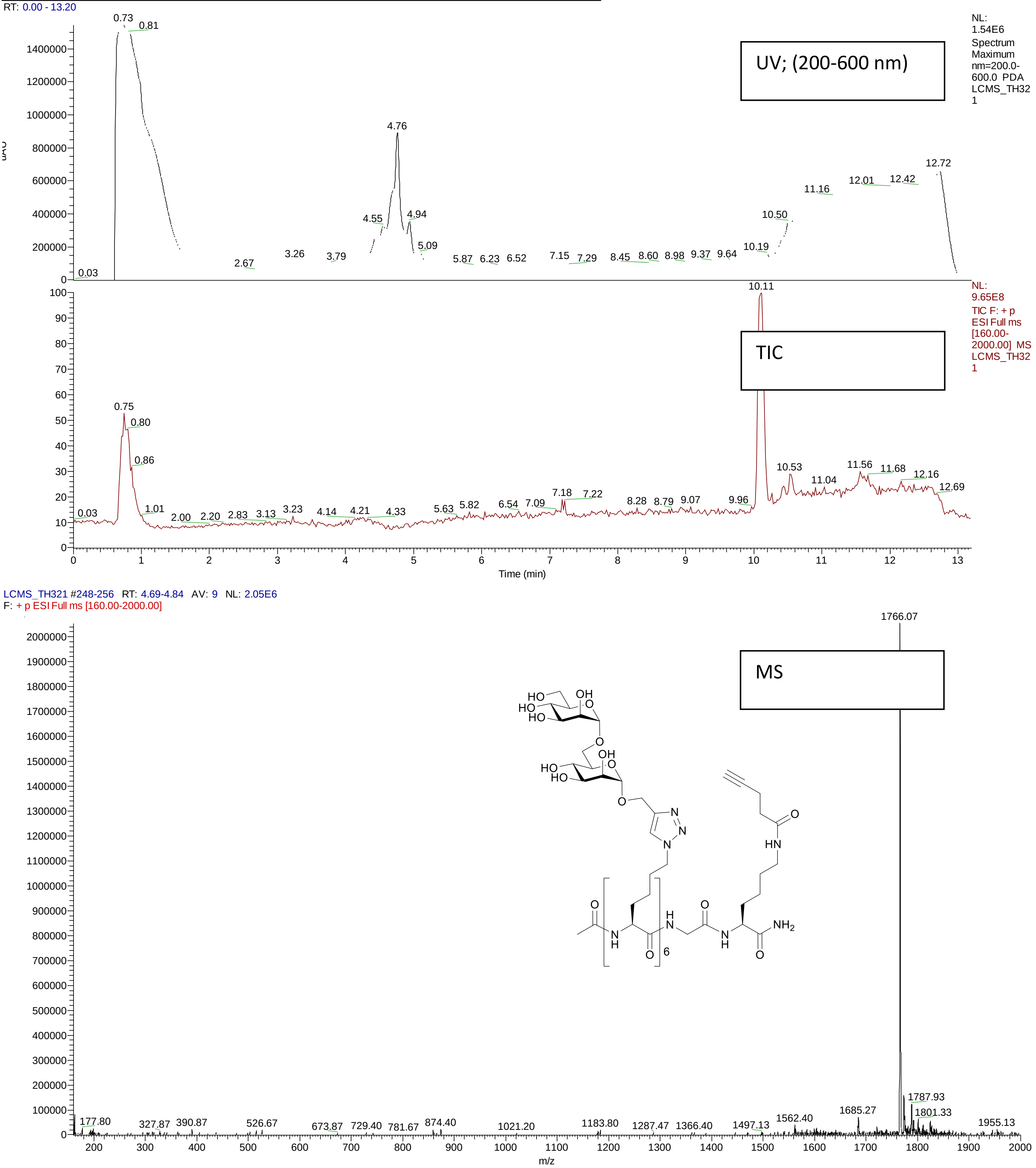

22

^{1}H NMR Spectrum

(Compound **22**)

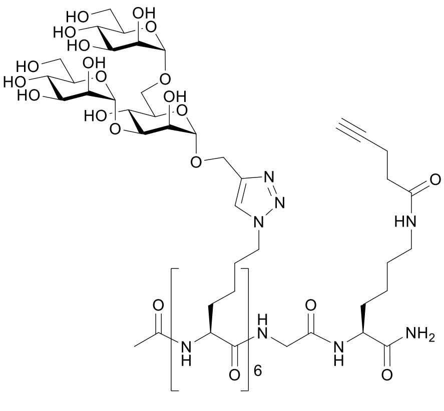

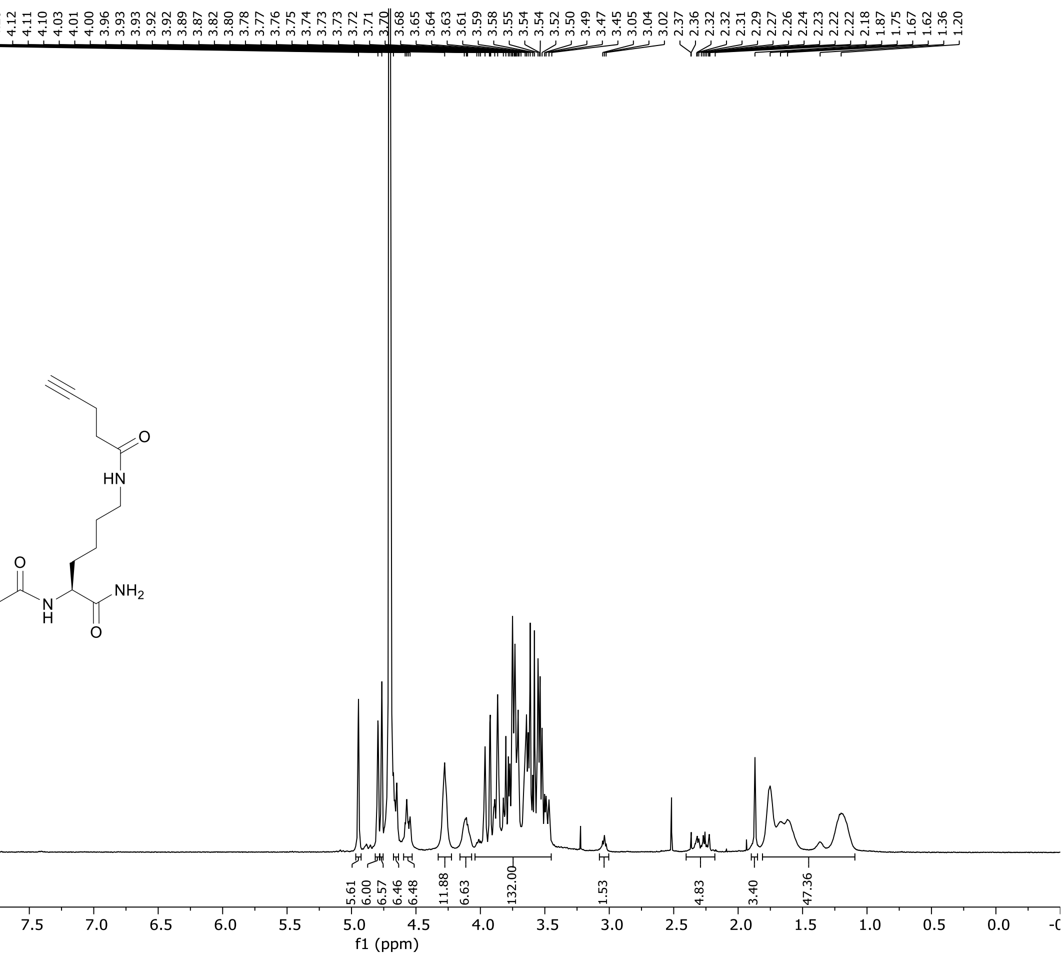

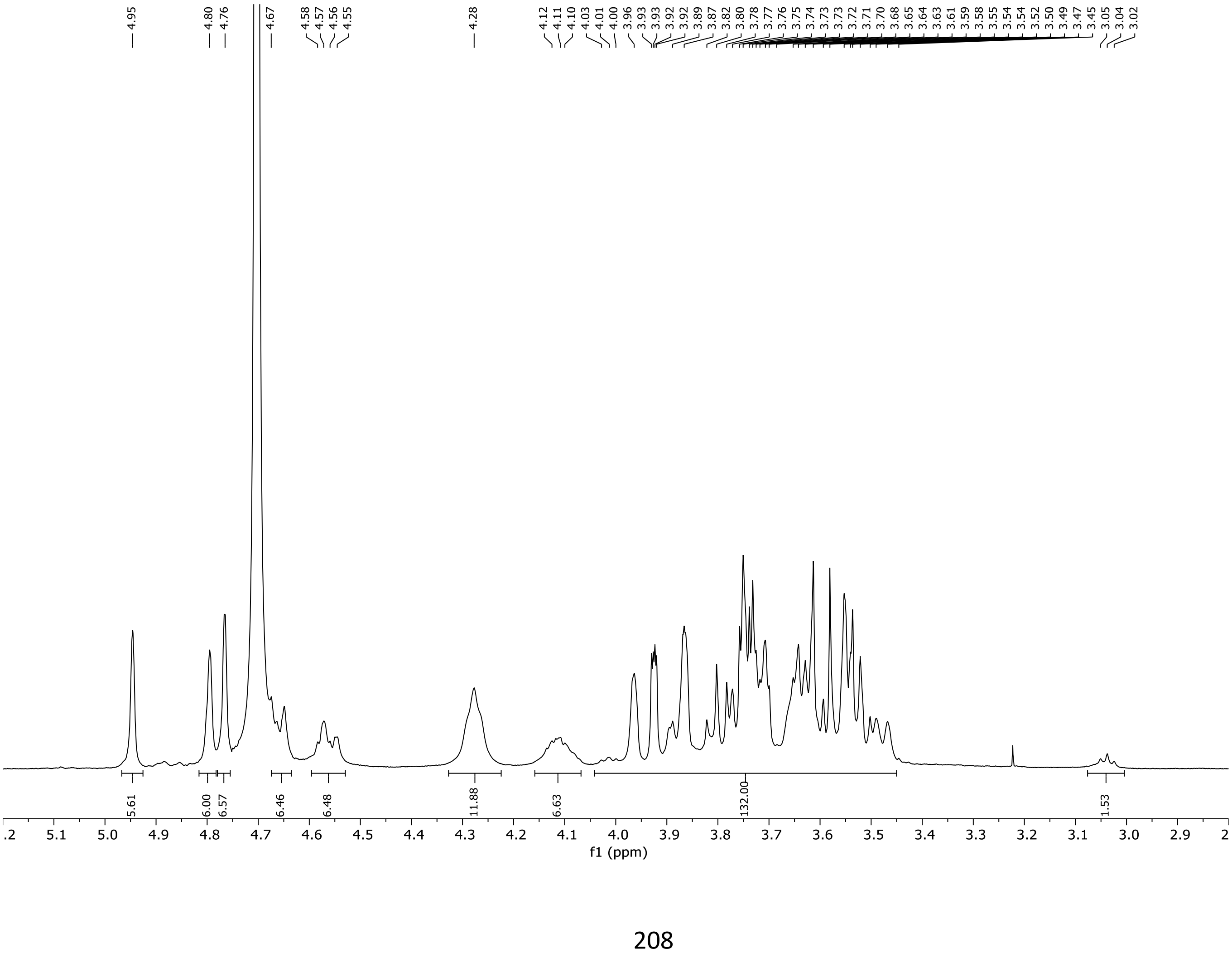

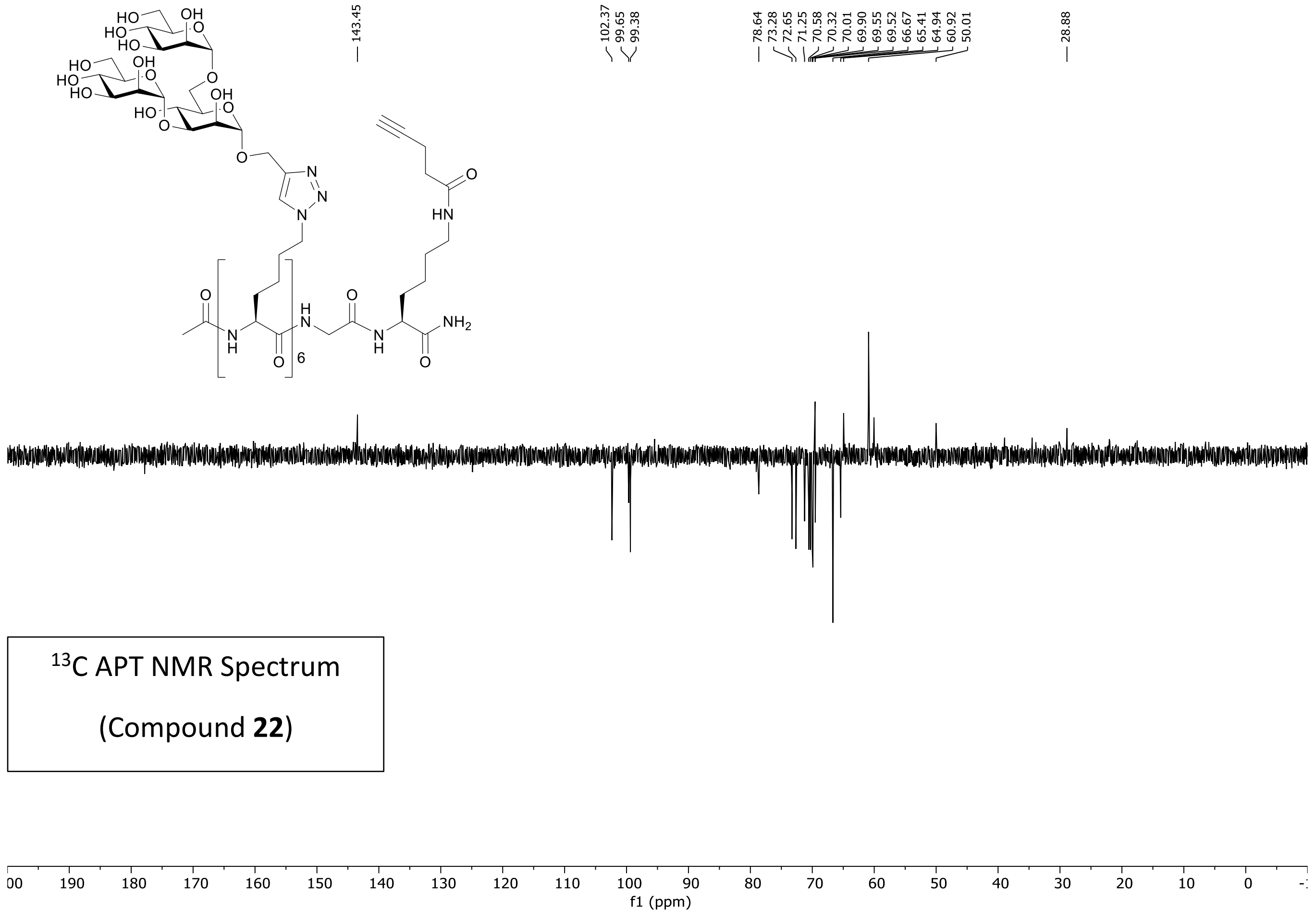

^{13}C APT NMR Spectrum

(Compound **22**)

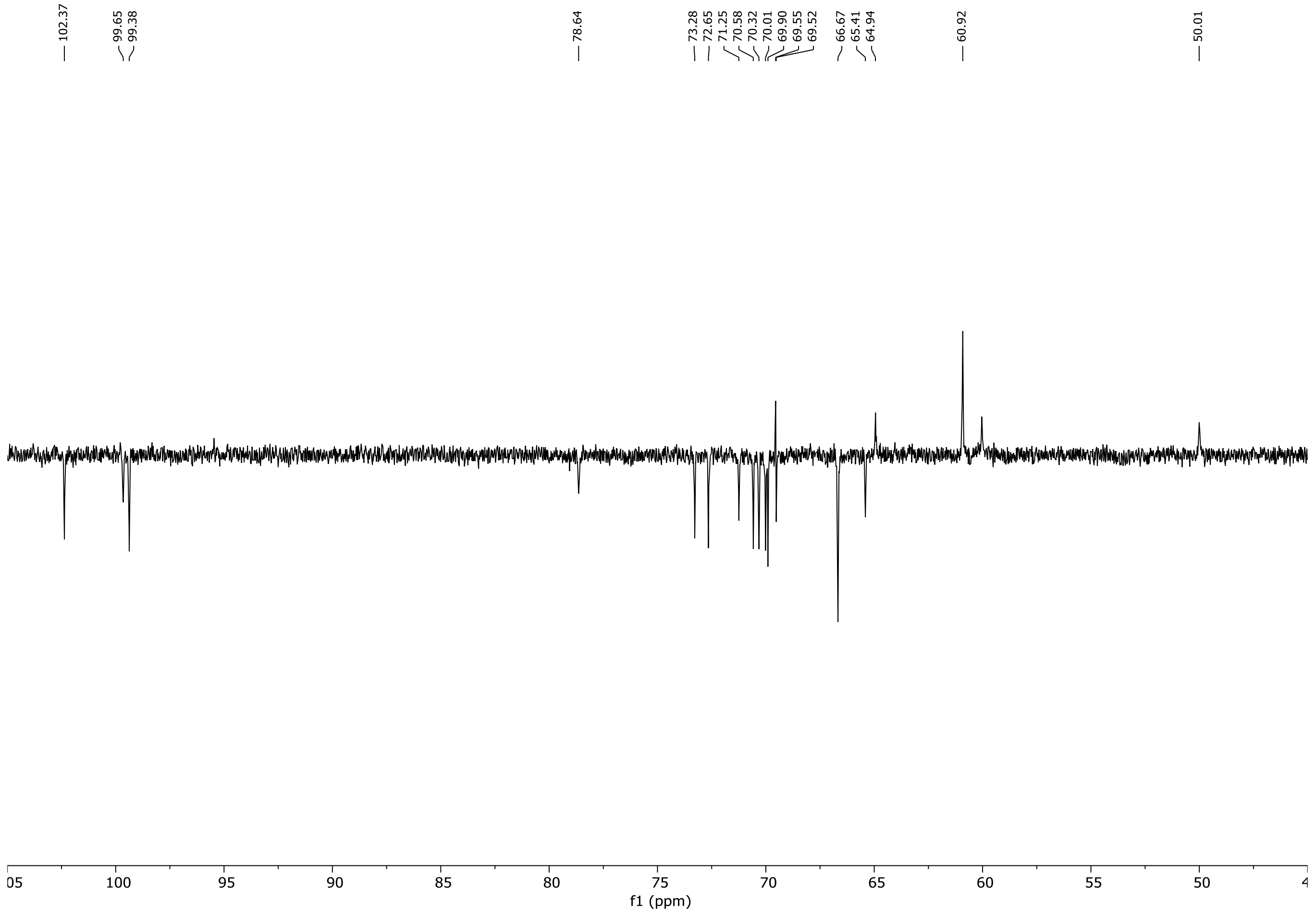

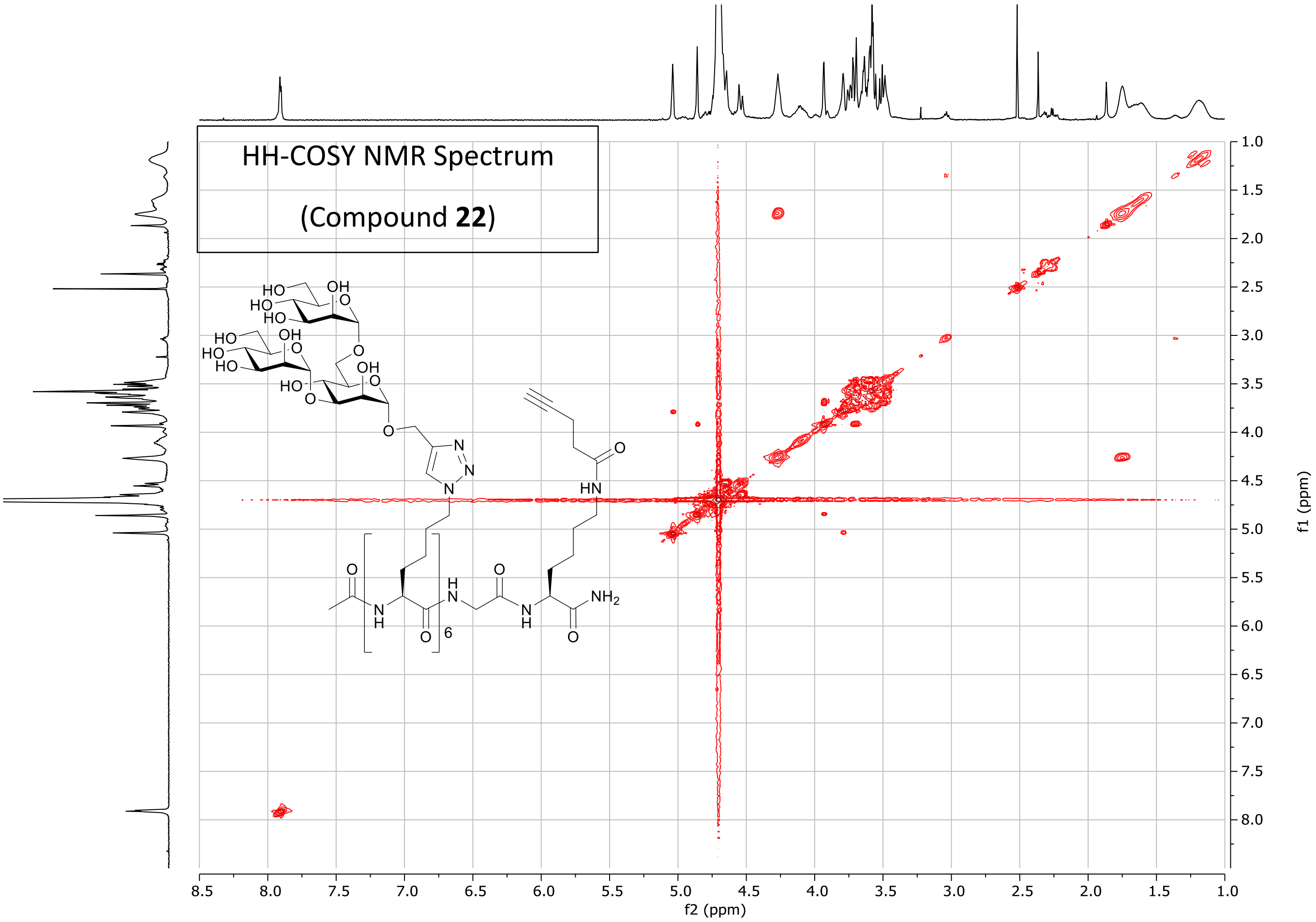
HH-COSY NMR Spectrum
(Compound **22**)
f2 (ppm)
f1 (ppm)

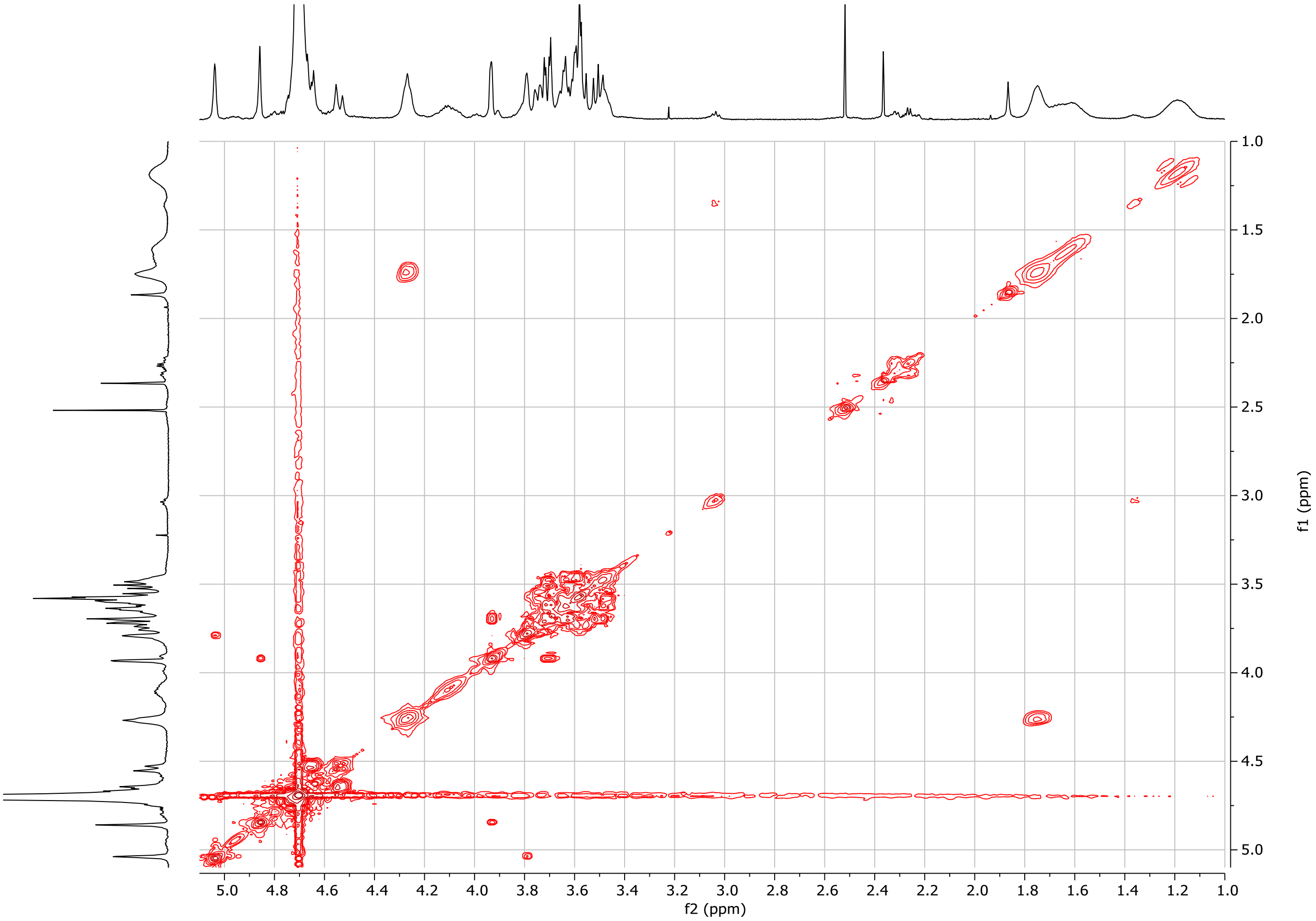
f2 (ppm)
f1 (ppm)

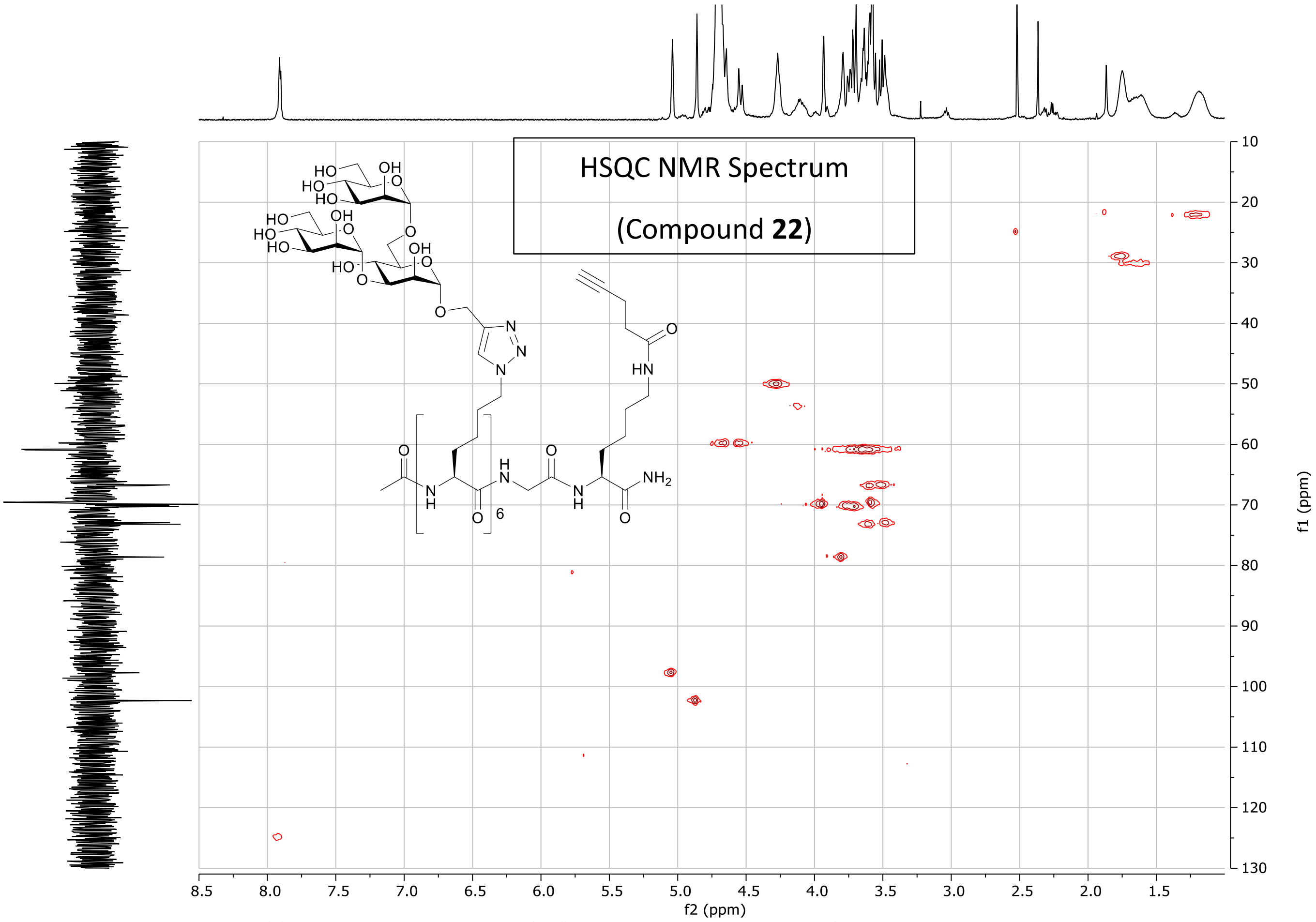
HSQC NMR Spectrum
(Compound 22)
f2 (ppm)
f1 (ppm)

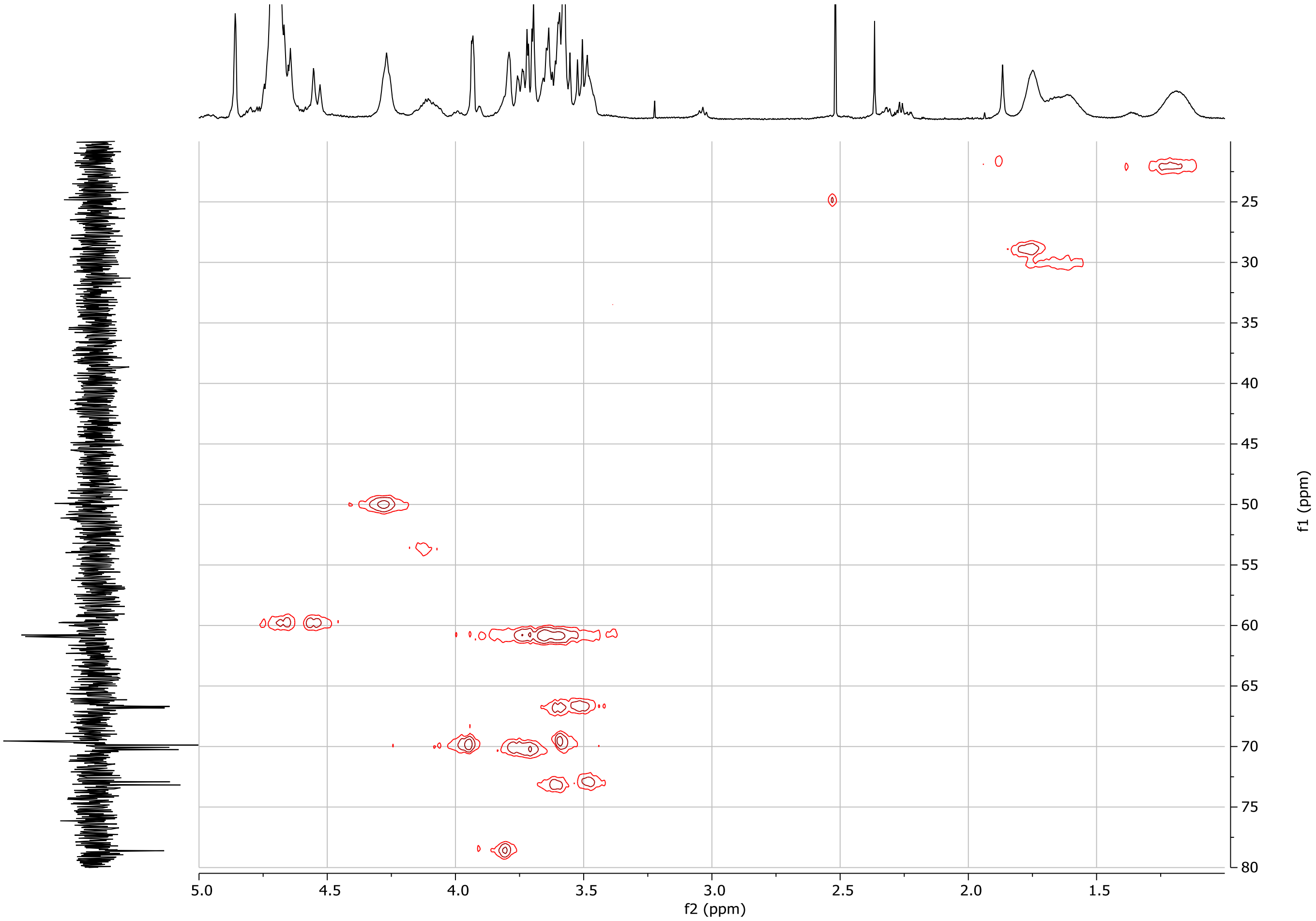
f2 (ppm)
f1 (ppm)

LC-MS Spectra; *(0 –> 50 % ACN, 13 min);* (Compound **22**)

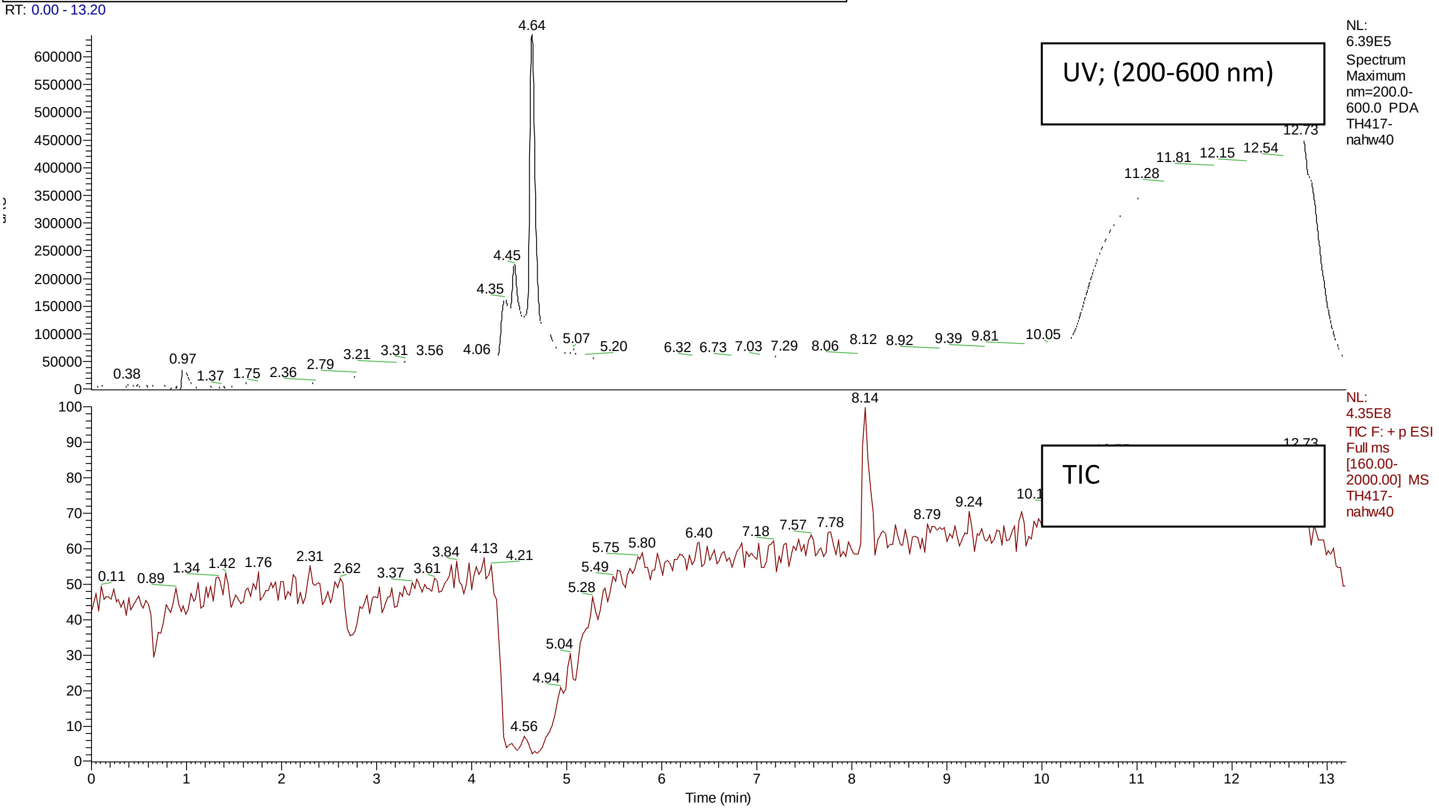

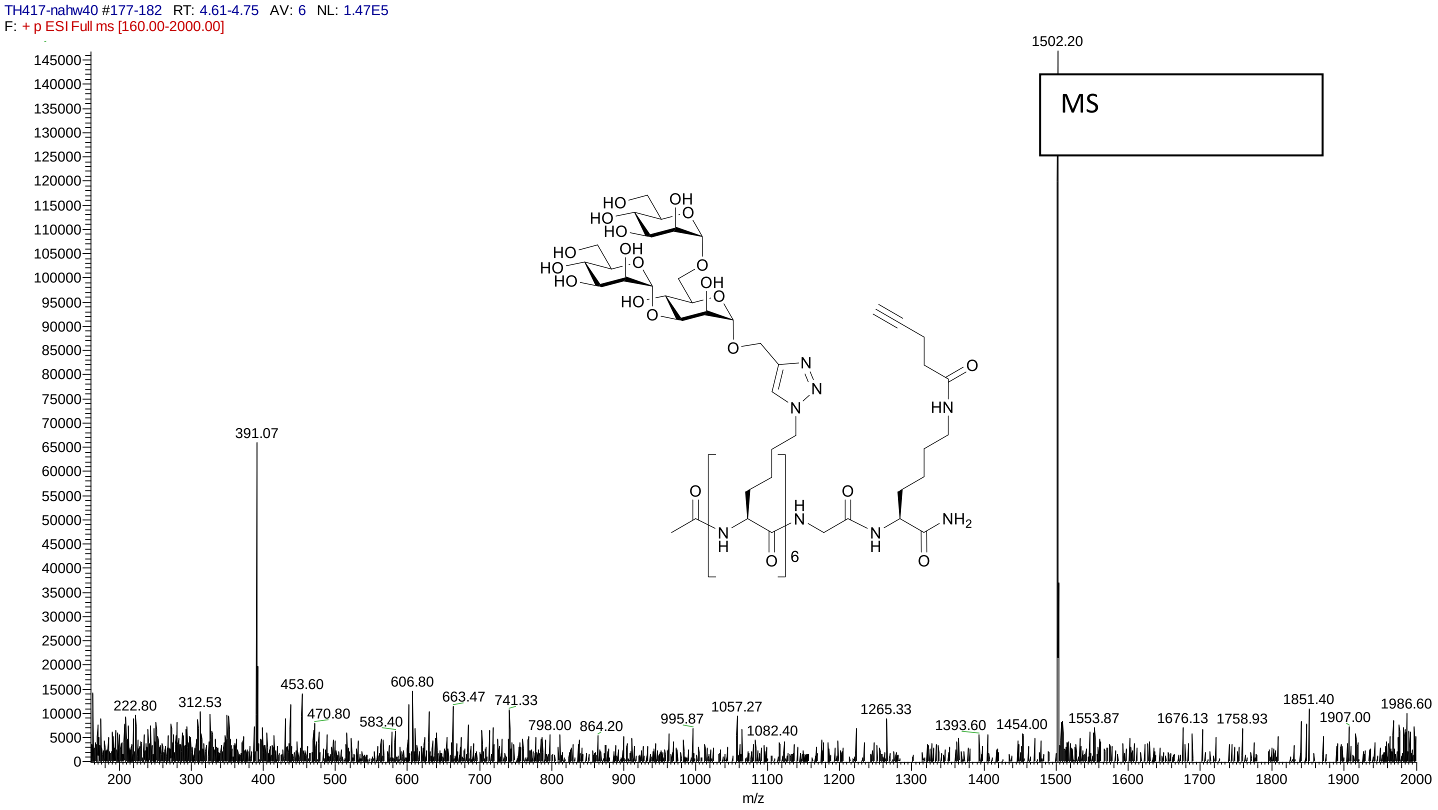

Spectra of gp100 peptides:

26

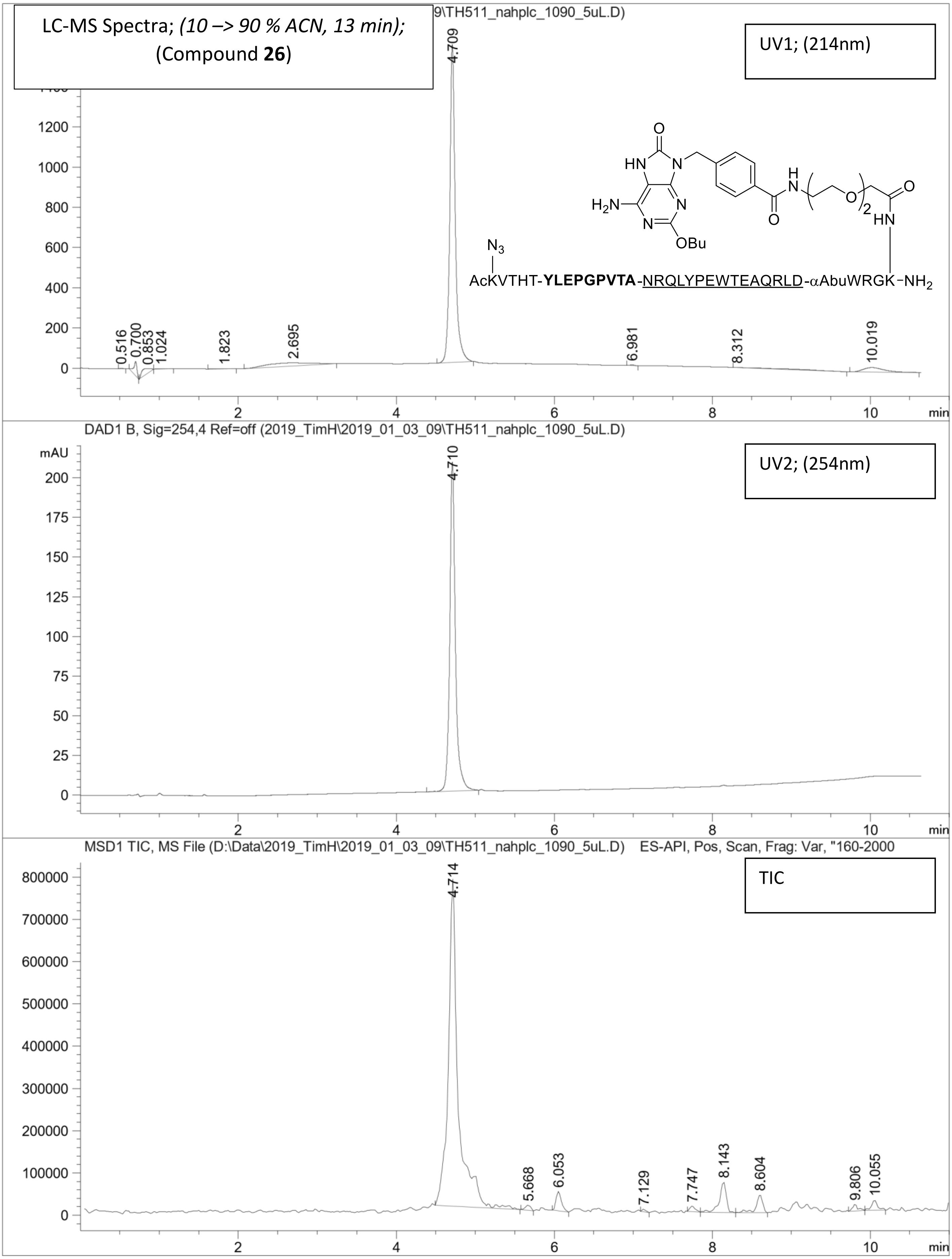

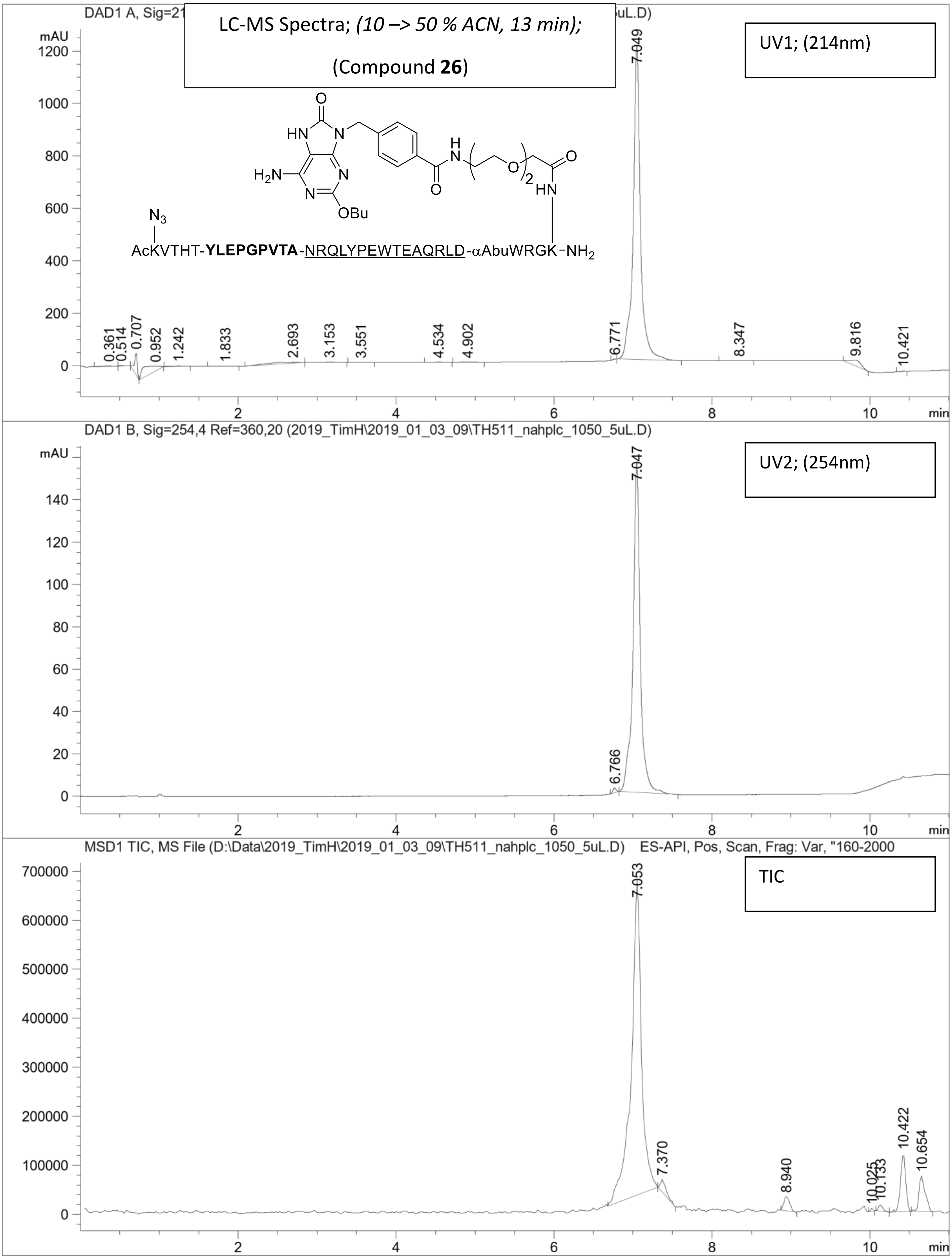
LC-MS Spectra; (10 –> 50 % ACN, 13 min);
(Compound 26)
UV1; (214nm)
DAD1 A, Sig=21
uL.D)
AcKVTHT-YLEPGPVTA-NRQLYPEWTEAQRLD-αAbuWRGK-NH2
N3
OBu
mAU
min
0.361
0.514
0.707
0.952
1.242
1.833
2.693
3.153
3.551
4.534
4.902
6.771
7.049
8.347
9.816
10.421
UV2; (254nm)
DAD1 B, Sig=254,4 Ref=360,20 (2019_TimH\2019_01_03_09\TH511_nahplc_1050_5uL.D)
6.766
7.047
TIC
MSD1 TIC, MS File (D:\Data\2019_TimH\2019_01_03_09\TH511_nahplc_1050_5uL.D) ES-API, Pos, Scan, Frag: Var, "160-2000
7.053
7.370
8.940
10.025
10.133
10.422
10.654

matogram(s)

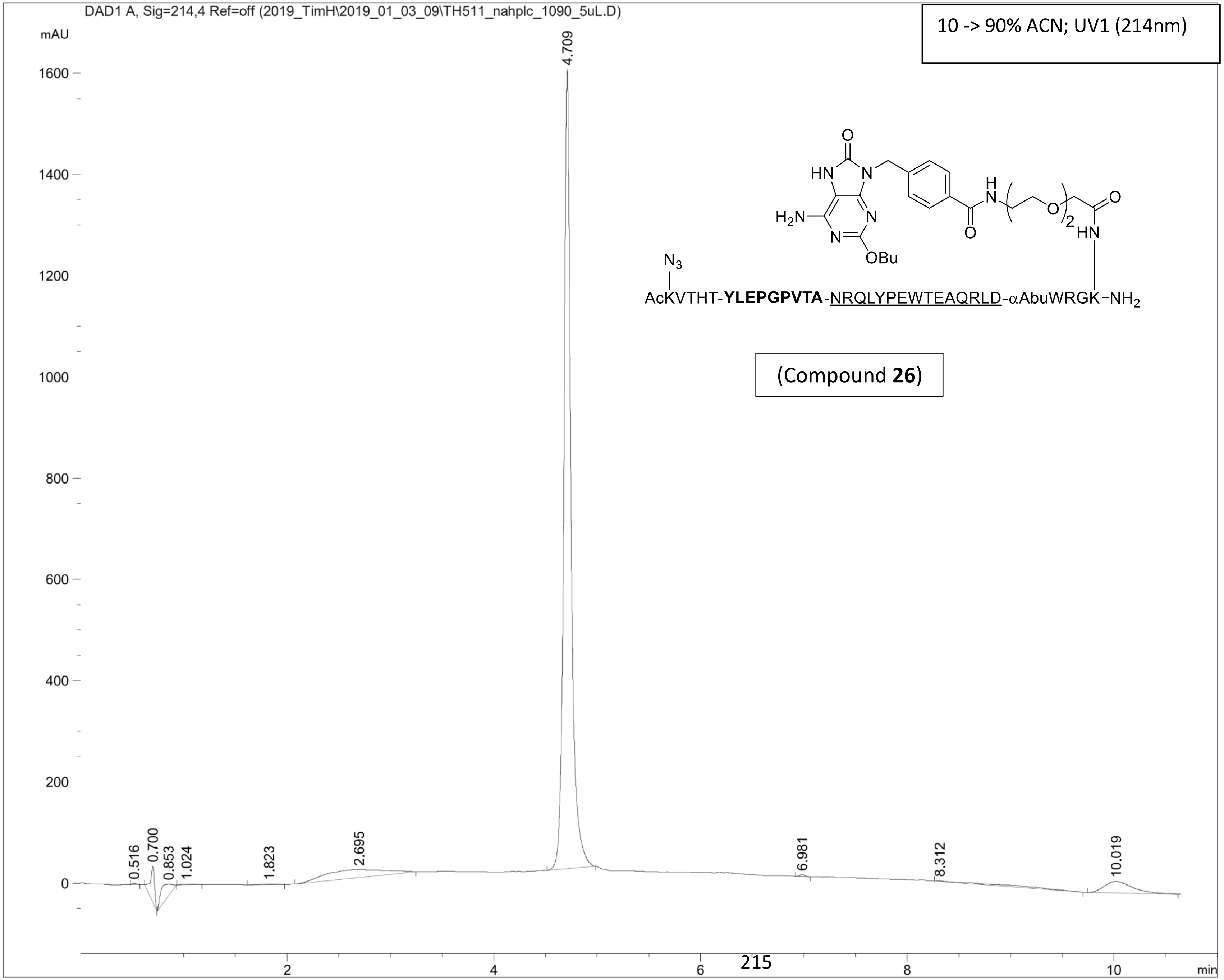

(Compound **26**)

4:07:05 PM SYSTEM

matogram(s)

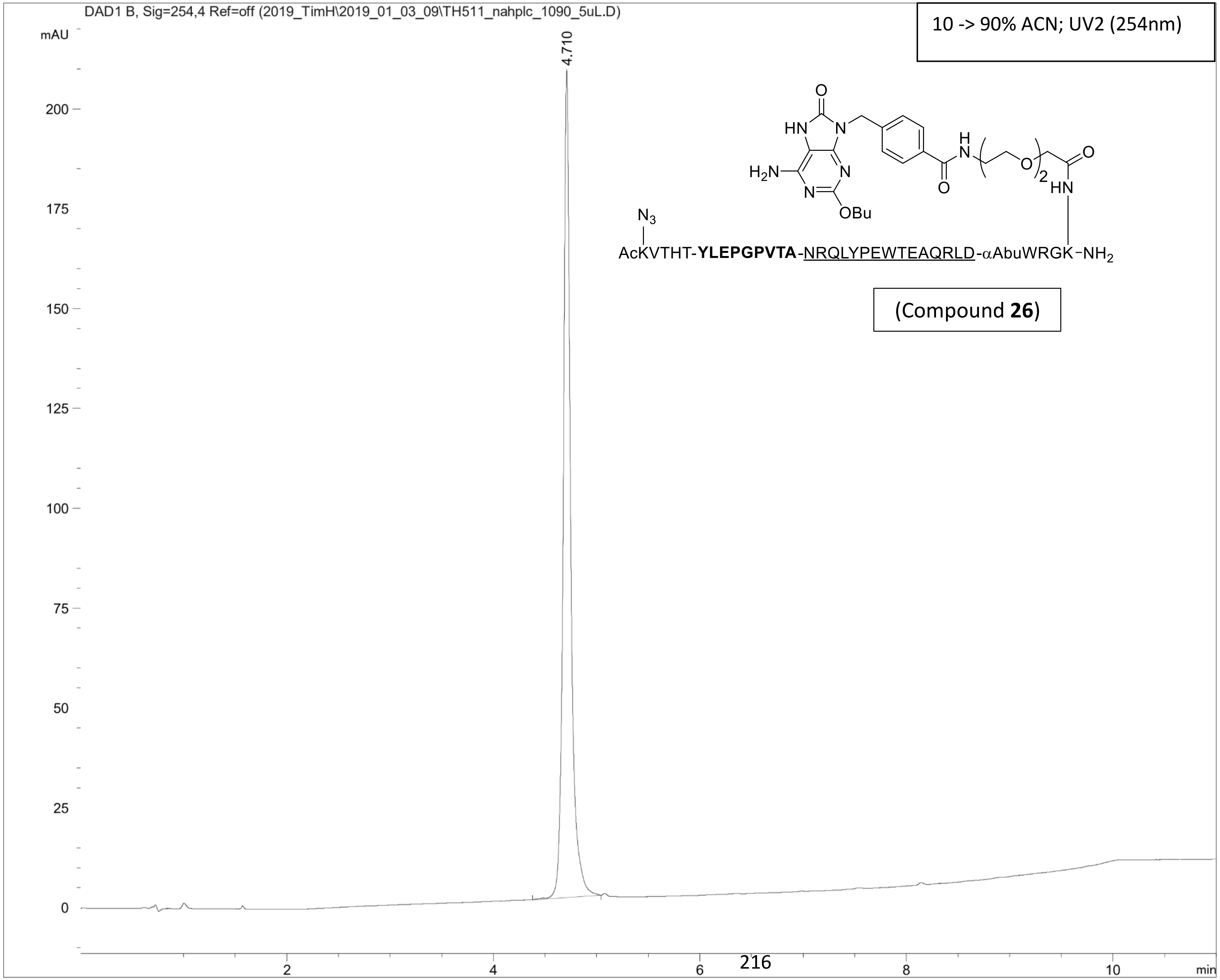

4:07:25 PM SYSTEM

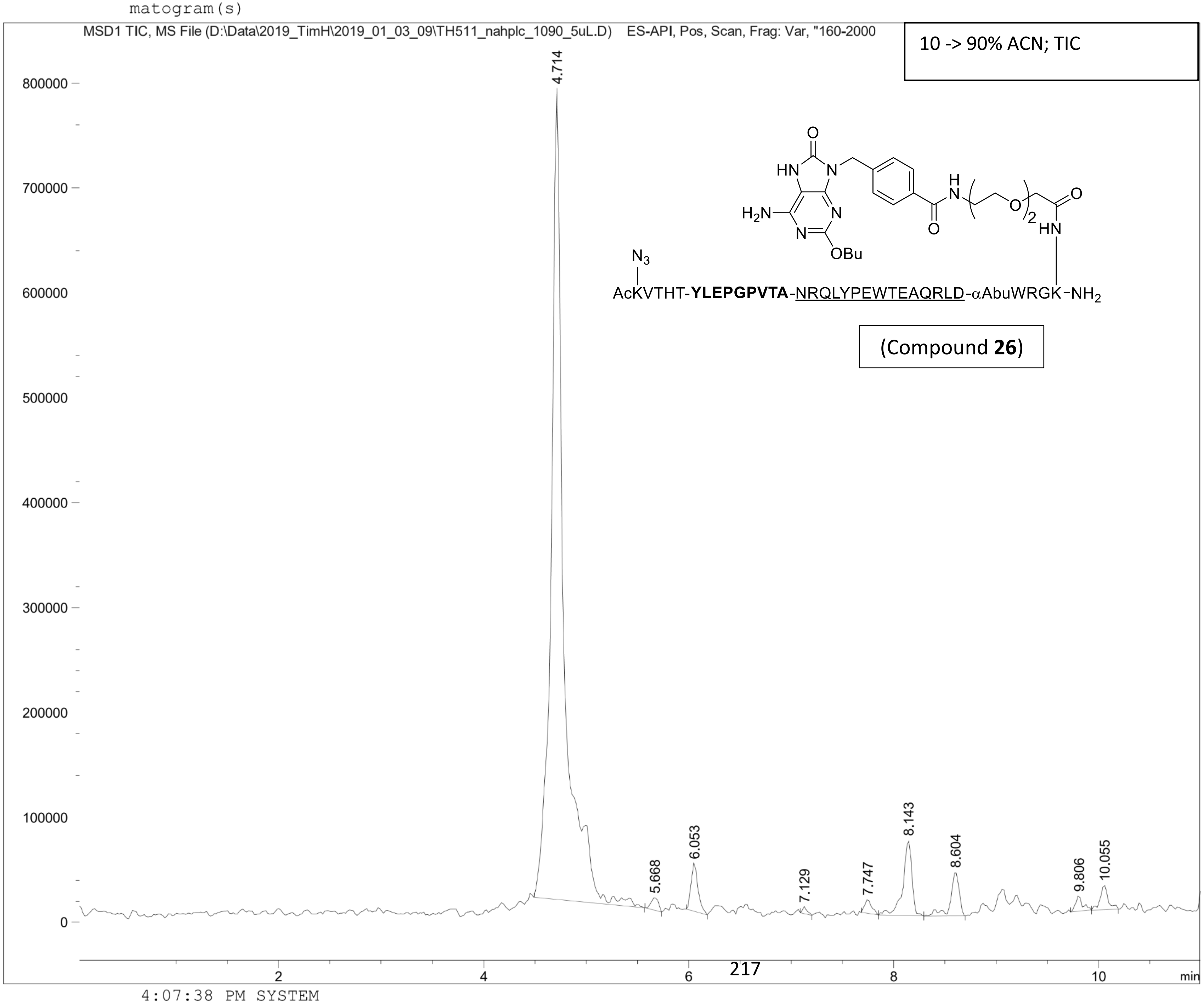
matogram(s)
MSD1 TIC, MS File (D:\Data\2019_TimH\2019_01_03_09\TH511_nahplc_1090_5uL.D) ES-API, Pos, Scan, Frag: Var, "160-2000
10 -> 90% ACN; TIC
AcKVTHT-YLEPGPVTA-NRQLYPEWTEAQRLD-αAbuWRGK-NH2
(Compound 26)
4.714
5.668
6.053
7.129
7.747
8.143
8.604
9.806
10.055
800000
700000
600000
500000
400000
300000
200000
100000
0
2
4
6
8
10
min
4:07:38 PM SYSTEM

matogram(s)

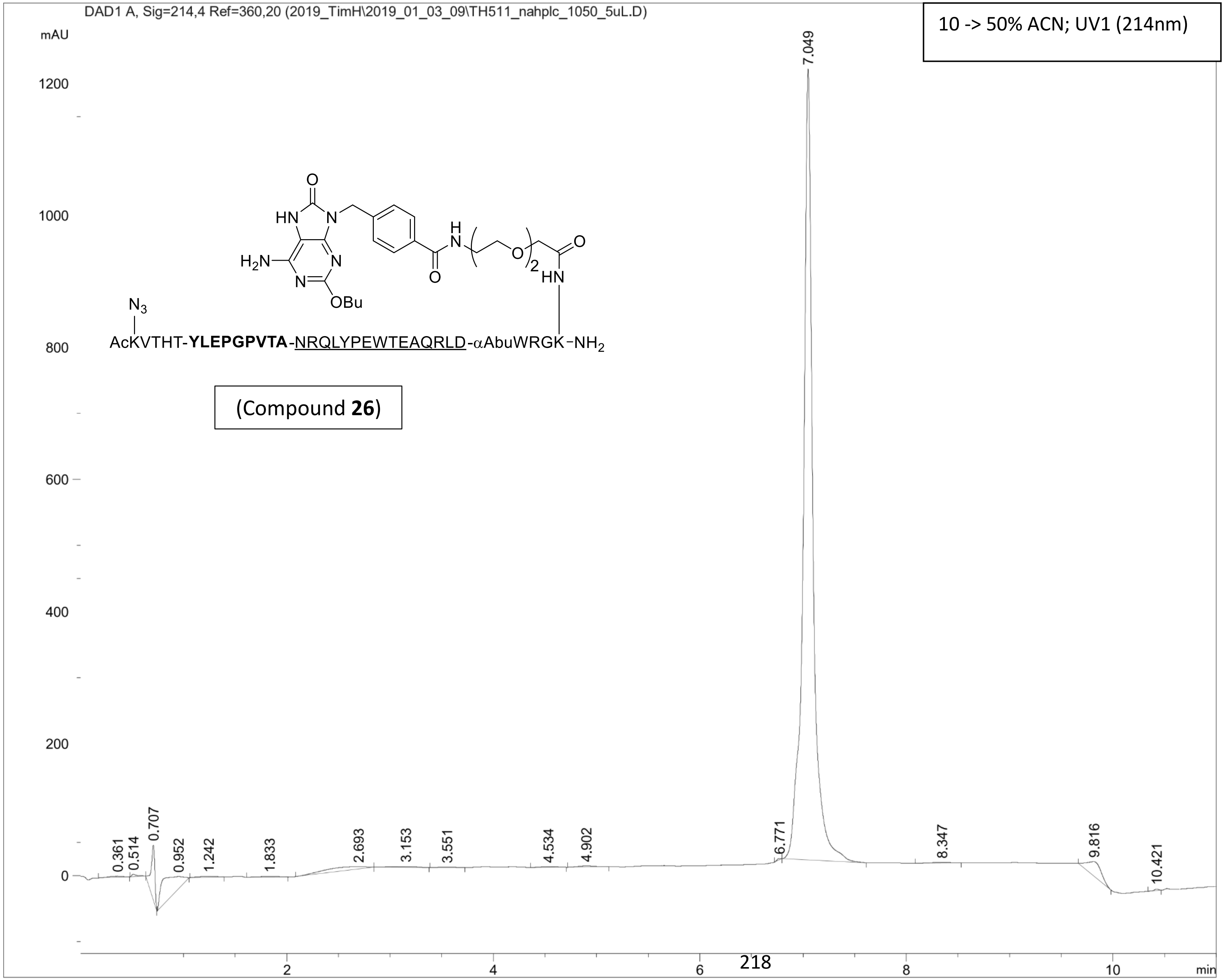

4:07:50 PM SYSTEM

matogram(s)

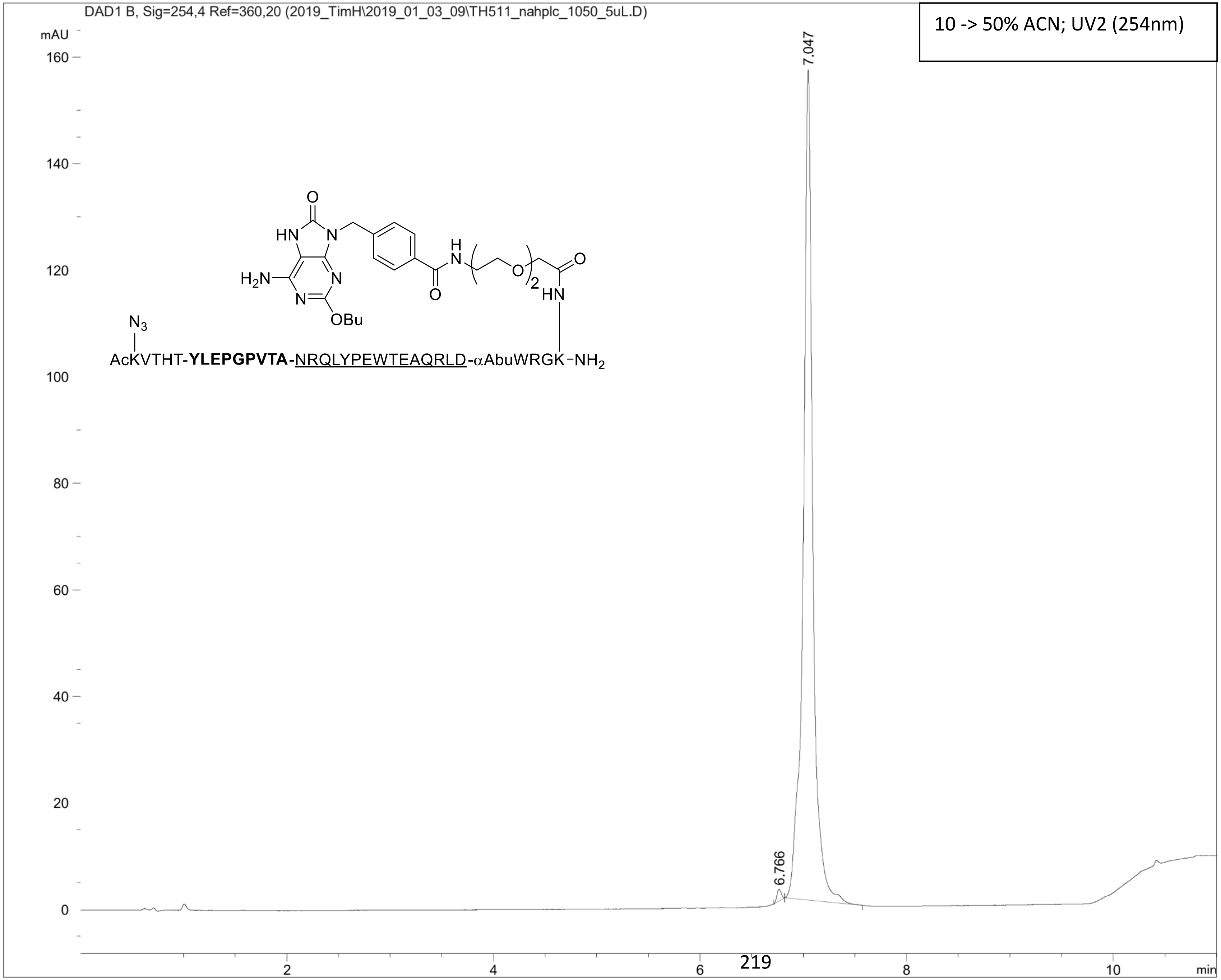

4:08:05 PM SYSTEM

matogram(s)

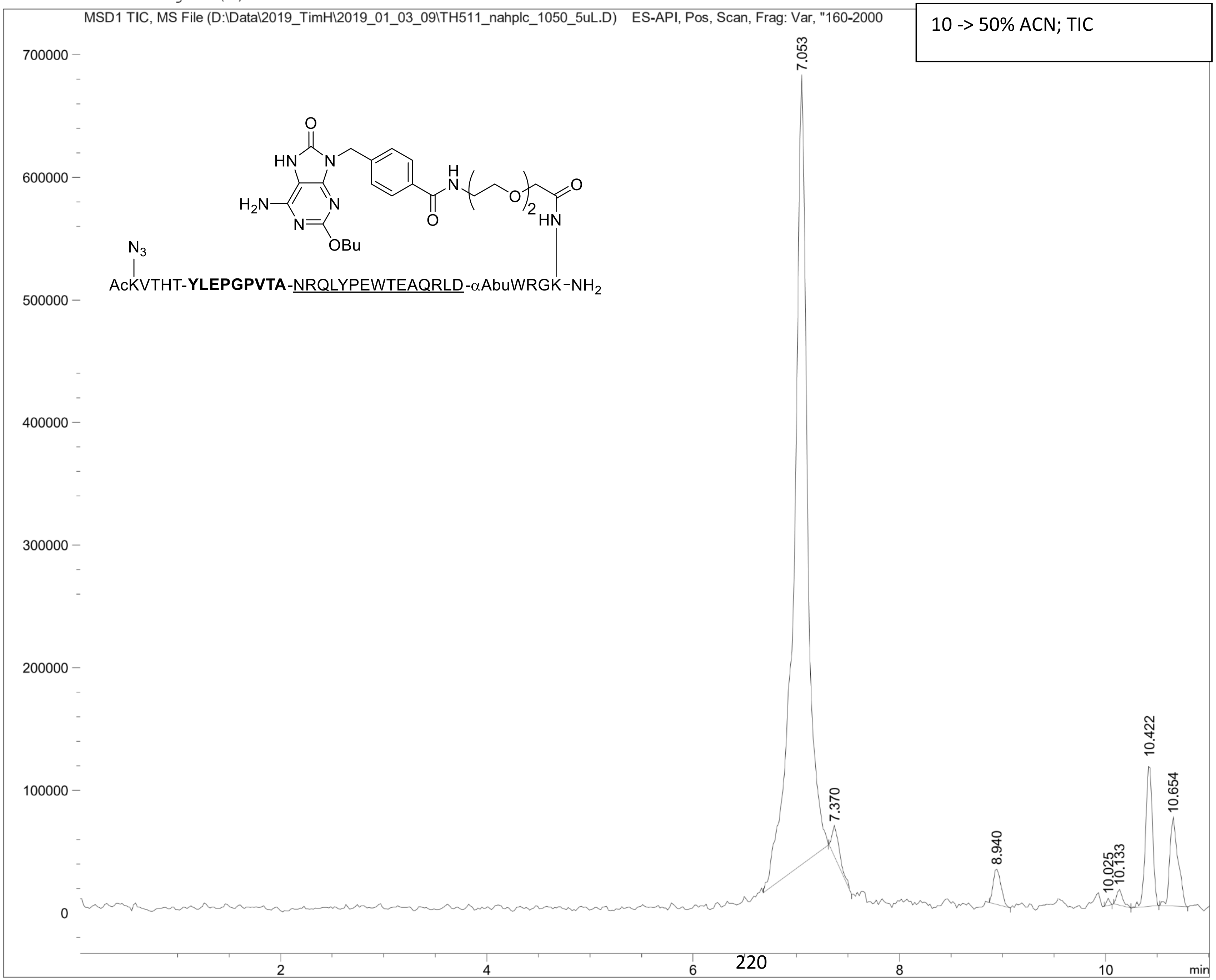

4:08:14 PM SYSTEM

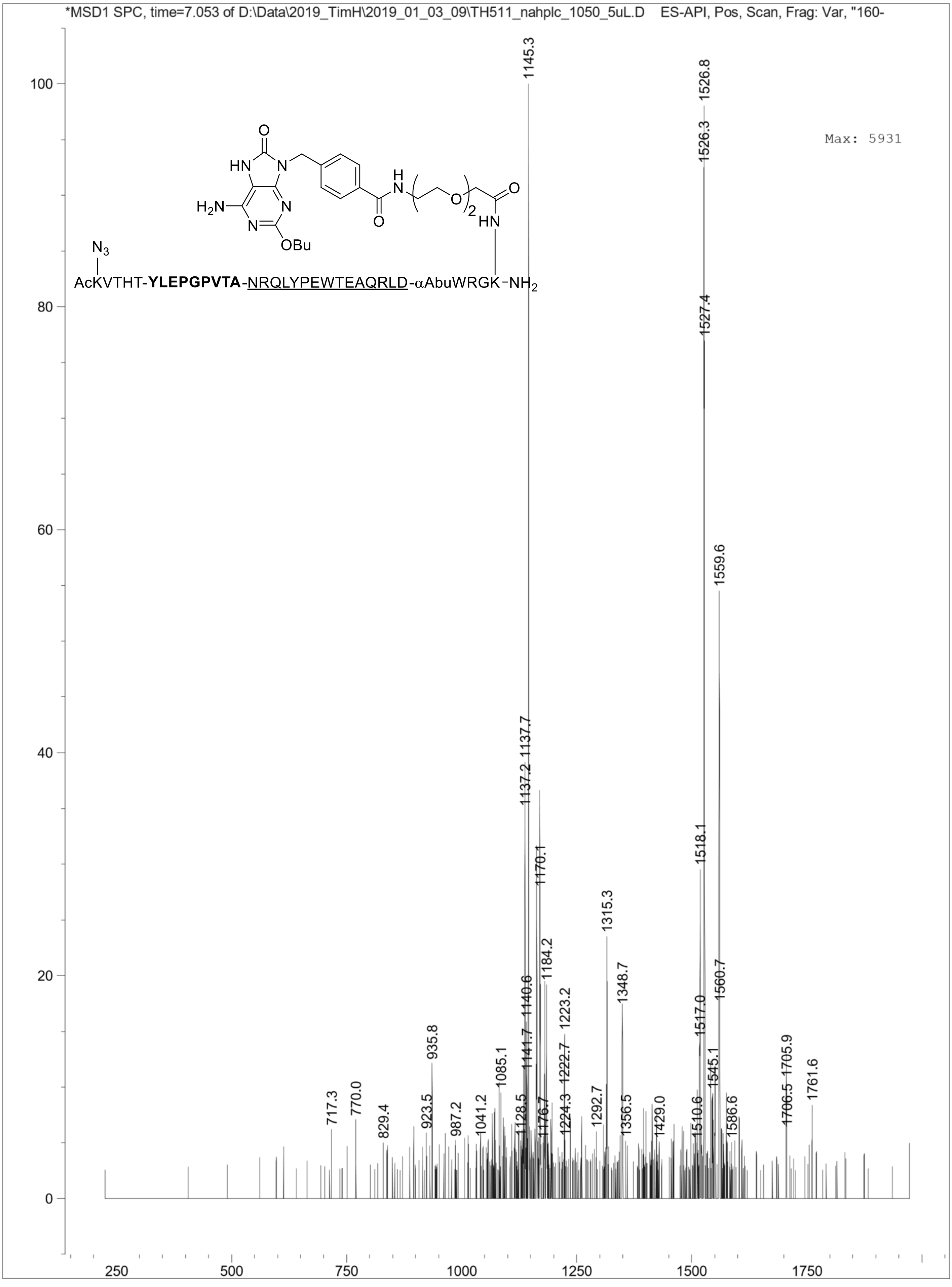
*MSD1 SPC, time=7.053 of D:\Data\2019_TimH\2019_01_03_09\TH511_nahplc_1050_5uL.D ES-API, Pos, Scan, Frag: Var, "160-
Max: 5931
AcKVTHT-YLEPGPVTA-NRQLYPEWTEAQRLD-αAbuWRGK-NH2
1145.3
1526.8
1526.3
1527.4
1559.6
1137.2 1137.7
1170.1
1518.1
1315.3
1184.2
1348.7
1560.7
1140.6
1223.2
1517.0
935.8
1141.7
1222.7
1705.9
1085.1
1545.1
1761.6
717.3
770.0
923.5
987.2
1041.2
1128.5
1176.7
1224.3
1292.7
1356.5
1429.0
1510.6
1586.6
1706.5
829.4
250
500
750
1000
1250
1500
1750
0
20
40
60
80
100

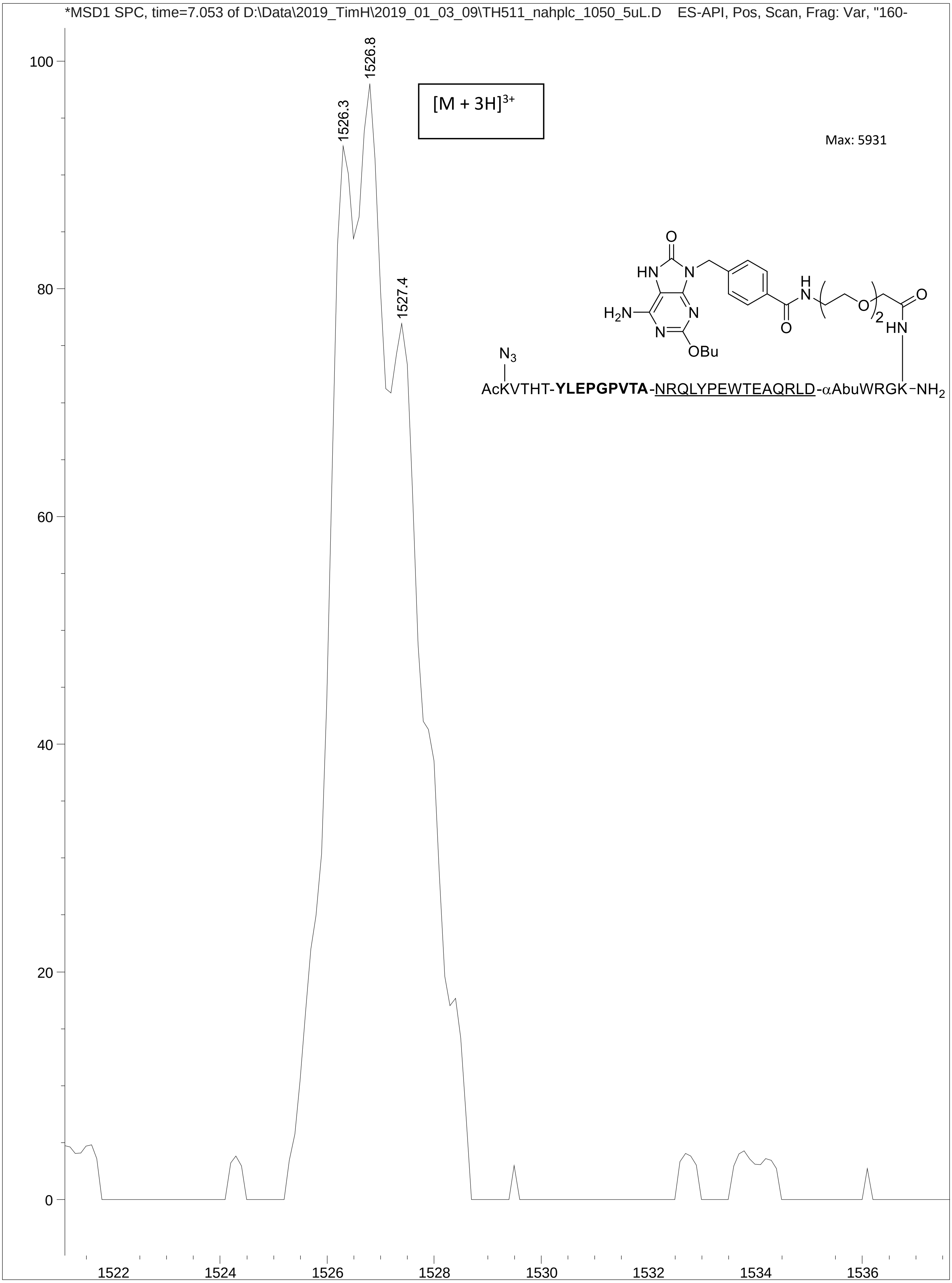

*MSD1 SPC, time=7.053 of D:\Data\2019_TimH\2019_01_03_09\TH511_nahplc_1050_5uL.D ES-API, Pos, Scan, Frag: Var, "160-
[M + 3H]3+
Max: 5931
1526.8
1526.3
1527.4
100
80
60
40
20
0
1522
1524
1526
1528
1530
1532
1534
1536
AcKVTHT-YLEPGPVTA-NRQLYPEWTEAQRLD-αAbuWRGK-NH2

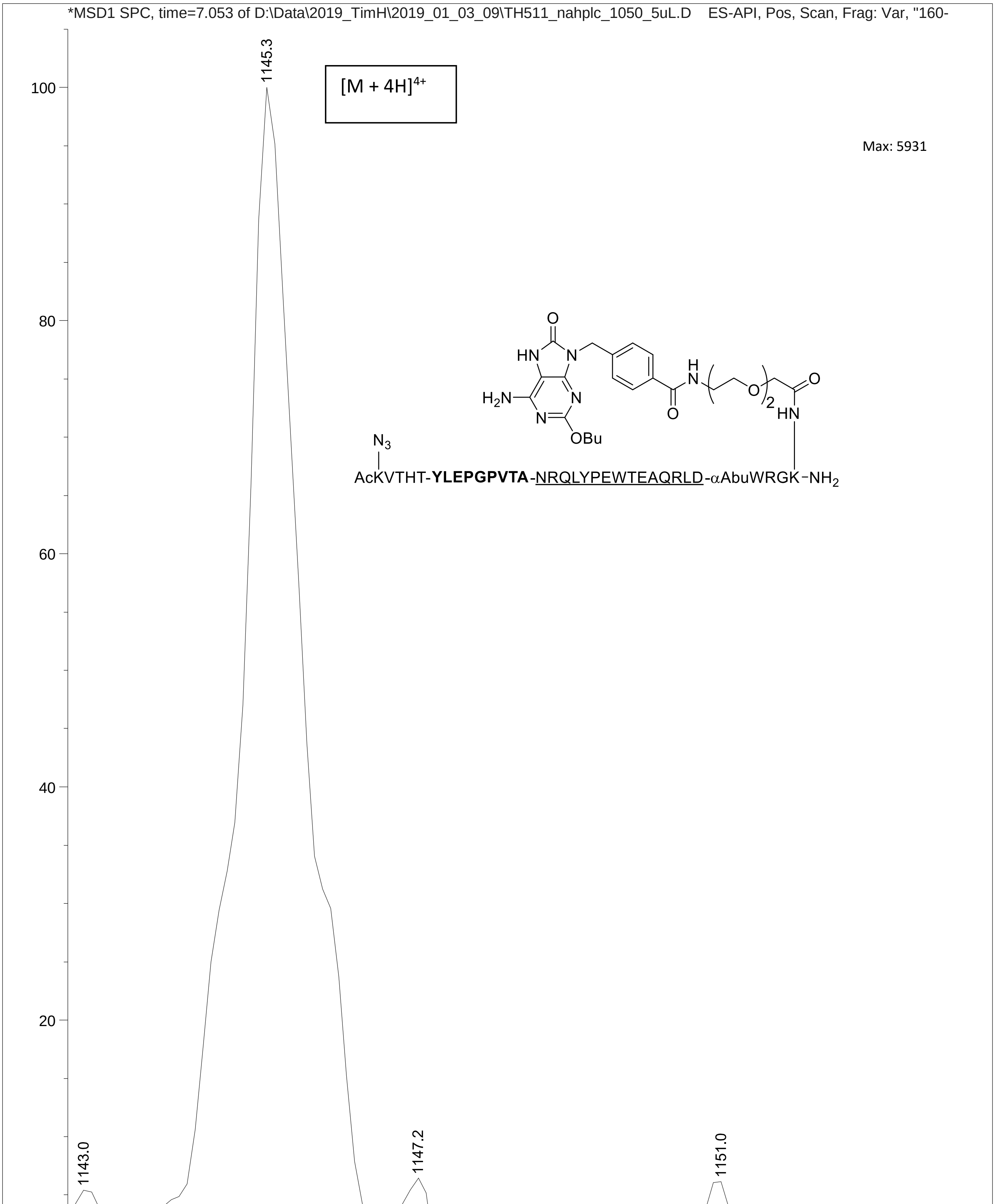
*MSD1 SPC, time=7.053 of D:\Data\2019_TimH\2019_01_03_09\TH511_nahplc_1050_5uL.D ES-API, Pos, Scan, Frag: Var, "160-
[M + 4H]4+
Max: 5931
1145.3
1143.0
1147.2
1151.0
AcKVTHT-YLEPGPVTA-NRQLYPEWTEAQRLD-αAbuWRGK-NH2
100
80
60
40
20
0
1144
1146
1148
1150
1152

gp100

LC-MS Spectra; *(10 –> 90 % ACN, 13 min)*;(Compound **gp100**)

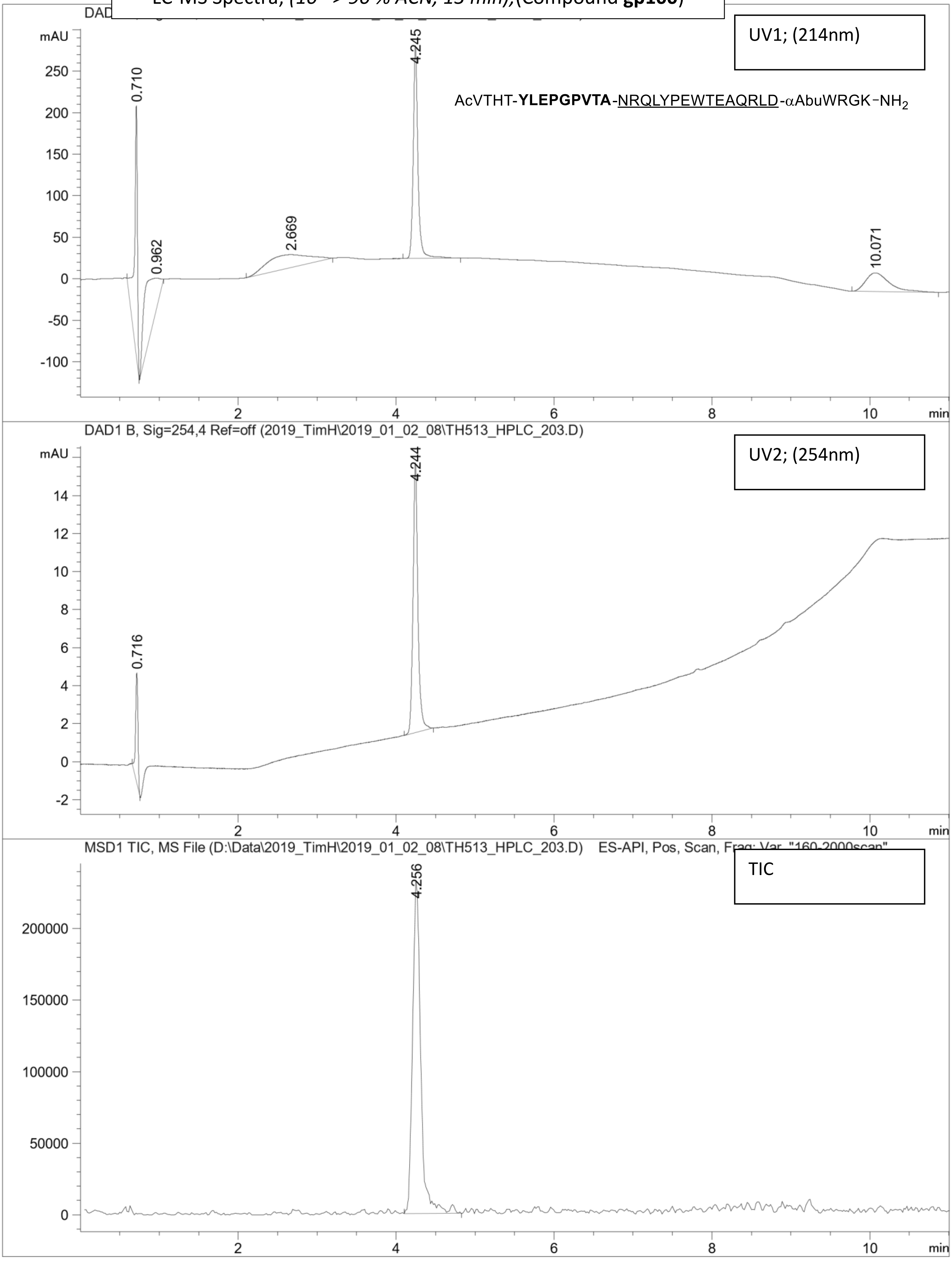

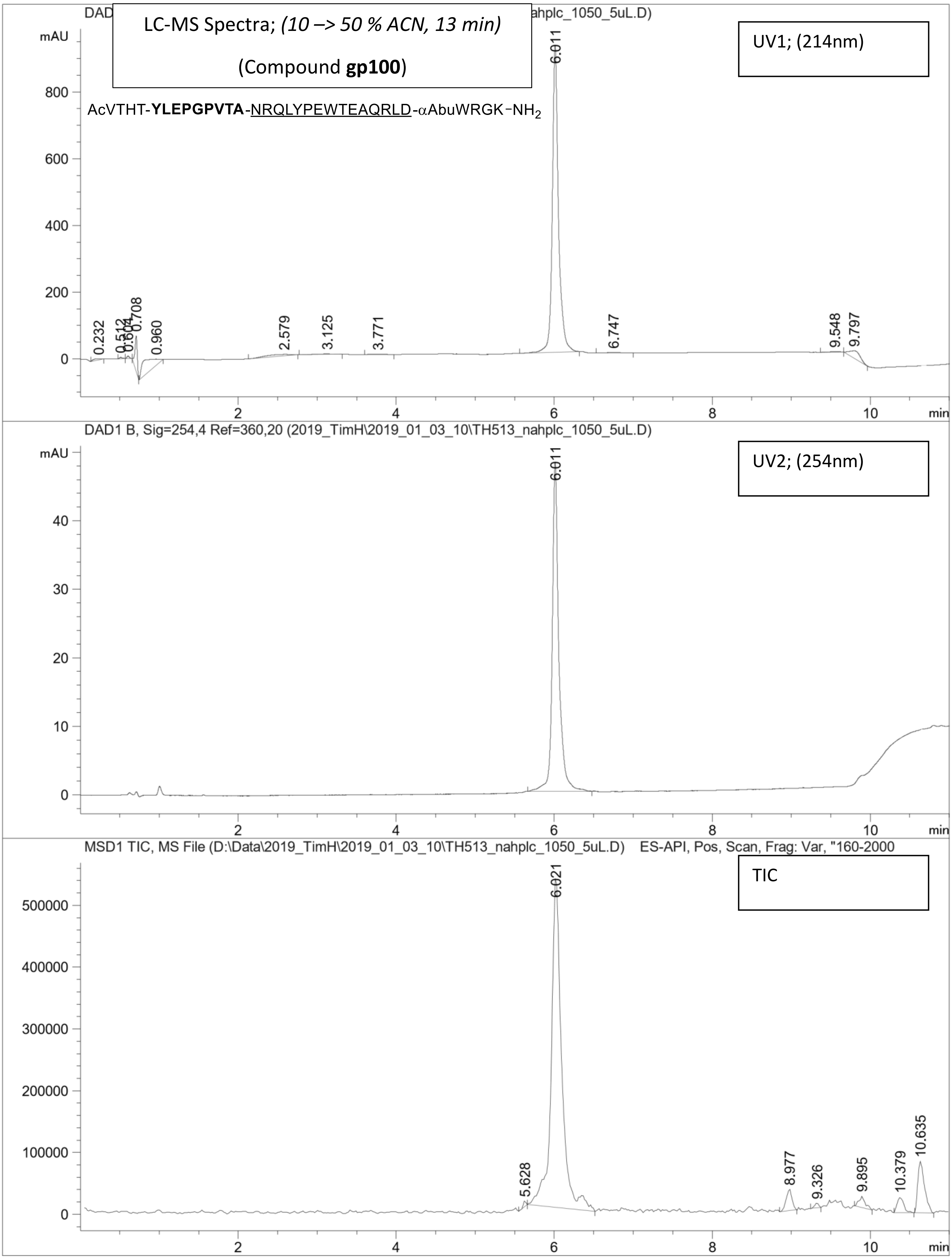
LC-MS Spectra; (10 –> 50 % ACN, 13 min)
(Compound gp100)
AcVTHT-YLEPGPVTA-NRQLYPEWTEAQRLD-αAbuWRGK-NH2
UV1; (214nm)
mAU
6.011
0.232
0.512
0.604
0.708
0.960
2.579
3.125
3.771
6.747
9.548
9.797
DAD1 B, Sig=254,4 Ref=360,20 (2019_TimH\2019_01_03_10\TH513_nahplc_1050_5uL.D)
UV2; (254nm)
MSD1 TIC, MS File (D:\Data\2019_TimH\2019_01_03_10\TH513_nahplc_1050_5uL.D) ES-API, Pos, Scan, Frag: Var, "160-2000
TIC
6.021
5.628
8.977
9.326
9.895
10.379
10.635
min

matogram(s)

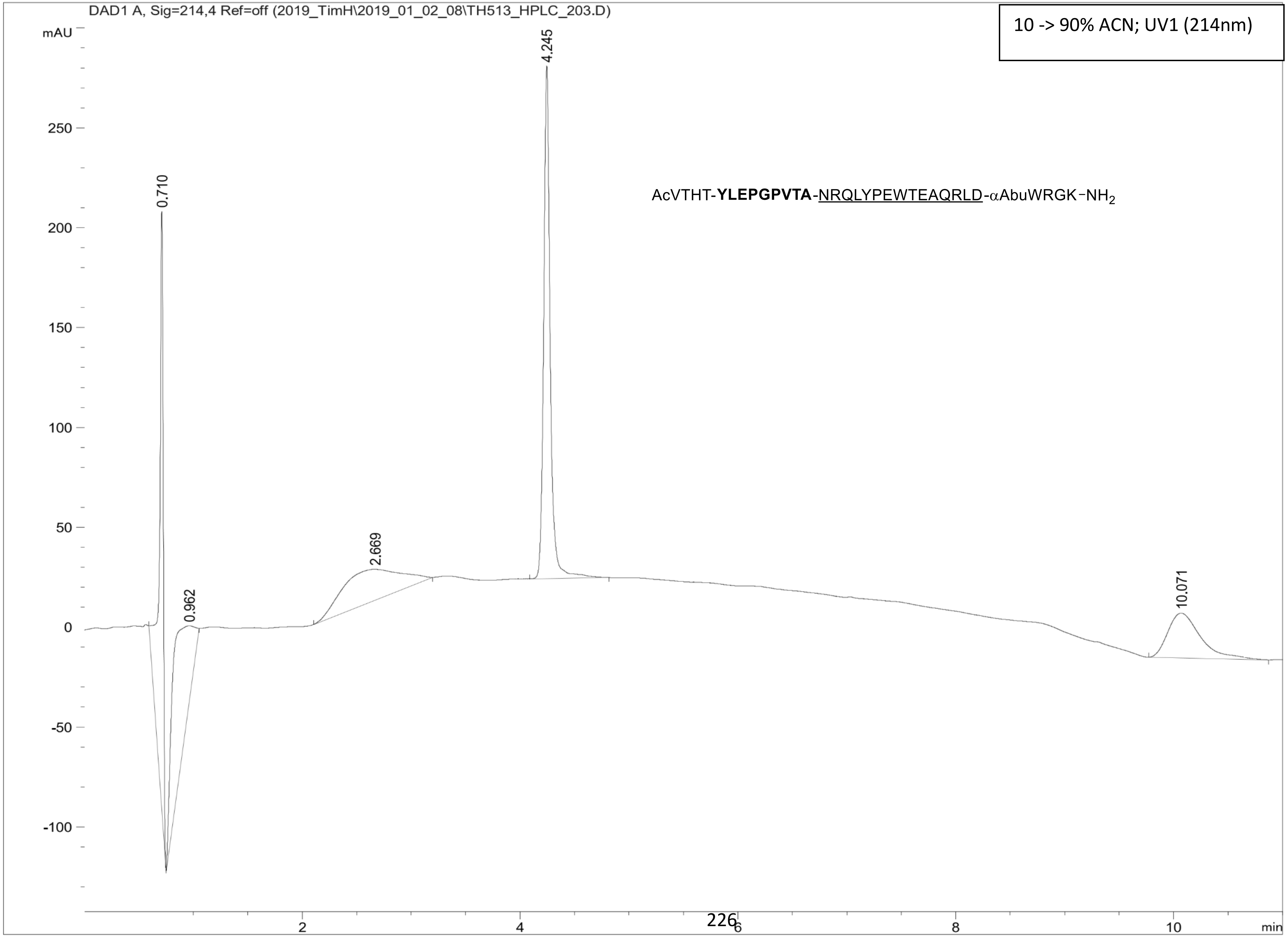

4:00:41 PM SYSTEM

Current Chromatogram(s)

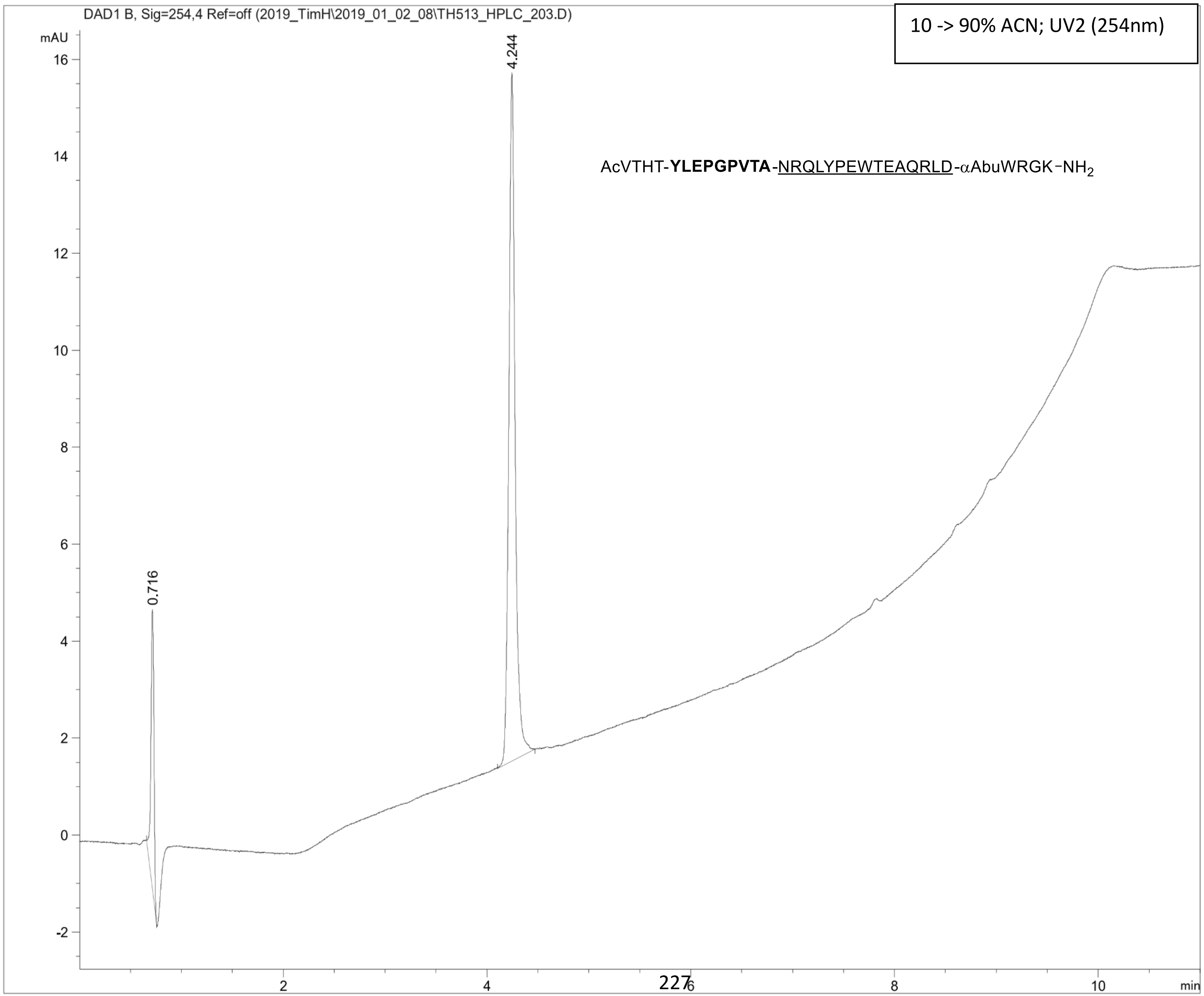

LC-MS_6120B 1/10/2019 4:01:13 PM SYSTEM Page 1 of 1

matogram(s)

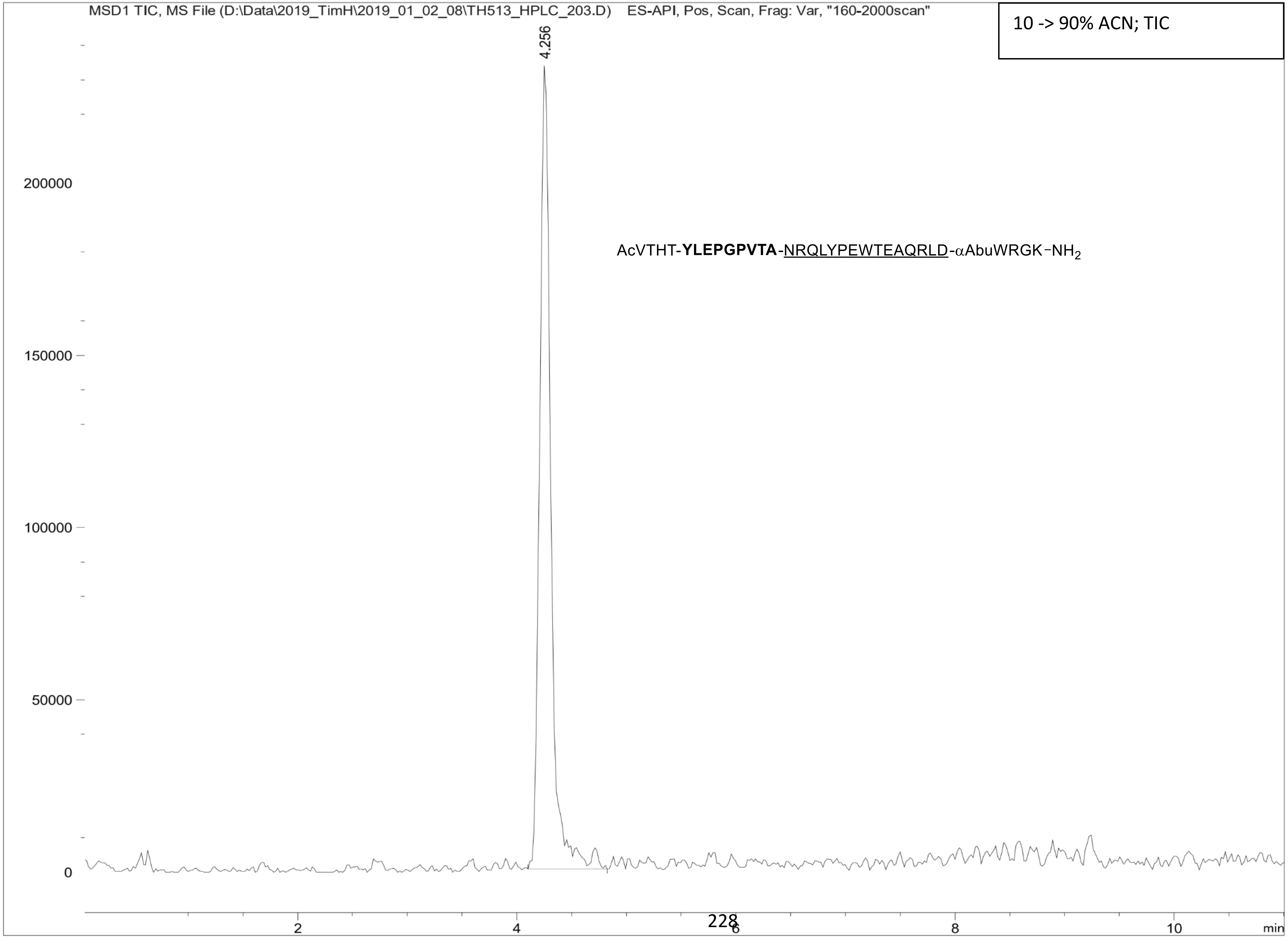

4:01:19 PM SYSTEM

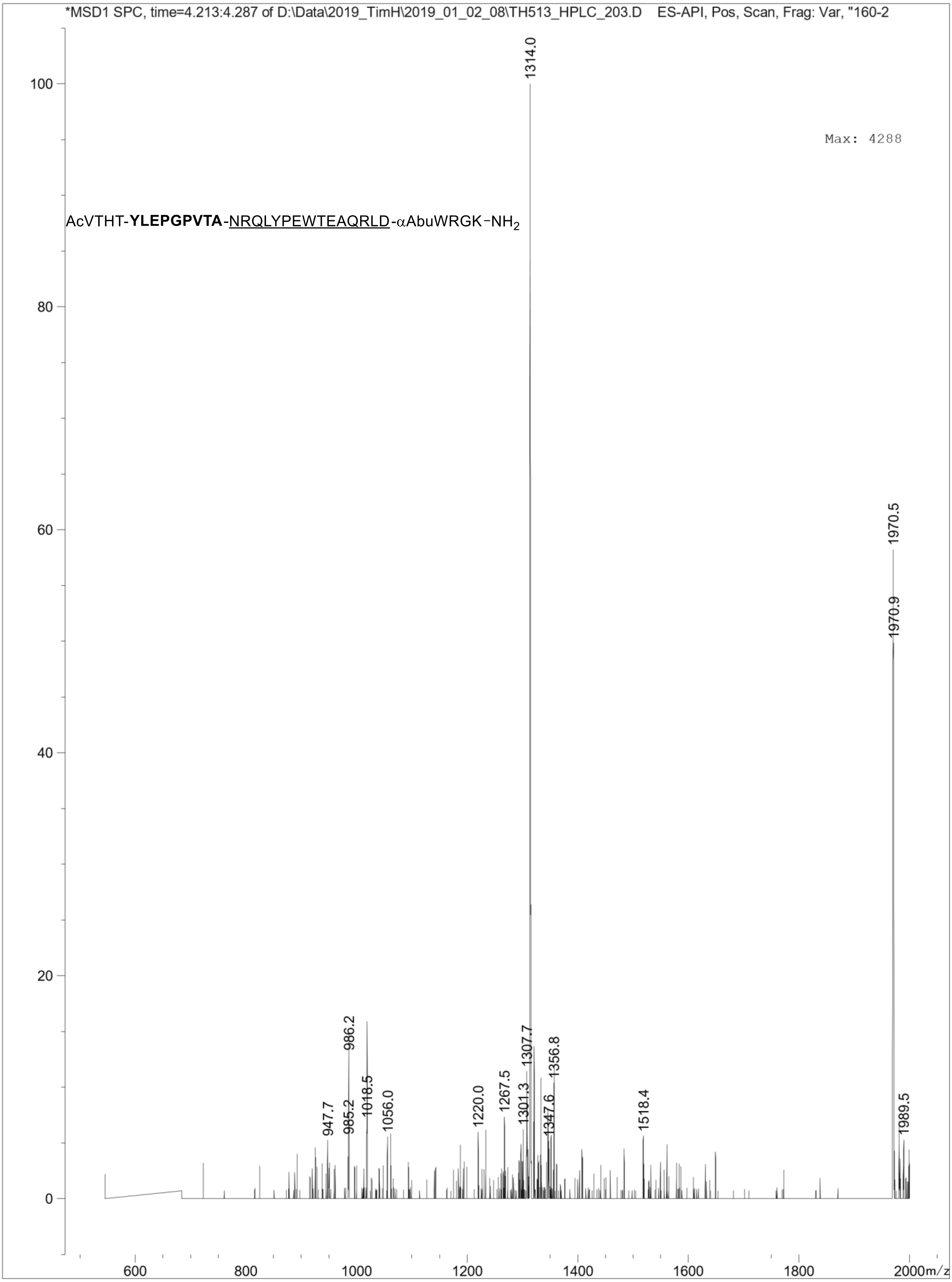
*MSD1 SPC, time=4.213:4.287 of D:\Data\2019_TimH\2019_01_02_08\TH513_HPLC_203.D ES-API, Pos, Scan, Frag: Var, "160-2
Max: 4288
AcVTHT-YLEPGPVTA-NRQLYPEWTEAQRLD-αAbuWRGK-NH2
1314.0
1970.5
1970.9
986.2
947.7
985.2
1018.5
1056.0
1220.0
1267.5
1301.3
1307.7
1347.6
1356.8
1518.4
1989.5
100
80
60
40
20
0
600
800
1000
1200
1400
1600
1800
2000
m/z

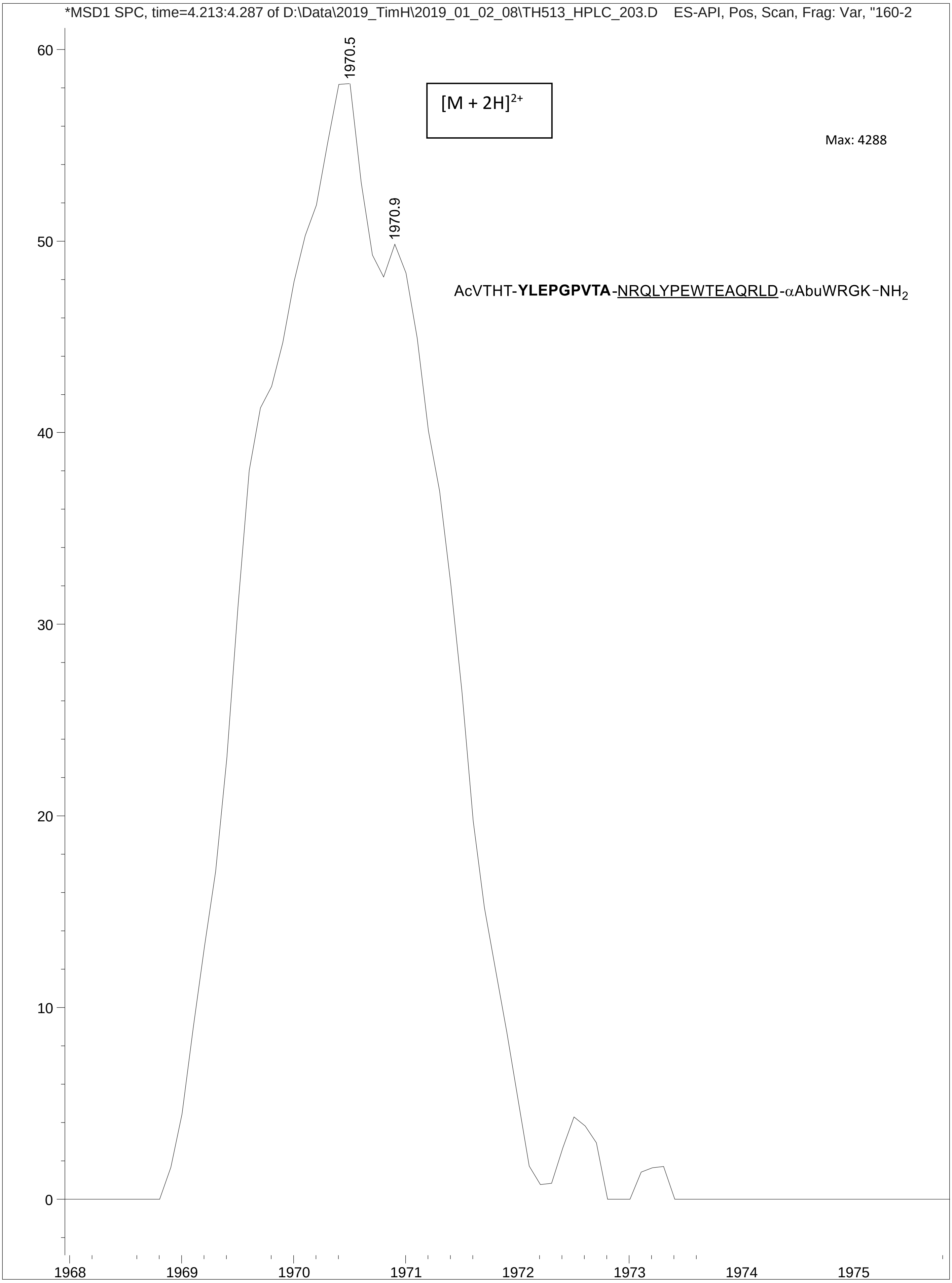

*MSD1 SPC, time=4.213:4.287 of D:\Data\2019_TimH\2019_01_02_08\TH513_HPLC_203.D ES-API, Pos, Scan, Frag: Var, "160-2
1970.5
1970.9
[M + 2H]2+
Max: 4288
AcVTHT-YLEPGPVTA-NRQLYPEWTEAQRLD-αAbuWRGK-NH2
60
50
40
30
20
10
0
1968
1969
1970
1971
1972
1973
1974
1975

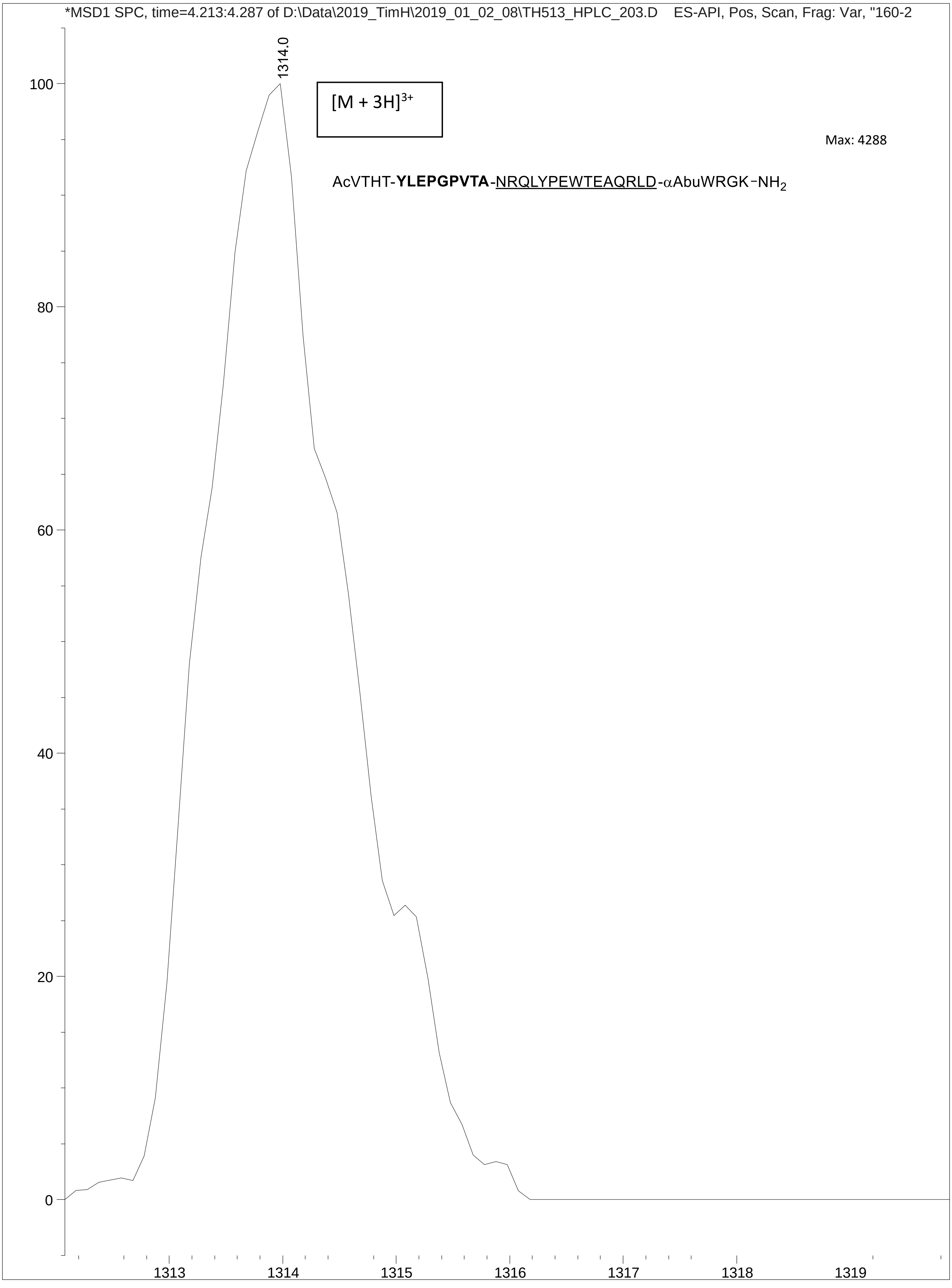
*MSD1 SPC, time=4.213:4.287 of D:\Data\2019_TimH\2019_01_02_08\TH513_HPLC_203.D ES-API, Pos, Scan, Frag: Var, "160-2
1314.0
[M + 3H]3+
Max: 4288
AcVTHT-YLEPGPVTA-NRQLYPEWTEAQRLD-αAbuWRGK-NH2
100
80
60
40
20
0
1313
1314
1315
1316
1317
1318
1319

gp100-TLR7L

LC-MS Spectra; *(10 –> 90 % ACN, 13 min);* (Compound **gp100-TLR7L**)

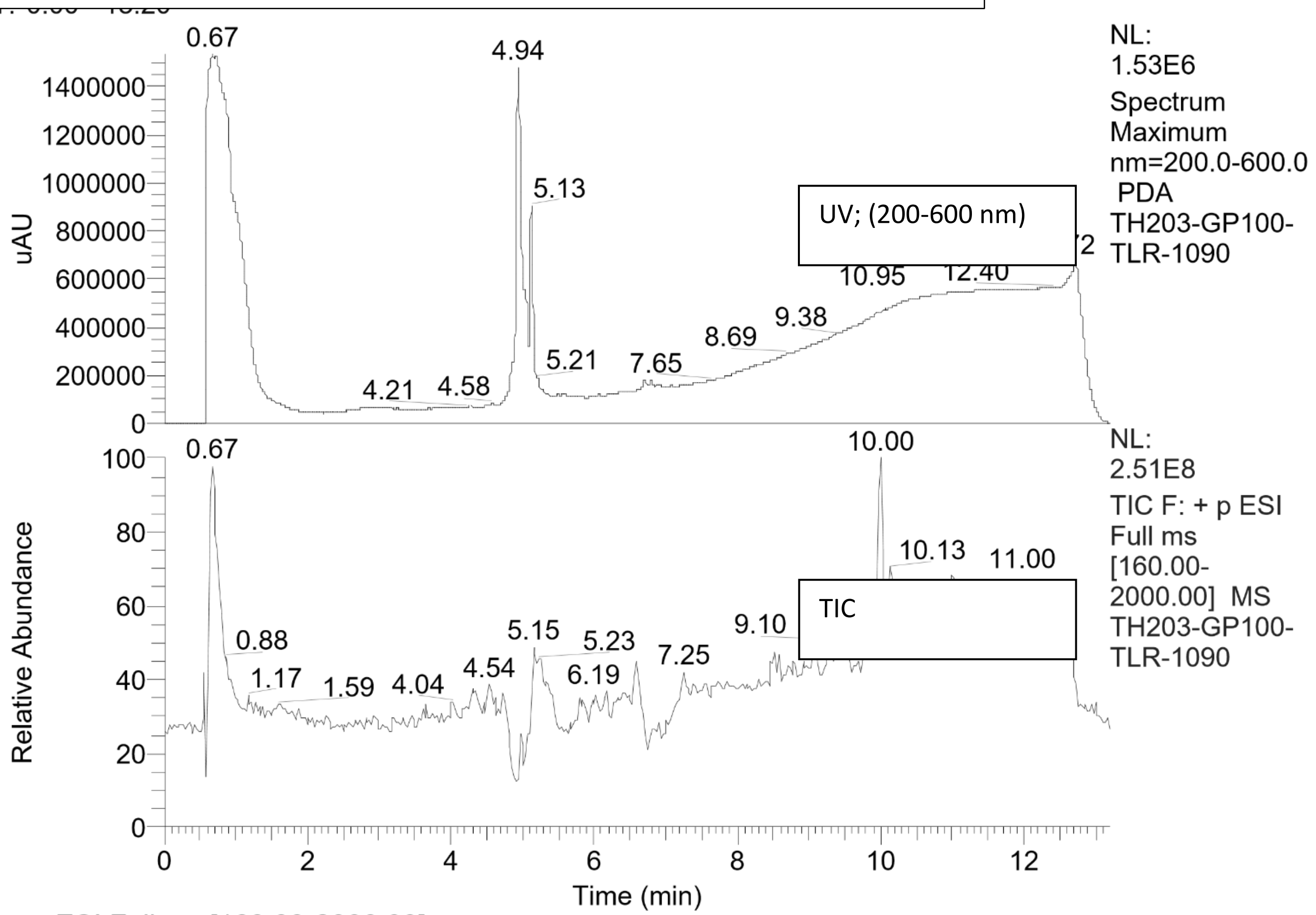

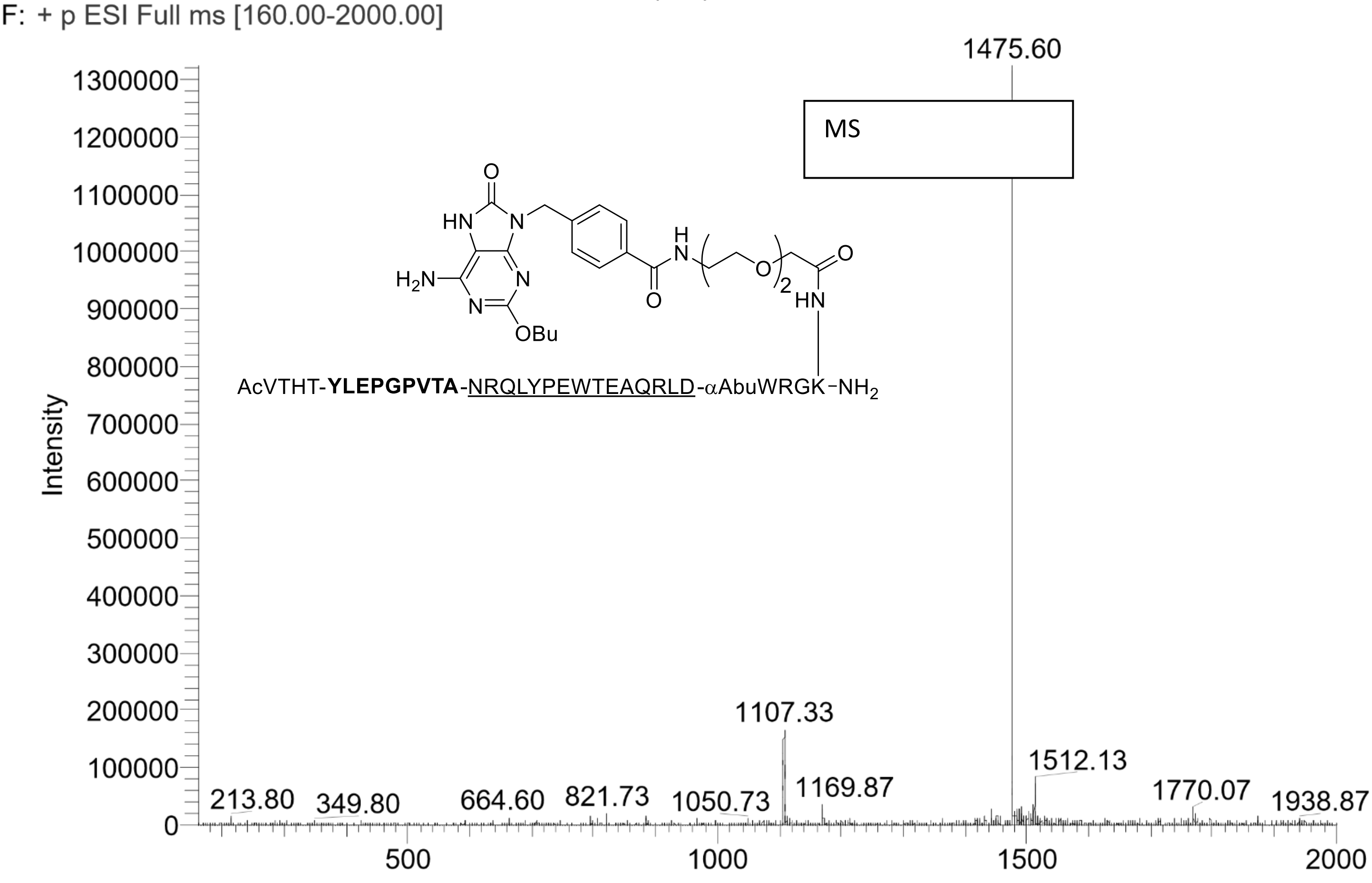

gp100-(cys)-TLR7L

LC-MS Spectra; *(10 –> 90 % ACN, 13 min);* (Compound **gp100-(Cys)-TLR7L**)

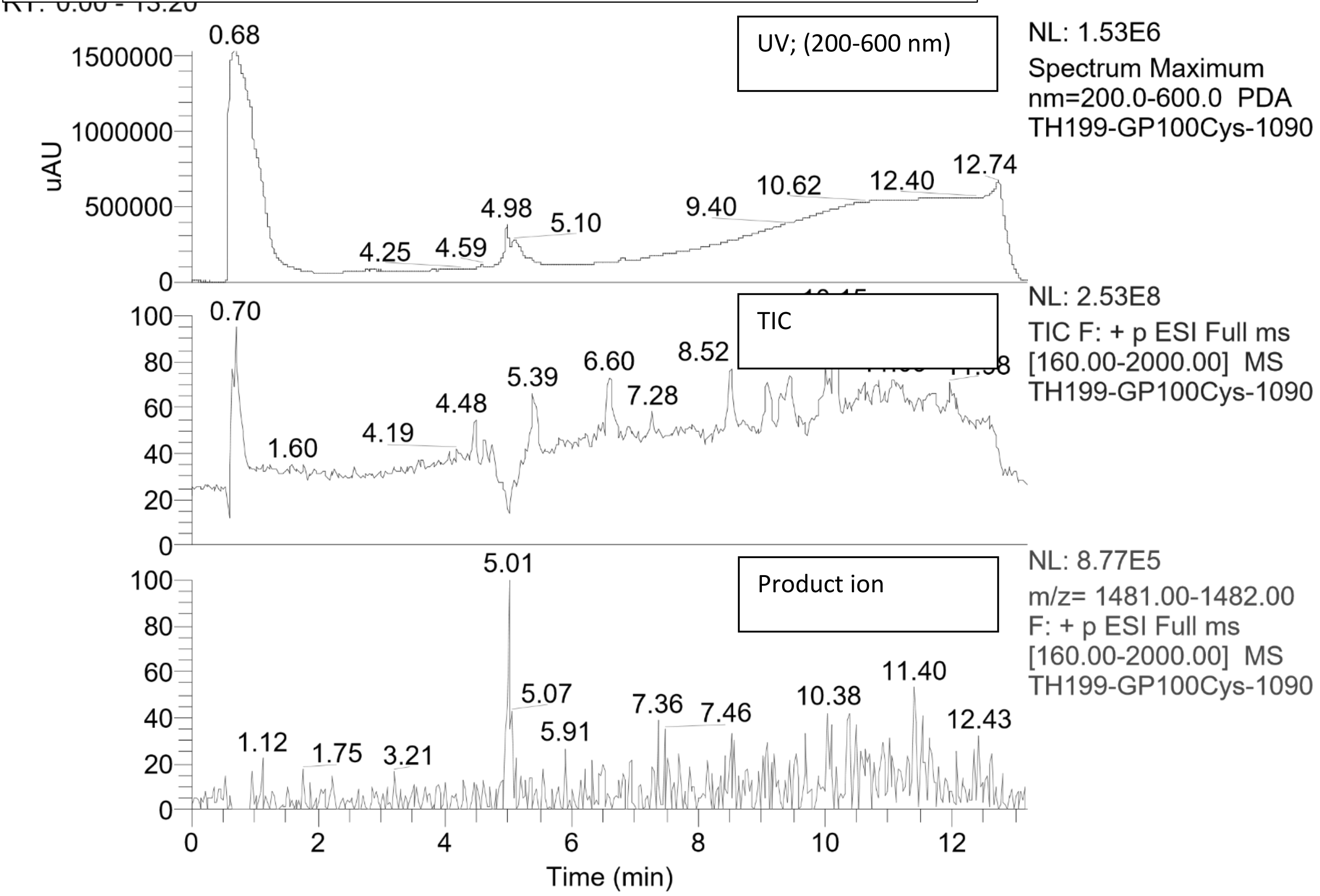

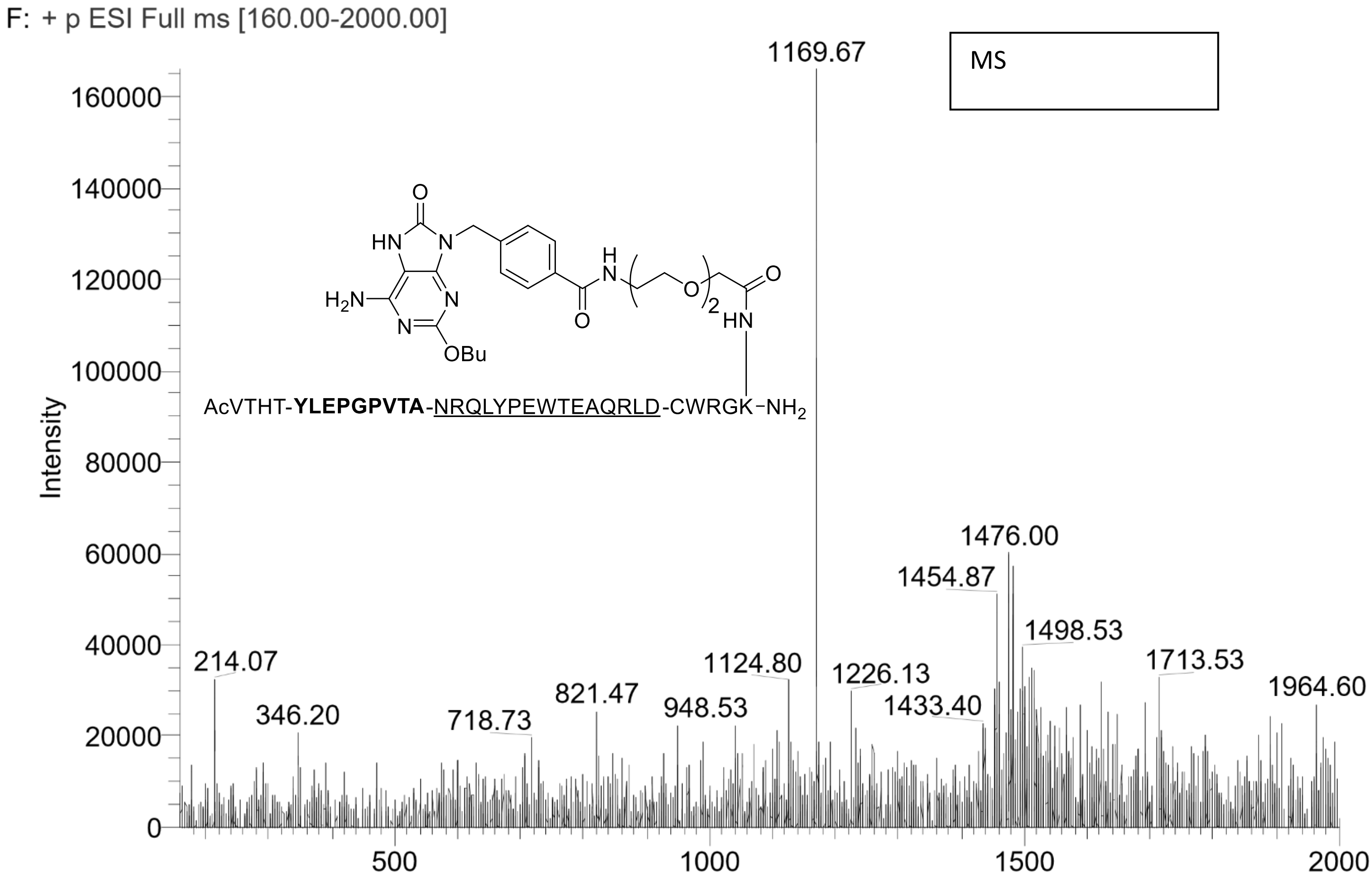
F: + p ESI Full ms [160.00-2000.00]
MS
1169.67
1476.00
1454.87
1498.53
1713.53
1964.60
1433.40
1226.13
1124.80
948.53
821.47
718.73
346.20
214.07
Intensity
AcVTHT-YLEPGPVTA-NRQLYPEWTEAQRLD-CWRGK-NH2
OBu

Spectra of glycopeptides

B6-gp100-TLR7L

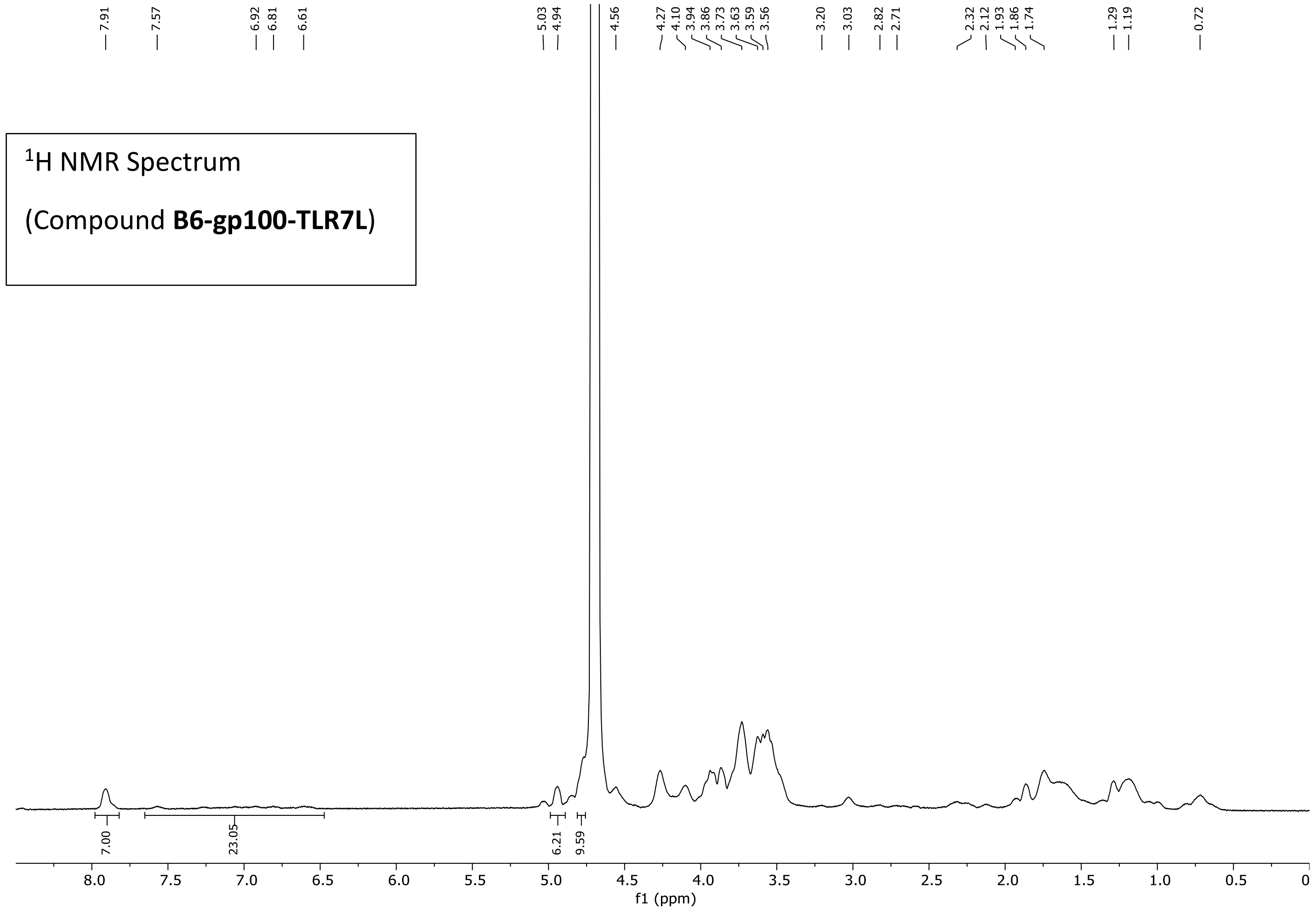

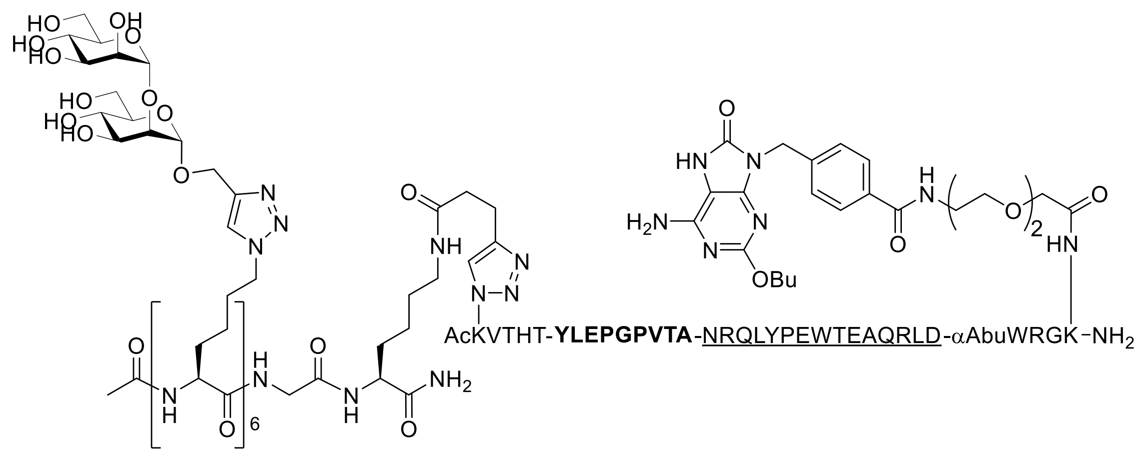

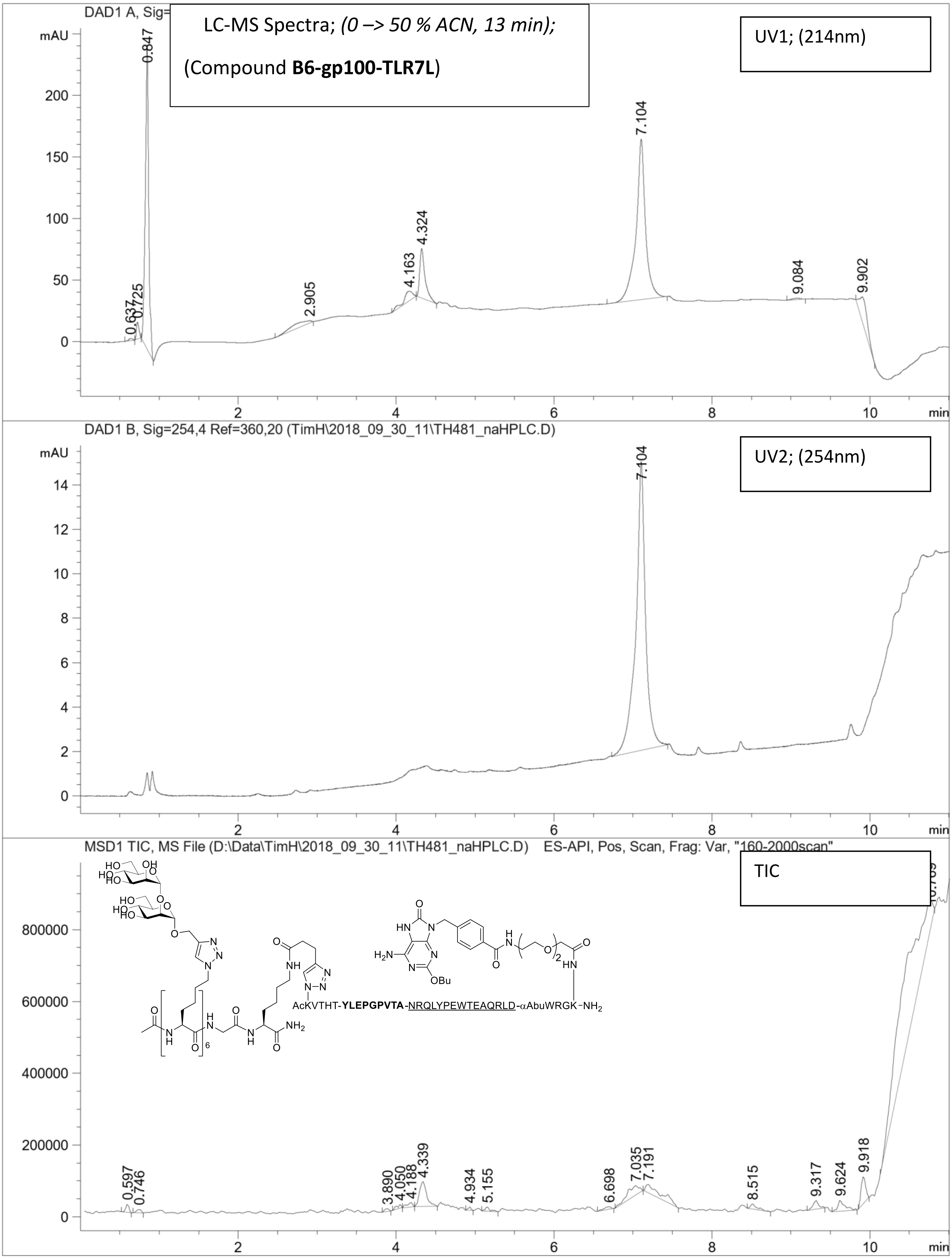
LC-MS Spectra; (0 –> 50 % ACN, 13 min);
(Compound B6-gp100-TLR7L)
DAD1 A, Sig=
UV1; (214nm)
mAU
0.637
0.725
0.847
2.905
4.163
4.324
7.104
9.084
9.902
min
DAD1 B, Sig=254,4 Ref=360,20 (TimH\2018_09_30_11\TH481_naHPLC.D)
UV2; (254nm)
mAU
7.104
min
MSD1 TIC, MS File (D:\Data\TimH\2018_09_30_11\TH481_naHPLC.D) ES-API, Pos, Scan, Frag: Var, "160-2000scan"
TIC
0.597
0.746
3.890
4.050
4.188
4.339
4.934
5.155
6.698
7.035
7.191
8.515
9.317
9.624
9.918
min
AcKVTHT-YLEPGPVTA-NRQLYPEWTEAQRLD-αAbuWRGK-NH2
OBu

C6-gp100-TLR7L

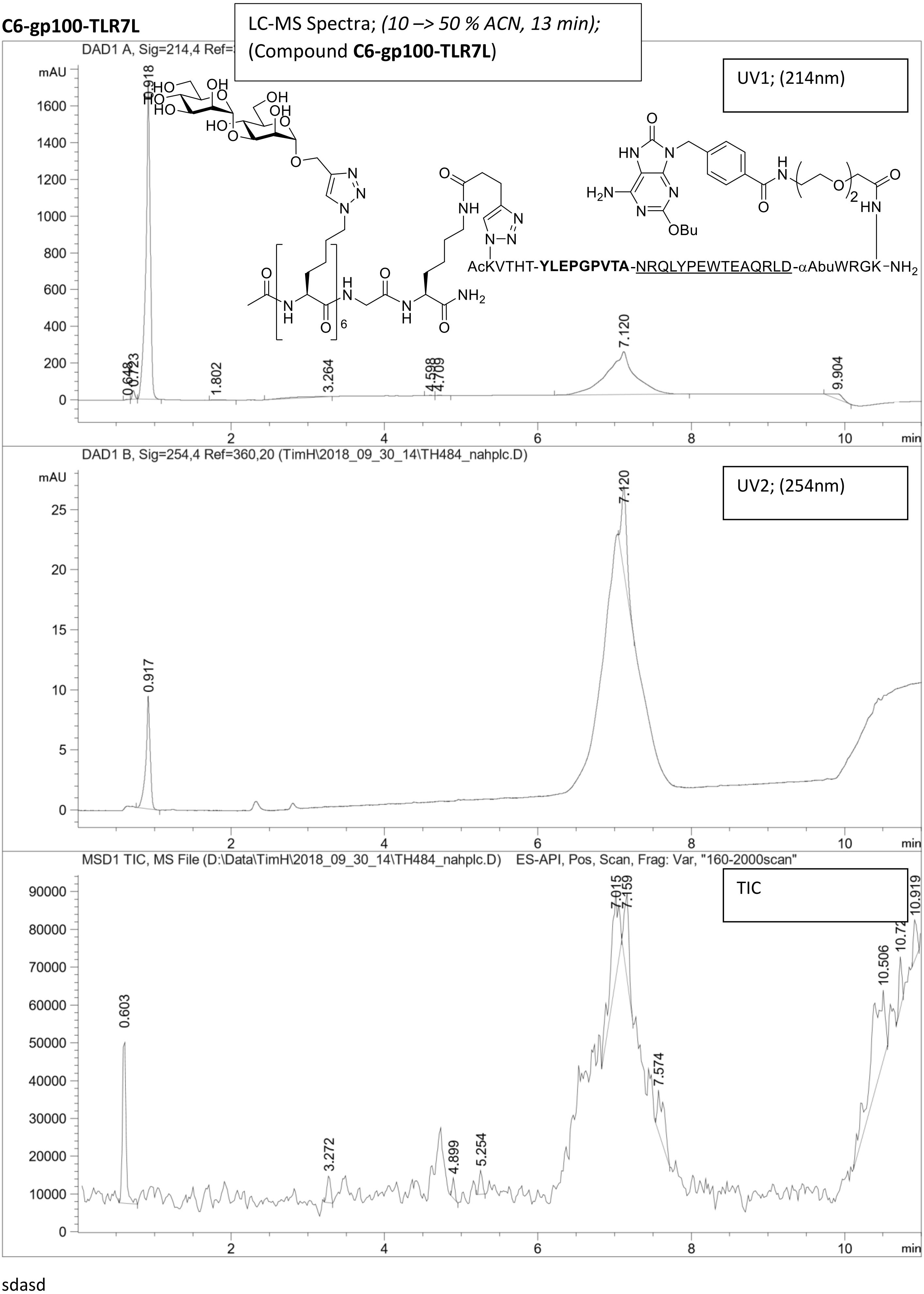

sdasd

Current Chromatogram(s)

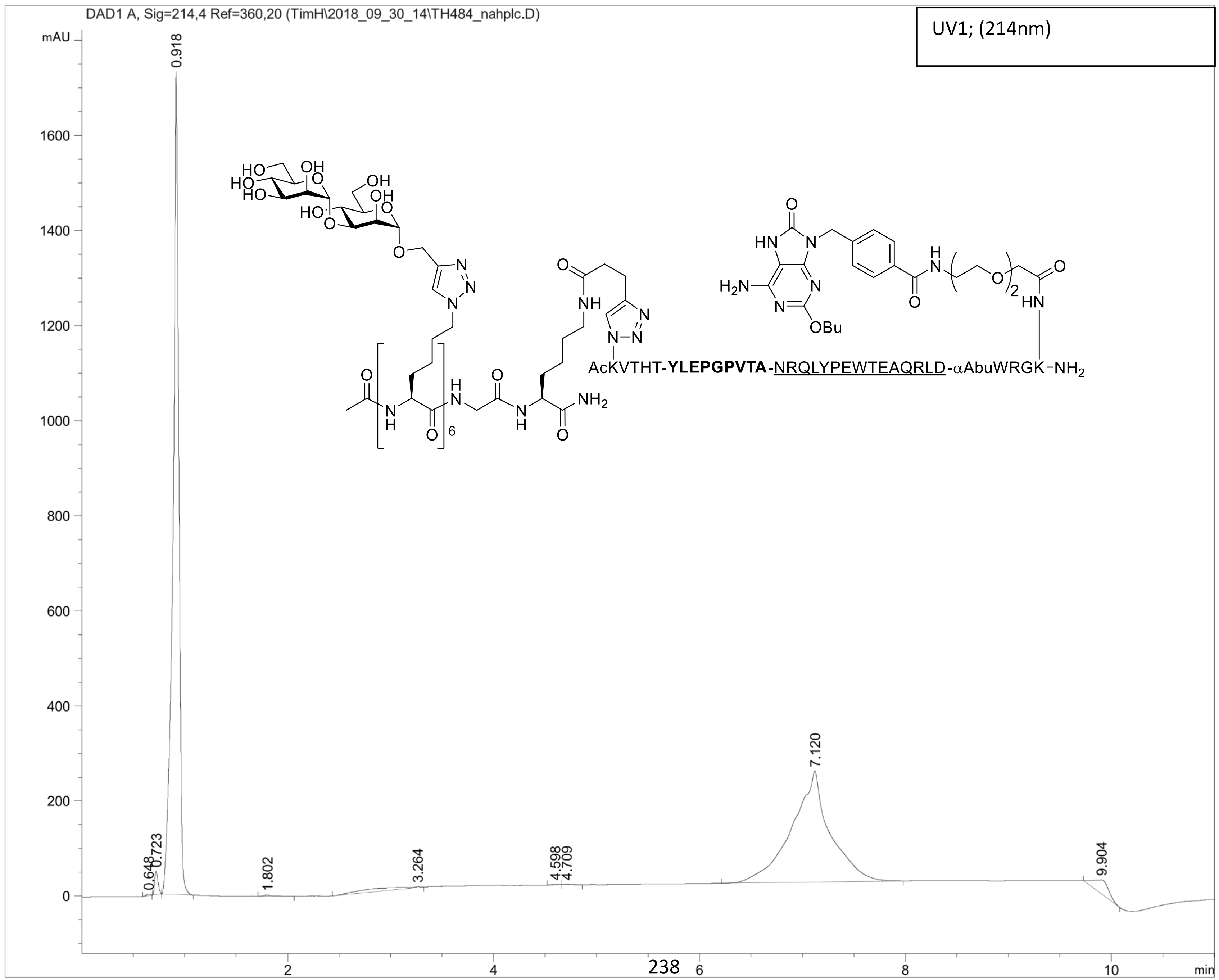

Current Chromatogram(s)

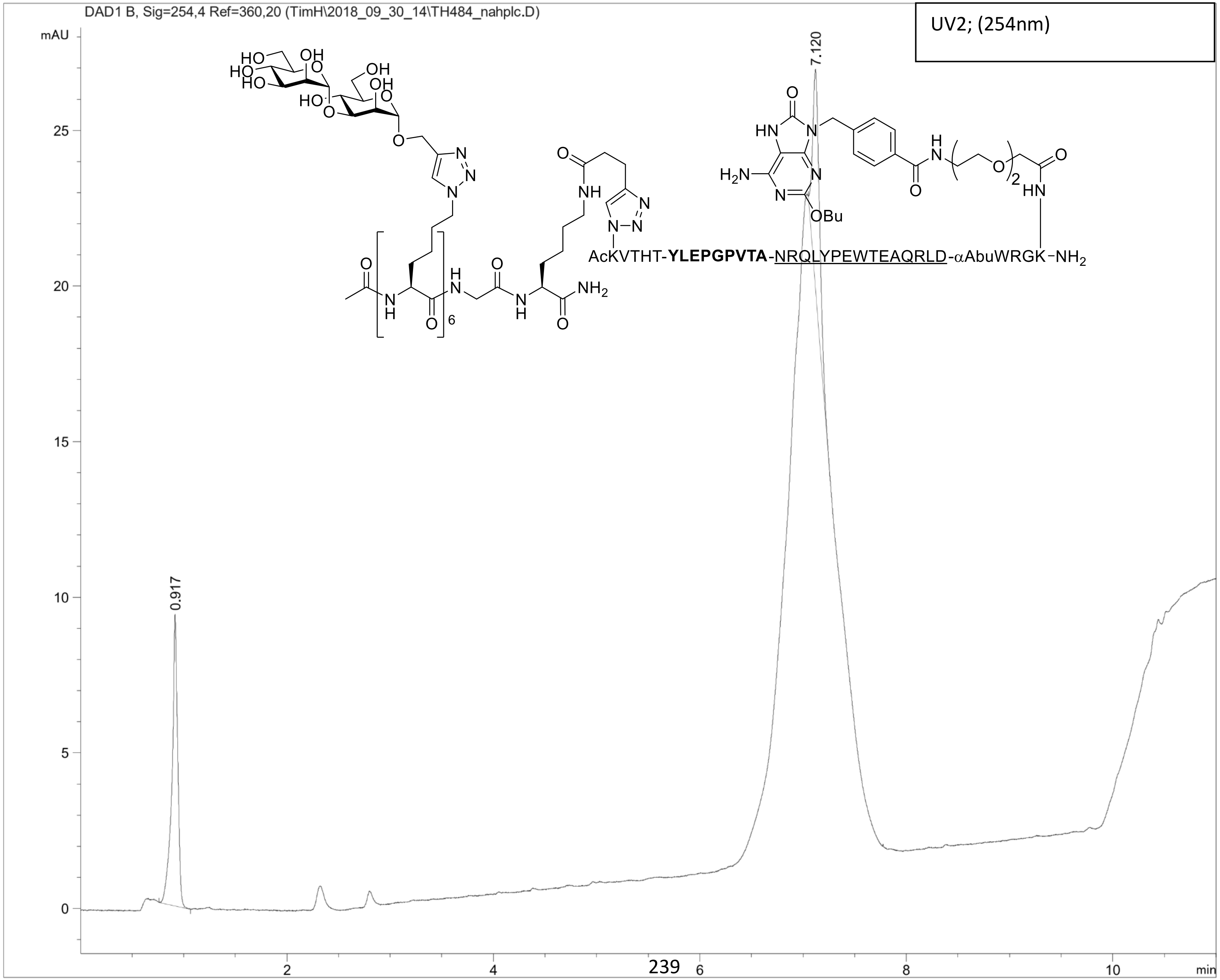

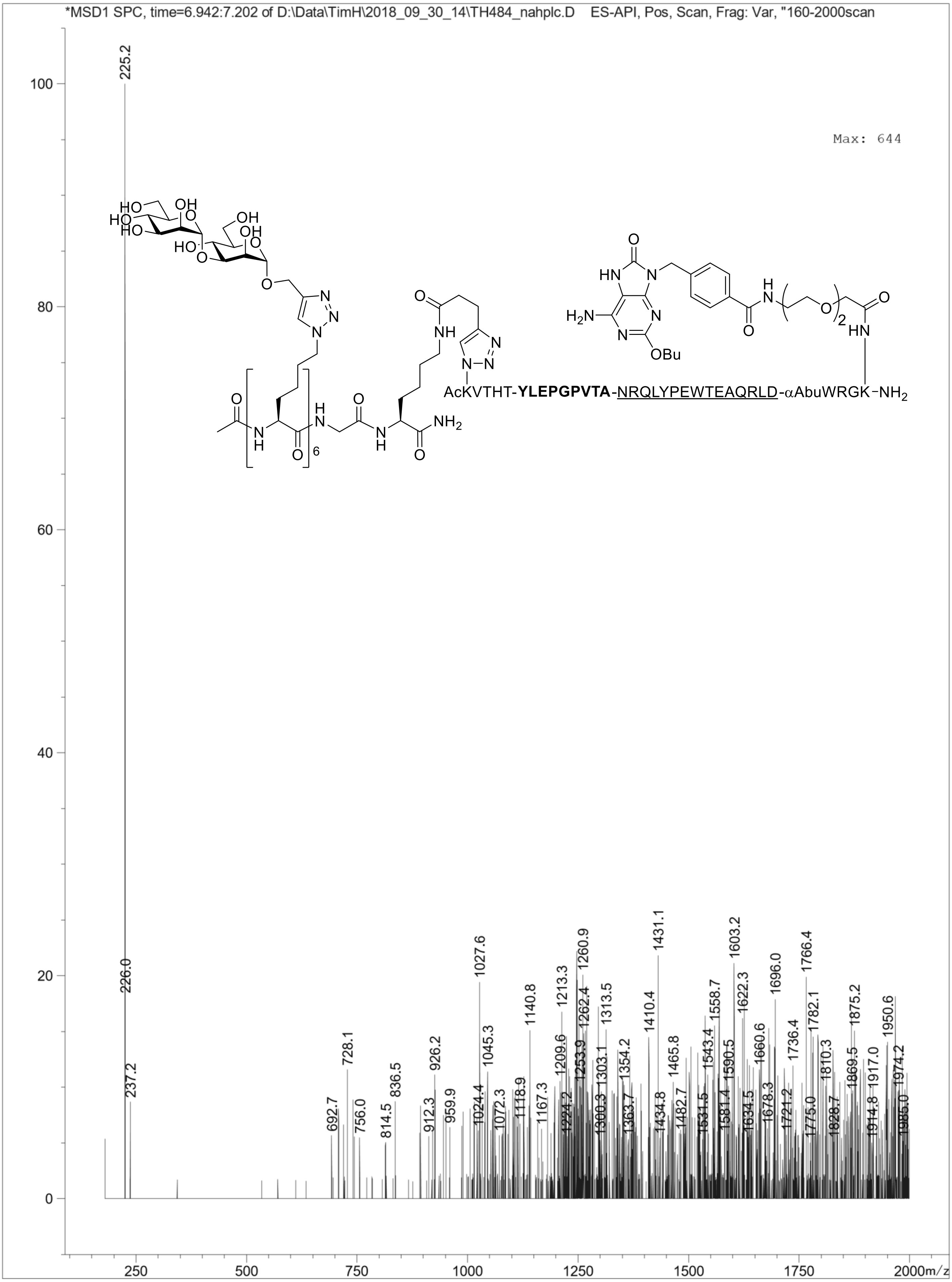
*MSD1 SPC, time=6.942:7.202 of D:\Data\TimH\2018_09_30_14\TH484_nahplc.D ES-API, Pos, Scan, Frag: Var, "160-2000scan
Max: 644
AcKVTHT-YLEPGPVTA-NRQLYPEWTEAQRLD-αAbuWRGK-NH2
225.2
226.0
237.2
1027.6
1260.9
1431.1
1603.2
1766.4
m/z

D6-gp100-TLR7L

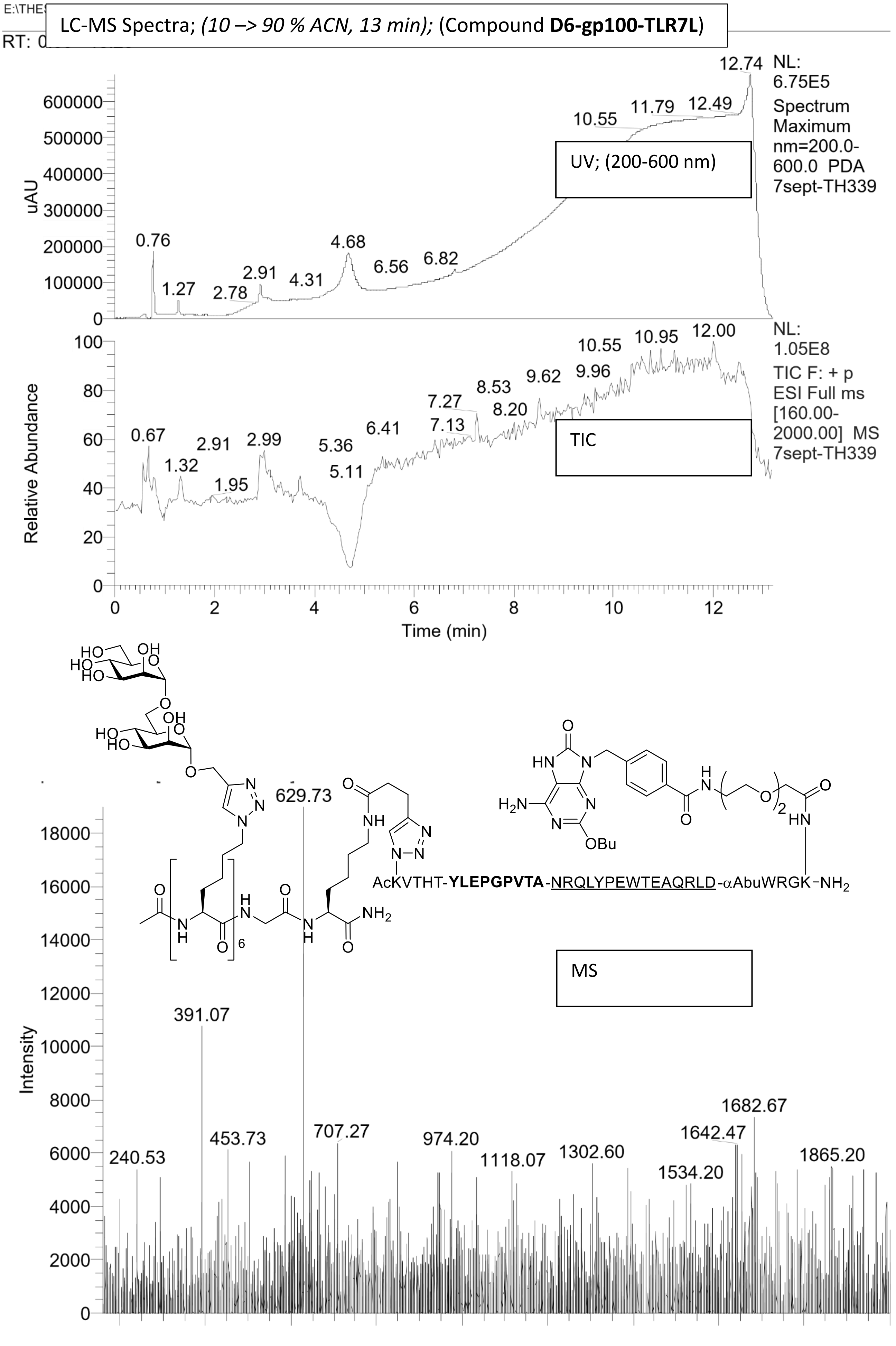

E6-gp100-TLR7L

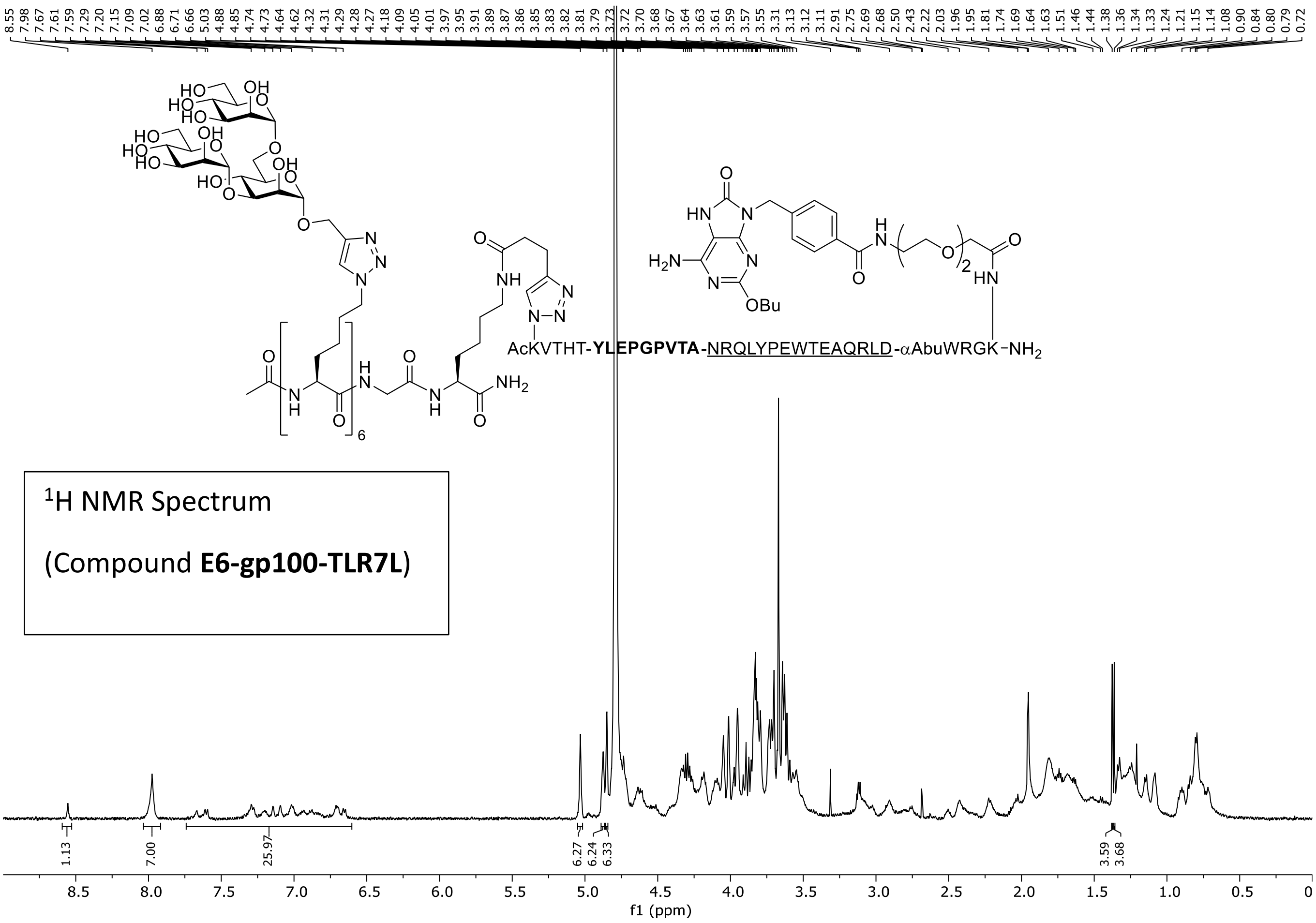

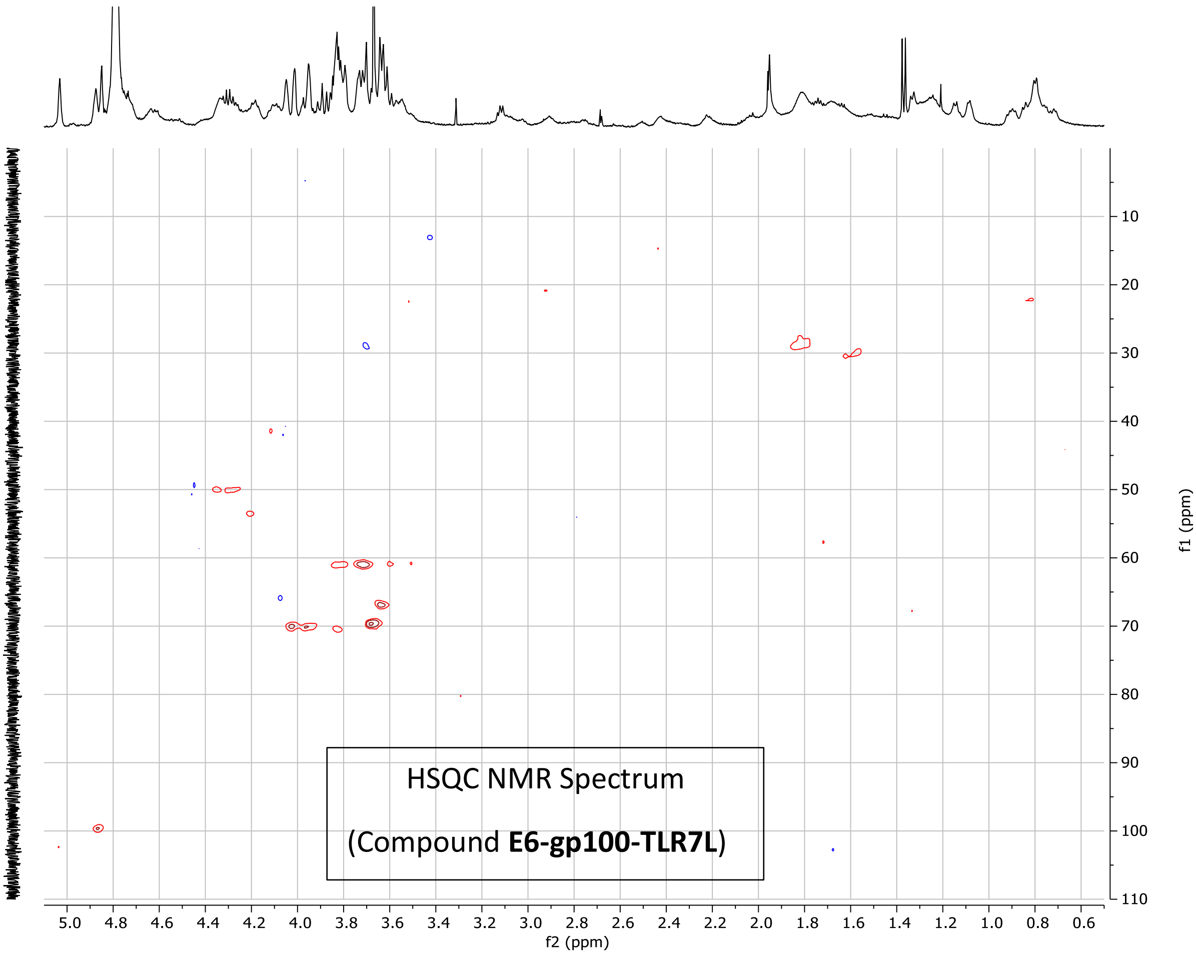

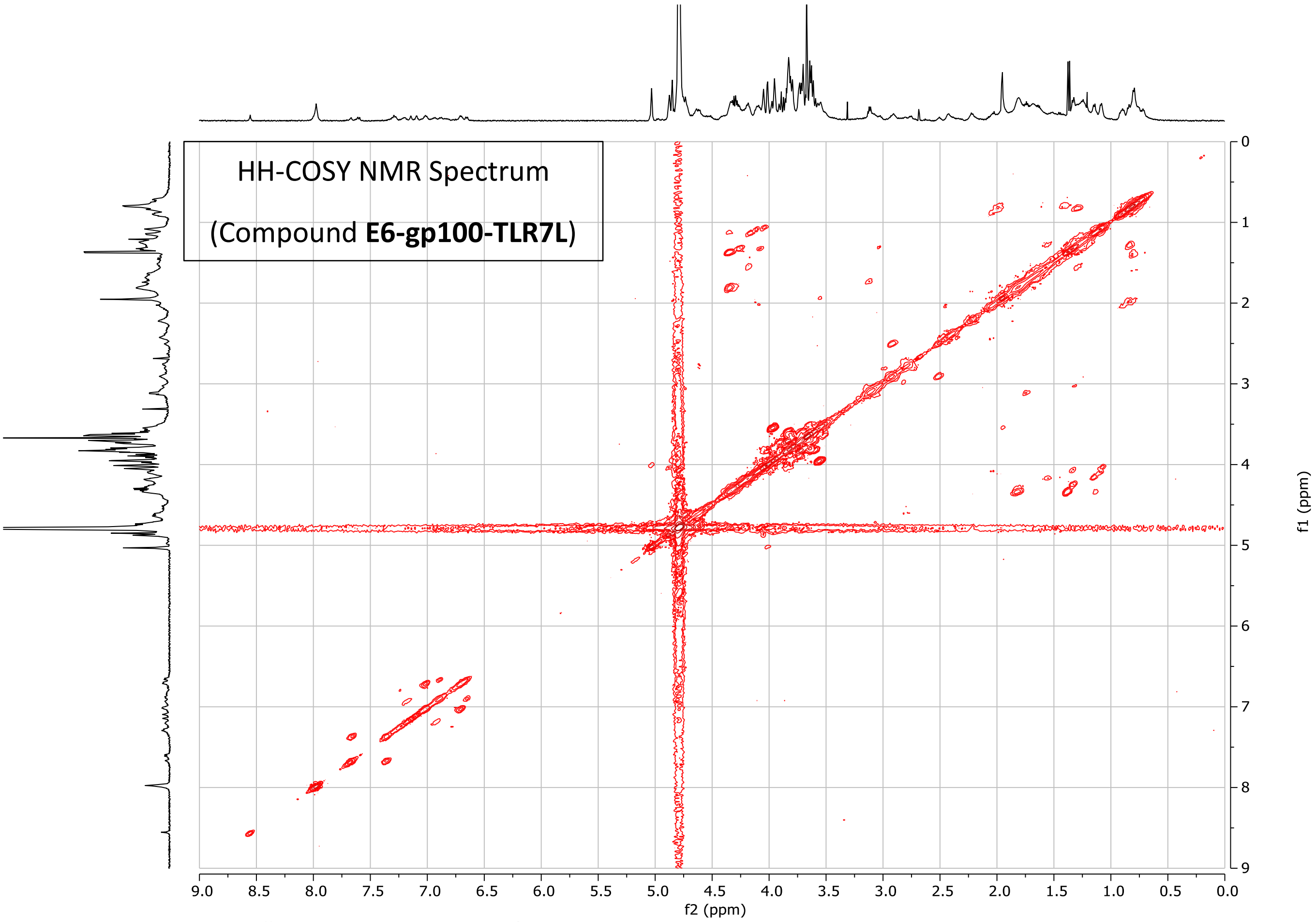
HH-COSY NMR Spectrum
(Compound **E6-gp100-TLR7L**)
f2 (ppm)
f1 (ppm)

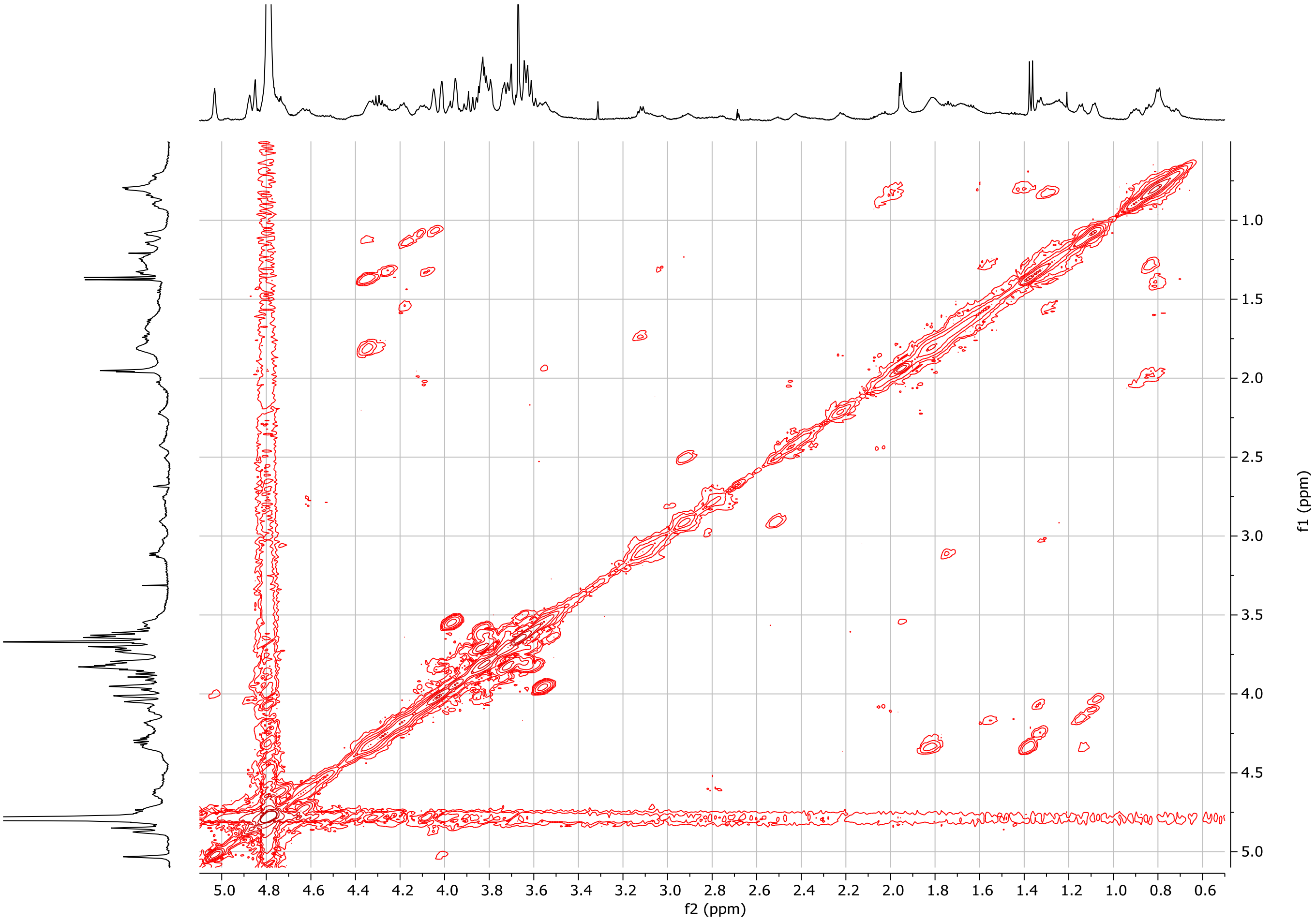
f2 (ppm)
f1 (ppm)

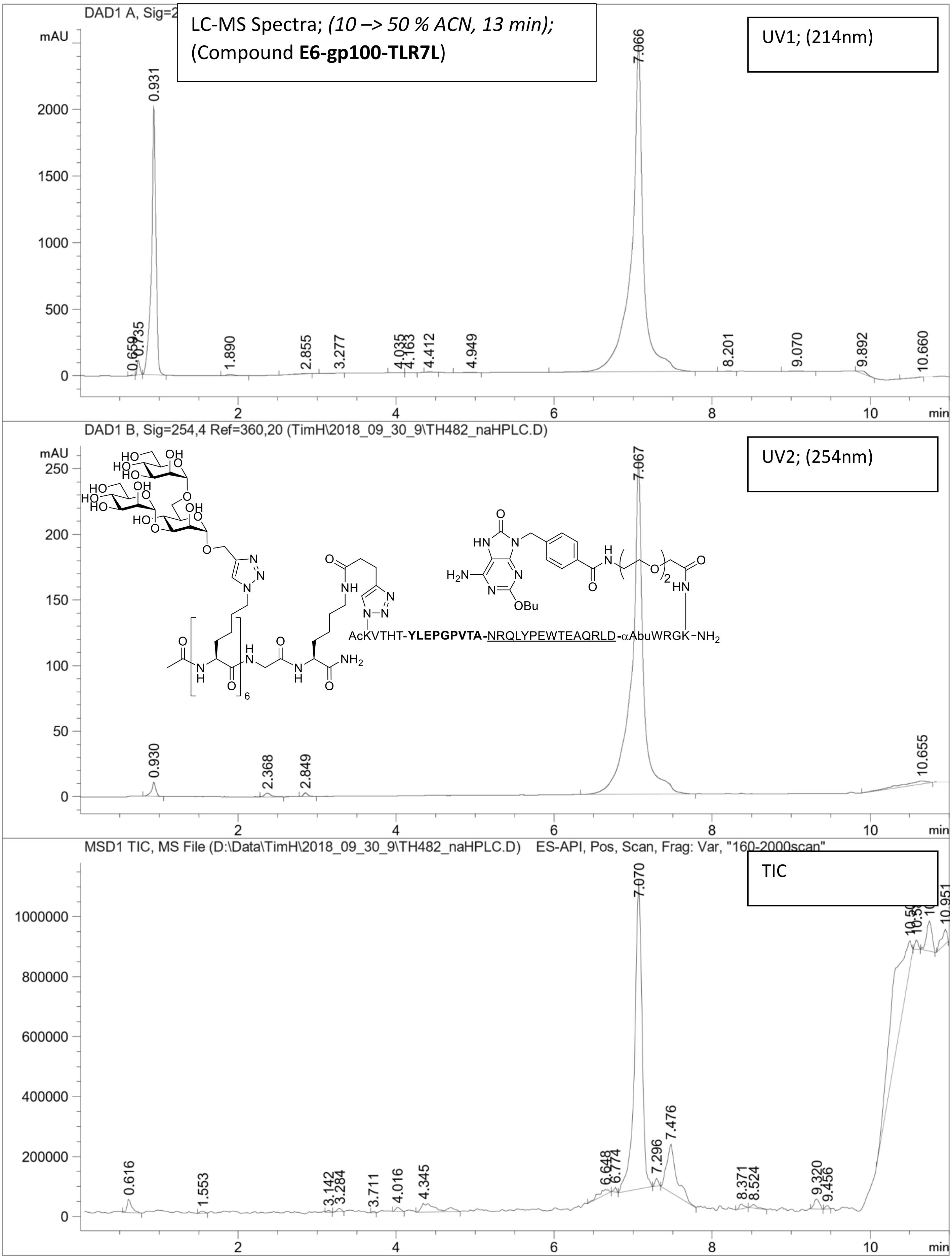
LC-MS Spectra; (10 –> 50 % ACN, 13 min);
(Compound E6-gp100-TLR7L)
UV1; (214nm)
DAD1 A, Sig=2
DAD1 B, Sig=254,4 Ref=360,20 (TimH\2018_09_30_9\TH482_naHPLC.D)
UV2; (254nm)
AcKVTHT-YLEPGPVTA-NRQLYPEWTEAQRLD-αAbuWRGK-NH2
MSD1 TIC, MS File (D:\Data\TimH\2018_09_30_9\TH482_naHPLC.D) ES-API, Pos, Scan, Frag: Var, "160-2000scan"
TIC
mAU
min

Sdasd

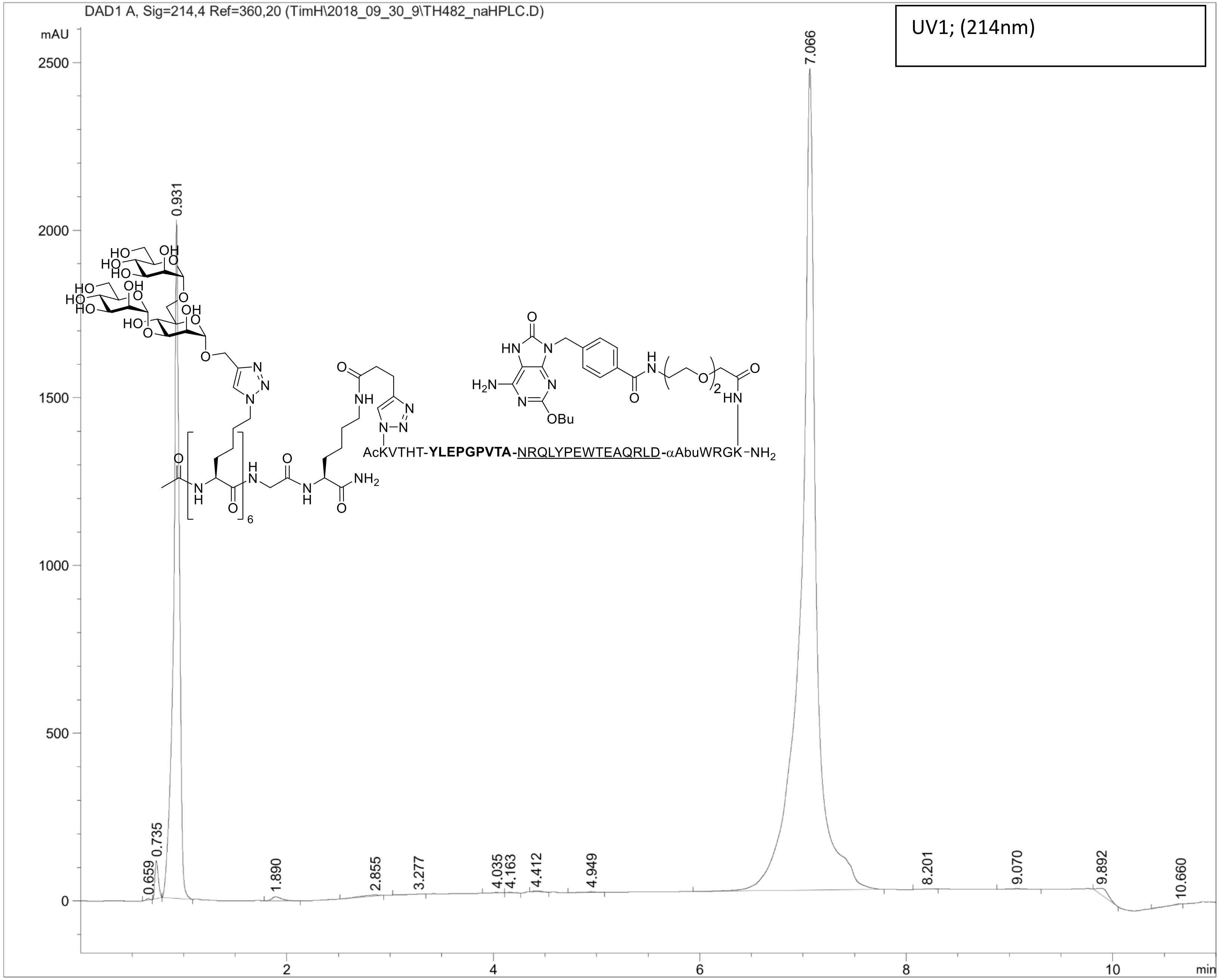
DAD1 A, Sig=214,4 Ref=360,20 (TimH\2018_09_30_9\TH482_naHPLC.D)
UV1; (214nm)
mAU
2500
2000
1500
1000
500
0
0.931
7.066
0.659
0.735
1.890
2.855
3.277
4.035
4.163
4.412
4.949
8.201
9.070
9.892
10.660
2
4
6
8
10
min
AcKVTHT-YLEPGPVTA-NRQLYPEWTEAQRLD-αAbuWRGK-NH2
OBu

Current Chromatogram(s)

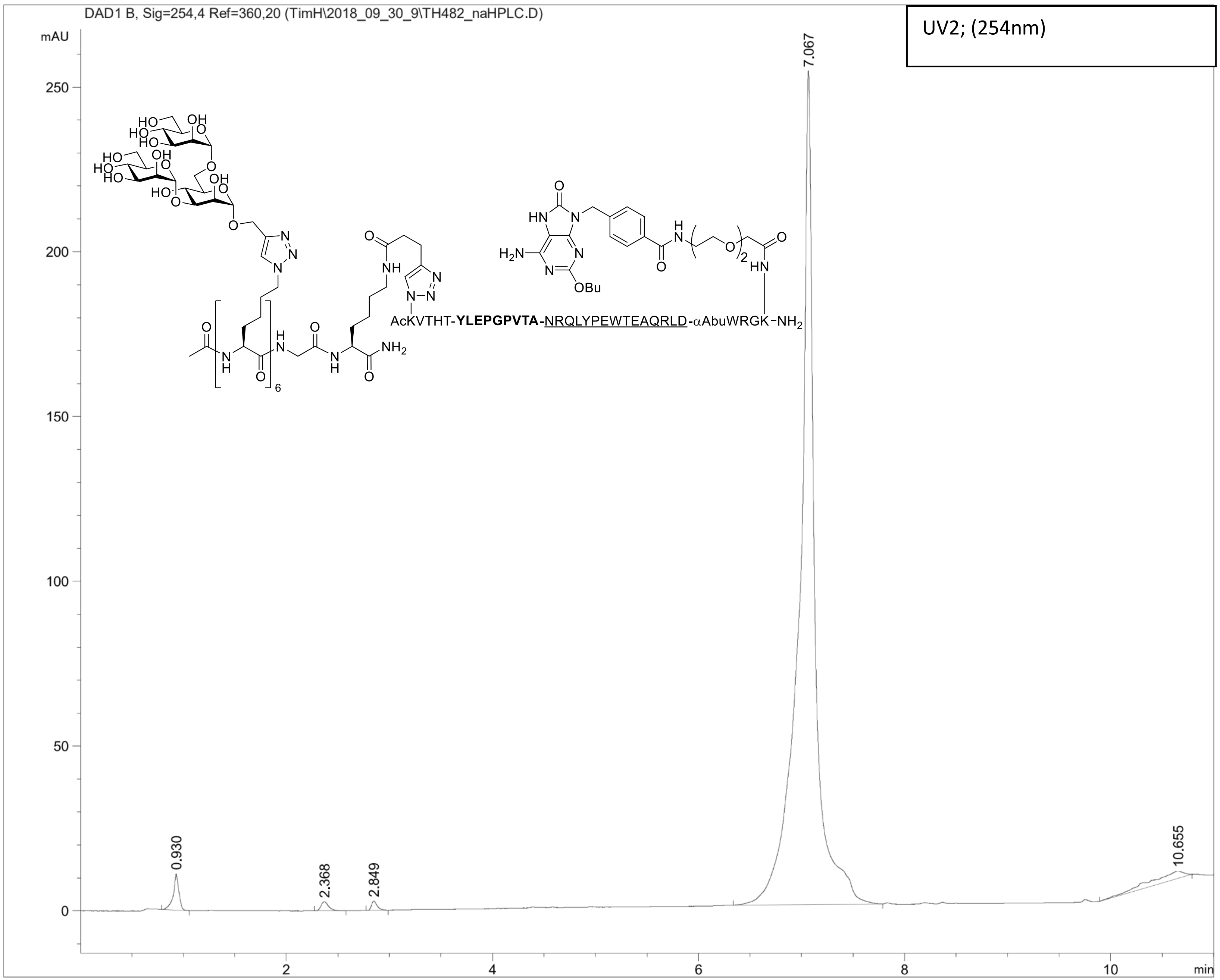

LC-MS_6120B 12/13/2018 12:20:27 PM SYSTEM Page 1 of 1)

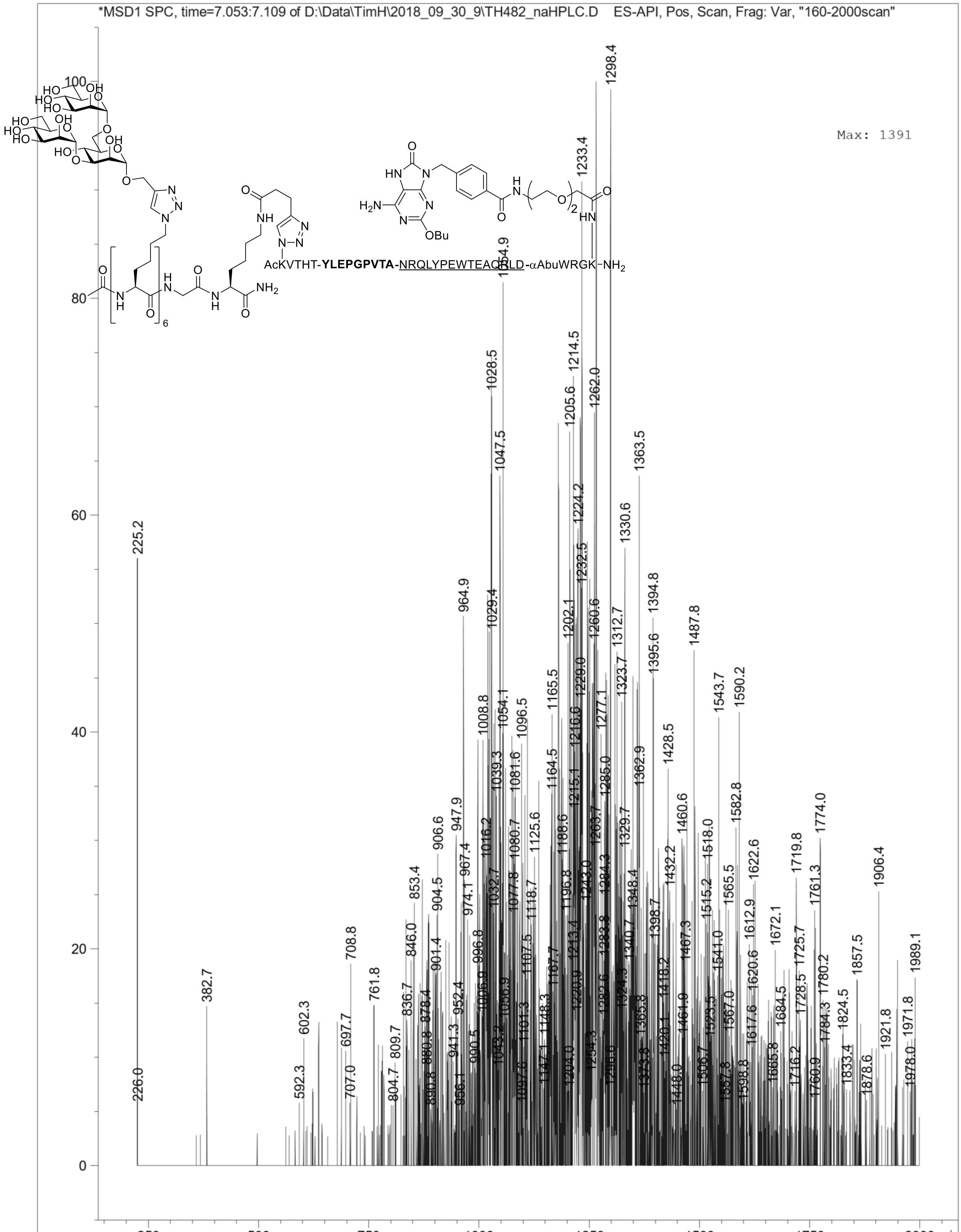
*MSD1 SPC, time=7.053:7.109 of D:\Data\TimH\2018_09_30_9\TH482_naHPLC.D ES-API, Pos, Scan, Frag: Var, "160-2000scan"
Max: 1391
AcKVTHT-YLEPGPVTA-NRQLYPEWTEACRLD-αAbuWRGK-NH2
OBu
m/z
